# Supplementary material for: Enyne diketones as substrate in asymmetric Nazarov cyclization for construction of chiral allene cyclopentenones
Source: Nat Commun. 2022 Jun 7;13:3146. doi: 10.1038/s41467-022-30846-y (PMC9174488; doi:10.1038/s41467-022-30846-y)
Supplement: Supplementary file 1 — Supplementary Information [file 41467_2022_30846_MOESM1_ESM.pdf]

Supplementary Information

Enyne diketones as substrate in asymmetric Nazarov cyclization for construction of chiral allene cyclopentenones

Shengbiao Tang [1]✉, Peng Zhang[1], Ying Shao[1] & Jiangtao Sun [1]✉

[1]Jiangsu Key Laboratory of Advanced Catalytic Materials & Technology, School of Petrochemical Engineering, Changzhou University, 1 Gehu Road, 213164 Changzhou, China

*E-mail: shengbiaotang@cczu.edu.cn; jtsun@cczu.edu.cn

Table of Contents

I. Supplementary Notes……………………………………………………2

II. Supplementary Methods.................... 2

Preparation of **Q9**...2

General procedure for the synthesis of 1,4-enyn-3-ols (**2**)………….……… .3

General procedure for preparation of allenols (**4-44**) and (-)-(**4-44**)…...… 6

Preparation of compound **3**……………….……………………………18

Preparation of compound **45-53**………………………………….....…19

III. Supplementary Discussion…………………………….…………24

Supplementary table 1. Optimization of the method A……….…...…...…24

Supplementary table 2. Screening of the chiral organocatalysts…….…....25

Supplementary Table 3. Optimazition of the asymmertric conditions…....26

Supplementary Fig. 1.Proposed mechanism of the alkyne-to-allene isomerization process……………………………………….……....27

Supplementary table 4. X-ray structures of (-)-**20**………...………...…28

Supplementary table 5. X-ray structures of compound **49**……………..…29

Supplementary table 6. X-ray structures of compound **53**……………..…30

NMR Spectra……………………………………………..………..…31

HPLC Chromatograms……………………………….……………170

IV. Supplementary References………………………….…...…………..171

I. Supplementary notes

All reactions were carried out using oven-dried glassware with magnetic stirring under argon atmosphere unless otherwise noted. Anhydrous solvents were dried prior to use. Reagents were purchased from Energy Chemical and used without further purification. For column chromatography, 200-300 mesh silica gel was used. Thin layer chromatography (TLC) was performed on Silicycle 250μm silica gel 60Å plates. Visualization was accomplished with UV light (254 nm), Iodine, or Potassium Permanganate.

^{1}H NMR and ^{13}C NMR spectra were recorded on a Bruker 300 MHz (300 MHz for ^{1}H; 282 MHz for ^{19}F; 75 MHz for ^{13}C) or 400 MHz (400 MHz for ^{1}H; 376 MHz for ^{19}F; 100 MHz for ^{13}C) spectrometers at ambient temperature. The chemical shifts (δ) are given in parts per million relative to $CDCl_3$ (7.26 ppm for ^{1}H) or TMS (0 ppm for ^{1}H) and $CDCl_3$ (77.16 ppm for ^{13}C). Coupling constants (*J*) are reported in Hz, and multiplicity is described using the following abbreviations: s = singlet, d = doublet, t = triplet, q = quartet, m = multiplet, br = broad, or combinations thereof. HRMS were performed on Agilent 6540 Q-TOF mass spectrometer (ESI). Melting points were determined on a SGW X-4B melting point apparatus.

All of diazo compounds were known compounds and prepared according to the literature procedures.[1-10] All of chiral organocatalysts were purchased and used as received. Optimal organocatalyst **Q9** was prepared according to the literature procedures[11-13], owing to a large amount of **Q9** was required.

II. Supplementary Methods

Procedure for the synthesis of Q9

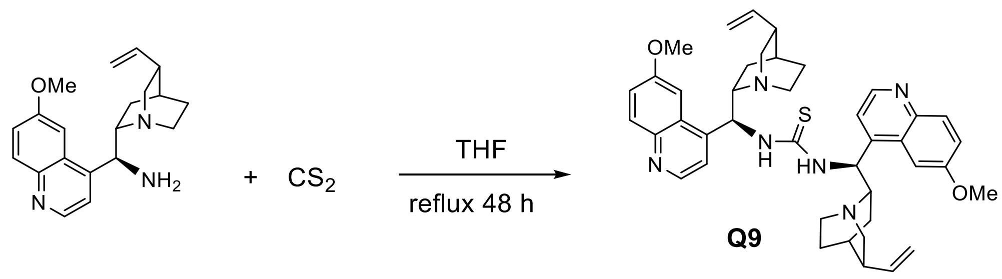

Q9 was synthesized according to the literature procedure[11-13]. CS2 (450μL, 2.5 mmol) was added to the free amine (4.8 g, 5.0 mmol) in dry THF (10.0 mL). The mixture solution turned to heterogeneous, and 30 mL additional dry THF was added. Then the mixture was reflux for 48 h. After cooling down, the reaction mixture was concentrated under reduced pressure. The residue was purified by flash chromatography (elution gradient: EtOAc/MeOH/Et3N = 100: 2: 3 to 100: 10: 3) to afford the desired product **Q9** (2.60 g, 50%) as a white solid. ^{1}H NMR (400 MHz, DMSO) δ 8.71 (s, 2H), 8.20 (brs, 2H), 7.92 (d, *J* = 9.1 Hz, 2H), 7.82 (s, 2H), 7.52-7.39 (m, 4H), 5.80-5.64 (m, 4H), 4.97-4.88 (m, 2H), 3.90 (s, 6H), 3.41 (s, 2H), 3.14-2.97 (m, 6H), 2.64-2.59 (m, 2H), 2.50-2.48 (m, 2H), 2.31-2.27 (m, 2H), 1.50-1.37 (m, 6H), 1.22-1.14 (m, 2H), 0.85-0.68 (m, 2H). ^{13}C NMR (100 MHz, DMSO) δ 182.3, 157.4, 147.9, 146.4, 144.5, 142.2, 131.6, 128.4, 121.7, 121.2, 114.7, 103.4, 59.9, 56.0, 55.4, 41.1, 39.1, 27.5, 26.8, 25.8. ^{1}H and 13C NMR were consistent with those reported literature [11].

General procedure for synthesis of enyn-3-ols

All of 1,4-enyne alcohols were prepared according to the literature procedures.[14-17]

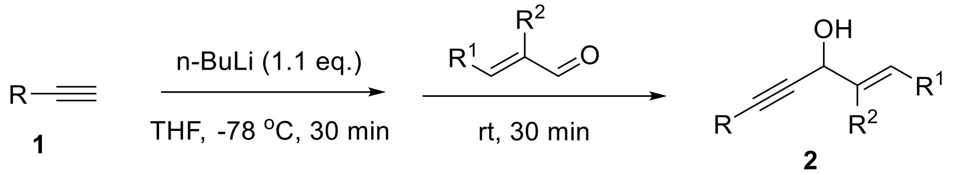

To a flame-dried roundbottom flask were added corresponding alkyne **1** (5.0 mmol, 1 equiv.) and THF (20 mL) under N_2. The temperature of the solution was dropped to -78 °C. Then n-BuLi (1.6 M solution in hexane, 5.5 mmol, 1.1 equiv) was added dropwise, and the reaction mixture was stirred at the same temperature for 30 min. The aldehyde **2** (5.0 mmol) was added, the solution warm to room temperature and stirred for another 30 min. The reaction was quenched with sat. NH_4Cl (40 mL) and diluted with CH_2Cl_2 (3*20 ml). The separated organic layer was washed with brine, dried over anhydrous Na_2SO_4 and concentrated to provide pure compound **2**. The characterization data of new compounds were summarized as follows:

(E)-4-methyl-1-(4-tert-butylphenyl)-hept-4-en-1-yn-3-ol (2c)

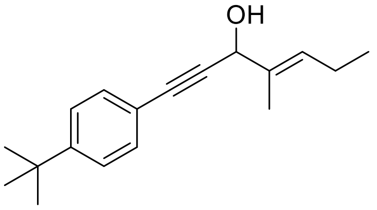

The title compound was prepared via general procedure, silica gel column chromatography (EtOAc/PE = 1/5), colorless oil (1.07 g, 84% yield). ^{1}H NMR (400 MHz, $CDCl_3$) δ 7.30 (d, *J* = 8.4 Hz, 2H), 7.23 (d, *J* = 8.4 Hz, 2H), 5.58 (t, *J* = 7.2 Hz, 1H), 4.88 (s, 1H), 2.12 (s, 1H), 2.00 (p, *J* = 7.4 Hz, 2H), 1.72 (s, 3H), 1.22 (s, 9H), 0.91 (t, *J* = 7.6 Hz, 3H). ^{13}C NMR (100 MHz, $CDCl_3$) δ 151.7, 133.8, 131.5, 130.2, 125.3, 119.7, 88.0, 86.1, 68.7, 34.8, 31.2, 21.2, 13.9, 12.3. HRMS (ESI): m/z calculated for $C_{18}H_{22}$ $[M-H_2O+H]^+$: 239.1794, found: 239.1787.

(E)-1-(4-methoxyphenyl)-4-methyl-hept-4-en-1-yn-3-ol (2d)

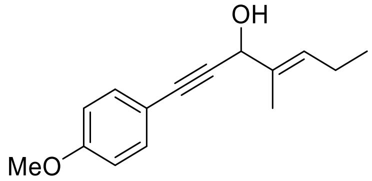

The title compound was prepared via general procedure, silica gel column chromatography (EtOAc/PE = 1/3), colorless oil (985 mg, 85% yield). ^{1}H NMR (400 MHz, $CDCl_3$) δ 7.38 (d, *J* = 8.8 Hz, 2H), 6.83 (d, *J* = 8.8 Hz,2H), 5.65 (t, *J* = 7.2 Hz, 1H), 4.95 (s, 1H), 3.80 (s, 3H), 2.12 (s, 1H, -OH), 2.10-2.04 (m, 2H), 1.81 (s, 3H), 1.00 (t, *J* = 7.5 Hz, 3H). ^{13}C NMR (100 MHz, $CDCl_3$) δ 159.7, 133.8, 133.3, 130.2, 114.8, 114.0, 87.2, 85.9, 68.74, 55.4, 21.2, 13.9, 12.3. HRMS (ESI): m/z calculated for $C_{15}H_{16}O$ $[M-H_2O+H]^+$: 213.1274, found: 213.1275.

(E)-methyl-(4-methylhept-4-en-1-yn-3-ol)-benzoate (2g)

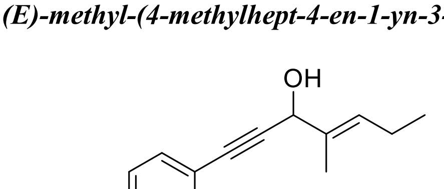

The title compound was prepared via general procedure, silica gel column chromatography (EtOAc/PE = 1/3), colorless oil (460 mg, 36% yield). ^{1}H NMR (400 MHz, $CDCl_3$) δ 7.94 (d, *J* = 8.4 Hz, 2H), 7.46 (d, *J* = 8.4 Hz, 2H), 7.38 (d, *J* = 8.4 Hz, 0.2H), 7.28 (d, *J* = 8.4 Hz, 0.2H), 5.64 (t, *J* = 7.2 Hz, 1H), 4.97 (s, 1H), 4.95 (s, 0.1H), 3.89 (s, 3H), 2.48 (s, 1H), 2.07 (p, *J* = 7.4 Hz, 2H), 1.80 (s, 3H), 0.98 (t, *J* = 7.6 Hz, 3H). ^{13}C NMR (100 MHz, $CDCl_3$) δ 166.7, 133.4, 131.7, 130.5, 129.6, 129.5, 127.5, 91.7, 85.0, 68.5, 52.3, 21.2, 13.8, 12.3. HRMS (ESI): m/z calculated for $C_{16}H_{18}NaO_3$ $[M+Na]^+$: 281.1148, found: 281.1144.

(E)-4-methyl-1-(4-phenylphenyl)-hept-4-en-1-yn-3-ol (2h)

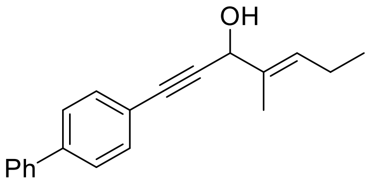

The title compound was prepared via general procedure, silica gel column chromatography (EtOAc/PE = 1/3), colorless oil (1.20 g, 87% yield). ^{1}H NMR (400 MHz, $CDCl_3$) δ 7.68-7.49 (m, 6H), 7.45 (t, J = 7.6 Hz, 2H), 7.36 (t, J = 7.2 Hz, 1H), 5.70 (t, J = 7.2 Hz, 1H), 5.01 (s, 1H), 2.15-2.08 (m, 3H, $-CH_2-$, -OH), 1.85 (s, 3H), 1.02 (t, J = 7.6 Hz, 3H). ^{13}C NMR (100 MHz, $CDCl_3$) δ 141.3, 140.4, 133.7, 132.3, 130.4, 129.0, 127.8, 127.13, 127.07, 121.6, 89.3, 85.9, 68.8, 21.2, 13.9, 12.3. HRMS (ESI): m/z calculated for $C_{20}H_{20}NaO$ $[M+Na]^+$: 299.1406, found: 299.1400.

(E)-1-(2-chlorophenyl)-4-methyl-hept-4-en-1-yn-3-ol (2j)

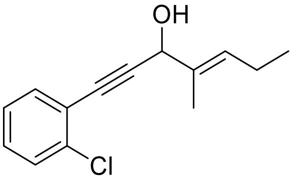

The title compound was prepared via general procedure, silica gel column chromatography (EtOAc/PE = 1/8), colorless oil (830 mg, 71% yield). ^{1}H NMR (400 MHz, $CDCl_3$) δ 7.47 (dd, J = 7.6, 1.6 Hz, 1H), 7.39-7.33 (m, 1H), 7.30-7.19 (m, 2H), 5.72 (t, J = 7.2 Hz, 1H), 5.01 (s, 1H), 2.24-2.00 (m, 3H, $-CH_2-$, -OH), 1.84 (s, 3H), 1.00 (t, J = 7.6 Hz, 3H). ^{13}C NMR (100 MHz, $CDCl_3$) δ 136.2, 133.6, 133.4, 130.7, 129.6, 129.3, 126.5, 122.7, 93.9, 82.8, 68.7, 21.2, 13.9, 12.4. HRMS (ESI): m/z calculated for $C_{14}H_{13}Cl$ $[M-H_2O+H]^+$: 217.0779, found: 217.0781.

(E)-4-methyl-1-(2-naphthyl)-hept-4-en-1-yn-3-ol (2k)

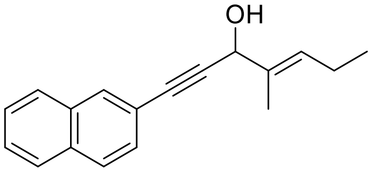

The title compound was prepared via general procedure, silica gel column chromatography (EtOAc/PE = 1/3), colorless oil (1.07 g, 85% yield). ^{1}H NMR (300 MHz, $CDCl_3$) δ 7.96 (d, J = 0.3 Hz, 1H), 7.83-7.68 (m, 3H), 7.57-7.35 (m, 3H), 5.79-5.62 (m, 1H), 5.02 (s, 1H), 2.19 (s, 1H, -OH), 2.10 (p, J = 7.5 Hz, 2H), 1.85 (d, J = 1.2 Hz, 3H), 1.01 (t, J = 7.5 Hz, 3H). ^{13}C NMR (75 MHz, $CDCl_3$) δ 133.7, 132.98, 132.93, 131.7, 130.4, 128.5, 128.0, 127.8, 126.8, 126.6, 120.0, 88.9, 86.3, 68.8, 21.2, 13.9, 12.3. HRMS (ESI): m/z calculated for $C_{18}H_{17}$ $[M-H_2O+H]^+$: 233.1325, found: 233.1322.

4-ethyl-1-phenylpent-4-en-1-yn-3-ol (2l)

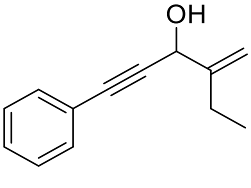

The title compound was prepared via general procedure, silica gel column chromatography (EtOAc/PE = 1/3), colorless oil (752 mg, 83% yield). ^{1}H NMR (300 MHz, $CDCl_3$) δ 7.52-7.38 (m, 2H), 7.34-7.27 (m, 3H), 5.40-5.14 (m, 1H), 5.06 (s, 1H), 5.01-4.89 (m, 1H), 2.30 (q, J = 7.4 Hz, 2H), 1.14 (t, J = 7.4 Hz, 3H). ^{13}C NMR (75 MHz, $CDCl_3$) δ 149.9, 131.8, 128.6, 128.4, 122.6, 110.5, 88.4, 85.9, 66.3, 24.6, 12.3. HRMS (ESI): m/z calculated for $C_{13}H_{13}$ $[M-H_2O+H]^+$: 169.1012, found: 169.1008.

(E)-8-methyl-undec-8-en-5-yn-7-ol (2m)

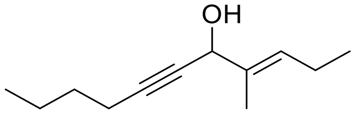

The title compound was prepared via general procedure, silica gel column chromatography (EtOAc/PE = 1/10), colorless oil (837 mg, 93% yield). ^{1}H NMR (400 MHz, $CDCl_3$) δ 5.56 (t, J = 7.2 Hz, 1H), 4.71 (s, 1H), 2.22 (td, J = 7.0, 2.0 Hz, 2H), 2.08-2.00 (m, 2H), 1.91 (d, J = 3.6 Hz, 1H, -OH), 1.72 (s, 3H), 1.53-1.44 (m, 2H), 1.43-1.35 (m, 2H), 0.96 (t, J = 7.6 Hz, 3H), 0.89 (t, J = 7.2 Hz, 3H). ^{13}C NMR (100 MHz, $CDCl_3$) δ 134.2, 129.7, 86.8, 79.6, 68.4, 30.8, 22.0, 21.1, 18.5, 13.9, 13.7, 12.1. HRMS (ESI): m/z calculated for $C_{12}H_{18}$ $[M-H_2O+H]^+$: 163.1481, found: 163.1477.

(E)-1-chloro-7-methyl-dec-7-en-4-yn-6-ol (2n)

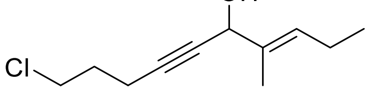

The title compound was prepared via general procedure, silica gel column chromatography (EtOAc/PE = 1/10), colorless oil (680 mg, 68% yield). ^{1}H NMR (400 MHz, $CDCl_3$) δ 5.53 (t, J = 7.2 Hz, 1H), 4.69 (s, 1H), 3.62 (t, J = 6.4 Hz, 2H),

2.41 (td, J = 6.8, 2.0 Hz, 2H), 2.11 (s, 1H, -OH), 2.06-1.99 (m, 2H), 1.97-1.91 (m, 2H), 1.70 (s, 3H), 0.95 (t, J = 7.6 Hz, 3H). ^{13}C NMR (100 MHz, $CDCl_3$) δ 133.9, 129.8, 84.5, 80.7, 68.2, 43.7, 31.3, 21.1, 16.3, 13.8, 12.0. HRMS (ESI) calculated for $C_{11}H_{16}Cl$ [M-H_2O+H]$^+$: 185.0906, found: 185.0902

(E)-2,2,6-trimethyl-non-6-en-3-yn-5-ol (2p)

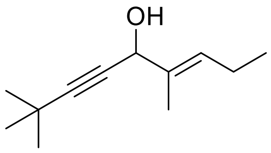

The title compound was prepared via general procedure, silica gel column chromatography (EtOAc/PE = 1/10), colorless oil (811 mg, 90% yield). ^{1}H NMR (400 MHz, $CDCl_3$) δ 5.55 (t, J = 7.0 Hz, 1H), 4.70 (s, 1H), 2.04 (p, J = 7.6 Hz, 2H), 1.85 (s, 1H), 1.71 (s, 3H), 1.21 (s, 9H), 0.97 (t, J = 7.6 Hz, 3H). ^{13}C NMR (100 MHz, $CDCl_3$) δ 134.2, 129.7, 95.1, 78.1, 68.3, 31.0, 27.5, 21.1, 13.9, 12.1. HRMS (ESI): m/z calculated for $C_{12}H_{20}NaO$ [M+Na]$^+$: 203.1406, found: 203.1406.

(E)-1,5-diphenyl-4-n-pentylpent-4-en-1-yn-3-ol (2u)

The title compound was prepared via general procedure, silica gel column chromatography (EtOAc/PE = 1/5), colorless

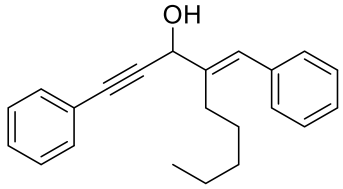

oil (1.30 g, 88% yield). ^{1}H NMR (300 MHz, $CDCl_3$) δ 7.47-7.44 (m, 2H), 7.37-7.18 (m, 8H), 6.85 (s, 1H), 5.22 (s, 1H), 2.48-2.43 (m, 3H, -CH_2-, -OH), 1.66-1.58 (m, 2H), 1.37-1.22 (m, 4H), 0.85 (t, J = 6.9 Hz, 3H). ^{13}C NMR (75 MHz, $CDCl_3$) δ 141.5, 137.3, 131.8, 128.8, 128.6, 128.4, 128.3, 127.4, 126.8, 122.6, 88.5, 86.5, 67.1, 32.2, 28.6, 22.5, 14.1. HRMS (ESI): m/z calculated for $C_{22}H_{22}$ [M-H_2O+H]$^+$: 287.1794, found: 287.1791.

(E)-4-methyl-1-phenylhex-4-en-1-yn-3-ol (2r)

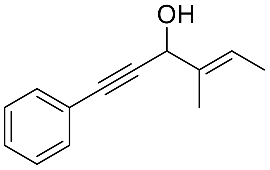

The title compound was prepared via general procedure, silica gel column chromatography (EtOAc/PE = 1/5), colorless oil (855 mg, 92% yield). ^{1}H NMR (300 MHz, $CDCl_3$) δ 7.51-7.34 (m, 2H), 7.34-7.19 (m, 3H), 5.86-5.63 (m, 1H), 4.97 (s, 1H), 2.56 (s, 1H), 1.81 (s, 3H), 1.65 (d, J = 6.9 Hz, 3H). ^{13}C NMR (75 MHz, $CDCl_3$) δ 135.0, 131.7, 128.4, 128.3, 122.76, 122.69, 88.6, 85.8, 68.5, 13.4, 12.1. The NMR was same with the reported literature.[14]

(E)-4-ethyl-1-phenyloct-4-en-1-yn-3-ol (2s)

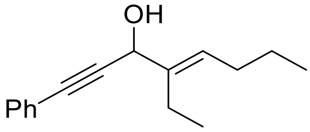

The title compound was prepared via general procedure, silica gel column chromatography (EtOAc/PE = 1/5), colorless oil (1.01 g, 88% yield). ^{1}H NMR (400 MHz, $CDCl_3$) δ 7.50-7.39 (m, 2H), 7.32-7.29 (m, 3H), 5.71 (t, J = 7.2 Hz, 1H), 5.03 (s, 1H), 2.28 (q, J = 7.6 Hz, 2H), 2.15 (s, 1H,-OH), 2.14-1.99 (m, 2H), 1.43 (dd, J = 14.8, 7.4 Hz, 2H), 1.12 (t, J = 7.6 Hz, 3H), 0.93 (t, J = 7.6 Hz, 3H). ^{13}C NMR (100 MHz, $CDCl_3$) δ 140.0, 131.7, 128.6, 128.4, 128.3, 122.8, 88.9, 85.9, 67.2, 29.7, 22.8, 20.9, 14.3, 14.0. HRMS (ESI): m/z calculated for $C_{16}H_{20}NaO$ [M+Na]$^+$: 251.1406, found: 251.1399.

(E)-1,7-Diphenyl-4-benzyl-hept-4-en-1-yn-3-ol (2t)

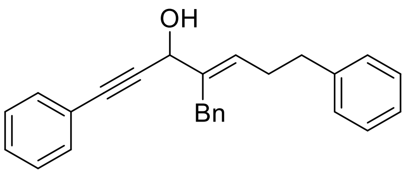

The title compound was prepared via general procedure, silica gel column chromatography (EtOAc/PE = 1/5), colorless oil (1.38 g, 78% yield). ^{1}H NMR (300 MHz, $CDCl_3$) δ 7.35-7.15 (m, 15H), 6.05 (t, J = 7.2 Hz, 1H), 4.93 (s, 1H), 3.70-3.48 (m, 2H), 2.72 (t, J = 7.5 Hz, 2H), 2.49 (dd, J = 15.0, 7.5 Hz, 2H). ^{13}C NMR (75 MHz, $CDCl_3$) δ 141.6, 139.6, 137.4, 131.7, 129.2, 128.63, 128.60, 128.53, 128.49, 128.4, 128.3, 126.15, 126.0, 122.6, 88.4, 86.7, 66.4, 35.6, 33.4, 30.2. HRMS (ESI): m/z calculated for $C_{26}H_{24}NaO$ [M+Na]$^+$: 375.1719, found: 375.1717.

General procedure for the synthesis and experiment data of (±) 4-44 and (-)-4-(-)43

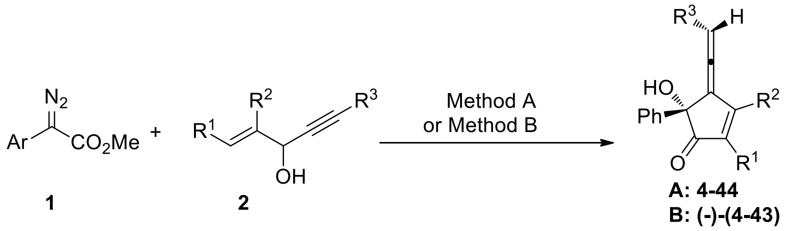

Method A (one-pot process for racemic products): To the tube was added freshly distilled hexane (2.0 mL), diazo **1** (0.3 mmol), enyn-3-ol **2** (0.2 mmol) and then $Rh_2(OAc)_4$ (2.6 mg, 2 mol%) was added to the mixture solution. The tube was sealed and stirred at rt for 12 h. And then MgO (400 mg, 50 eq.) and CH_2Cl_2 (5 mL) was added to the mixture solution, and stirred at 40 °C for another 3 h. Then the mixture solution was filtrated by diatomite, and wash with EtOAc (10*3). The filtrate was concentrated by rotary evaporation. The crude product was purified by silica gel column chromatography corresponding eluent to afford the desired products **(±) 4-44**.

Method B (for asymmetric version): To the dried tube was added freshly distilled hexane (2.0 mL), diazo **1** (0.3 mmol), enyn-3-ol **2** (0.2 mmol), and then $Rh_2(OAc)_4$ (2.6 mg, 2 mol%) was added to the mixture solution. The tube was sealed and stirred at rt. for 12 h. The solvent was removed directly, and then, the CPME (2.0 mL), **Q9** (15 mol%) and 4 ÅMs (40 mg) was added to the tube, stirred at rt. for another 72 h. The solution was concentrated under reduced pressure and purified by flash chromatography corresponding eluent to afford the desired products (-)-**4-43**.

***(-)4-(2-phenyl-1-enylidene)-2-ethyl-5-hydroxy-3-methyl-5-phenylcyclopent-2-en-1-one* ((-)-4)**

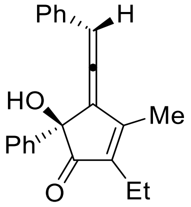

The compound **4** was prepared via method A, silica gel column chromatography (EtOAc/PE = 1:5), pale yellow solid (49.6 mg, 79% yield, dr > 19:1).

The compound **(-)-4** was prepared via method B, silica gel column chromatography (EtOAc/PE = 1:5), pale yellow solid (41.2 mg, 65% yield, 95% ee), m.p. 165-168 °C. The enantiomeric excess was determined by HPLC analysis on a Daicel Chiralpak IC column (hexane/iPrOH = 70:30, flow rate: 1.0 mL/min, λ= 254 nm, t_R(major) = 7.67 min, t_R (minor) = 10.17 min. $[\alpha]_D^{25}$ = - 331.3 (c = 0.25 in $CHCl_3$). ^{1}H NMR (400 MHz, $CDCl_3$) δ 7.47-7.42 (m, 2H), 7.34-7.25 (m, 3H), 7.20-7.17 (m, 3H), 6.94-6.92 (m, 2H), 6.86 (s, 1H), 3.23 (s, 1H), 2.58-2.34 (m, 2H), 2.17 (s, 3H), 1.16 (t, J = 7.6 Hz, 3H). ^{13}C NMR (100 MHz, $CDCl_3$) δ 204.0, 202.9, 162.4, 141.3, 141.1, 132.8, 128.7, 128.5, 128.0, 127.9, 127.3, 125.2, 118.4, 103.3, 78.9, 17.2, 13.9, 12.9. HRMS (ESI) m/z calculated for $C_{22}H_{20}NaO_2$ $[M+Na]^+$: 339.1356, found: 339.1360.

4-(2-(4-methylphenyl)-1-enylidene)-2-ethyl-5-hydroxy-3-methyl-5-phenyl-cyclopent-2-en-1-one ((-)-5).

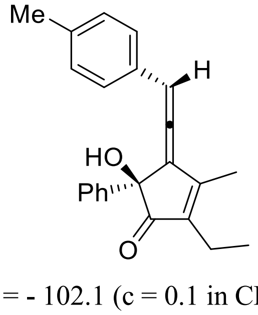

The compound **5** was prepared via method A, silica gel column chromatography (EtOAc/PE = 1:5), pale yellow solid (53.7 mg, 76% yield, dr > 19:1).

The compound **(-)-5** was prepared via method B, silica gel column chromatography (EtOAc/PE = 1:5), pale yellow solid (40.2 mg, 61% yield, 90% ee), m.p. 160-162 °C. The enantiomeric excess was determined by HPLC analysis on a Daicel Chiralpak IC column (hexane/iPrOH = 70:30, flow rate: 1.0 mL/min, λ= 254 nm, t_R(major) = 8.64 min, t_R (minor) = 11.22 min. $[\alpha]_D^{25}$ = - 102.1 (c = 0.1 in $CHCl_3$).^{1}H NMR (400 MHz, $CDCl_3$) δ 7.43 (dd, J = 7.8, 1.5 Hz, 2H), 7.33-7.24 (m, 4H), 6.97 (d, J = 7.8 Hz, 1H), 6.82 (s, 1H), 6.80 (d, J = 8.1 Hz, 2H), 3.09 (s, 1H), 2.55-2.31 (m, 2H), 2.28 (s, 3H), 2.13 (s, 3H), 1.13 (t,

J = 7.6 Hz, 3H). ^{13}C NMR (75 MHz, $CDCl_3$) δ 204.1, 202.7, 162.6, 141.3, 141.0, 138.0, 129.8, 129.5, 128.5, 128.0, 127.2, 125.2, 118.4, 103.2, 78.9, 21.3, 17.2, 13.9, 12.9. HRMS (ESI) m/z calculated for $C_{23}H_{22}NaO_2$ [M+Na]$^+$: 353.1512, found: 353.1516.

4-(2-(4-tert-butylphenyl)-1-enylidene)-2-ethyl-5-hydroxy-3-methyl-5-phenylcyclopent-2-en-1-one ((-)-6)

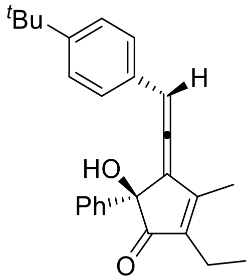

The compound **6** was prepared via method A, silica gel column chromatography (EtOAc/PE = 1:6), pale yellow solid (61.2 mg, 82% yield, dr > 19:1).

The compound **(-)-6** was prepared via method B, silica gel column chromatography (EtOAc/PE = 1:6), pale yellow solid (49.1 mg, 66% yield, 93% ee), m.p. 173-175 °C. The enantiomeric excess was determined by HPLC analysis on a Daicel Chiralpak IC column (hexane/iPrOH = 70:30, flow rate: 1.0 mL/min, λ= 254 nm, t_R(major) = 7.86 min, t_R (minor) = 11.02 min. $[\alpha]_D^{25}$ = - 78.4 (c = 0.5 in $CHCl_3$). ^{1}H NMR (400 MHz, $CDCl_3$) δ 7.43 (dd, J = 8.0, 1.6 Hz, 2H), 7.34-7.26 (m, 3H), 7.18 (d, J = 8.4 Hz, 2H), 6.86 (d, J = 8.4 Hz, 2H), 6.82 (s, 1H), 3.20 (s, 1H), 2.51-2.33 (m, 2H), 2.13 (s, 3H), 1.26 (s, 9H), 1.12 (t, J = 7.6 Hz, 3H). ^{13}C NMR (100 MHz, $CDCl_3$) δ 204.0, 202.8, 162.6, 151.2, 141.3, 140.9, 129.9, 128.6, 128.0, 127.1, 125.7, 125.2, 118.3, 103.0, 78.9, 34.7, 31.3, 17.2, 13.8, 12.9. HRMS (ESI) calculated for $C_{26}H_{28}NaO_2$ [M+Na]$^+$: 395.1982, found: 395.1992.

4-(2-(4-methoxyphenyl)-1-enylidene)-2-ethyl-5-hydroxy-3-methyl-5-phenyl-cyclopent-2-en-1-one ((-)-7).

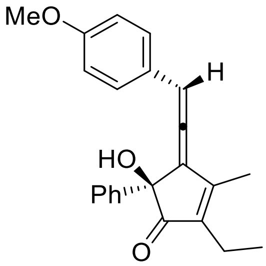

The compound **7** was prepared via method A, silica gel column chromatography (EtOAc/PE = 1:4), pale yellow solid (58.2 mg, 78% yield, dr > 19:1).

The compound **(-)-7** was prepared via method B, silica gel column chromatography (EtOAc/PE = 1:4), pale yellow solid (41.6 mg, 60% yield, 96% ee), m.p. 144-146 °C. The enantiomeric excess was determined by HPLC analysis on a Daicel Chiralpak IC column (hexane/iPrOH = 60:40, flow rate: 1.0 mL/min, λ= 254 nm, t_R(major) = 9.04 min, t_R (minor) = 12.40 min. $[\alpha]_D^{25}$ = - 130.3 (c = 0.1 in $CHCl_3$).

^{1}H NMR (400 MHz, $CDCl_3$) δ 7.43-7.40 (m, 2H), 7.33-7.22 (m, 3H), 6.90-6.75 (m, 3H), 6.75-6.63 (m, 2H), 3.75 (s, 3H), 3.20 (s, 1H), 2.56-2.34 (m, 2H), 2.13 (s, 3H), 1.13 (t, J = 7.6 Hz, 3H). ^{13}C NMR (100 MHz, $CDCl_3$) δ 204.1, 202.5, 162.6, 159.5, 141.4, 140.9, 128.5, 128.5, 128.0, 125.2, 124.9, 118.4, 114.2, 102.8, 78.8, 55.4, 17.2, 13.8, 12.9. HRMS (ESI) calculated for $C_{23}H_{22}NaO_3$ [M+Na]$^+$: 369.1461, found: 369.1460.

4-(2-(4-Fluorophenyl)-1-enylidene)-2-ethyl-5-hydroxy-3-methyl-5-phenyl-cyclopent-2-en-1-one ((-)-8).

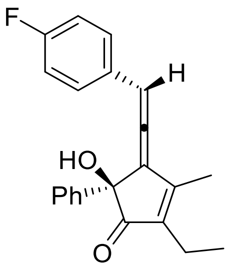

The compound **8** was prepared via method A, silica gel column chromatography (EtOAc/PE = 1:5), pale yellow solid (48.5 mg, 71% yield, dr > 19:1).

The compound (-)-**8** was prepared via method B, silica gel column chromatography (EtOAc/PE = 1:5), pale yellow solid (41.3 mg, 62% yield, 93% ee), m.p. 85-87 °C. The enantiomeric excess was determined by HPLC analysis on a Daicel Chiralpak IC column (hexane/iPrOH = 60:40, flow rate: 1.0 mL/min, λ= 254 nm, t_R(major) = 6.38 min, t_R (minor) = 8.37 min. $[\alpha]_D^{25}$ = - 180.0 (c = 0.2 in $CHCl_3$). ^{1}H NMR (300 MHz, $CDCl_3$) δ 7.43-7.37 (m, 2H), 7.33-7.27 (m, 3H), 6.83 (d, J = 7.0 Hz, 4H), 6.81 (s, 1H), 3.16 (s, 1H), 2.58-2.33 (m, 2H), 2.14 (s, 3H), 1.14 (t, J = 7.6 Hz, 3H). ^{19}F NMR (282 MHz, $CDCl_3$) δ -113.64. ^{13}C NMR (75 MHz, $CDCl_3$) δ 204.0, 202.7 (d, J = 2.4 Hz), 162.5 (d, J = 247.7 Hz), 162.2, 141.4 (d, J = 3.3 Hz), 128.84, 128.78, 128.73, 128.6, 128.1, 125.1, 118.7, 115.7 (d, J = 21.8 Hz), 102.4, 78.8, 17.22, 13.89, 12.93. HRMS (ESI) m/z calculated for $C_{22}H_{19}FO_2Na$ [M+H]$^+$: 357.1261, found: 357.1256.

4-(2-(4-bromophenyl)-1-enylidene)-2-ethyl-5-hydroxy-3-methyl-5-phenylcyclopent-2-en-1-one ((-)-9)

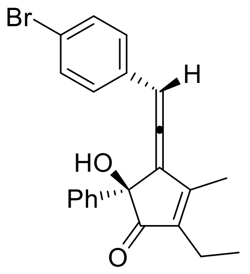

The compound **9** was prepared via method A, silica gel column chromatography (EtOAc/PE = 1:5), pale yellow solid (50.0 mg, 63% yield, dr > 19:1).

The compound (-)-**9** was prepared via method B, silica gel column chromatography (EtOAc/PE = 1:5), pale yellow solid (40.7 mg, 52% yield, 90% ee), m.p. 181-182 °C. The enantiomeric excess was determined by HPLC analysis on a Daicel Chiralpak IC column (hexane/iPrOH = 70:30, flow rate: 1.0 mL/min, λ= 254 nm, t_R(major) = 8.43 min, t_R (minor) = 11.34 min. $[\alpha]_D^{18}$ = - 252.1 (c = 0.1 in $CHCl_3$). ^1H NMR (300 MHz, $CDCl_3$) δ 7.45-7.33 (m, 2H), 7.33-7.22 (m, 5H), 6.77 (s, 1H), 6.75-6.68 (m, 2H), 3.16 (s, 1H), 2.62-2.30 (m, 2H), 2.13 (s, 3H), 1.14 (t, J = 7.6 Hz, 3H). ^{13}C NMR (75 MHz, $CDCl_3$) δ 203.9, 203.1, 162.0, 141.6, 141.3, 131.9, 128.7, 128.6, 128.2, 127.3, 125.1, 121.8, 118.8, 102.5, 78.9, 17.2, 13.9, 12.9. HRMS (ESI) m/z calculated for $C_{22}H_{19}BrNaO_2$ $[M+Na]^+$: 417.0461, 419.0440 found: 417.0457, 419.0442.

4-(2-(4-methoxycarbonylphenyl)-1-enylidene)-2-ethyl-5-hydroxy-3-methyl-5-phenylcyclopent-2-en-1-one ((-)-10)

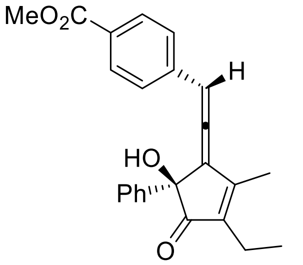

The compound **10** was prepared via method A, silica gel column chromatography (EtOAc/PE = 1:3), pale yellow solid (37.6 mg, 50% yield, dr > 19:1).

The compound (-)-**10** was prepared via method B, silica gel column chromatography (EtOAc/PE = 1:3), pale yellow solid (36.0 mg, 48% yield, 87% ee), m.p. 165-168 °C. The enantiomeric excess was determined by HPLC analysis on a Daicel Chiralpak IC column (hexane/iPrOH = 60:40, flow rate: 1.0 mL/min, λ= 254 nm, t_R(major) = 8.67 min, t_R (minor) = 11.32 min. $[\alpha]_D^{18}$ = - 96.6 (c = 0.1 in $CHCl_3$). ^1H NMR (300 MHz, $CDCl_3$) δ 7.85-7.70 (m, 2H), 7.47-7.37 (m, 2H), 7.31-7.27 (m, 3H), 6.95-6.88 (m, 2H), 6.86 (s, 1H), 3.88 (s, 3H), 3.16 (s, 1H), 2.54-2.31 (m, 2H), 2.15 (s, 3H), 1.15 (t, J = 7.6 Hz, 3H). ^{13}C NMR (75 MHz, $CDCl_3$) δ 204.0, 203.8, 166.8, 161.8, 141.8, 141.2, 137.8, 130.0, 129.4, 128.7, 128.3, 127.1, 125.0, 118.7, 102.8, 79.1, 52.26, 17.3, 13.9, 12.9. HRMS (ESI) calculated for $C_{24}H_{23}O_4$ $[M+H]^+$: 375.1596, found: 375.1596.

4-(2-(4-biphenyl)-1-enylidene)-2-ethyl-5-hydroxy-3-methyl-5-phenylpent-2-en-1-one ((-)-11)

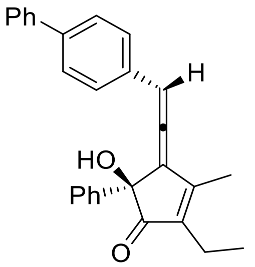

The compound **11** was prepared via method A, silica gel column chromatography (EtOAc/PE = 1:5), pale yellow solid (58.9 mg, 75% yield, dr > 19:1).

The compound (-)-**11** was prepared via method B, silica gel column chromatography (EtOAc/PE = 1:5), pale yellow solid (49.2 mg, 63% yield, 90% ee), m.p. 159-162 °C. The enantiomeric excess was determined by HPLC analysis on a Daicel Chiralpak IC column (hexane/iPrOH = 70:30, flow rate: 1.0 mL/min, λ= 254 nm, t_R(major) = 9.63 min, t_R (minor) = 13.40 min. $[\alpha]_D^{25}$ = -96.5 (c = 0.2 in CHCl3). ^1H NMR (400 MHz, $CDCl_3$) δ 7.60-7.49 (m, 2H), 7.48-7.36 (m, 6H), 7.36-7.19 (m, 4H), 6.96 (d, J = 8.2 Hz, 2H), 6.88 (s, 1H), 3.21 (s, 1H), 2.54-2.37 (m, 1H), 2.16 (s, 3H), 1.14 (t, J = 7.6 Hz, 3H). ^{13}C NMR (75 MHz, $CDCl_3$) δ 204.0, 203.2, 162.3, 141.4, 141.2, 140.8, 140.6, 131.8, 128.9, 128.6, 128.1, 127.7, 127.55, 127.46, 127.0, 125.2, 118.5, 103.0, 79.0, 17.2, 13.9, 12.9. HRMS (ESI) m/z calculated for $C_{28}H_{24}NaO_2$ $[M+Na]^+$: 415.1669, found: 415.1676.

3-(2-(3-chlorophenyl)-1-enylidene)-2-ethyl-5-hydroxy-3-methyl-5-phenylcyclopent-2-en-1-one ((-)-12)

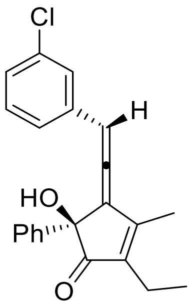

The compound **12** was prepared via method A, silica gel column chromatography (EtOAc/PE = 1:5), white solid (56.7 mg, 81% yield, dr > 19:1).

The compound (-)-**12** was prepared via method B, silica gel column chromatography (EtOAc/PE = 1:5), (49.0 mg, 70% yield, 93% ee), m.p. 165-166 °C, The enantiomeric excess was determined by HPLC analysis on a Daicel Chiralpak IC column (hexane/iPrOH = 70:30, flow rate: 1.0 mL/min, λ= 254 nm, t_R(major) = 5.59 min, t_R (minor) = 7.38 min. $[\alpha]_D^{25}$ = - 330.1 (c = 0.1 in $CHCl_3$). ^1H NMR (400 MHz, $CDCl_3$) δ 7.44-7.41 (m, 2H), 7.35-7.28 (m, 3H), 7.16-7.06 (m, 2H), 6.81-6.79 (m, 3H), 3.29 (s, 1H), 2.56-2.35 (m, 2H), 2.16 (s, 3H), 1.16 (t, *J* = 7.6 Hz, 3H). ^{13}C NMR (100 MHz, $CDCl_3$) δ 203.9, 203.2, 161.9, 141.6, 141.2, 134.8, 134.7, 129.9, 128.7, 128.3, 127.9, 126.9, 125.4, 124.9, 118.9, 102.4, 79.0, 17.2, 13.9, 12.9. HRMS (ESI) m/z calculated for $C_{22}H_{20}ClO_2$ $[M+H]^+$: 351.1152, found: 351.1150.

4-(2-(2-chlorophenyl)-1-enylidene)-2-ethyl-5-hydroxy-3-methyl-5-phenylcyclopent-2-en-1-one ((-)-13).

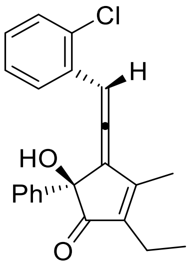

The compound **13** was prepared via method A, silica gel column chromatography (EtOAc/PE = 1:5), white solid (51.1 mg, 73% yield, dr > 19:1).

The compound (-)-**13** was prepared via method B, silica gel column chromatography (EtOAc/PE = 1:5), white solid (41.7 mg, 60% yield, 84% ee), m.p. 103-105 °C. The enantiomeric excess was determined by HPLC analysis on a Daicel Chiralpak IC column (hexane/iPrOH = 70:30, flow rate: 1.0 mL/min, λ= 254 nm, t_R(major) = 6.82 min, t_R (minor) = 8.70 min. $[\alpha]_D^{25}$ = - 302.1 (c = 0.2 in $CHCl_3$). ^1H NMR (300 MHz, $CDCl_3$) δ 7.42-7.38 (m, 2H), 7.36-7.24 (m, 5H), 7.10 -7.01 (m, 1H), 6.89 (td, *J* = 7.5, 1.1 Hz, 1H), 6.57 (dd, *J* = 7.8, 1.6 Hz, 1H), 3.22 (s, 1H), 2.49-2.38 (m, 1H), 2.16 (s, 3H), 1.14 (t, *J* = 7.6 Hz, 3H). ^{13}C NMR (75 MHz, $CDCl_3$) δ 203.9, 203.7, 162.2, 141.4, 141.2, 132.5, 130.7, 129.9, 129.0, 128.7, 128.6, 128.1, 126.9, 125.1, 118.5, 99.7, 79.0, 17.2, 14.0, 12.9. HRMS (ESI) m/z calculated for $C_{22}H_{19}ClNaO_2$ $[M+Na]^+$: 373.0966, found: 373.0968.

4-(2-(2-naphthylphenyl)-1-enylidene)-2-ethyl-5-hydroxy-3-methyl-5-phenylcyclopent-2-en-1-one ((-)-14)

The compound **14** was prepared via method A, silica gel column chromatography (EtOAc/PE = 1:5), pale yellow solid (47.8 mg, 65% yield, dr > 19:1).

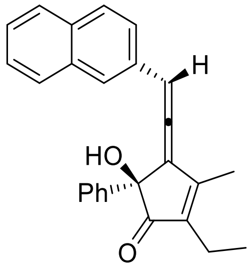

The compound (-)-**14** was prepared via method B, silica gel column chromatography (EtOAc/PE = 1:5), pale yellow solid (40.3 mg, 55% yield, 92% ee), m.p. 133-134 °C. The enantiomeric excess was determined by HPLC analysis on a Daicel Chiralpak IC column (hexane/iPrOH = 70:30, flow rate: 1.0 mL/min, λ= 254 nm, t_R(major) = 8.39 min, t_R (minor) = 11.53 min. $[\alpha]_D^{18}$ = - 231.0 (c = 0.2 in $CHCl_3$). ^1H NMR (300 MHz, $CDCl_3$) δ 7.75-7.67 (m, 1H), 7.68-7.59 (m, 1H), 7.56 (d, *J* = 8.4 Hz, 1H), 7.50-7.37 (m, 5H), 7.36-7.27 (m, 3H), 7.01 (s, 1H), 6.88 (dd, *J* = 8.4, 1.8 Hz, 1H), 3.24 (brs, 1H), 2.60-2.31 (m, 2H), 2.16 (s, 3H), 1.16 (t, *J* = 7.5 Hz, 3H). ^{13}C NMR (75 MHz, $CDCl_3$) δ 204.1, 203.6, 162.4, 141.5, 141.3, 133.6, 133.1, 130.3, 128.6, 128.4, 128.1, 127.9, 127.8, 126.54, 126.46, 126.23, 125.2, 124.8, 118.6, 103.7, 78.9, 17.2, 13.9, 12.9. HRMS (ESI) m/z calculated for $C_{26}H_{22}NaO_2$ $[M+Na]^+$: 389.1512, found: 389.1520.

4-(2-butyl-1-enylidene)-2-ethyl-5-hydroxy-3-methyl-5-phenylpent-2-en-1-one ((-)-15)

The compound **15** was prepared via method A, silica gel column chromatography (EtOAc/PE/tBuOMe = 1:5:1), (42.9 mg, 72% yield, dr > 19:1).

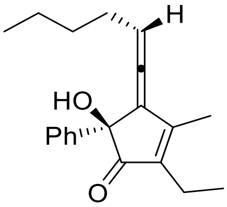

The compound (-)-**15** was prepared via modified method B (step 1 same with standard procedure; step 2 for 96 h, and then was added MgO (200 mg) stirred for another 6 h), silica gel column chromatography (EtOAc/PE/^{t}BuOMe = 1:5:1), yellow oil (32.1 mg, 54% yield, 85% ee). The enantiomeric excess was determined by HPLC analysis on a Daicel Chiralpak IC column (hexane/iPrOH = 60:40, flow rate: 1.0 mL/min, λ= 254 nm, t_R(major) = 6.65 min, t_R (minor) = 9.89 min. $[\alpha]_D^{25}$ = - 4.0 (c = 0.1 in $CHCl_3$).^{1}H NMR (300 MHz, $CDCl_3$) δ 7.40-7.34 (m, 2H), 7.33-7.21 (m, 3H), 5.88 (t, J = 6.6 Hz, 1H), 3.09 (s, 1H), 2.47-2.26 (m, 2H), 2.13 (s, 3H), 2.01-1.89 (m, 2H), 1.22-1.10 (m, 4H), 1.08 (t, J = 7.5 Hz, 3H), 0.76 (t, J = 7.2 Hz, 3H). ^{13}C NMR (75 MHz, $CDCl_3$) δ 204.1, 200.1, 163.6, 141.1, 139.6, 128.3, 127.8, 125.3, 115.3, 100.6, 78.4, 30.9, 28.4, 22.2, 17.1, 13.9, 13.7, 12.9. HRMS (ESI) m/z calculated for $C_{20}H_{24}NaO_2$ $[M+Na]^+$: 319.1669, found: 319.1667.

4-(2-(3-chloropropyl)-1-enylidene)-2-ethyl-5-hydroxy-3-methyl-5-phenylcyclopent-2-en-1-one ((-)-16)

The compound **16** was prepared via method A, silica gel column chromatography (EtOAc/PE = 1:5), yellow oil, (44.1 mg, 70% yield, dr > 19:1).

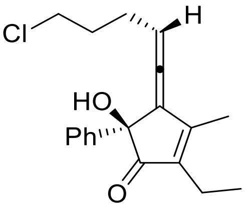

The compound (-)-**16** was prepared via modified method B (step 1 was same with standard procedure; step 2 for 96 h, and then was added MgO (200 mg) stirred for another 6 h), silica gel column chromatography (EtOAc/PE = 1:5), yellow oil (36.3 mg, 57% yield, 89% ee). The enantiomeric excess was determined by HPLC analysis on a Daicel Chiralpak IC column (hexane/iPrOH = 50:50, flow rate: 1.0 mL/min, λ= 254 nm, t_R(major) = 6.28 min, t_R (minor) = 10.44 min. $[\alpha]_D^{25}$ = - 10.3 (c = 0.1 in $CHCl_3$). ^{1}H NMR (400 MHz, $CDCl_3$) δ 7.45-7.16 (m, 5H), 5.88 (t, J = 6.4 Hz, 1H), 3.53 (s, 1H, -OH), 3.37-3.14 (m, 2H, $-CH_2-Cl$), 2.47-2.24 (m, 2H), 2.17-2.05 (m, 5H, $-CH_2-$, $-CH_3$), 1.66-1.51 (m, 1H), 1.53-1.39 (m, 1H), 1.09 (t, J = 7.6 Hz, 3H). ^{13}C NMR (100 MHz, $CDCl_3$) δ 204.0, 200.1, 163.2, 141.0, 140.2, 128.4, 127.9, 125.1, 116.0, 99.1, 78.5, 44.1, 31.2, 25.7, 17.1, 13.7, 12.9. HRMS (ESI) calculated for $C_{19}H_{21}ClNaO_2$ $[M+Na]^+$: 339.1122, found: 339.1124.

4-(2-cyclopropyl-1-enylidene)-2-ethyl-5-hydroxy-3-methyl-5-phenylcyclopent-2-en-1-one ((-)-17)

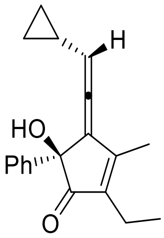

The compound **17** was prepared via method A, silica gel column chromatography (EtOAc/PE = 1:6), (colorless oil, 39.8 mg, 72% yield, dr > 19:1).

The compound **(-)-17** was prepared via modified method B (step 1 same with standard procedure; step 2 for 96 h, and then was added MgO (200 mg) stirred for another 6 h), silica gel column chromatography (EtOAc/PE = 1:6), (colorless oil, 28.3 mg, 50% yield, 86% ee), the enantiomeric excess was determined by HPLC analysis on a Daicel Chiralpak IC column (hexane/iPrOH = 50:50, flow rate: 1.0 mL/min, λ= 254 nm, t_R (major) = 7.56 min, t_R (minor) = 12.46 min. $[\alpha]_D^{25}$ = - 13.1 (c = 0.1 in $CHCl_3$). ^{1}H NMR (400 MHz, $CDCl_3$) δ 7.45-7.19 (m, 5H), 5.86 (d, J = 7.2 Hz, 1H), 3.03 (s, 1H), 2.52-2.24 (m, 2H), 2.12 (s, 3H), 1.15-1.08 (m, 1H, -CH-), 1.08 (t, J = 7.2 Hz, 3H), 0.67-0.49 (m, 2H), 0.24-0.12 (m, 1H), 0.09-0.01 (m, 1H). ^{13}C NMR (100 MHz, $CDCl_3$) δ 204.1, 199.7, 163.2, 141.3, 140.0, 128.4, 127.9, 125.3, 116.9, 105.2, 78.3, 17.10, 13.7, 12.9, 9.1, 8.0, 7.3. HRMS (ESI) calculated for $C_{19}H_{20}NaO_2$ $[M+Na]^+$: 303.1356, found: 303.1360.

4-(2-tert-butyl-1-enylidene)-2-ethyl-5-hydroxy-3-methyl-5-phenylcyclopent-2-en-1-one ((-)-18)

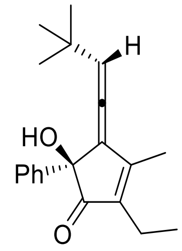

The compound **18** was prepared via method A, silica gel column chromatography (EtOAc/PE = 1:5), colorless solid, (39.0 mg, 66% yield, dr > 19:1).

The compound **(-)-18** was prepared via modified method B (step 1 same with standard procedure; step 2 for 96 h, and then was added MgO (200 mg) stirred for another 6 h), silica gel column chromatography (EtOAc/PE = 1:5), colorless solid (32.6 mg, 61% yield, 86% ee). m.p. 86-88 °C. The enantiomeric excess was determined by HPLC analysis on a Daicel Chiralpak IC column (hexane/iPrOH = 50:50, flow rate: 1.0 mL/min, λ= 254 nm, t_R(major) = 6.48 min, t_R (minor) = 9.41 min. $[\alpha]_D^{25}$ = - 65.5 (c = 0.2 in $CHCl_3$). ^1H NMR (300 MHz, $CDCl_3$) δ 7.46-7.06 (m, 5H), 5.84 (s, 1H), 3.02 (s, 1H), 2.49-2.34 (m, 2H), 2.11 (s, 3H), 1.12 (t, J = 7.6 Hz, 3H), 0.81 (s, 9H). ^{13}C NMR (75 MHz, $CDCl_3$) δ 204.6, 197.8, 163.8, 141.5, 139.9, 128.3, 127.7, 125.1, 116.7, 112.1, 78.3, 33.8, 30.0, 17.1, 13.6, 13.0. HRMS (ESI) m/z calculated for $C_{20}H_{24}NaO_2$ $[M+Na]^+$: 319.1669, found: 319.1672.

4-(2-phenyl-1-enylidene)-3-ethyl-5-hydroxy-5-phenylcyclopent-2-en-1-one ((-)-19).

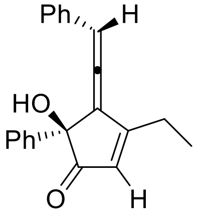

The compound **19** was prepared via method A, silica gel column chromatography (EtOAc/PE = 1:5), colorless oil, (43.4 mg, 73% yield, dr > 19:1).

The compound (-)-**19** was prepared via method B, silica gel column chromatography (EtOAc/PE = 1:5), colorless oil (39.8 mg, 66% yield, 84% ee). The enantiomeric excess was determined by HPLC analysis on a Daicel Chiralpak IC column (hexane/iPrOH = 50:50, flow rate: 1.0 mL/min, λ= 254 nm, t_R(major) = 7.94 min, t_R (minor) = 5.65 min. $[\alpha]_D^{25}$ = - 124.1 (c = 0.2 in $CHCl_3$). ^1H NMR (400 MHz, $CDCl_3$) δ 7.47-7.44 (m, 2H), 7.33-7.24 (m, 3H), 7.18-7.13 (m, 3H), 6.92-6.88 (m, 2H), 6.83 (d, J = 0.8 Hz, 1H), 6.20 (d, J = 1.2 Hz, 1H), 3.24 (s, 1H), 2.66-2.42 (m, 2H), 1.24 (t, J = 7.4 Hz, 3H). ^{13}C NMR (100 MHz, $CDCl_3$) δ 204.4, 203.4, 176.0, 140.9, 132.2, 128.8, 128.6, 128.2, 127.4, 125.8, 125.2, 117.7, 103.2, 80.0, 23.9, 11.9. HRMS (ESI) m/z calculated for $C_{21}H_{18}NaO_2$ $[M+Na]^+$: 325.1199, found: 325.1193.

4-(2-phenyl-1-enylidene)-5-hydroxy-2,3-dimethyl-5-phenylpent-2-en-1-one ((-)-21)

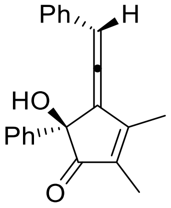

The compound **20** was prepared via method A, silica gel column chromatography (EtOAc/PE = 1:3), white solid (50.3 mg, 83% yield, dr > 19:1).

The compound (-)-**20** was prepared via method B, silica gel column chromatography (EtOAc/PE = 1:3), white solid (46.0 mg, 76% yield, 97% ee). m.p. 191-193 °C. The enantiomeric excess was determined by HPLC analysis on a Daicel Chiralpak IC column (hexane/iPrOH = 70:30, flow rate: 1.0 mL/min, λ= 254 nm, t_R(major) = 8.28 min, t_R (minor) = 10.55 min. $[\alpha]_D^{25}$ = - 330.2 (c = 0.5 in $CHCl_3$). ^1H NMR (300 MHz, $CDCl_3$) δ 7.46-7.37 (m, 2H), 7.33-7.21 (m, 3H), 7.20-7.12 (m, 3H), 6.97-6.87 (m, 2H), 6.84 (s, 1H), 3.25 (s, 1H), 2.13 (s, 3H), 1.97-1.86 (m, 3H). ^{13}C NMR (75 MHz, $CDCl_3$) δ 204.2, 202.7, 162.9, 141.1, 135.5, 132.8, 128.7, 128.5, 128.1, 128.0, 127.3, 125.3, 118.4, 103.4, 79.0, 14.1, 8.9. HRMS (ESI) m/z calculated for $C_{21}H_{18}NaO_2$ $[M+Na]^+$: 325.1199, found: 325.1204.

4-(2-phenyl-1-enylidene)-3-ethyl-5-hydroxy-2-propyl-5-phenylcyclopent-2-en-1-one ((-)-21)

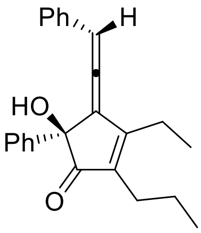

The compound **21** was prepared via method A, silica gel column chromatography (EtOAc/PE = 1:5), yellow oil (49.0 mg, 71% yield, dr > 19:1)

The compound (-)-**21** was prepared via method B, silica gel column chromatography (EtOAc/PE = 1:5), yellow oil (41.3 mg, 60% yield, 92% ee), the enantiomeric excess was determined by HPLC analysis on a Daicel Chiralpak IC column (hexane/iPrOH = 60:40, flow rate: 1.0 mL/min, λ= 254 nm, t_R(major) = 6.82 min, t_R (minor) = 8.96 min. $[\alpha]_D^{25}$ = -316.2 (c = 0.5 in $CHCl_3$). ^1H NMR (300 MHz, $CDCl_3$) δ 7.47-

7.38 (m, 2H), 7.32-7.21 (m, 3H), 7.18-7.11 (m, 3H), 6.95-6.86 (m, 2H), 6.80 (s, 1H), 3.24 (s, 1H), 2.65-2.48 (m, 2H), 2.47-2.28 (m, 2H), 1.64-1.49 (m, 2H), 1.14 (t, J = 7.6 Hz, 3H), 0.97 (t, J = 7.4 Hz, 3H). ^{13}C NMR (75 MHz, $CDCl_3$) δ 204.5, 202.7, 168.3, 141.4, 139.0, 132.8, 128.7, 128.5, 128.0, 127.9, 127.3, 125.1, 116.9, 103.0, 79.0, 25.9, 22.0, 21.9, 14.3, 13.3. HRMS (ESI) calculated for $C_{24}H_{24}NaO_2$ $[M+Na]^+$: 367.1669, found: 367.1674.

4-(2-phenyl-1-enylidene)-3-benzyl-2-(2-phenylethyl)-5-hydroxy-5-phenyl-pent-2-en-1-one ((-)-22)

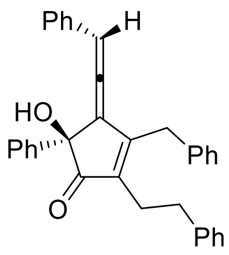

The compound **22** was prepared via method A, silica gel column chromatography (EtOAc/PE = 1:5), pale yellow solid (57.6 mg, 61% yield, dr > 19:1).

The compound (-)-**22** was prepared via method B, silica gel column chromatography (EtOAc/PE = 1:5), pale yellow solid (39.2 mg, 42% yield, 91% ee). m.p. 100-103 °C. The enantiomeric excess was determined by HPLC analysis on a Daicel Chiralpak IC column (hexane/iPrOH = 70:30, flow rate: 1.0 mL/min, λ= 254 nm, t_R(major) = 8.20 min, t_R (minor) = 9.64 min. $[\alpha]_D^{25}$ = - 122.3 (c = 0.5 in $CHCl_3$). ^{1}H NMR (400 MHz, $CDCl_3$) δ 7.35-7.19 (m, 8H), 7.16-7.03 (m, 8H), 6.95-6.87 (m, 2H), 6.73 (s, 1H), 6.67-6.61 (m, 2H), 3.73 (d, J = 14.4 Hz, 1H), 3.58 (d, J = 14.4 Hz, 1H), 3.16 (s, 1H), 2.91-2.64 (m, 4H). ^{13}C NMR (75 MHz, $CDCl_3$) δ 204.3, 203.5, 165.1, 141.1, 140.8, 138.6, 137.0, 132.3, 128.79, 128.75, 128.62, 128.52, 128.48, 128.1, 127.9, 127.5, 126.7, 126.4, 125.5, 117.2, 103.5, 79.2, 34.1, 33.9, 26.6. HRMS (ESI) calculated for $C_{34}H_{29}O_2$ $[M+H]^+$: 469.2168, found: 469.2166.

4-(2-phenyl-1-enylidene)-5-hydroxy-3-pentyl-2,5-diphenylcyclopent-2-en-1-one ((-)23)

The compound **23** was prepared via method A, silica gel column chromatography (EtOAc/PE = 1:5), yellow oil (54.0 mg, 61% yield, dr > 19:1).

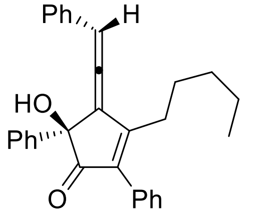

The compound (-)-**23** was prepared via method B, silica gel column chromatography (EtOAc/PE = 1:5), yellow oil (39.5 mg, 47% yield, 81% ee). The enantiomeric excess was determined by HPLC analysis on a Daicel Chiralpak IC column (hexane/iPrOH = 50:50, flow rate: 1.0 mL/min, λ= 254 nm, t_R(major) = 6.22 min, t_R (minor) = 9.38 min. $[\alpha]_D^{25}$ = - 304.5 (c = 0.1 in $CHCl_3$). ^{1}H NMR (300 MHz, $CDCl_3$) δ 7.52-7.44 (m, 7H), 7.30-7.27 (m, 3H), 7.24-7.14 (m, 3H), 6.94-6.87 (m, 3H), 3.52 (s, 1H), 2.69-2.60 (m, 2H), 1.57-1.51 (m, 2H), 1.17-1.09 (m, 4H), 0.70 (t, J = 7.1 Hz, 3H). ^{13}C NMR (75 MHz, $CDCl_3$) δ 204.1, 203.0, 167.9, 141.5, 137.8, 132.4, 131.1, 129.2, 128.8, 128.68, 128.67, 128.60, 128.2, 128.1, 127.4, 125.2, 117.3, 103.3, 79.2, 31.9, 29.0, 28.5, 22.2, 13.8. HRMS (ESI) m/z calculated for $C_{30}H_{29}NaO_2$ $[M+Na]^+$: 443.1982, found: 443.1997.

4-(2-phenyl-1-enylidene)-2-ethyl-5-hydroxy-3-methyl-5-(4-methylphenyl)-cyclopent-2-en-1-one ((-)-24)

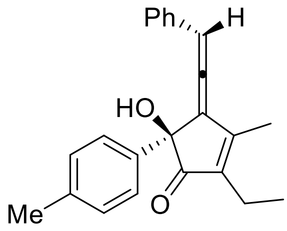

The compound **24** was prepared via method A, silica gel column chromatography (EtOAc/PE = 1:5), white solid (50.6 mg, 76% yield, dr > 19:1).

The compound **(-)-24** was prepared via method B, silica gel column chromatography (EtOAc/PE = 1:6-1:3), pale yellow solid (42.8 mg, 65% yield, 90% ee), m.p. 126-128 °C. The enantiomeric excess was determined by HPLC analysis on a Daicel Chiralpak IC column (hexane/iPrOH = 70:30, flow rate: 1.0 mL/min, λ= 254 nm, t_R(major) = 8.64 min, t_R (minor) = 11.22 min. $[\alpha]_D^{25}$ = - 102.7 (c = 0.1 in CHCl3). ^{1}H NMR (400 MHz, $CDCl_3$) δ 7.31 (d, J = 8.0 Hz, 2H), 7.19-7.16 (m, 3H), 7.09 (d, J = 8.0 Hz, 2H), 6.99-6.94 (m, 2H), 6.83 (s, 1H), 3.18 (s, 1H), 2.52-2.36 (m, 2H), 2.30 (s, 3H), 2.13 (s, 3H), 1.12 (t, J = 7.6 Hz, 3H). ^{13}C NMR (100 MHz, $CDCl_3$) δ 204.0, 202.8, 162.1, 140.9, 138.2, 137.8, 132.9, 129.2, 128.7, 127.9, 127.3, 125.2, 118.5, 103.3, 78.9, 21.2, 17.2, 13.8, 12.9. HRMS (ESI) m/z calculated for $C_{23}H_{22}NaO_2$ $[M+Na]^+$: 353.1512, found: 353.1510.

4-(2-phenyl-1-enylidene)-2-ethyl-5-hydroxy-3-methyl-5-(4-methoxyphenyl)-cyclopent-2-en-1-one ((-)-25)

The compound **25** was prepared via method A, silica gel column chromatography (toluene/EtOAc/PE = 5:2:3), yellow

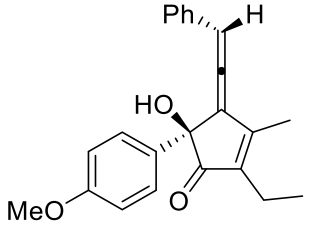

oil (54.3 mg, 78% yield, dr > 19:1).

The compound (-)-**25** was prepared via method B, silica gel column chromatography (toluene/EtOAc/PE = 5:2:3), yellow oil (45.8 mg, 62% yield, 86% ee). The enantiomeric excess was determined by HPLC analysis on a Daicel Chiralpak IC column (hexane/iPrOH = 70:30, flow rate: 1.0 mL/min, λ= 254 nm, t_R(major) = 15.79 min, t_R (minor) = 17.81 min. $[\alpha]_D^{25}$ = - 88.0 (c = 0.2 in $CHCl_3$). ^{1}H NMR (300 MHz, $CDCl_3$) δ 7.39-7.33 (m, 2H), 7.23-7.12 (m, 3H), 7.03-6.95 (m, 2H), 6.85 (s, 1H), 6.84-6.77 (m, 2H), 3.76 (s, 3H), 3.10 (s, 1H), 2.52-2.30 (m, 2H), 2.13 (s, 3H), 1.12 (t, J = 7.6 Hz, 3H). ^{13}C NMR (75 MHz, $CDCl_3$) δ 204.0, 202.7, 162.0, 159.5, 140.9, 133.3, 132.9, 128.8, 128.0, 127.3, 126.7, 118.4, 113.9, 103.3, 78.7, 55.4, 17.2, 13.8, 12.9. HRMS (ESI) calculated for $C_{23}H_{22}NaO_3$ $[M+Na]^+$: 369.1461, found: 369.1456.

4-(2-phenyl-1-enylidene)-2-ethyl-5-hydroxy-3-methyl-5-(4-biphenyl)-cyclopent-2-en-1-one ((-)-26)

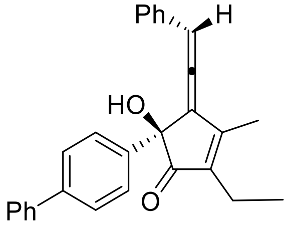

The compound **26** was prepared via method A, silica gel column chromatography (EtOAc/PE = 1:4), white solid (58.6 mg, 75% yield, dr > 19:1).

The compound (-)-**26** was prepared via method B, silica gel column chromatography (EtOAc/PE = 1:4), white solid (50.2 mg, 64% yield, 89% ee). m.p. 156-158 °C. The enantiomeric excess was determined by HPLC analysis on a Daicel Chiralpak IC column (hexane/iPrOH = 70:30, flow rate: 1.0 mL/min, λ= 254 nm, t_R(major) = 13.03 min, t_R (minor) = 9.37 min. $[\alpha]_D^{25}$ = - 24.0 (c = 0.1 in $CHCl_3$). ^{1}H NMR (300 MHz, $CDCl_3$) δ 7.55-7.47 (m, 6H), 7.45-7.38 (m, 2H), 7.36-7.30 (m, 1H), 7.17-7.11 (m, 3H), 6.97-6.90 (m, 2H), 6.86 (s, 1H), 3.23 (s, 1H), 2.59-2.35 (m, 2H), 2.16 (s, 3H), 1.15 (t, J = 7.6 Hz, 3H). ^{13}C NMR (75 MHz, $CDCl_3$) δ 203.9, 202.9, 162.4, 141.1, 141.0, 140.8, 140.3, 132.8, 128.9, 128.7, 128.0, 127.5, 127.35, 127.33, 127.2, 125.7, 118.4, 103.4, 78.9, 17.2, 13.9, 12.9. HRMS (ESI) m/z calculated for $C_{28}H_{24}NaO_2$ $[M+Na]^+$: 415.1669, found: 415.1657.

4-(2-phenyl-1-enylidene)-2-ethyl-5-hydroxy-3-methyl-5-(4-tert- butoxycarbonylaminophenyl)-cyclopent-2-en-1-one (27)

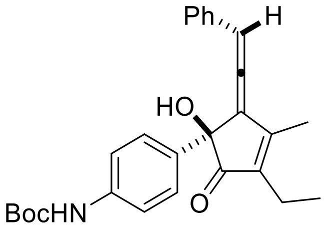

The compound **27** was prepared via method A, silica gel column chromatography (CH_2Cl_2), colorless oil (32.7 mg, 38% yield). ^{1}H NMR (300 MHz, $CDCl_3$) δ 7.39-7.31 (m, 2H), 7.27-7.16 (m, 5H), 7.02-6.97 (m, 2H), 6.84 (s, 1H), 6.56 (s, 1H, -NH), 2.51-2.34 (m, 2H), 2.13 (s, 3H), 1.50 (s, 9H), 1.10 (t, J = 7.6 Hz, 3H). ^{13}C NMR (75 MHz, $CDCl_3$) δ 203.9, 202.6, 162.2, 152.7, 140.9, 138.3, 135.5, 132.8, 128.8, 128.0, 127.3, 126.2, 118.5, 118.3, 103.3, 80.7, 78.8, 28.45, 17.19, 13.8, 12.9. HRMS (ESI) calculated for $C_{27}H_{29}NNaO_4$ $[M+Na]^+$: 454.1989, found: 454.1999.

4-(2-phenyl-1-enylidene)-2-ethyl-5-hydroxy-3-methyl-5-(4-fluorophenyl)-cyclopent-2-en-1-one ((-)-28)

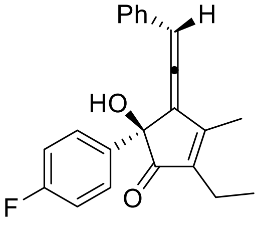

The compound **28** was prepared via method A, silica gel column chromatography (EtOAc/PE = 1:5), pale yellow solid (42.1 mg, 63% yield, dr > 19:1).

The compound (-)-**28** was prepared via method B, silica gel column chromatography (EtOAc/PE = 1:5), pale yellow solid (33.4 mg, 50% yield, 89% ee), m.p. 133-136 °C. The enantiomeric excess was determined by HPLC analysis on a Daicel Chiralpak OD-H column (hexane/iPrOH = 90:10, flow rate: 1.0 mL/min, λ= 254 nm, t_R(major) = 5.59 min, t_R (minor) = 7.39 min. $[\alpha]_D^{25}$ = - 249.0 (c = 0.1 in $CHCl_3$). ^{1}H NMR (300 MHz, $CDCl_3$) δ 7.45-7.34 (m, 2H), 7.24-7.16 (m, 3H), 7.02-6.91 (m, 4H), 6.85 (s, 1H), 3.15 (s, 1H), 2.55-2.32 (m, 2H), 2.15 (s, 3H), 1.13 (t, J = 7.6 Hz, 3H). ^{19}F NMR (282 MHz, $CDCl_3$) δ -114.40. ^{13}C

NMR (75 MHz, $CDCl_3$) δ 203.8, 202.8, 162.60 (d, J = 246.6 Hz), 162.50, 141.07, 137.11 (d, J = 3.0 Hz), 132.63, 128.85, 128.16, 127.22, 127.19 (d, J = 8.2 Hz), 118.37, 115.40 (d, J = 21.6 Hz), 103.5, 78.43, 17.2, 13.9, 12.9. HRMS (ESI) m/z calculated for $C_{22}H_{19}FNaO_2$ $[M+Na]^+$: 357.1261, found: 357.1261.

4-(2-phenyl-1-enylidene)-2-ethyl-5-hydroxy-3-methyl-5-(4-bromophenyl)-cyclopent-2-en-1-one ((-)-29)

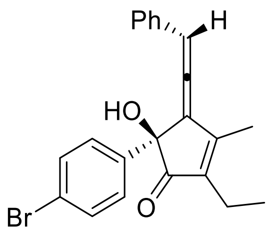

The compound **29** was prepared via method A, silica gel column chromatography (EtOAc/PE = 1:4), pale yellow solid (52.1 mg, 67% yield, dr > 19:1).

The compound (-)-**29** was prepared via method B, silica gel column chromatography (EtOAc/PE = 1:4), pale yellow solid (37.9 mg, 48% yield, 90% ee), m.p. 138-140 °C. The enantiomeric excess was determined by HPLC analysis on a Daicel Chiralpak IC column (hexane/iPrOH = 70:30, flow rate: 1.0 mL/min, λ= 254 nm, t_R(major) = 6.59 min, t_R (minor) = 6.06 min. $[\alpha]_D^{25}$ = - 298.1 (c = 0.1 in $CHCl_3$). ^1H NMR (300 MHz, $CDCl_3$) δ 7.44-7.37 (m, 2H), 7.33-7.25 (m, 2H), 7.21-7.18 (m, 3H), 6.97-6.90 (m, 2H), 6.85 (s, 1H), 3.26 (s, 1H, -OH), 2.53-2.31 (m, 2H), 2.15 (s, 3H), 1.12 (t, J = 7.6 Hz, 3H). ^{13}C NMR (75 MHz, $CDCl_3$) δ 203.4, 202.8, 162.6, 141.1, 140.4, 132.5, 131.6, 128.9, 128.2, 127.2, 127.1, 122.1, 118.1, 103.6, 78.5, 17.2, 13.9, 12.9. HRMS (ESI) m/z calculated for $C_{22}H_{19}BrNaO_2$ $[M+Na]^+$: 417.0461, 419.0440 found: 417.0454, 419.0442.

4-(2-phenyl-1-enylidene)-2-ethyl-5-hydroxy-3-methyl-5-(4-methoxycarbonylphenyl)-cyclopent-2-en-1-one ((-)-30)

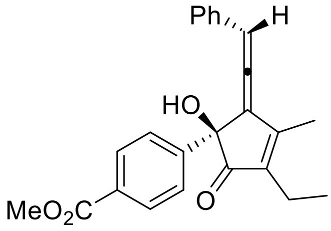

The compound **30** was prepared via method A, silica gel column chromatography (EtOAc/PE = 1:5), pale yellow solid (41.2 mg, 55% yield, dr > 19:1).

The compound (-)-**30** was prepared via method B, silica gel column chromatography (EtOAc/PE = 1:5), pale yellow solid (32.1 mg, 43% yield, 91% ee). m.p. 125-126 °C. The enantiomeric excess was determined by HPLC analysis on a Daicel Chiralpak IC column (hexane/iPrOH = 60:40, flow rate: 1.0 mL/min, λ= 254 nm, t_R(major) = 9.16 min, t_R (minor) = 12.34 min. $[\alpha]_D^{25}$ = - 134.0 (c = 0.1 in $CHCl_3$). ^1H NMR (400 MHz,) δ 7.96 (d, J = 8.3 Hz, 2H), 7.50 (d, J = 8.3 Hz, 2H), 7.21-7.11 (m, 3H), 6.90 (dd, J = 6.3, 2.8 Hz, 2H), 6.85 (s, 1H), 3.89 (s, 3H, -OMe), 3.17 (s, 1H, -OH), 2.50-2.32 (m, 2H), 2.17 (s, 3H), 1.14 (t, J = 7.6 Hz, 3H). ^{13}C NMR (75 MHz, $CDCl_3$) δ 203.3, 202.9, 166.9, 162.9, 146.2, 141.2, 132.4, 129.9, 128.9, 128.2, 127.2, 125.3, 118.1, 103.6, 78.9, 52.3, 17.3, 13.9, 12.9. HRMS (ESI) m/z calculated for $C_{24}H_{22}NaO_4$ $[M+H]^+$: 397.1410, found: 397.1401.

4-(2-phenyl-1-enylidene)-2-ethyl-5-hydroxy-3-methyl-5-(4-trifluoromethylphenyl)-pent-2-en-1-one ((-)-31)

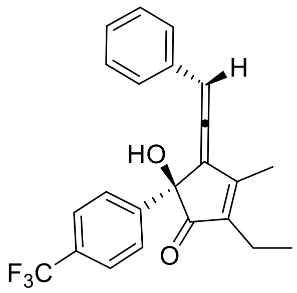

The compound **31** was prepared via method A, silica gel column chromatography (EtOAc/PE = 1:5), yellow solid (44.5 mg, 58% yield, dr > 19:1).

The compound (-)-**31** was prepared via method B, silica gel column chromatography (EtOAc/PE = 1:5), yellow solid (33.8 mg, 44% yield, 89% ee), m.p. 114-115 °C. The enantiomeric excess was determined by HPLC analysis on a Daicel Chiralpak IC column (hexane/iPrOH = 60:40, flow rate: 1.0 mL/min, λ= 254 nm, t_R(major) = 5.59 min, t_R (minor) = 7.39 min. $[\alpha]_D^{25}$ = - 96.0 (c = 0.1 in $CHCl_3$). ^1H NMR (300 MHz, $CDCl_3$) δ 7.65-7.46 (m, 4H), 7.24-7.09 (m, 3H), 6.93-6.76 (m, 3H), 3.20 (s, 1H), 2.54-2.34 (m, 2H), 2.17 (s, 3H), 1.15 (t, J = 7.6 Hz, 3H). ^{19}F NMR (282 MHz, $CDCl_3$) δ -62.61. ^{13}C NMR (75 MHz, $CDCl_3$) δ 203.3, 203.1, 163.0, 145.4, 141.3, 132.3, 128.8, 128.3, 127.2, 125.9, 125.60, 125.55, 125.50, 118.1, 103.7, 78.6, 17.2, 14.0, 12.9. HRMS (ESI) m/z calculated for $C_{23}H_{20}F_3NaO_2$ $[M+Na]^+$: 407.1229, found: 407.1244.

4-(2-phenyl-1-enylidene)-2-ethyl-5-hydroxy-3-methyl-5-(3-methylphenyl)-cyclopent-2-en-1-one ((-)-32)

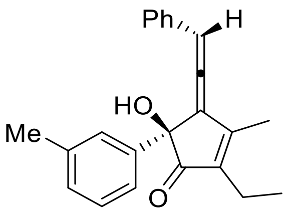

The compound **32** was prepared via method A, silica gel column chromatography (EtOAc/PE = 1:5), pale yellow solid (52.2 mg, 74% yield, dr > 19:1).

The compound (-)-**32** was prepared via method B, silica gel column chromatography (EtOAc/PE = 1:5), pale yellow solid (43.6 mg, 66% yield, 90% ee), m.p. 154-157 °C, The enantiomeric excess was determined by HPLC analysis on a Daicel Chiralpak IC column (hexane/iPrOH = 60:40, flow rate: 1.0 mL/min, λ= 254 nm, t_R(major) = 6.28 min, t_R (minor) = 8.36 min. $[\alpha]_D^{25}$ = - 180.0 (c = 0.2 in CHCl3). ^{1}H NMR (400 MHz, $CDCl_3$) δ 7.28 (s, 1H), 7.25-7.16 (m, 5H), 7.09 (d, *J* = 7.2 Hz, 1H), 7.01-6.96 (m, 2H), 6.87 (s, 1H), 3.12 (s, 1H), 2.53-2.35 (m, 2H), 2.27 (s, 3H), 2.17 (s, 3H), 1.15 (t, *J* = 7.6 Hz, 3H). ^{13}C NMR (100 MHz, $CDCl_3$) δ 203.9, 202.9, 162.2, 141.0, 138.2, 132.9, 128.8, 128.7, 128.4, 127.9, 127.3, 126.1, 122.4, 118.4, 103.3, 78.9, 21.6, 17.2, 13.9, 12.9. HRMS (ESI) m/z calculated for $C_{23}H_{22}NaO_2$ $[M+Na]^+$: 353.1512, found: 353.1516.

4-(2-phenyl-1-enylidene)-2-ethyl-5-hydroxy-3-methyl-5-(3-methoxyphenyl)-cyclopent-2-en-1-one ((-)-33)

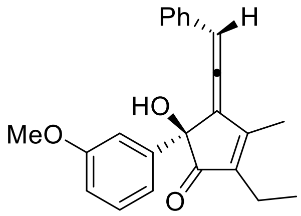

The compound **33** was prepared via method A, silica gel column chromatography (EtOAc/PE = 1:5), pale yellow solid (47.1 mg, 68% yield, dr > 19:1).

The compound (-)-**33** was prepared via method B, silica gel column chromatography (EtOAc/PE = 1:5), pale yellow solid (44.2 mg, 64% yield, 90% ee). m.p. 109-111 °C. The enantiomeric excess was determined by HPLC analysis on a Daicel Chiralpak OD-H column (hexane/iPrOH = 95:05, flow rate: 1.0 mL/min, λ= 254 nm, t_R(major) = 17.41 min, t_R (minor) = 14.51 min. $[\alpha]_D^{25}$ = - 88.0 (c = 0.1 in $CHCl_3$). ^{1}H NMR (400 MHz, $CDCl_3$) δ 7.23-7.14 (m, 4H), 7.06-7.01 (m, 1H), 6.97-6.89 (m, 3H), 6.84 (s, 1H), 6.81-6.76 (m, 1H), 3.66 (s, 3H), 3.23 (s, 1H), 2.54-2.36 (m, 2H), 2.14 (s, 3H), 1.13 (t, *J* = 7.6 Hz, 3H). ^{13}C NMR (100 MHz, $CDCl_3$) δ 203.8, 202.9, 162.3, 159.8, 142.9, 141.1, 132.8, 129.5, 128.8, 128.0, 127.3, 118.4, 117.5, 114.0, 110.8, 103.4, 78.8, 55.2, 17.2, 13.9, 12.9. HRMS (ESI) calculated for $C_{23}H_{22}NaO_3$ $[M+Na]^+$: 369.1461, found: 369.1450.

4-(2-phenyl-1-enylidene)-2-ethyl-5-hydroxy-3-methyl-5-(3-chlorophenyl)-cyclopent-2-en-1-one ((-)-34)

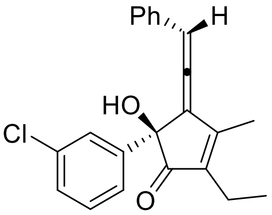

The compound **35** was prepared via method A, silica gel column chromatography (EtOAc/PE = 1:5), pale yellow solid (52.0 mg, 74% yield, dr > 19:1).

The compound (-)-**35** was prepared via method B, silica gel column chromatography (EtOAc/PE = 1:5), pale yellow solid (44.0 mg, 63% yield, 92% ee). m.p. 158-160 °C. The enantiomeric excess was determined by HPLC analysis on a Daicel Chiralpak IC column (hexane/iPrOH = 70:30, flow rate: 1.0 mL/min, λ= 254 nm, t_R(major) = 6.67 min, t_R (minor) = 8.24 min. $[\alpha]_D^{25}$ = - 127.0 (c = 0.1 in $CHCl_3$). ^{1}H NMR (400 MHz, $CDCl_3$) δ 7.41 (s, 1H), 7.34-7.31 (m, 1H), 7.25-7.17 (m, 5H), 6.97-6.95 (m, 2H), 6.85 (s, 1H), 3.22 (s, 1H), 2.51-2.37 (m, 2H), 2.17 (s, 3H), 1.13 (t, *J* = 7.6 Hz, 3H). ^{13}C NMR (100 MHz, $CDCl_3$) δ 203.2, 202.9, 162.8, 143.2, 141.1, 134.5, 132.5, 129.8, 128.9, 128.2, 127.3, 125.7, 123.5, 118.2, 103.6, 78.5, 17.2, 13.9, 12.9. HRMS (ESI) m/z calculated for $C_{22}H_{19}ClNaO_2$ $[M+Na]^+$: 373.0966, found: 373.0962.

4-(2-phenyl-1-enylidene)-2-ethyl-5-hydroxy-3-methyl-5-(-2-methylphenyl)-cyclopent-2-en-1-one (35)

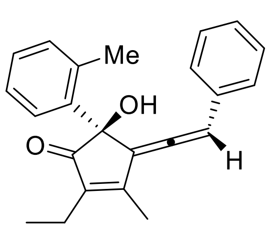

The compound **35** was prepared via method A, silica gel column chromatography (EtOAc/PE = 1:6), pale yellow solid (22.5 mg, 34% yield, dr > 19:1).

The compound (-)-**35** was prepared via method B, silica gel column chromatography (EtOAc/PE = 1:6), pale yellow solid (18.5 mg, 28% yield, 80% ee), m.p. 153-155 °C. The enantiomeric excess was determined by HPLC analysis on a Daicel Chiralpak IC column (hexane/iPrOH = 85:15, flow rate: 1.0 mL/min, λ= 254 nm, t_R(major) = 8.05 min, t_R (minor) = 14.60 min. $[\alpha]_D^{25}$ = - 235

(c = 0.2 in CHCl3). ^{1}H NMR (400 MHz, $CDCl_3$) δ 7.67-7.55 (m, 1H), 7.19-7.08 (m, 6H), 6.79-6.76 (m, 2H), 6.76 (s, 1H), 2.97 (s, 1H), 2.56-2.41 (m, 2H), 2.25 (s, 3H), 2.14 (s, 3H), 1.16 (t, J = 7.6 Hz, 3H). ^{13}C NMR (100 MHz, $CDCl_3$) δ 204.8, 203.1, 161.5, 140.2, 138.8, 134.9, 134.2, 132.9, 131.6, 128.7, 128.1, 127.9, 127.2, 126.4, 126.1, 102.9, 79.1, 21.3, 17.2, 13.7, 12.7. HRMS (ESI) m/z calculated for $C_{23}H_{22}NaO_2$ $[M+Na]^+$: 353.1512, found: 353.1508.

4-(2-phenyl-1-enylidene)-2-ethyl-5-hydroxy-3-methyl-5-(2,4-dichlorophenyl)-cyclopent-2-en-1-one ((-)-36)

The compound **36** was prepared via method A, silica gel column chromatography (EtOAc/PE = 1:5), pale yellow solid (40.4 mg, 53% yield, dr > 19:1).

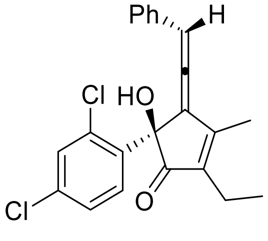

The compound (-)-**36** was prepared via method B, silica gel column chromatography (EtOAc/PE = 1:5), pale yellow solid (36.0 mg, 46% yield, 82% ee). m.p. 166-168 °C. The enantiomeric excess was determined by HPLC analysis on a Daicel Chiralpak IC column (hexane/iPrOH = 95:05, flow rate: 1.0 mL/min, λ= 254 nm, t_R(major) = 10.28 min, t_R (minor) = 11.46 min. $[\alpha]_D^{25}$ = - 126.0 (c = 0.1 in $CHCl_3$). ^{1}H NMR (300 MHz, $CDCl_3$) δ 7.83 (d, J = 8.4 Hz, 1H), 7.30 (d, J = 2.1 Hz, 1H), 7.24-7.14 (m, 4H), 7.00-6.93 (m, 2H), 6.72 (s, 1H), 3.21 (br, 1H, -OH), 2.63-2.33 (m, 2H), 2.14 (s, 3H), 1.13 (t, J = 7.6 Hz, 3H). ^{13}C NMR (75 MHz, $CDCl_3$) δ 202.74, 202.67, 160.9, 140.3, 137.1, 134.5, 132.4, 131.3, 129.9, 129.6, 128.8, 128.1, 127.3, 127.2, 116.6, 103.2, 77.8, 17.3, 13.7, 12.5. HRMS (ESI) m/z calculated for $C_{22}H_{19}Cl_2O_2$ $[M+H]^+$: 385.0757, found: 385.0751.

4-(2-phenyl-1-enylidene)-2-ethyl-5-hydroxy-3-methyl-5-(3,4-dichlorophenyl)-cyclopent-2-en-1-one ((-)-37)

The compound **37** was prepared via method A, silica gel column chromatography (EtOAc/PE = 1:4), pale yellow solid (52.9 mg, 65% yield, dr > 19:1).

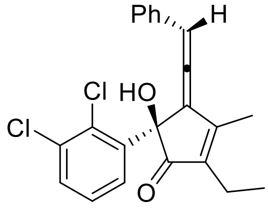

The compound (-)-**37** was prepared via method B, silica gel column chromatography (EtOAc/PE = 1:4), pale yellow solid (38.4 mg, 50% yield, 86% ee). m.p. 197-199 °C. The enantiomeric excess was determined by HPLC analysis on a Daicel Chiralpak IC column (hexane/iPrOH = 70:30, flow rate: 1.0 mL/min, λ= 254 nm, t_R(major) = 4.74 min, t_R (minor) = 5.26 min. $[\alpha]_D^{17}$ = - 82.2 (c = 0.1 in CHCl3). ^{1}H NMR (400 MHz, $CDCl_3$) δ 7.82 (dd, J = 8.0, 1.6 Hz, 1H), 7.33 (dd, J = 8.0, 1.6 Hz, 1H), 7.22-7.12 (m, 4H), 6.91 (dd, J = 8.0, 1.6 Hz, 2H), 6.69 (s, 1H), 3.20 (s, 1H), 2.58 -2.35 (m, 2H), 2.15 (s, 3H), 1.15 (t, J = 7.6 Hz, 3H). ^{13}C NMR (100 MHz, $CDCl_3$) δ 202.8, 202.7, 160.7, 140.9, 140.2, 133.5, 132.4, 130.2, 129.0, 128.8, 128.1, 127.5, 127.1, 116.6, 103.1, 78.4, 17.3, 13.7, 12.4. HRMS (ESI) m/z calculated for $C_{22}H_{18}Cl_2NaO_2$ $[M+Na]^+$: 407.0576, found: 407.0575.

4-(2-phenyl-1-enylidene)-2-ethyl-5-hydroxy-3-methyl-5-(3,4-dimethoxyphenyl)-pent-2-en-1-one ((-)-38)

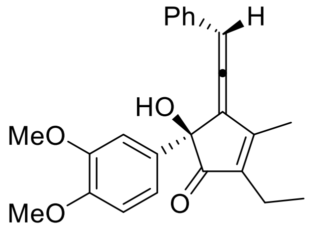

The compound **38** was prepared via method A, silica gel column chromatography (^{t}BuOMe/PE = 1:3), colorless oil (53.6 mg, 67% yield, dr > 19:1).

The compound (-)-**38** was prepared via method B, silica gel column chromatography (^{t}BuOMe/PE = 1:3), colorless oil (45.1 mg, 60% yield, 86% ee), m.p. 165-168 °C, The enantiomeric excess was determined by HPLC analysis on a Daicel Chiralpak IC column (hexane/iPrOH = 70:30, flow rate: 1.0 mL/min, λ= 254 nm, t_R(major) = 7.07 min, t_R (minor) = 9.41 min. $[\alpha]_D^{17}$ = - 222.1 (c = 0.1 in $CHCl_3$). ^{1}H NMR (300 MHz, $CDCl_3$) δ 7.23-7.15 (m, 3H), 7.08 (d, J = 2.1 Hz, 1H), 7.06-7.00 (m, 2H), 6.89 (s, 1H), 6.85 (dd, J = 8.4, 2.1 Hz, 1H), 6.75 (d, J = 8.4 Hz, 1H), 3.84 (s, 3H), 3.60 (s, 3H), 3.17 (s, 1H), 2.58-2.28 (m, 2H), 2.14 (s, 3H), 1.13 (t, J = 7.6 Hz, 3H). ^{13}C NMR (75 MHz, $CDCl_3$) δ 204.0, 202.6, 161.7, 149.3, 149.1, 141.1, 133.8, 132.7, 128.9, 128.1, 127.3, 118.3, 117.7, 110.6, 109.3, 103.3, 78.6, 56.1, 55.7, 17.2, 13.8, 12.9. HRMS (ESI) calculated for $C_{24}H_{24}NaO_4$ $[M+Na]^+$: 399.1567, found: 399.1565.

4-(2-phenyl-1-enylidene)-2-ethyl-5-hydroxy-3-methyl-5-(3,4,5-trimethoxyphenyl)-cyclopent-2-en-1-one ((-)-39)

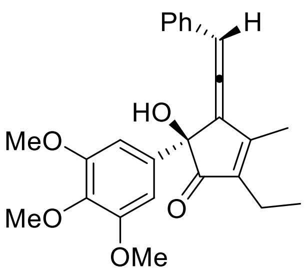

The compound **39** was prepared via method A, silica gel column chromatography (^{t}BuOMe/PE = 1:3), colorless oil (51.2 mg, 63% yield, dr > 19:1).

The compound (-)-**39** was prepared via method B, silica gel column chromatography (^{t}BuOMe/PE = 1:3), colorless oil (45.5 mg, 56% yield, 87% ee), m.p. 165-168 °C, The enantiomeric excess was determined by HPLC analysis on a Daicel Chiralpak IC column (hexane/iPrOH = 60:40, flow rate: 1.0 mL/min, λ= 254 nm, t_R(major) = 19.98 min, t_R (minor) = 15.74 min. $[\alpha]_D^{18}$ = - 270.0 (c = 0.1 in $CHCl_3$). ^{1}H NMR (300 MHz, $CDCl_3$) δ 7.22-7.16 (m, 3H), 7.04-6.97 (m, 2H), 6.89 (s, 1H), 6.65 (s, 2H), 3.79 (s, 3H), 3.66 (s, 6H), 3.17 (s, 1H, -OH), 2.55-2.35 (m, 2H), 2.15 (s, 3H), 1.15 (t, *J* = 7.6 Hz, 3H). ^{13}C NMR (75 MHz, $CDCl_3$) δ 203.8, 202.8, 162.1, 153.2, 141.2, 138.0, 136.9, 132.6, 128.9, 128.2, 127.3, 118.2, 103.5, 102.9, 78.6, 60.9, 56.1, 17.2, 13.9, 12.9. HRMS (ESI) calculated for $C_{25}H_{26}NaO_5$ $[M+Na]^+$: 429.1672, found: 429.1676.

4-(2-phenyl-1-enylidene)-2-ethyl-5-hydroxy-3-methyl-5-(2-naphyl)-cyclopent-2-en-1-one ((-)-40)

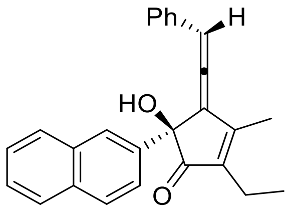

The compound **40** was prepared via method A, silica gel column chromatography (EtOAc/PE = 1:3), colorless solid (39.6 mg, 54% yield, dr > 19:1).

The compound (-)-**40** was prepared via method B, silica gel column chromatography (EtOAc/PE = 1:3), colorless solid (33.7 mg, 46% yield, 80% ee), m.p. 165-168 °C, The enantiomeric excess was determined by HPLC analysis on a Daicel Chiralpak IA column (hexane/iPrOH = 70:30, flow rate: 1.0 mL/min, λ= 254 nm, t_R(major) = 8.05 min, t_R (minor) = 15.05 min. $[\alpha]_D^{18}$ = - 27.0 (c = 0.1 in $CHCl_3$). ^{1}H NMR (300 MHz, $CDCl_3$) δ 7.89 (d, *J* = 1.5 Hz, 1H), 7.80-7.74 (m, 3H), 7.54 (dd, *J* = 8.7, 1.9 Hz, 1H), 7.49-7.43 (m, 2H), 7.16-7.03 (m, 3H), 6.98-6.91 (m, 2H), 6.88 (s, 1H), 3.27 (s, 1H), 2.56-2.37 (m, 1H), 2.18 (s, 3H), 1.16 (t, *J* = 7.6 Hz, 3H). ^{13}C NMR (75 MHz, $CDCl_3$) δ 203.8, 202.9, 162.4, 141.1, 138.3, 133.1, 132.7, 128.7, 128.5, 128.4, 128.0, 127.6, 127.3, 126.4, 126.3, 124.3, 123.6, 118.2, 103.4, 79.2, 17.3, 13.9, 13.0. HRMS (ESI) m/z calculated for $C_{26}H_{22}NaO_2$ $[M+Na]^+$: 389.1512, found: 389.1509.

4-(2-phenyl-1-enylidene)-2-ethyl-5-hydroxy-3-methyl-5-(3-Benzodioxole)-cyclopent-2-en-1-one ((-)-41)

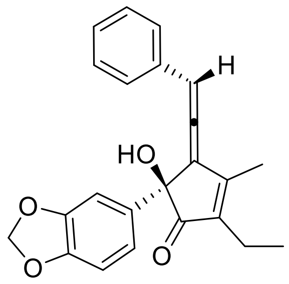

The compound **41** was prepared via method A, silica gel column chromatography (EtOAc/PE = 1:5), colorless oil (49.0 mg, 68% yield, dr > 19:1).

The compound (-)-**41** was prepared via method B, silica gel column chromatography (EtOAc/PE = 1:5), colorless oil (43.9 mg, 61% yield, 86% ee). The enantiomeric excess was determined by HPLC analysis on a Daicel Chiralpak ID column (hexane/iPrOH = 70:30, flow rate: 1.0 mL/min, λ= 254 nm, t_R(major) = 12.87 min, t_R (minor) = 11.30 min. $[\alpha]_D^{25}$ = - 78.0 (c = 0.1 in $CHCl_3$). ^{1}H NMR (400 MHz, $CDCl_3$) δ 7.24-7.18 (m, 3H), 7.00 (dd, *J* = 7.4, 1.9 Hz, 2H), 6.95 (d, *J* = 1.8 Hz, 1H), 6.87 (dd, *J* = 8.1, 1.5 Hz, 1H), 6.84 (s, 1H), 6.71 (d, *J* = 8.1 Hz, 1H), 5.89 (d, *J* = 14.4 Hz, 2H), 3.24 (s, 1H), 2.48-2.28 (m, 2H), 2.14 (s, 3H), 1.12 (t, *J* = 7.6 Hz, 3H). ^{13}C NMR (101 MHz, $CDCl_3$) δ 203.8, 202.8, 162.4, 147.9, 147.4, 140.9, 135.1, 132.8, 128.8, 128.0, 127.3, 118.7, 118.5, 108.2, 106.4, 103.4, 101.2, 78.7, 17.18, 13.9, 12.9. HRMS (ESI) m/z calculated for $C_{23}H_{20}NaO_4$ $[M+Na]^+$: 383.1254, found: 383.1258.

4-(2-phenyl-1-enylidene)-2-ethyl-5-hydroxy-3-methyl-5-(3-Boc-indolyl)-cyclopent-2-en-1-one ((-)-42)

The compound **42** was prepared via method A, silica gel column chromatography (EtOAc/PE = 1:5), yellow oil (54.6 mg, 60% yield, dr > 19:1).

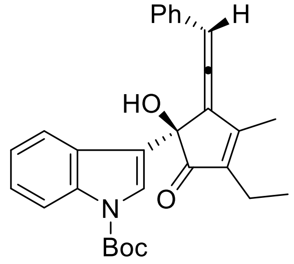

The compound (-)-**42** was prepared via method B, silica gel column chromatography (EtOAc/PE = 1:5), yellow oil (46.3 mg, 51% yield, 84% ee). The enantiomeric excess was determined by HPLC analysis on a Daicel Chiralpak IA column (hexane/iPrOH = 70:30, flow rate: 1.0 mL/min, λ= 254 nm, t_R(major) = 5.65 min, t_R (minor) = 5.20 min. $[\alpha]_D^{18}$ = -76.6 (c = 0.2 in $CHCl_3$). ^{1}H NMR (400 MHz, $CDCl_3$) δ 8.12 (d, J = 7.6 Hz, 1H), 7.60 (s, 1H), 7.50 (d, J = 7.9 Hz, 1H), 7.33-7.18 (m, 1H), 7.1-6.95 (m, 4H), 6.85 (s, 1H), 6.80 (d, J = 7.3 Hz, 2H), 3.14 (s, 1H), 2.55-2.42 (m, 2H), 2.19 (s, 3H), 1.62 (s, 9H), 1.15 (t, J = 7.6 Hz, 3H). ^{13}C NMR (100 MHz, $CDCl_3$) δ 203.1, 202.6, 161.7, 149.5, 140.3, 136.3, 132.5, 128.6, 127.9, 127.5, 127.4, 124.6, 123.8, 122.7, 120.7, 120.6, 116.7, 115.4, 103.3, 83.9, 76.5, 28.3, 17.3, 13.9, 12.9. HRMS (ESI) m/z calculated for $C_{29}H_{29}NNaO_4$ $[M+Na]^+$: 478.1989, found: 478.1991.

4-(2-phenyl-1-enylidene)-2-ethyl-5-hydroxy-3-methyl-5-(3-thiophenyl)-cyclopent-2-en-1-one ((-)-43)

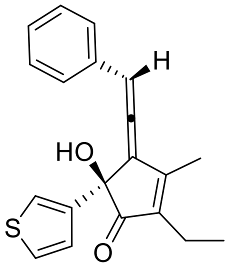

The compound **43** was prepared via method A, silica gel column chromatography (EtOAc/PE = 1:5), brown yellow solid (43.2 mg, 61% yield, dr > 19:1).

The compound (-)-**43** was prepared via method B, silica gel column chromatography (EtOAc/PE = 1:5), brown yellow solid (29.7 mg, 46% yield, 85% ee), m.p. 133-134 ºC, The enantiomeric excess was determined by HPLC analysis on a Daicel Chiralpak IC column (hexane/iPrOH = 70:30, flow rate: 1.0 mL/min, λ= 254 nm, t_R(major) = 9.09 min, t_R (minor) = 10.56 min. $[\alpha]_D^{18}$ = - 87.0 (c = 0.1 in $CHCl_3$). ^{1}H NMR (300 MHz, $CDCl_3$) δ 7.30-7.20 (m, 5H), 7.11-7.03 (m, 3H), 6.89 (s, 1H), 3.17 (s, 1H), 2.53-2.30 (m, 2H), 2.12 (s, 3H), 1.12 (t, J = 7.6 Hz, 3H). ^{13}C NMR (75 MHz, $CDCl_3$) δ 203.1, 202.6, 161.5, 141.7, 140.6, 132.8, 128.9, 128.1, 127.4, 126.6, 125.8, 122.1, 117.5, 103.4, 76.99, 17.2, 13.8, 12.9. HRMS (ESI) m/z calculated for $C_{20}H_{18}O_2NaS$ $[M+Na]^+$: 345.0920, found: 345.0924.

4-(2-phenyl-1-enylidene)-2-ethyl-5-hydroxy-3-methyl-5-(3-(4-chloro)-pyridyl)-pent-2-en-1-one (44)

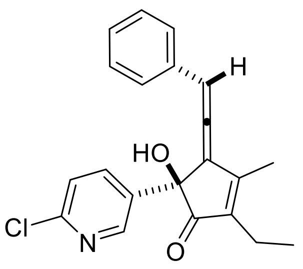

The compound **44** was prepared via method A, silica gel column chromatography (EtOAc/PE = 1:3), yellow oil (25.3 mg, 36% yield). ^{1}H NMR (400 MHz, $CDCl_3$) δ 8.36 (d, J = 2.4 Hz, 1H), 7.82 (dd, J = 8.4, 2.4 Hz, 1H), 7.26-7.20 (m, 4H), 7.06-6.96 (m, 2H), 6.90 (s, 1H), 3.33 (s, 1H), 2.51-2.32 (m, 2H), 2.18 (s, 3H), 1.11 (t, J = 7.6 Hz, 3H). ^{13}C NMR (75 MHz, $CDCl_3$) δ 202.7, 202.4, 162.9, 151.2, 146.7, 141.0, 136.6, 136.0, 132.0, 129.1, 128.5, 127.2, 124.2, 117.6, 104.0, 77.58, 17.2, 13.9, 12.8. HRMS (ESI) calculated for $C_{21}H_{19}ClNNaO_2$ $[M+Na]^+$: 374.0918, found: 374.0920.

Preparation of compound 3

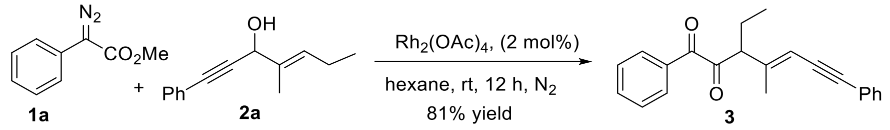

A flame dried tube was cooled to rt., evacuated and backfilled with nitrogen. To the tube was added freshly distilled hexane (2.0 mL), diazo **1a** (0.3 mmol), enyn-3-ol **2a** (0.2 mmol), and then $Rh_2(OAc)_4$ (1.8 mg, 2 mol%) was added to the mixture solution. The tube was sealed and stirred at rt., after complete consumption of the starting material (monitored by TLC), the mixture solution was quenched by H_2O (10 mL) and extracted with EtOAc (3 * 10 mL). The combined organic layer was washed with brine solution and dried over anhydrous Na_2SO_4, concentrated under reduced pressure, the crude product was purified by silica gel column chromatography (EtOAc/PE = 1:20), give compound **3**, as yellow

oil, 37.3 mg, 78% yield, ^{1}H NMR (400 MHz, $CDCl_3$) δ 7.96-7.89 (m, 2H), 7.63 (t, *J* = 7.4 Hz, 1H), 7.49 (t, *J* = 7.8 Hz, 2H), 7.38 (dd, *J* = 6.6, 3.0 Hz, 2H), 7.31-7.26 (m, 3H), 5.68 (s, 1H), 4.05-3.95 (m, 1H), 1.99 (d, *J* = 0.7 Hz, 3H), 2.03-1.90 (m, 1H), 1.81-1.68 (m, 1H), 0.96 (t, *J* = 7.4 Hz, 3H). ^{13}C NMR (75 MHz, $CDCl_3$) δ 200.1, 192.6, 145.5, 134.7, 132.5, 131.5, 130.2, 129.0, 128.4, 128.3, 123.5, 111.4, 94.6, 86.6, 57.0, 21.9, 17.8, 11.9. HRMS (ESI) m/z calculated for $C_{22}H_{20}NaO_2$ $[M+Na]^+$: 339.1356, found: 339.1360.

Preparation of compound 45

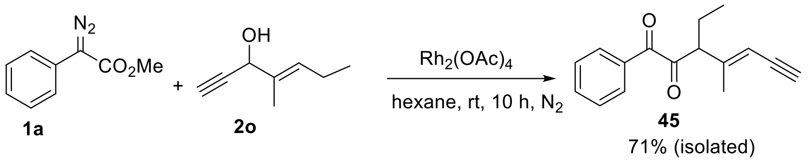

A flame dried tube was cooled to rt., evacuated and backfilled with nitrogen. To the tube was added freshly distilled hexane (2.0 mL), diazo **1a** (0.3 mmol), enyn-3-ol **2o** (0.2 mmol), and then $Rh_2(OAc)_4$ (1.8 mg, 2 mol%) was added to the mixture solution. The tube was sealed and stirred at rt., after complete consumption of the starting material (monitored by TLC), the mixture solution was quenched by H_2O (10 mL) and extracted with EtOAc (3 * 10 mL). The combined organic layer was washed with brine solution and dried over anhydrous Na_2SO_4, concentrated under reduced pressure, the crude product was purified by silica gel column chromatography (EtOAc/PE = 1:20), give compound **46**, as yellow oil, 34.3 mg, 71% yield, ^{1}H NMR (300 MHz, $CDCl_3$) δ 7.94-7.88 (m, 2H), 7.68-7.59 (m, 1H), 7.51-7.43 (m, 2H), 5.45 (s, 1H), 4.01-3.79 (m, 1H), 3.09 (d, *J* = 2.0 Hz, 1H), 2.02-1.83 (m, 1H), 1.93(s, 3H), 1.77-1.64 (m, 1H), 0.93 (t, *J* = 7.4 Hz, 3H). ^{13}C NMR (75 MHz, $CDCl_3$) δ 200.1, 188.4, 147.8, 134.7, 132.4, 130.2, 129.0, 110.3, 82.3, 80.6, 56.9, 21.9, 17.7, 11.9. HRMS (ESI) m/z calculated for $C_{16}H_{16}NaO_2$ $[M+Na]^+$: 263.1043, found: 263.1038.

Preparation of compound 46

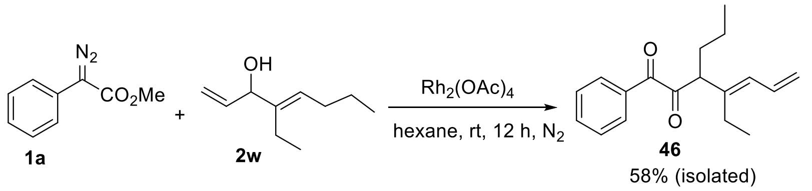

A flame dried tube was cooled to rt., evacuated and backfilled with nitrogen. To the tube was added freshly distilled hexane (2.0 mL), diazo **1a** (0.3 mmol), enyn-3-ol **2w** (0.2 mmol), and then $Rh_2(OAc)_4$ (1.8 mg, 2 mol%) was added to the mixture solution. The tube was sealed and stirred at rt., after complete consumption of the starting material (monitored by TLC), the mixture solution was quenched by H_2O (10 mL) and extracted with EtOAc (3 * 10 mL). The combined organic layer was washed with brine solution and dried over anhydrous Na_2SO_4, concentrated under reduced pressure, the crude product was purified by silica gel column chromatography (EtOAc/PE = 1:20), give compound **46**, as yellow oil, 31.3 mg, 58% yield, ^{1}H NMR (400 MHz, $CDCl_3$) δ 7.90 (d, *J* = 7.7 Hz, 2H), 7.60 (t, *J* = 7.4 Hz, 1H), 7.45 (t, *J* = 7.7 Hz, 2H), 6.47 (dt, *J* = 16.8, 10.5 Hz, 1H), 5.91 (d, *J* = 10.9 Hz, 1H), 5.15 (d, *J* = 16.7 Hz, 1H), 5.06 (d, *J* = 10.1 Hz, 1H), 3.96 (t, *J* = 7.1 Hz, 1H), 2.19 (q, *J* = 7.6 Hz, 2H), 1.96 (dd, *J* = 14.0, 7.4 Hz, 1H), 1.62 (dd, *J* = 14.1, 7.1 Hz, 1H), 1.35 (dt, *J* = 14.8, 7.4 Hz, 2H), 1.00 (t, *J* = 7.6 Hz, 3H), 0.95 (t, *J* = 7.3 Hz, 3H). ^{13}C NMR (75 MHz, $CDCl_3$) δ 200.1, 193.3, 139.3, 134.3, 132.6, 132.1, 130.5, 130.1, 128.7, 117.9, 53.1, 32.2, 24.6, 20.9, 14.1, 13.3. HRMS (ESI) calculated for $C_{18}H_{22}NaO_2$ $[M+Na]^+$: 293.1512, found: 293.1515.

Preparation of compound 47'

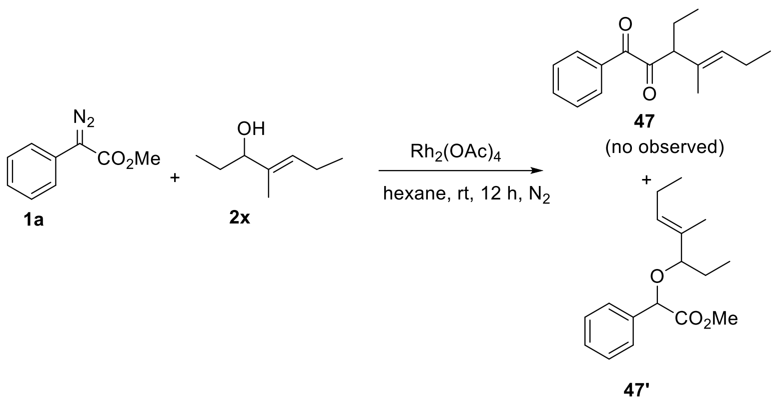

A flame dried tube was cooled to rt., evacuated and backfilled with nitrogen. To the tube was added freshly distilled hexane (2.0 mL), diazo **1a** (0.3 mmol), enyn-3-ol **2x** (0.2 mmol), and then $Rh_2(OAc)_4$ (1.8 mg, 2 mol%) was added to the mixture solution. The tube was sealed and stirred at rt., after complete consumption of the starting material (monitored by TLC), the mixture solution was quenched by H_2O (10 mL) and extracted with EtOAc (3 * 10 mL). The combined organic layer was washed with brine solution and dried over anhydrous Na_2SO_4, concentrated under reduced pressure, the crude product was purified by silica gel column chromatography (EtOAc/PE = 1:20), give compound **47'**, as a oil, 37.5 mg, 68% yield. ^{1}H NMR (300 MHz, $CDCl_3$) δ 7.44-7.39 (m, 2H), 7.36-7.27 (m, 3H), 5.35 (t, J = 6.8 Hz, 1H), 4.86 (s, 1H), 3.71 (s, 3H), 3.70-3.64 (m, 1H), 2.11-1.98 (m, 2H), 1.86-1.71 (m, 1H), 1.63-1.49 (m, 1H), 1.45 (s, 3H), 0.97 (t, J = 7.5 Hz, 3H), 0.90 (t, J = 7.4 Hz, 3H). ^{13}C NMR (75 MHz, $CDCl_3$) δ 172.3, 137.4, 132.6, 132.4, 128.5, 128.3, 127.2, 87.1, 77.3, 52.0, 26.3, 20.9, 14.1, 10.4, 10.3. HRMS (ESI) calculated for $C_{17}H_{25}O_3$ $[M+H]^+$: 277.1798, found: 277.1796.

Preparation of compound 49

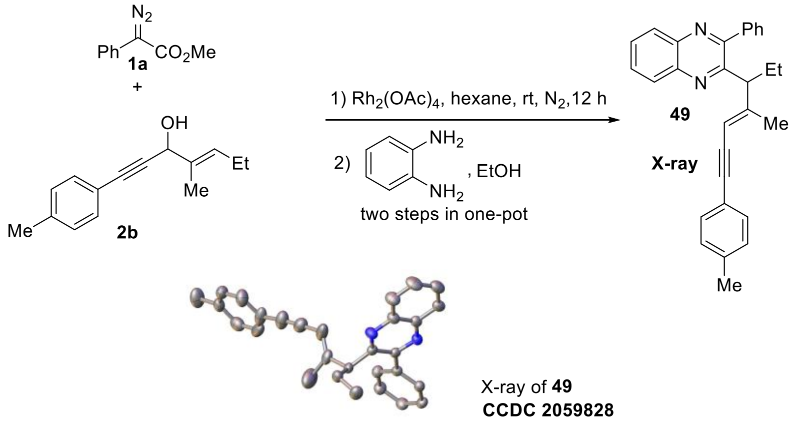

A flame dried tube was cooled to rt., evacuated and backfilled with nitrogen. To the tube was added freshly distilled hexane (2.0 mL), diazo **1** (0.3 mmol), enyn-3-ol **2** (0.2 mmol), and then $Rh_2(OAc)_4$ (1.8 mg, 2 mol%) was added to the mixture solution. The tube was sealed and stirred at rt. until the enyn-3-ol disappear. And then the 1,2-diaminobenzene (0.22 mmol) and EtOH (8 mL) was added to the mixture solution, and stirred for another 1 h. The mixture solution was quenched with water, and extraction with ethyl acetate (3*10 mL). The combine organic phase was concentrated by rotary evaporation. The crude product was purified by silica gel column chromatography (EtOAc/PE = 1:10), give compound **49** as white solid (71.6 mg, 89% yield). m.p. 94-96 °C. ^{1}H NMR (300 MHz, $CDCl_3$) δ 8.16-8.07 (m, 2H), 7.80-7.69 (m, 2H), 7.58-7.48 (m, 5H), 7.27 (d, J = 6.6 Hz, 2H), 7.11-7.05 (m, 2H), 5.22 (s, 1H), 3.86 (dd, J = 8.1, 6.6 Hz, 1H), 2.32 (s, 3H), 2.34-2.16 (m, 1H), 2.07-1.91 (m, 1H), 1.90 (d, J = 1.2 Hz, 3H), 0.84 (t, J = 7.2 Hz, 1H). ^{13}C NMR (75 MHz, $CDCl_3$) δ 156.0, 155.6, 152.1, 141.6, 140.5, 139.2, 138.0, 131.3, 129.72, 129.65, 129.29, 129.26, 129.15, 129.1,

128.9, 128.6, 120.8, 107.9, 93.6, 86.8, 53.1, 26.0, 21.6, 17.6, 12.7. HRMS (ESI) m/z calculated for $C_{29}H_{27}N_2$ $[M+H]^+$: 403.2174, found: 403.2171.

Synthesis of intermediate (-)-**36'**

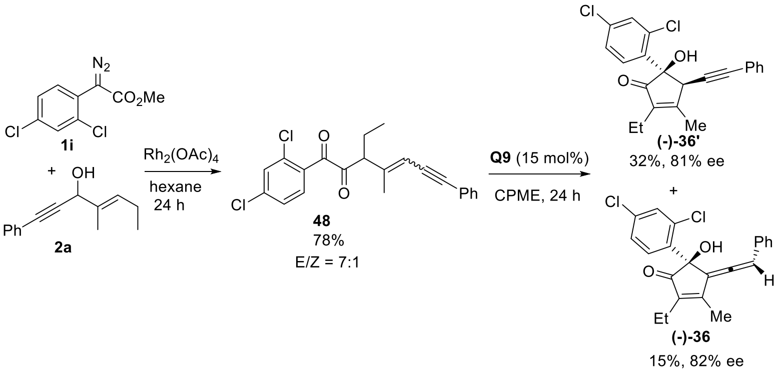

A flame dried tube was cooled to rt., evacuated and backfilled with argon. To the tube was added freshly distilled hexane (2.0 mL), diazo **1i** (0.3 mmol), enyn-3-ol **2a** (0.2 mmol), and then $Rh_2(OAc)_4$ (1.8 mg, 2 mol%) was added to the mixture solution. The tube was sealed and stirred at rt. until the enyn-3-ol disappear (24 h). Then, the solution was simple purified by short silica gel column, PE/EA = 20:1 as eluent, deliver the yellow oil **48** (60.2 mg, E/Z = 7:1, 78% yield), the E-isomer: ^{1}H NMR (400 MHz, $CDCl_3$) δ 7.50 (d, J = 8.3 Hz, 1H), 7.47-7.40 (m, 3H), 7.39-7.33 (m, 1H), 7.32-7.28 (m, 3H), 5.71 (s, 1H), 4.03 (dd, J = 8.2, 6.6 Hz, 1H), 2.01 (s, 3H), 1.93 (dt, J = 14.0, 6.9 Hz, 1H), 1.83-1.71 (m, 1H), 0.95 (t, J = 7.4 Hz, 3H). ^{13}C NMR (75 MHz, $CDCl_3$) δ 197.1, 191.3, 145.5, 139.5, 134.3, 132.4, 132.1, 131.3, 130.5, 128.3, 128.2, 127.5, 123.4, 111.5, 94.5, 86.6, 56.0, 22.3, 17.4, 11.8. HRMS (ESI) m/z calculated for $C_{22}H_{18}Cl_2NaO_2$ $[M+Na]^+$: 407.0576, found: 407.0575.

The compound **48** was dissolved in CPME and the **Q9** (15 mol%) was added to the solution. The tube was sealed and stirred for 24 h. The reaction solution was quenched with water, and extraction with ethyl acetate (3*10 mL). The combine organic phase was concentrated by rotary evaporation. Followed purified by silica gel column chromatography (EtOAc/PE = 1:10), give compound **(-)-36'** as colorless oil (24.6 mg, 32% yield, 81% ee). The enantiomeric excess was determined by HPLC analysis on a Daicel Chiralpak IC column (hexane/iPrOH = 95:05, flow rate: 1.0 mL/min, λ= 254 nm, t_R(major) = 6.54 min, t_R (minor) = 7.95 min. $[\alpha]_D^{25}$ = - 276.0 (c = 0.1 in $CHCl_3$). ^{1}H NMR (400 MHz, $CDCl_3$) δ 7.80 (s, 1H), 7.39-7.18 (m, 2H), 7.04 (dd, J = 7.7, 1.6 Hz, 2H), 3.99 (s, 1H), 3.26 (s, 1H), 2.38 (tq, J = 13.6, 6.8 Hz, 2H), 2.27 (s, 3H), 1.13 (t, J = 7.6 Hz, 3H). ^{13}C NMR (100 MHz, $CDCl_3$) δ 205.4, 139.4, 134.5, 131.4, 130.3, 129.7, 128.3, 128.3, 127.1, 122.8, 86.3, 83.9, 80.7, 50.6, 17.1, 15.7, 12.3. HRMS (ESI) m/z calculated for $C_{22}H_{18}Cl_2NaO_2$ $[M+Na]^+$: 407.0576, found: 407.0575.

Gram Scale for preparation of (-)-20

To the dried tube was added freshly distilled hexane (50.0 mL), diazo **1a** (1.32 g, 7.5 mmol, 1.5 eq.), enyn-3-ol **2a** (0.93 g, 5 mmol, 1eq.), and then $Rh_2(OAc)_4$ (65 mg, 2 mol%) was added to the mixture solution. The flask was sealed and stirred at rt. for 12 h. The solvent was removed directly; and then, the CPME (50 mL), **Q9** (15 mol%) and 4Å Ms (1000 mg) was added to the flask, stirred at rt. for another 72 h. The solution was concentrated under reduced pressure and purified by flash chromatography (EA:PE = 1:3) corresponding eluent to afford the desired products (-)-**20** (1.028 g, 68%).

Preparation of compound 50

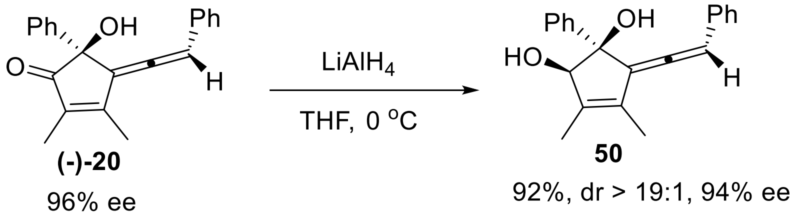

To the tube was added freshly THF (5.0 mL), (-)-**20** (30.2 mg, 0.1 mmol). The solution was cooled to 0 °C, the $LiAlH_4$ (9.2 mg, 0.2 mmol) was added to the mixture solution and stirred at the same temperature for 0.5 h. After complete consumption of the starting material (monitored by TLC), the mixture solution was quenched by H_2O (5 mL) and extracted with EtOAc (3 * 5 mL). The combined organic layer was washed with brine solution and dried over anhydrous Na_2SO_4, concentrated under reduced pressure and purified by silica gel column chromatography (EtOAc/PE = 1:5), give compound **50**, as white solid (27.7 mg, 92% yield, 94% ee). m.p. 132-134 °C. The enantiomeric excess was determined by HPLC analysis on a Daicel Chiralpak IC column (hexane/iPrOH = 90:10, flow rate: 1.0 mL/min, λ= 254 nm, t_R(major) = 15.72 min, t_R (minor) = 17.61 min. $[\alpha]_D^{17}$ = - 93.3 (c = 0.2 in $CHCl_3$). ^1H NMR (300 MHz, $CDCl_3$) δ 7.65-7.46 (m, 2H), 7.36-7.10 (m, 8H), 6.59 (s, 1H), 4.58 (d, J = 5.8 Hz, 1H), 2.50 (s, 1H, -OH), 1.89 (s, 3H), 1.83 (s, 3H), 1.22 (d, J = 7.0 Hz, 1H, -OH). ^{13}C NMR (75 MHz, $CDCl_3$) δ 201.3, 141.4, 137.9, 134.3, 132.2, 128.7, 128.1, 127.9, 127.5, 127.2, 124.5, 102.7, 87.0, 85.8, 12.5, 11.9. HRMS (ESI) calculated for $C_{21}H_{20}NaO_2$ $[M+H]^+$: 327.1356, found: 327.1355.

Preparation of compound 51:

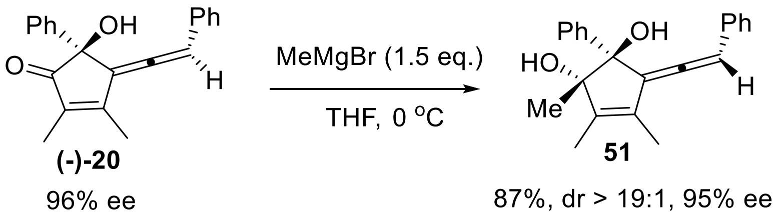

To the tube was added freshly THF (5.0 mL), (-)-**21** (30.2 mg, 0.1 mmol). The solution was cooled to 0 °C, the methyl magnesium bromide (13.5 mg, 0.3 mmol) was added to the mixture solution and stirred at the same temperature for 0.5 h. After complete consumption of the starting material (monitored by TLC), the mixture solution was quenched by H_2O (5 mL) and extracted with EtOAc (3 * 5 mL). The combined organic layer was washed with brine solution and dried over anhydrous Na_2SO_4, concentrated under reduced pressure and purified by silica gel column chromatography (EtOAc/PE = 1:5), give compound **51**, as white solid (27.9 mg, 87% yield, 95% ee), m.p. 141-142 °C. The enantiomeric excess was determined by HPLC analysis on a Daicel Chiralpak IC column (hexane/iPrOH = 50:50, flow rate: 1.0 mL/min, λ= 254 nm, t_R(major) = 6.04 min, t_R (minor) = 7.78 min. $[\alpha]_D^{17}$ = - 104.0 (c = 0.2 in $CHCl_3$). ^1H NMR (400 MHz, $CDCl_3$) δ 7.64-7.51 (m, 2H), 7.34-7.09 (m, 8H), 6.60 (s, 1H), 2.32 (s, 1H, -OH), 1.83 (s, 6H), 1.44 (s, 3H), 1.08 (s, 1H, -OH). ^{13}C NMR (75 MHz, $CDCl_3$) δ 200.9, 141.9, 141.5, 134.4, 130.2, 128.7, 128.0, 127.9, 127.5, 127.4, 127.2, 123.9, 102.8, 86.8, 86.1, 20.9, 11.9, 10.6. HRMS (ESI) m/z calculated for $C_{22}H_{22}NaO_2$ $[M+Na]^+$: 341.1512, found: 341.1516.

Preparation of compound 52:

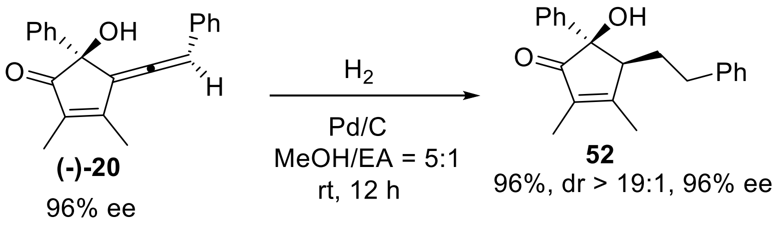

The **(-)-20** (30.2 mg, 0.1 mmol) was dissolved in EtOH:EtOAc = 5:1 (6.0 mL), Pd/C (15%wt, 4.5 mg) was added to the mixture solution, and the solution was then purged with hydrogen balloon and went 12 h under hydrogen balloon. Then, the reaction was filtered over a short path of Celite, concentrated in vacuo, and the crude mixture was purified by flash column chromatography (EtOAc/PE = 1/5), to afford the final product **52**, as white solid (29.4 mg, 96% yield, 96% ee), m.p. 102-104 °C. The enantiomeric excess was determined by HPLC analysis on a Daicel Chiralpak IC column (hexane/iPrOH = 95:05, flow rate: 1.0 mL/min, λ= 254 nm, t_R(major) = 9.33 min, t_R (minor) = 5.33 min. $[\alpha]_D^{17}$ = - 187.0 (c = 0.2 in CHCl3). ^{1}H NMR (400 MHz, $CDCl_3$) δ 7.39-7.27 (m, 5H), 7.18-7.07 (m, 3H), 6.75 (d, J = 7.2 Hz, 2H), 3.26 (brs, 1H), 2.96 (d, J = 11.6 Hz, 1H), 2.49-2.28 (m, 2H), 2.07 (s, 3H), 1.92-1.80 (m, 1H), 1.84 (s, 3H), 1.44-1.28 (m, 1H). ^{13}C NMR (100 MHz, $CDCl_3$) δ 208.1, 172.8, 142.1, 140.2, 134.9, 128.4, 128.3, 128.2, 127.8, 125.95, 125.88, 82.5, 55.7, 33.8, 30.0, 15.6, 8.6. HRMS (ESI) calculated for $C_{21}H_{23}O_2$ $[M+H]^+$: 307.1698, found: 307.1691.

Preparation of compound 53:

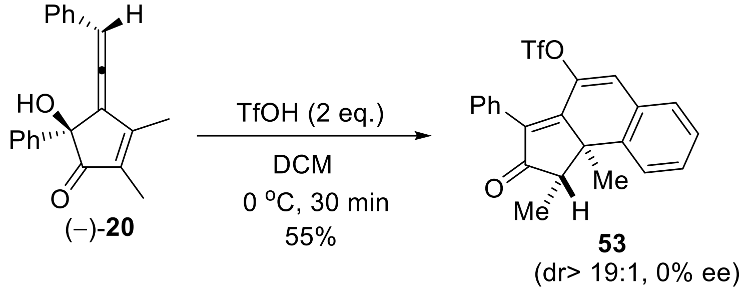

proposed pathway:

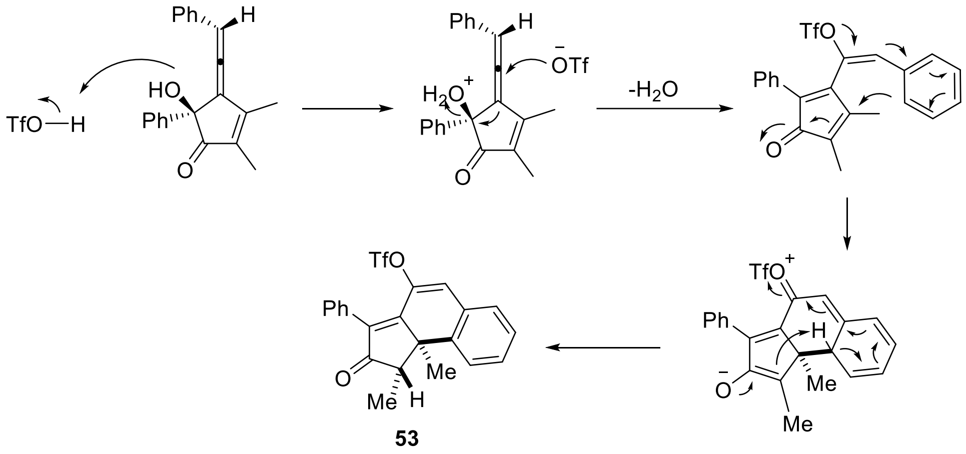

To the tube was added freshly DCM (3.0 mL), (-)-**20** (30.2 mg, 0.1 mmol). The solution was cooled to 0 °C, the TfOH (1.2 mmol, 12 eq.) was added to the mixture solution and stirred at the same temperature for 0.5 h. After complete consumption of the starting material (monitored by TLC), the mixture solution was quenched by sat.$NaHCO_3$ (10 mL) and extracted with EtOAc (3 * 10 mL). The combined organic layer was washed with brine solution and dried over anhydrous Na_2SO_4, concentrated under reduced pressure and purified by silica gel column chromatography (EtOAc/PE = 1:20), give compound **53**, as white solid, 14.5 mg, 55% yield, m.p. 114-117 °C. ^{1}H NMR (400 MHz, $CDCl_3$) δ 7.46-7.29 (m, 10H), 6.79(s, 1H), 3.11 (q, J = 7.2 Hz, 1H), 1.61 (d, J = 7.2 Hz, 3H), 1.46 (s, 3H). ^{13}C NMR (100 MHz, $CDCl_3$) δ 205.4, 160.2, 144.8, 142.4, 135.9, 130.9, 130.3, 130.2, 129.6, 128.98, 128.88, 128.1, 127.7, 126.0, 124.9, 51.2, 50.7, 29.4, 12.0. HRMS (ESI) calculated for $C_{22}H_{18}F_3O_4S$ $[M+H]^+$: 435.0877, found: 435.0872.

III. Supplementary Discussion

Supplementary table 1. Optimization of the method A [a]

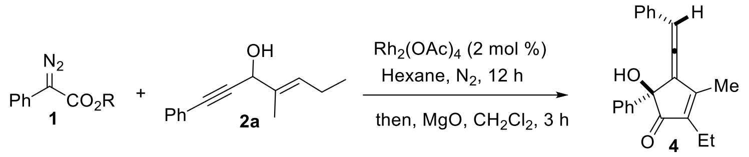

Entry	R	conditions	**3aa**[b]
1	Me	standard conditions	79%
2	Me	CH_2Cl_2 instead of hexane	31%
3	Me	Toluene instead of hexane	54%
4	Me	CHCN or MeOH or DMF instead of hexane	N.R.
5	Me	$Rh_2(esp)_4$ instead of $Rh_2(OAc)_4$	65%
6	Me	$Rh_2(TFA)_4$ instead of $Rh_2(OAc)_4$	63%
7	Me	$Rh_2(Oct)_4$ instead of $Rh_2(OAc)_4$	58%
8	Me	$Rh_2(Opiv)_4$ instead of $Rh_2(OAc)_4$	46%
9	Me	$Rh_2(DOSP)_4$ instead of $Rh_2(OAc)_4$	11%
10	Me	1 equiv. instead of 1.5 equiv. of **2a**	42%
11	Me	2 equiv. instead of 1.5 equiv. of **2a**	60%
12	Me	SiO_2 instead of MgO	43%
13	Me	Yb_2O_3 instead of MgO	N.P.
14	Me	Al_2O_3 instead of MgO	trace
15	Me	MnO_2 instead of MgO	N.P.
16	Me	Fe_3O_4 instead of MgO	trace
17	Me	CuO instead of MgO	trace
19	Me	performed in 0.05 M	72%
20	Me	performed in 0.2 M	62%
21	Me	performed in O_2	55%
22	Me	$Rh_2(OAc)_4$ (1 mol %)	70%
23	Me	$Rh_2(OAc)_4$ (4 mol %)	79%
24	Et	standard conditions	68%
25	^{i}Pr	standard conditions	55%
26	allyl	standard conditions	64%
27	Bn	standard conditions	25%
28	^{t}Bu	standard conditions	52%
29	Me	MgO (1 eq., 3 h)	12%

[a] General conditions: **1a** (0.3 mmol, 1.5 eq.), **2** (0.2 mmol, 1eq.) and [Rh] (2 mol %) in hexane (2 mL) stirred at rt for 12 h under N_2, then to the reaction solution were added MgO (200 mg, 50 eq.) and CH_2Cl_2 (5 mL) and then was stirred at 40 °C for another 3 h. [b] Isolated product, with dr > 19:1 determined by ^{1}H NMR analysis. N.R. = not reaction. N.P. = no product.

Supplementary table 2. Screening of the chiral organocatalysts [a]

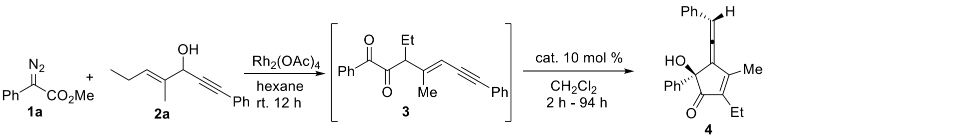

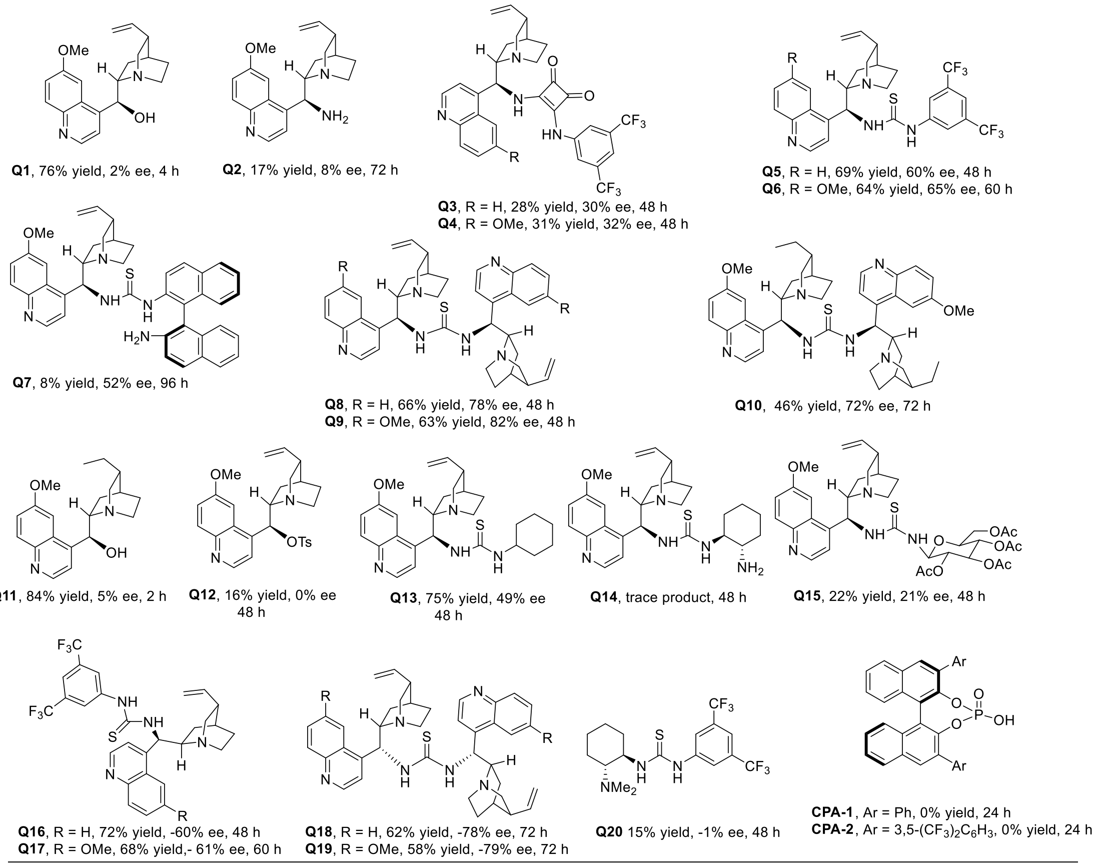

[a] Reaction conditions: **1a** (0.3 mmol, 1.5 eq.), **2a** (0.2 mmol, 1eq.), and $Rh_2(OAc)_4$ (2 mol%) in hexane (2 mL) stirred at rt. for 12 h under N_2, removed the solvent, and CH_2Cl_2 (2.0 mL), **Cat.** (10 mol%) and was added and stirred for 4-72 h. [b] Isolated yield for two steps; ee values were determined using chiral HPLC; Dr > 19:1, determined by crude ^1H NMR analysis.

Supplementary Table 3. Optimazition of the asymmertric conditions[a]

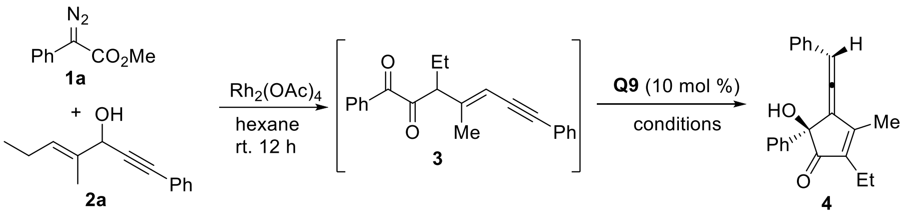

entry	solvent	additive	yield %[b]	ee %[c]
1	DCM	-	63	82
2	DCE	-	52	76
3	toluene	-	30	66
4	THF	-	N.P.	-
5	Et_2O	-	64	89
6	EA	-	15	79
7	Hexane	-	18	86
8	1,4-dioxane	-	N.P.	-
9	$^iPr_2O$	-	N.P.	-
10	^{t}BuOMe	-	59	83
11	CPME	-	62	91
12	CPME	3Å MS	60	89
13	CPME	4Å MS	61	95
14	CPME	5Å MS	58	90
15[d]	CPME	4Å MS	13	94
16[e]	CPME	4Å MS	64	92
17[f]	CPME	4Å MS	58	93
19[g]	CPME	4Å MS	63	95
20[h]	CPME	4Å MS	36	94
21[i]	CPME	4Å MS	65	95
22[j]	CPME	4Å MS	65	95
23	CPME/hexane =3 :1	4Å MS	56	94
24	CPME/DCM =3:1	4Å MS	58	90

[a] Reaction conditions: **1a** (0.3 mmol, 1.5 eq.), **2a** (0.2 mmol, 1eq.), and $Rh_2(OAc)_4$ (2 mol%) in hexane (2 mL) stirred at rt. for 12 h under N_2, removed the hexane, and new solvent (2.0 mL), **Q9** (10 mol%) and additives were added, stirred at rt. for 72 h. [b] Isolated yield for two steps; [c] Ee values were determined using chiral HPLC; dr > 19:1, determined by crude ^{1}H NMR analysis. [d] Performed at 0 °C for 72 h. [e] Performed at 40 °C for 72 h. [f] Conducted at 0.05 M (CPME, 4 ml). [g] Conducted at 0.2 M (CPME, 1 ml). [h] 5 mol% **Q9** was used. [i] 15 mol% **Q9** was used. [j] 20 mol% **Q9** was used.

Proposed mechanism of retained enantioselectivity in the alkyne-to-allene isomerization process

The proposed mechanistic model for the retained enantioselectivity in the alkyne-to-allene isomerization process under thiourea catalyst **Q9** and Lewis acidic SiO_2 as shown as follows (Supplementary Fig. 1):

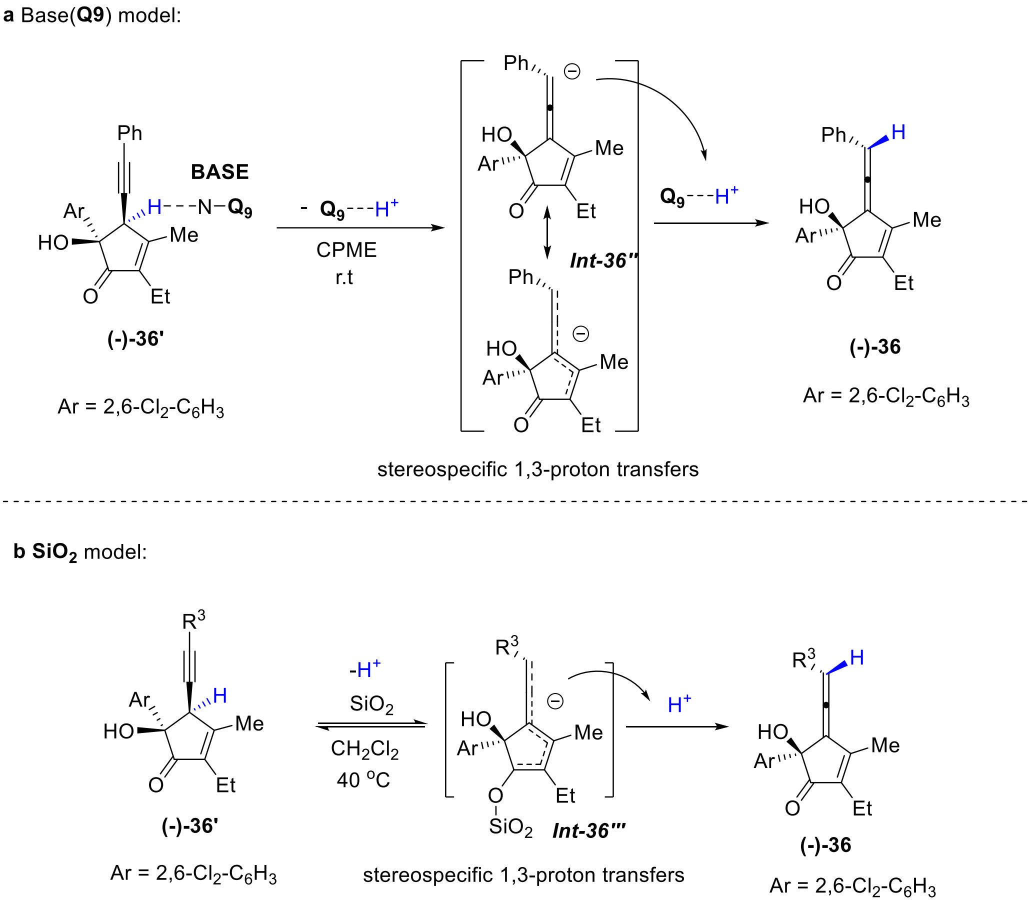

Supplementary Fig. 1 Proposed mechanism of retained enantioselectivity in the alkyne-to-allene isomerization process. **a.** the catalytic model of **Q9**: under the conditions of base **Q9**, deprotonation of (-)-**36'** was occurred, followed the process alkyne to allene isomerization to generates the intermediates ***int-36''***. And then the chiral anion subjected protonation process to afford the target product **(-)-36**, with enantioselective retained. **b.** the catalytic model of SiO2: In the presence of SiO2 and a temperature of 40 °C in solvent of CH_2Cl_2, (-)-**36'** occurred deprotonation to form ***int-36'''***, and then through an stereospecific transfers process to give the **(-)-36**, with enantioselective retained.

Supplementary Table 4. Crystal data and structure refinement for **(-)-20**

X-ray of (-)-20 (method of crystallization: In a 10 mL tube, the compound (-)-**20** (30 mg, >99% ee) was dissolved in a mixture n-hexane/CH_2Cl_2 (5 mL: 1 mL). The tube was sealed with a septum and a needle was inserted into the septum in order to slowly evaporation of CH_2Cl_2). Thermal ellipsoids are shown at the 50% level.

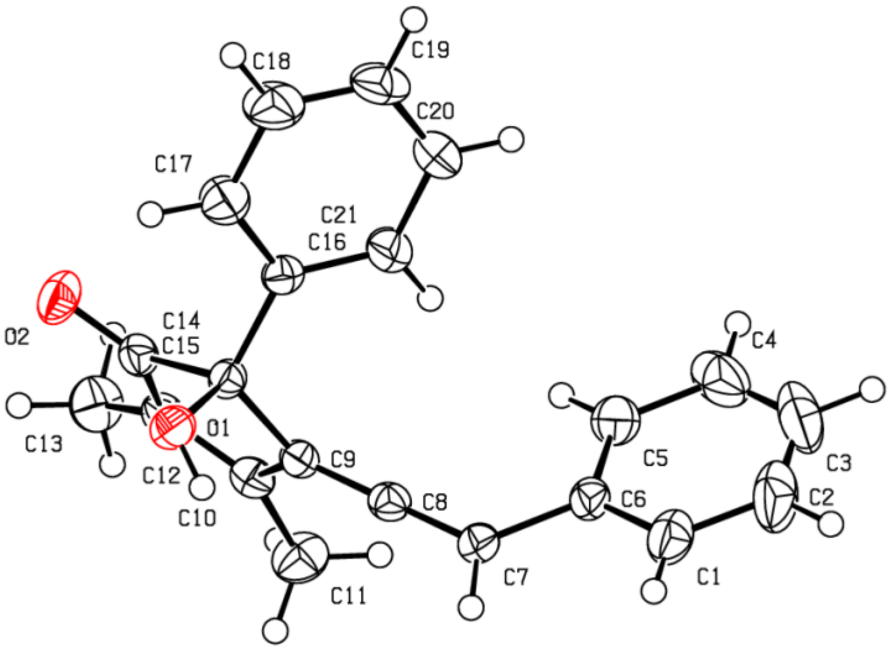

Crystallographic data of (-)-20

Empirical formula	$C_{21}H_{18}O_2$
Formula weigh	302.35
Wavelength	1.34139
Crystal system	monoclinic
Space group	P21
a (Å)	10.2784(3)
b (Å)	14.3841(4)
c (Å)	10.9798(3)
α (°)	90.00
β (°)	93.815(1)
γ (°)	90.00
V (Å^3)	1619.72(8)
Z	4
Temperature/K	193
F (000)	640.0
Crystal size/mm^3	0.10× 0.20 × 0.10
θ min, θ max (deg)	1.294, 60.638
Reflections collected	7101
Independent reflections	7309
Data/restraints/parameters	7101/0/421
Goodness-of-fit on F2	1.033
Final R indexes [I>=2σ (I)]	R1 = 0.0320, wR2 = 0.0853
Final R indexes [all data]	R1 = 0.0331, wR2 = 0.0881
Largest diff. peak and hole/ e Å^{-3}	0.188/-0.151
Flack parameter	0.08(5)

Supplementary Table 5. Crystal data and structure refinement for compound **49**

X-ray of 49 (method of crystallization: In a 10 mL tube, the compound **49** (30 mg) was dissolved in a mixture n-hexane/CH_2Cl_2 (5 mL: 1 mL). The tube was sealed with a septum and a needle was inserted into the septum in order to slowly evaporation of the mixture solvent). Thermal ellipsoids are shown at the 50% level.

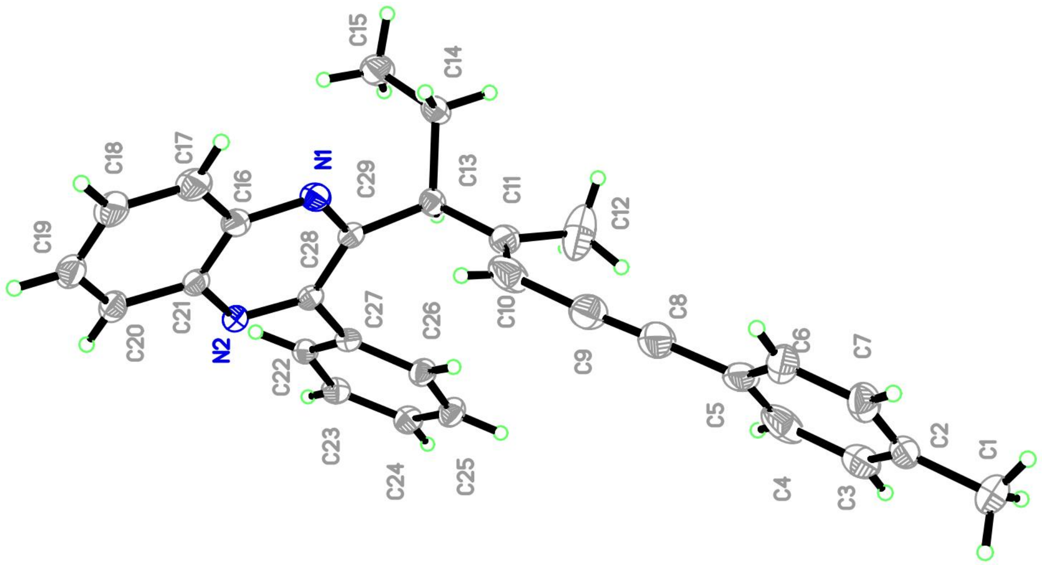

Crystallographic data of 49

Empirical formula	$C_{29}H_{26}N_2$
Formula weigh	402.52
Wavelength	0.71073
Crystal system	orthorhombic
Space group	P21
a (Å)	15.1266(12)
b (Å)	6.2486(4)
c (Å)	23.8425(17)
α (°)	90.00
β (°)	90.00
γ (°)	90.00
V (Å^3)	2253.6(3)
Z	4
Temperature/K	296(2)
F (000)	856.0
Crystal size/mm^3	0.160× 0.140 × 0.110
θ min, θ max (deg)	2.18, 27.553
Reflections collected	3729
Independent reflections	5161
Data/restraints/parameters	5161/0/283
Goodness-of-fit on F2	1.049
Final R indexes [I>=2σ (I)]	R1 = 0.0843, wR2 = 0.2165
Final R indexes [all data]	R1 = 0.1164, wR2 = 0.2426
Largest diff. peak and hole/ e Å^{-3}	0.921/-0.389

Supplementary Table 6. Crystal data and structure refinement for compound 53

X-ray of (-)-49 (method of crystallization: In a 10 mL tube, the compound **53** (50 mg) was dissolved in a mixture n-hexane/CH_2Cl_2 (5 mL: 1 mL). The tube was sealed with a septum and a needle was inserted into the septum in order to slowly evaporation of the mixture solvent). Thermal ellipsoids are shown at the 50% level.

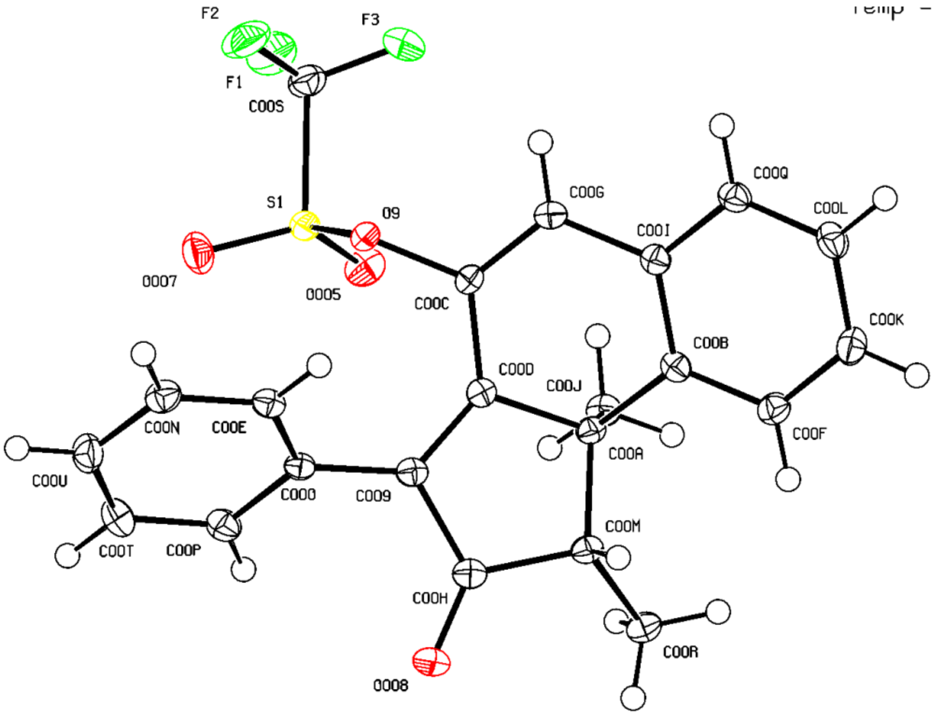

Crystallographic data of 53

Empirical formula	$C_{22}H_{17}F_3O_4S$
Formula weigh	434.41
Wavelength	1.54184
Crystal system	monoclinic
Space group	P21
a (Å)	9.38449(4)
b (Å)	19.59615(9)
c (Å)	10.45365(6)
α (°)	90.00
β (°)	96.8839(5)
γ (°)	90.00
V (Å^3)	1908.566(16)
Z	4
Temperature/K	100
F (000)	896.0
Crystal size/mm^3	0.21×0.19×0.18
θ min, θ max (deg)	5.257, 75.868
Reflections collected	3745
Independent reflections	3815
Data/restraints/parameters	3815/0/273
Goodness-of-fit on F2	1.026
Final R indexes [I>=2σ (I)]	R1 = 0.0304, wR2 = 0.0764
Final R indexes [all data]	R1 = 0.0300, wR2 = 0.0761
Largest diff. peak and hole/ e Å^{-3}	0.313/-0.355

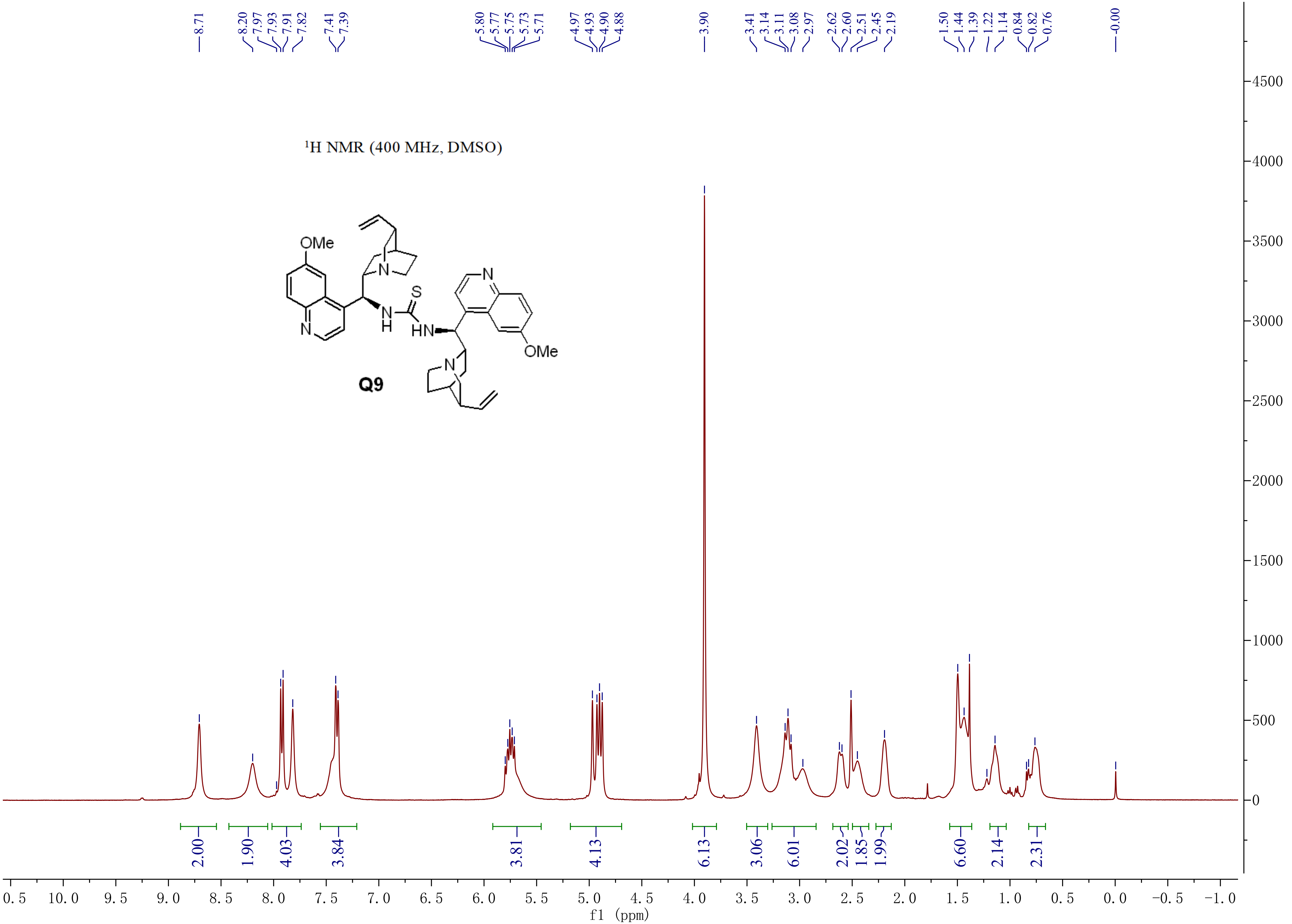

Supplementary Figure 7. ¹H NMR of compound Q9.

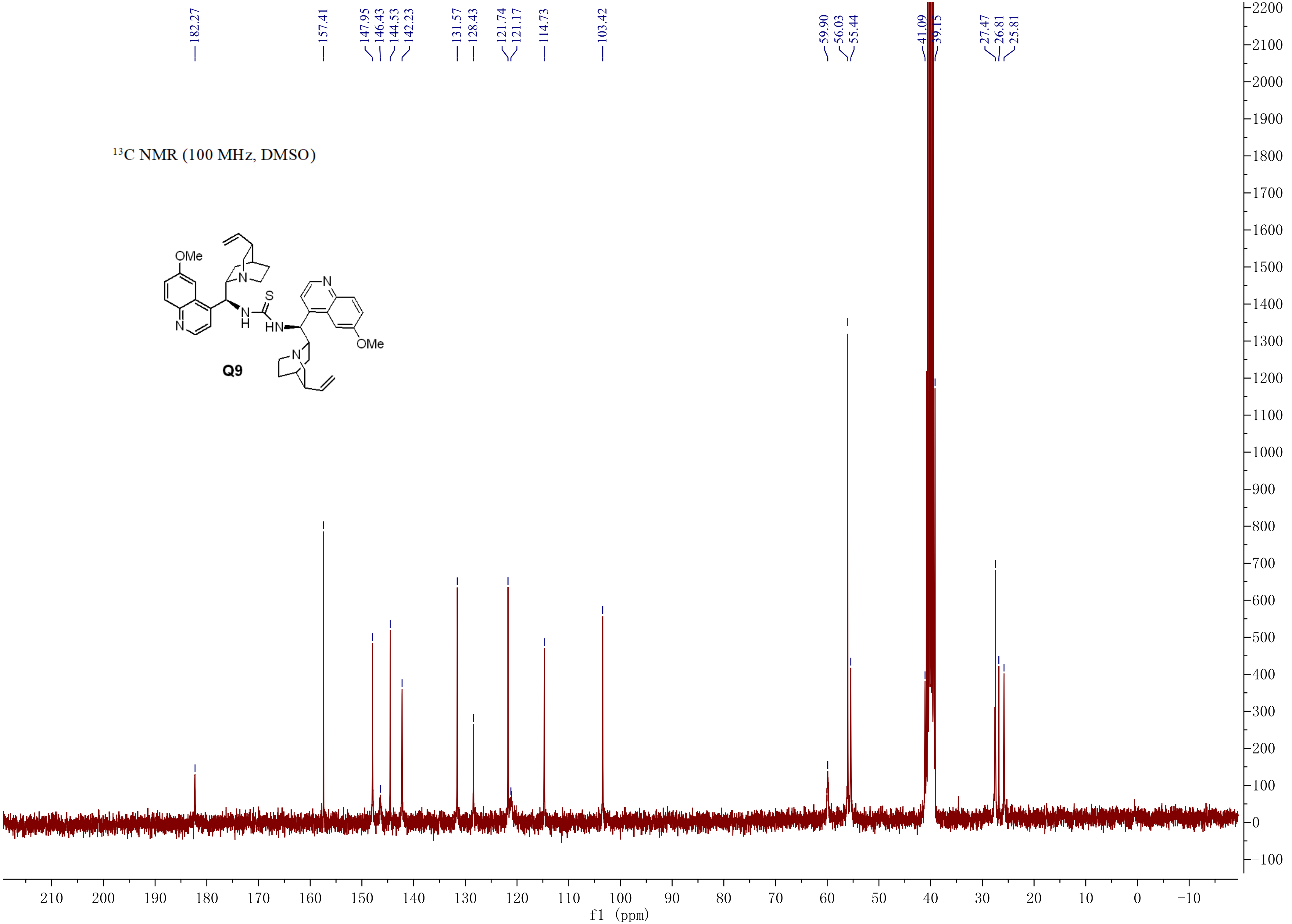

Supplementary Figure 8. ^{13}C NMR of compound Q9.

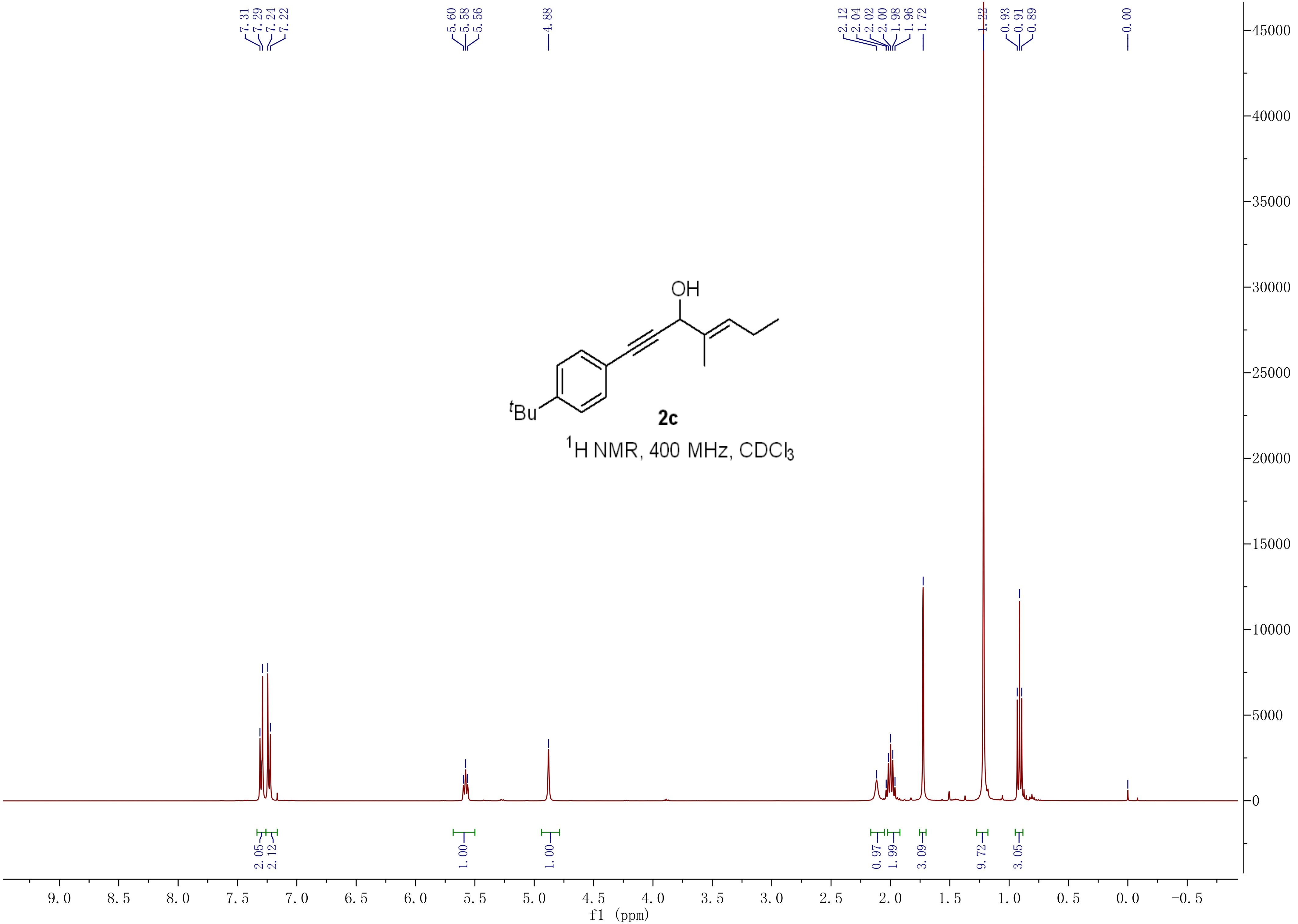

Supplementary Figure 9. ^{1}H NMR of compound 2c.

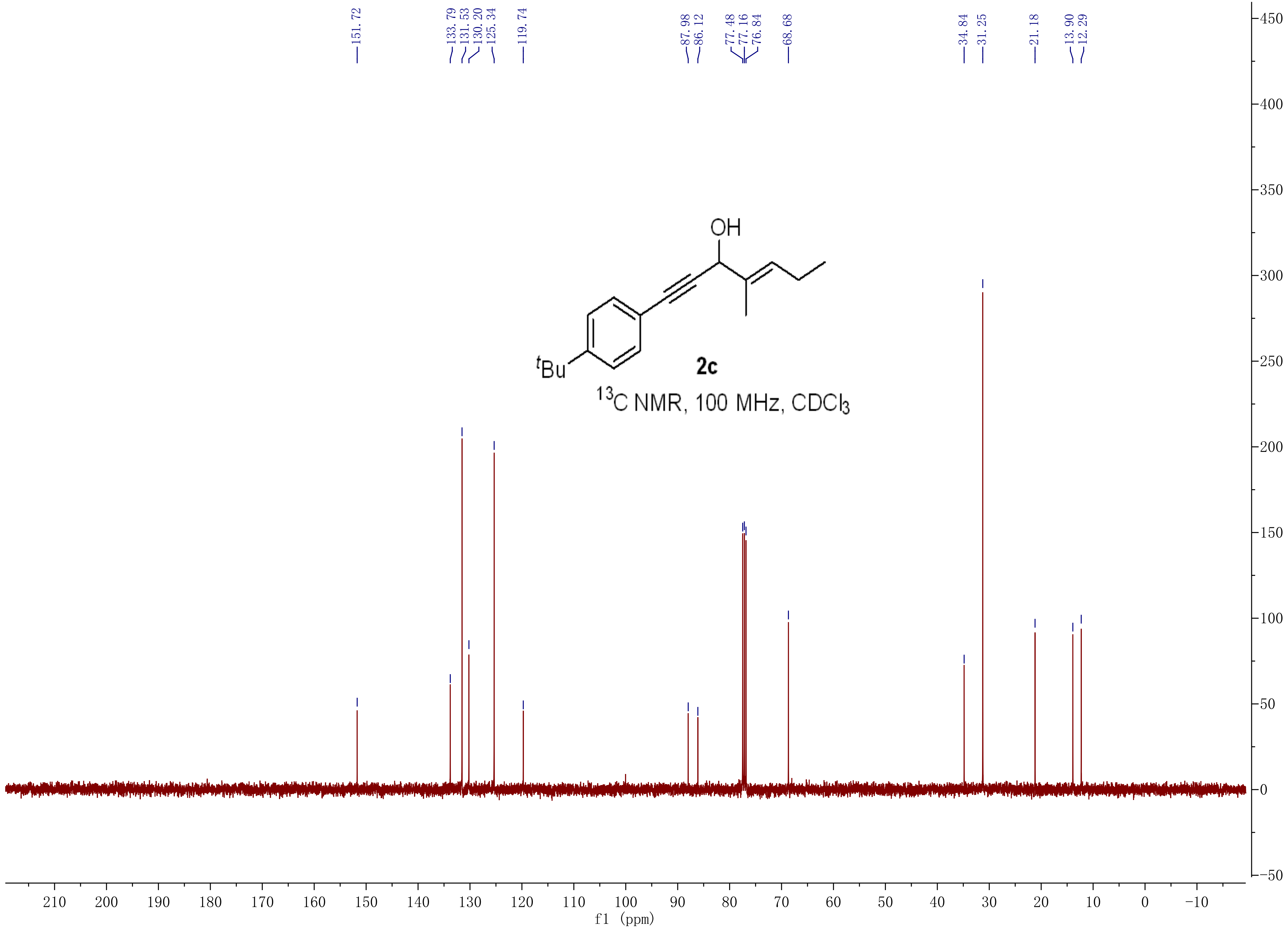

Supplementary Figure 10. ^{13}C NMR of compound 2c.

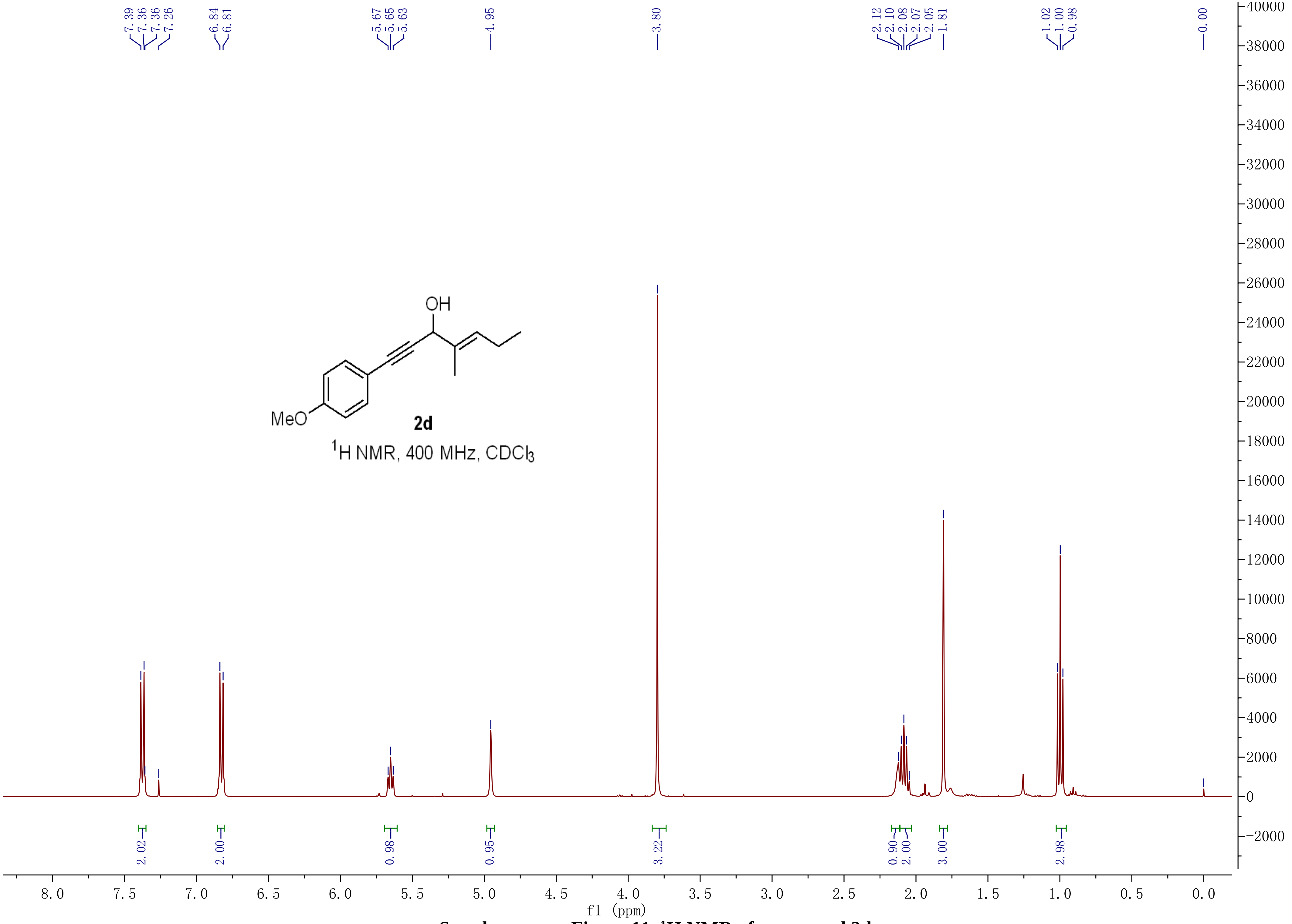

Supplementary Figure 11. ^{1}H NMR of compound 2d.

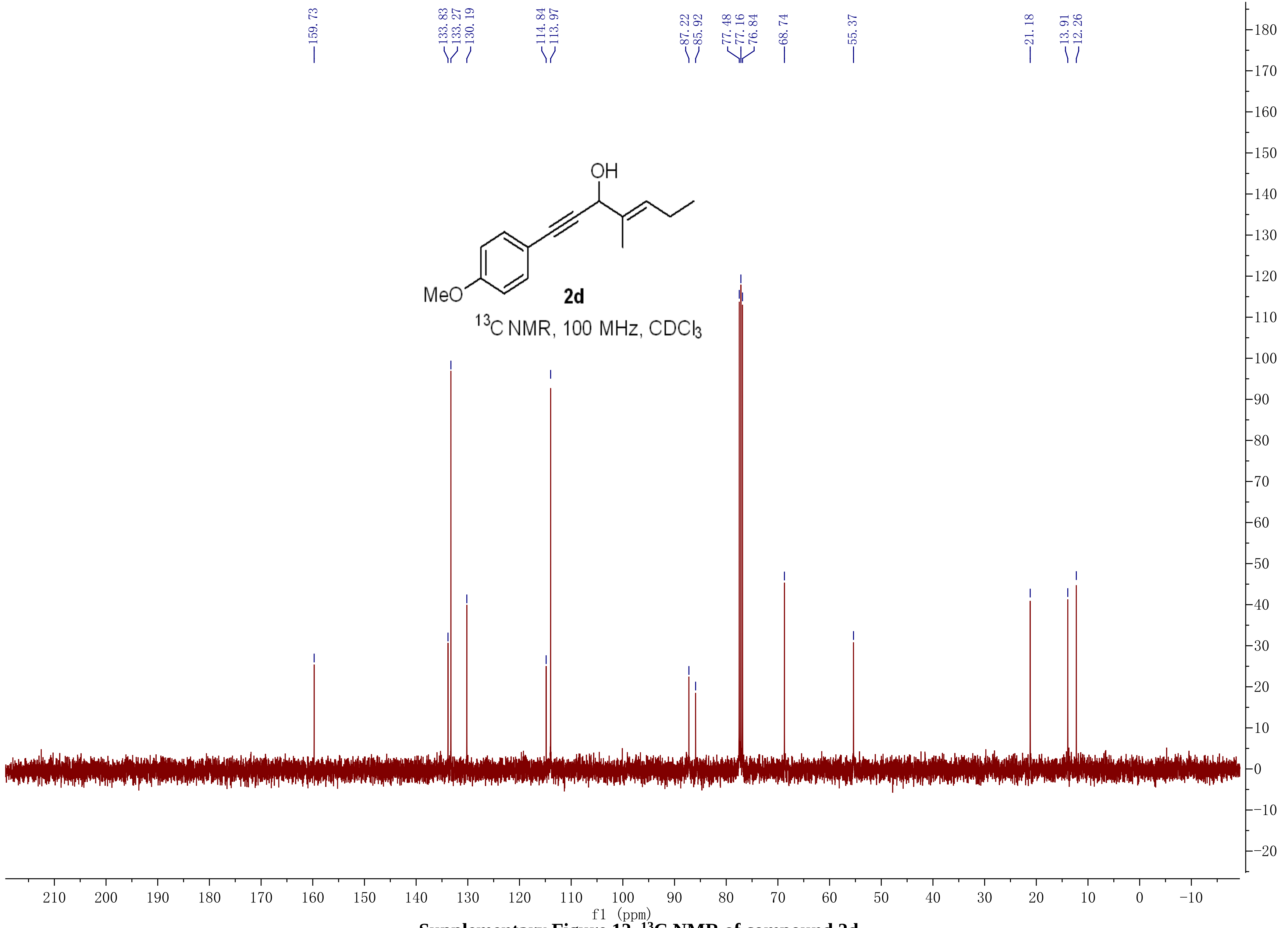

Supplementary Figure 12. ^{13}C NMR of compound 2d.

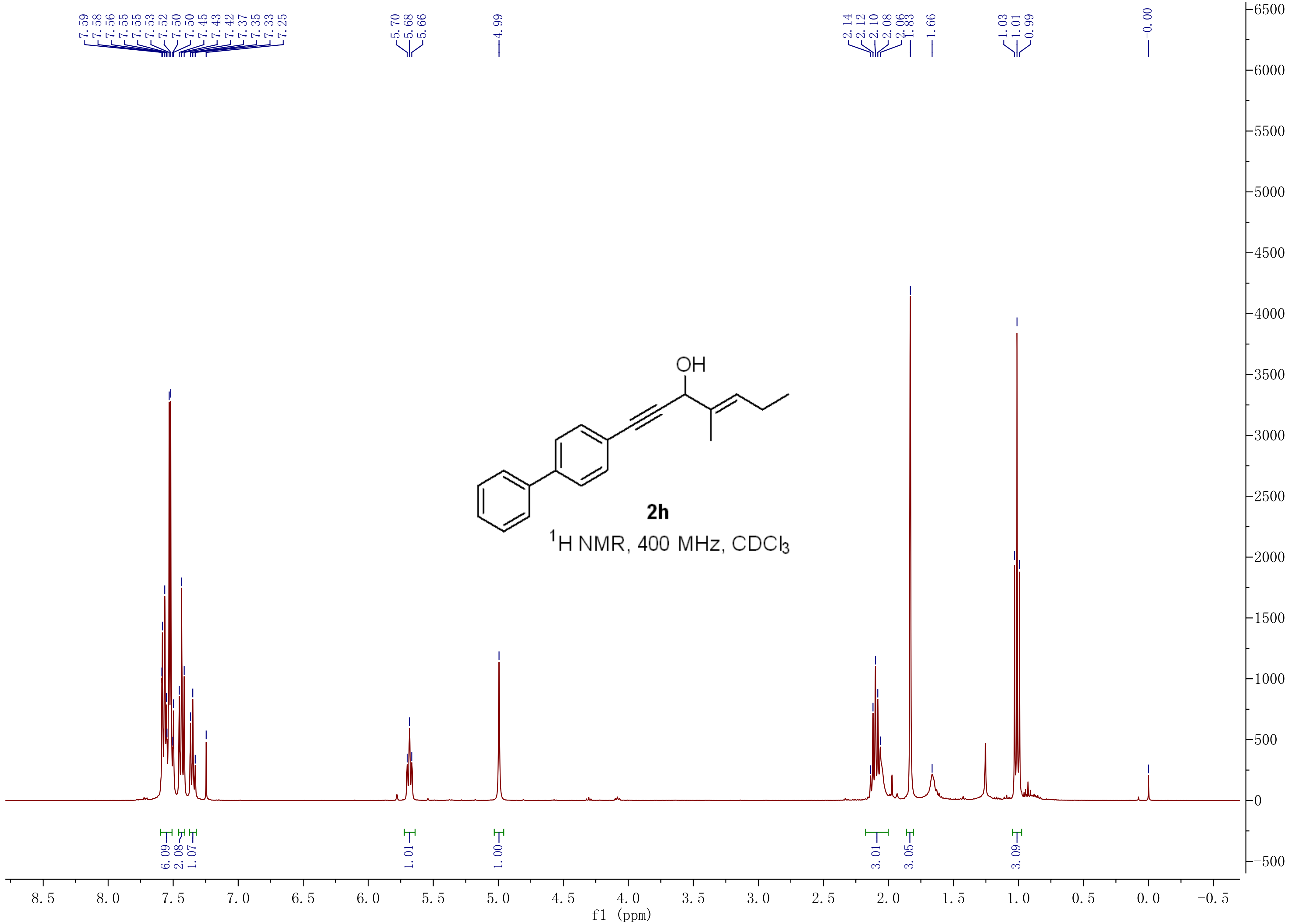

Supplementary Figure 13. ^{1}H NMR of compound 2h.

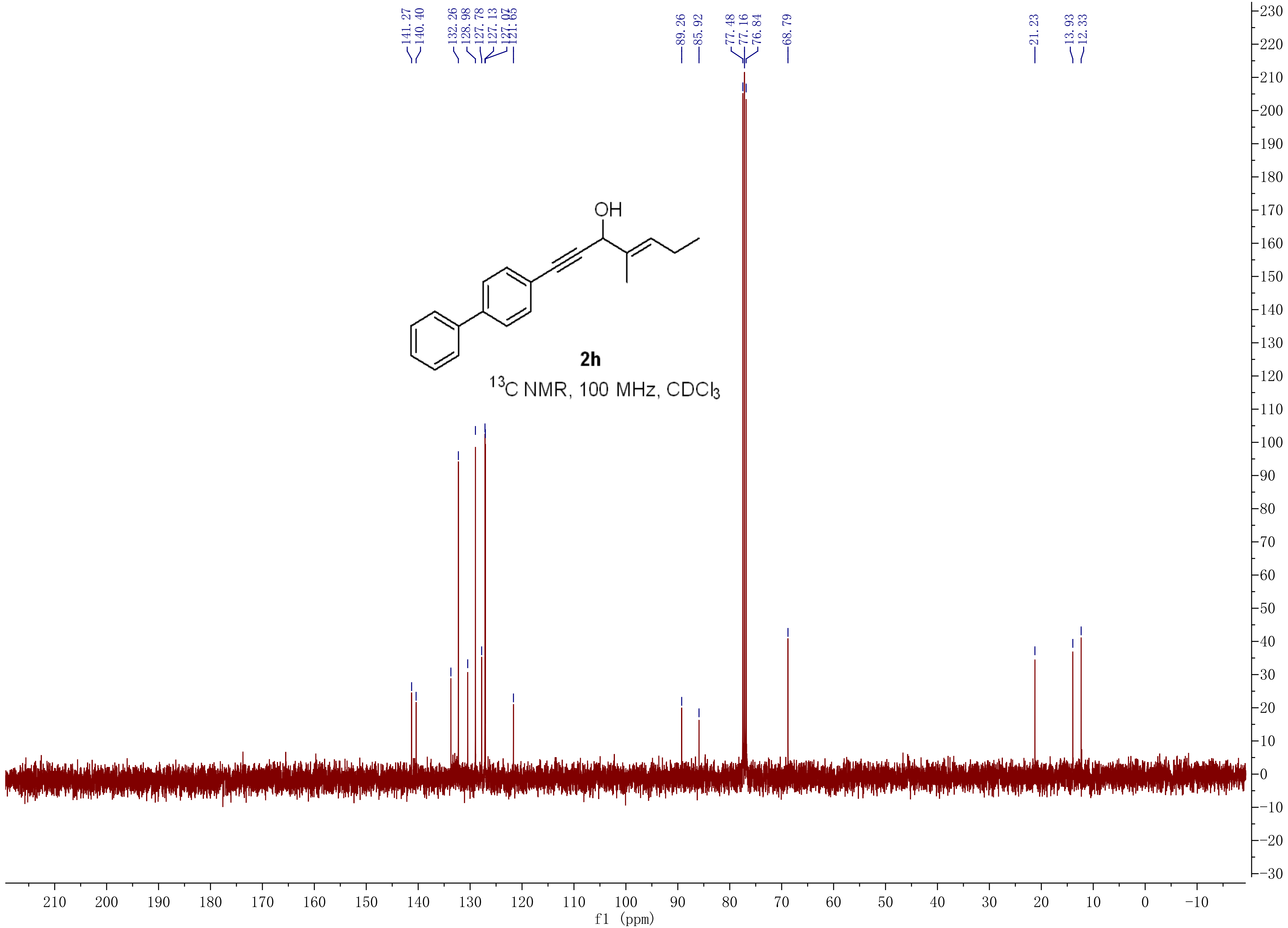

Supplementary Figure 14. ^{13}C NMR of compound 2h.

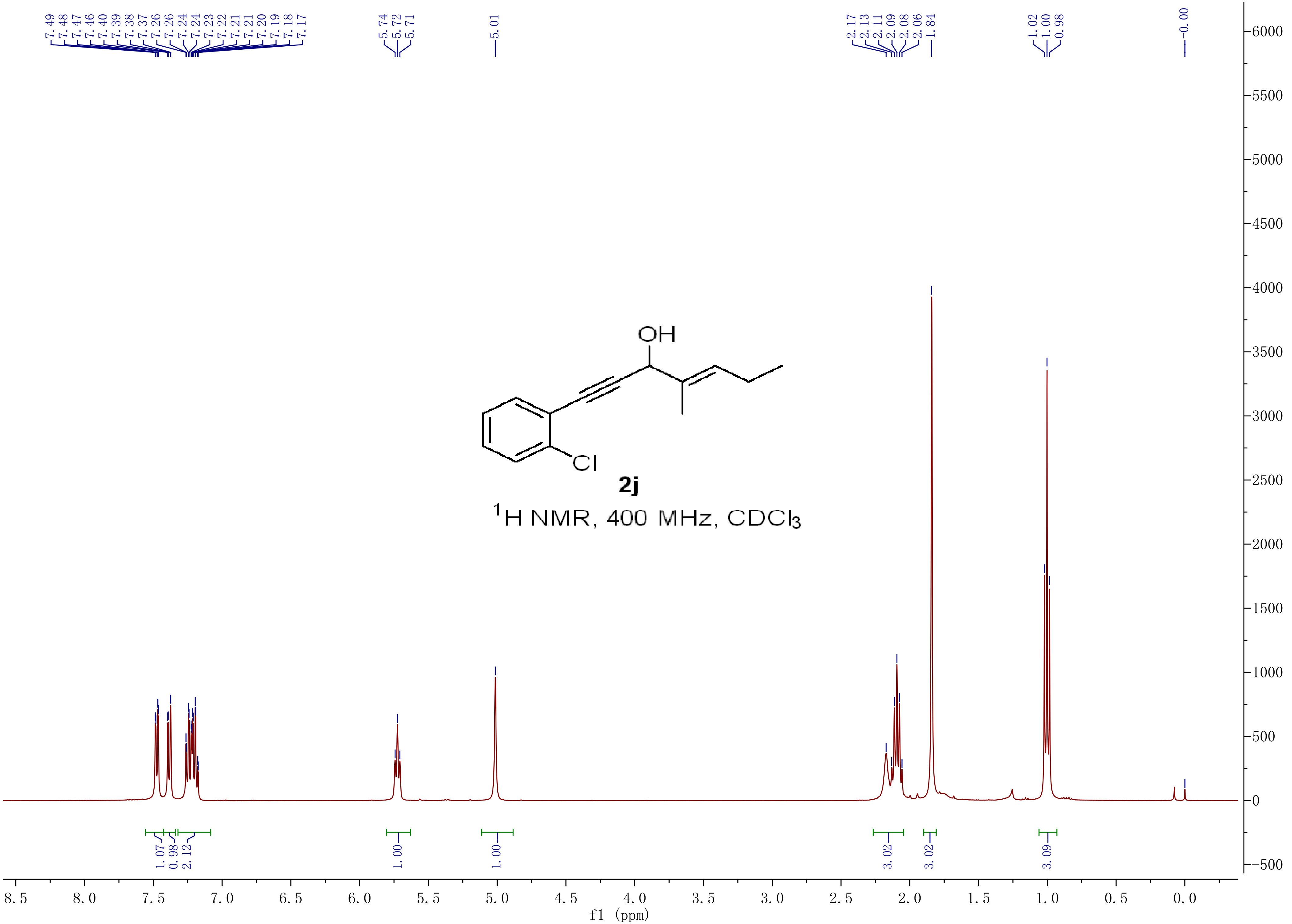

Supplementary Figure 15. ^{1}H NMR of compound 2j.

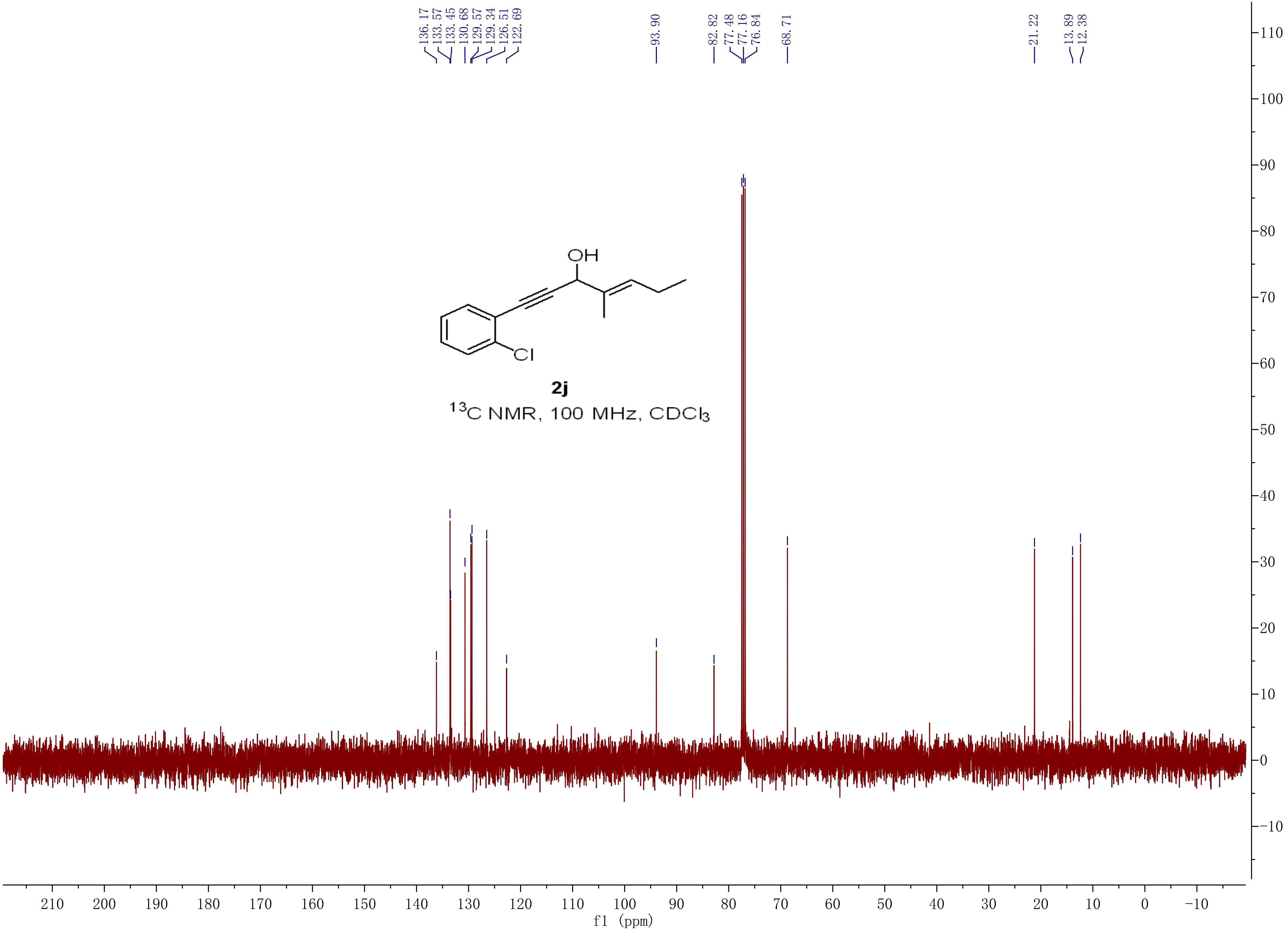

Supplementary Figure 16. ^{13}C NMR of compound 2j.

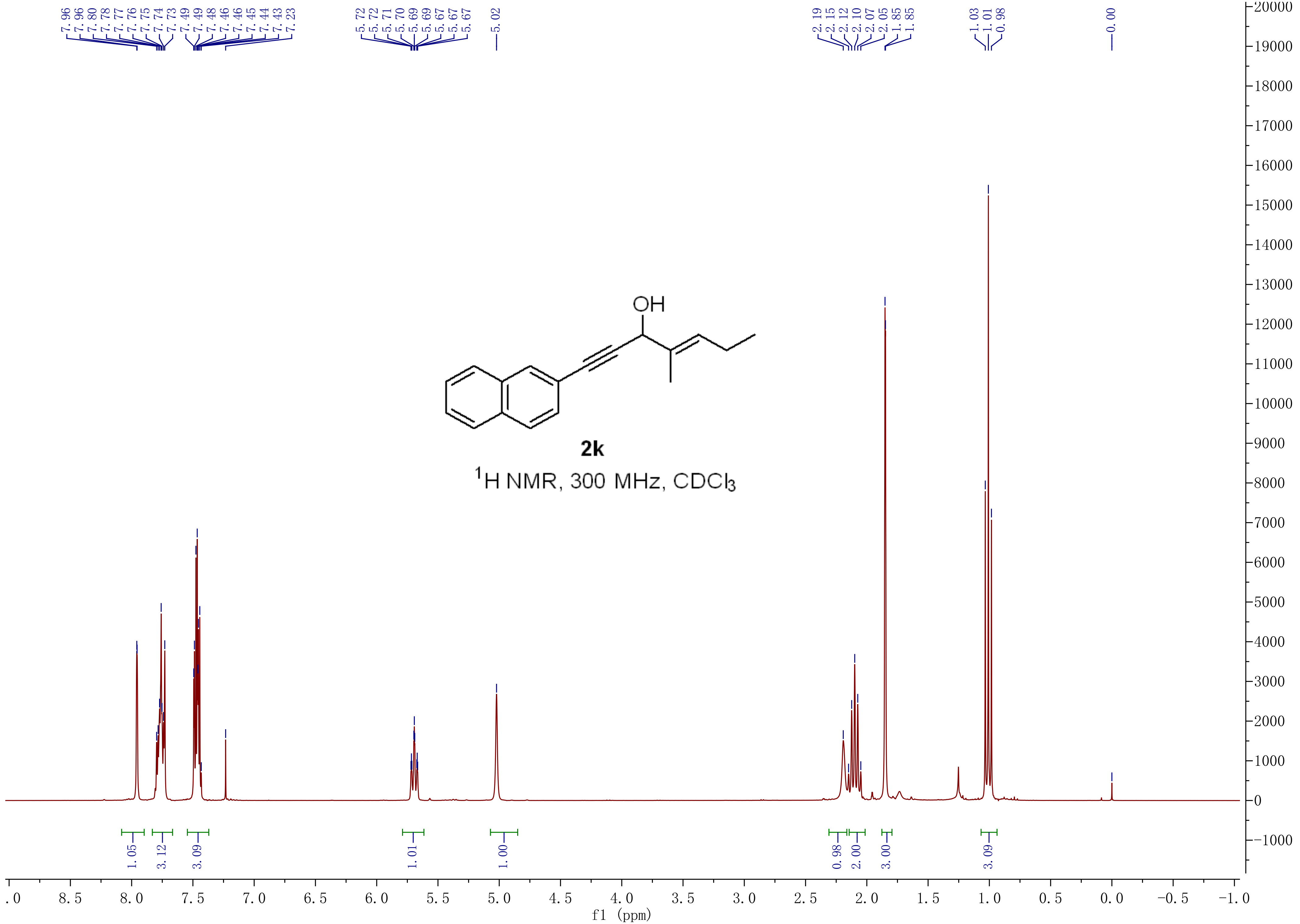

Supplementary Figure 17. ^{1}H NMR of compound 2k.

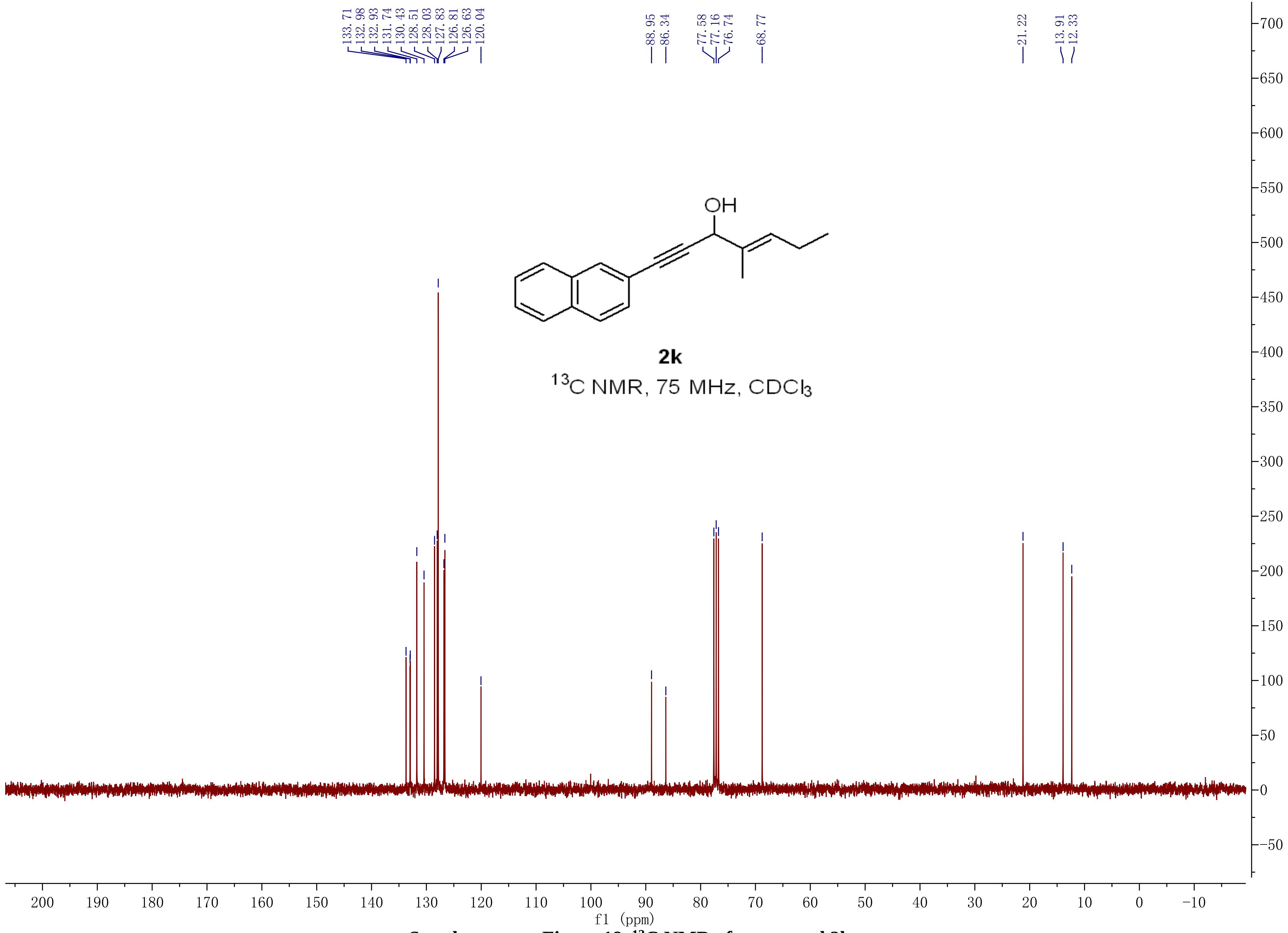

Supplementary Figure 18. ^{13}C NMR of compound 2k.

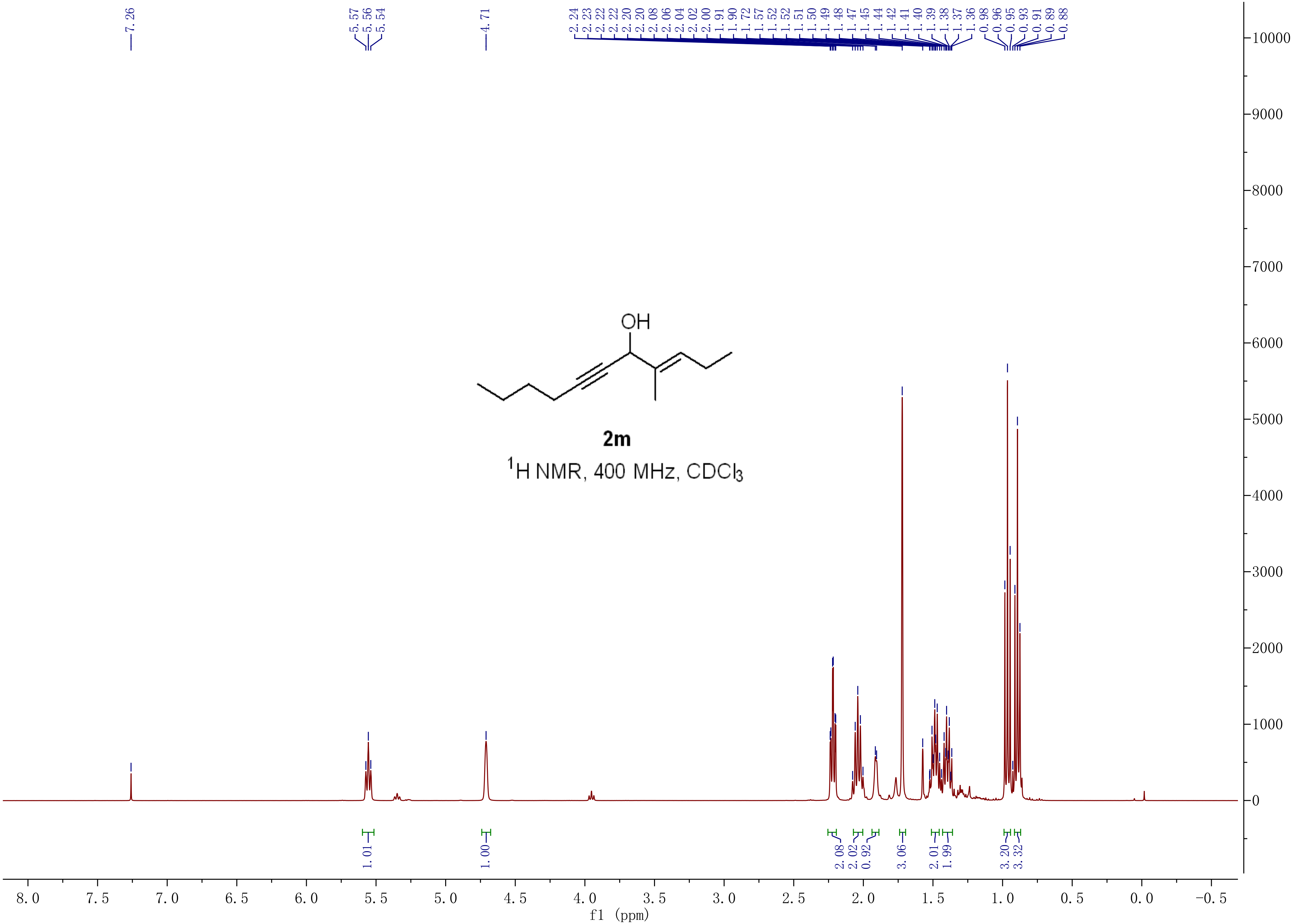

Supplementary Figure 19. ^{1}H NMR of compound 2m.

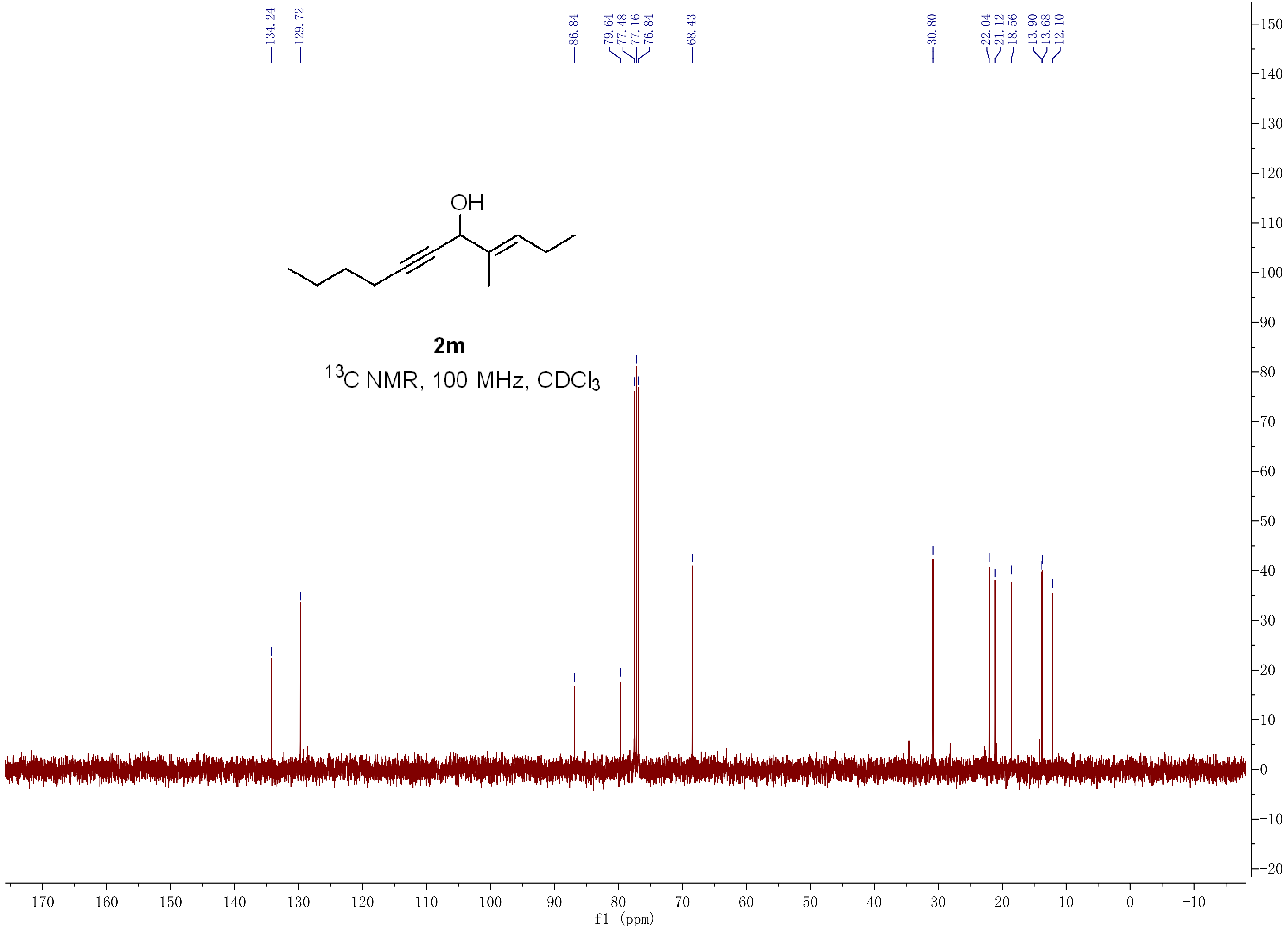

Supplementary Figure 20. ^{13}C NMR of compound 2m.

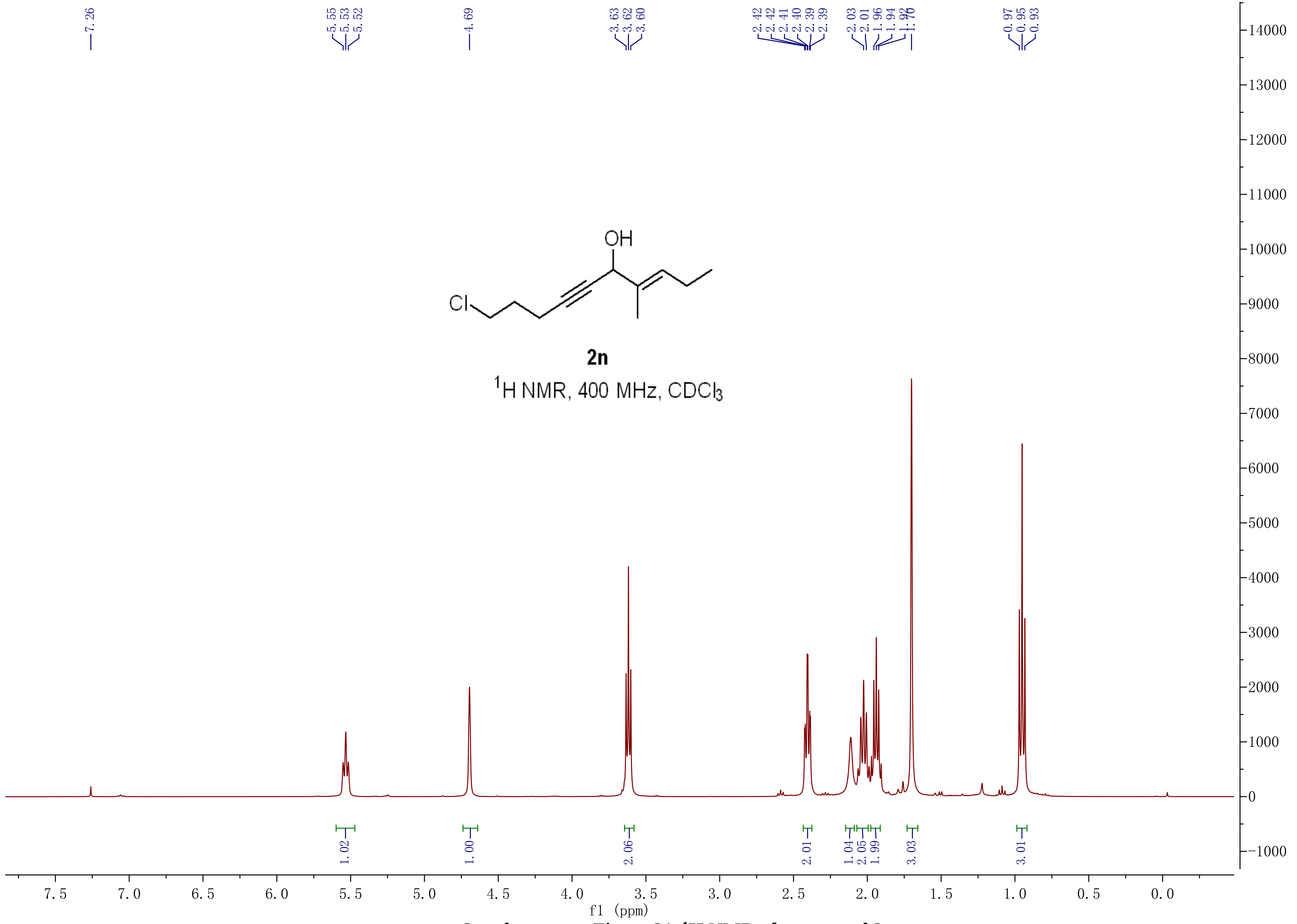

Supplementary Figure 21. ^{1}H NMR of compound 2n.

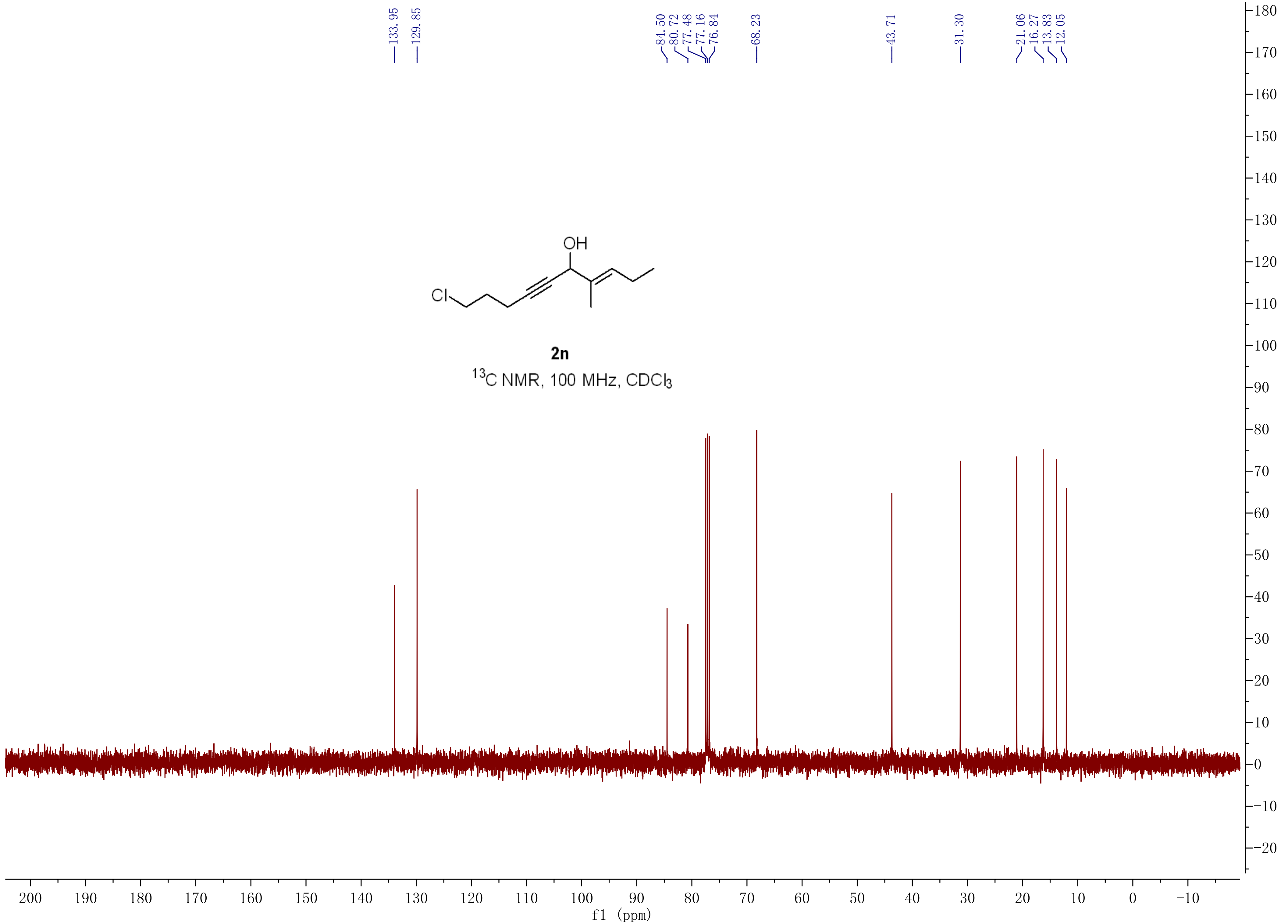

Supplementary Figure 22. ^{13}C NMR of compound 2n.

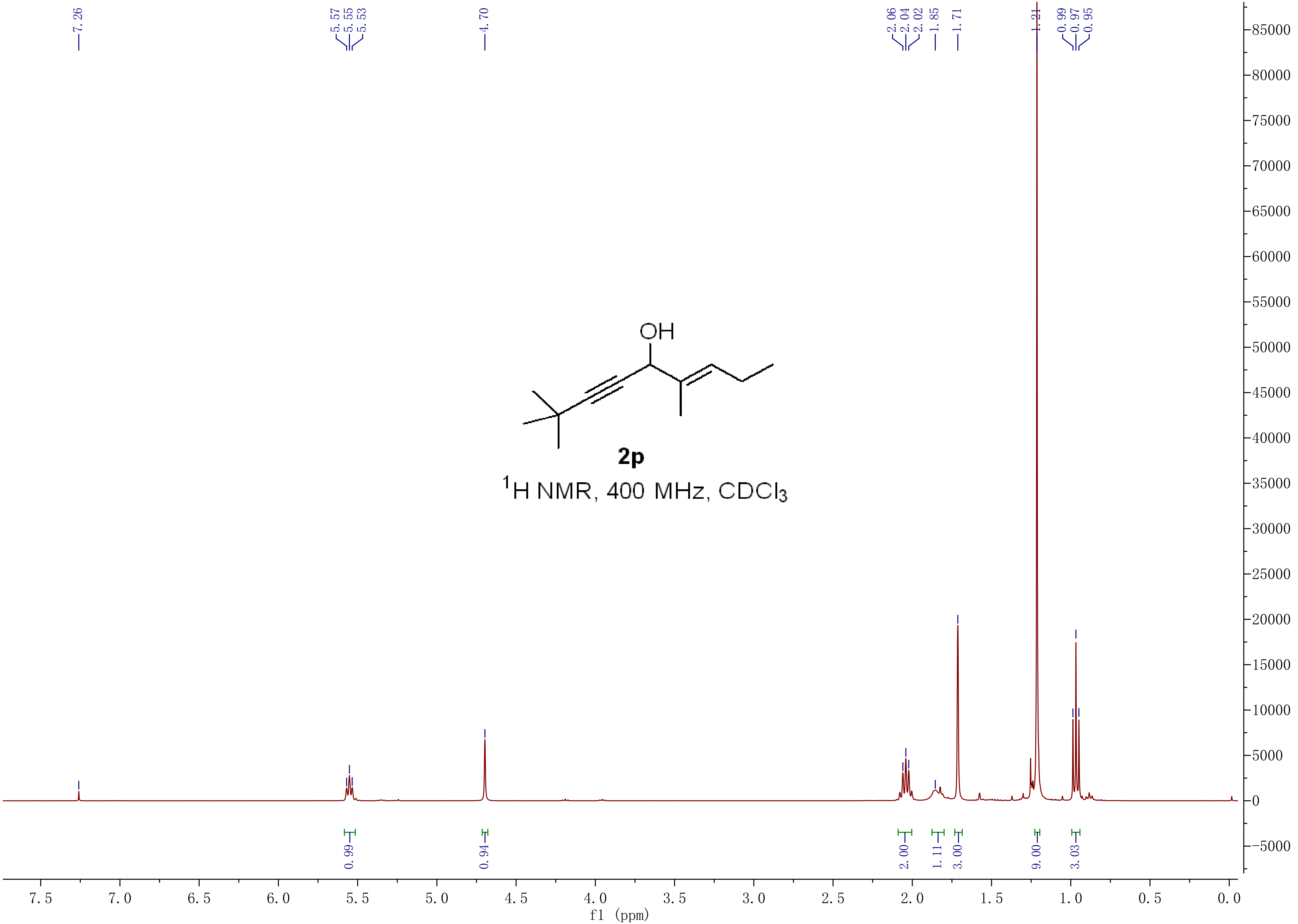

Supplementary Figure 23. ¹H NMR of compound 2p.

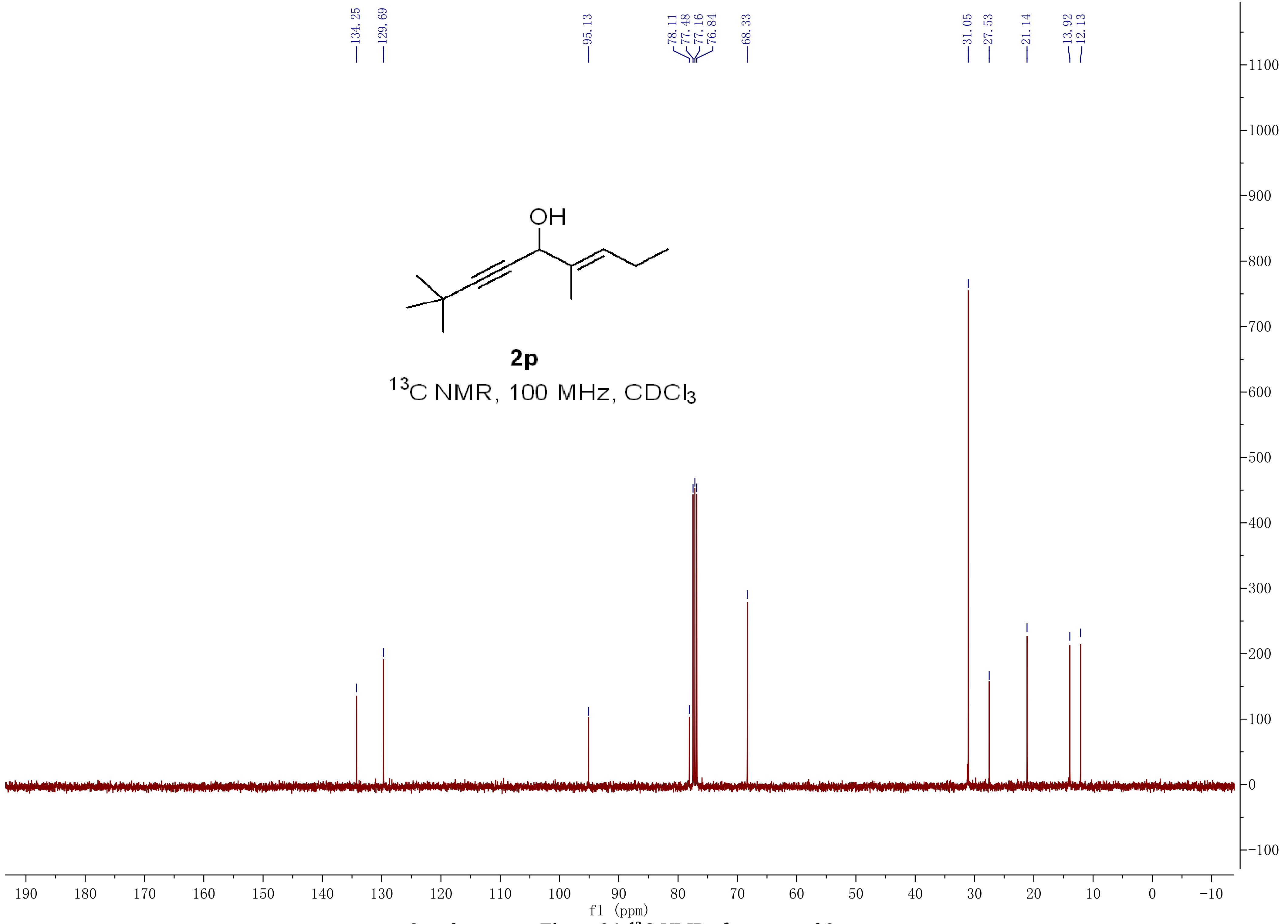

Supplementary Figure 24. ^{13}C NMR of compound 2p.

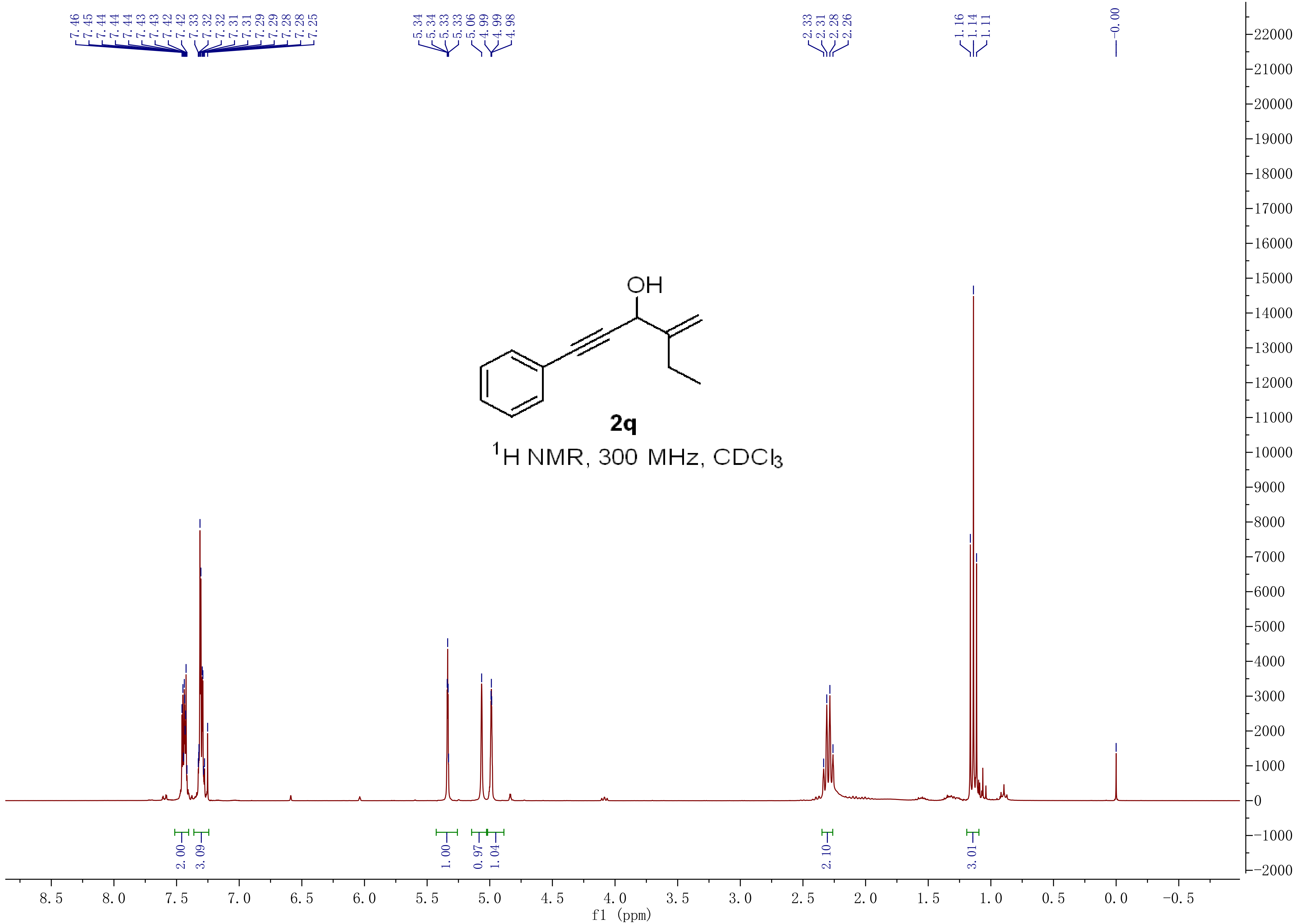

Supplementary Figure 25. ^{1}H NMR of compound 2q.

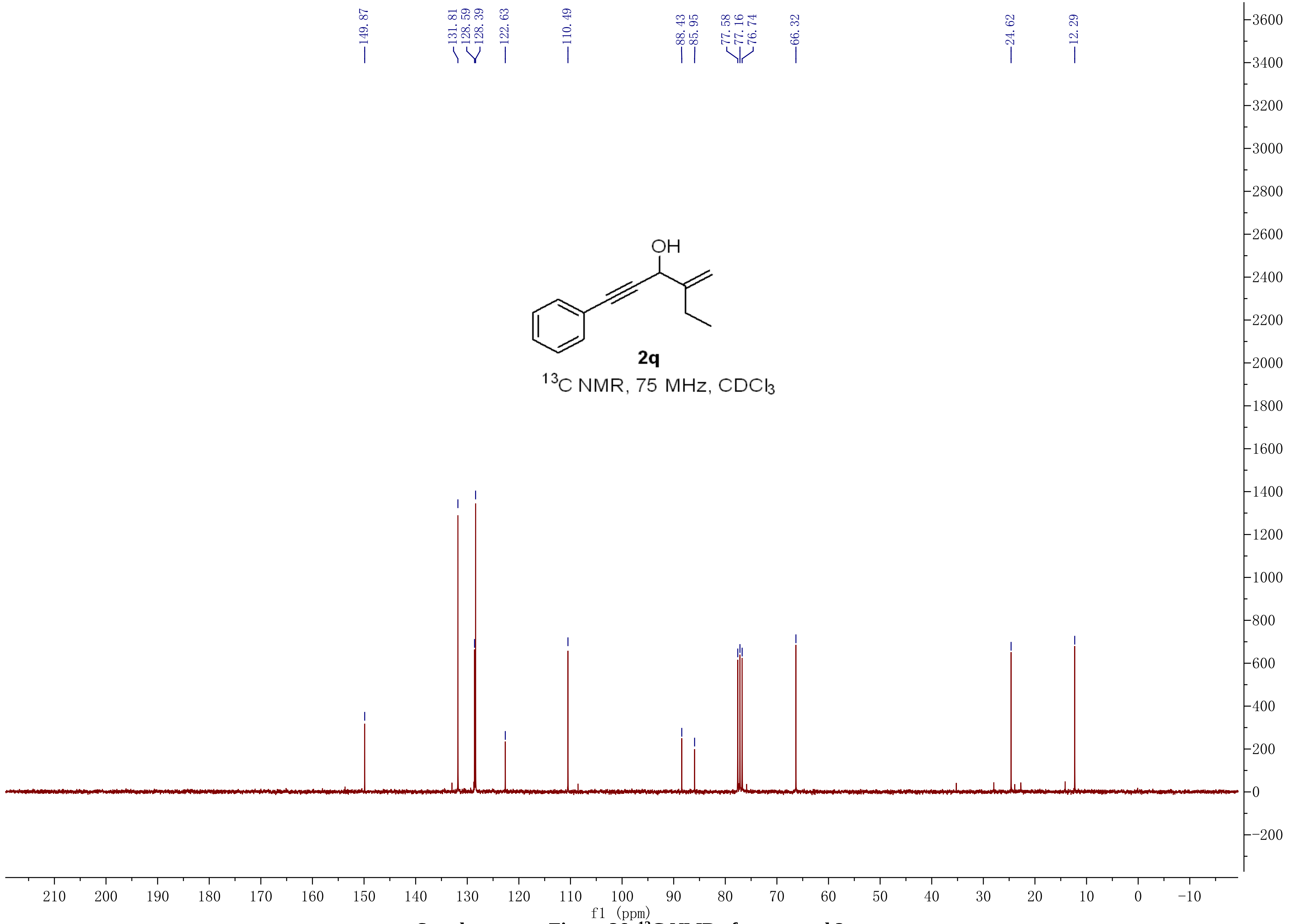

Supplementary Figure 26. ^{13}C NMR of compound 2q.

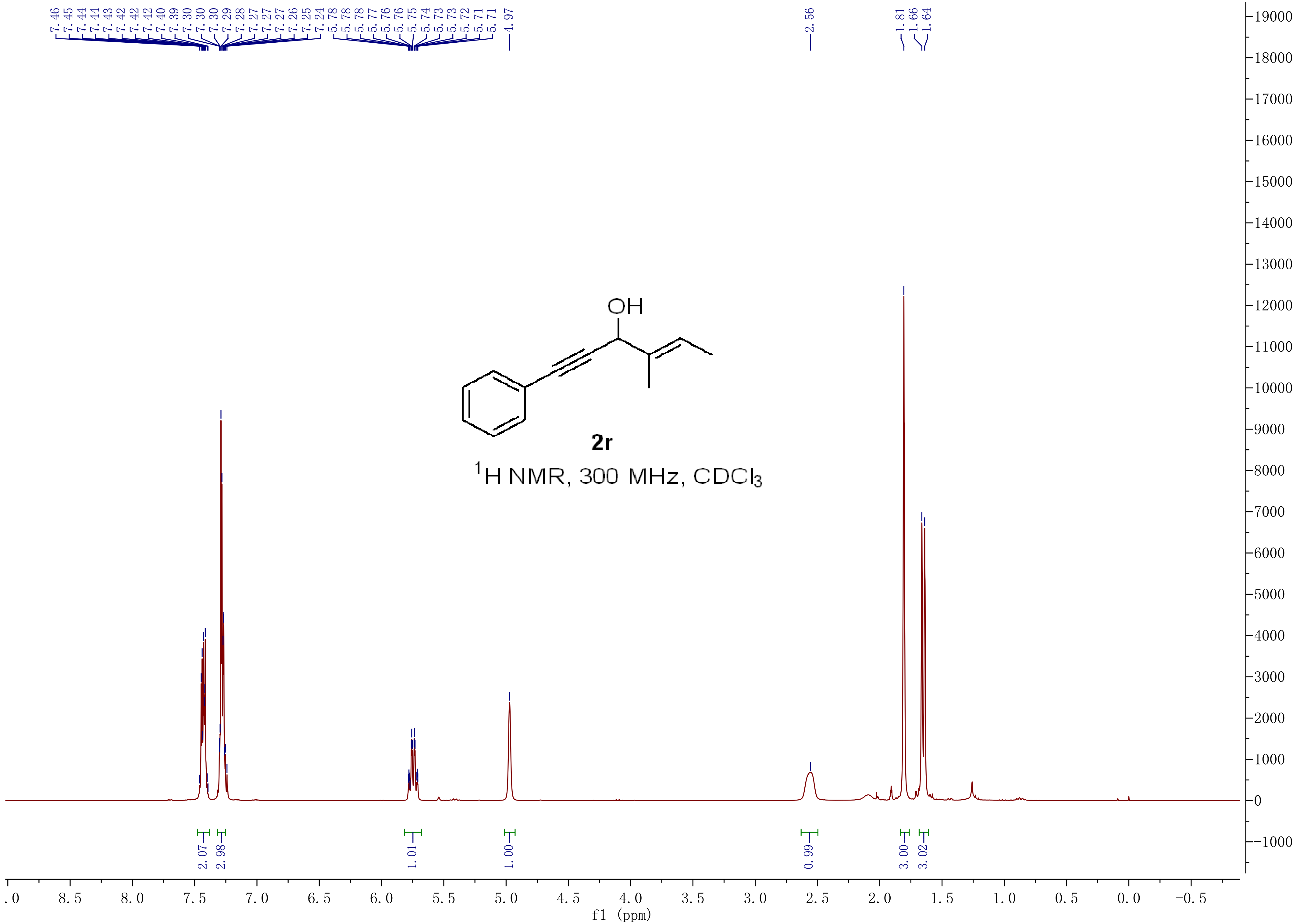

Supplementary Figure 27. ^{1}H NMR of compound 2r.

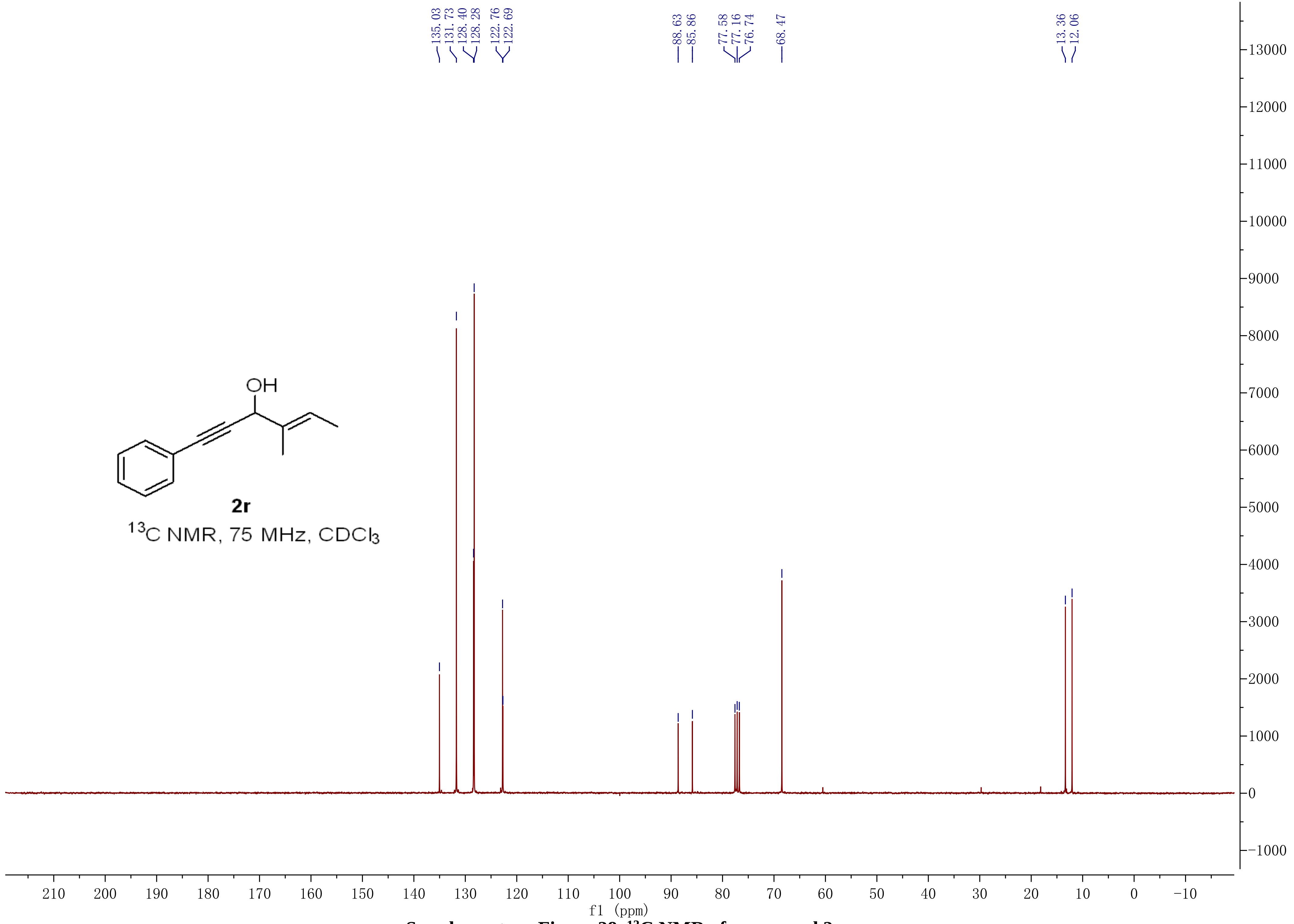

Supplementary Figure 28. ^{13}C NMR of compound 2r.

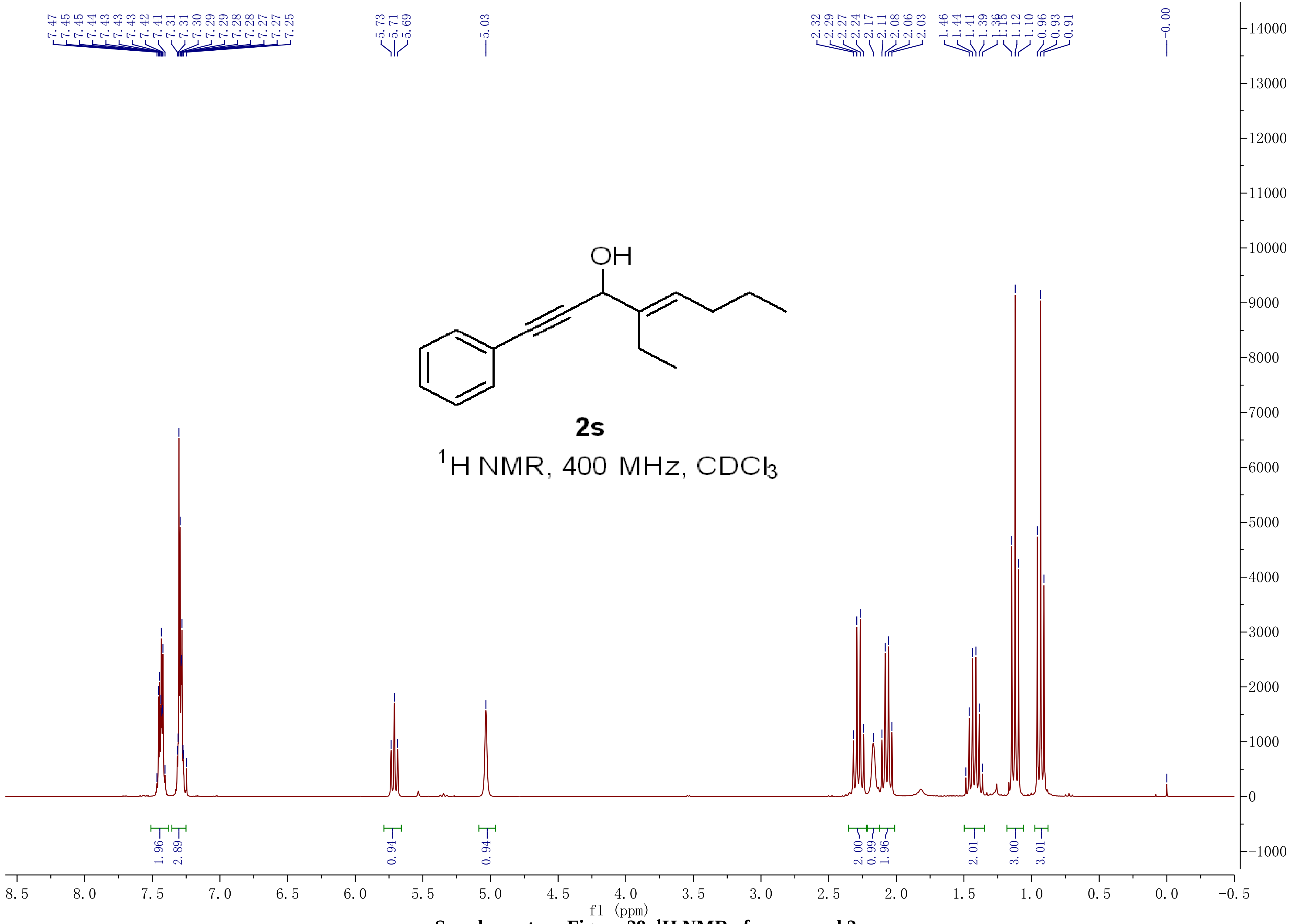

Supplementary Figure 29. ^{1}H NMR of compound 2s.

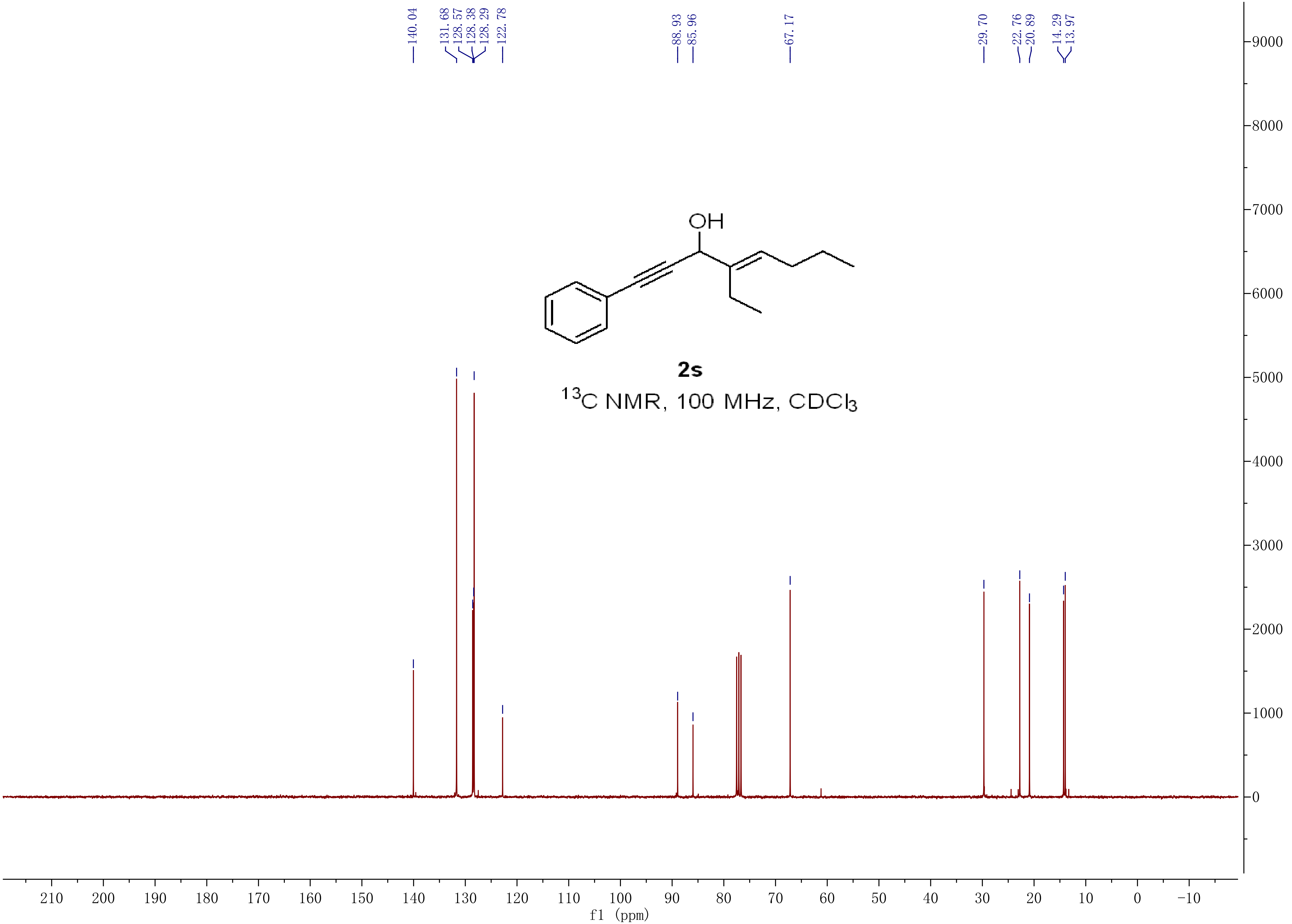

Supplementary Figure 30. ^{13}C NMR of compound 2s.

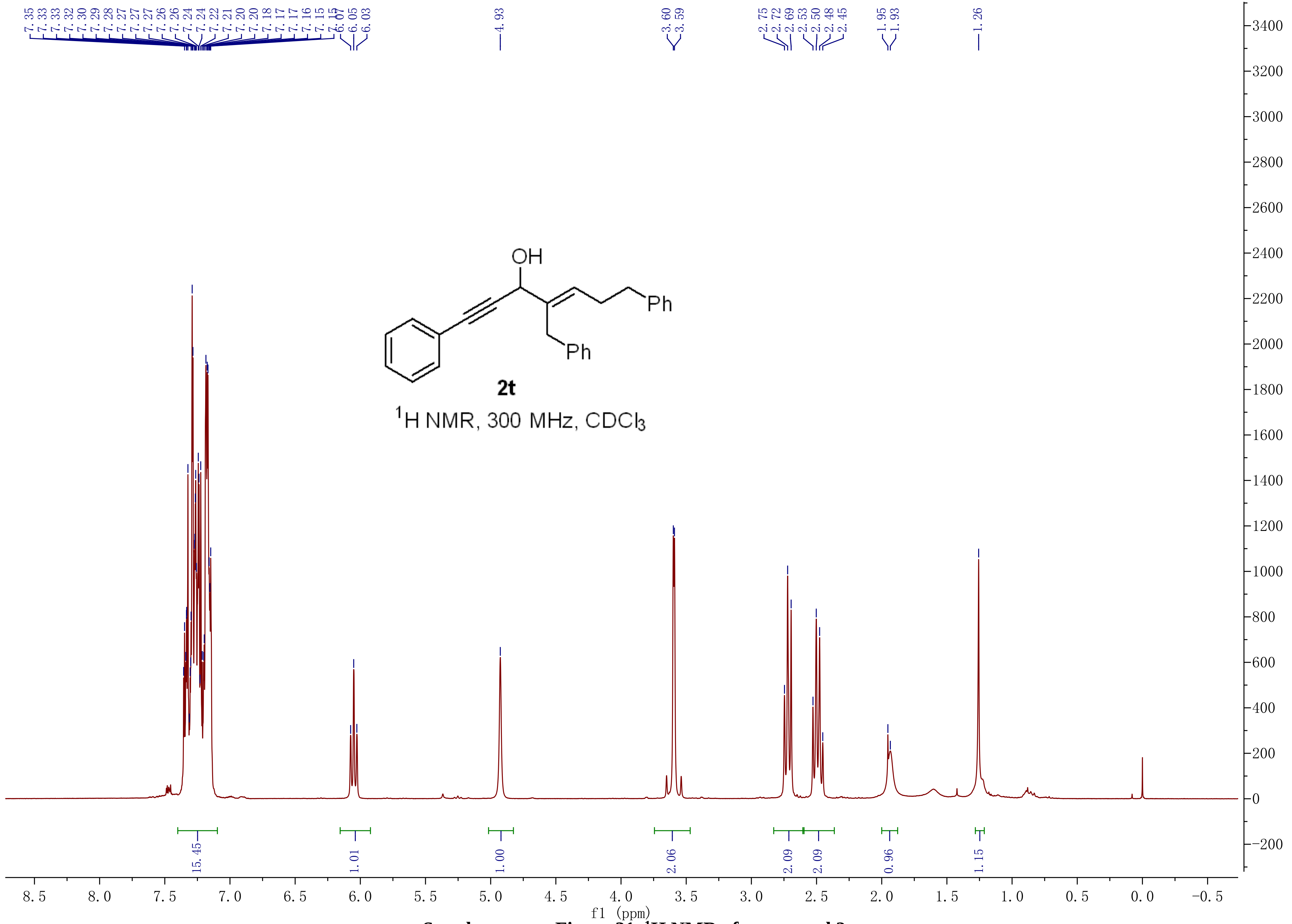

Supplementary Figure 31. ^{1}H NMR of compound 2t.

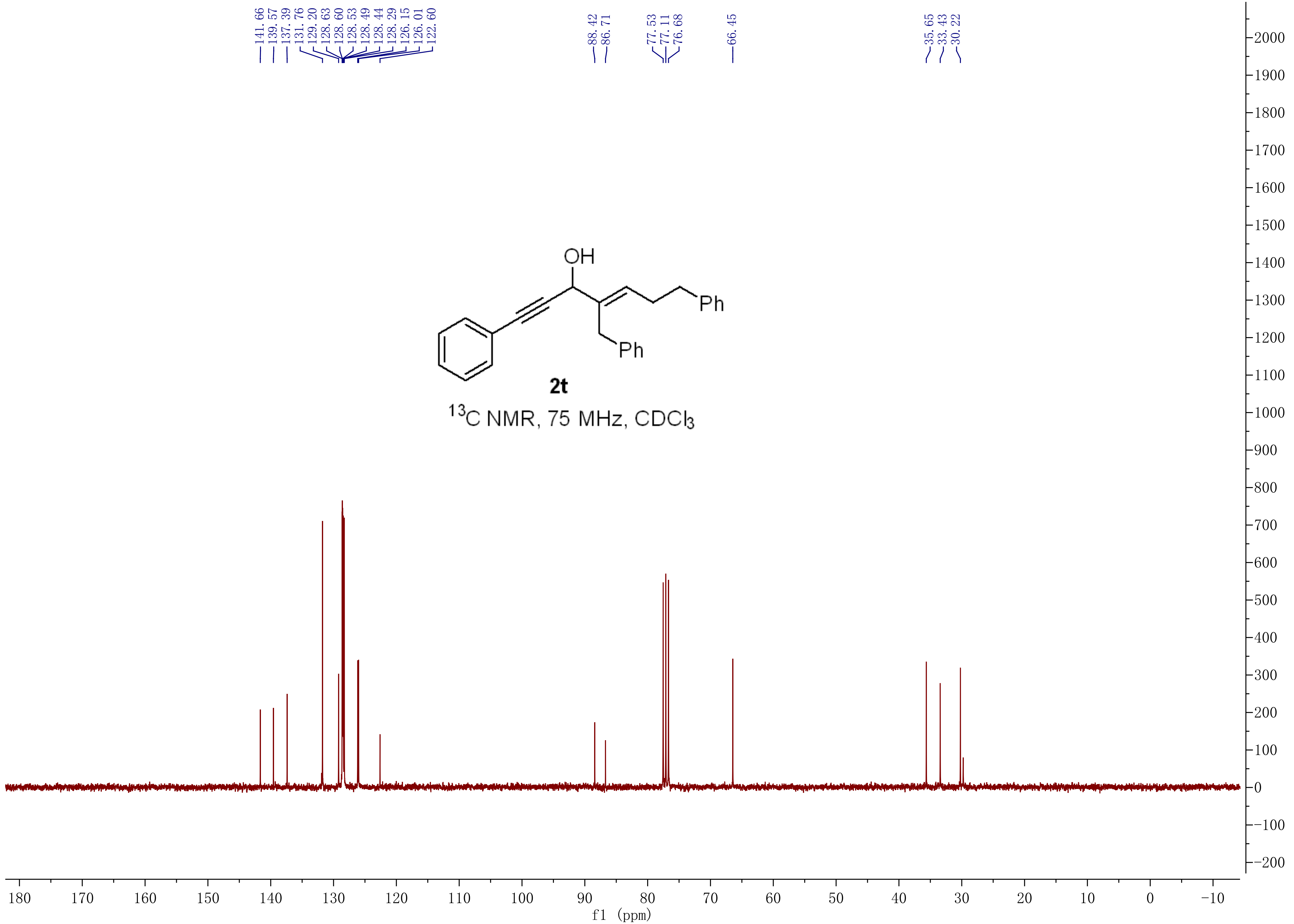

Supplementary Figure 32. ^{13}C NMR of compound 2t.

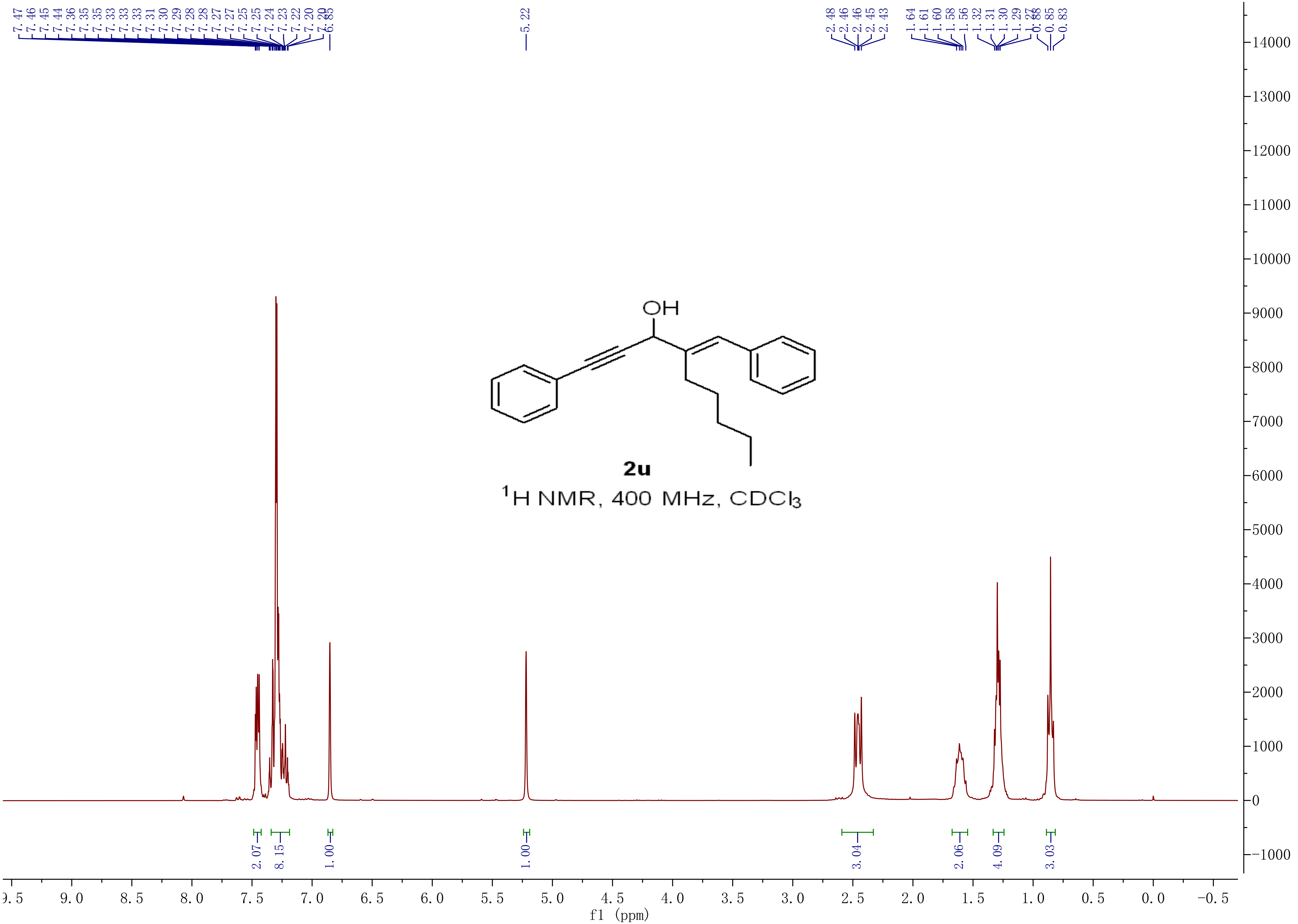

Supplementary Figure 33. ^{1}H NMR of compound 2u.

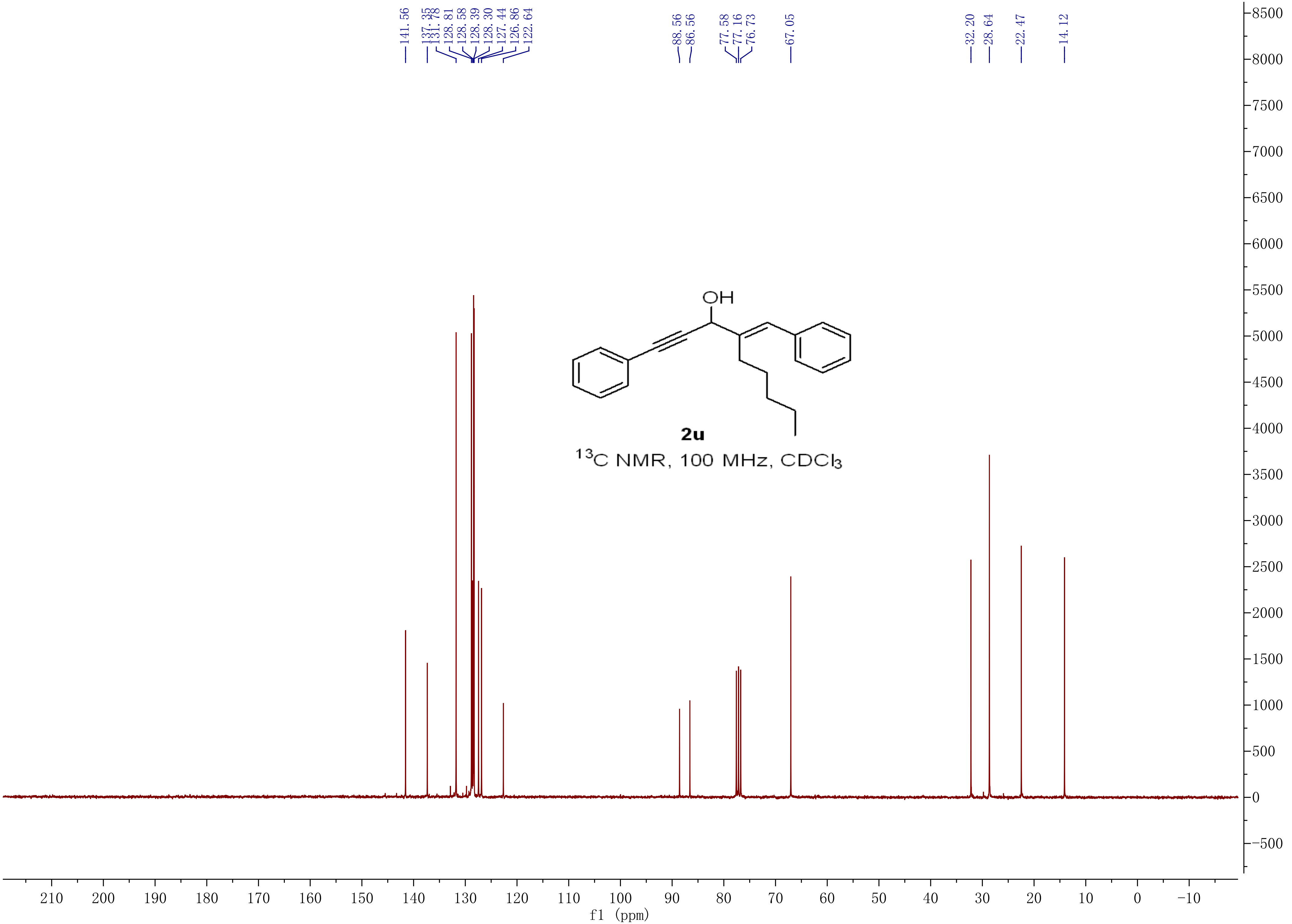

Supplementary Figure 34. ^{13}C NMR of compound 2u.

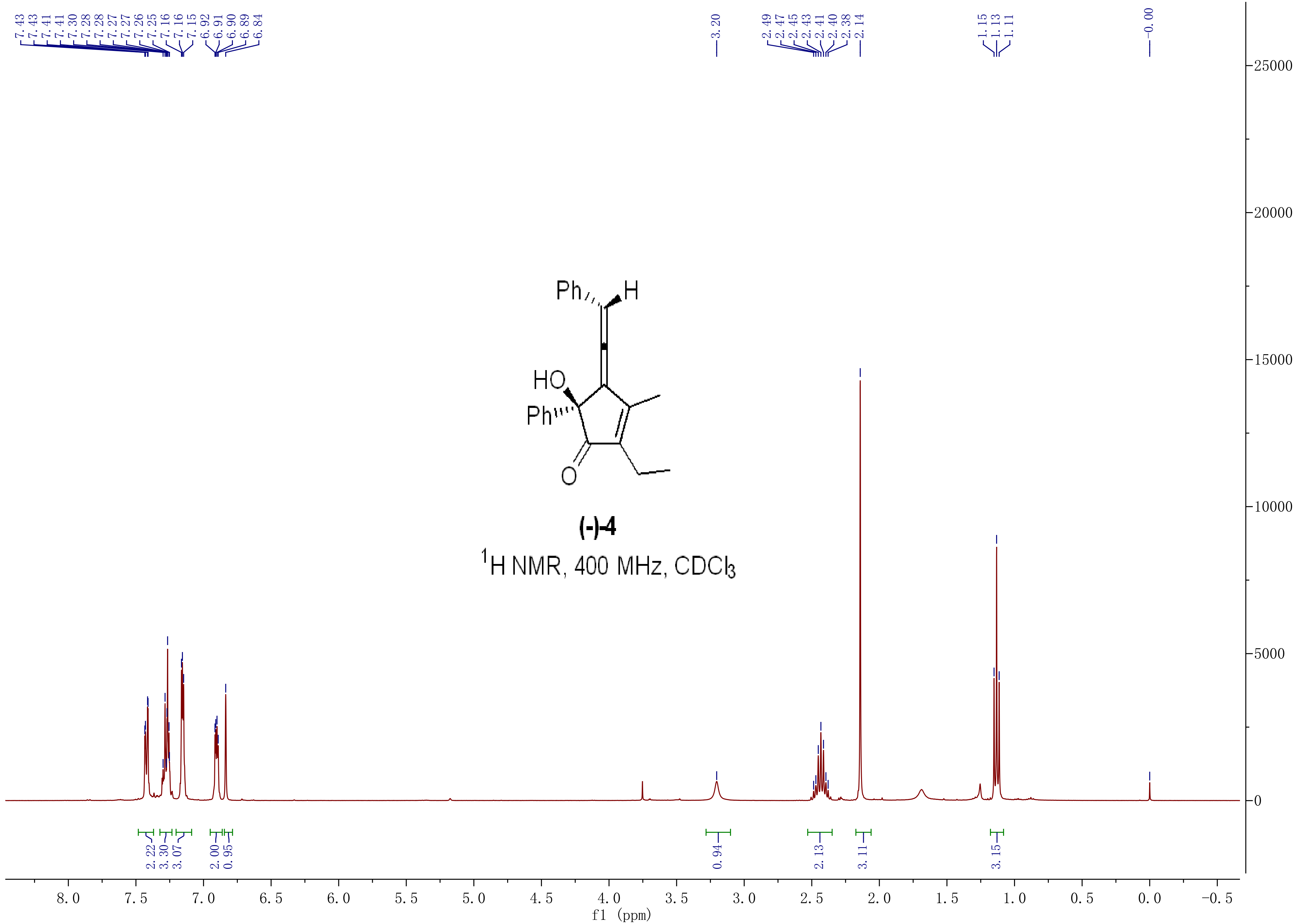

Supplementary Figure 35. ^{1}H NMR of compound (-)-4.

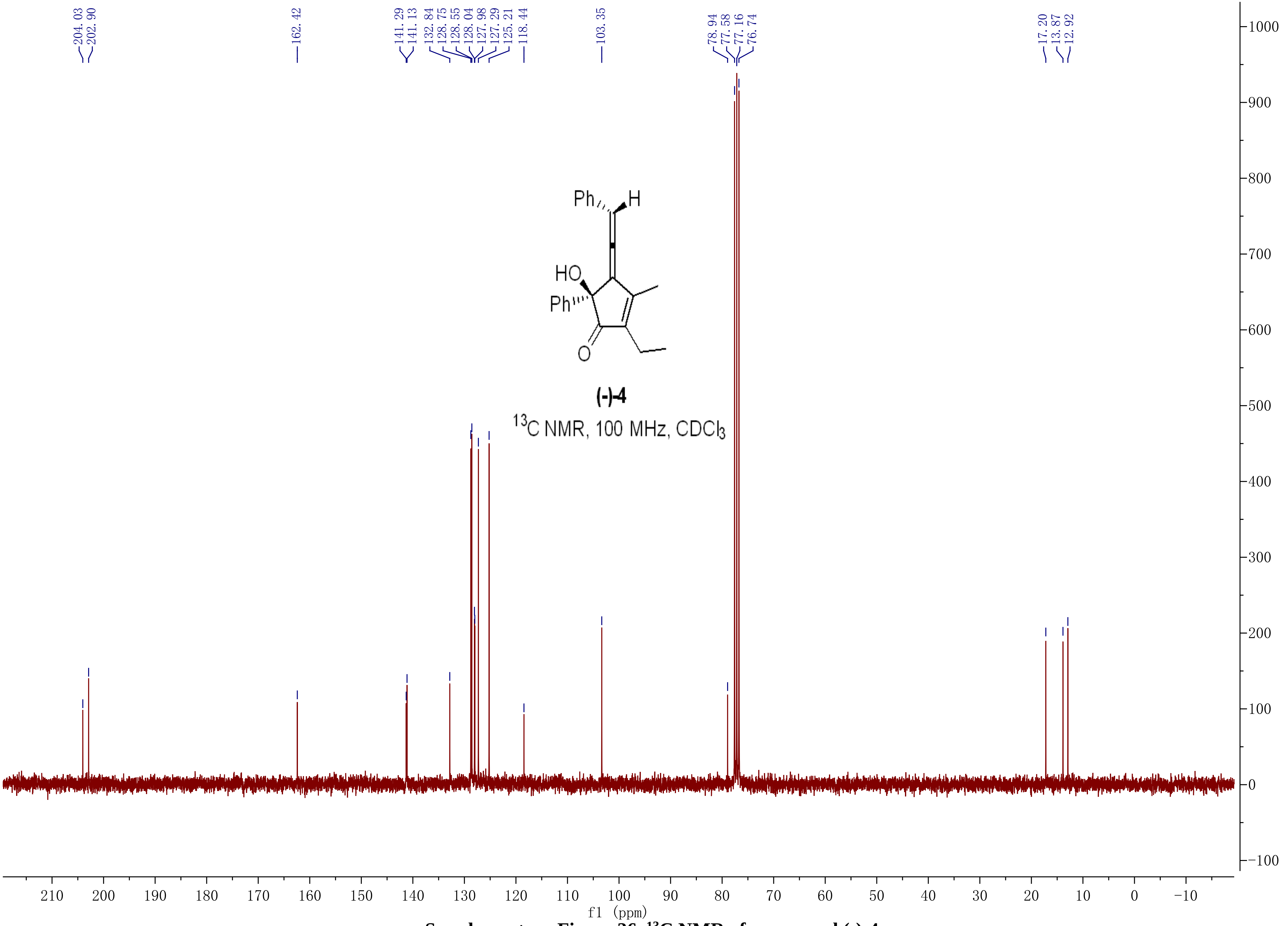

Supplementary Figure 36. ^{13}C NMR of compound (-)-4.

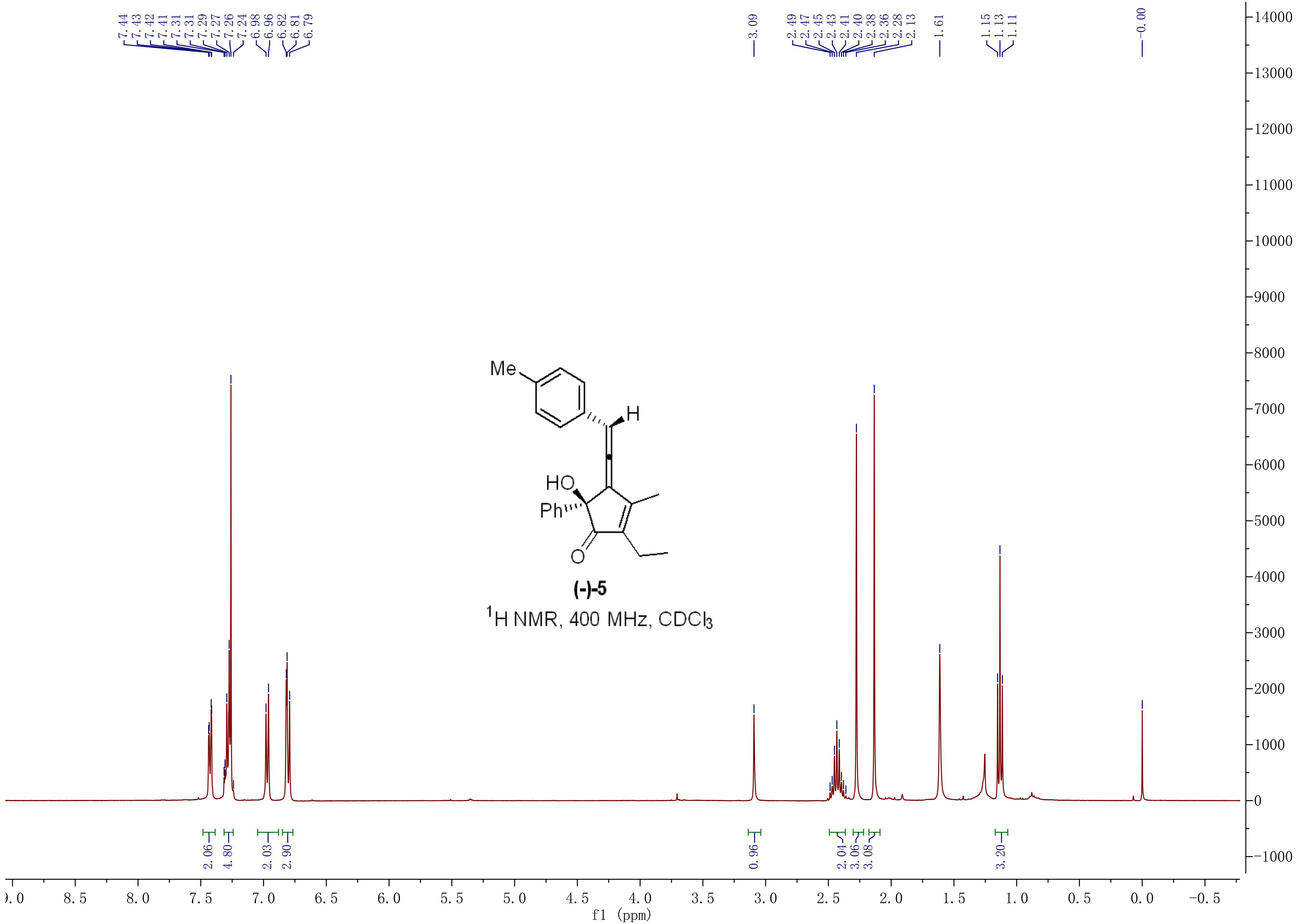

Supplementary Figure 37. ^{1}H NMR of compound (-)-5.

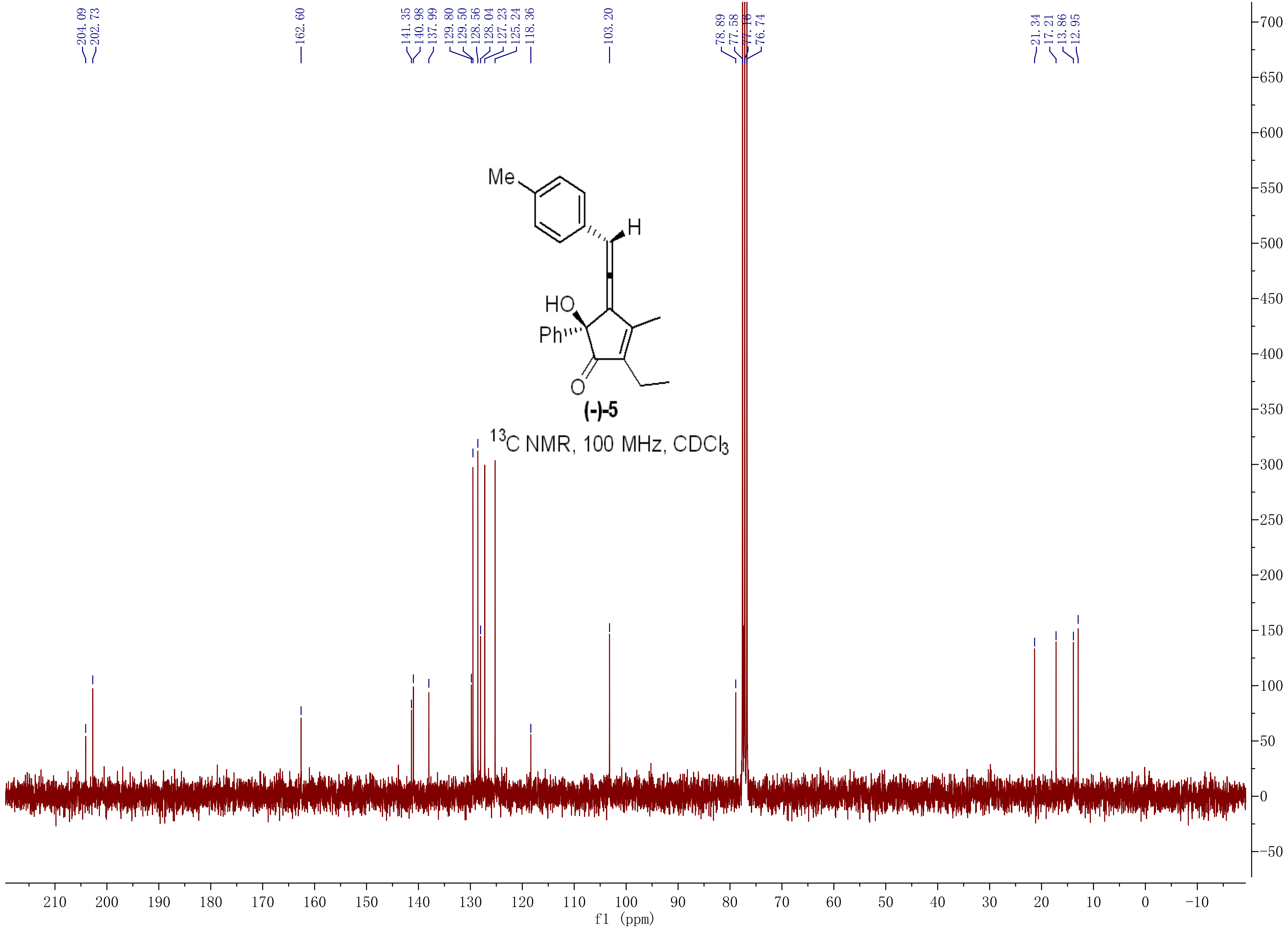

Supplementary Figure 38. ^{13}C NMR of compound (-)-5.

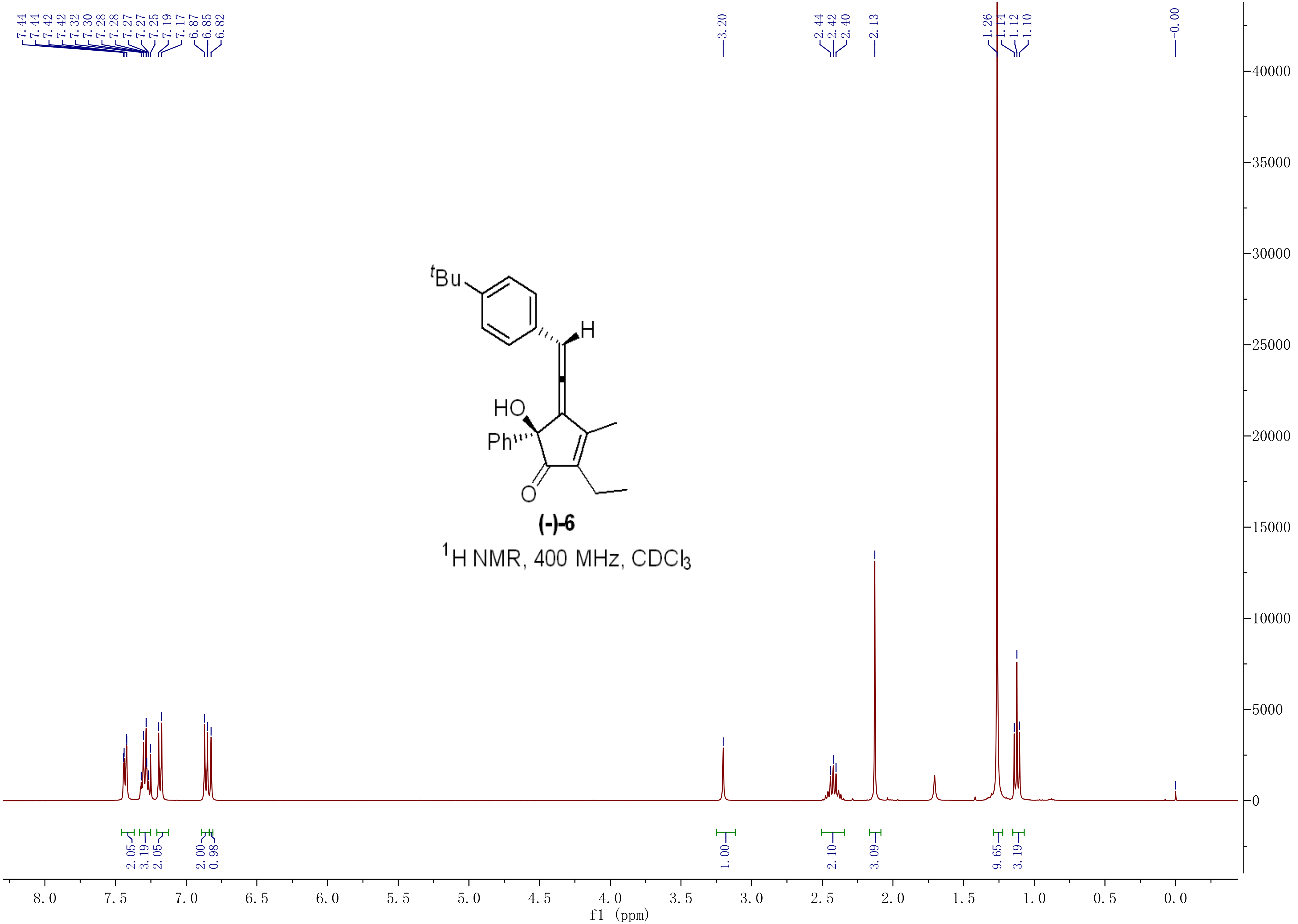

Supplementary Figure 39. ^{1}**H NMR of compound (-)-6.**

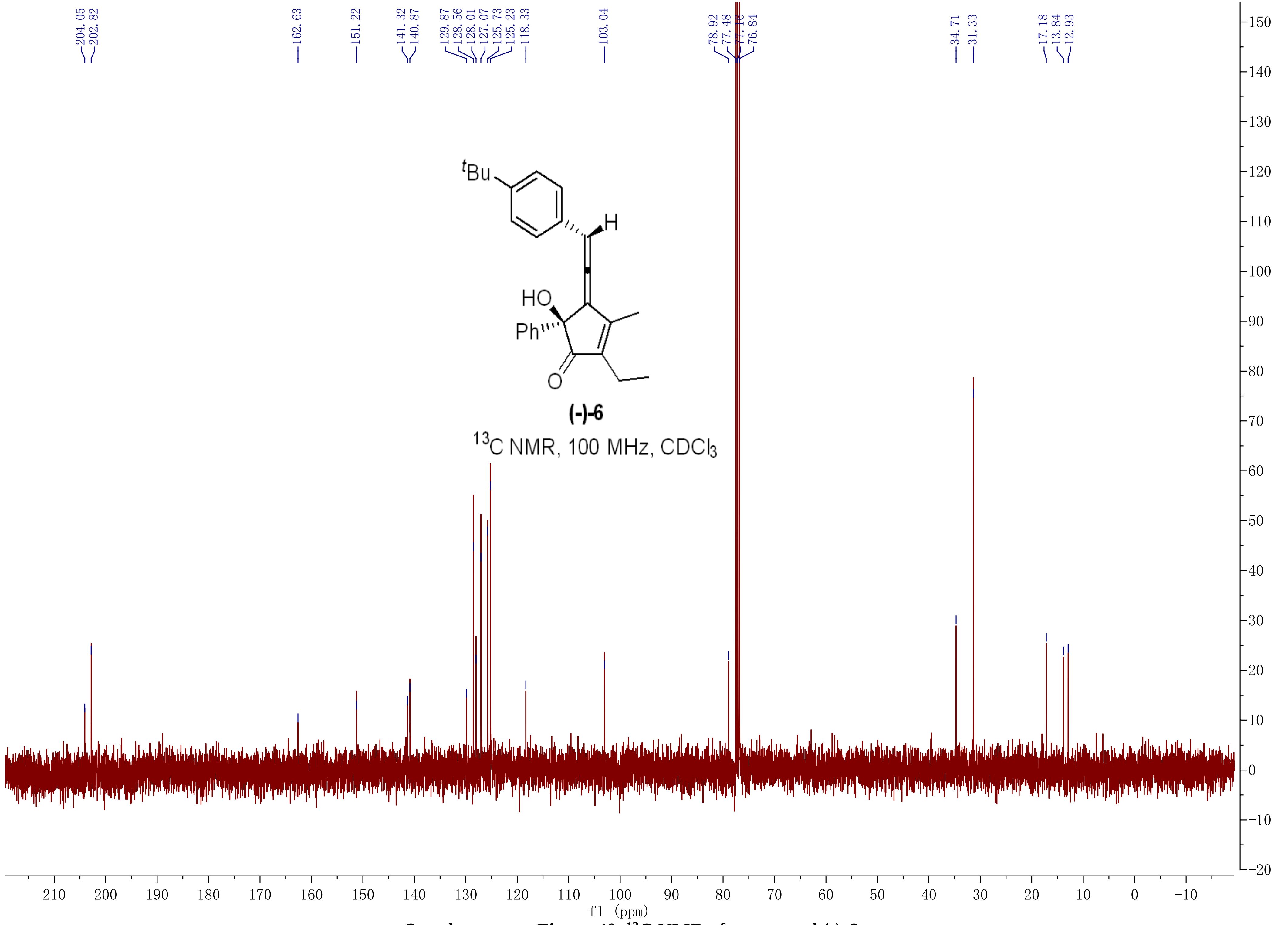

Supplementary Figure 40. ^{13}C NMR of compound (-)-6.

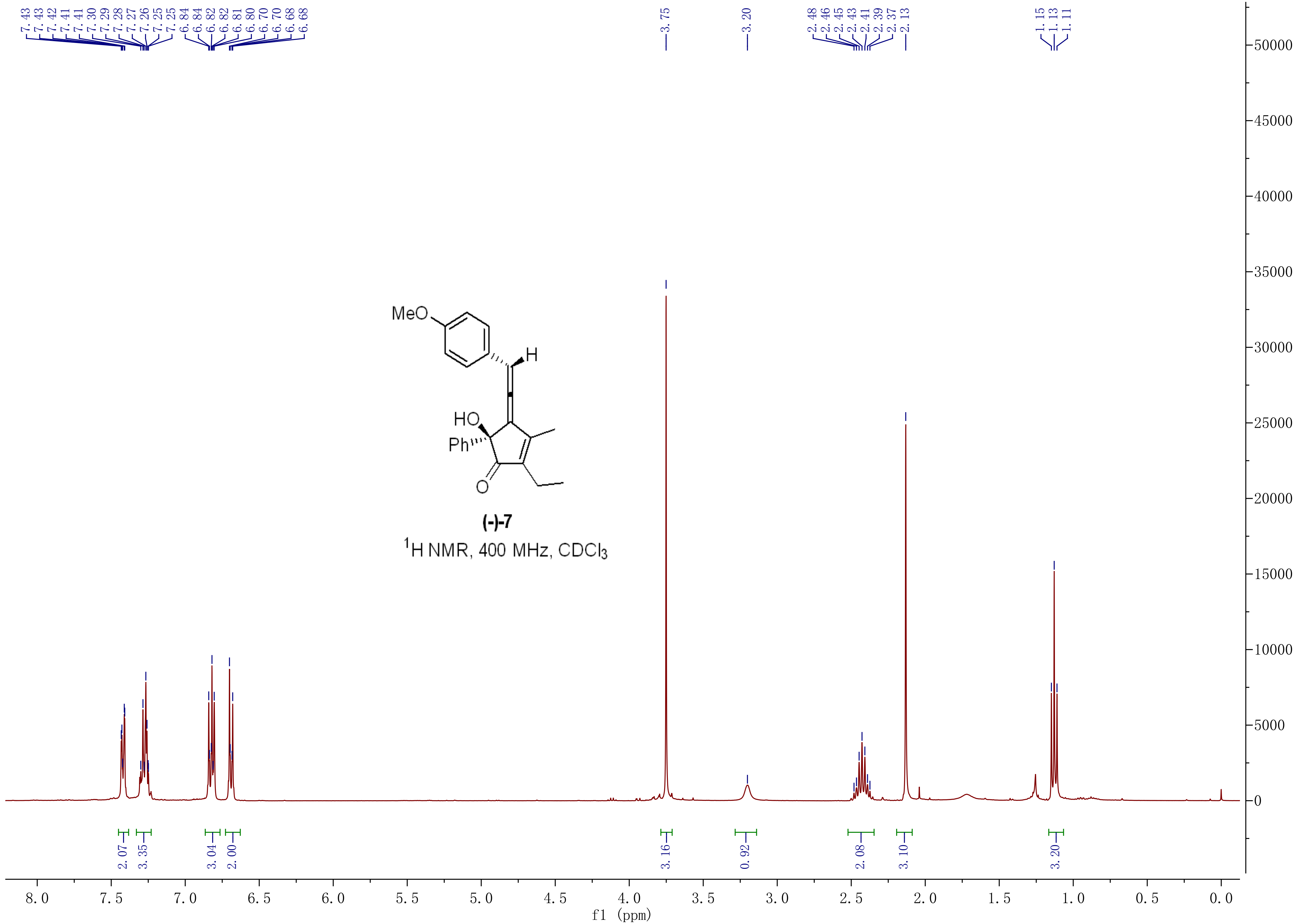

Supplementary Figure 41. ^{1}H NMR of compound (-)-7.

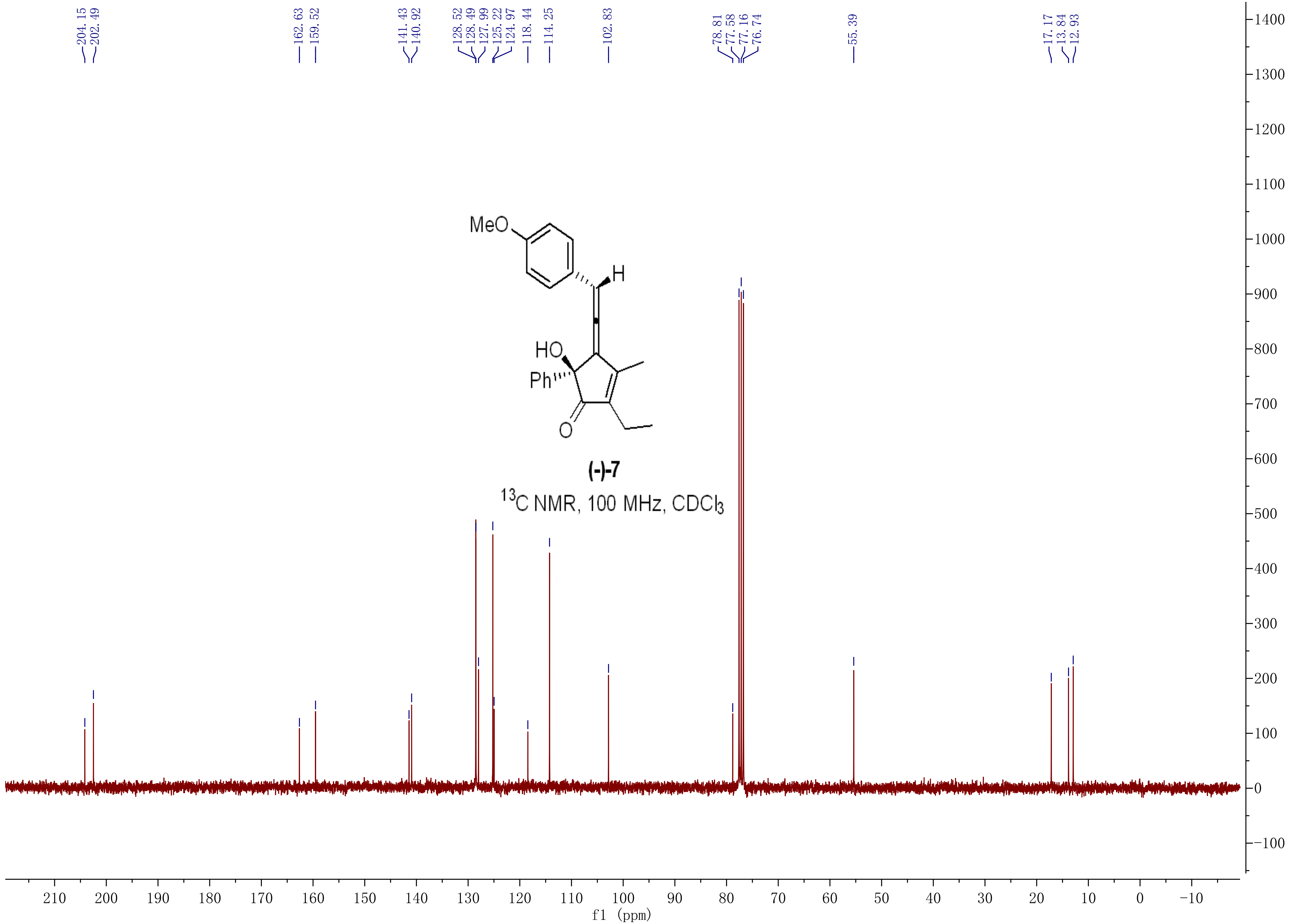

Supplementary Figure 42. ^{13}C NMR of compound (-)-7.

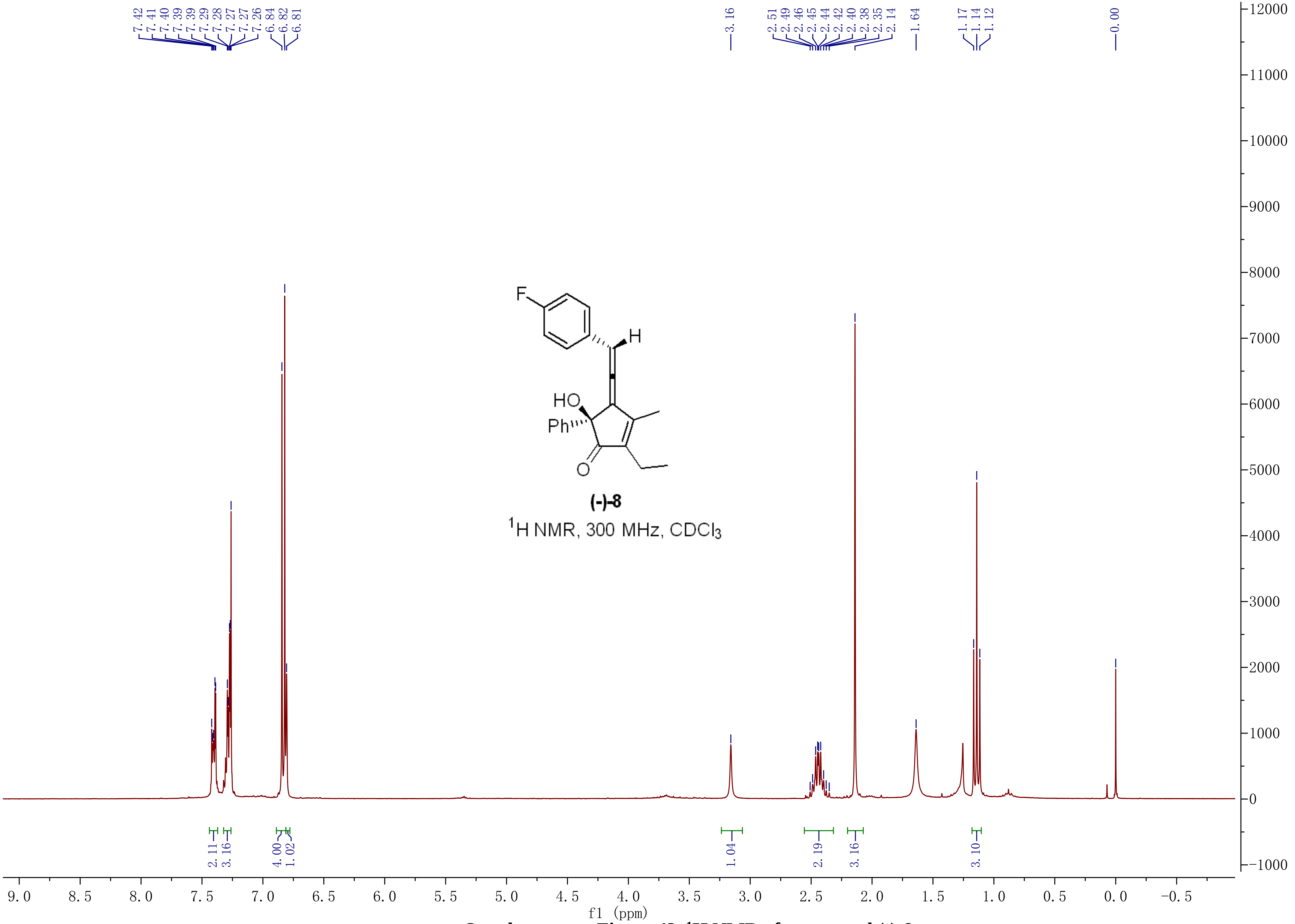

Supplementary Figure 43. ¹H NMR of compound (-)-8.

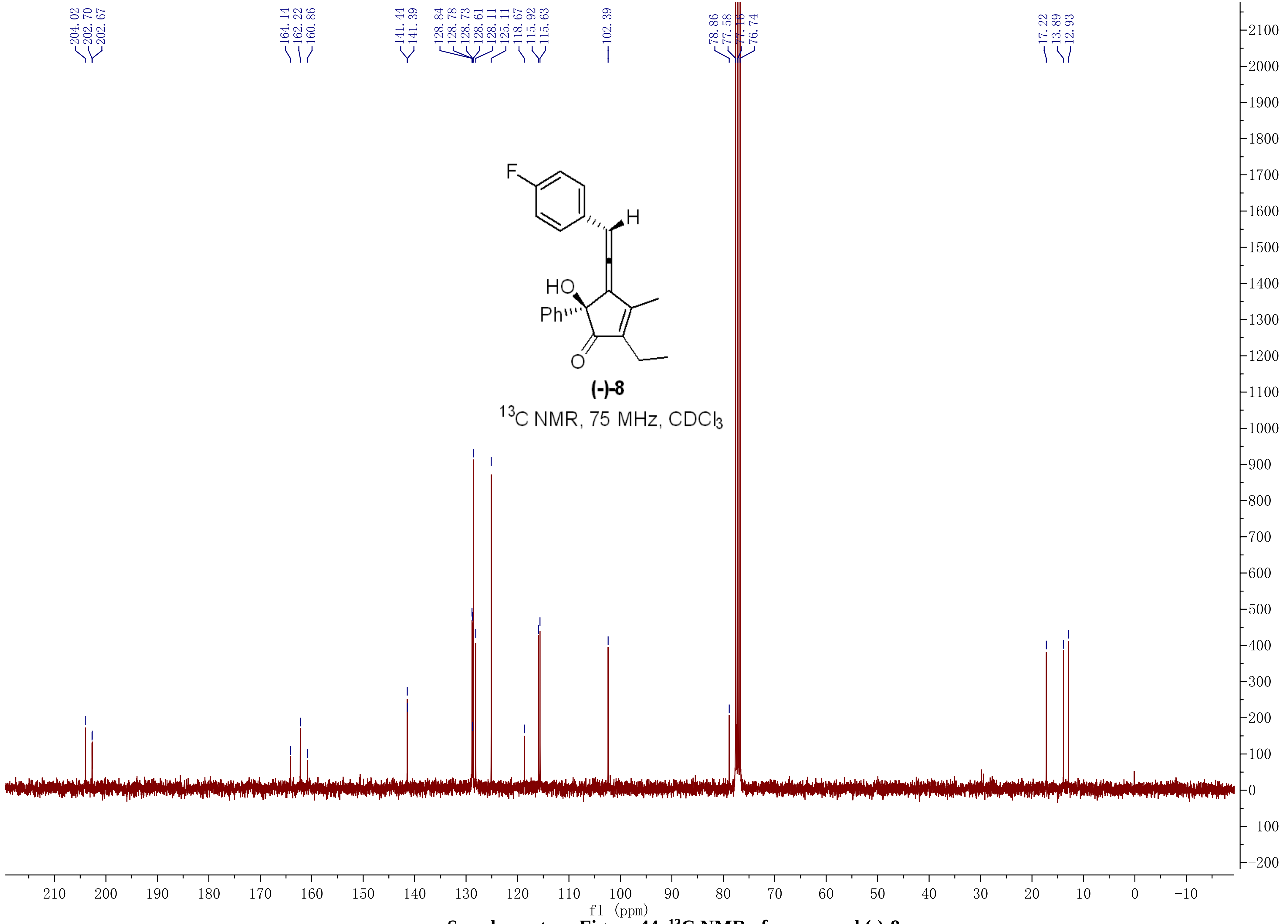

Supplementary Figure 44. ^{13}C NMR of compound (-)-8.

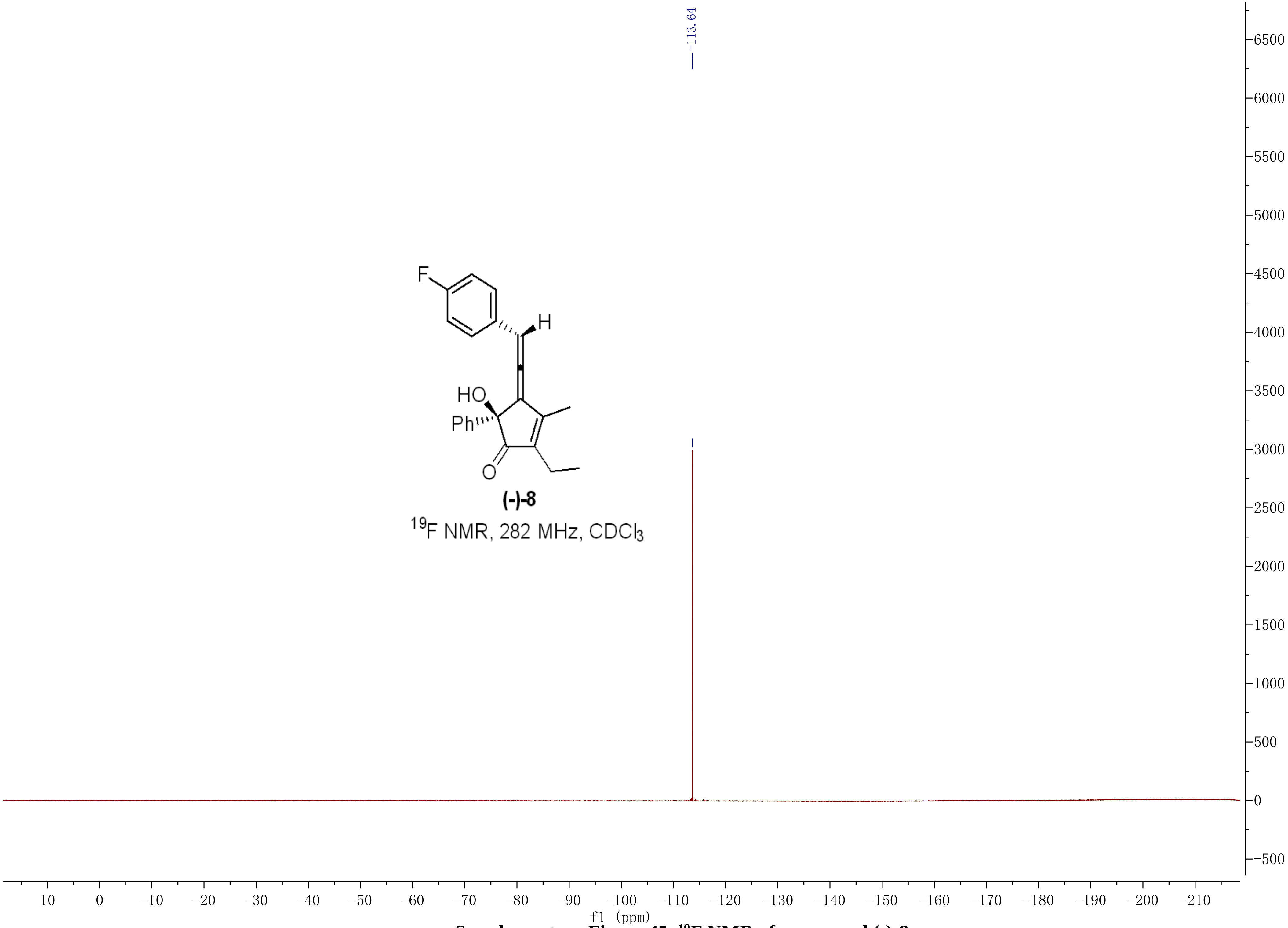

Supplementary Figure 45. ^{19}F NMR of compound (-)-8.

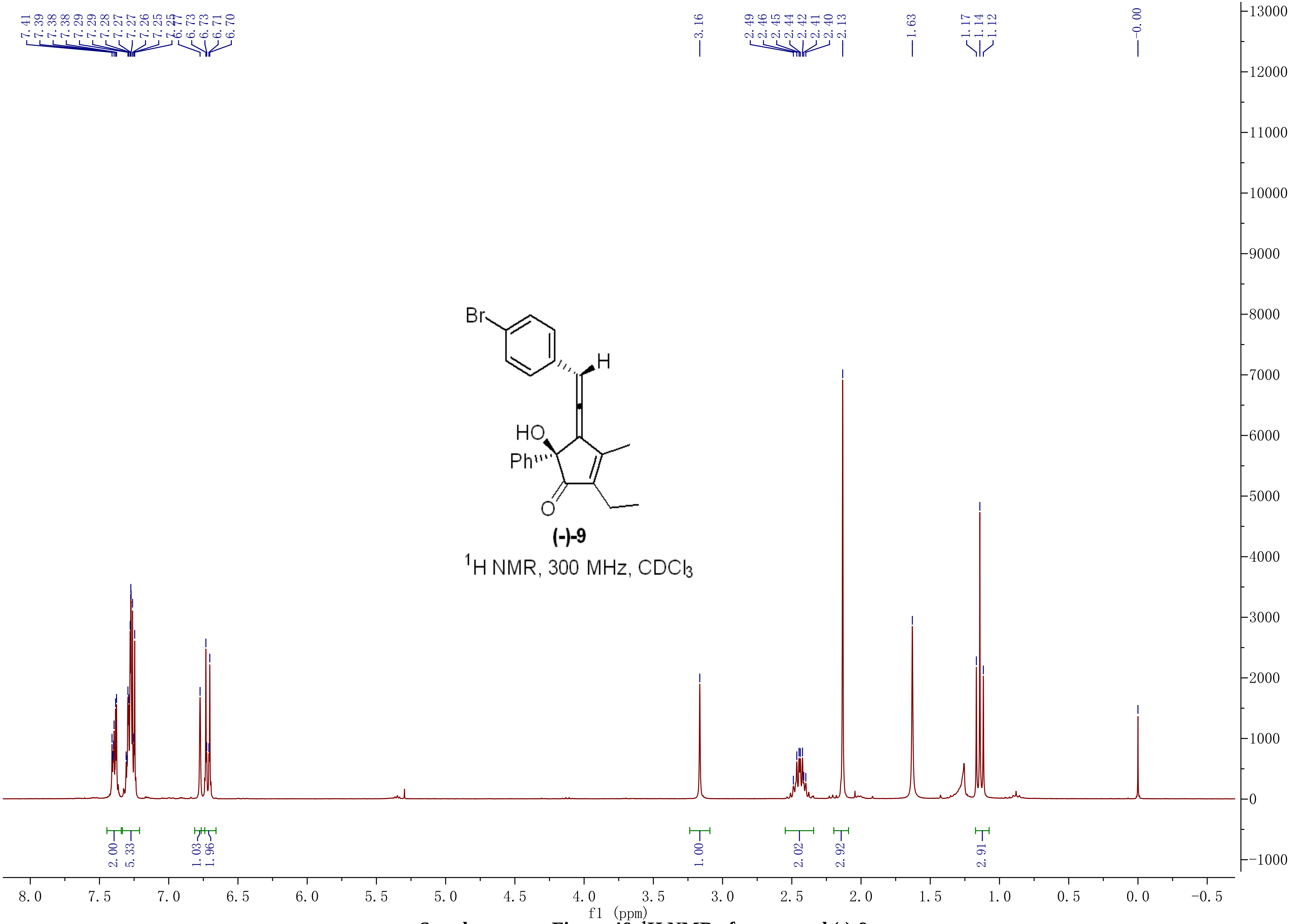

Supplementary Figure 46. ^{1}H NMR of compound (-)-9.

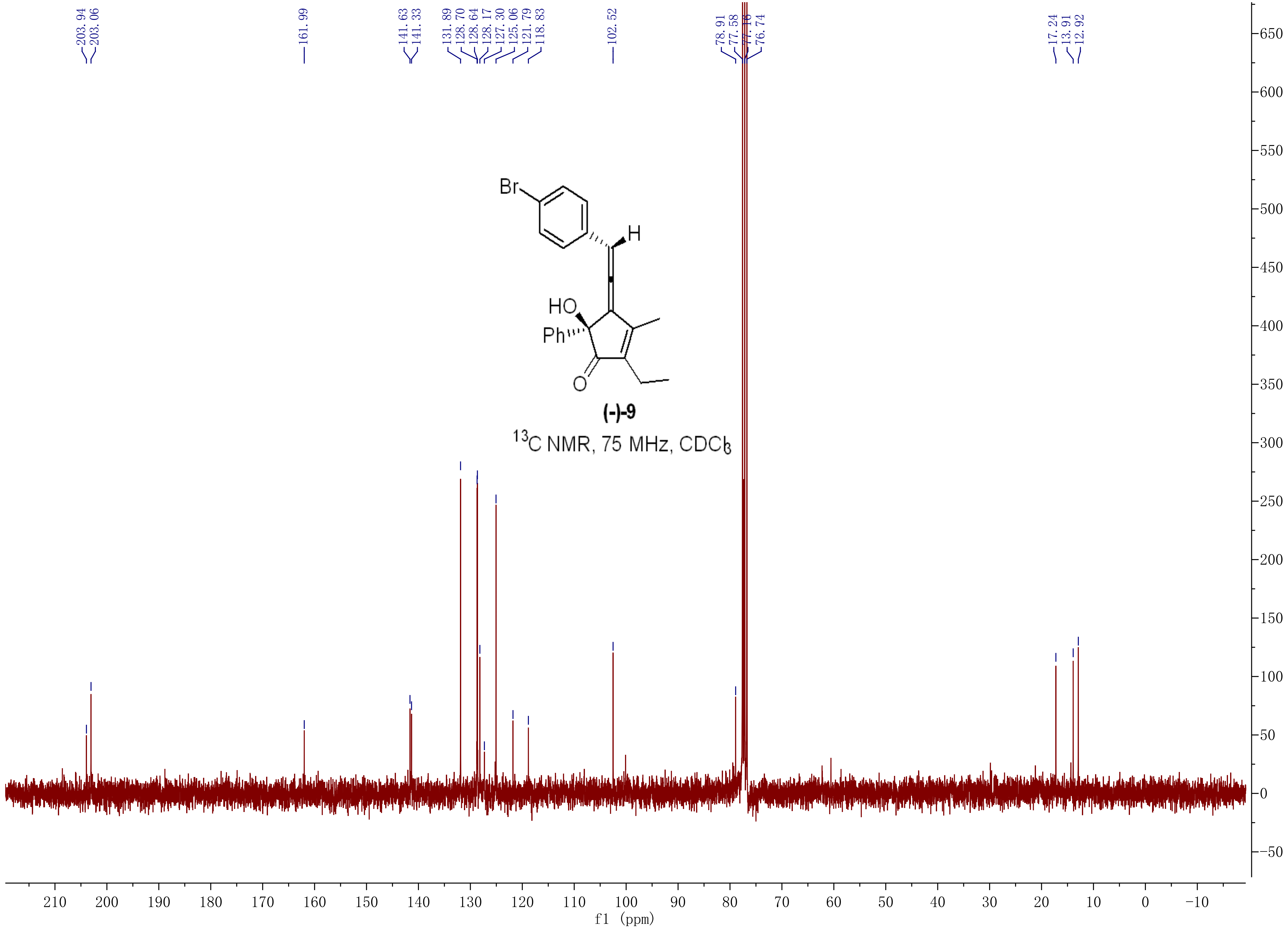

Supplementary Figure 47. ^{13}**C NMR of compound (-)-9.**

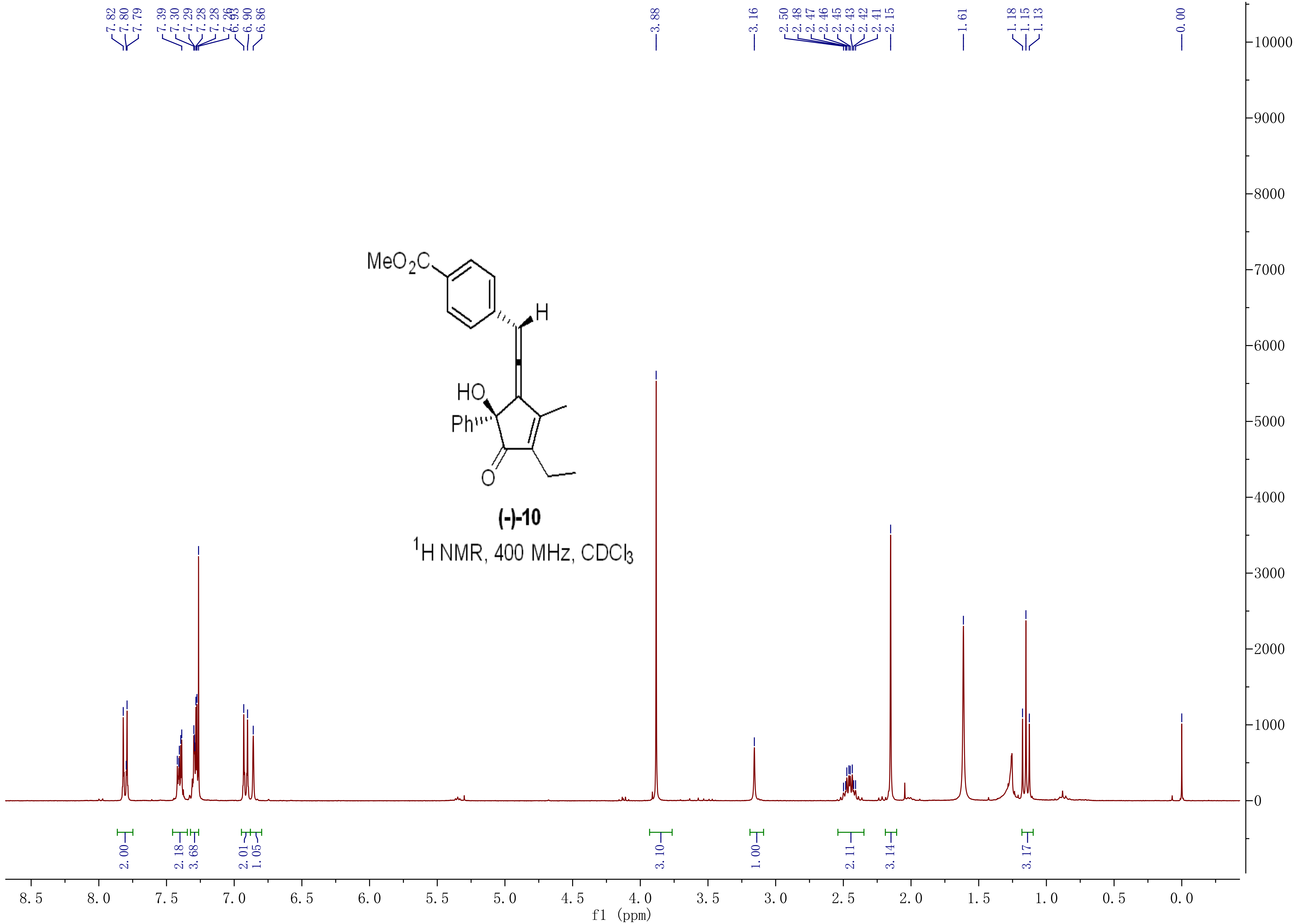

Supplementary Figure 48. ^{1}H NMR of compound (-)-10.

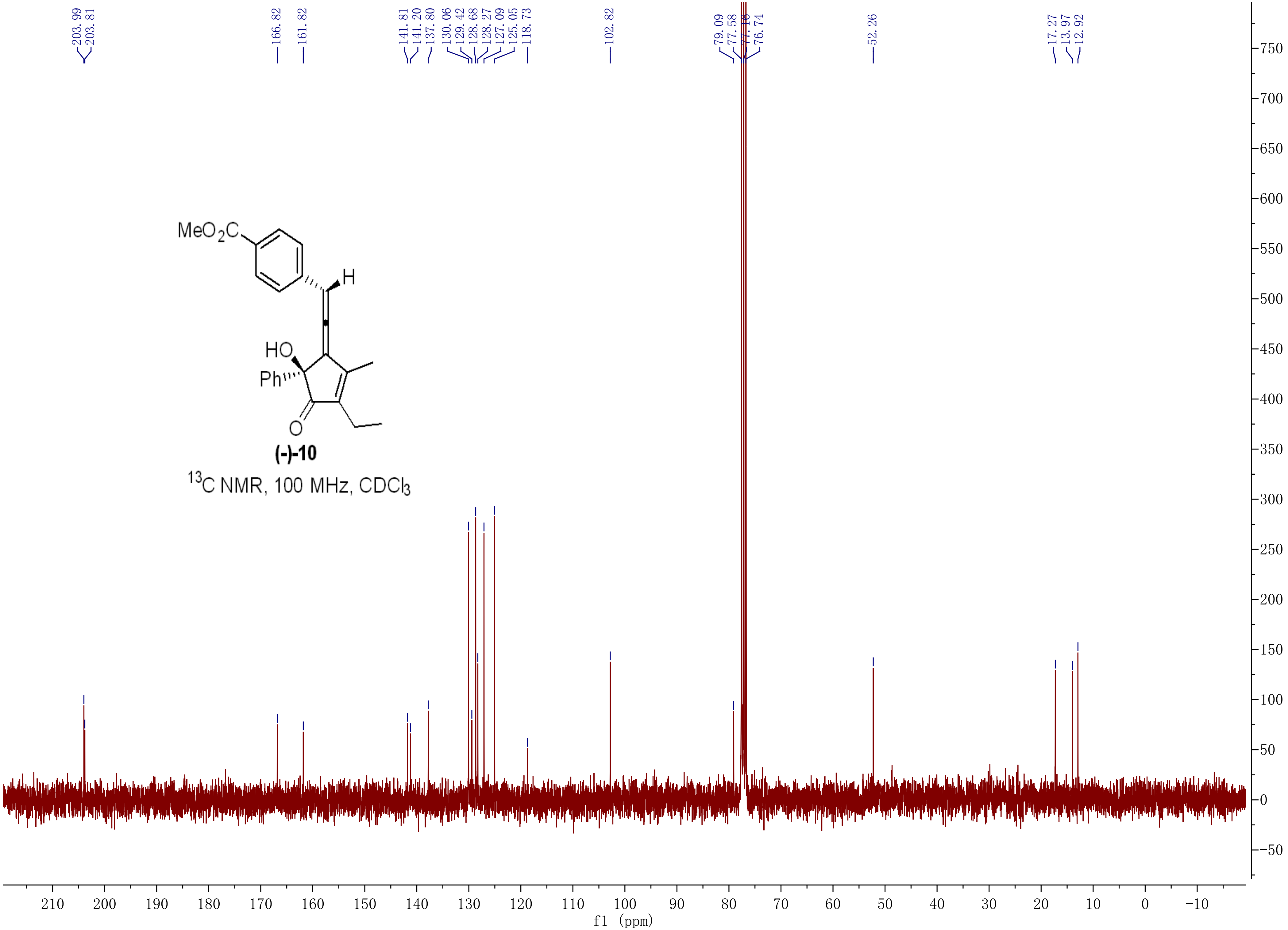

Supplementary Figure 49. ^{13}C NMR of compound (-)-10.

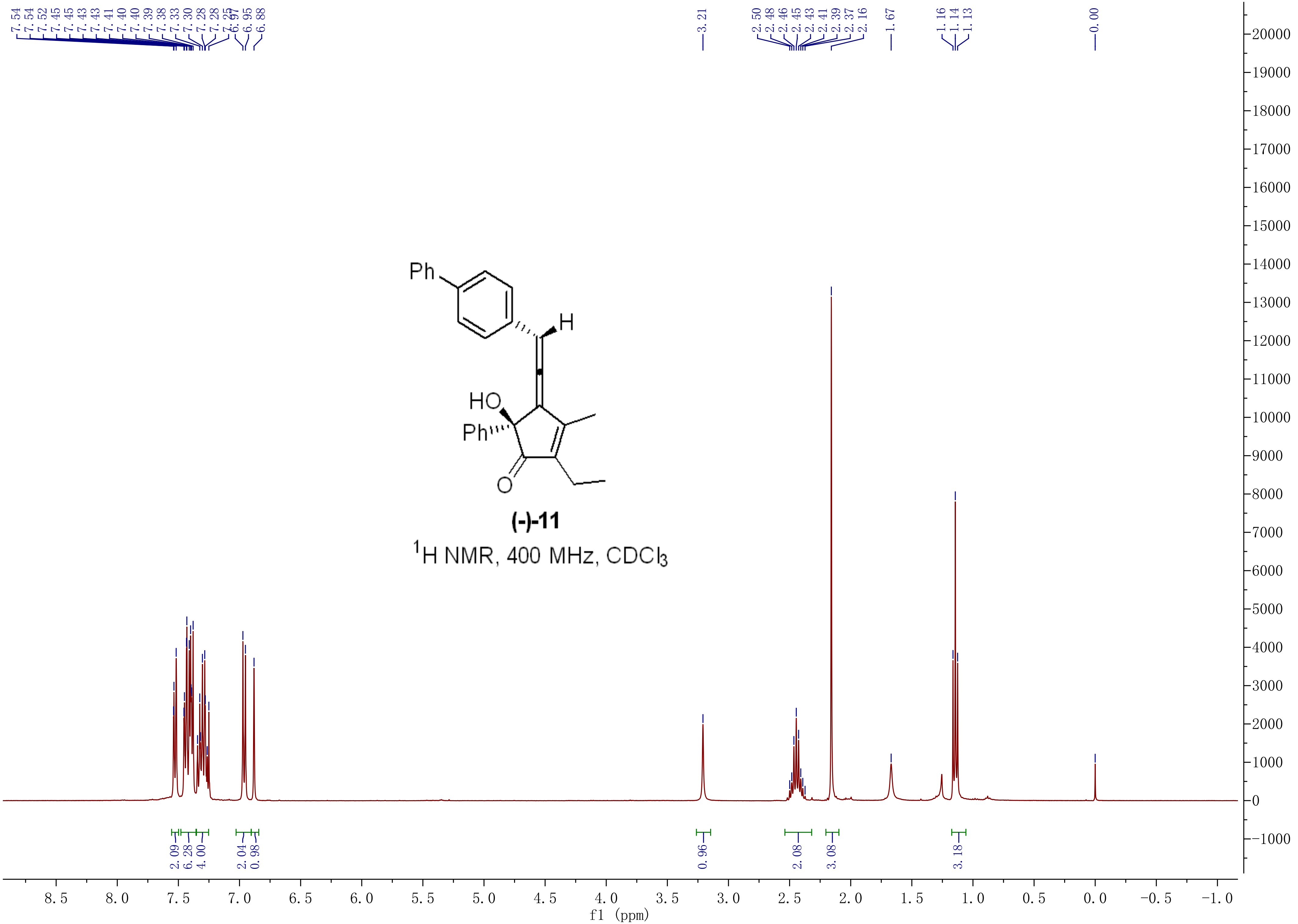

Supplementary Figure 50. ¹H NMR of compound (-)-11.

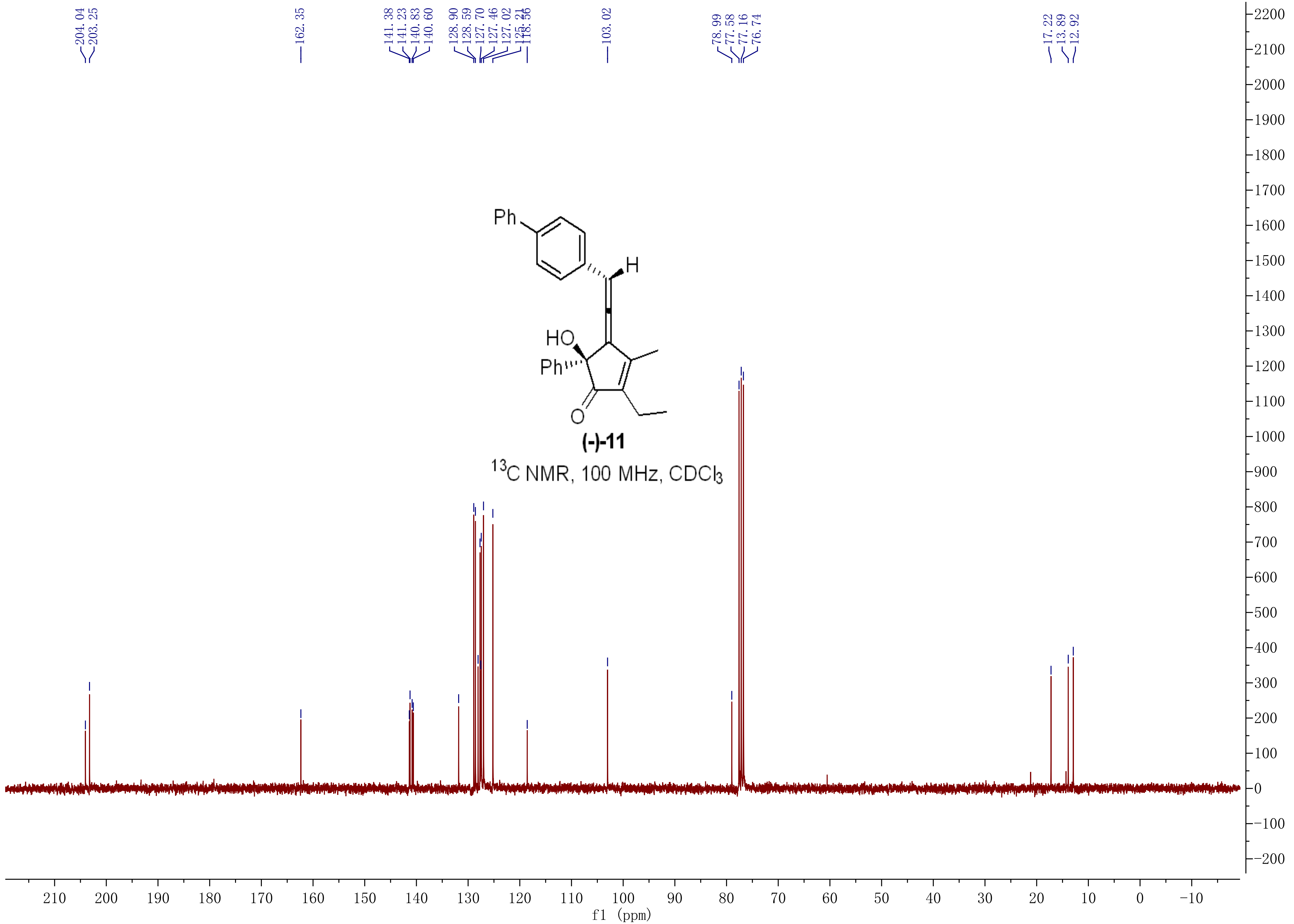

Supplementary Figure 51. ^{13}C NMR of compound (-)-11.

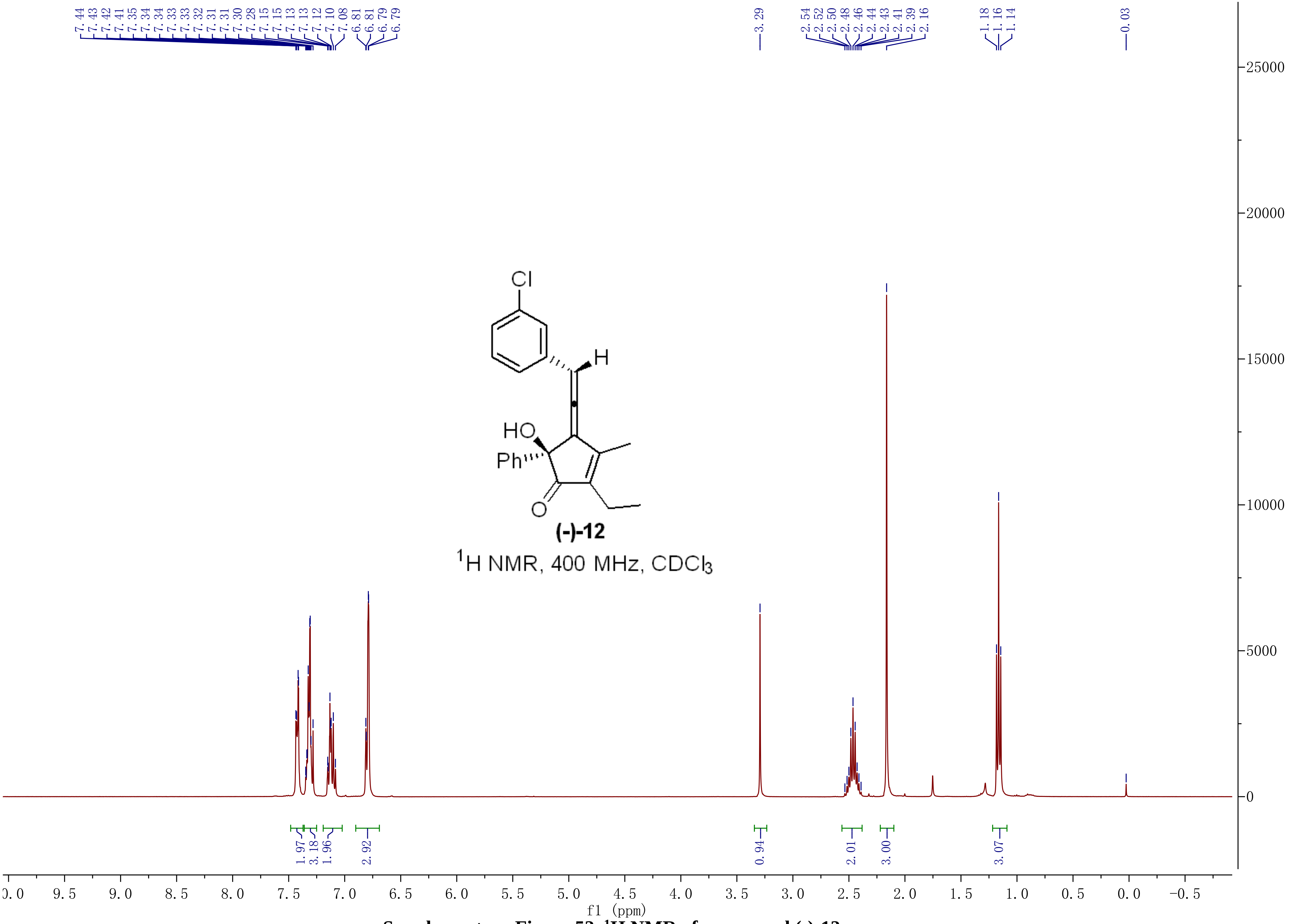

Supplementary Figure 52. ^{1}H NMR of compound (-)-12.

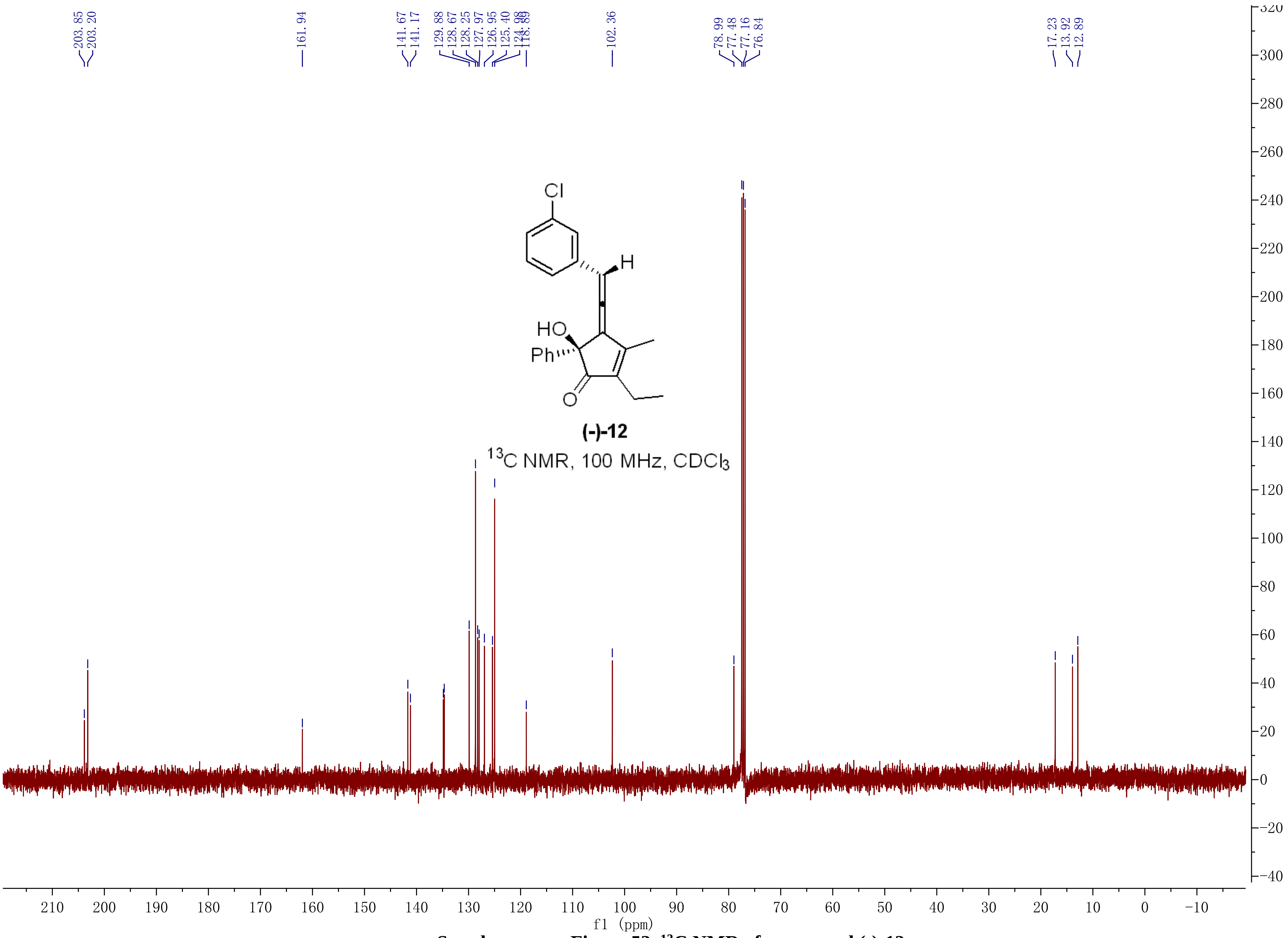

Supplementary Figure 53. ^{13}C NMR of compound (-)-12.

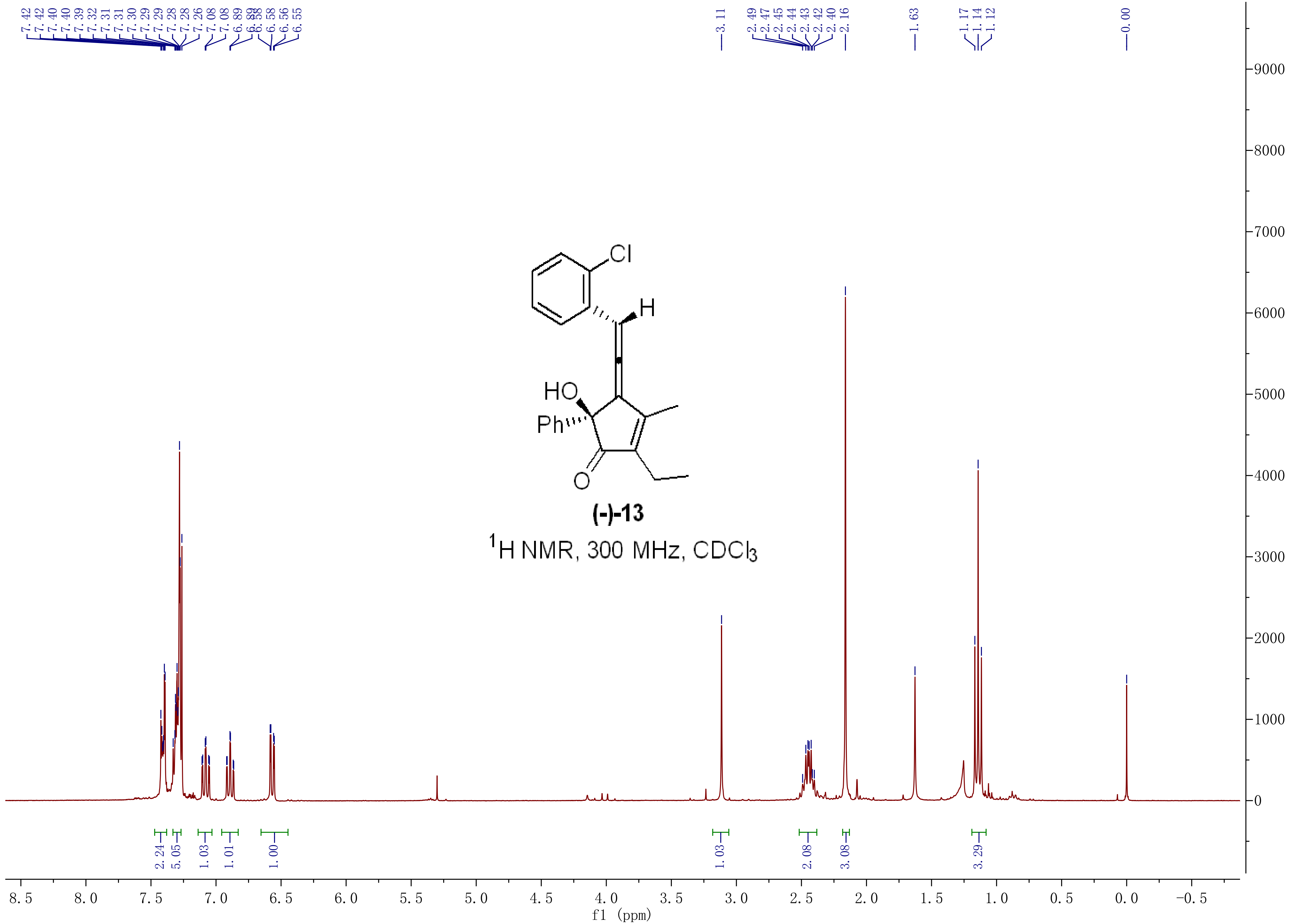

Supplementary Figure 54. ^{1}H NMR of compound (-)-13.

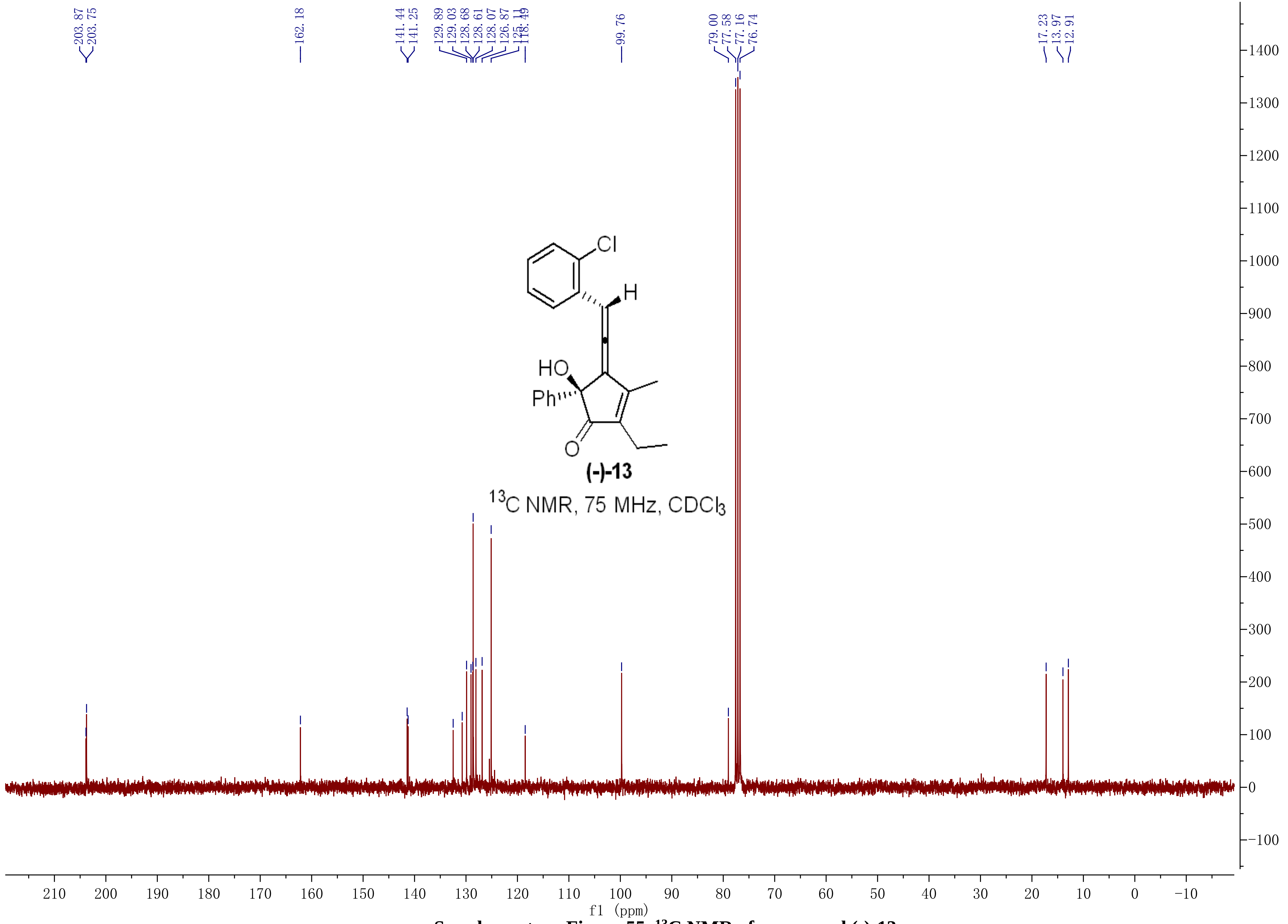

Supplementary Figure 55. ^{13}C NMR of compound (-)-13.

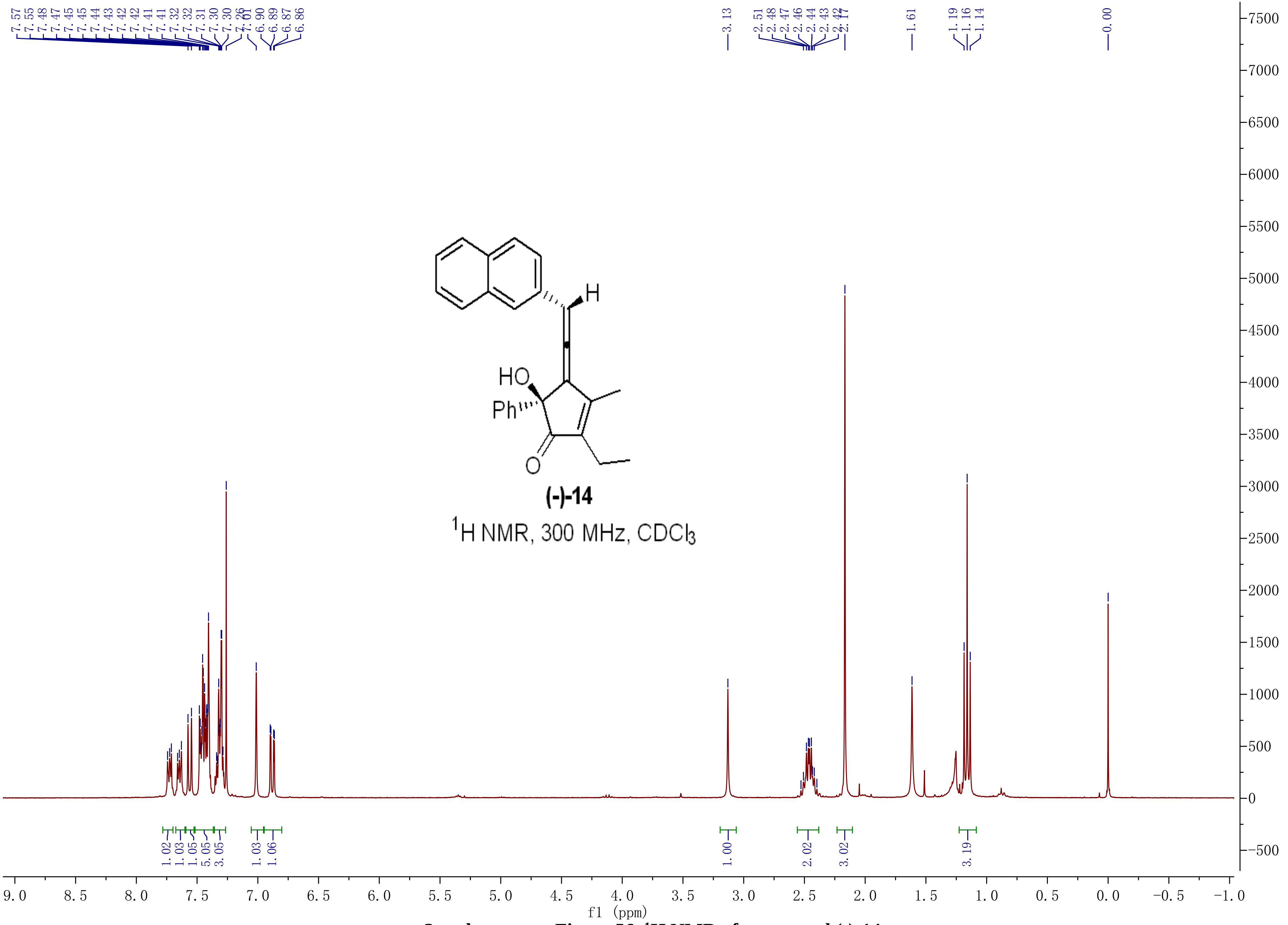

Supplementary Figure 56. ^{1}H NMR of compound (-)-14.

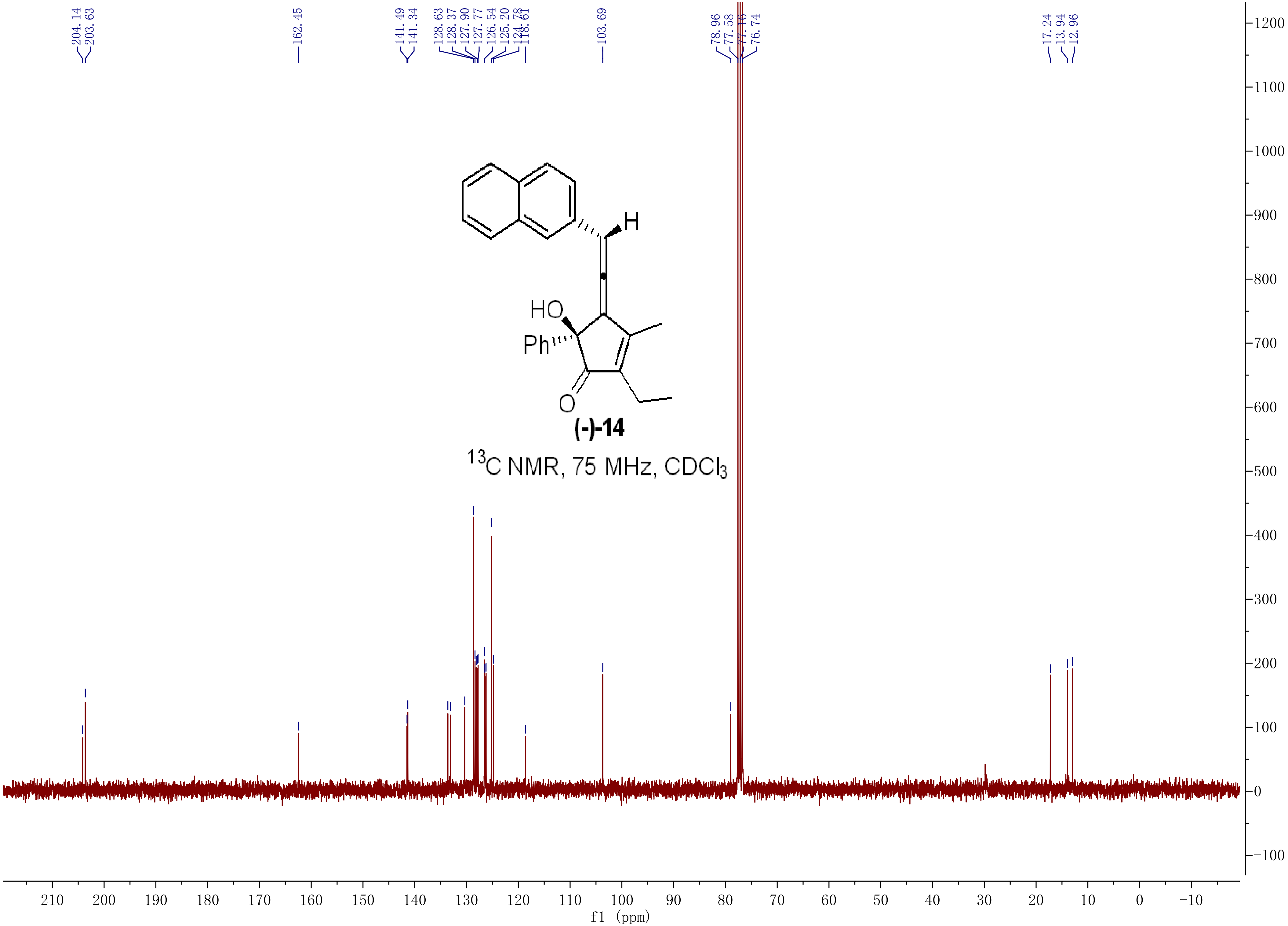

Supplementary Figure 57. ^{13}C NMR of compound (-)-14.

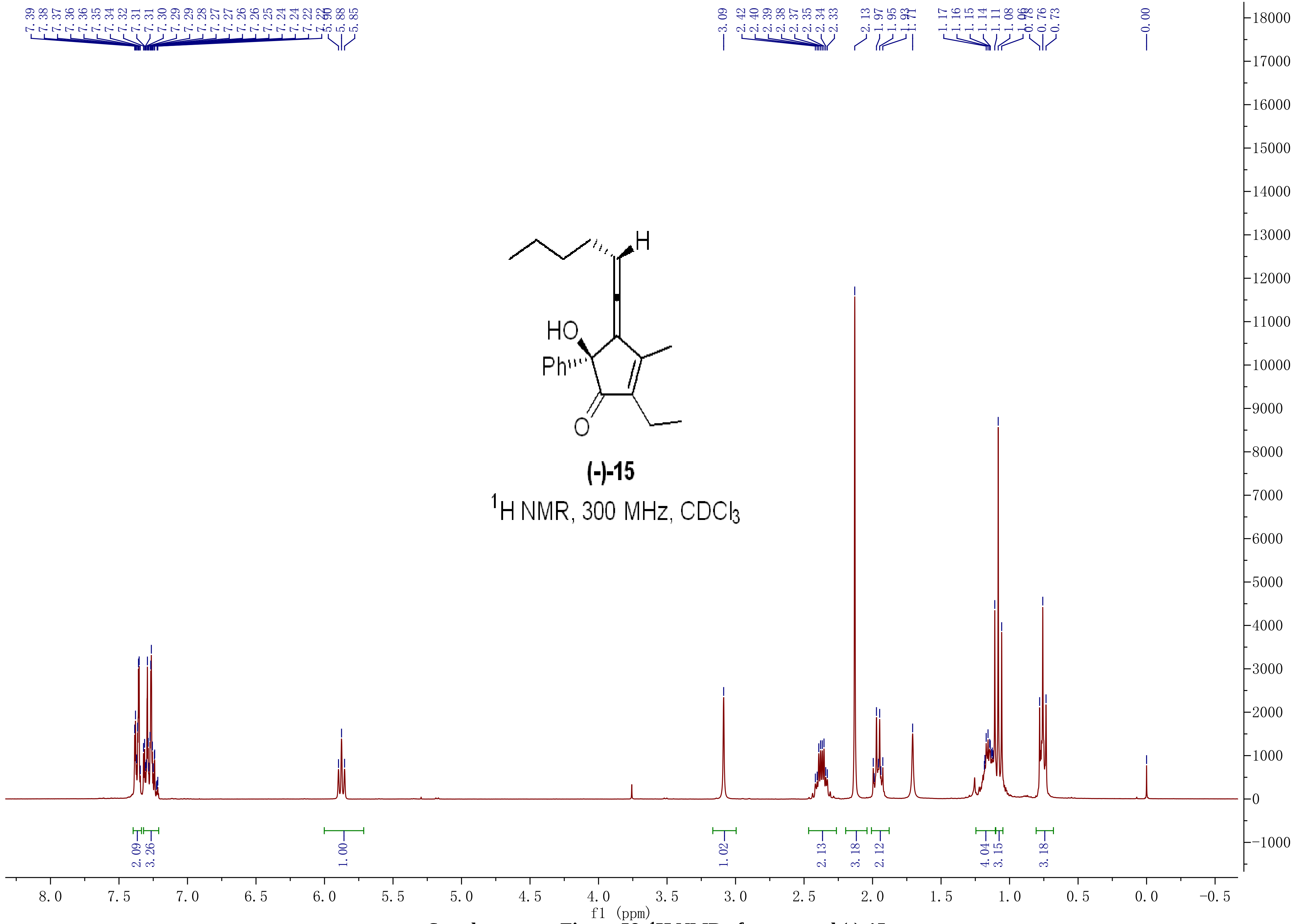

Supplementary Figure 58. ¹H NMR of compound (-)-15.

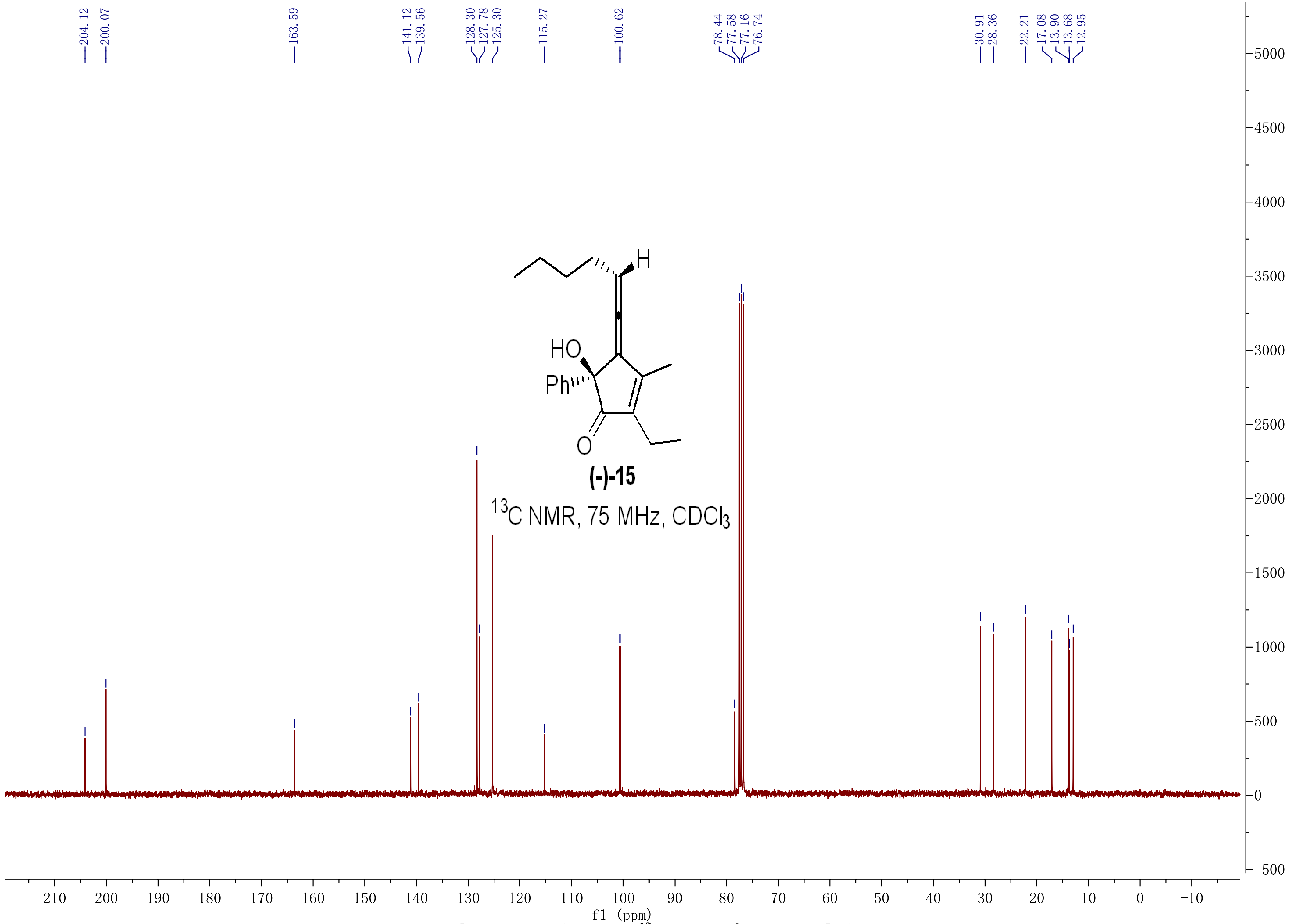

Supplementary Figure 59. ^{13}C NMR of compound (-)-15.

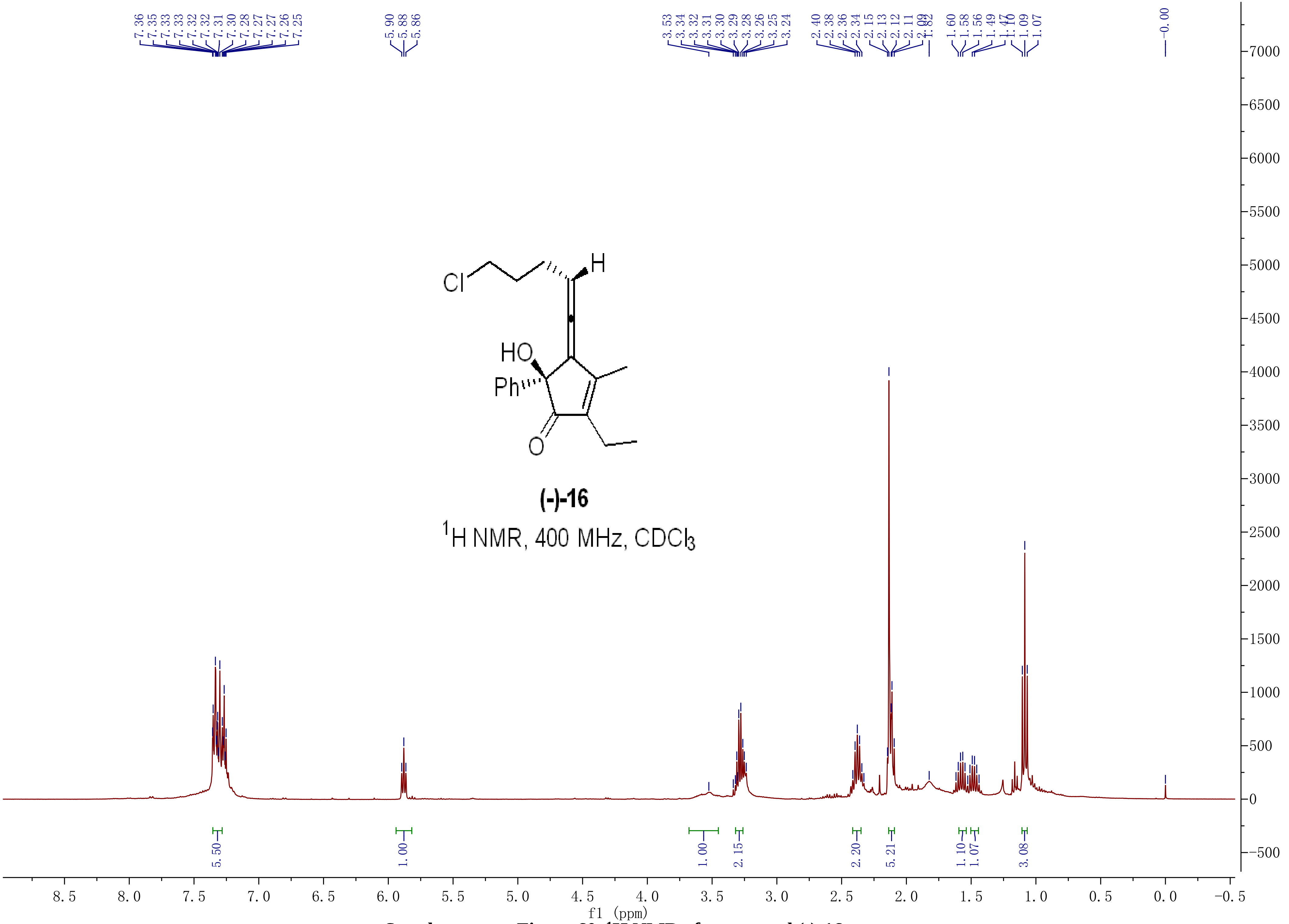

Supplementary Figure 60. ^{1}H NMR of compound (-)-16.

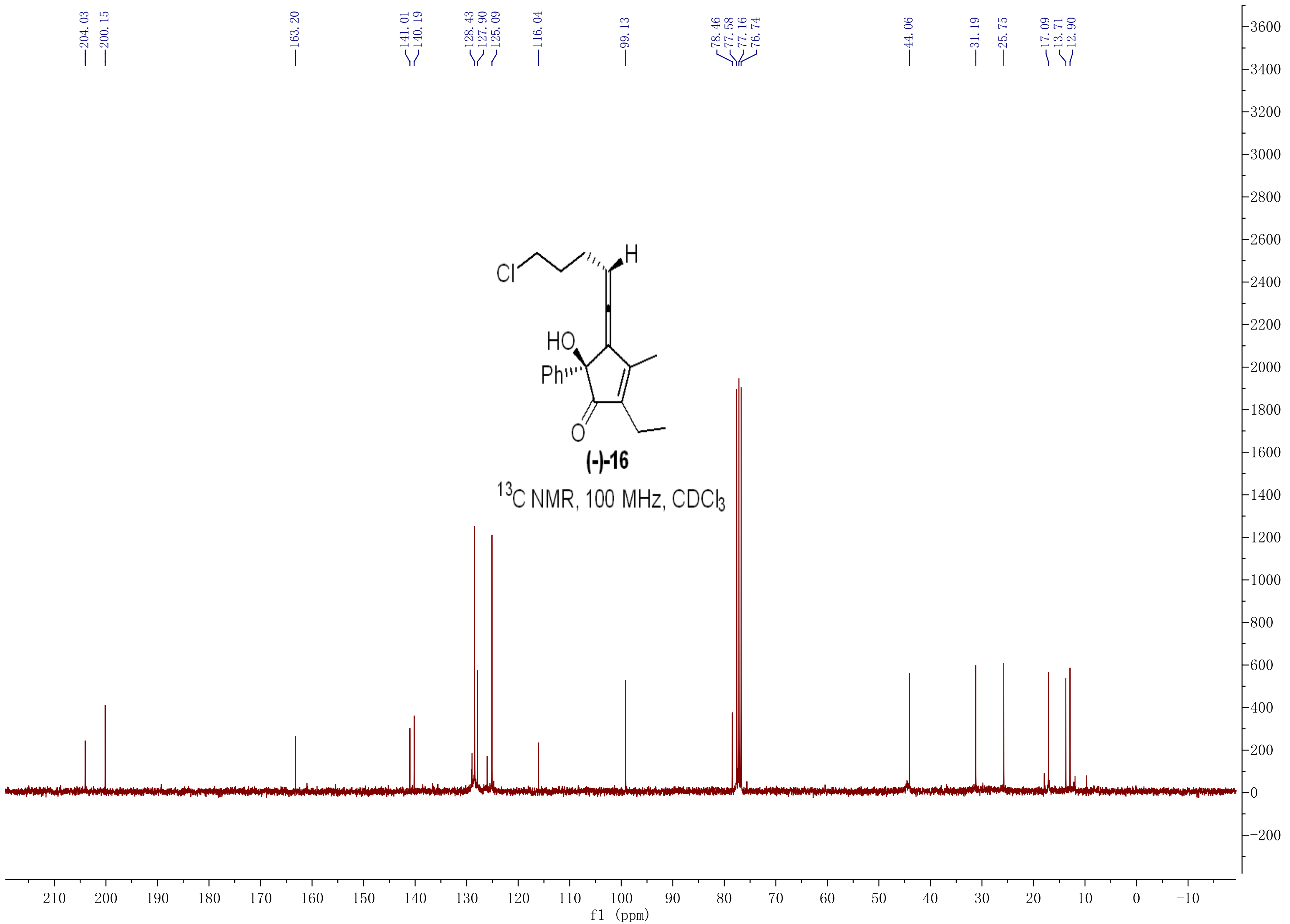

Supplementary Figure 61. ^{13}C NMR of compound (-)-16.

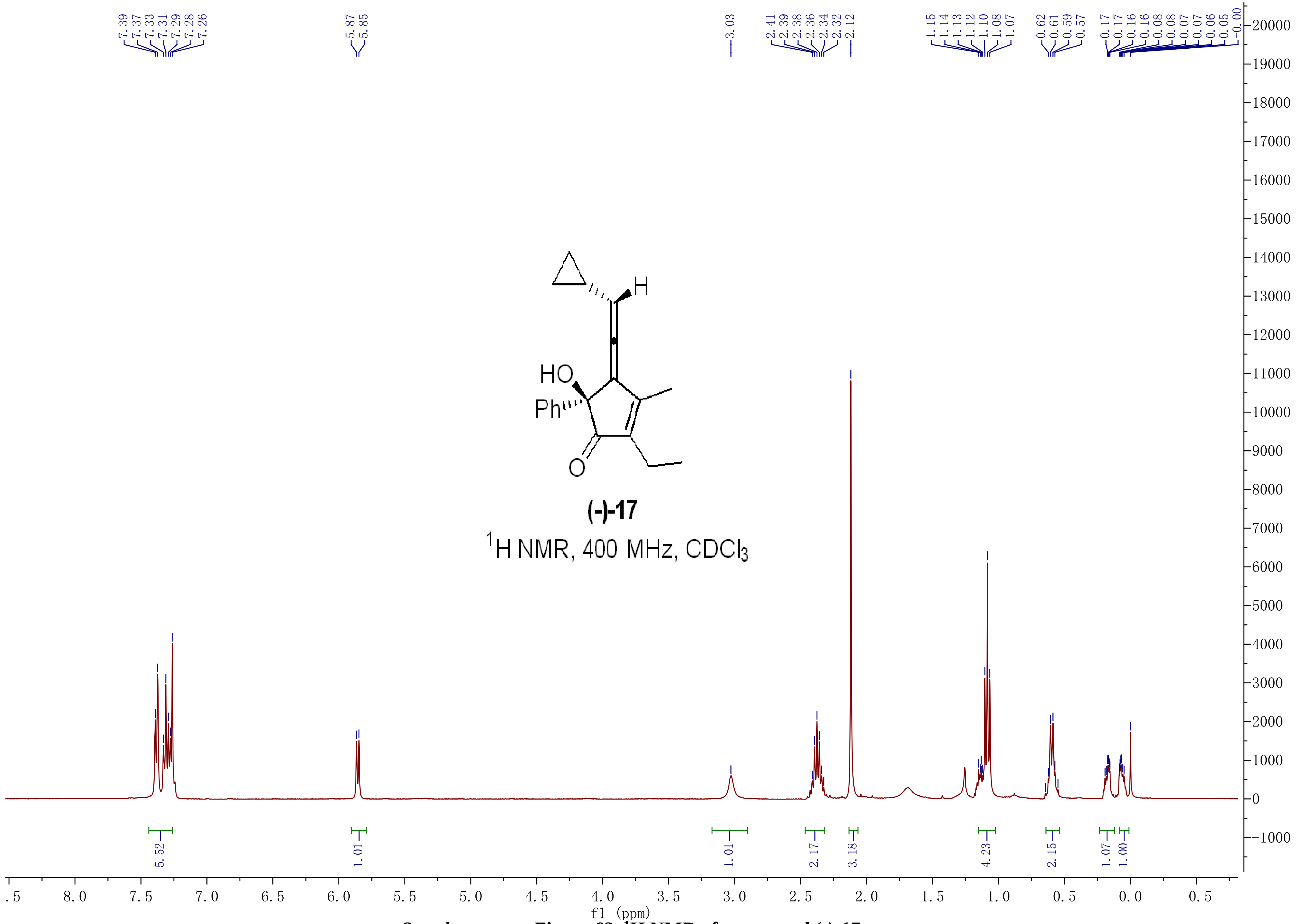

Supplementary Figure 62. ^{1}H NMR of compound (-)-17.

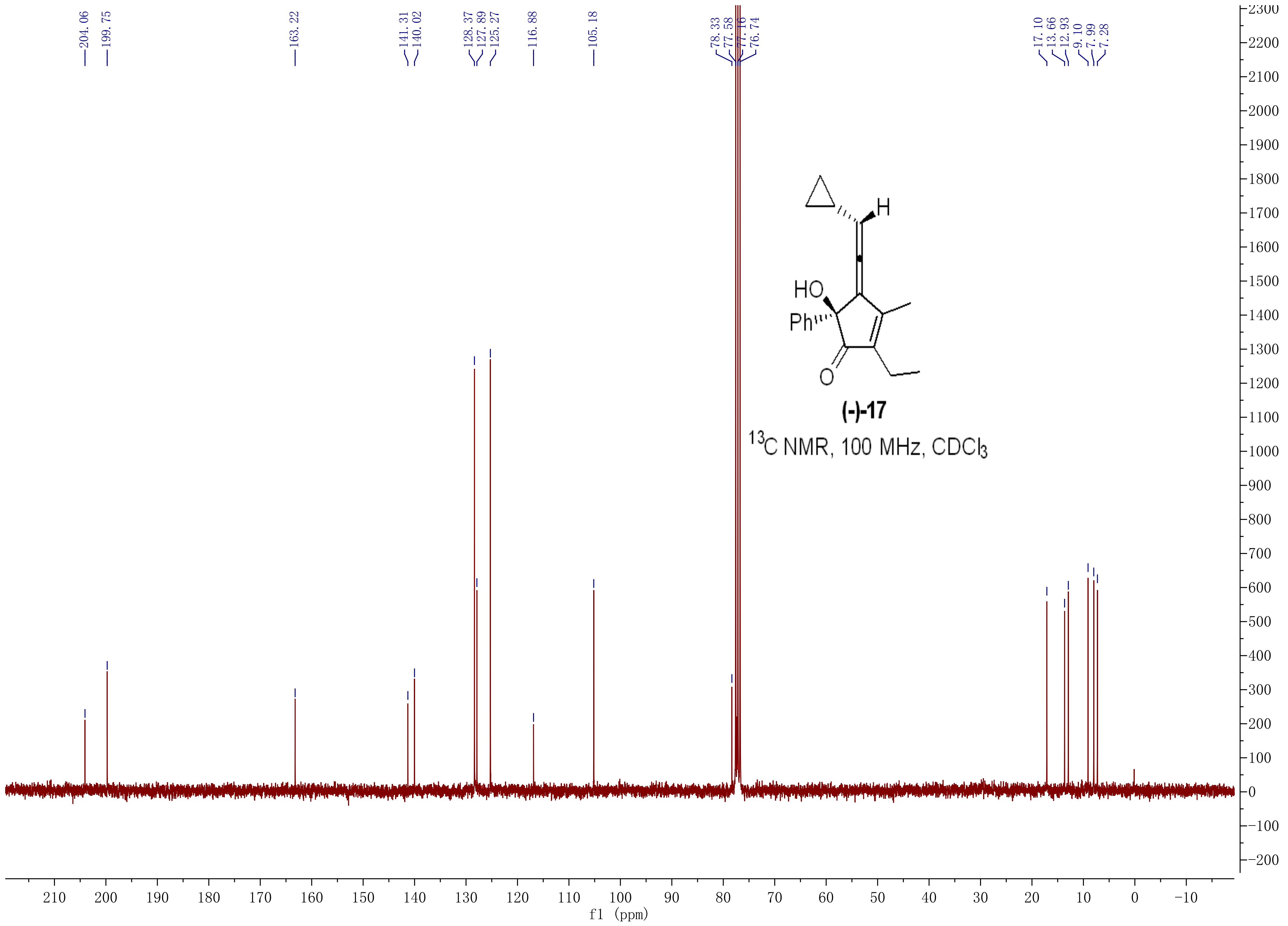

Supplementary Figure 63. ^{13}C NMR of compound (-)-17.

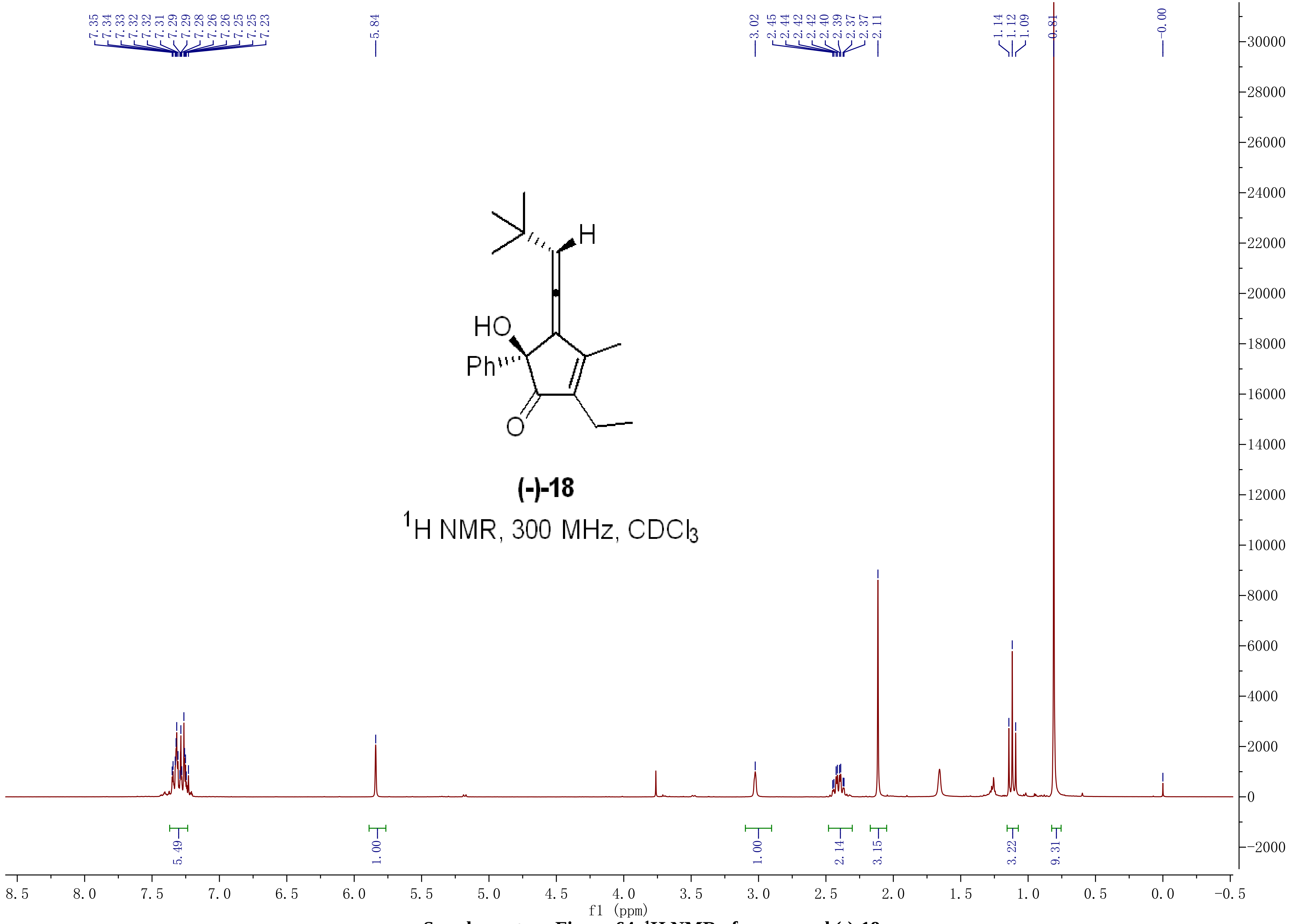

Supplementary Figure 64. ^{1}H NMR of compound (-)-18.

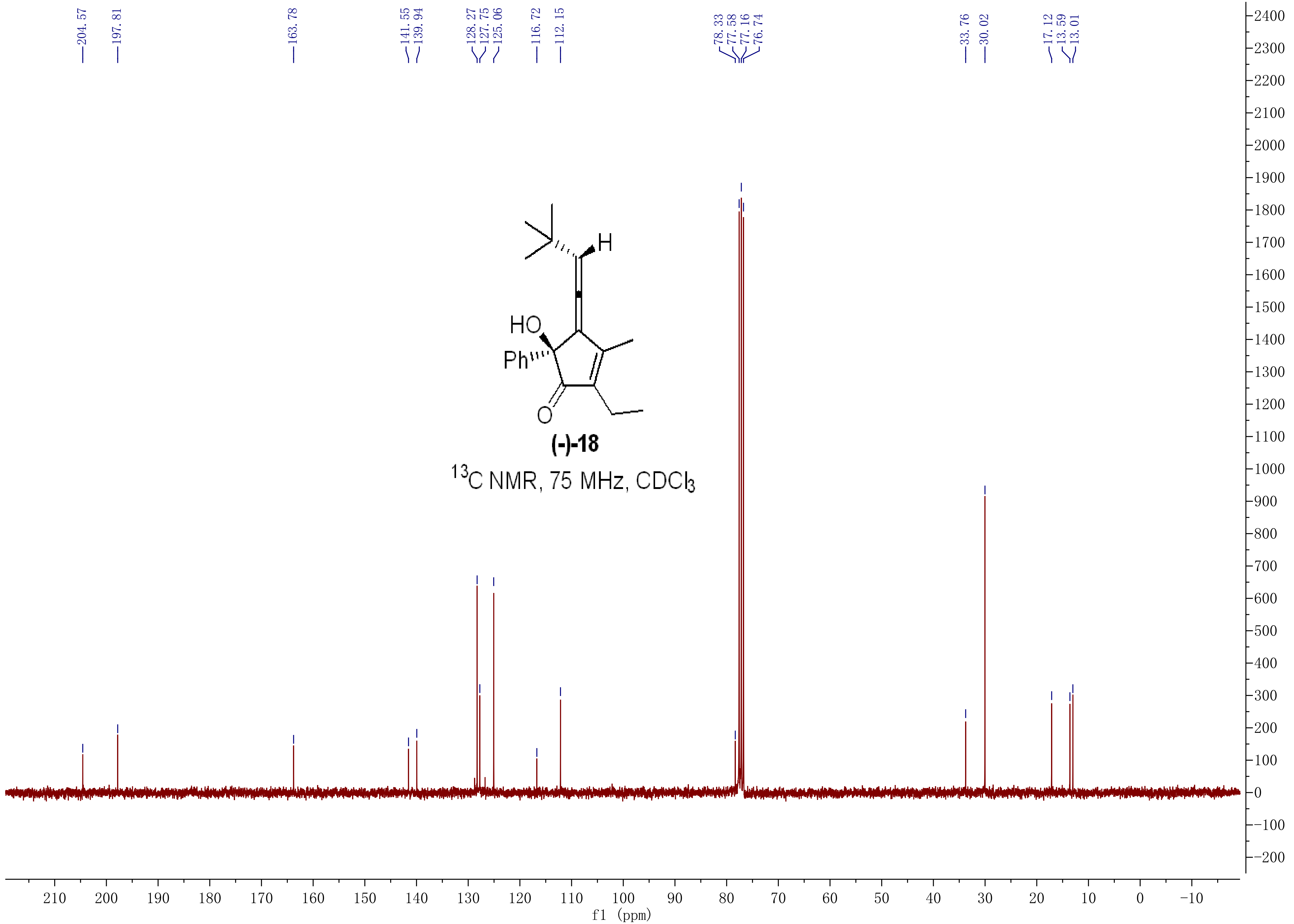

Supplementary Figure 65. ^{13}C NMR of compound (-)-18.

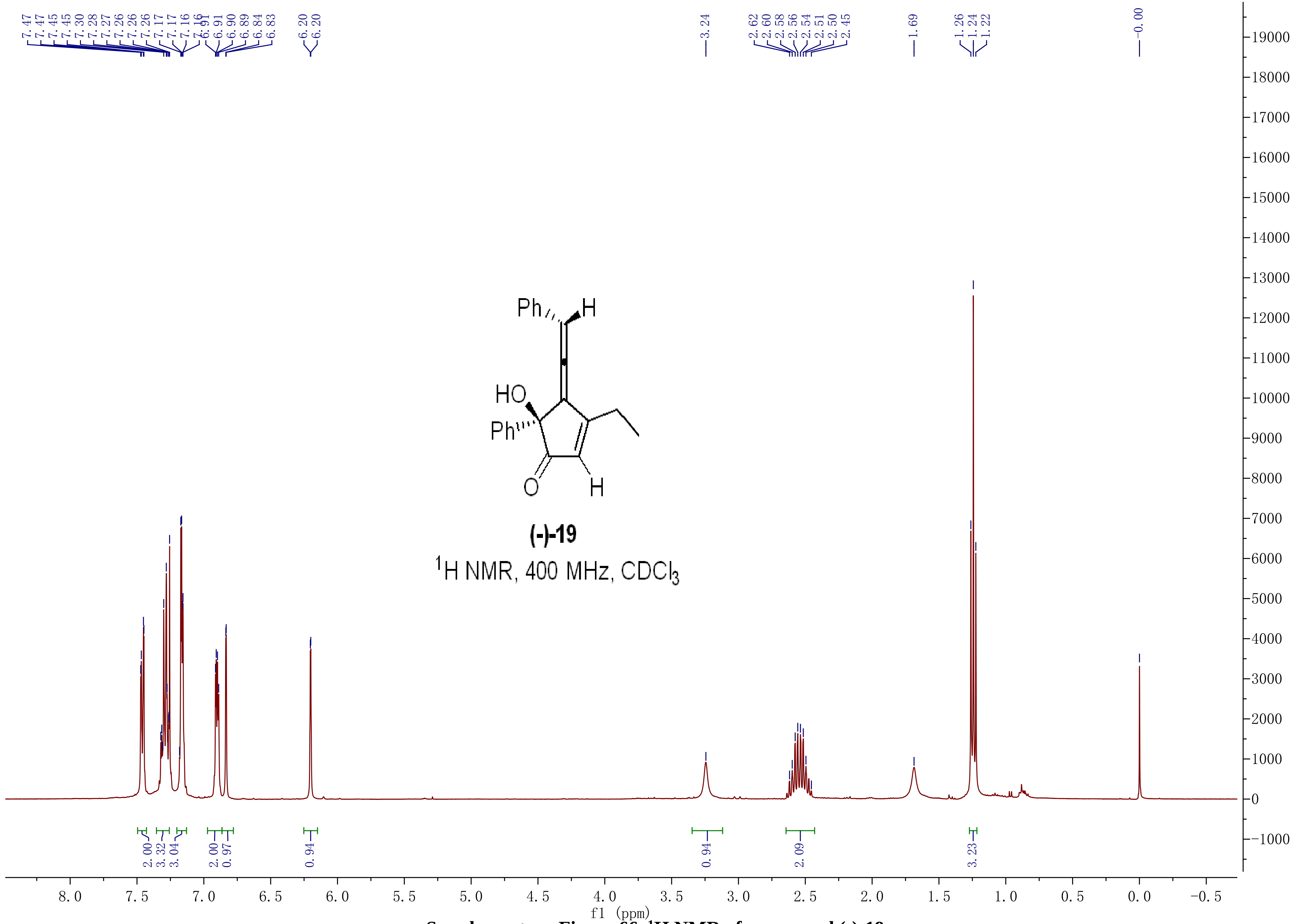

Supplementary Figure 66. ^{1}H NMR of compound (-)-19.

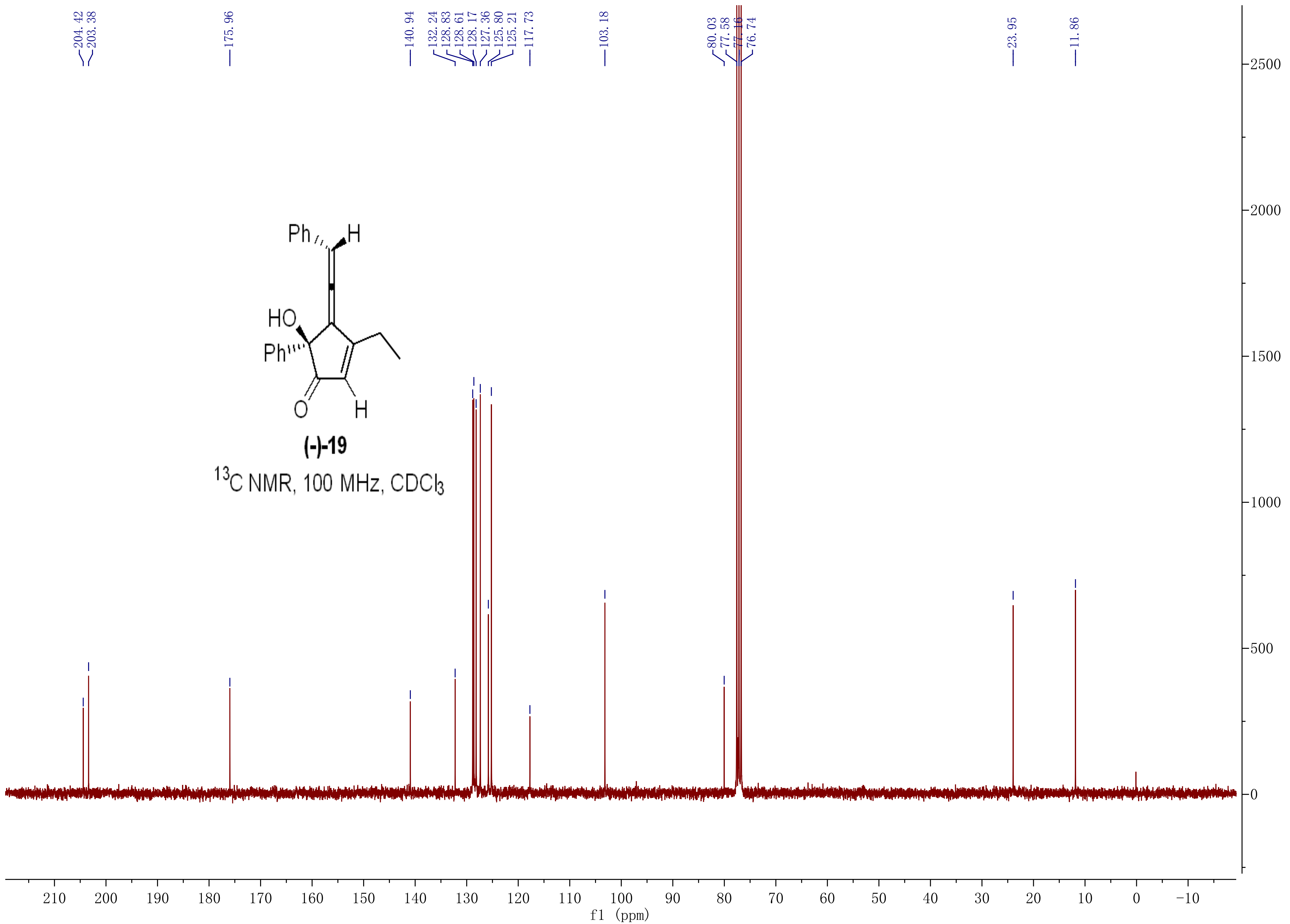

Supplementary Figure 67. ^{13}C NMR of compound (-)-19.

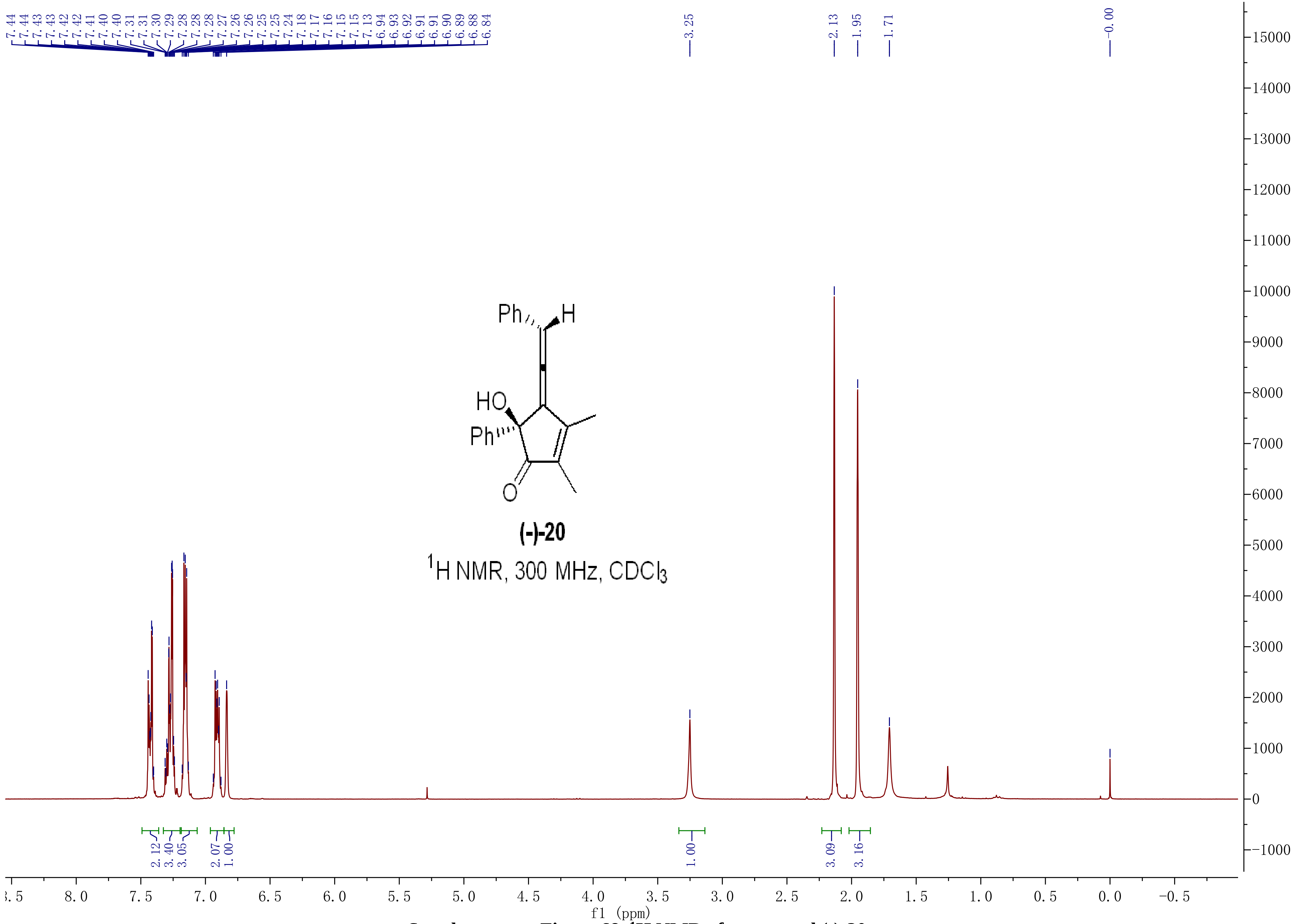

Supplementary Figure 68. ^{1}H NMR of compound (-)-20.

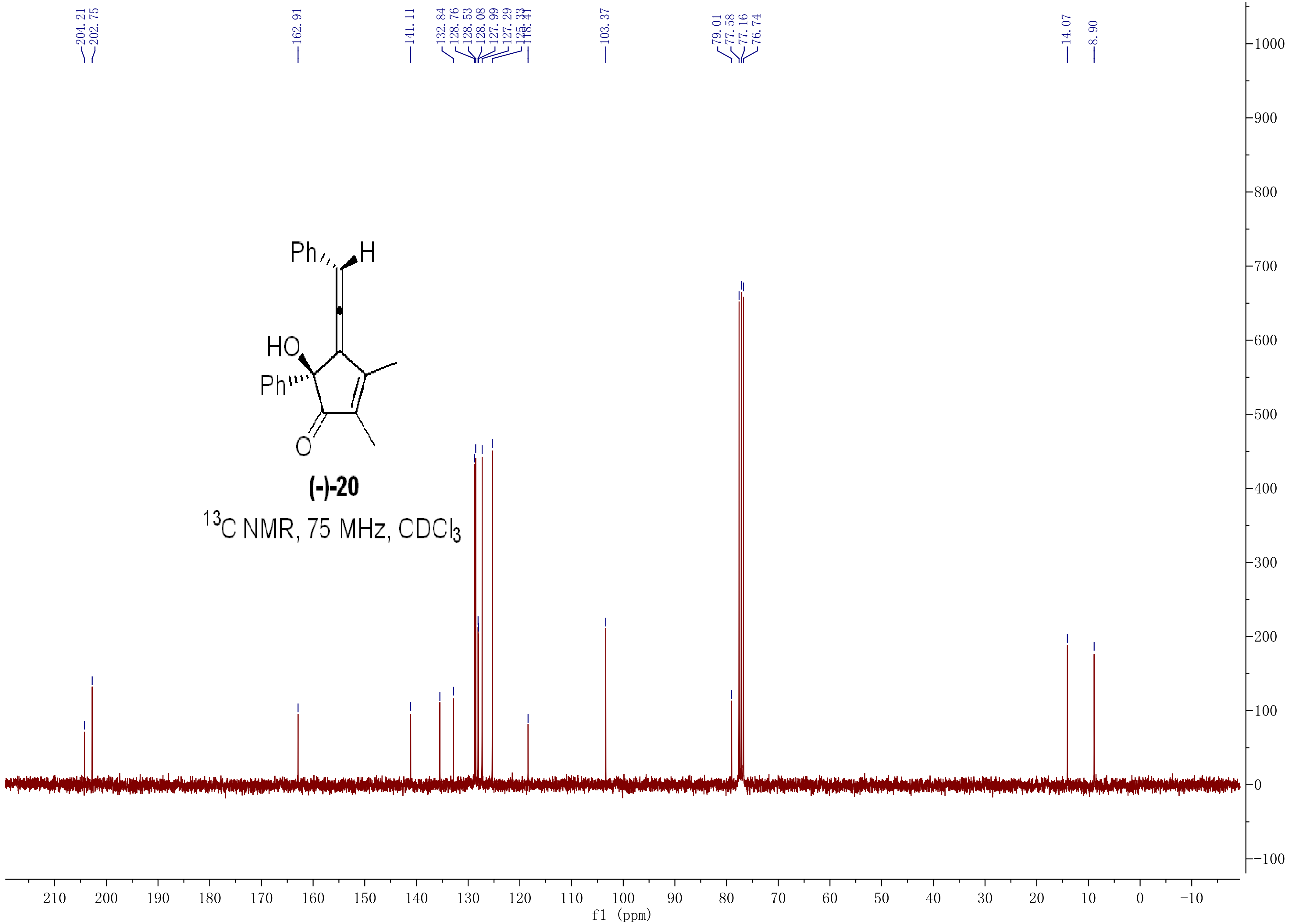

Supplementary Figure 69. ^{13}C NMR of compound (-)-20.

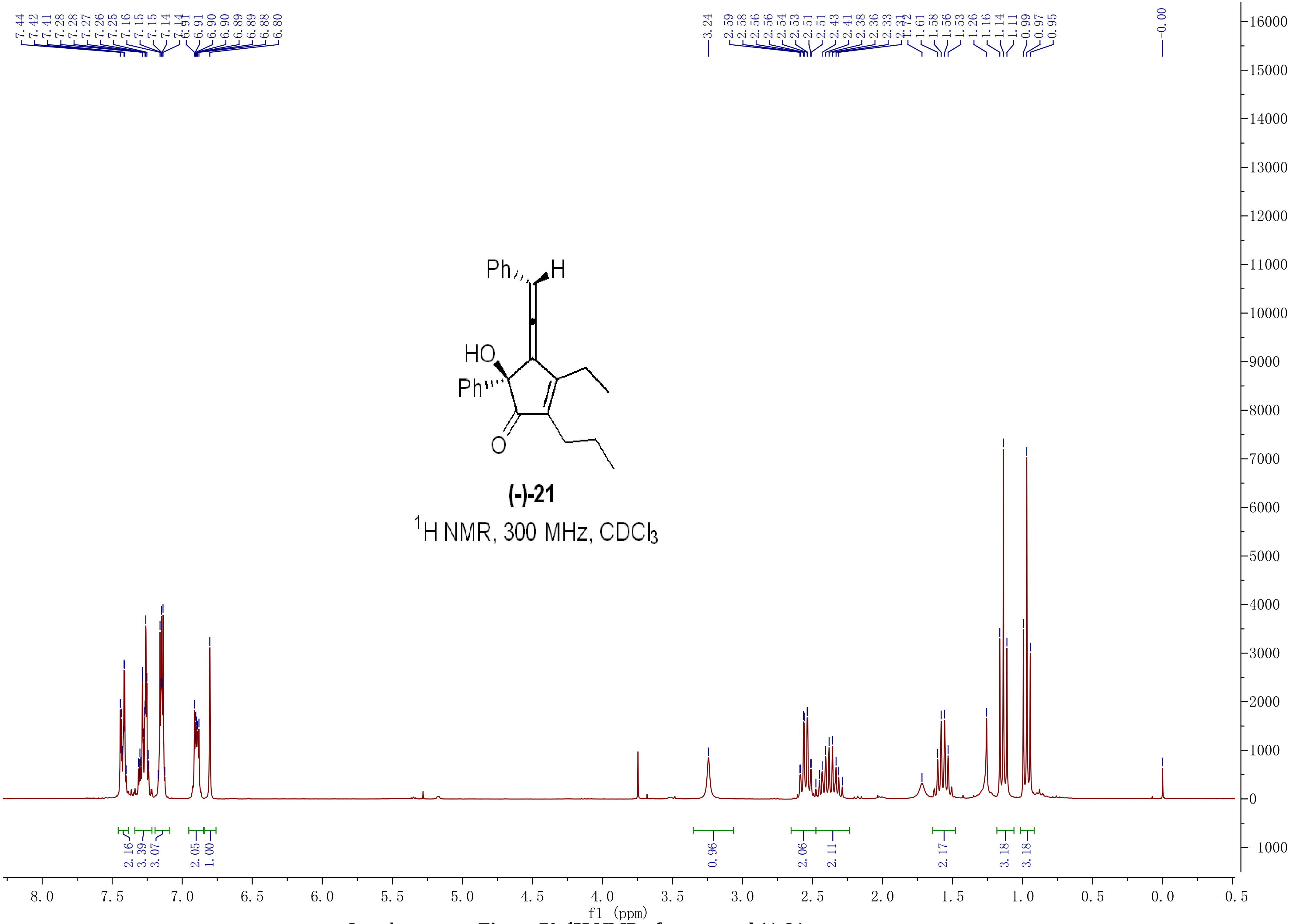

Supplementary Figure 70. ^{1}H NMR of compound (-)-21.

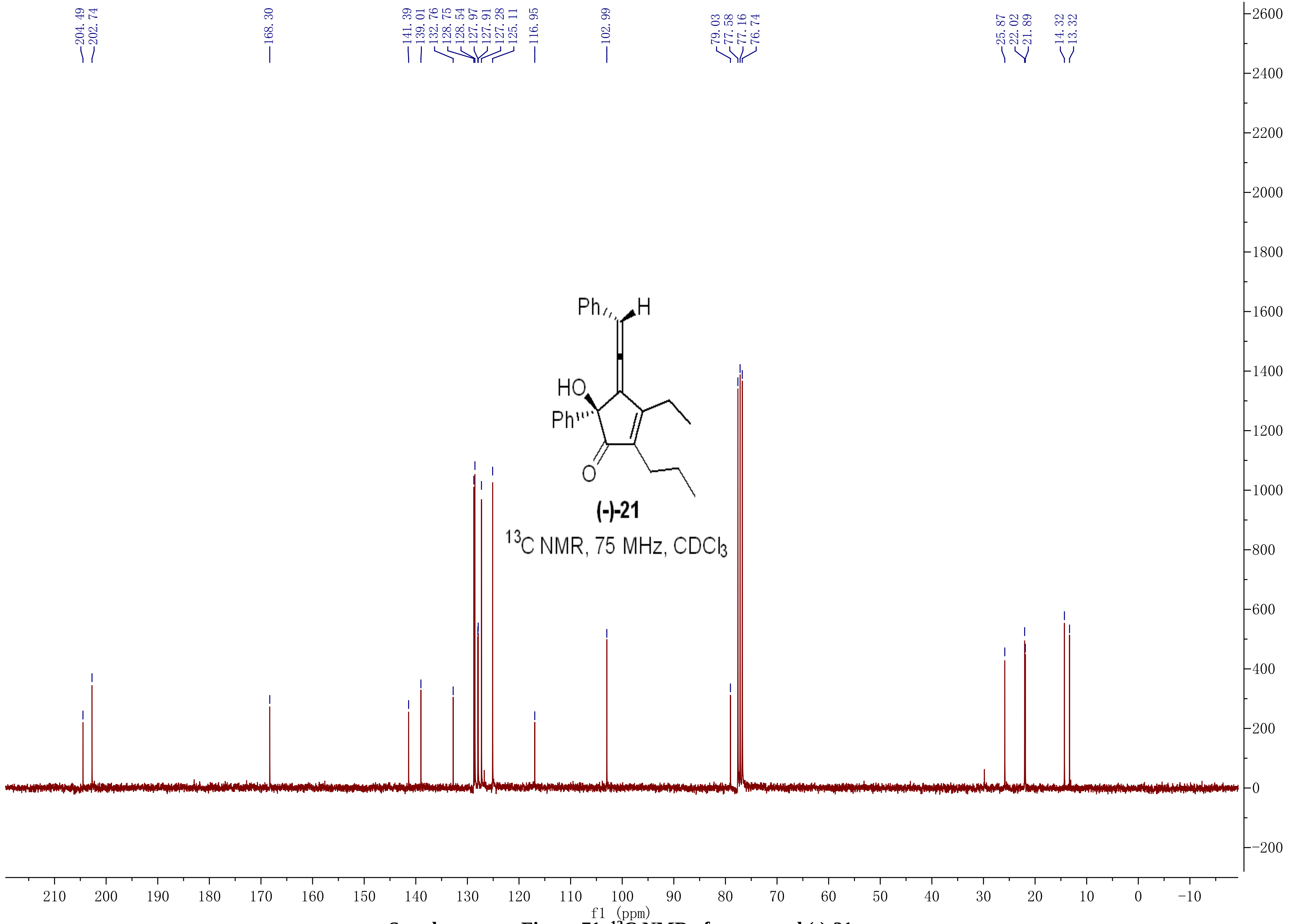

Supplementary Figure 71. ^{13}C NMR of compound (-)-21.

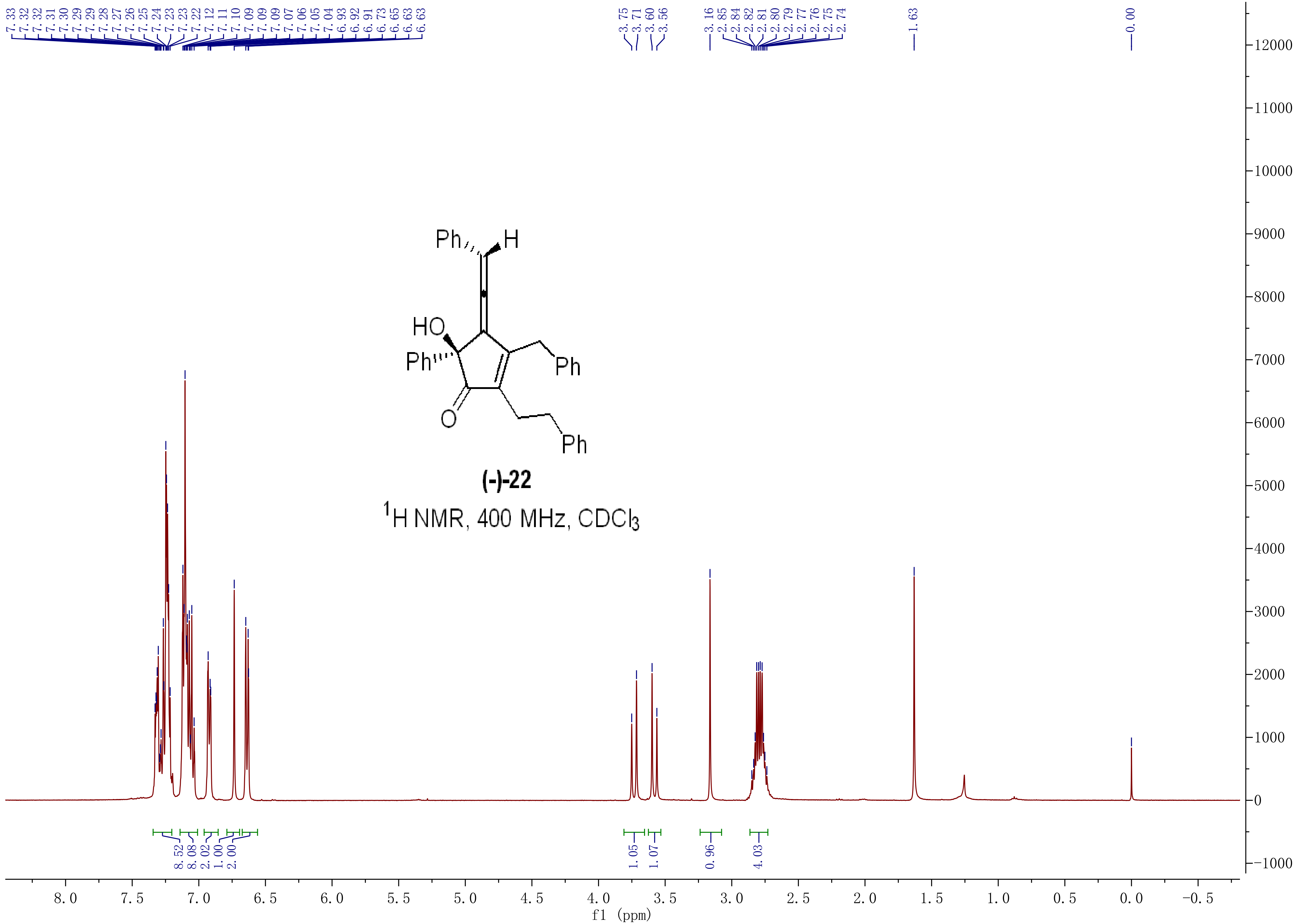

Supplementary Figure 72. ^{1}H NMR of compound (-)-22.

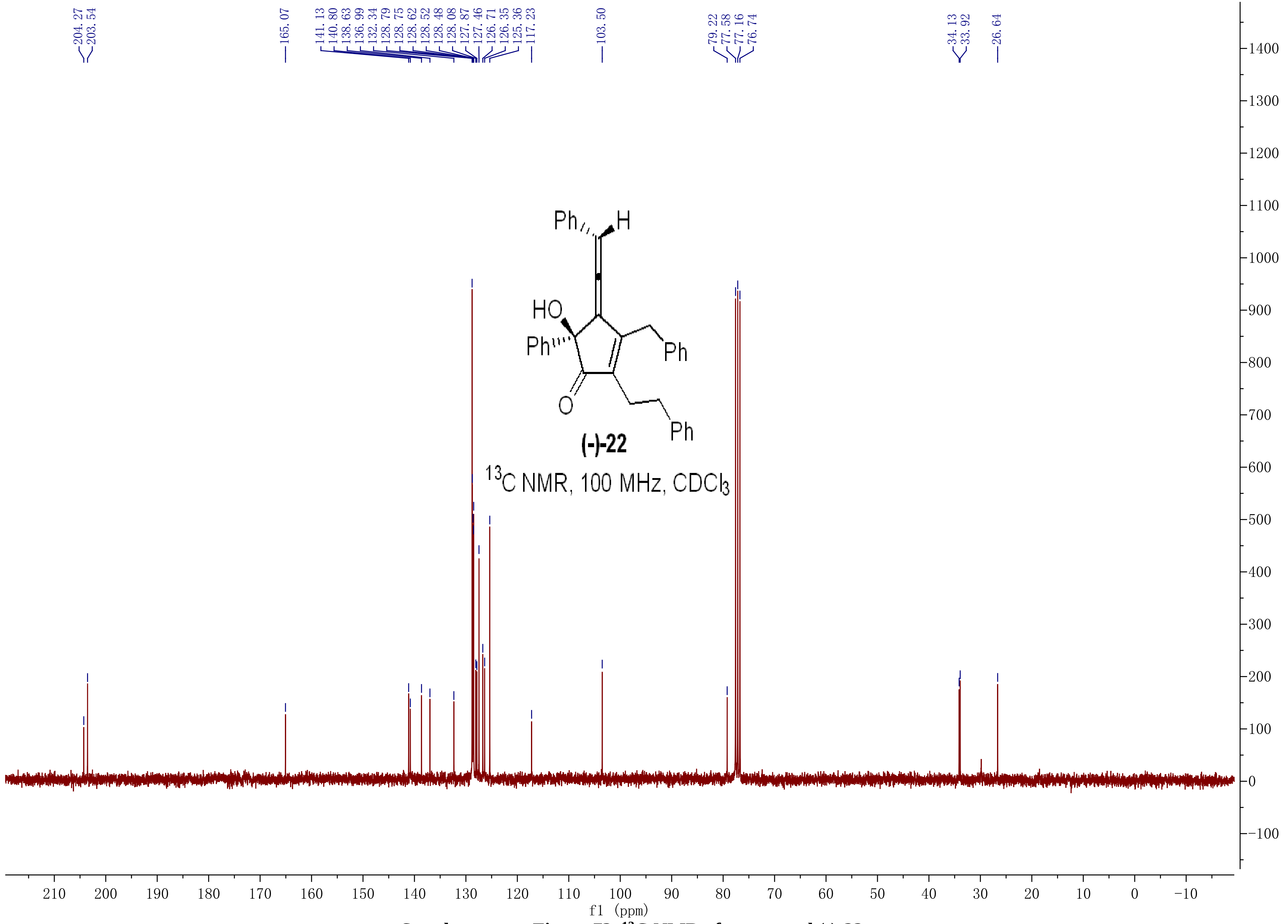

Supplementary Figure 73. ^{13}C NMR of compound (-)-22.

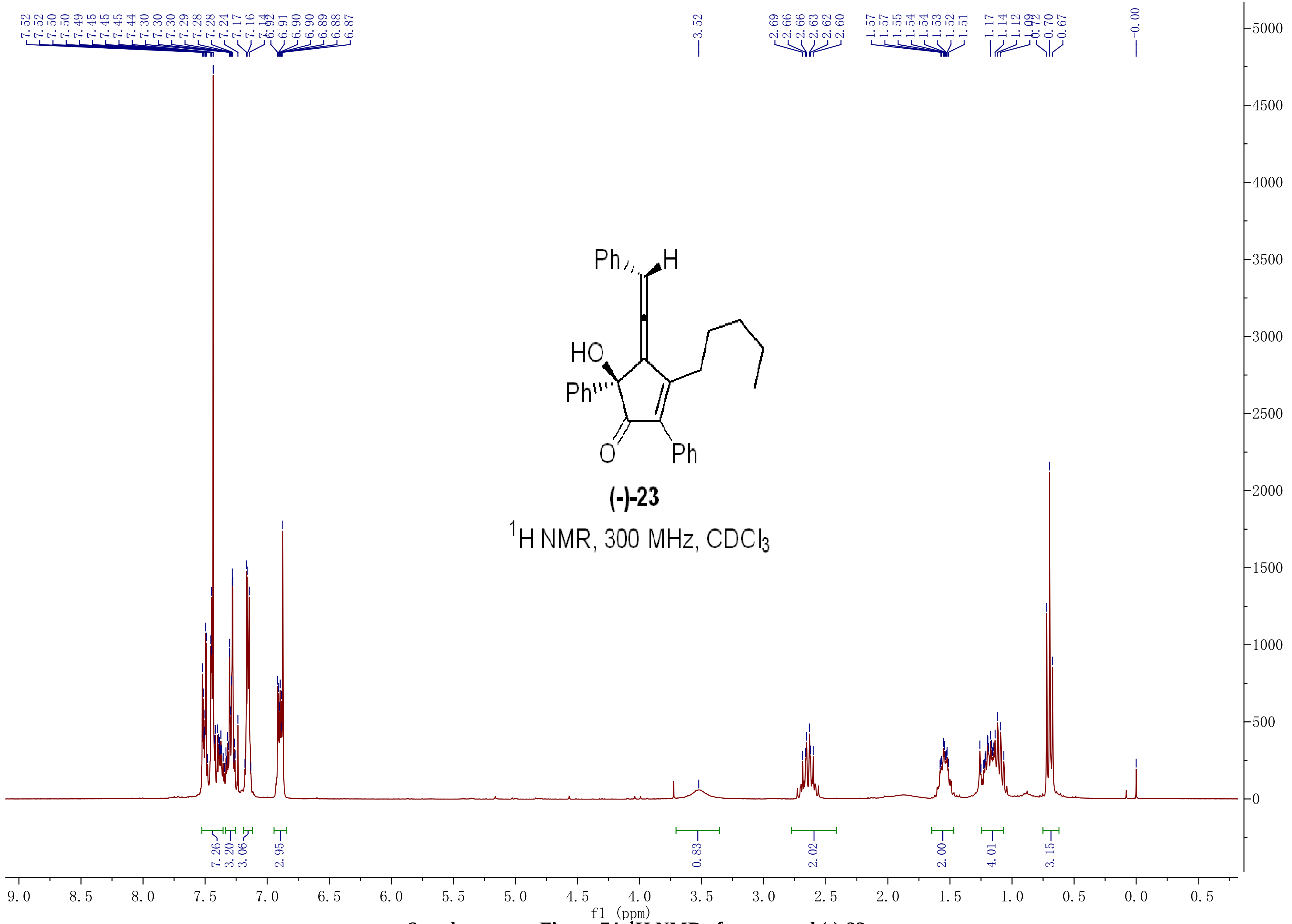

Supplementary Figure 74. ^{1}H NMR of compound (-)-23.

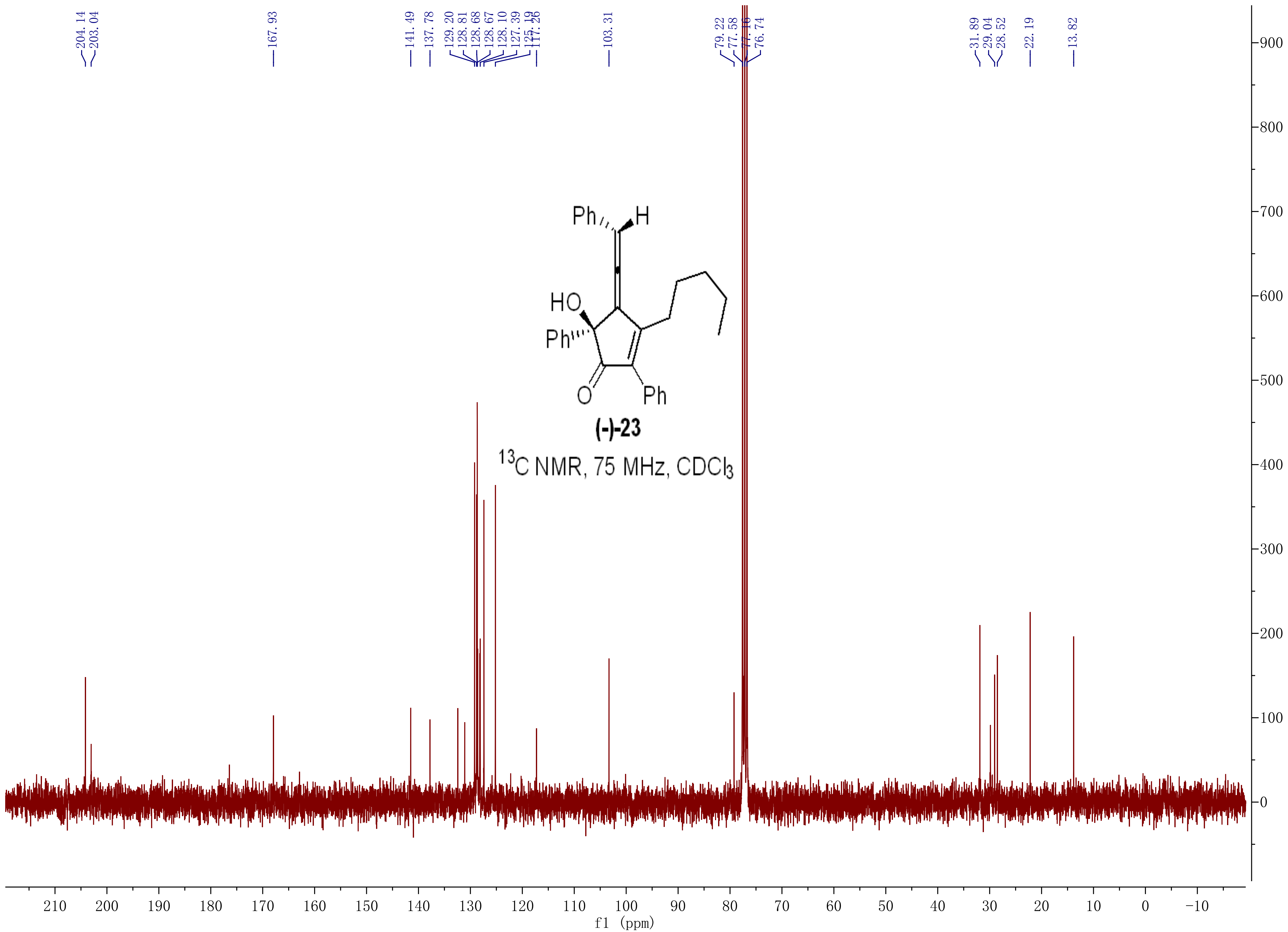

Supplementary Figure 75. ^{13}C NMR of compound (-)-23.

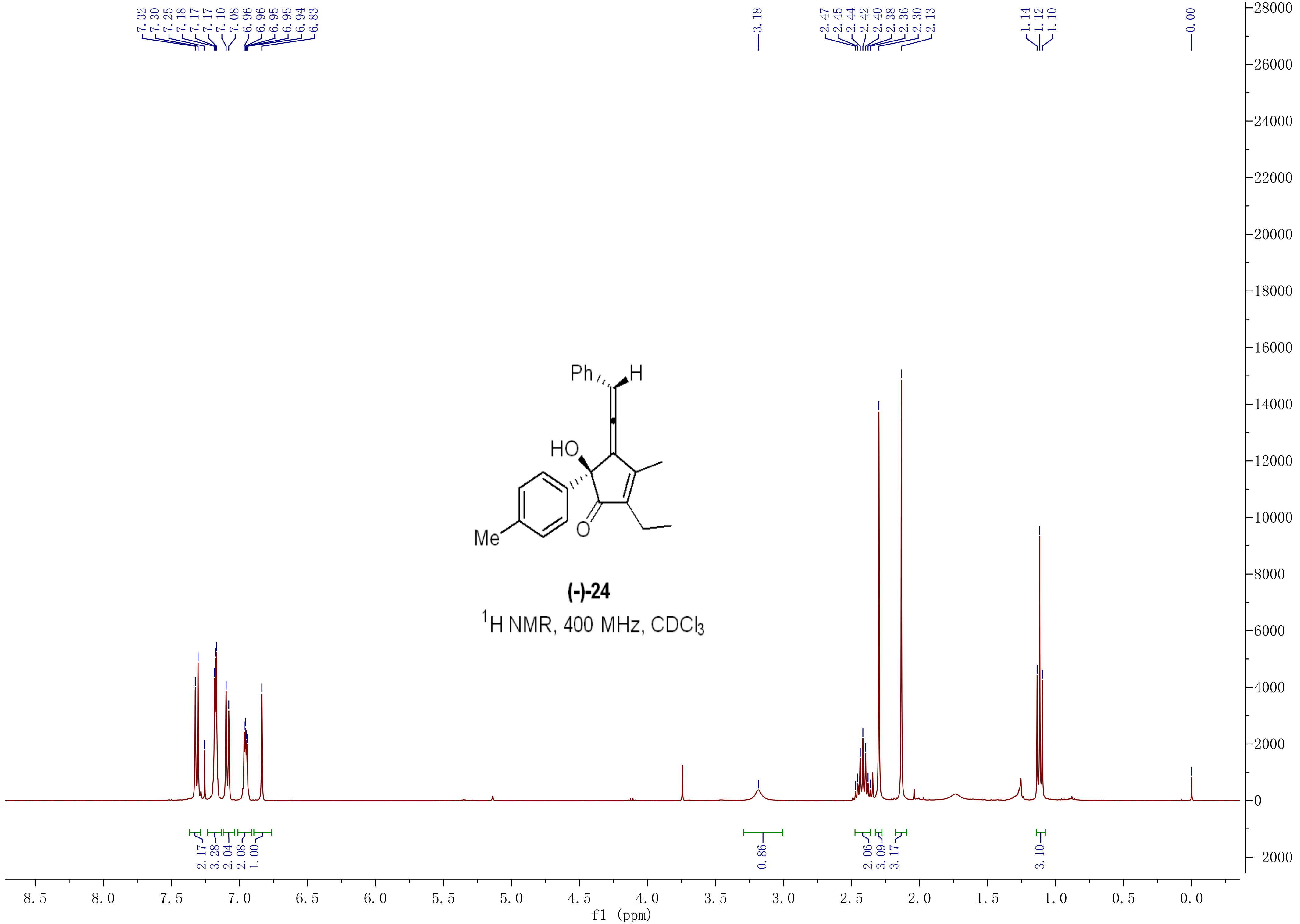

Supplementary Figure 76. ^{1}H NMR of compound (-)-24.

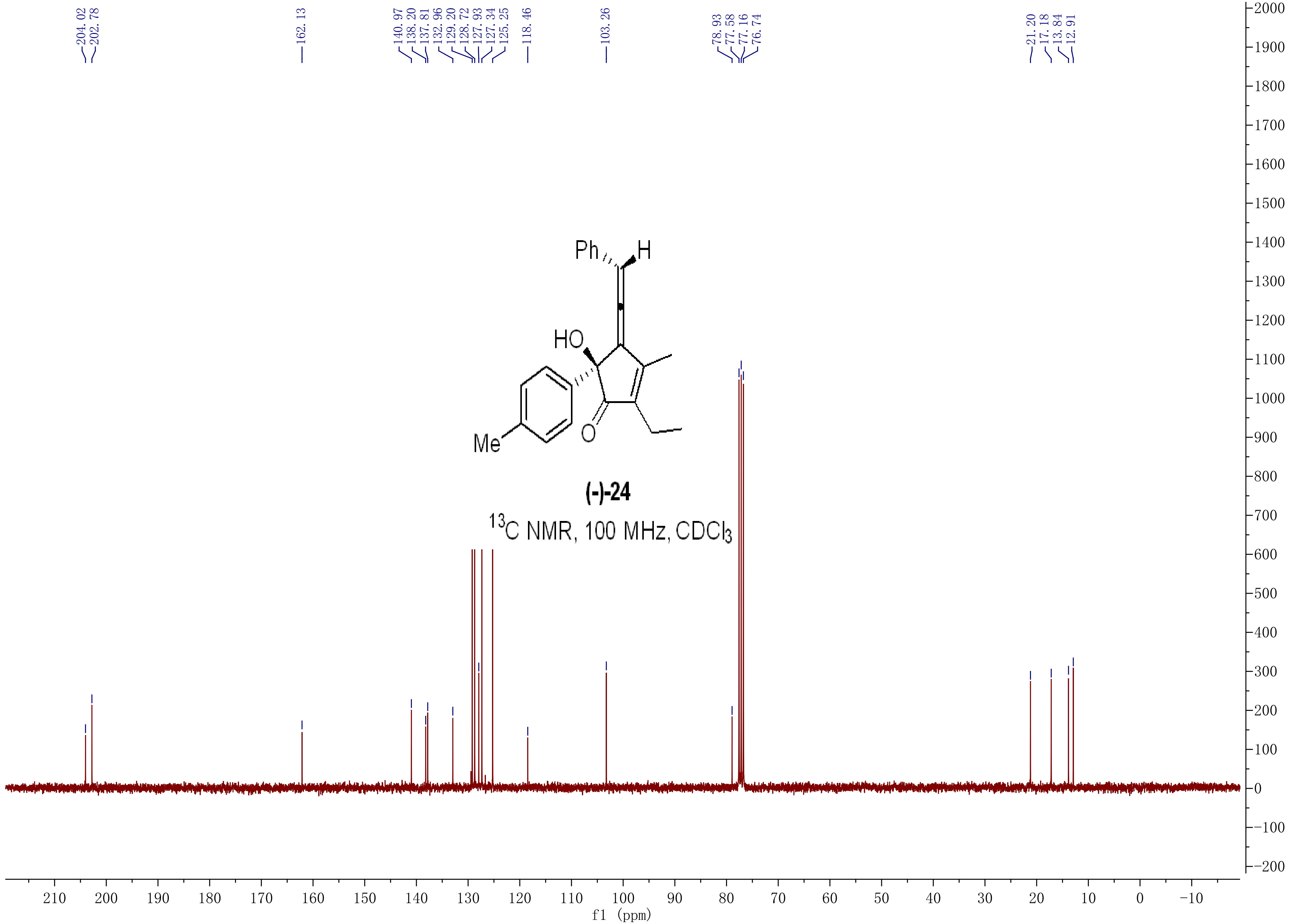

Supplementary Figure 77. ^{13}C NMR of compound (-)-24.

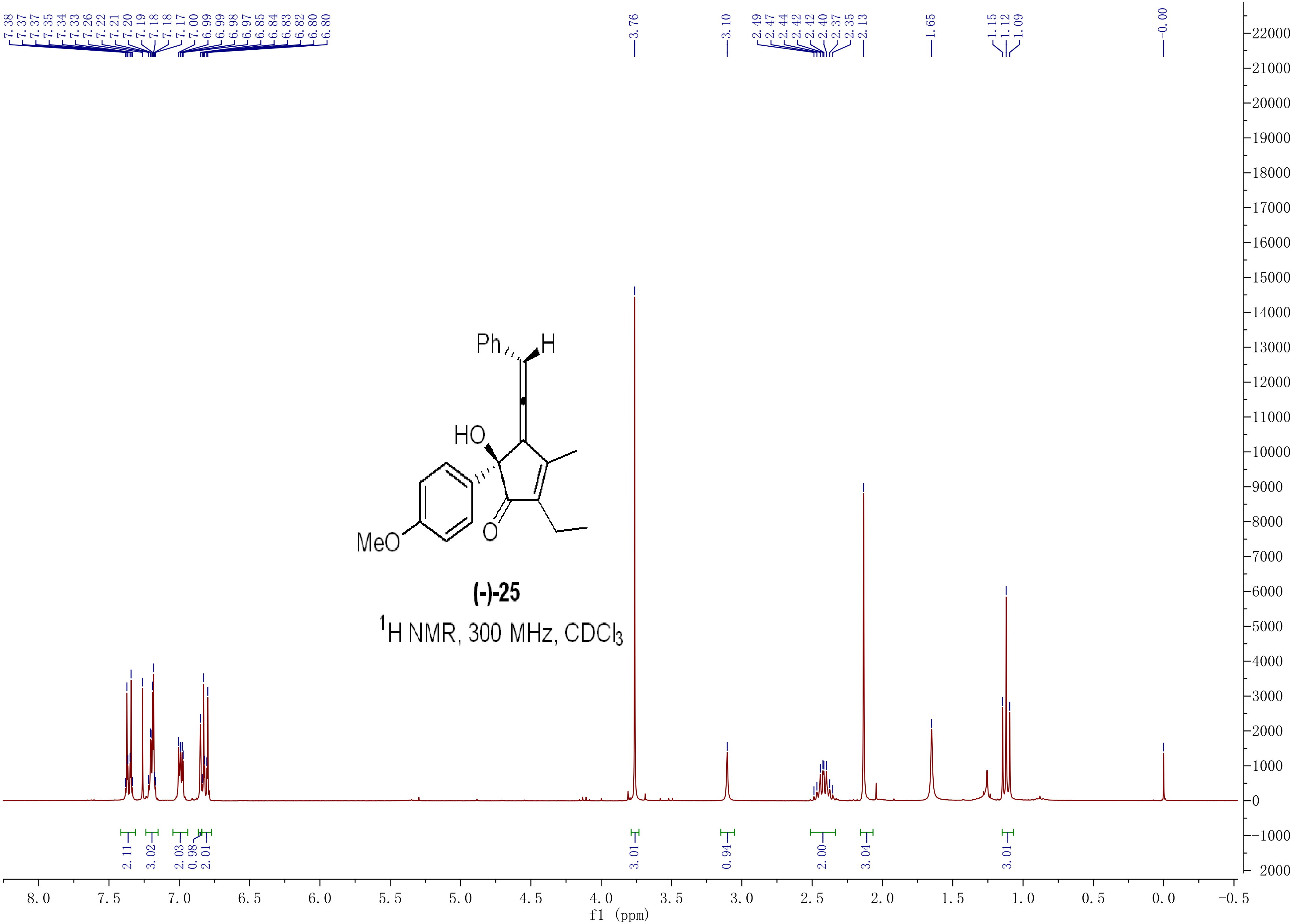

Supplementary Figure 78. ^{1}H NMR of compound (-)-25.

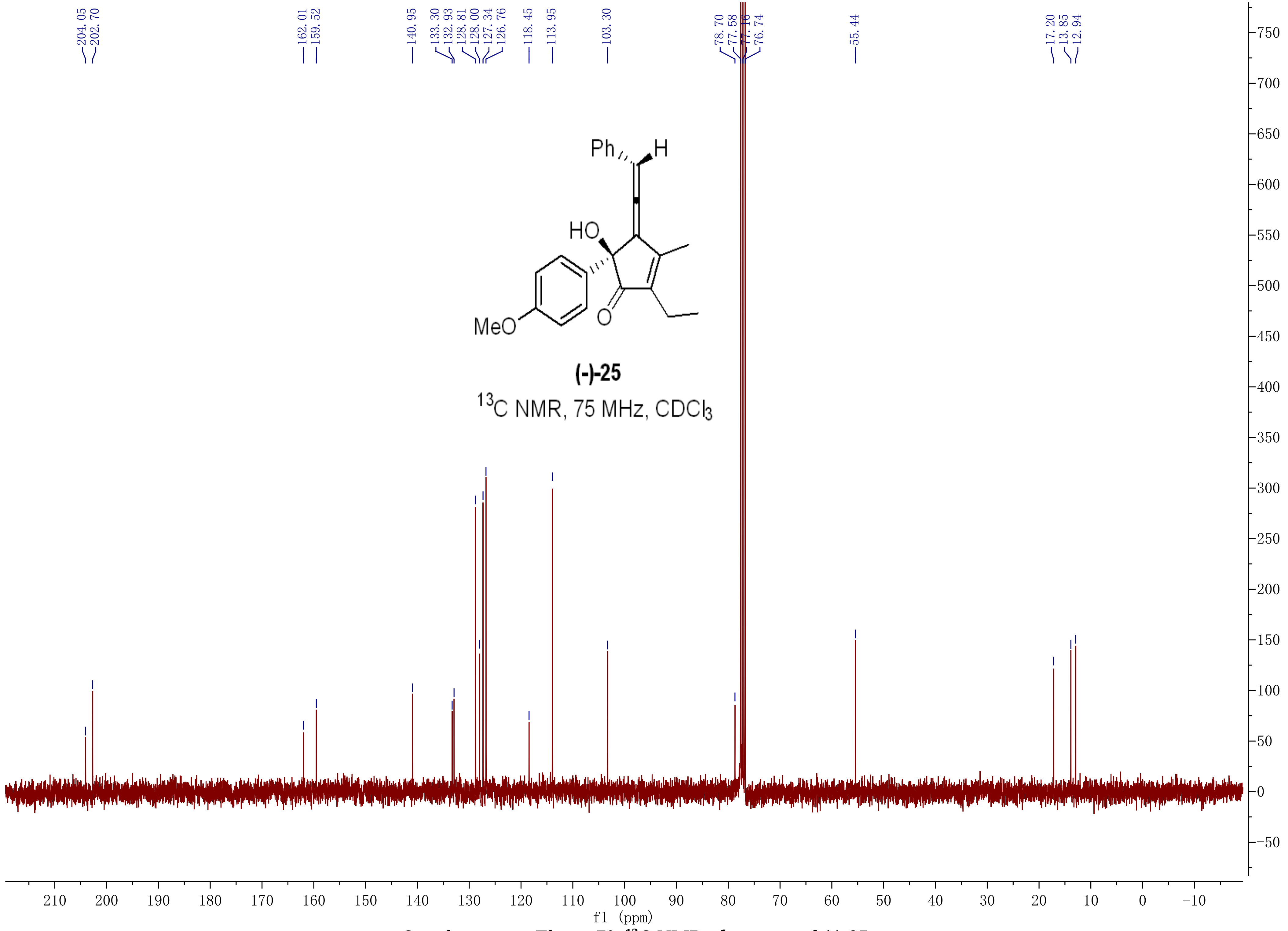

Supplementary Figure 79. ^{13}C NMR of compound (-)-25.

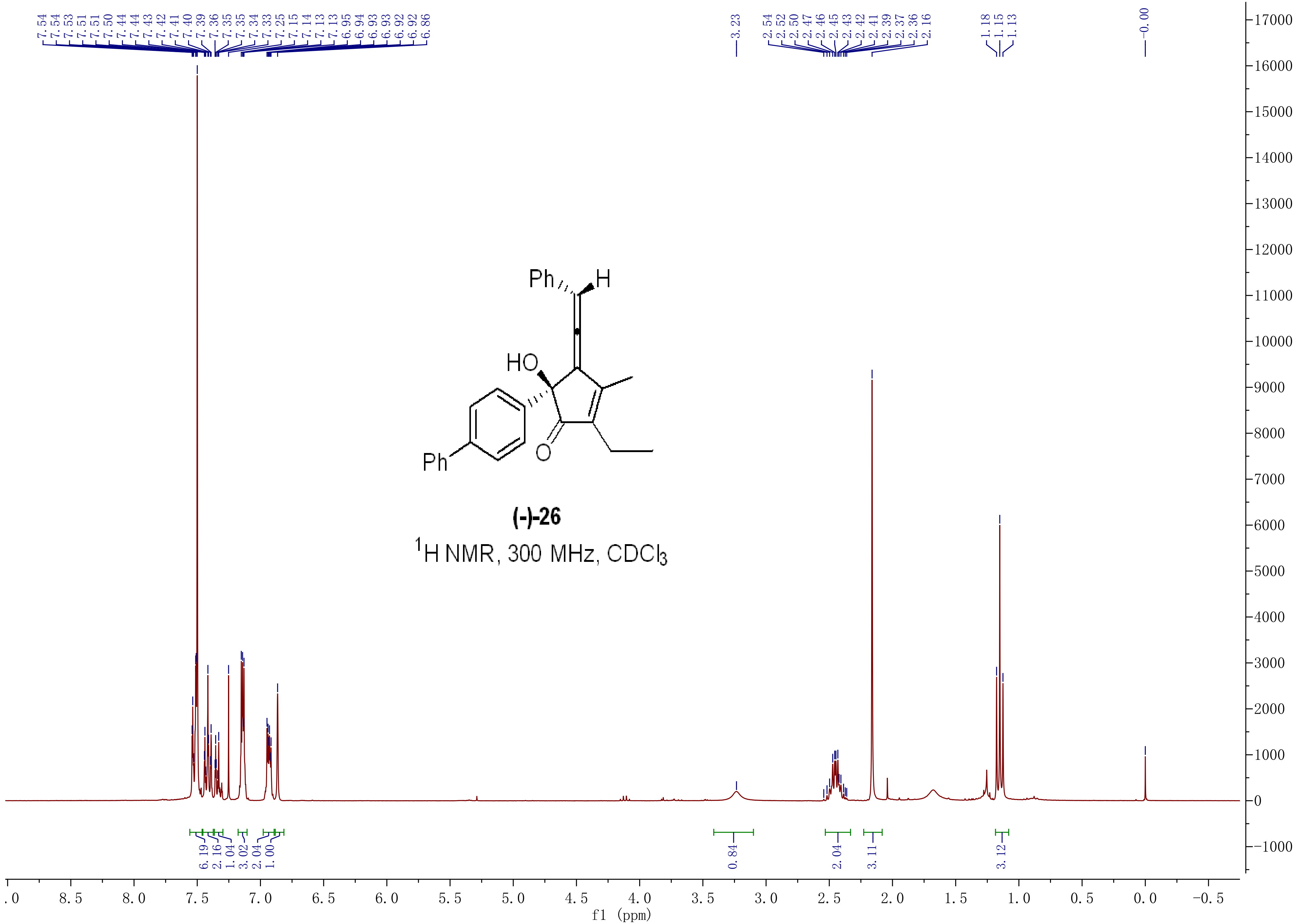

Supplementary Figure 80. ^{1}H NMR of compound (-)-26.

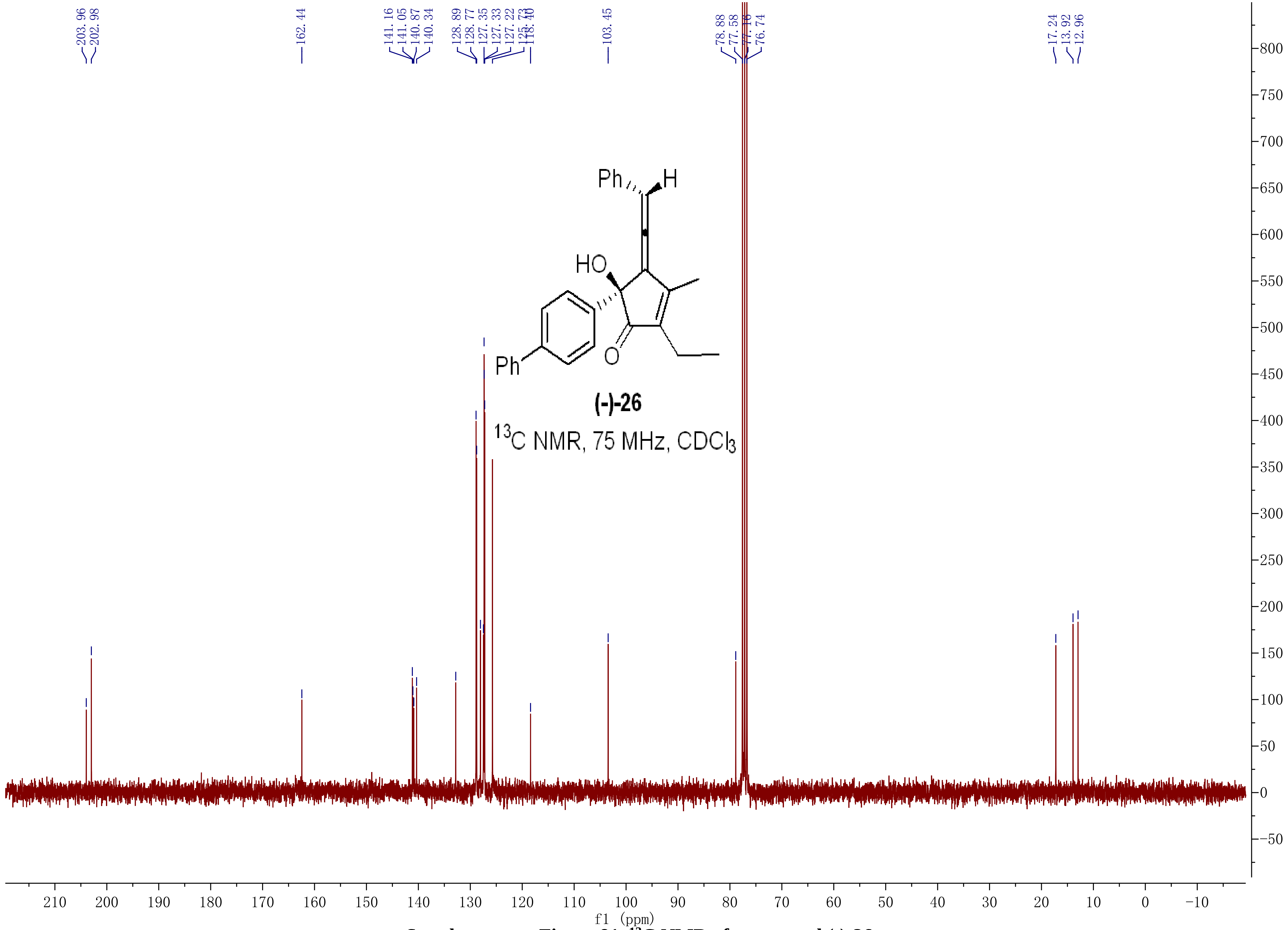

Supplementary Figure 81. ^{13}C NMR of compound (-)-26.

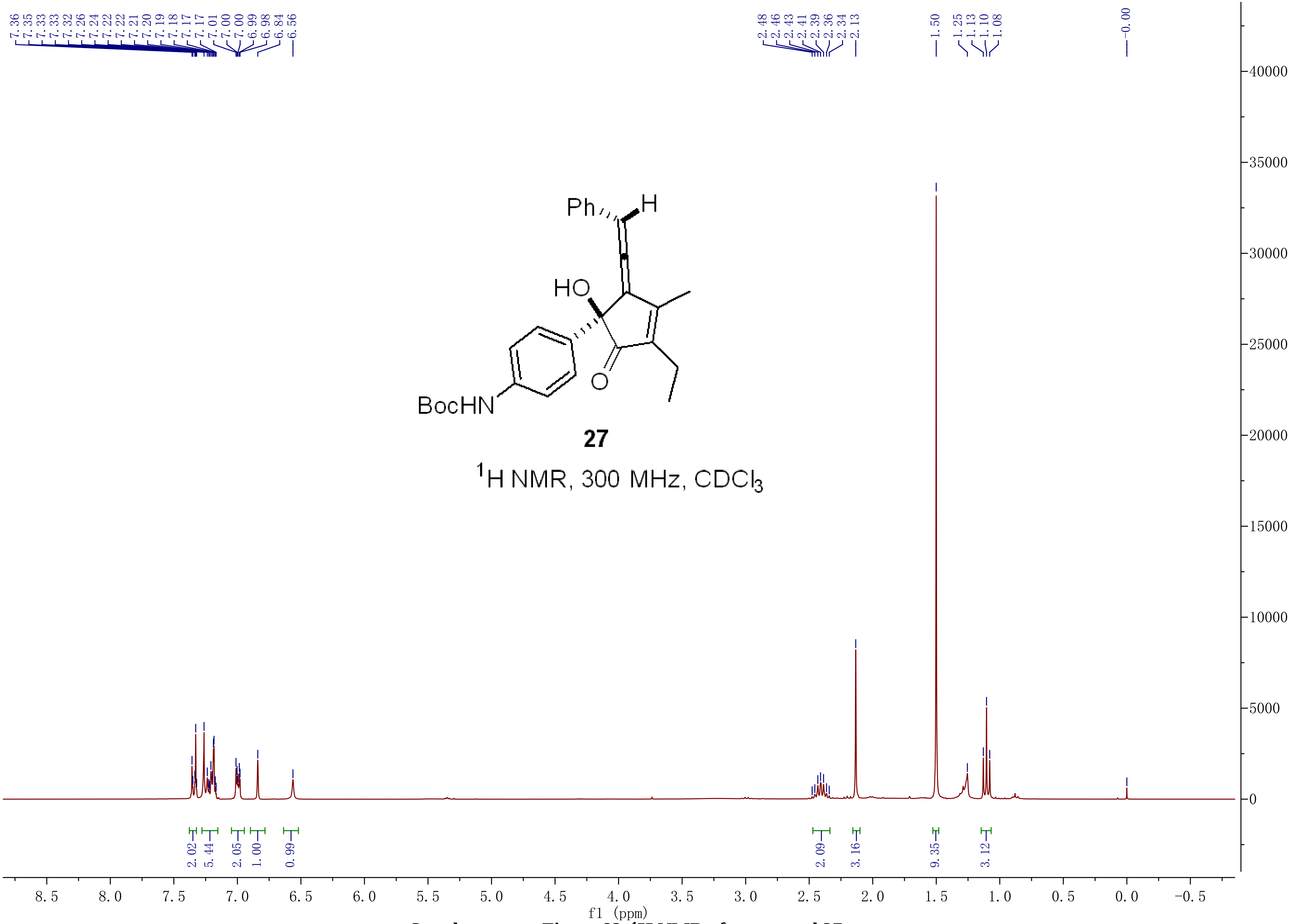

Supplementary Figure 82. ^{1}H NMR of compound 27.

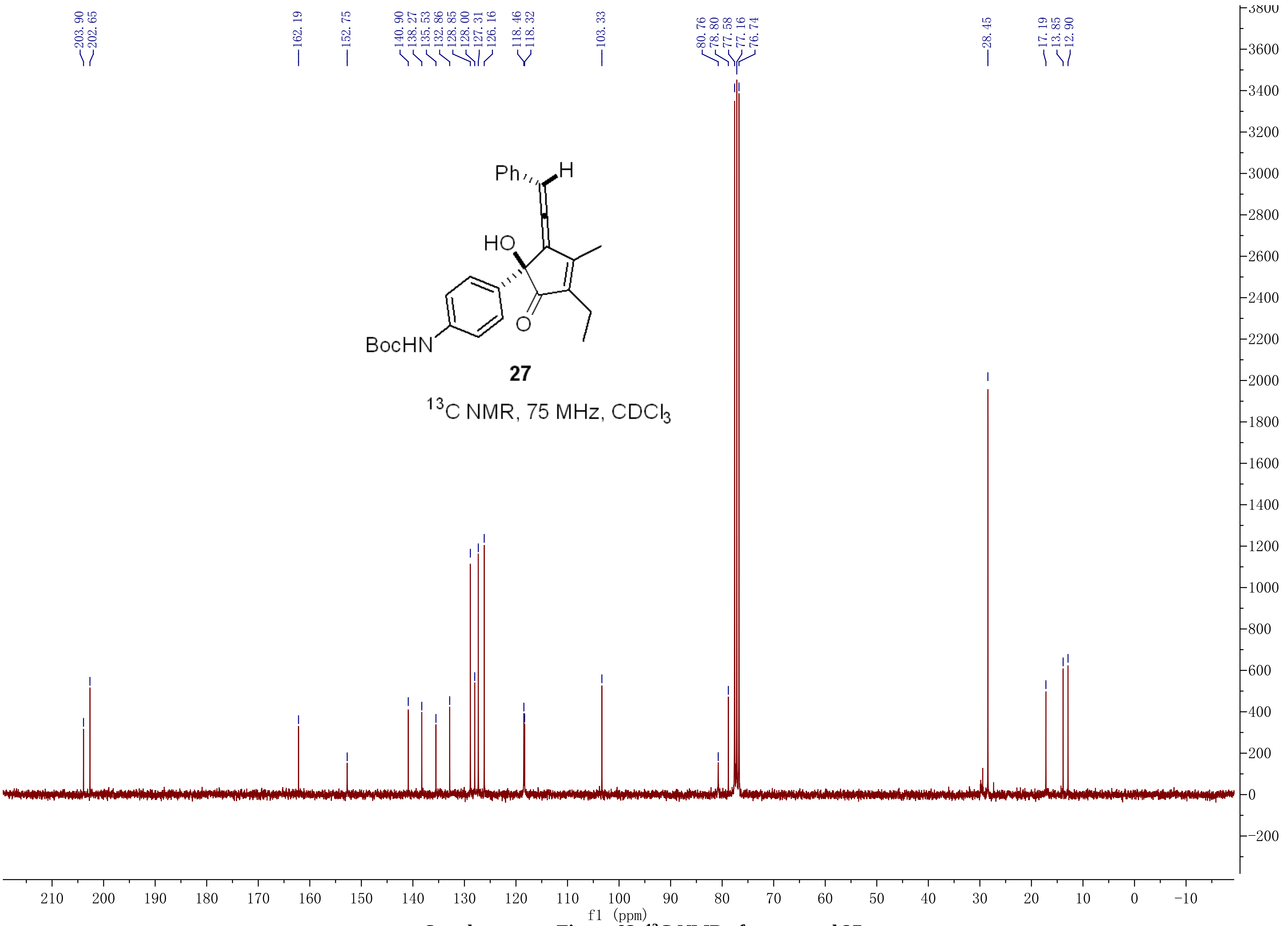

Supplementary Figure 83. ^{13}C NMR of compound 27.

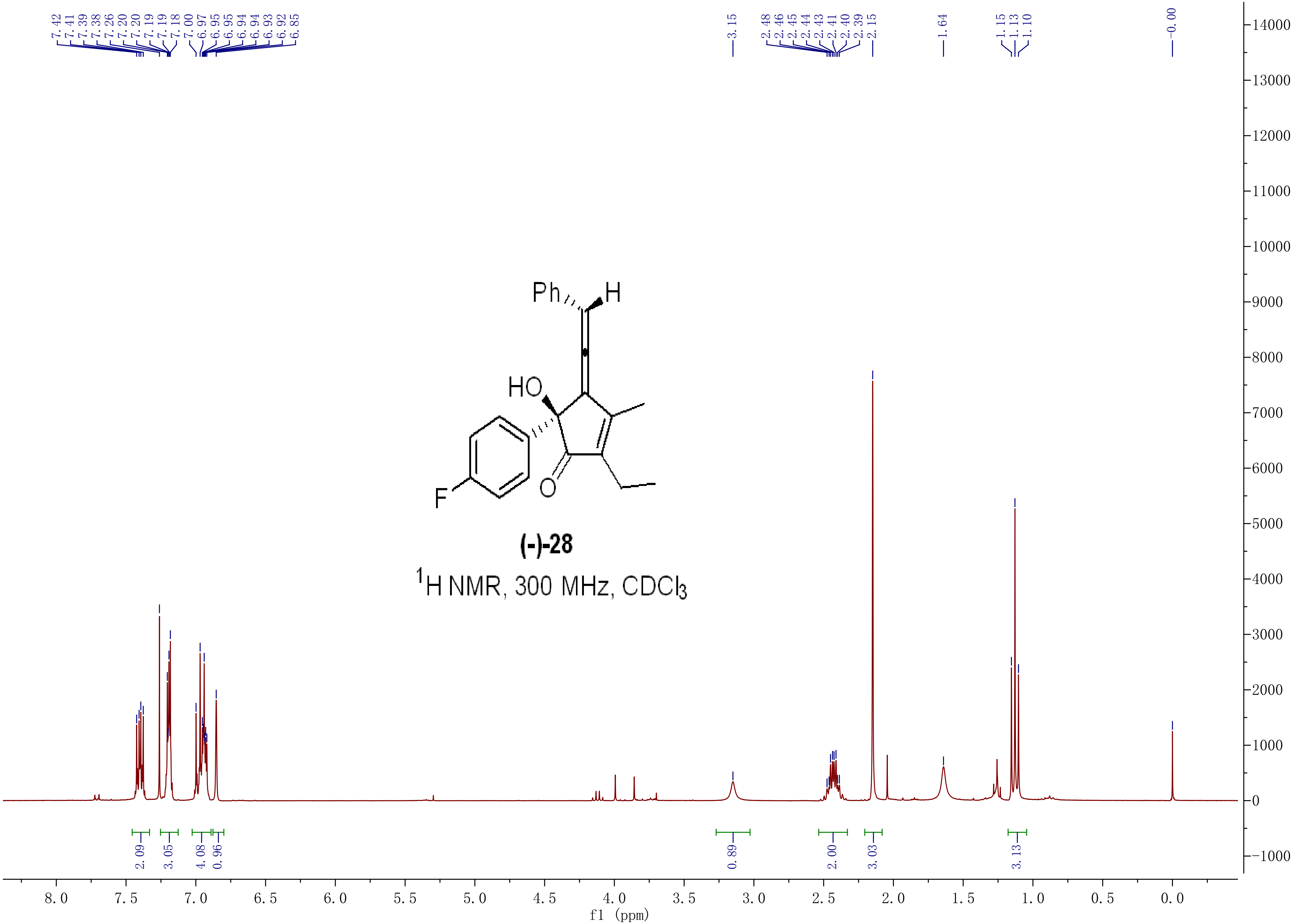

Supplementary Figure 84. ^{1}H NMR of compound (-)-28.

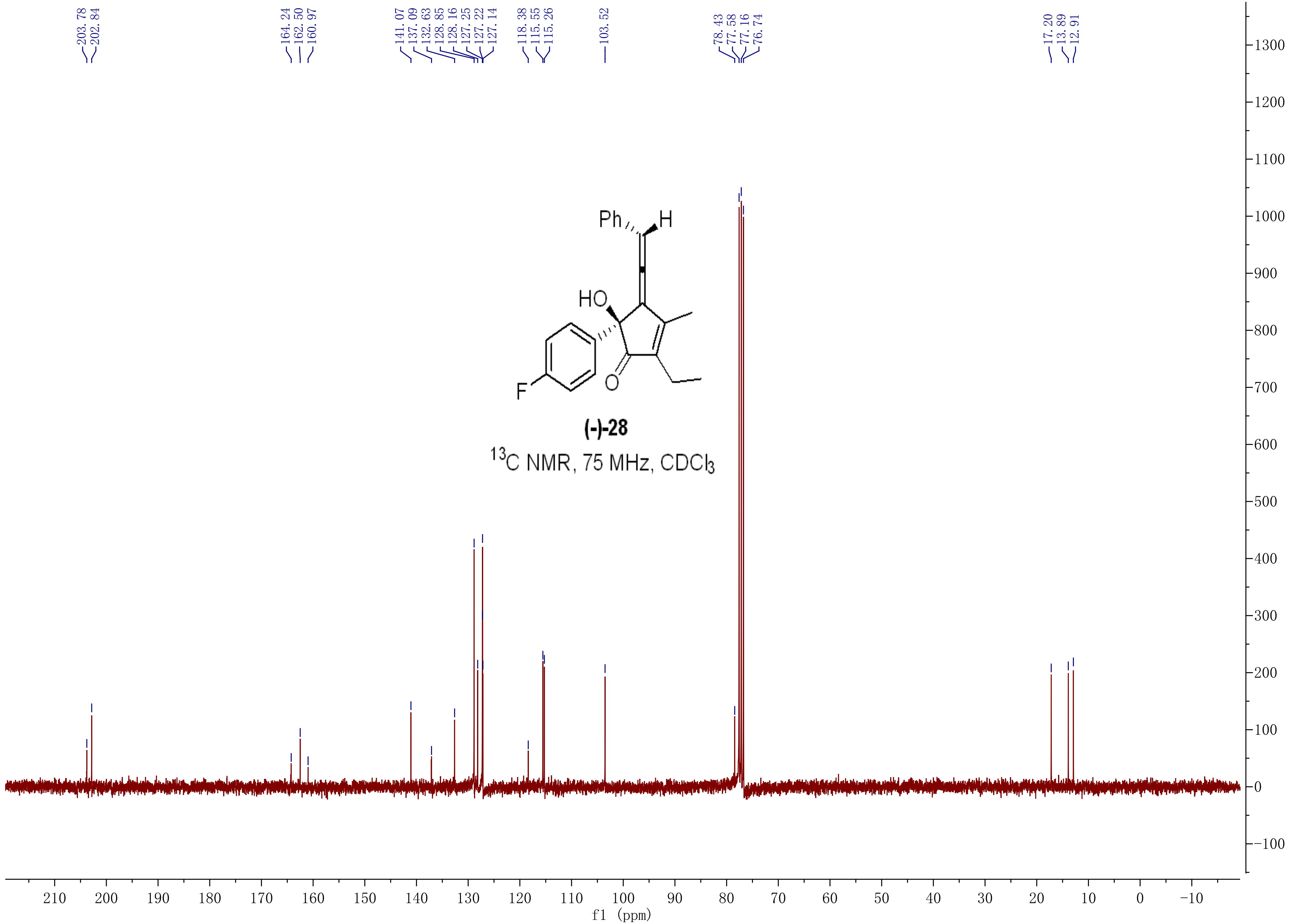

Supplementary Figure 85. ^{13}C NMR of compound (-)-28.

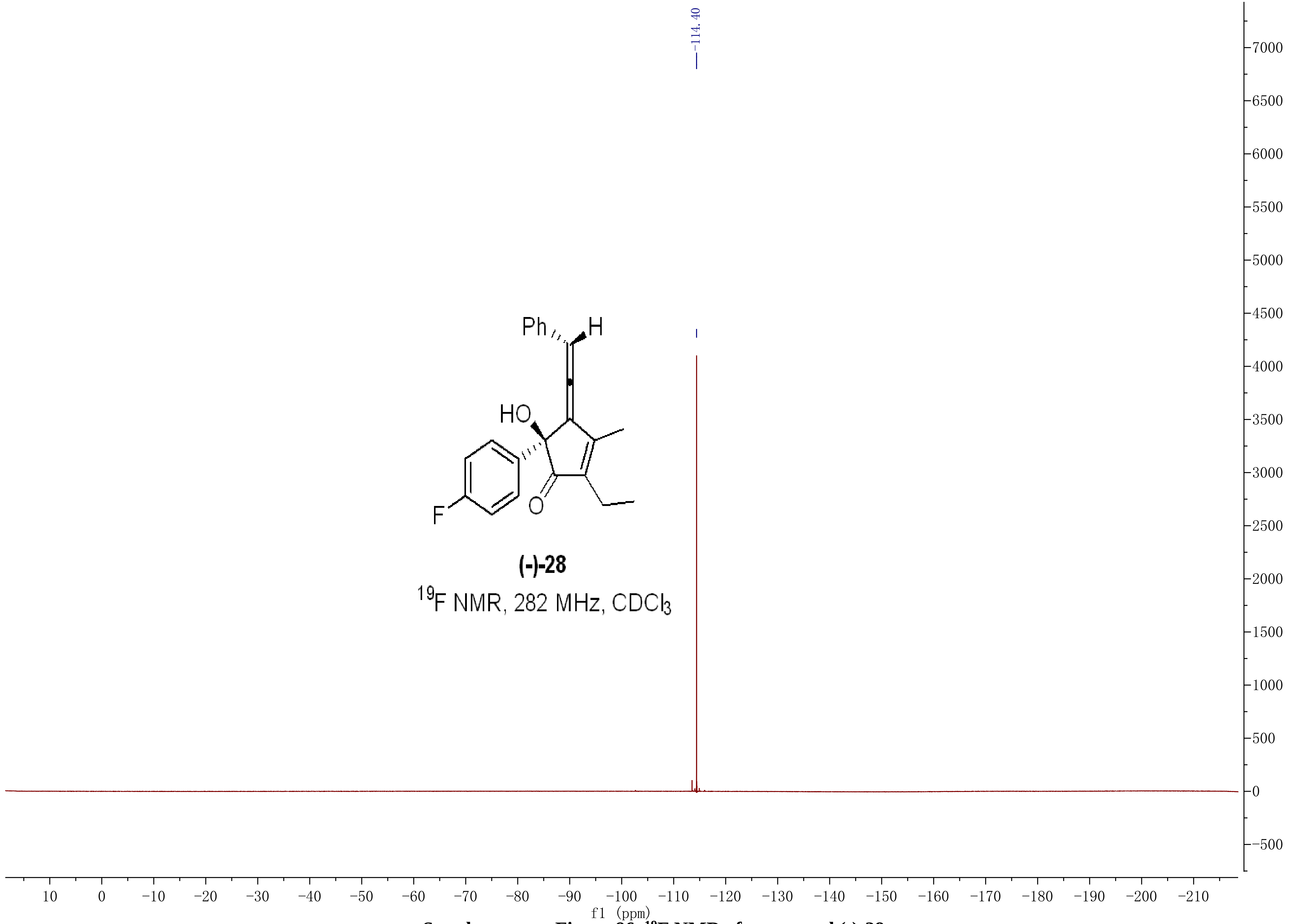

Supplementary Figure 86. ^{19}F NMR of compound (-)-28.

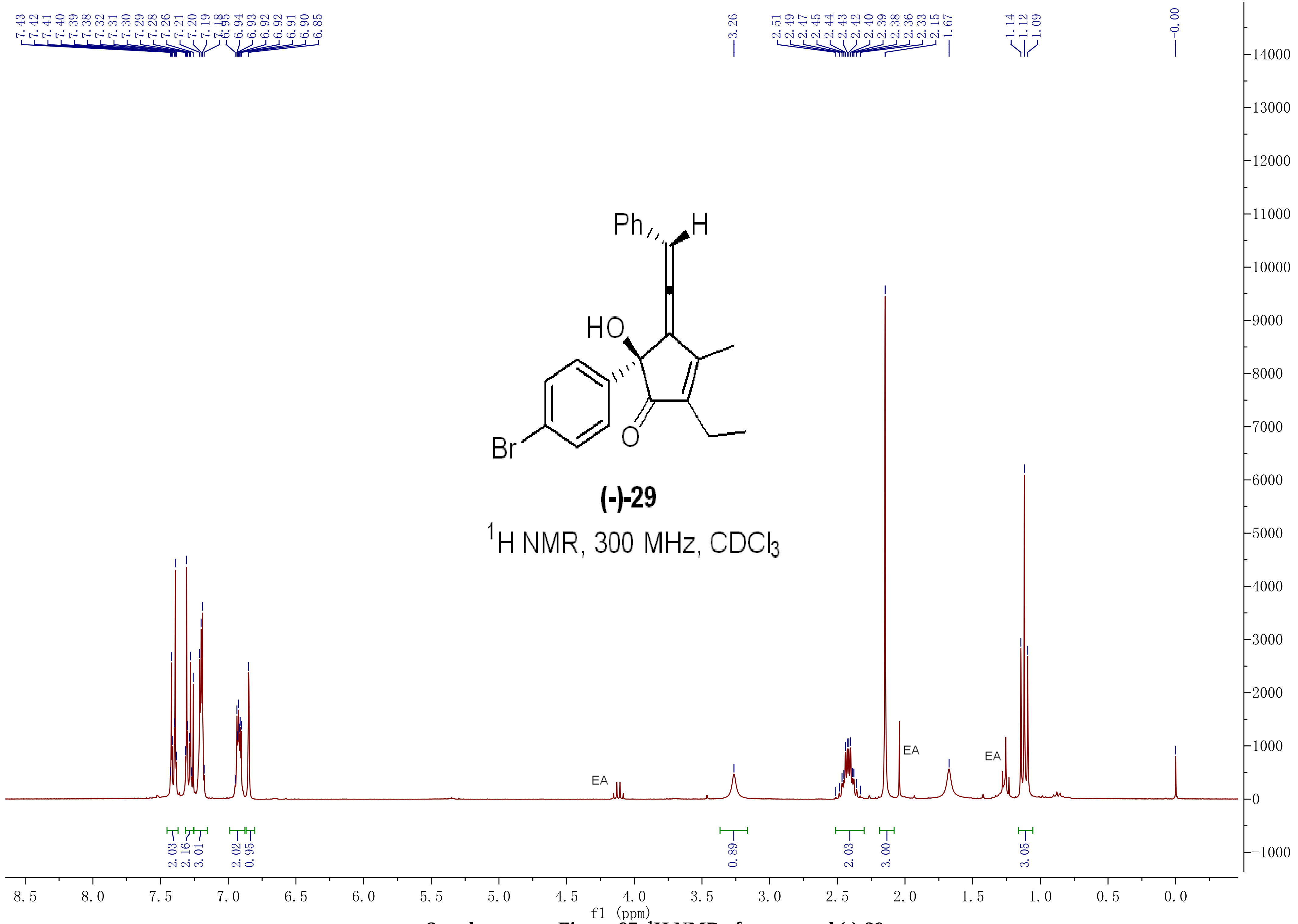

Supplementary Figure 87. ^{1}H NMR of compound (-)-29.

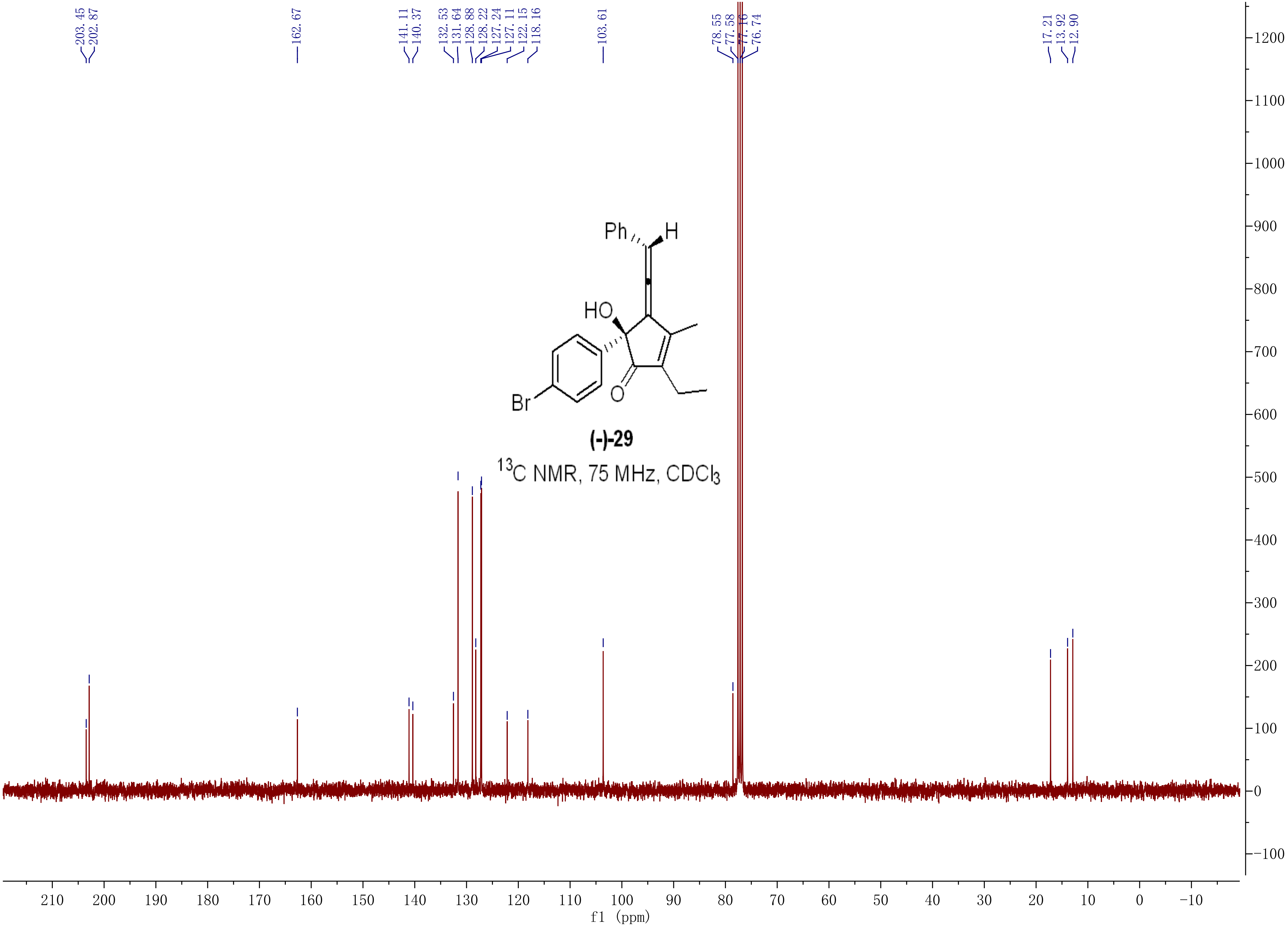

Supplementary Figure 88. ^{13}C NMR of compound (-)-29.

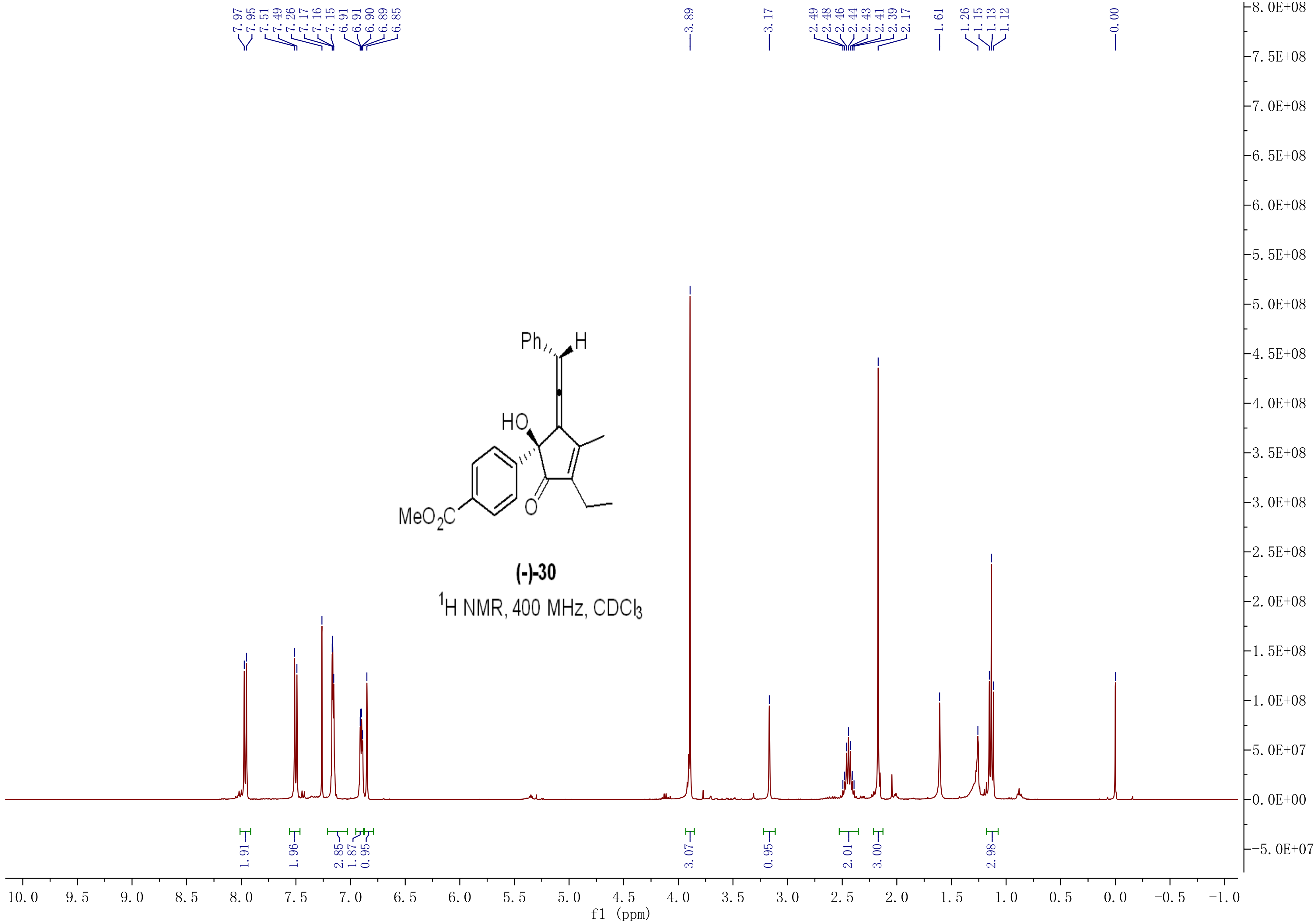

Supplementary Figure 89. ¹H NMR of compound (-)-30.

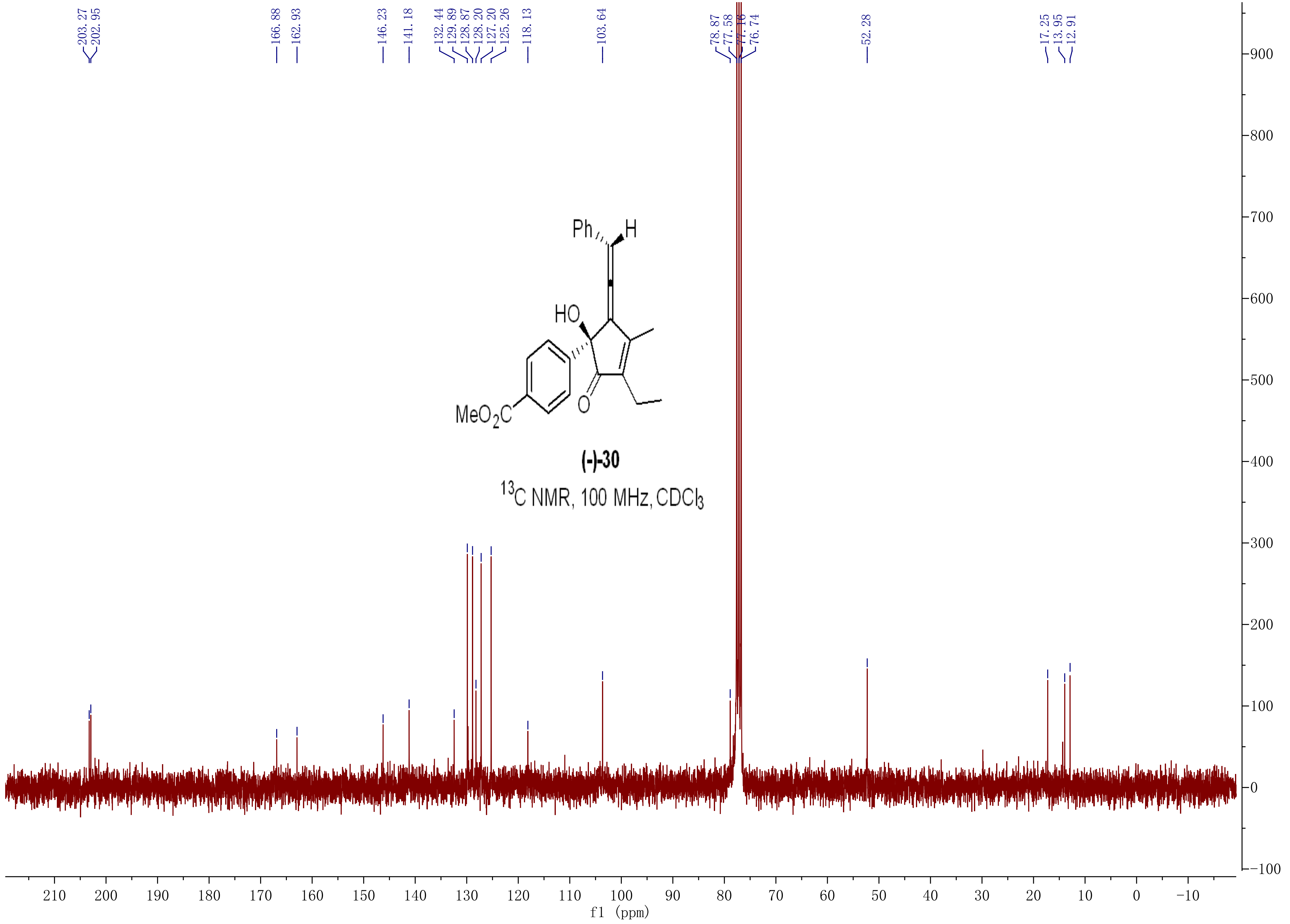

Supplementary Figure 90. ^{13}C NMR of compound (-)-30.

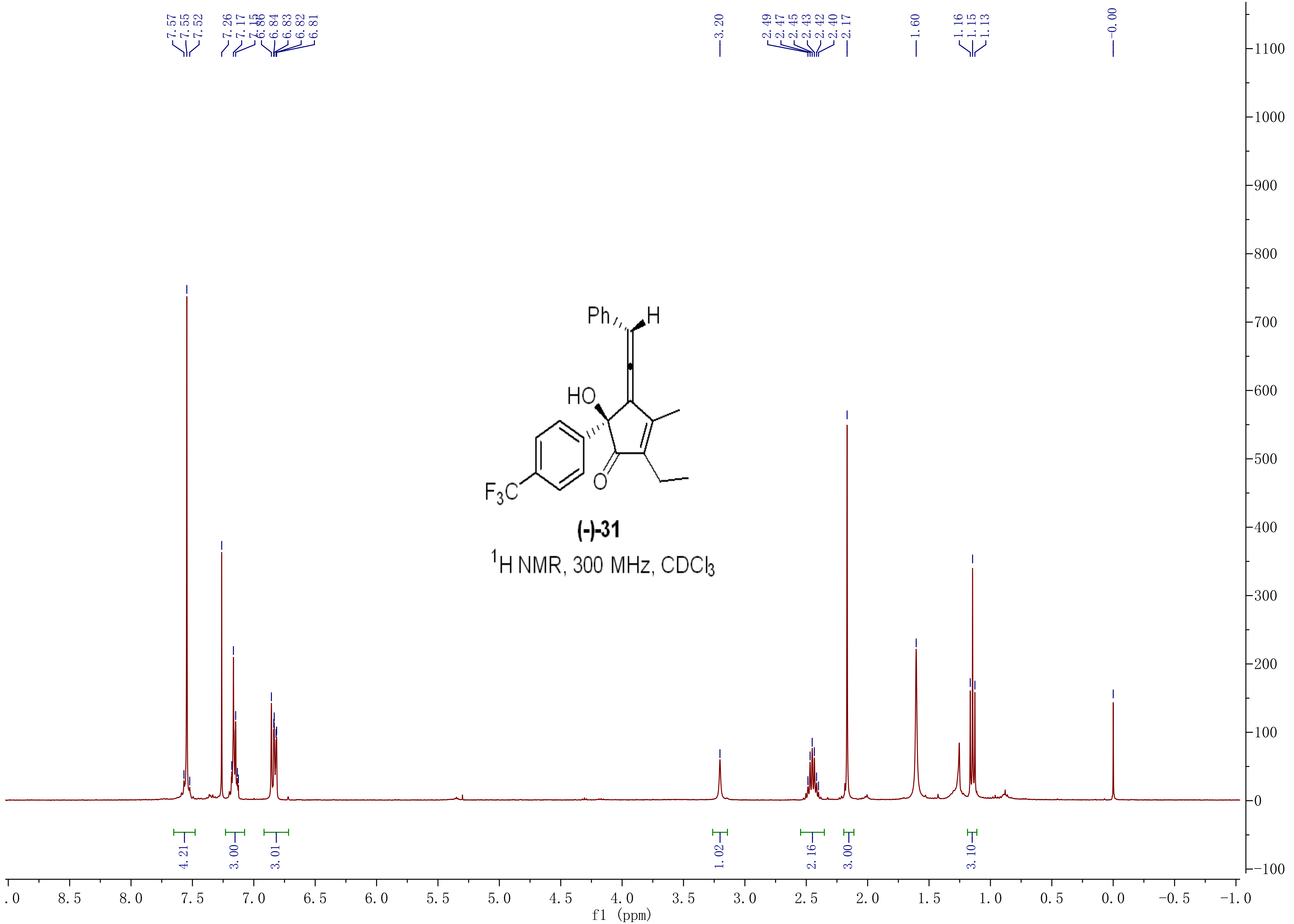

Supplementary Figure 91. ^{1}H NMR of compound (-)-31.

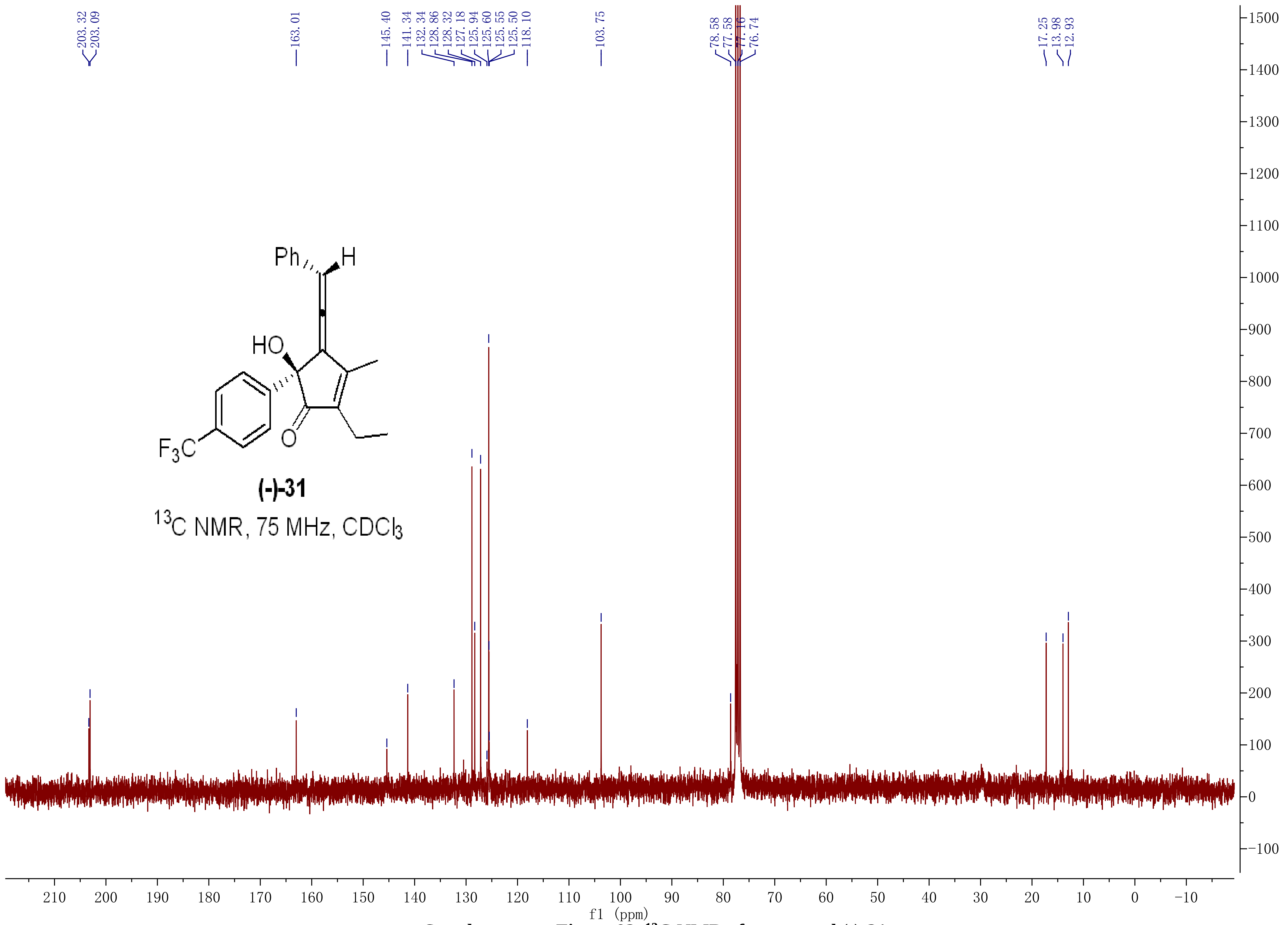

Supplementary Figure 92. ^{13}C NMR of compound (-)-31.

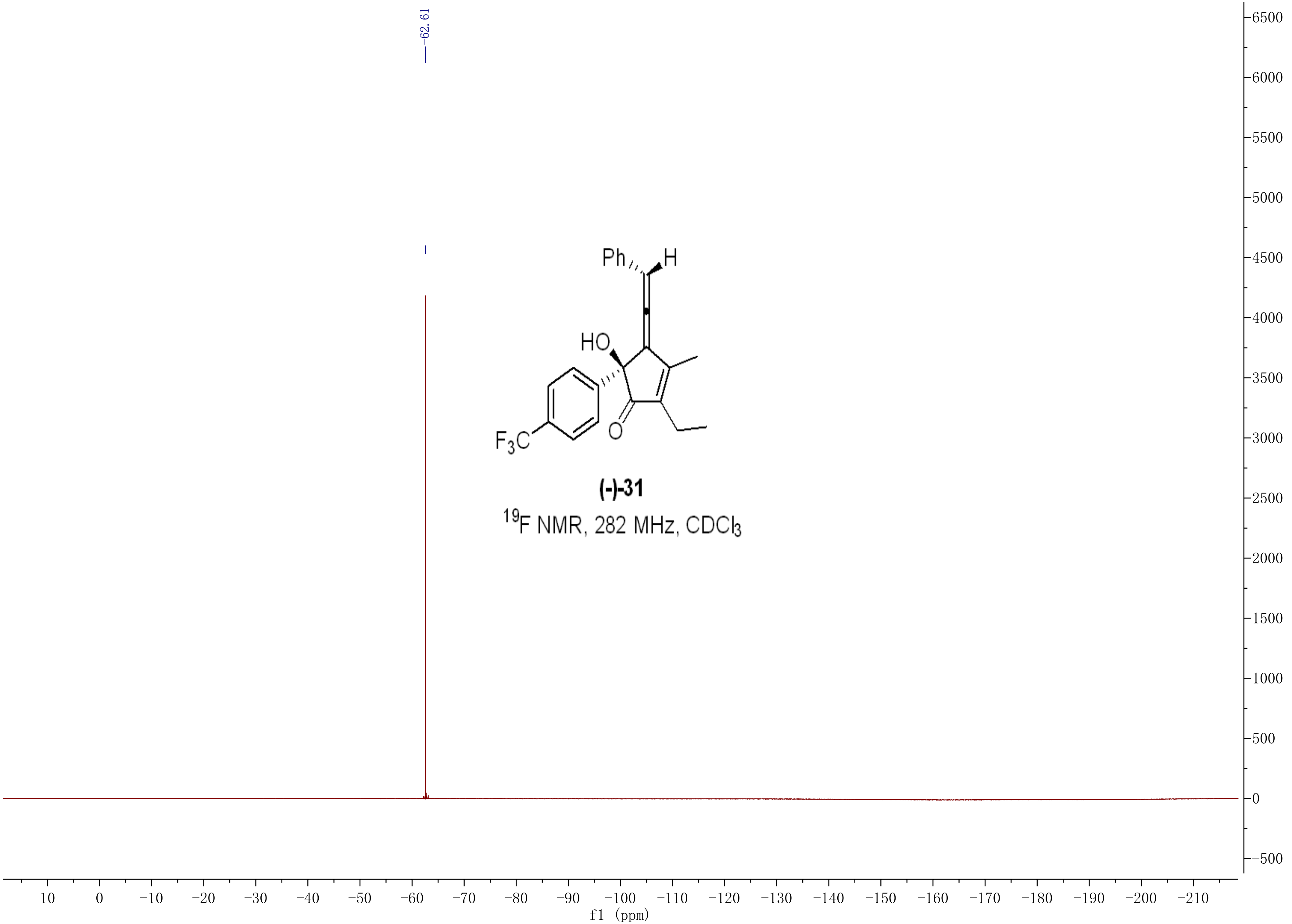

Supplementary Figure 93. ^{19}F NMR of compound (-)-31.

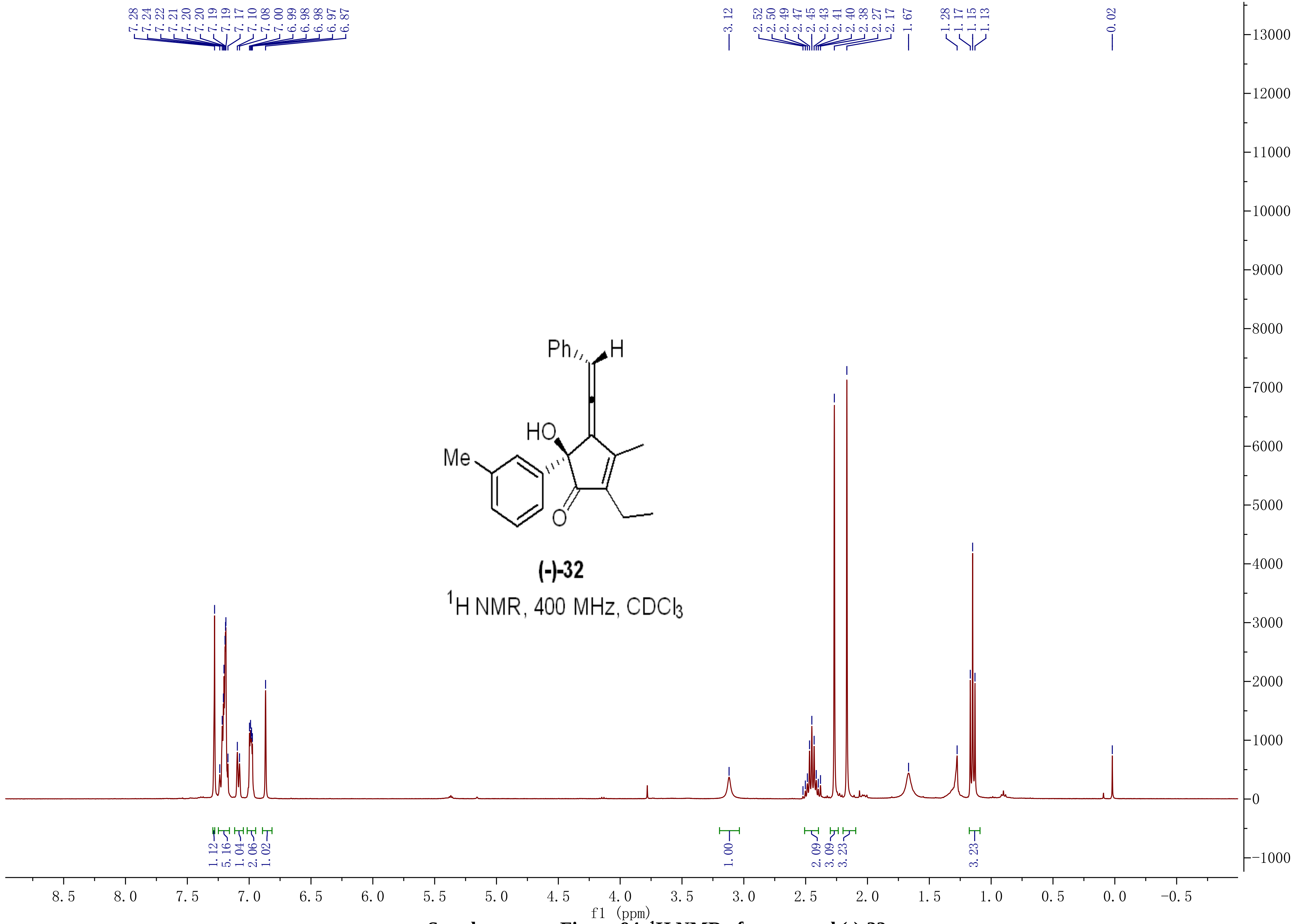

Supplementary Figure 94. ^{1}H NMR of compound (-)-32.

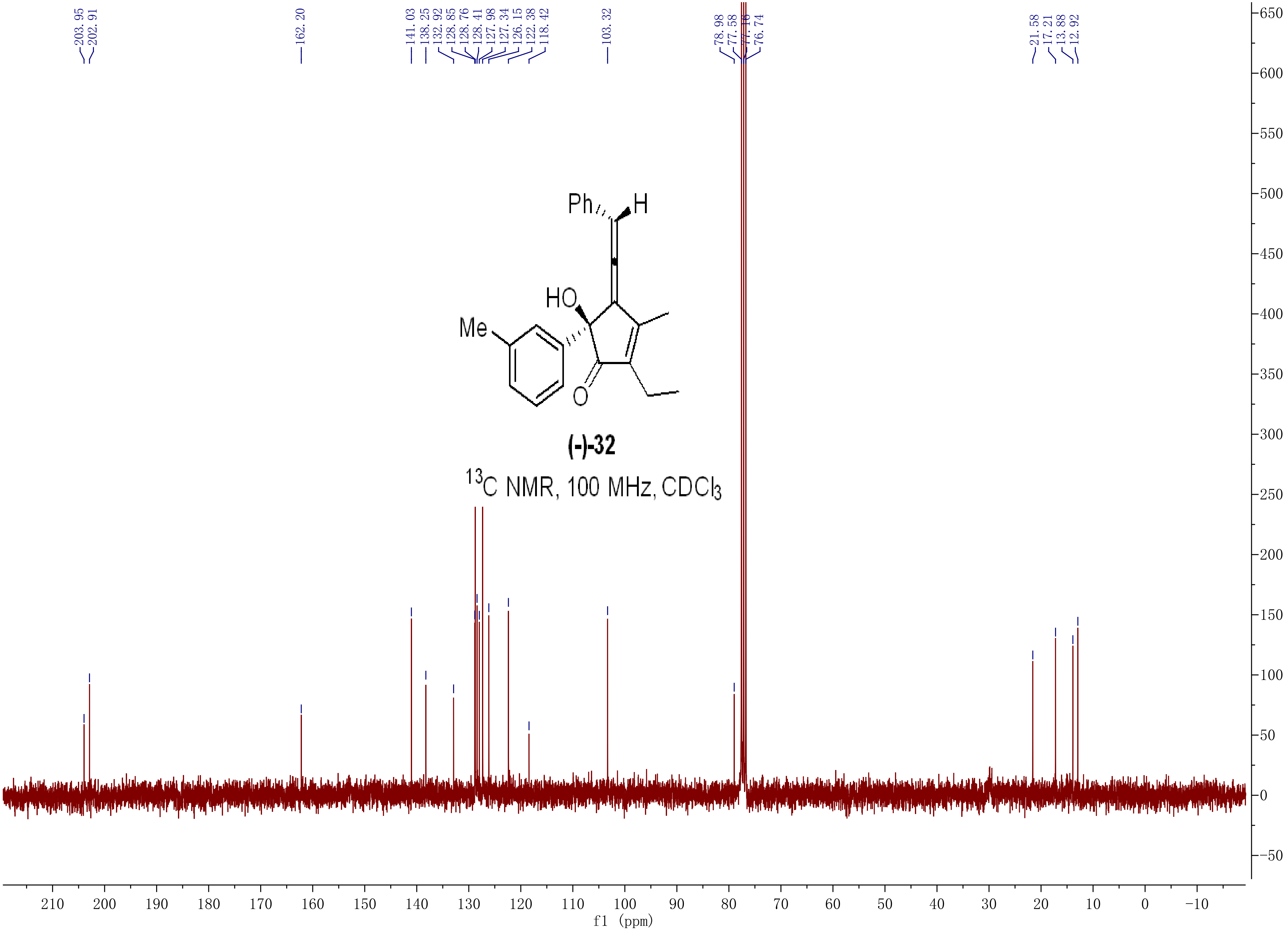

Supplementary Figure 95. ^{13}C NMR of compound (-)-32.

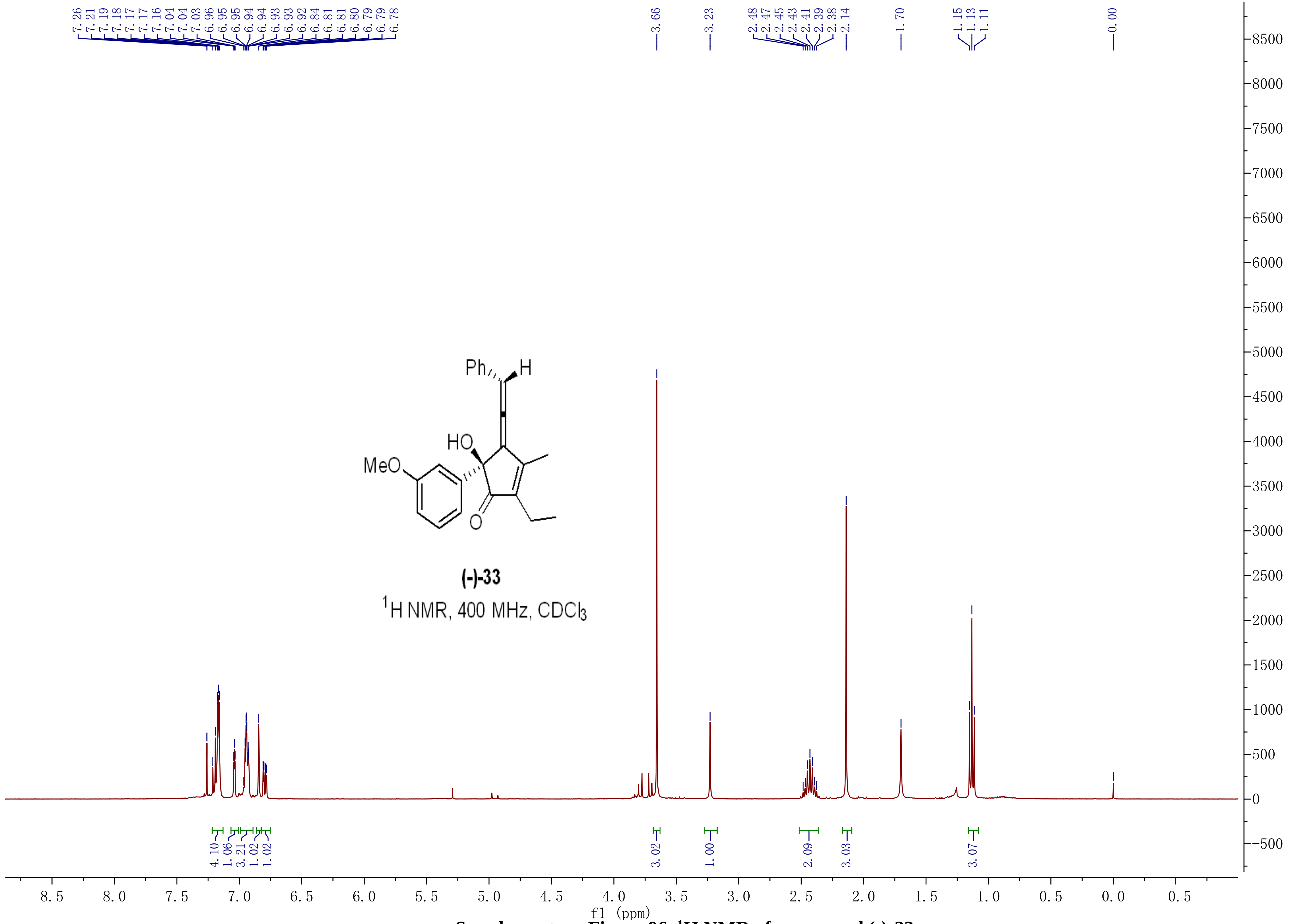

Supplementary Figure 96. ^{1}H NMR of compound (-)-33.

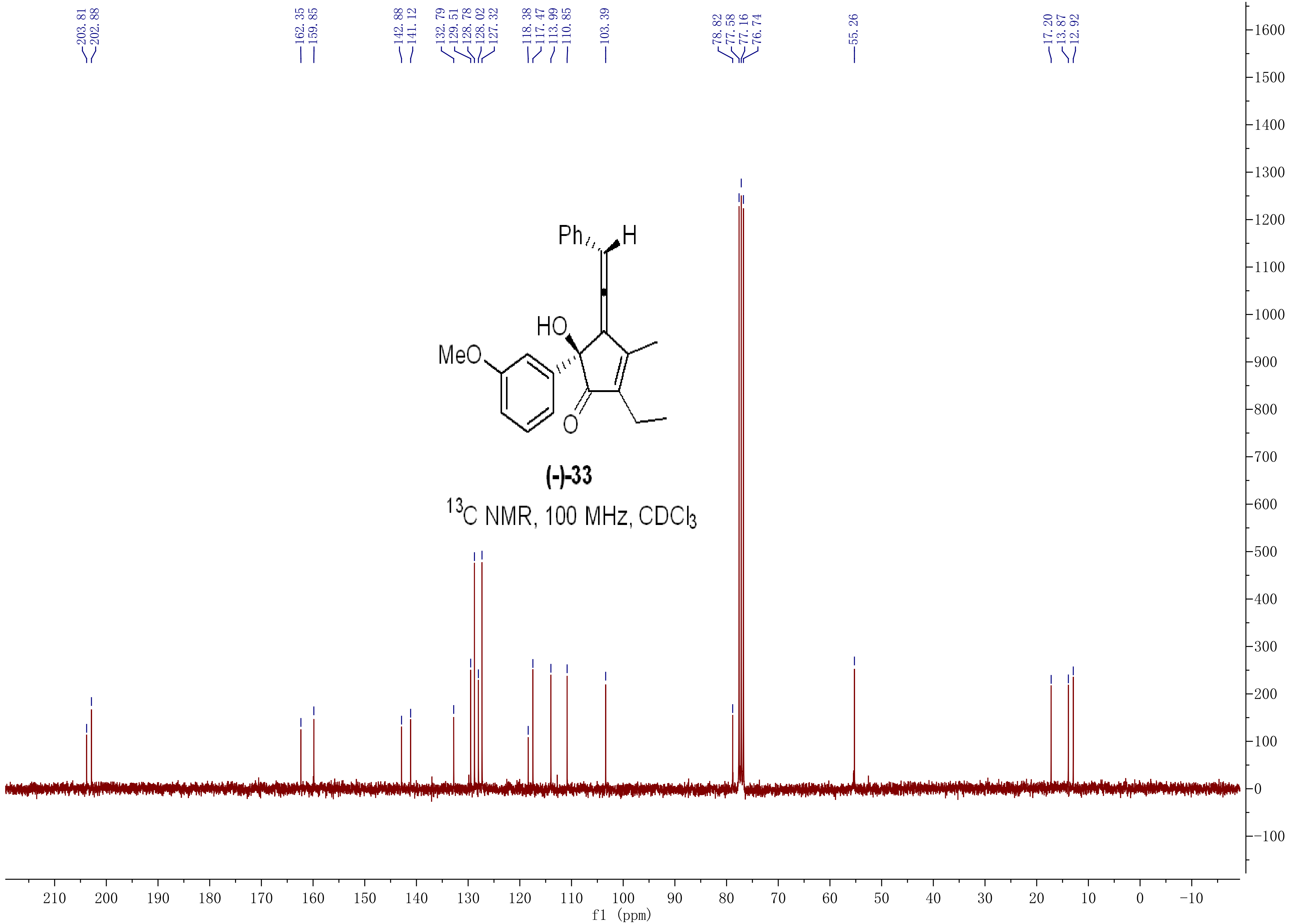

Supplementary Figure 97. ^{13}C NMR of compound (-)-33.

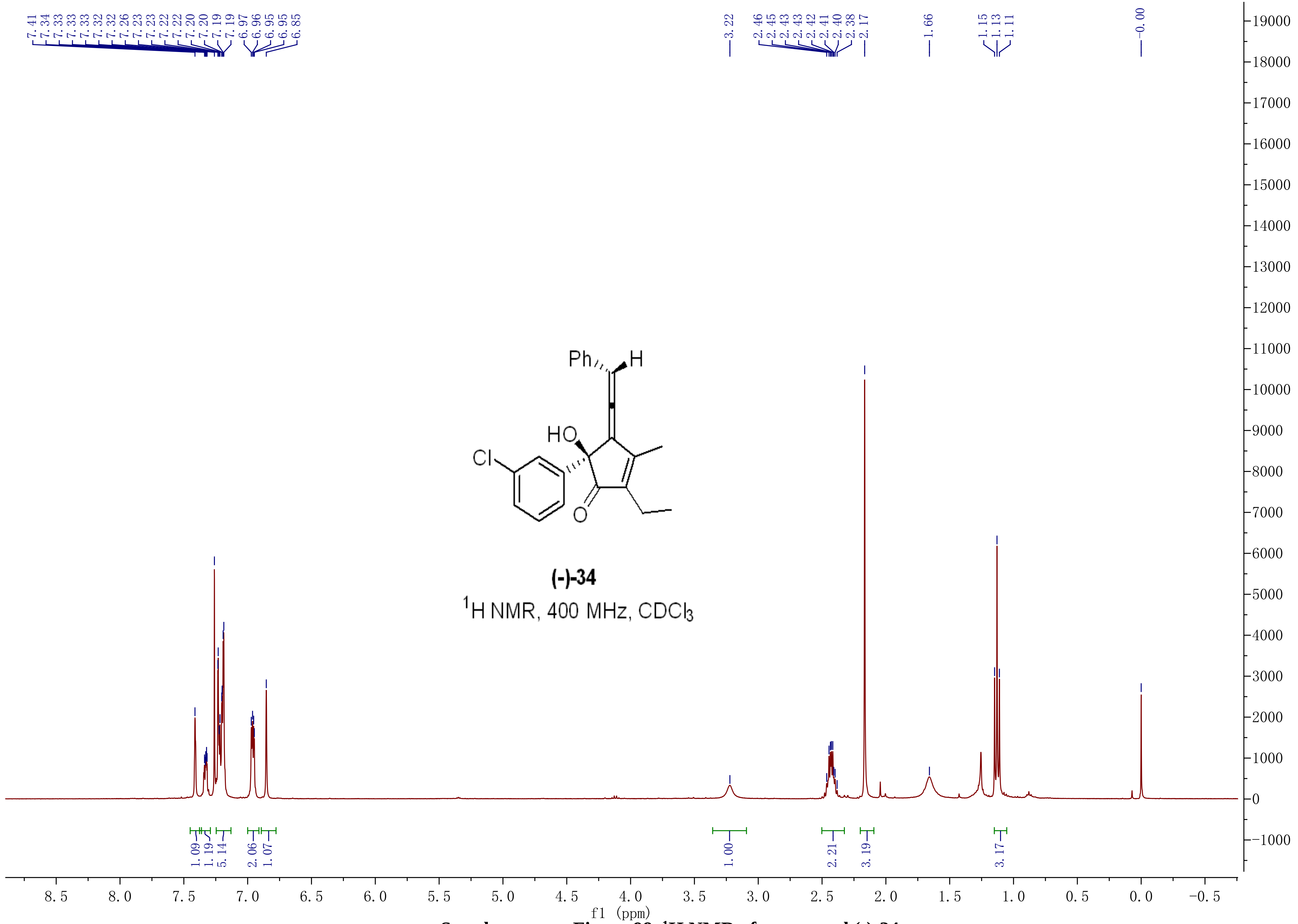

Supplementary Figure 98. ^{1}H NMR of compound (-)-34.

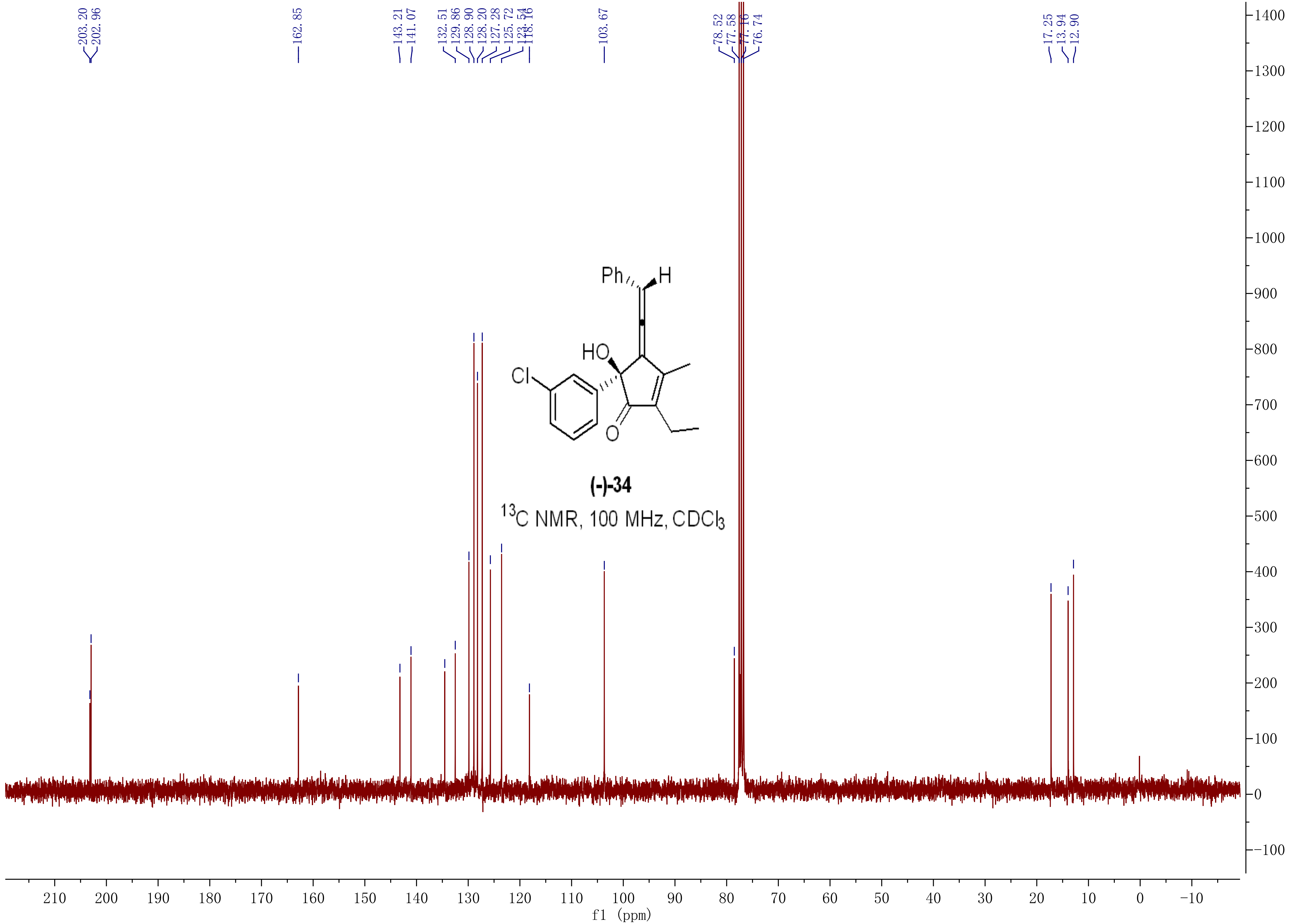

Supplementary Figure 99. ^{13}C NMR of compound (-)-34.

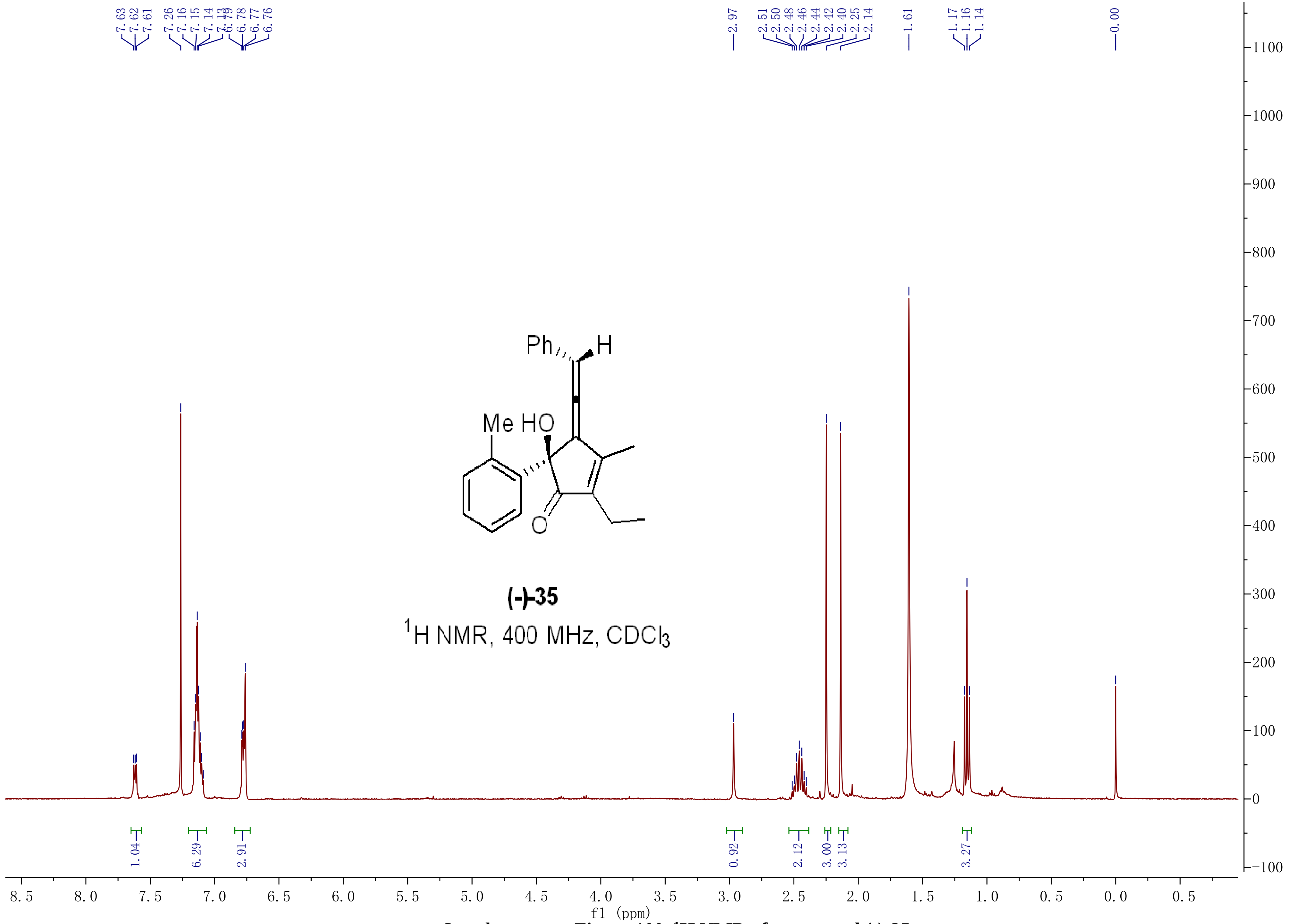

Supplementary Figure 100. ^{1}H NMR of compound (-)-35.

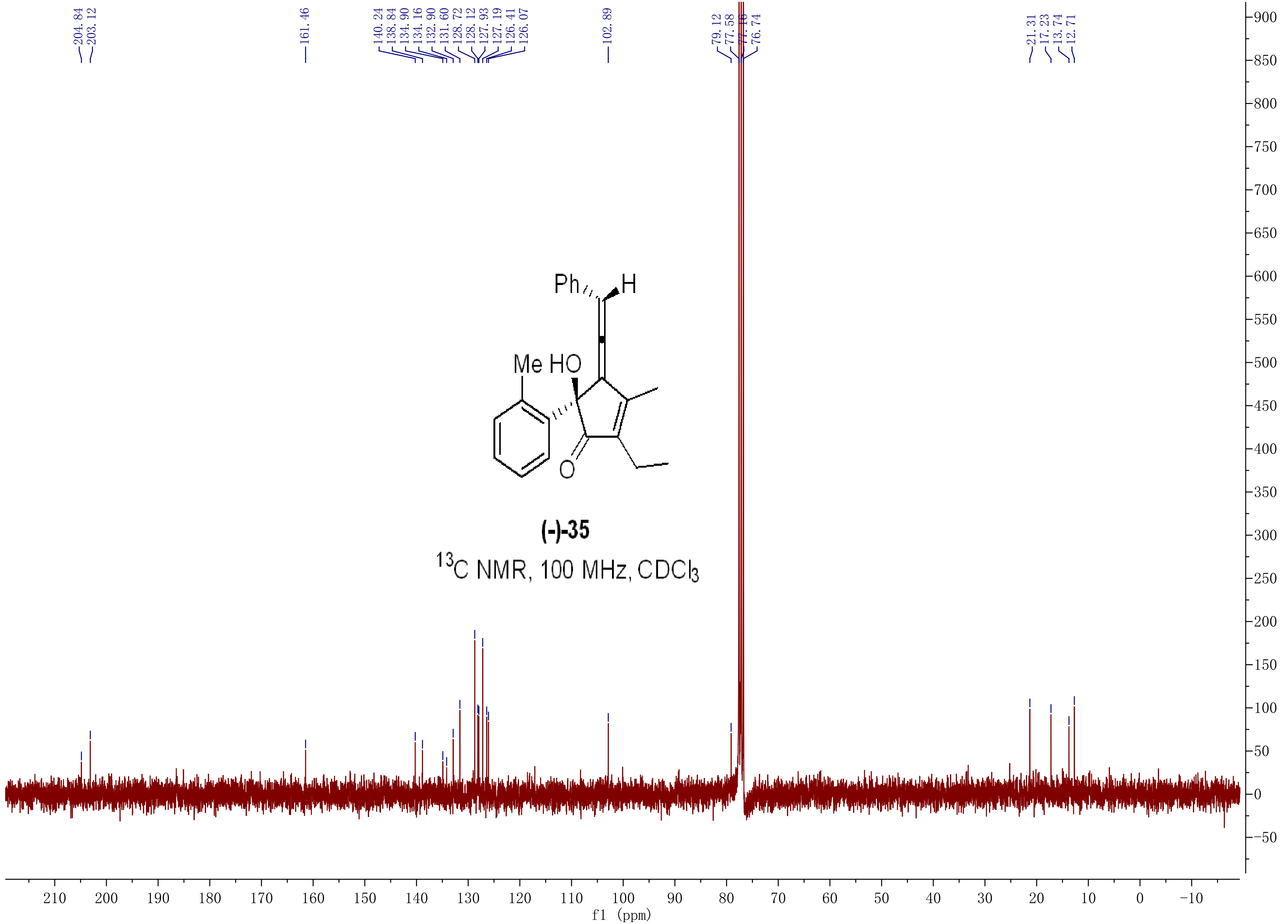

Supplementary Figure 101. ^{13}C NMR of compound (-)-35.

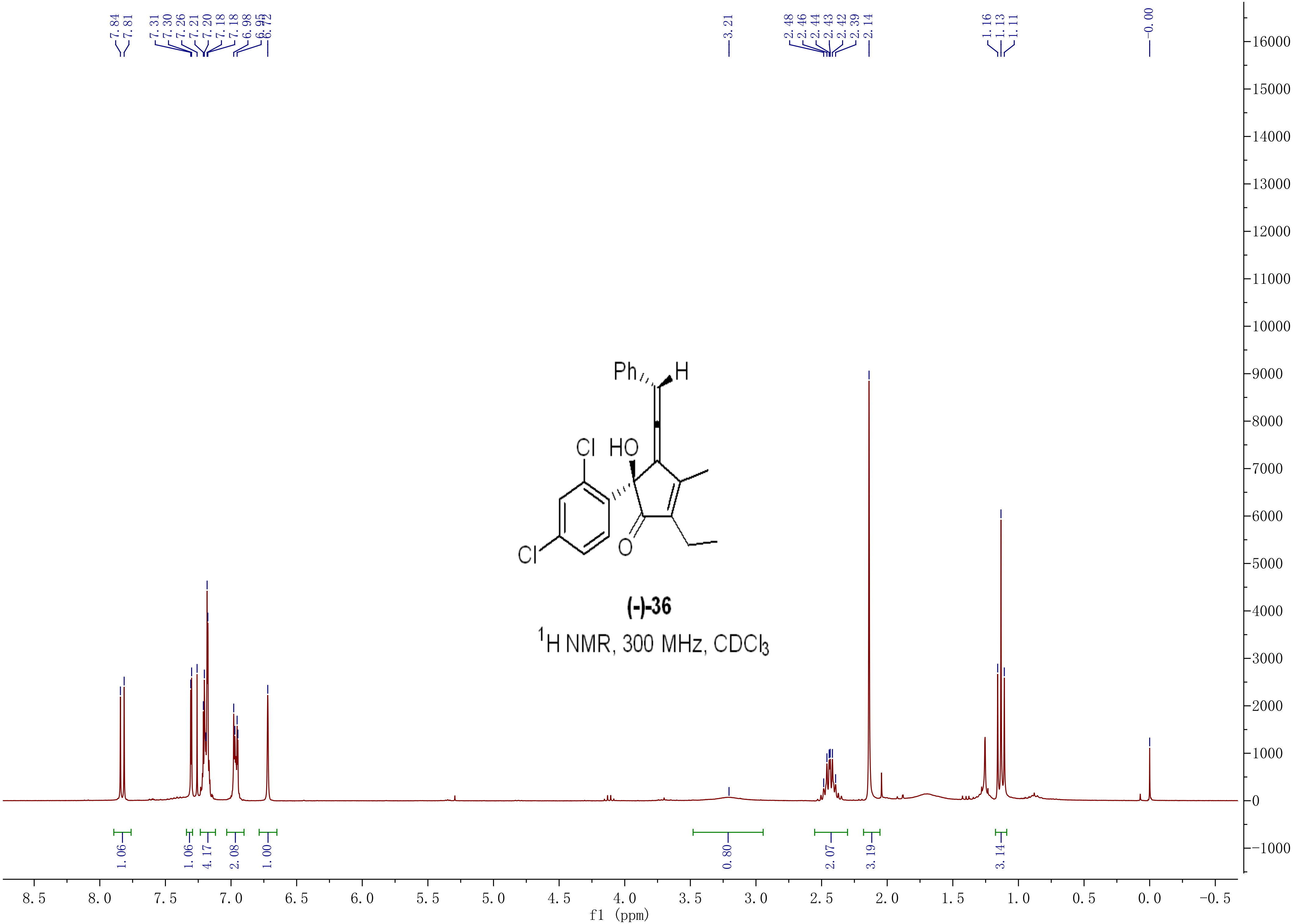

Supplementary Figure 102. ^{13}C NMR of compound (-)-36.

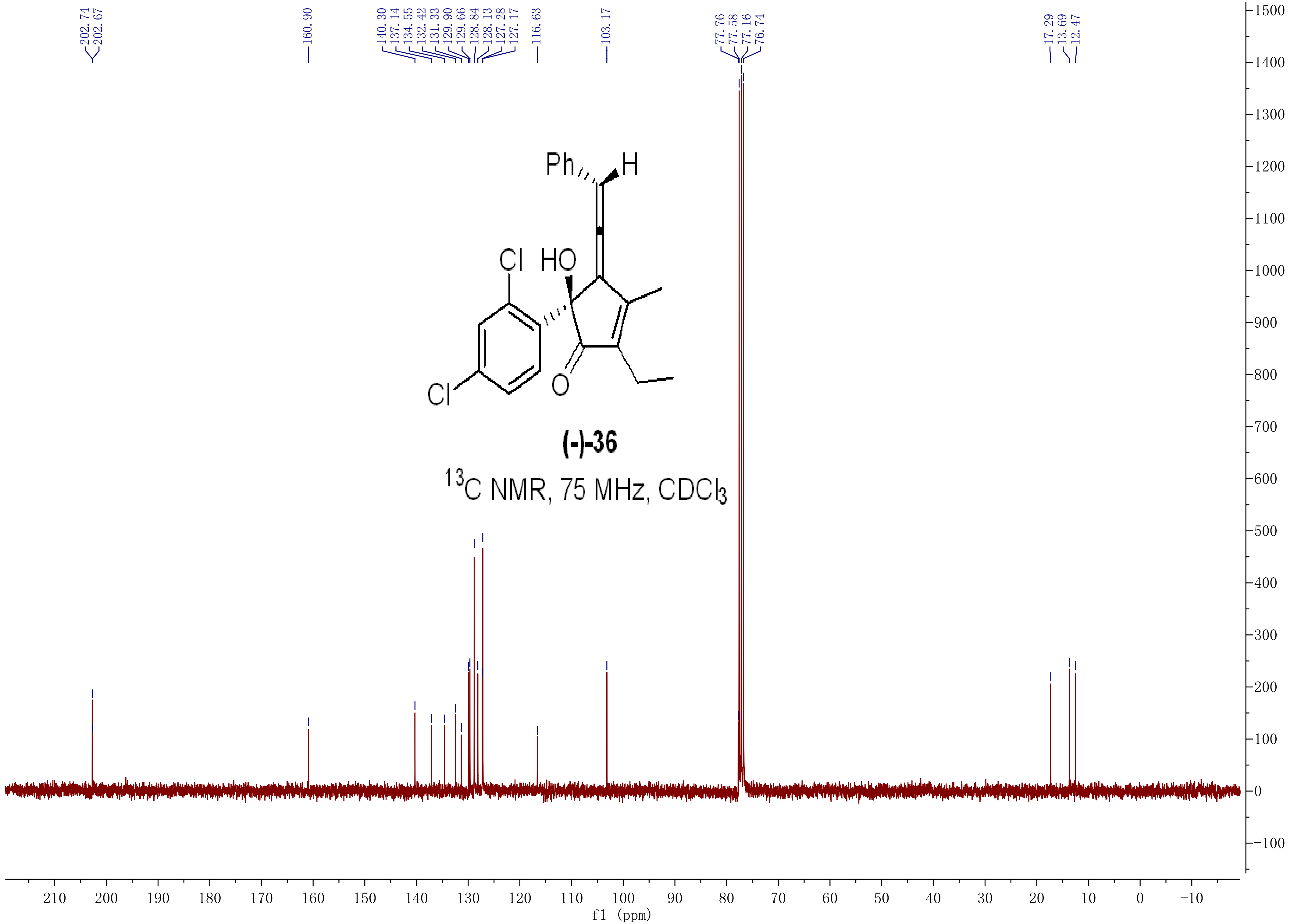

Supplementary Figure 103. ^{13}C NMR of compound (-)-36.

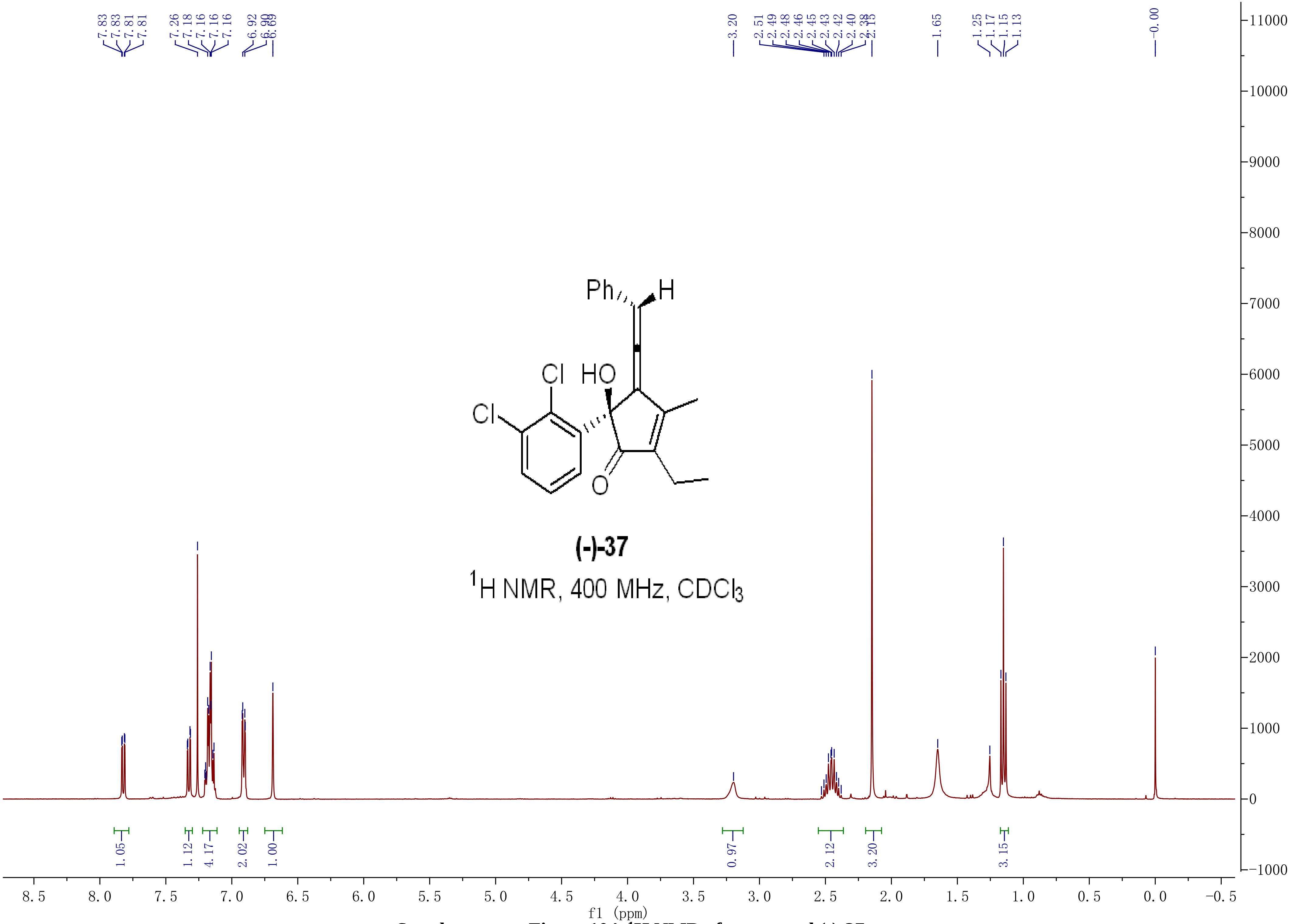

Supplementary Figure 104. ^{1}H NMR of compound (-)-37.

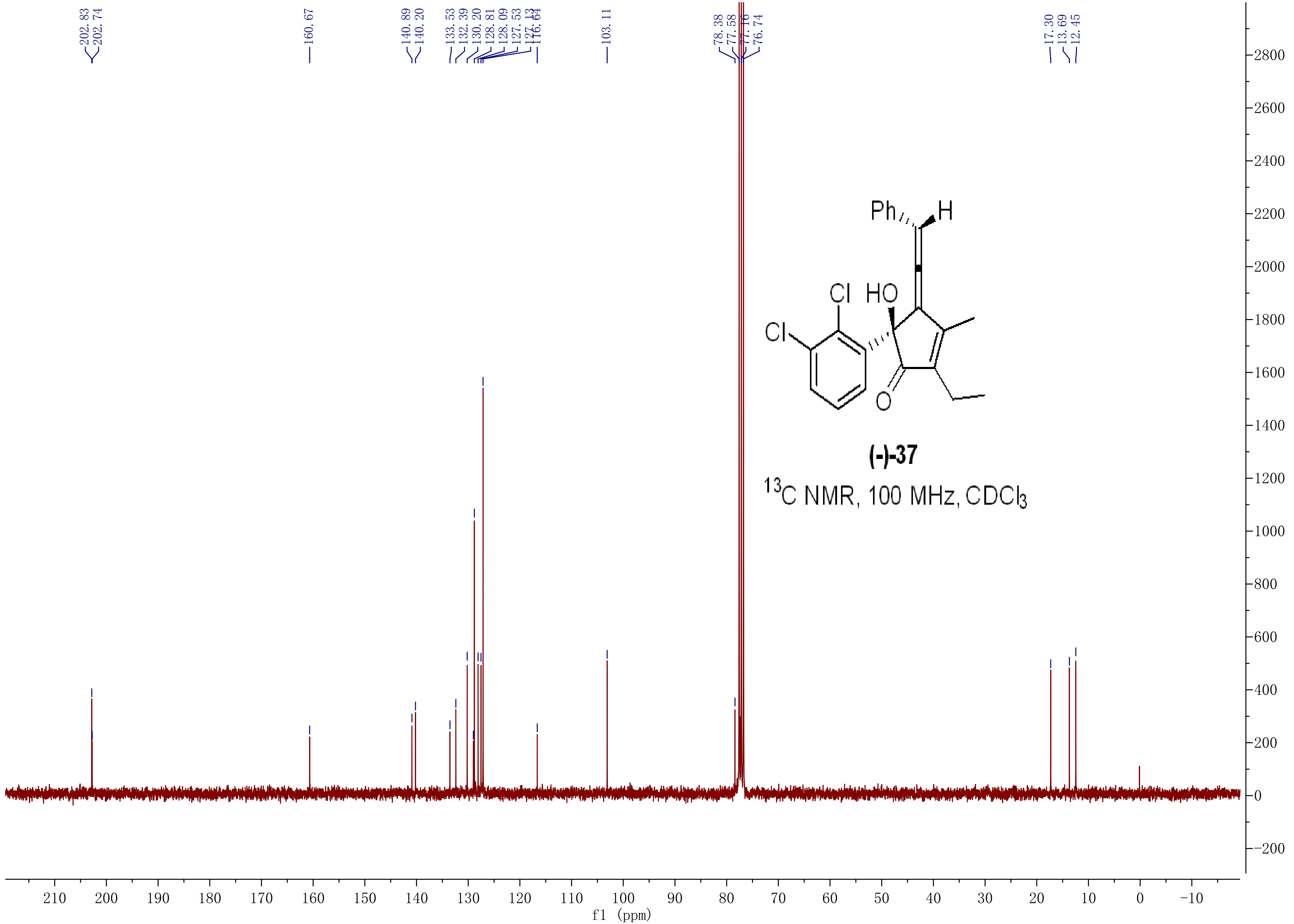

Supplementary Figure 105. ^{13}C NMR of compound (-)-37.

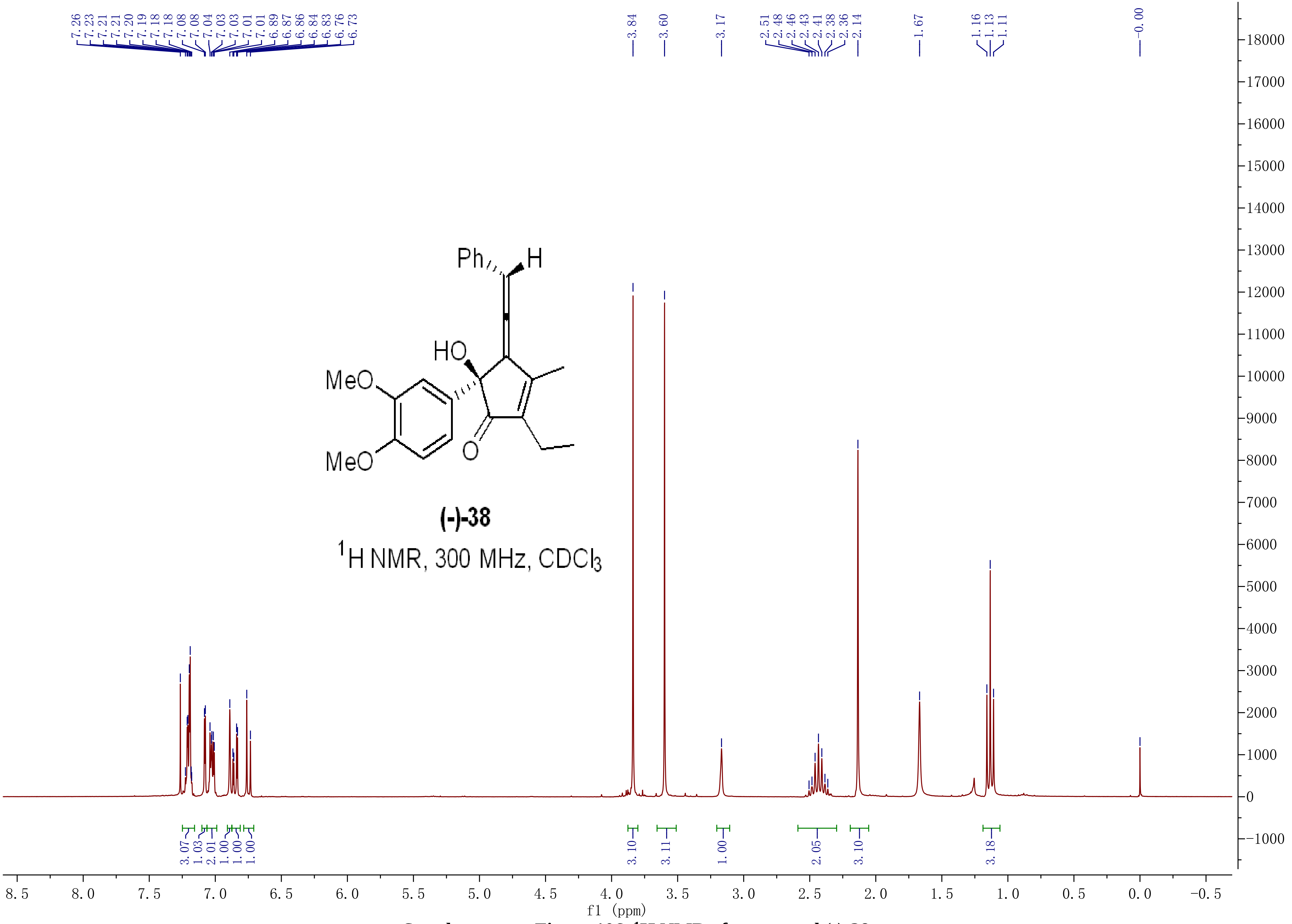

Supplementary Figure 106. ^{1}H NMR of compound (-)-38.

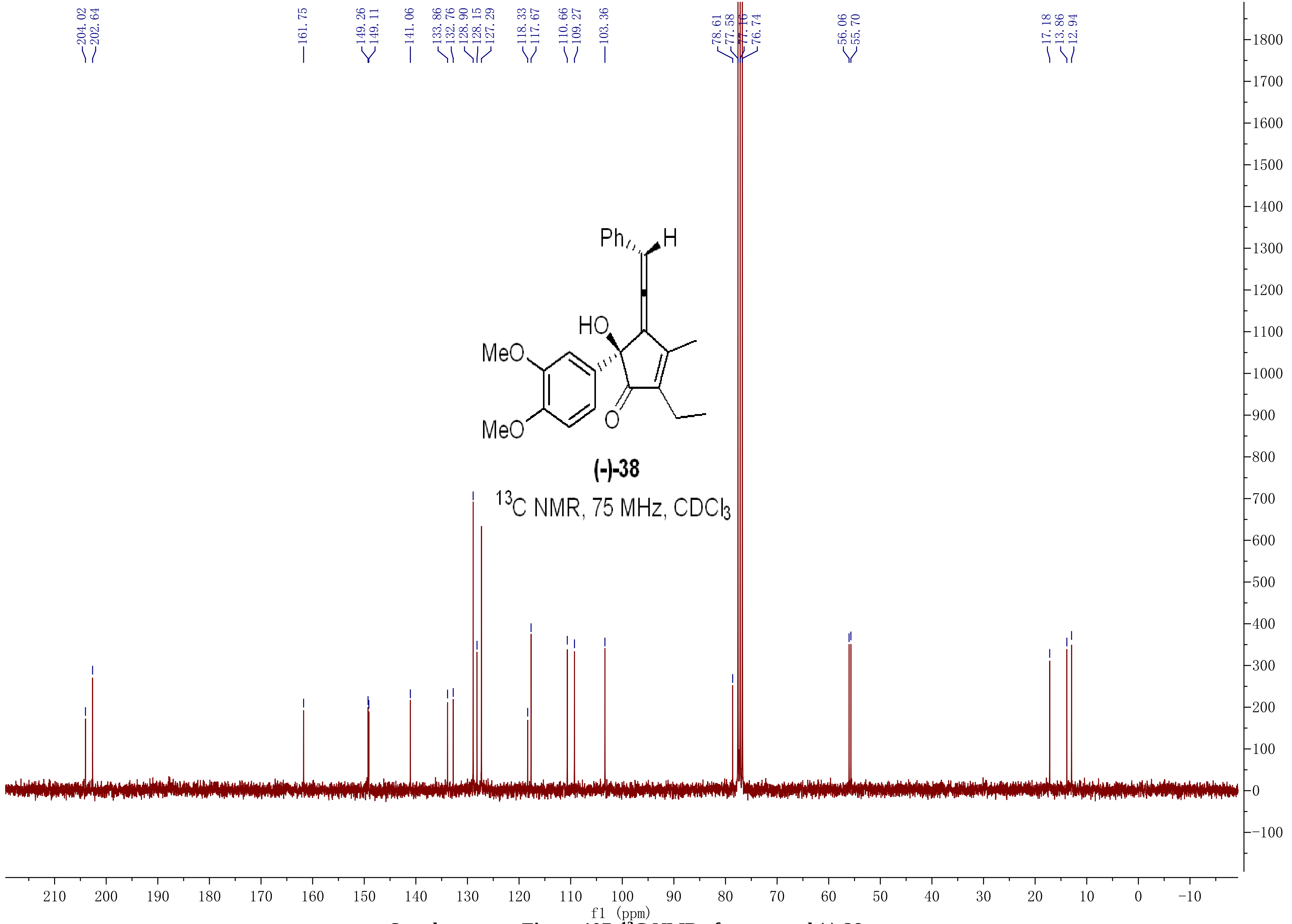

Supplementary Figure 107. ^{13}C NMR of compound (-)-38.

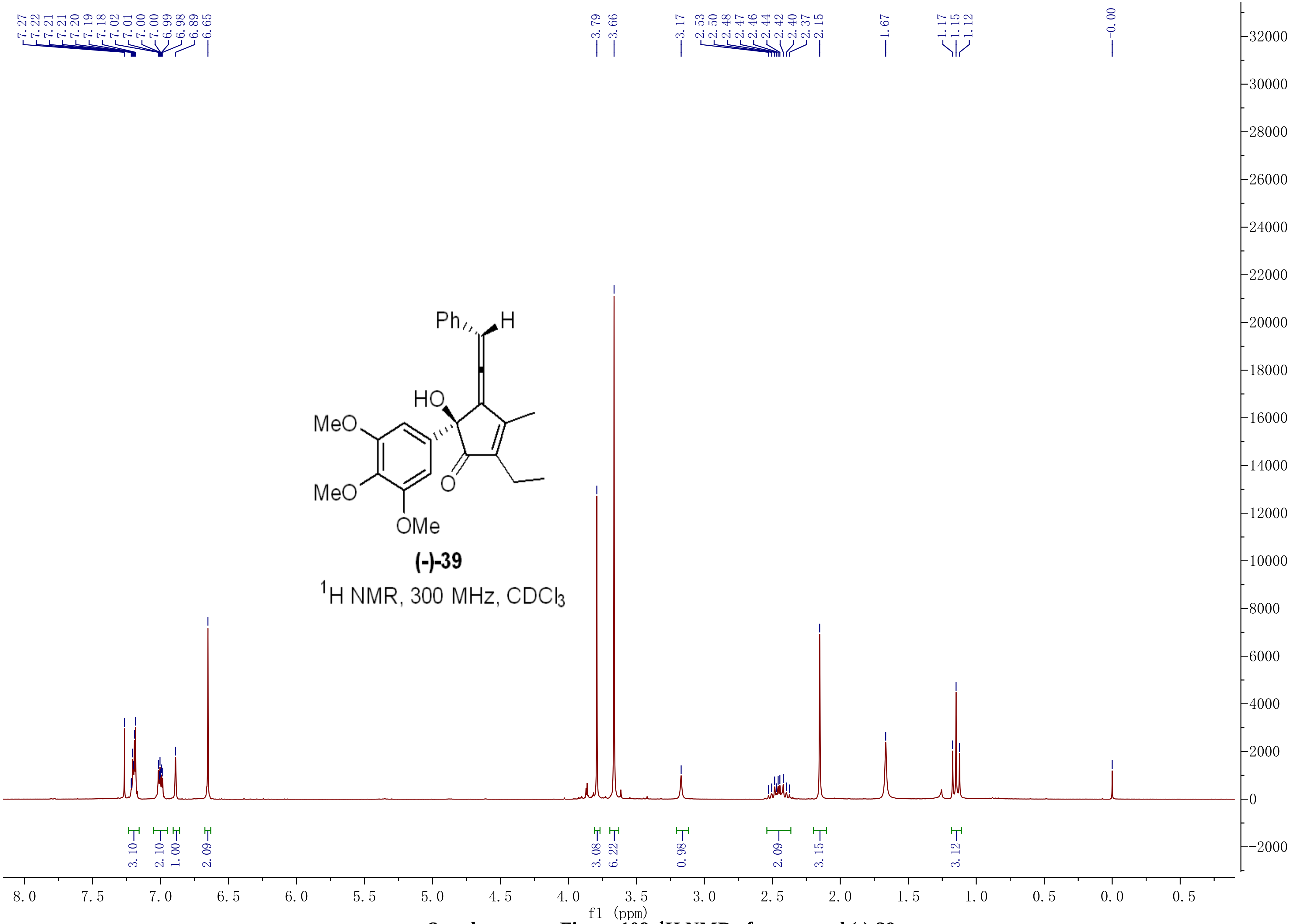

Supplementary Figure 108. ^{1}H NMR of compound (-)-39.

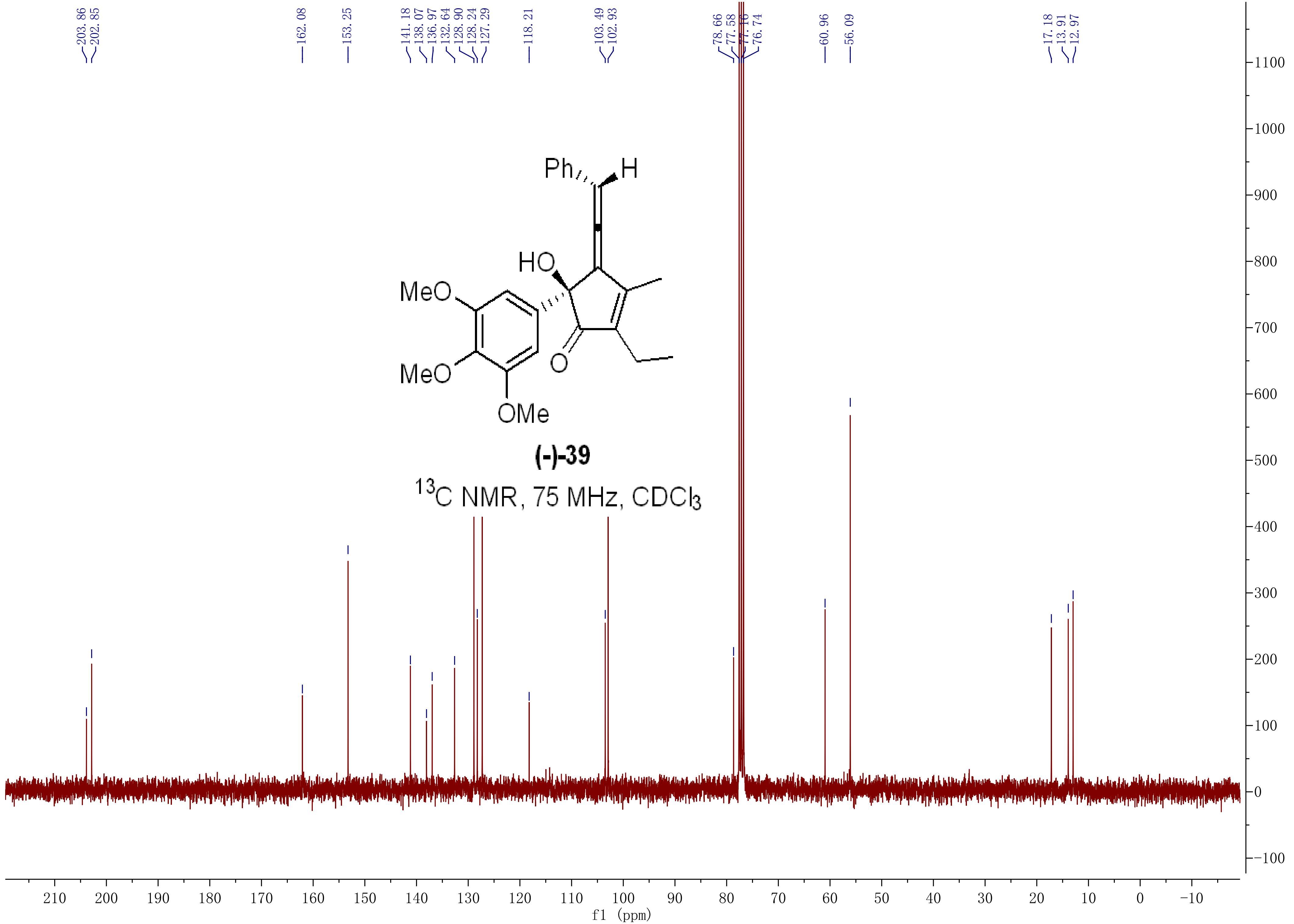

Supplementary Figure 109. ^{13}C NMR of compound (-)-39.

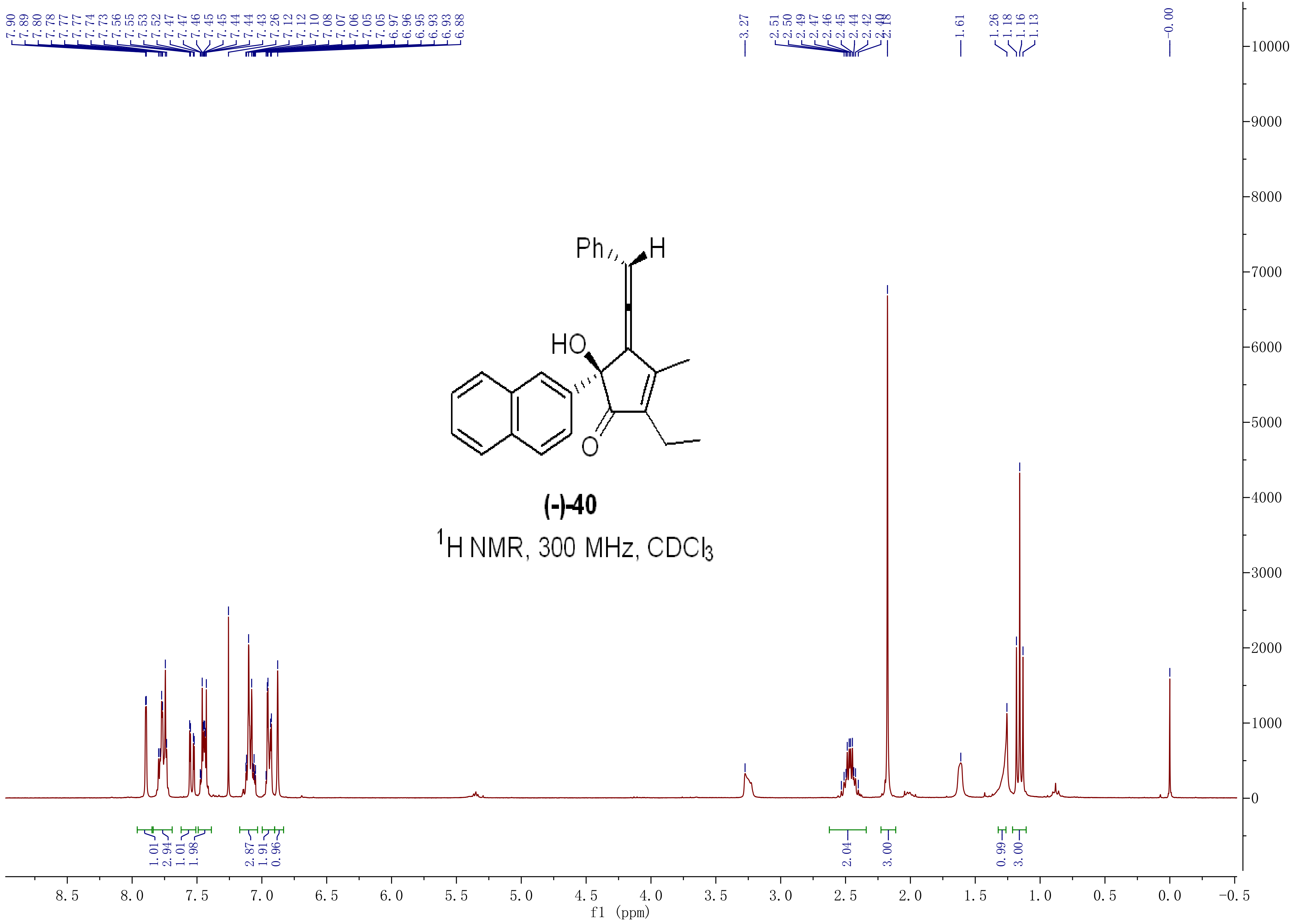

Supplementary Figure 110. ^{1}H NMR of compound (-)-40.

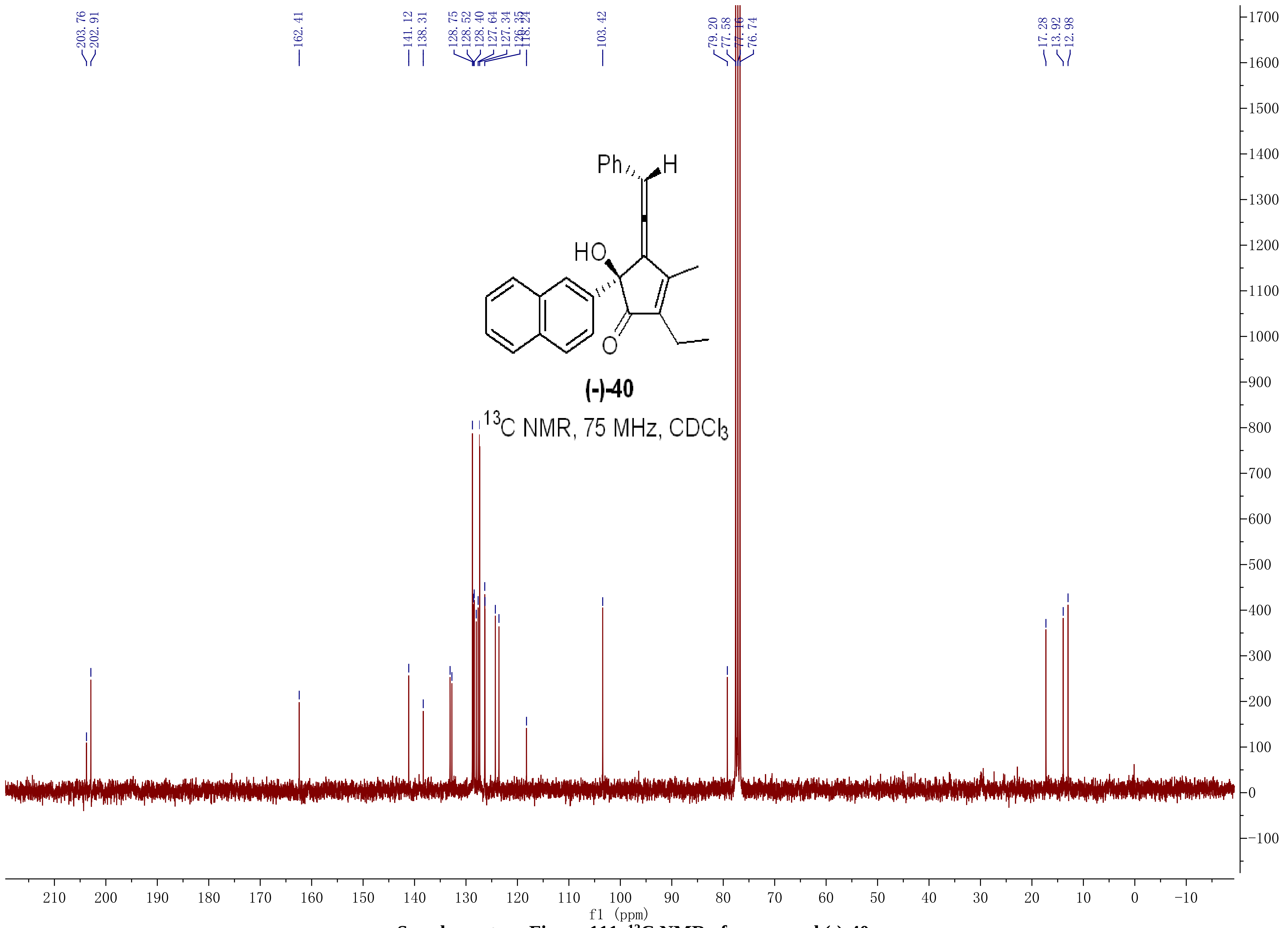

Supplementary Figure 111. ^{13}C NMR of compound (-)-40.

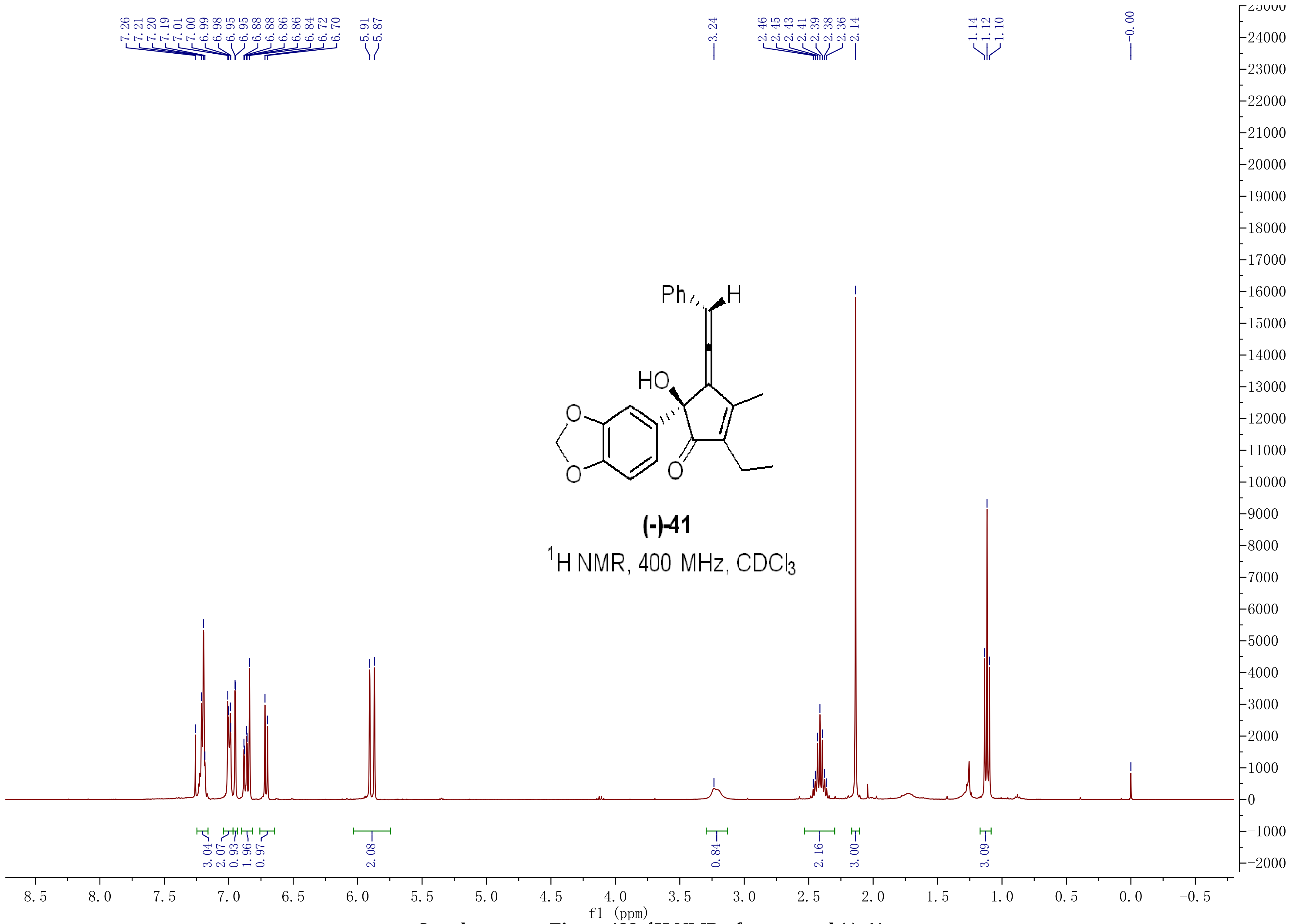

Supplementary Figure 122. ^{1}H NMR of compound (-)-41.

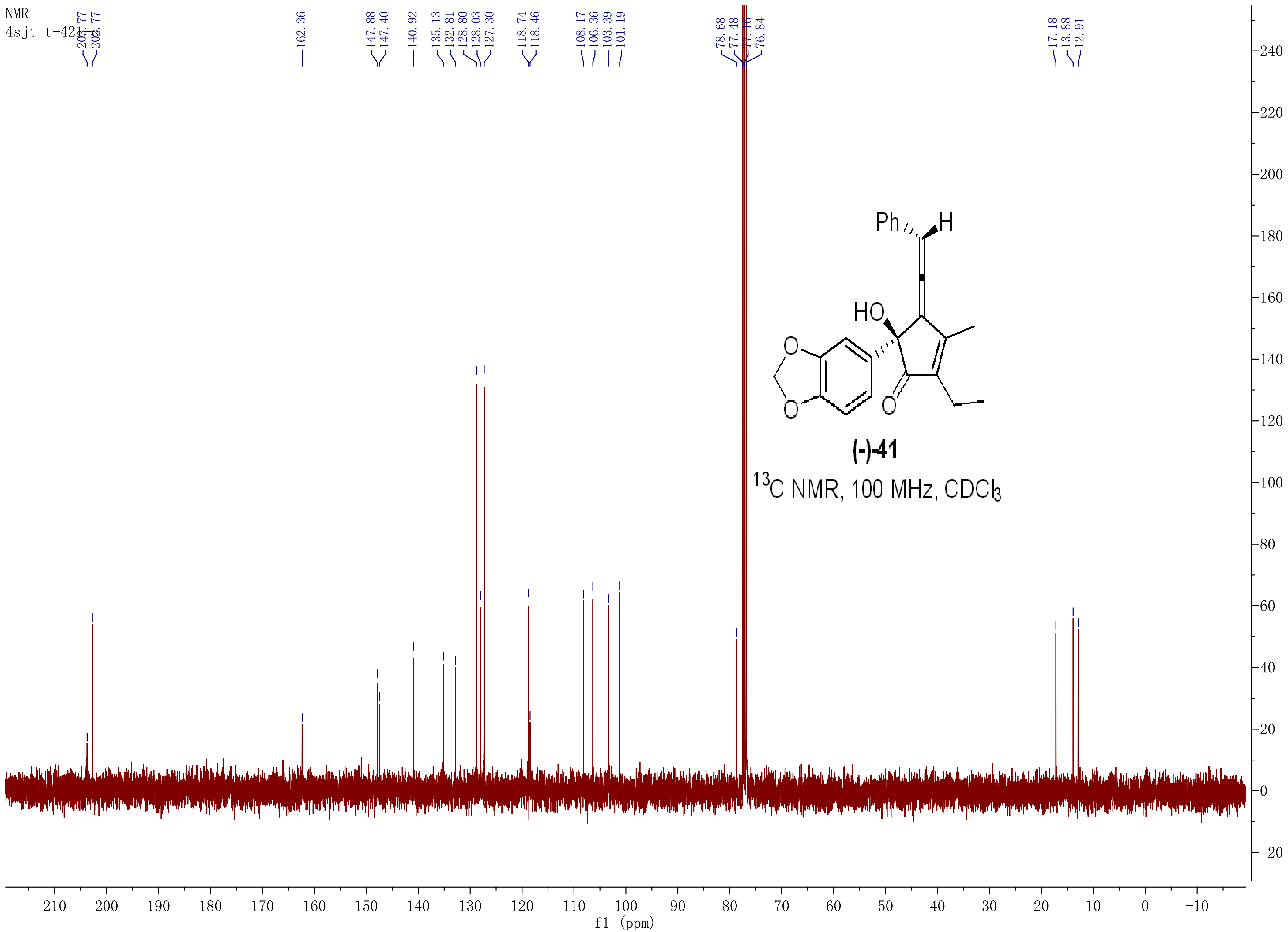

Supplementary Figure 113. ^{13}C NMR of compound (-)-41.

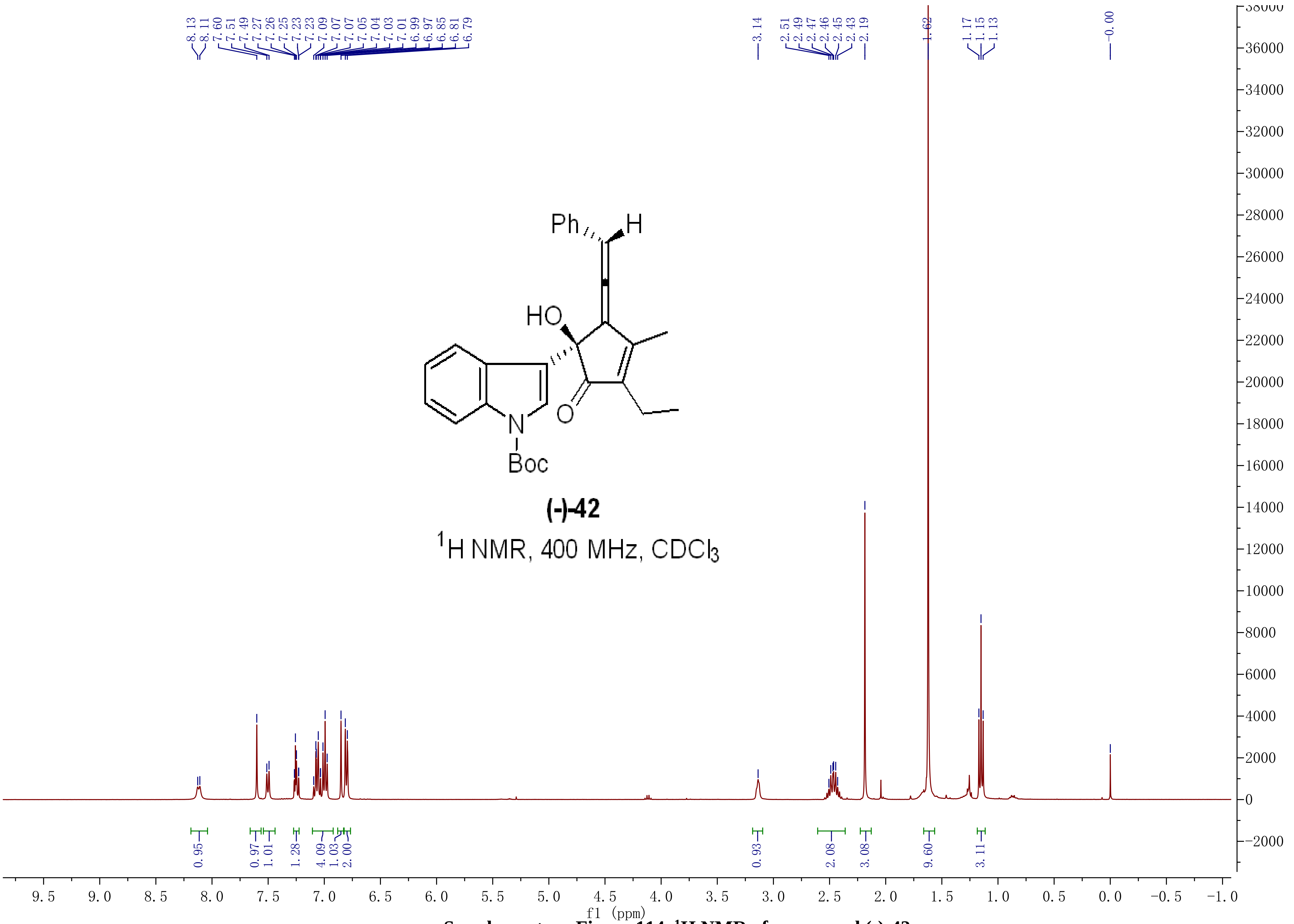

Supplementary Figure 114. ¹H NMR of compound (-)-42.

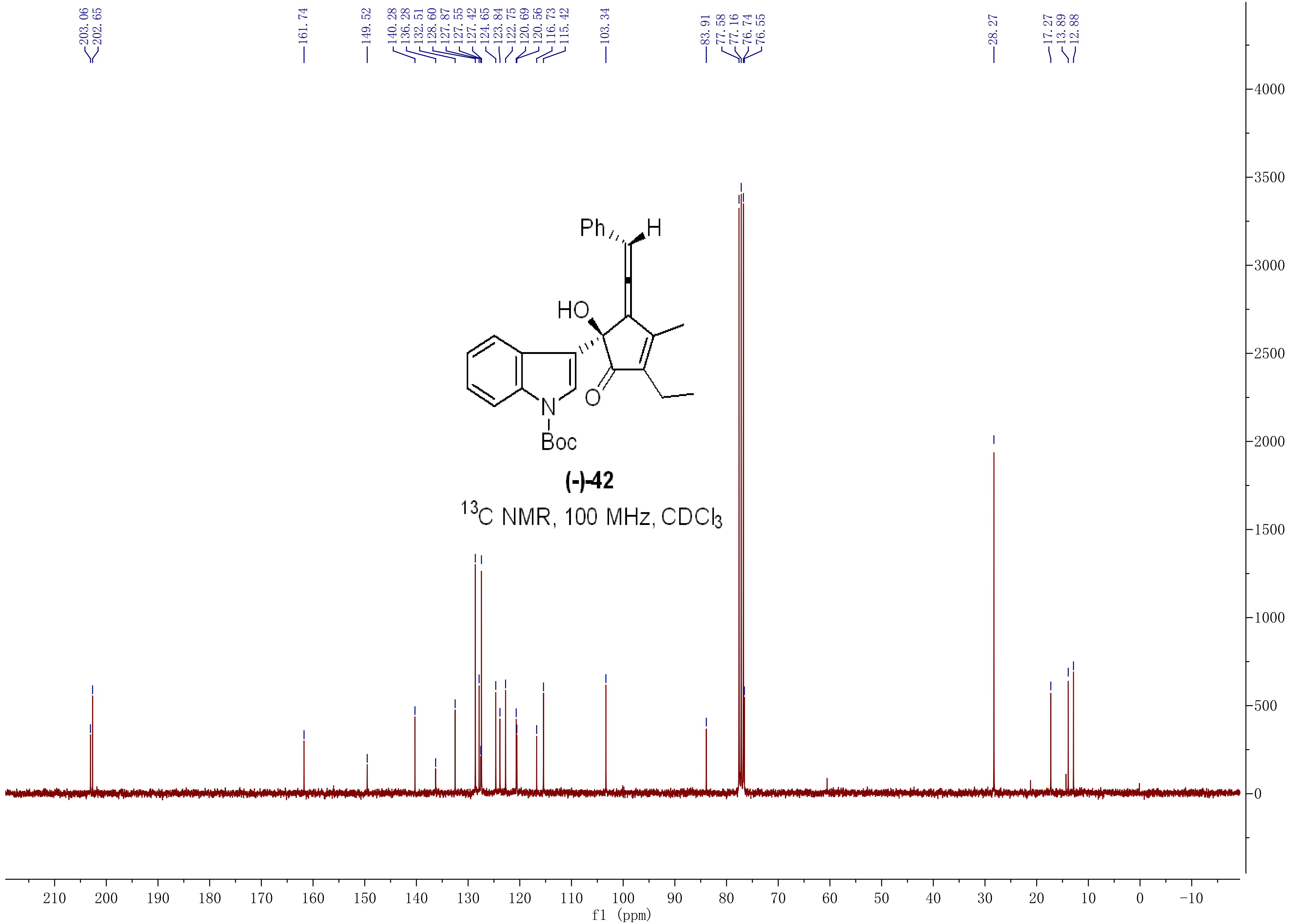

Supplementary Figure 115. ^{13}C NMR of compound (-)-42.

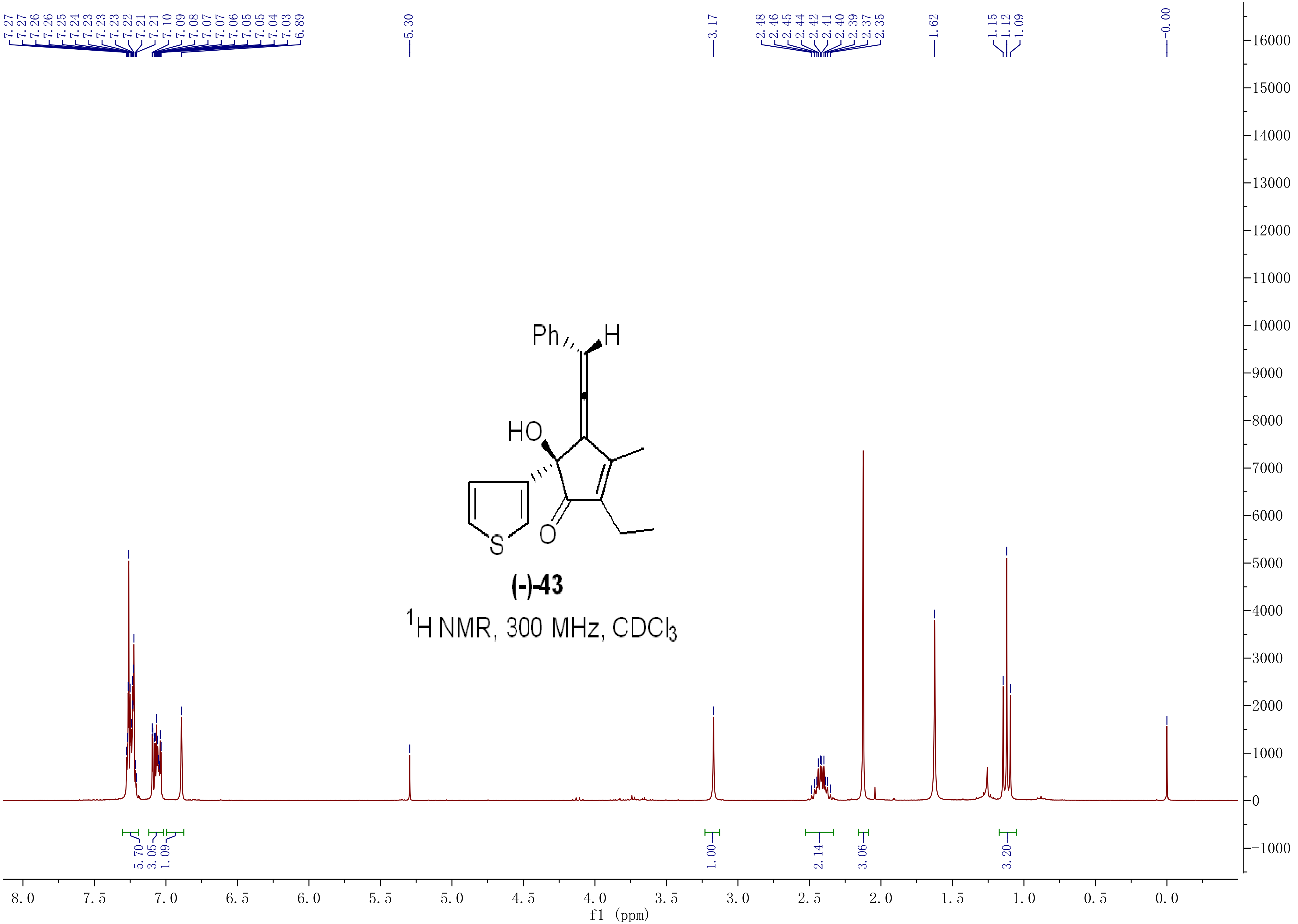

Supplementary Figure 116. ^{1}H NMR of compound (-)-43.

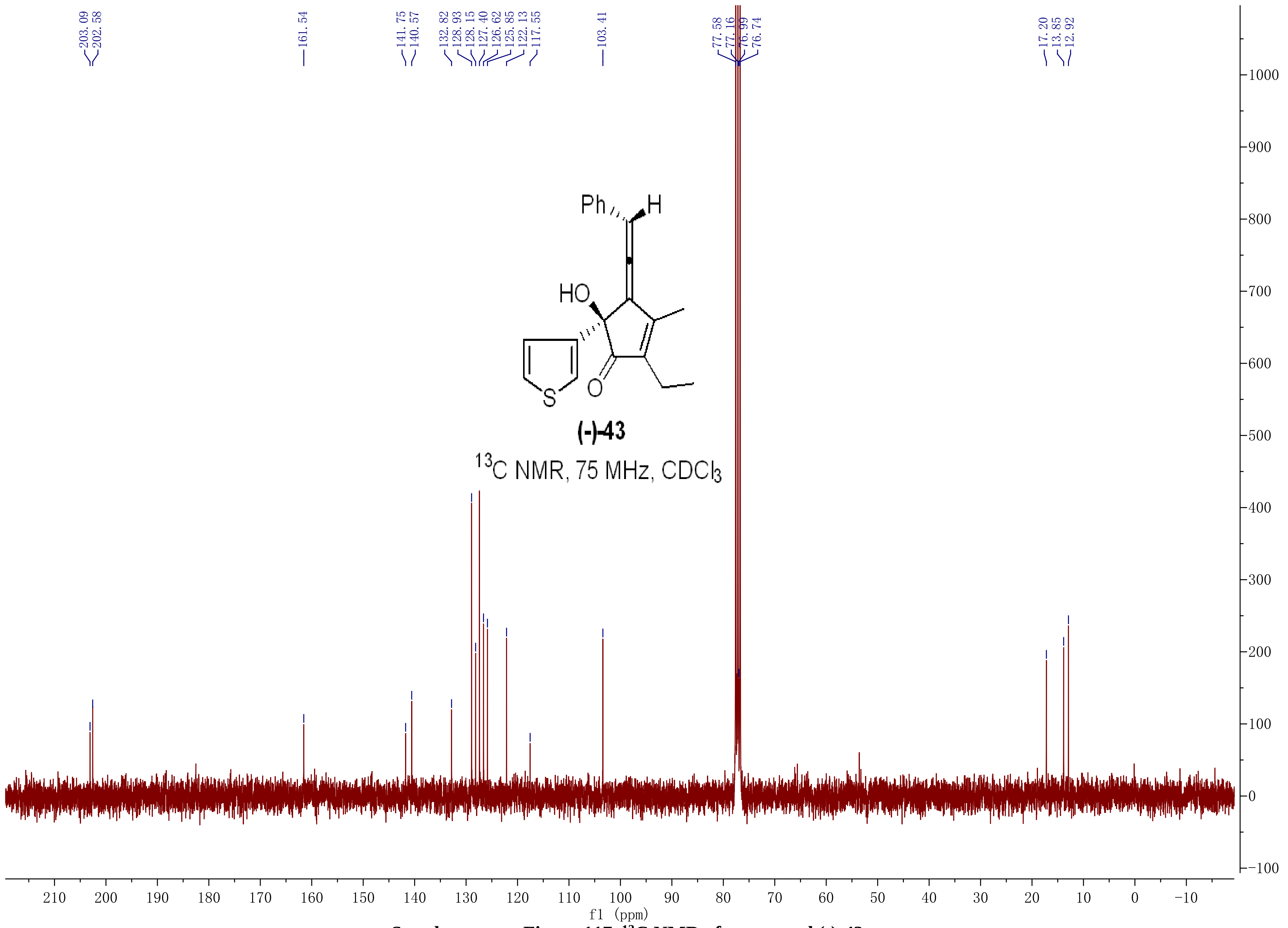

Supplementary Figure 117. ^{13}C NMR of compound (-)-43.

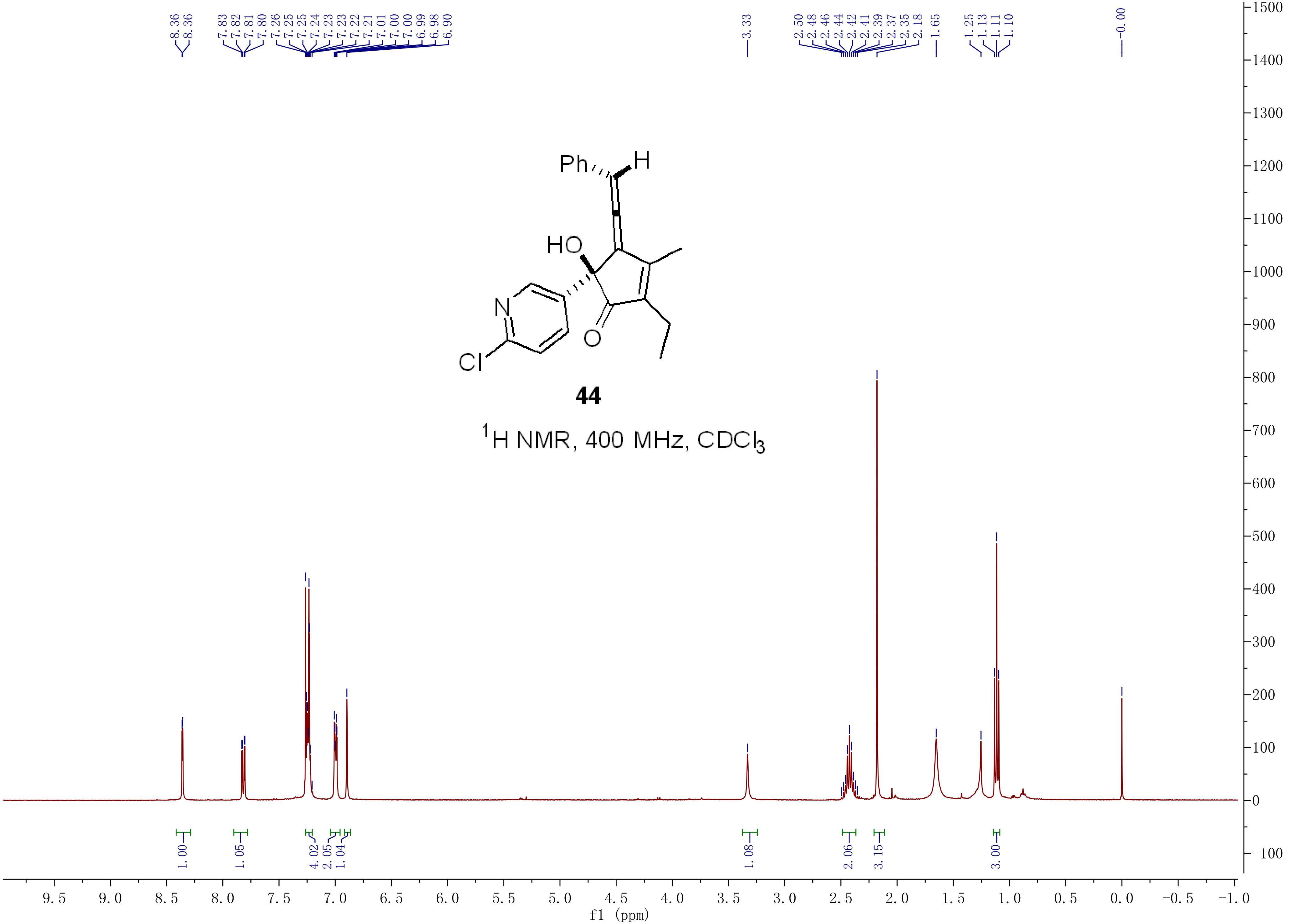

Supplementary Figure 118. ^{1}H NMR of compound 44.

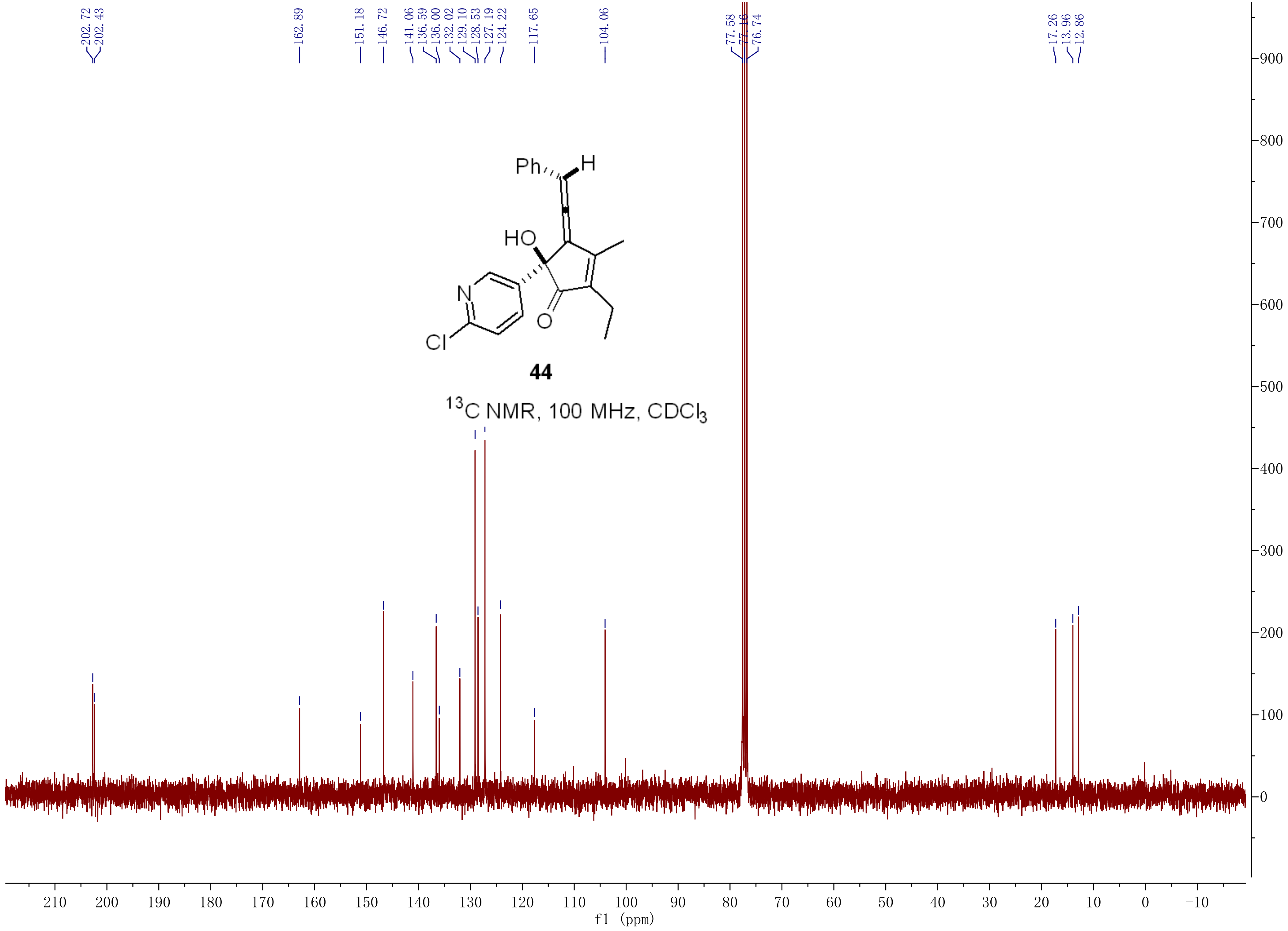

Supplementary Figure 119. ^{13}C NMR of compound 44.

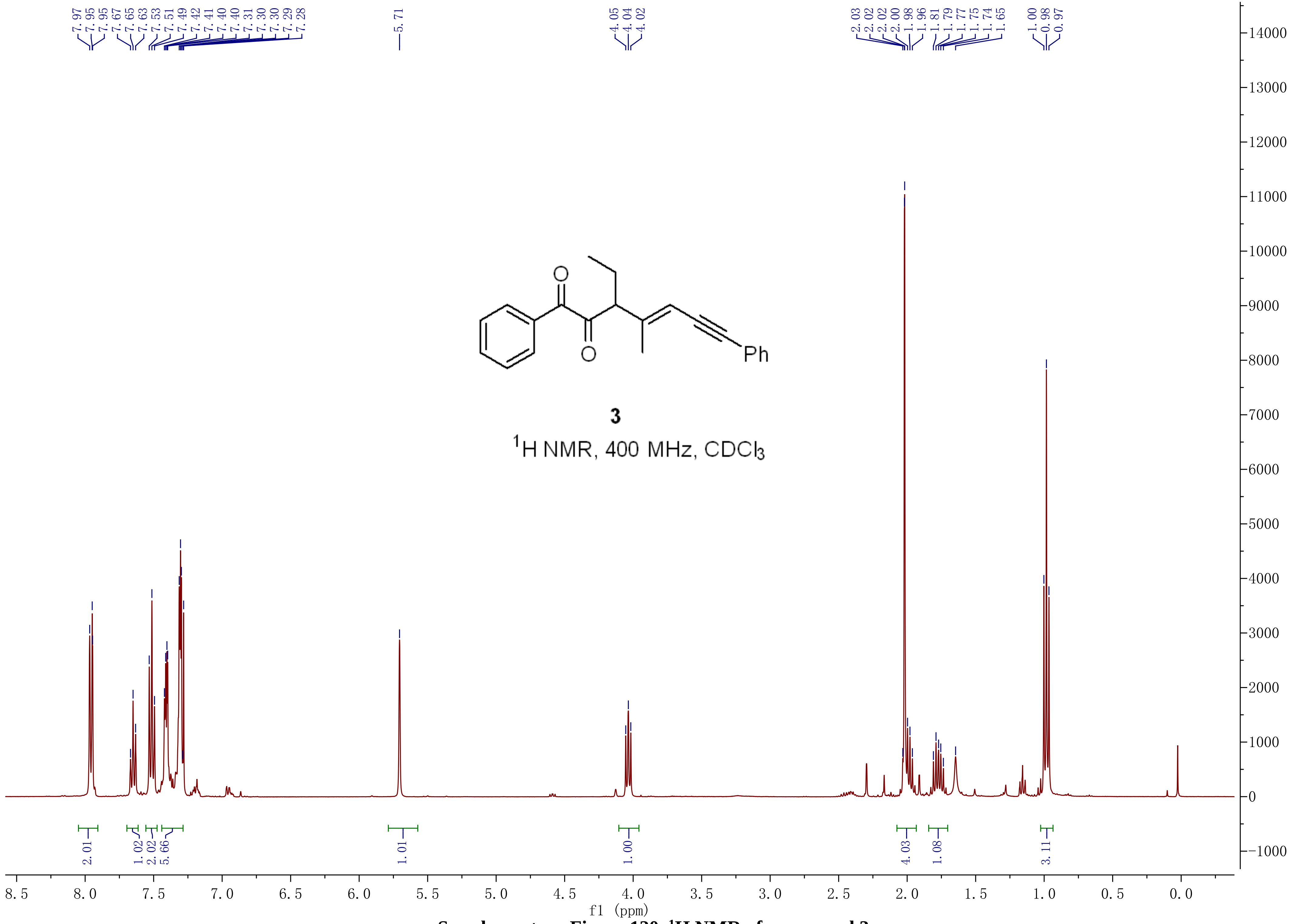

Supplementary Figure 120. ^{1}H NMR of compound 3.

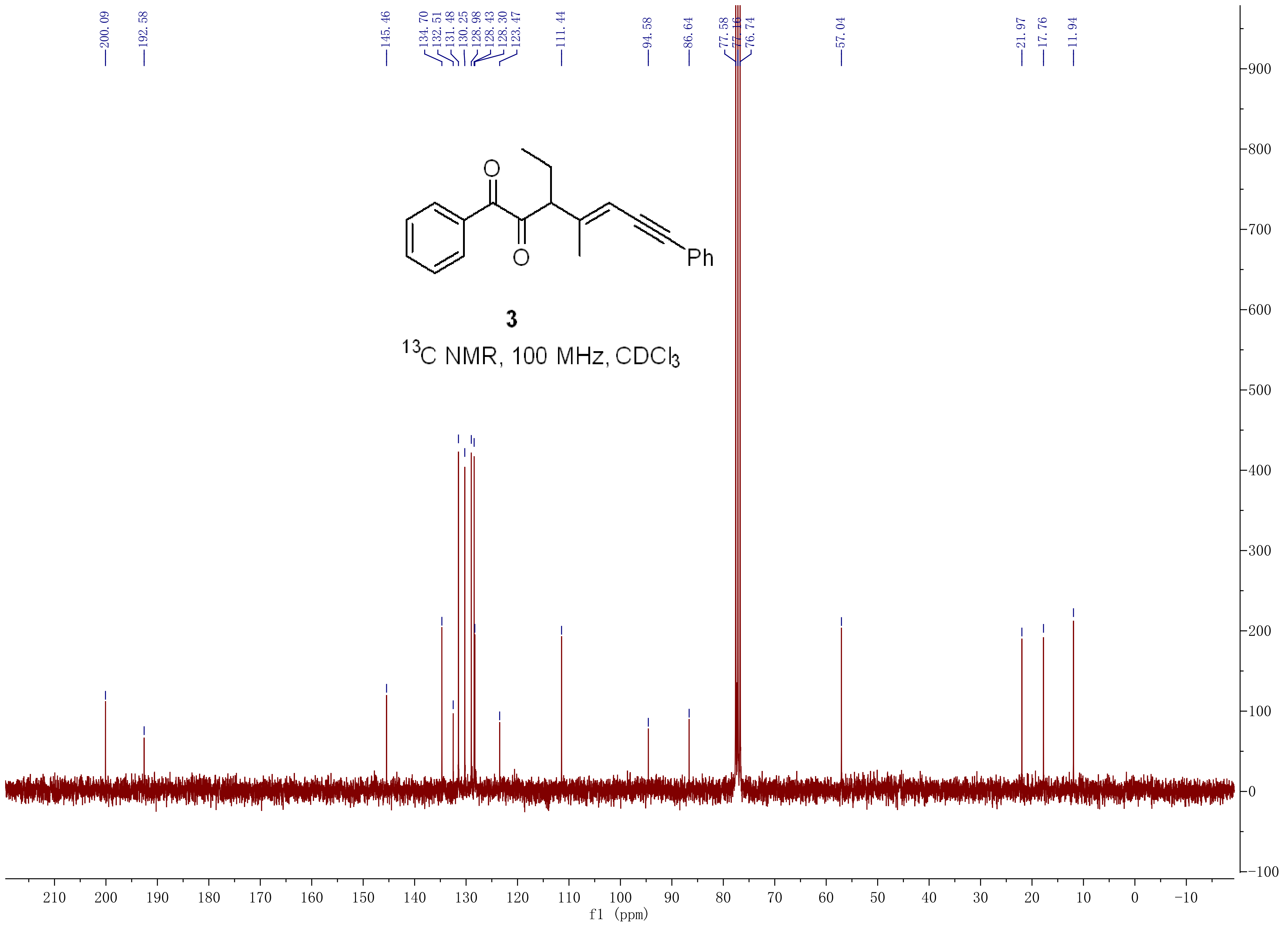

Supplementary Figure 121. ^{13}C NMR of compound 3.

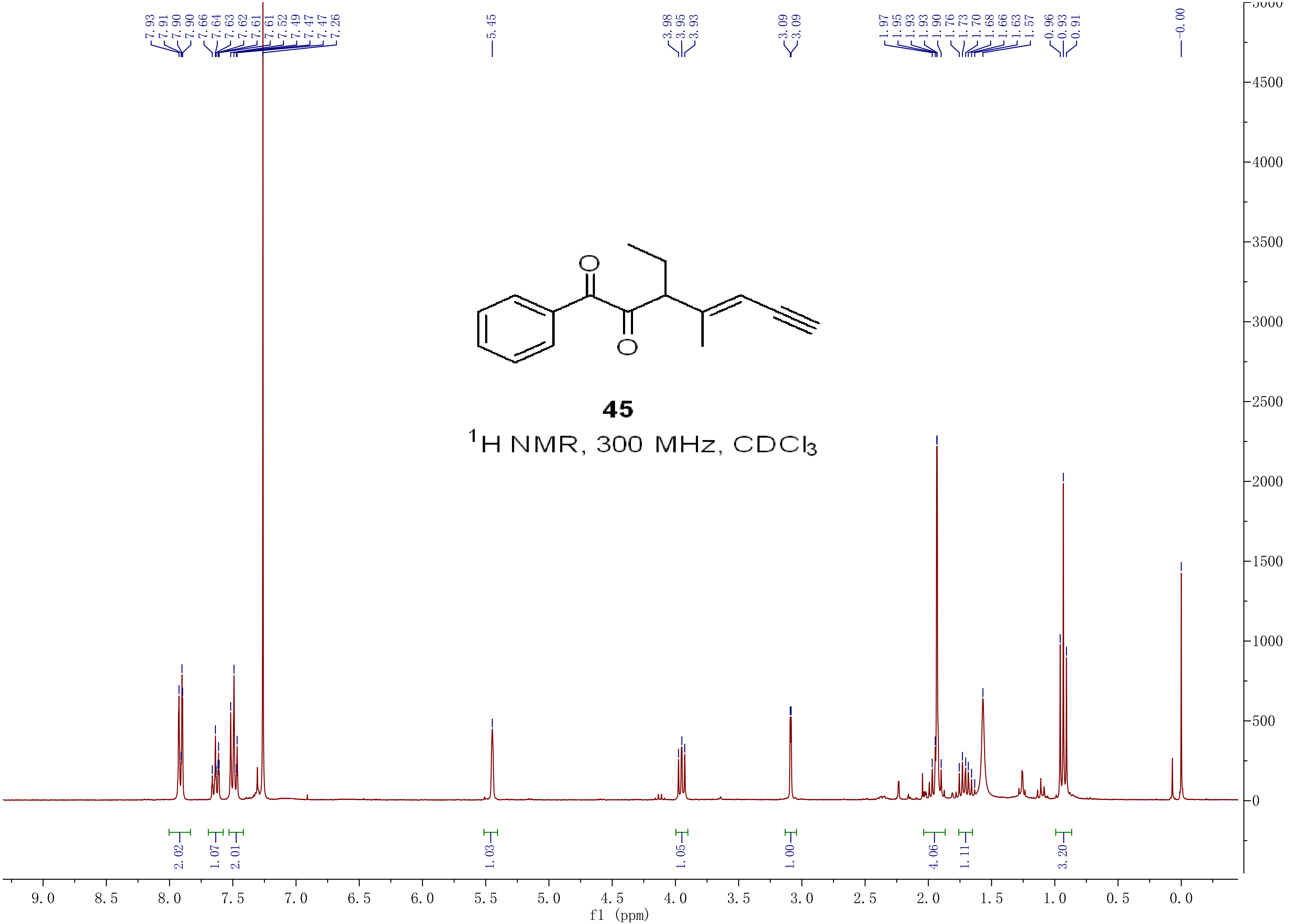

Supplementary Figure 122. ^{1}H NMR of compound 45.

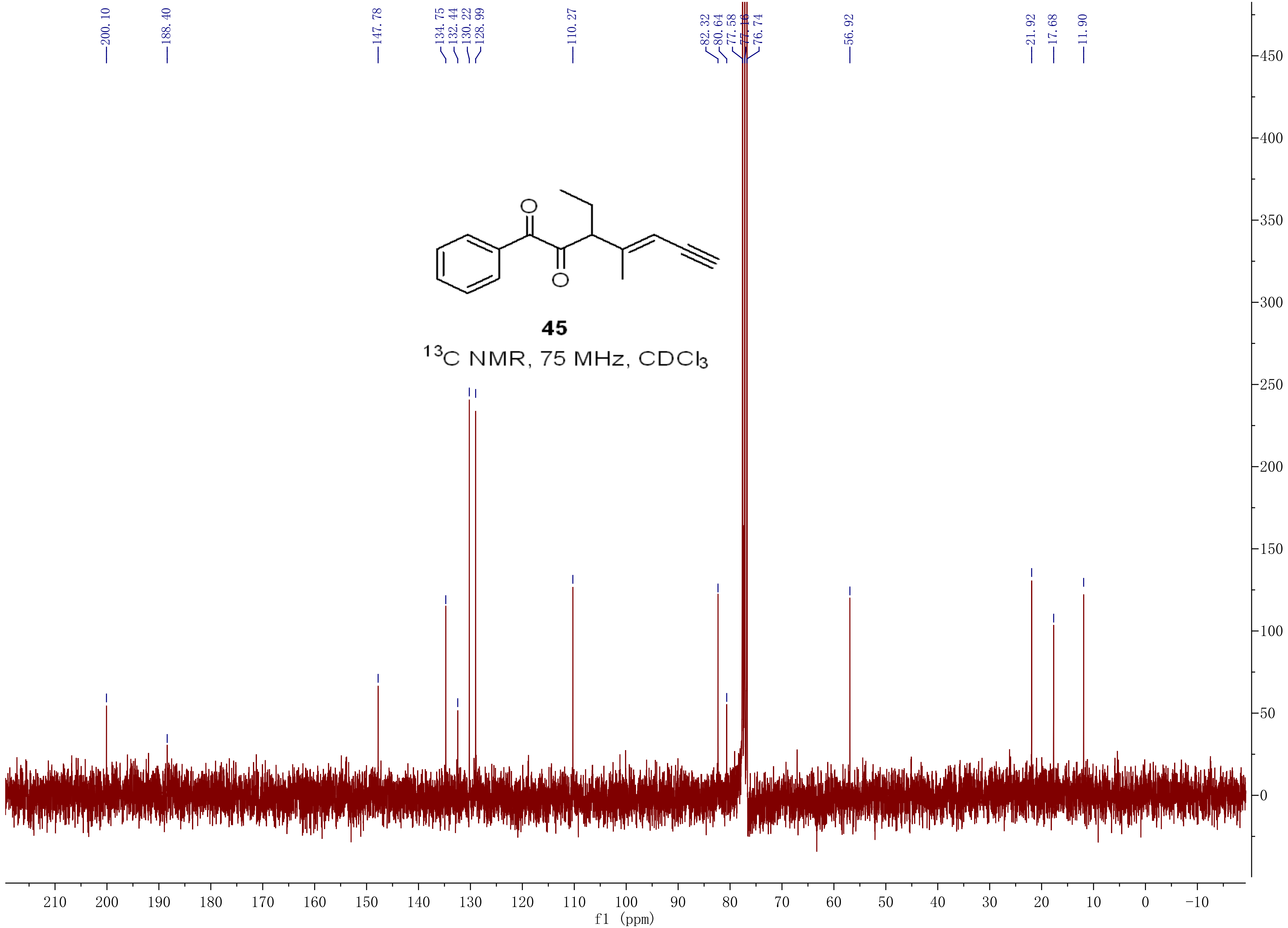

Supplementary Figure 123. ^{13}C NMR of compound 45.

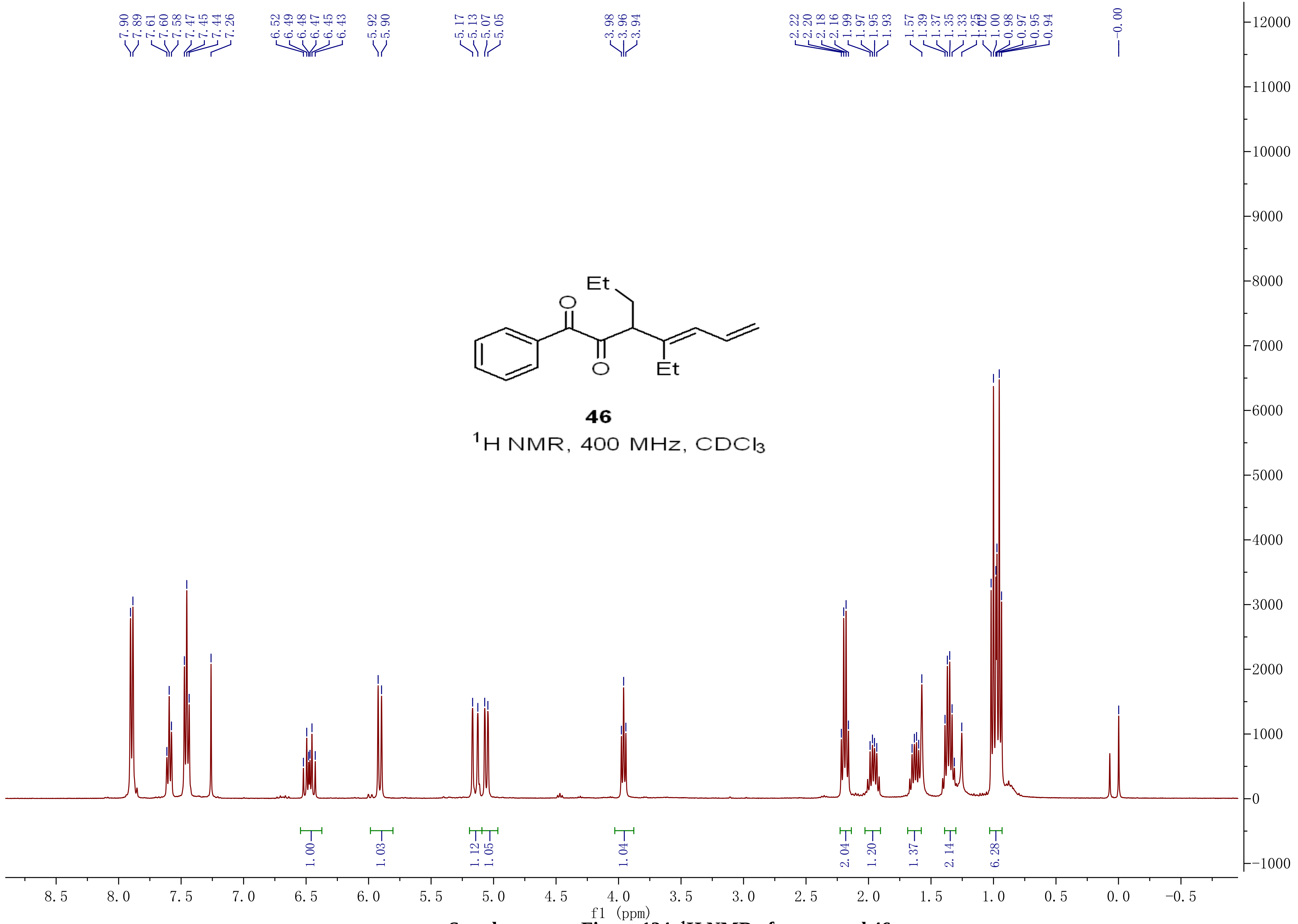

Supplementary Figure 124. ^{1}H NMR of compound 46.

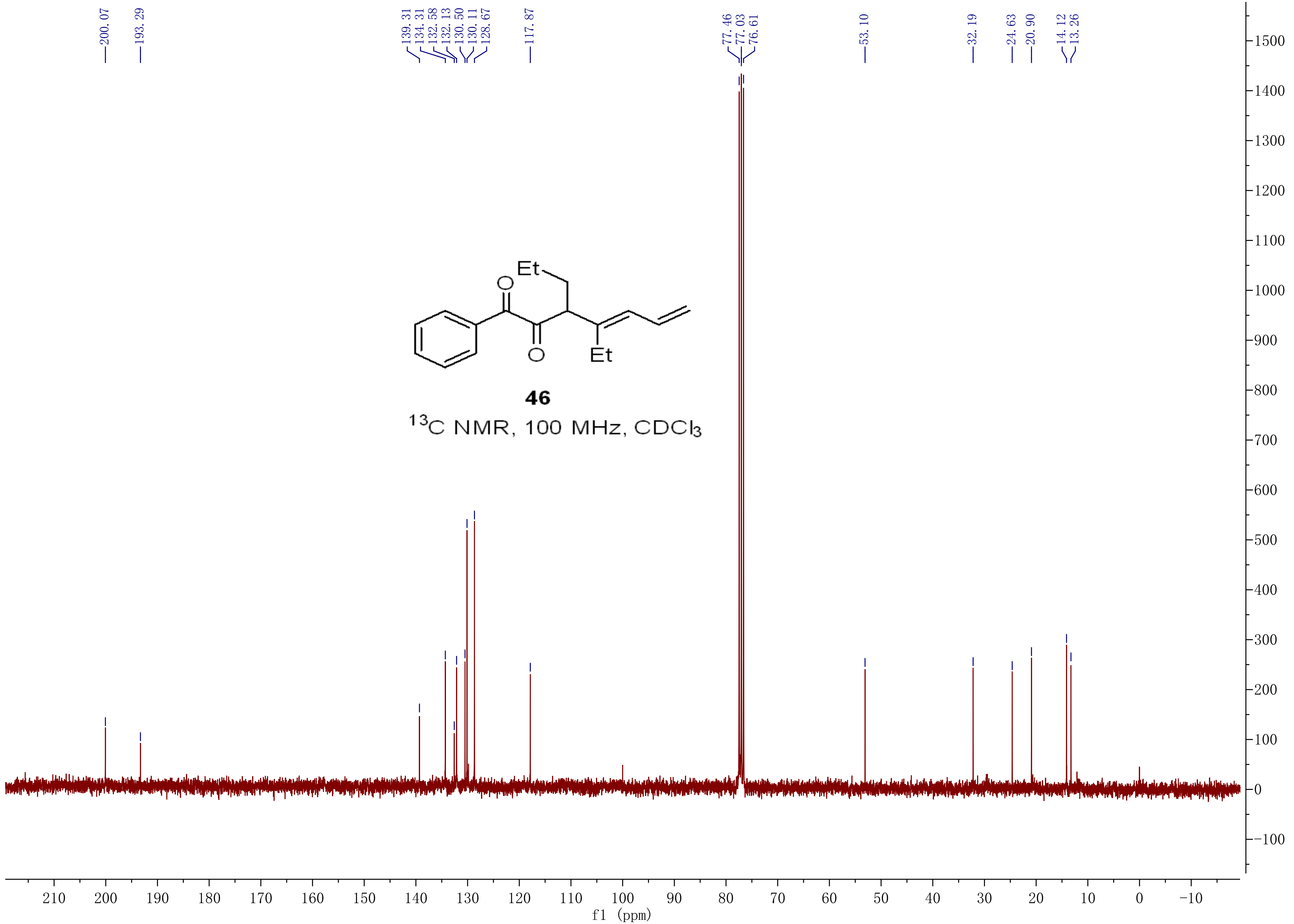

Supplementary Figure 125. ^{13}C NMR of compound 46.

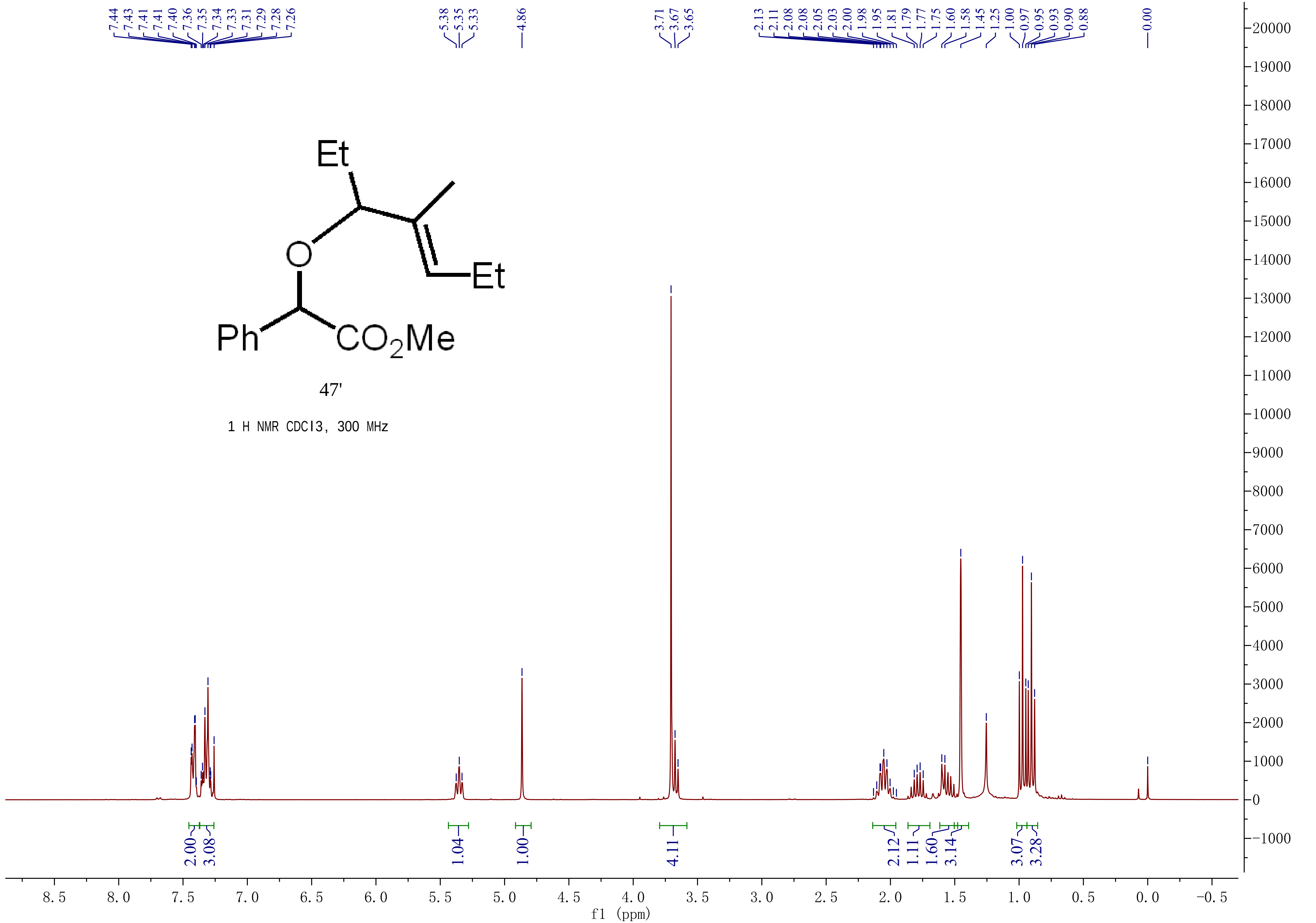

Supplementary Figure 126. ^{1}H NMR of compound 47'.

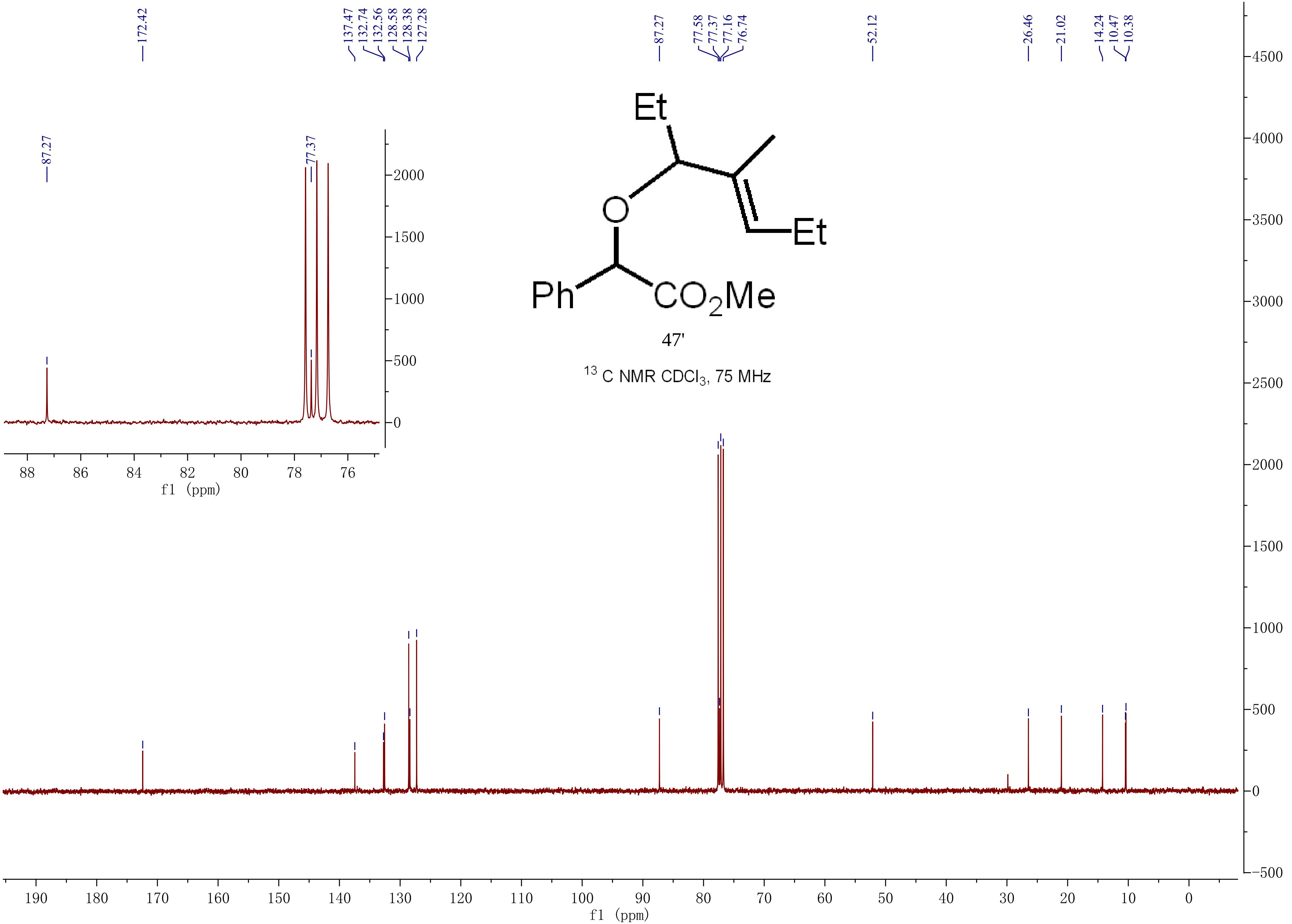

Supplementary Figure 127. ^{13}C NMR of compound 47'.

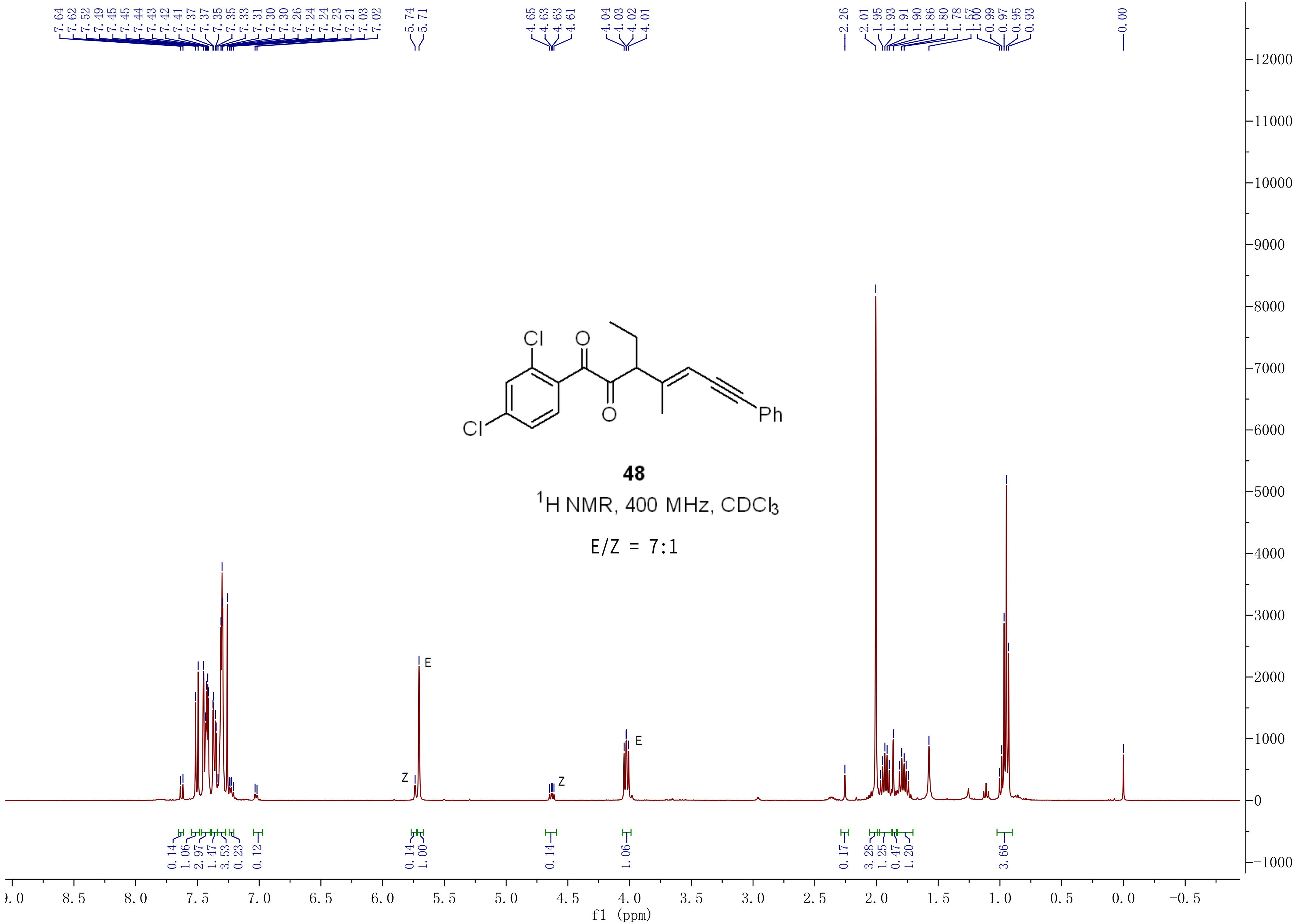

Supplementary Figure 128. ^{1}H NMR of compound 48.

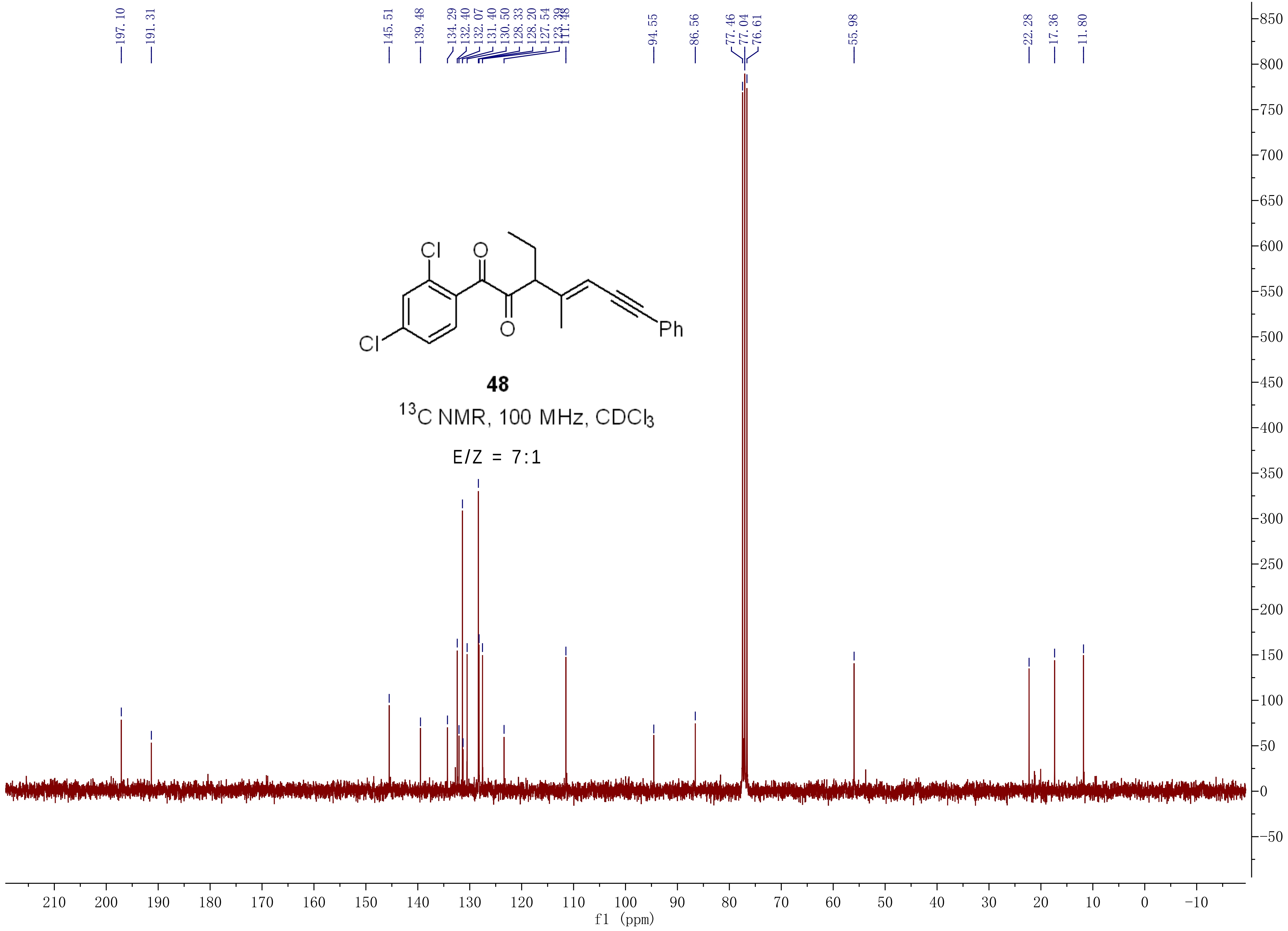

Supplementary Figure 129. ^{13}C NMR of compound 48.

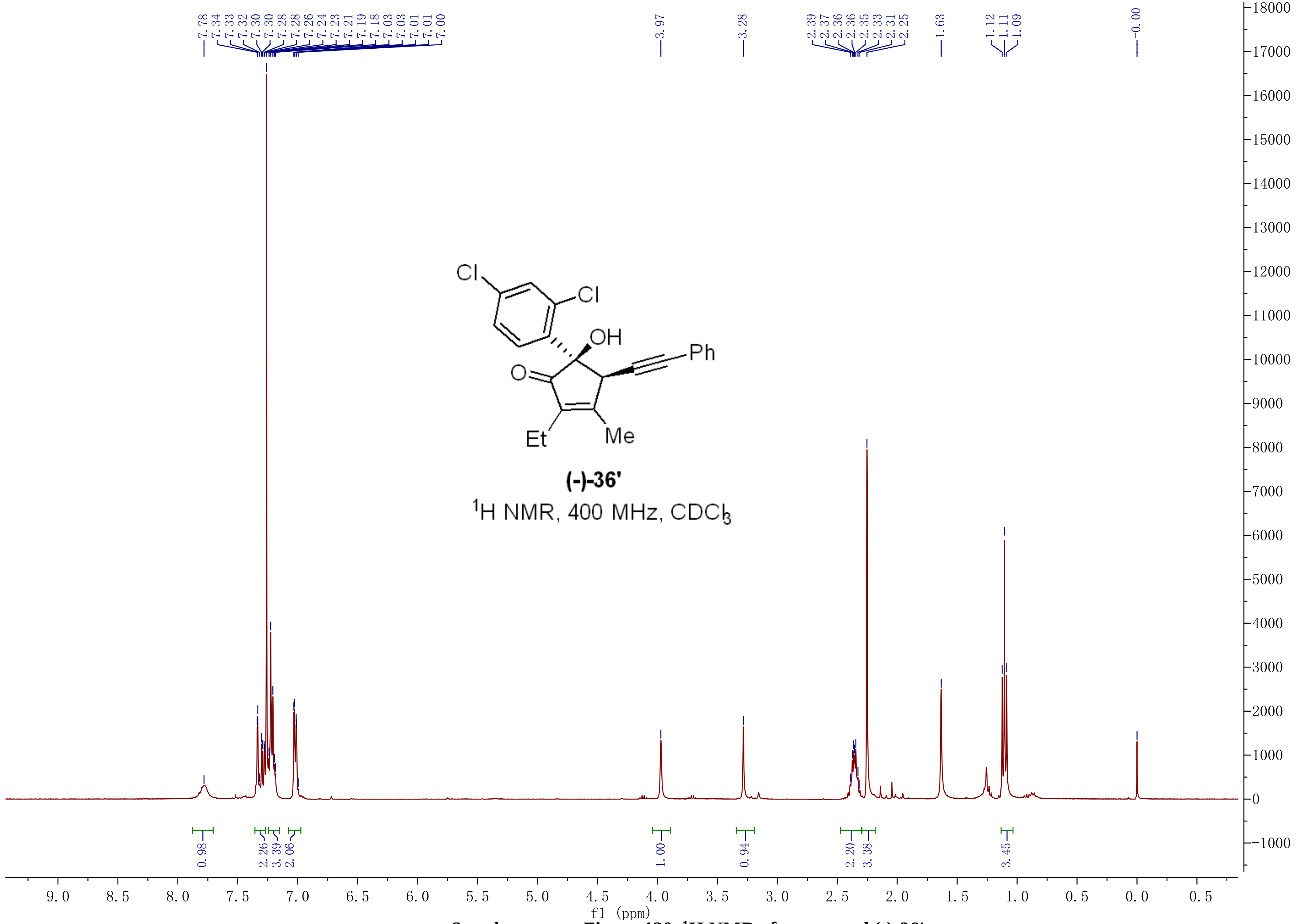

Supplementary Figure 130. ^{1}H NMR of compound (-)-36'.

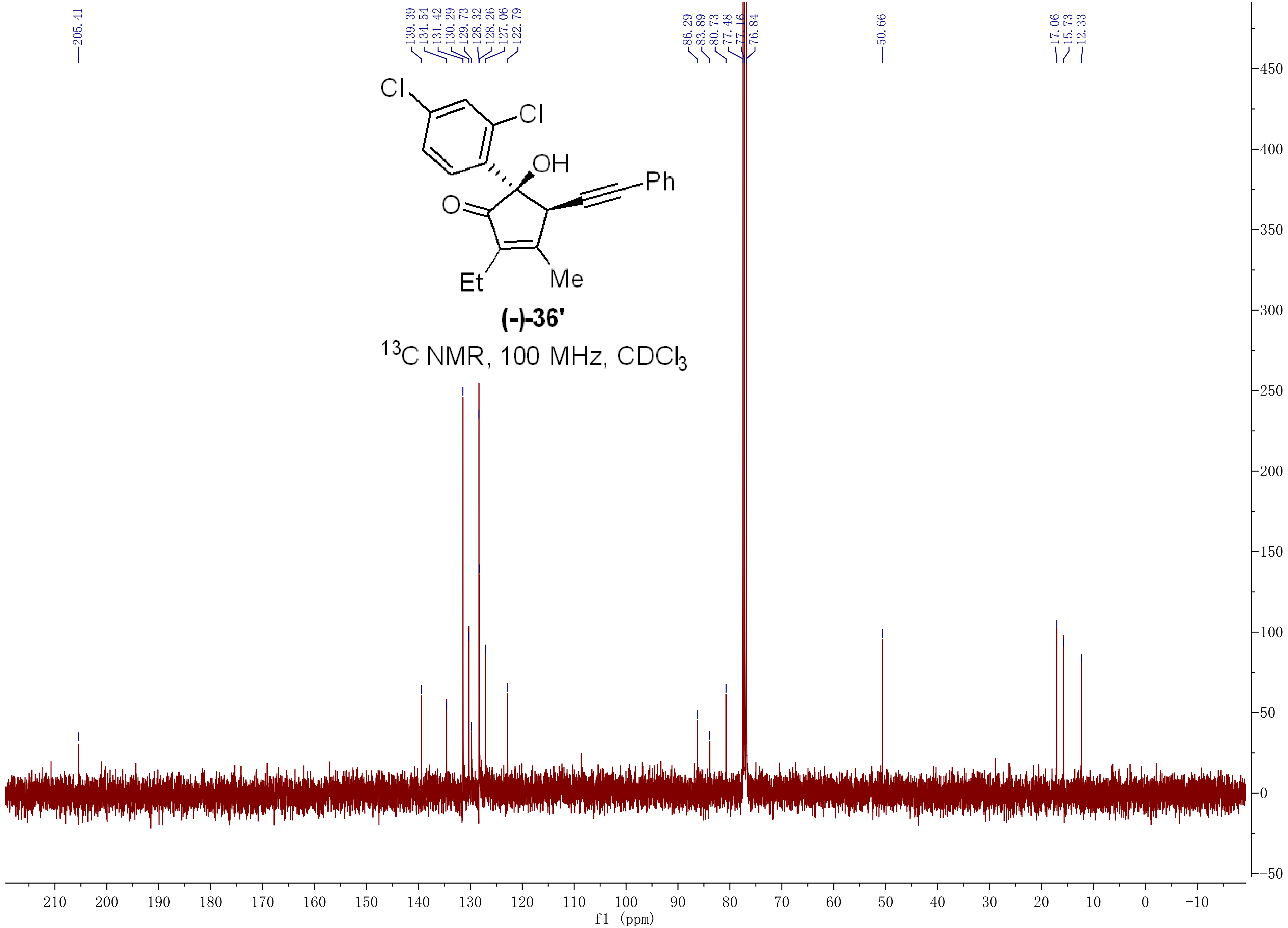

Supplementary Figure 131. ^{13}**C NMR of compound (-)-36'.**

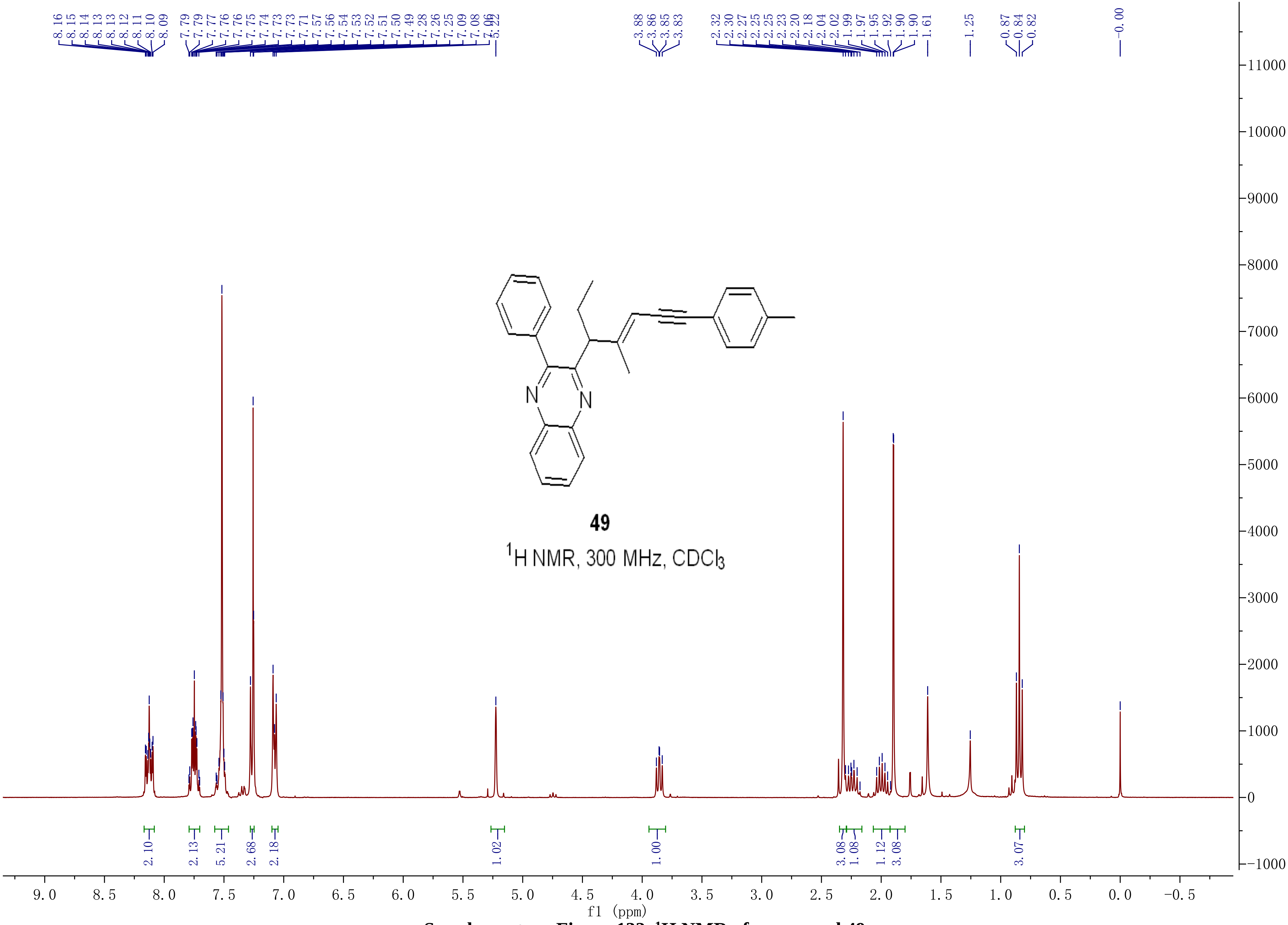

Supplementary Figure 132. ^{1}H NMR of compound 49.

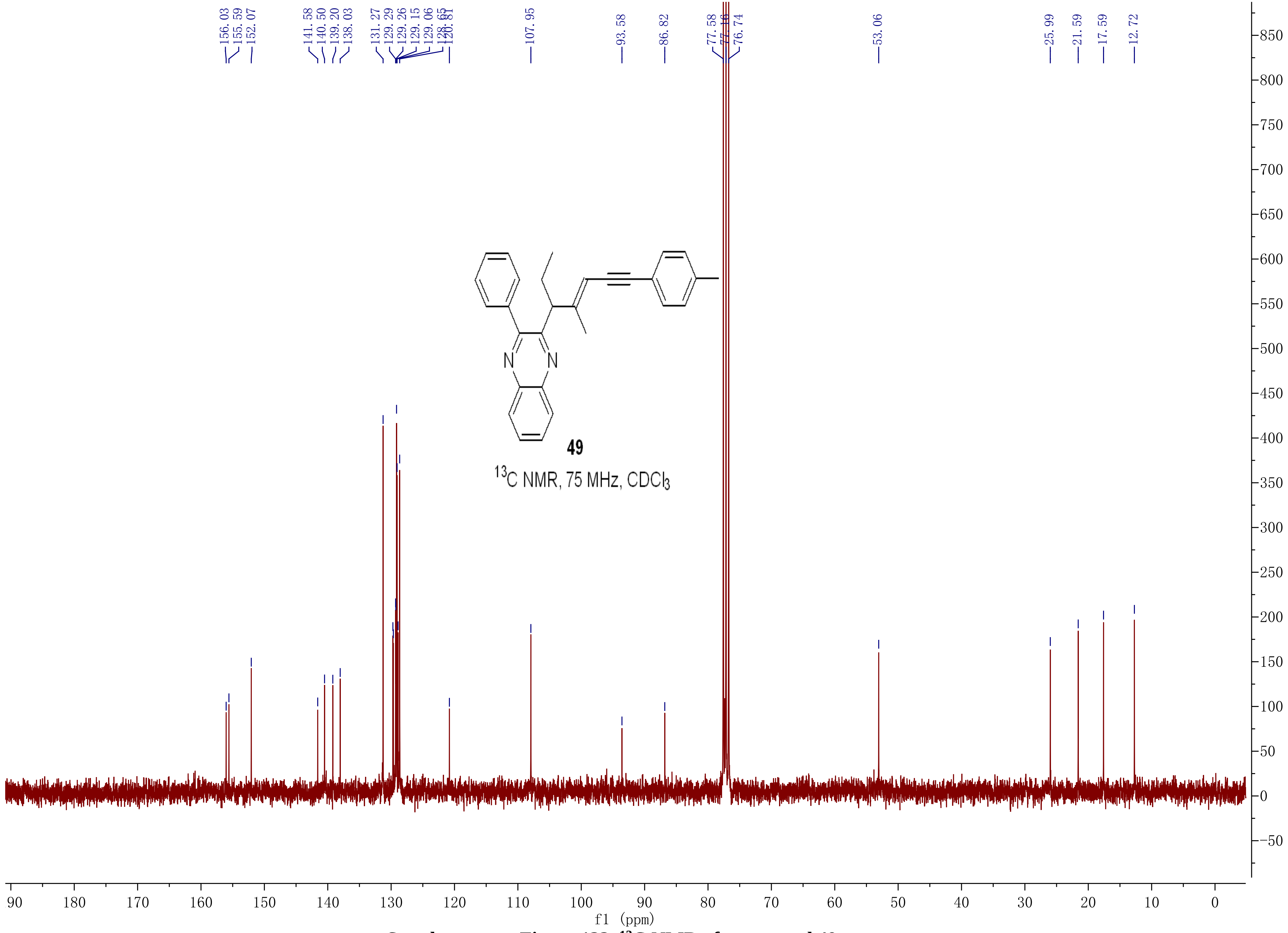

Supplementary Figure 133. ^{13}C NMR of compound 49.

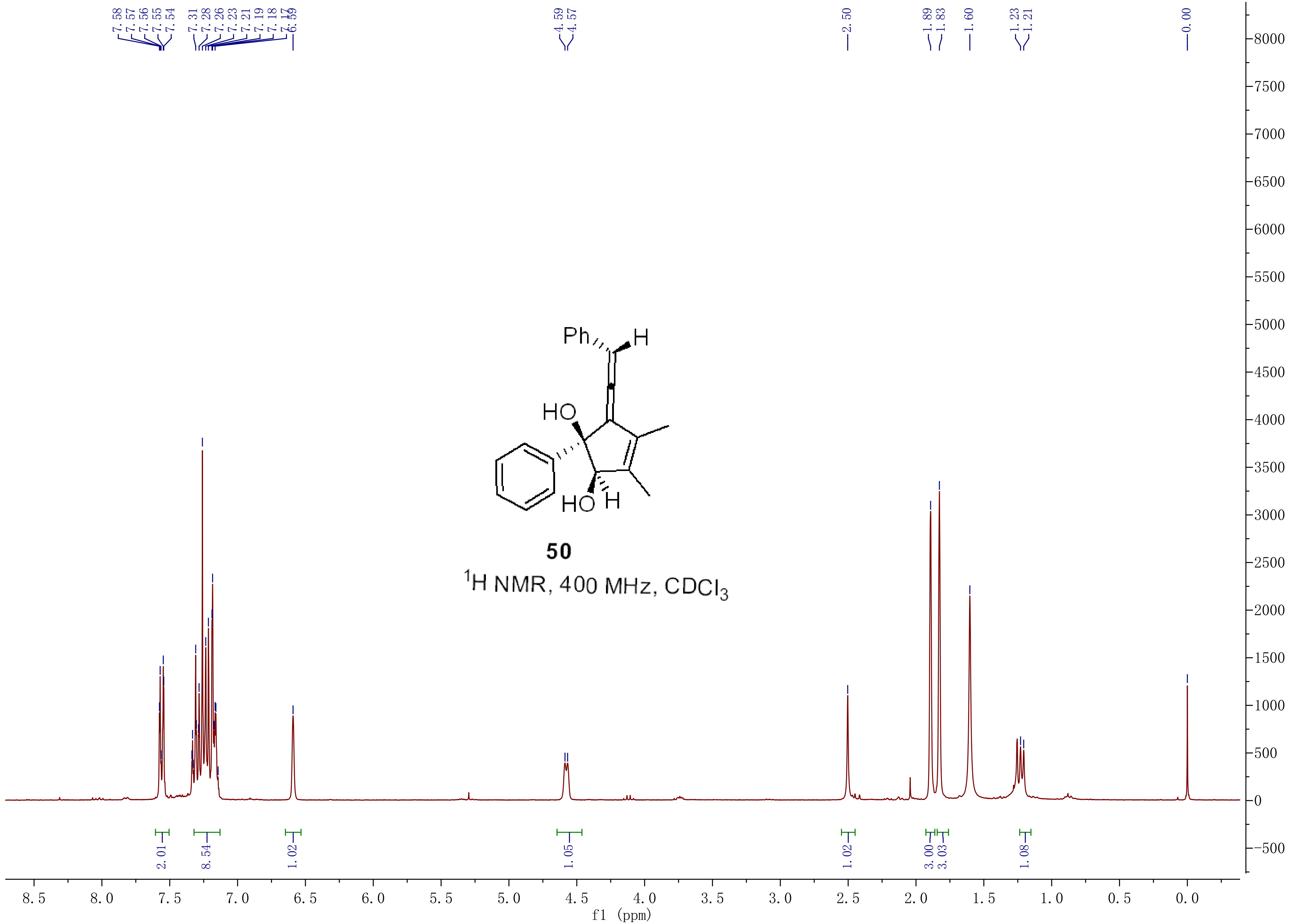

Supplementary Figure 134. ^{1}H NMR of compound (-)-50.

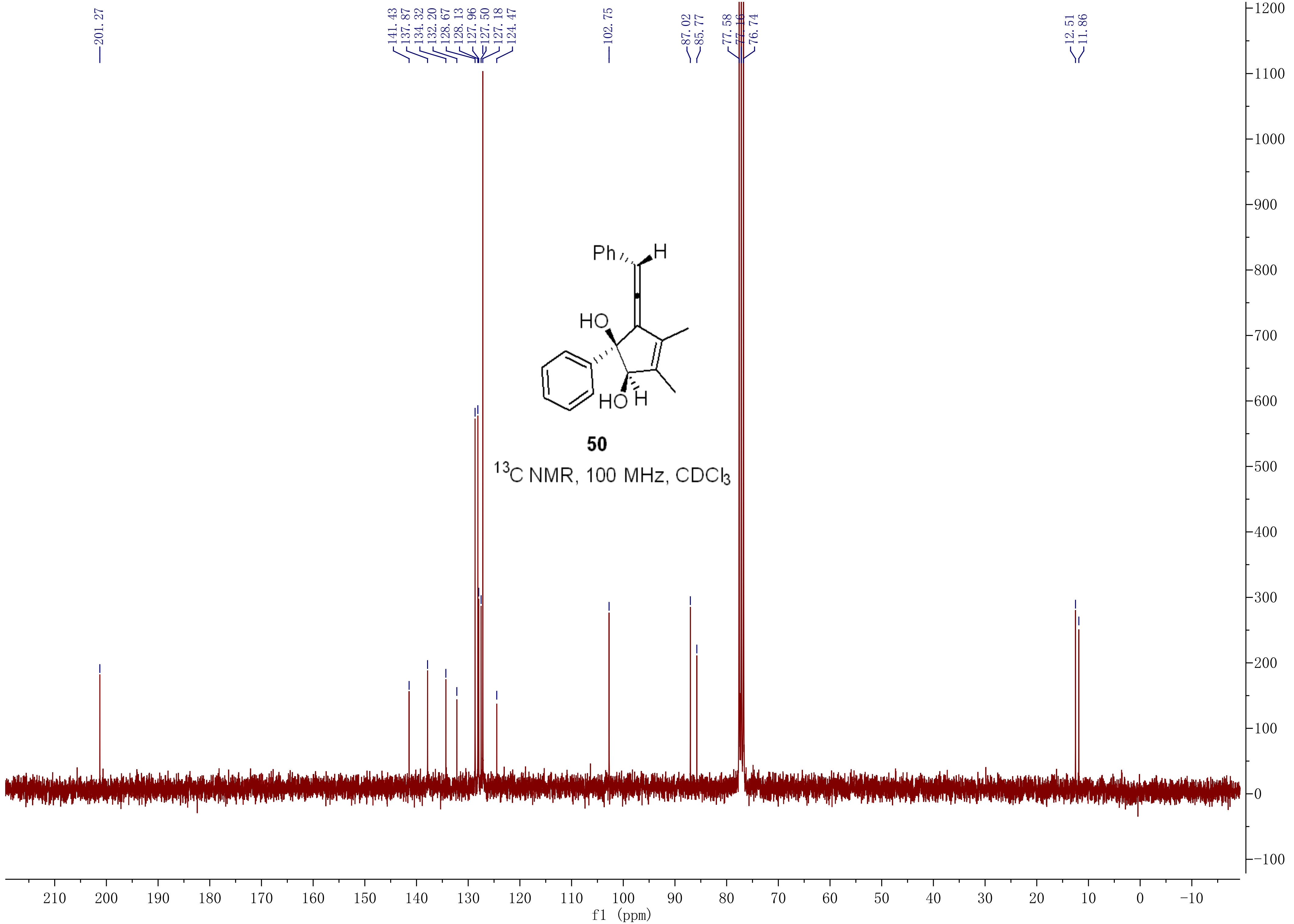

Supplementary Figure 135. ^{13}C NMR of compound (-)-50.

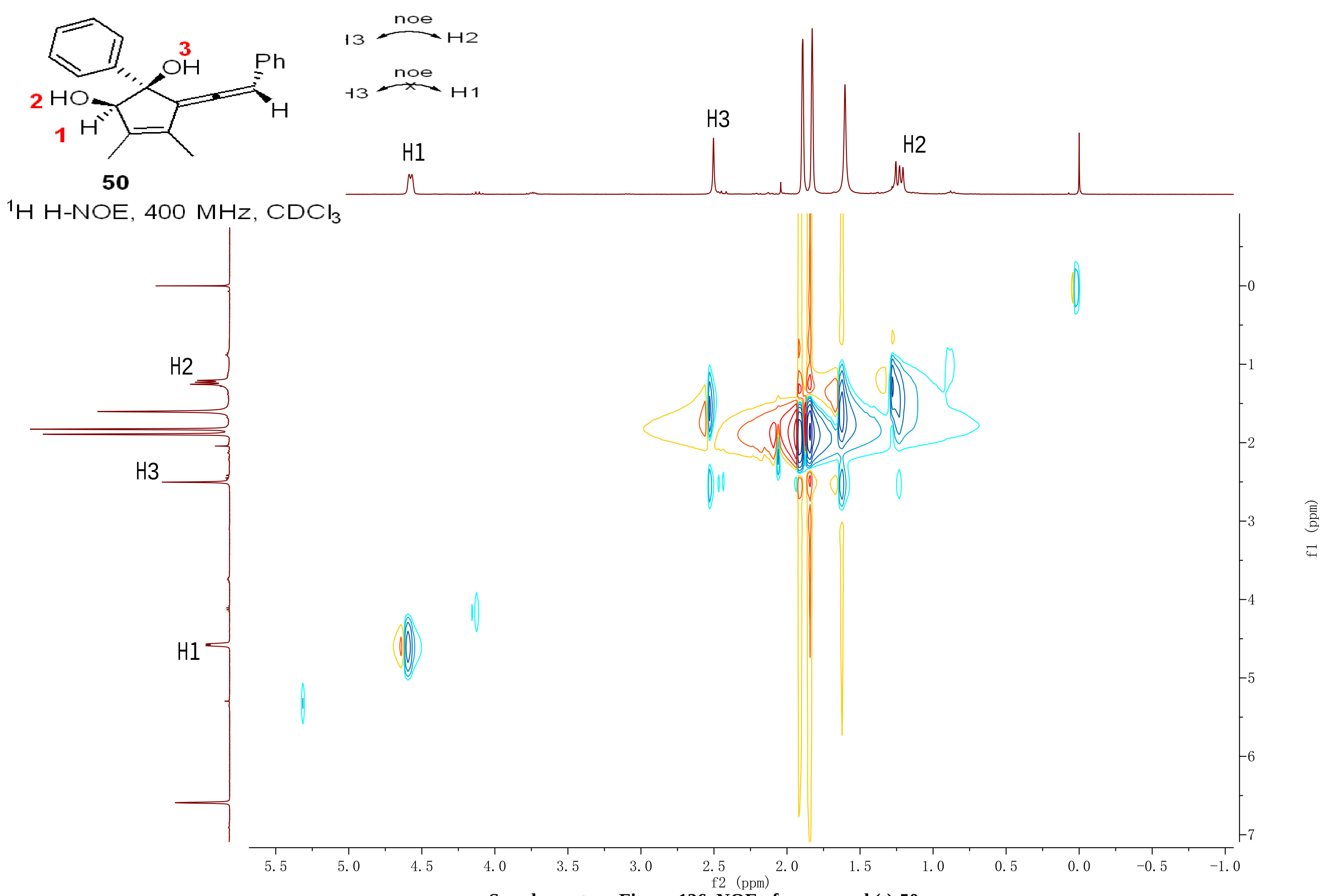

Supplementary Figure 136. NOE of compound (-)-50.

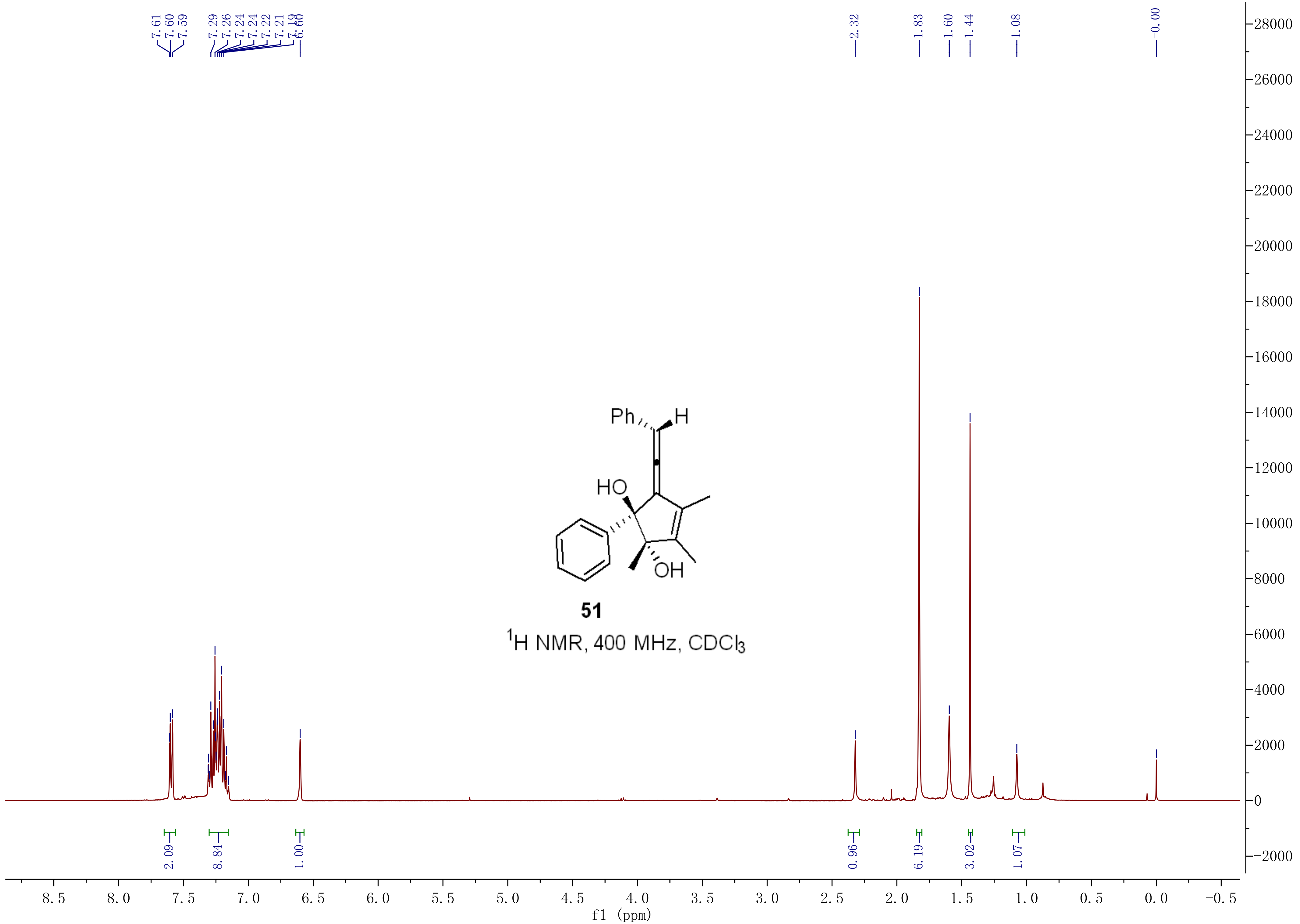

Supplementary Figure 137. ^{1}H NMR of compound (-)-51.

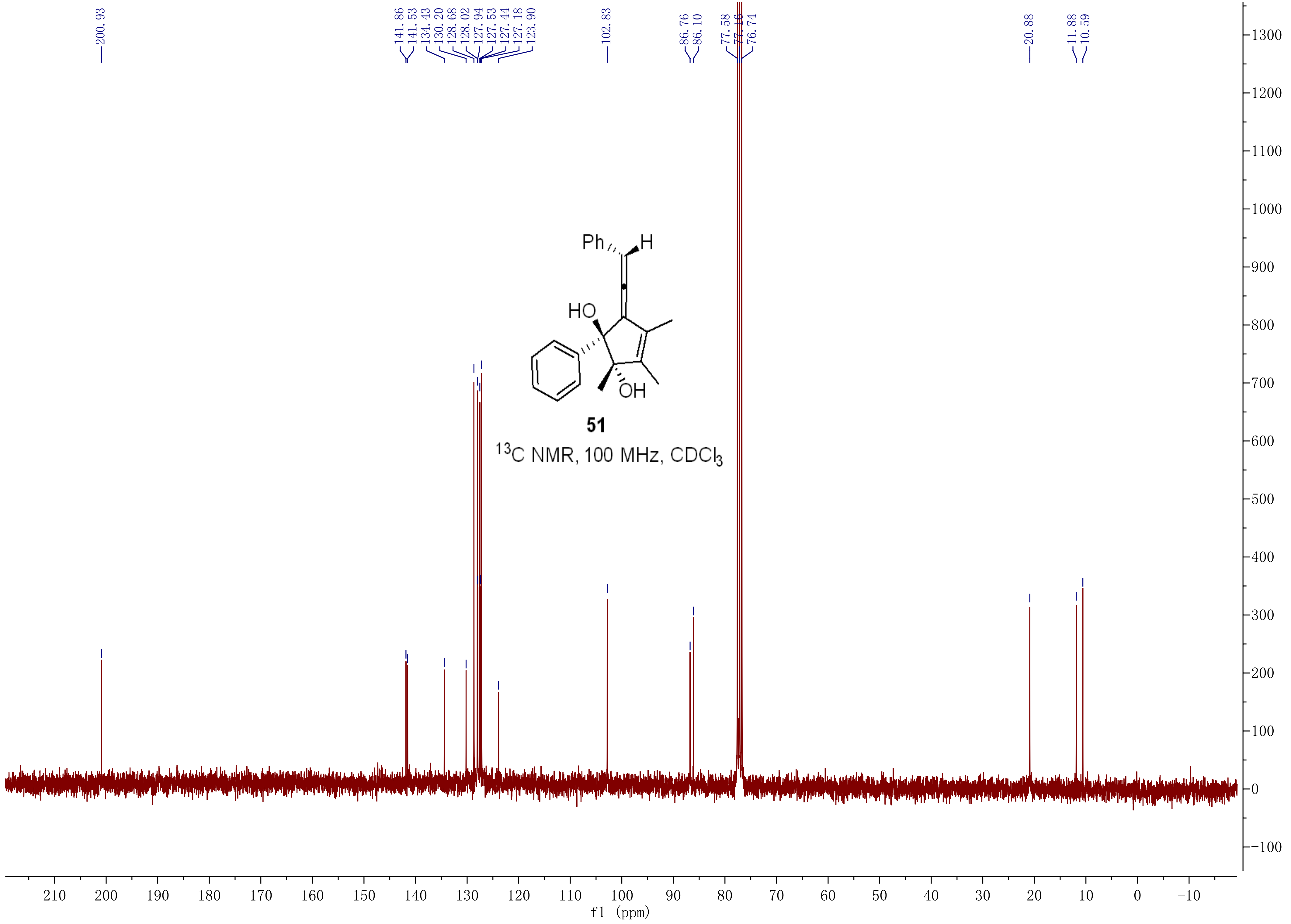

Supplementary Figure 138. ^{13}C NMR of compound (-)-51.

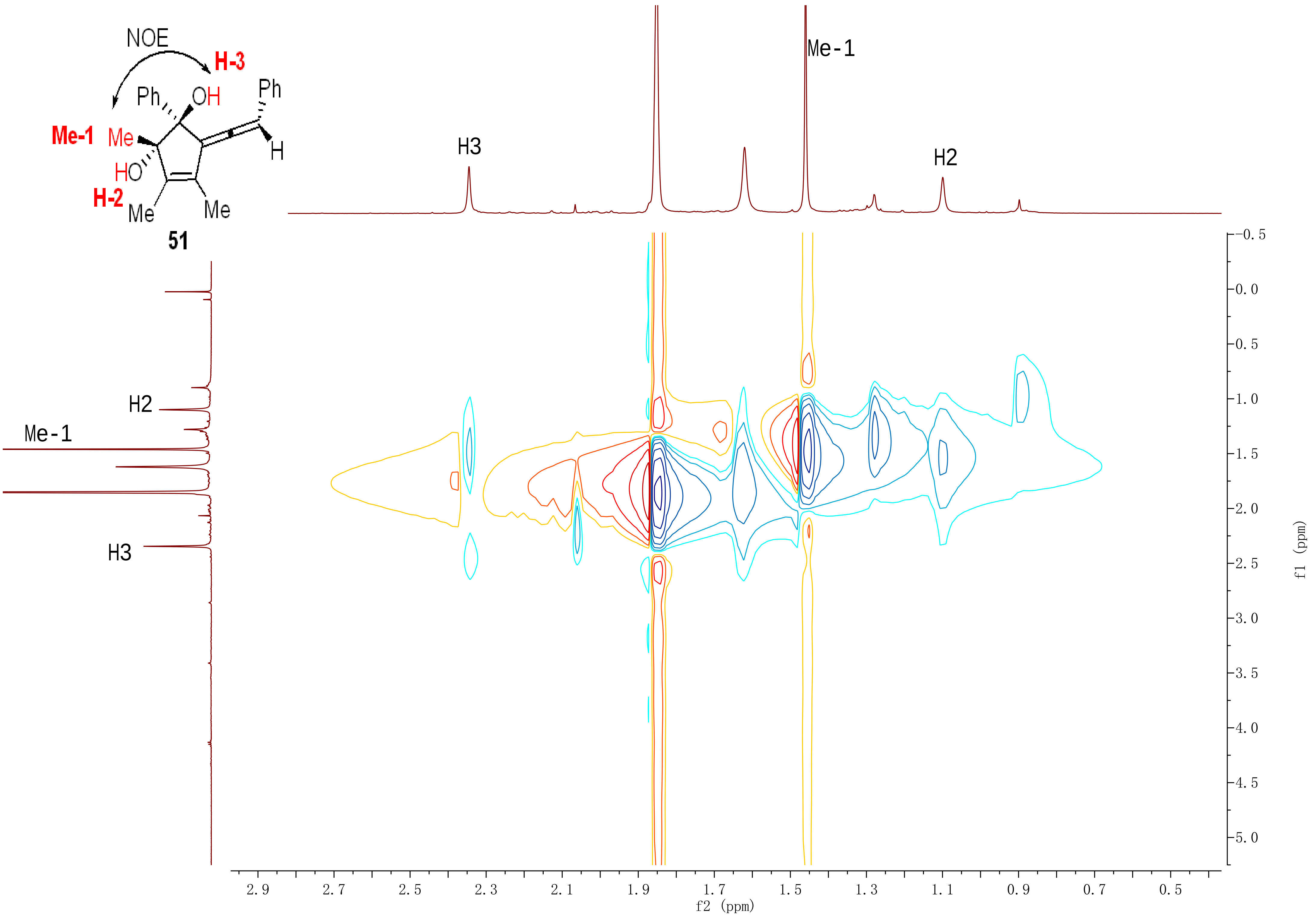

Supplementary Figure 139. NOE of compound (-)-51.

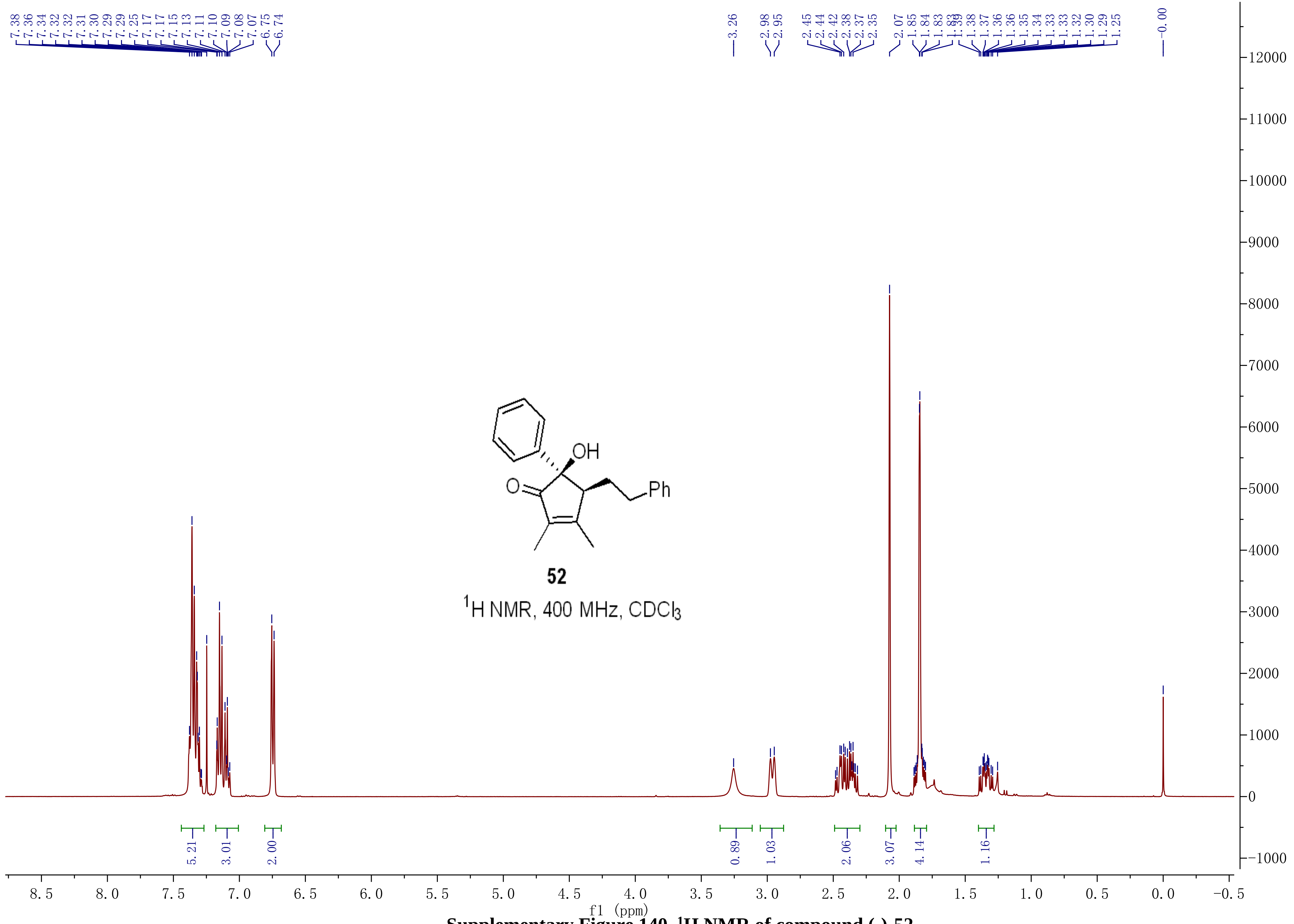

Supplementary Figure 140. ^{1}H NMR of compound (-)-52.

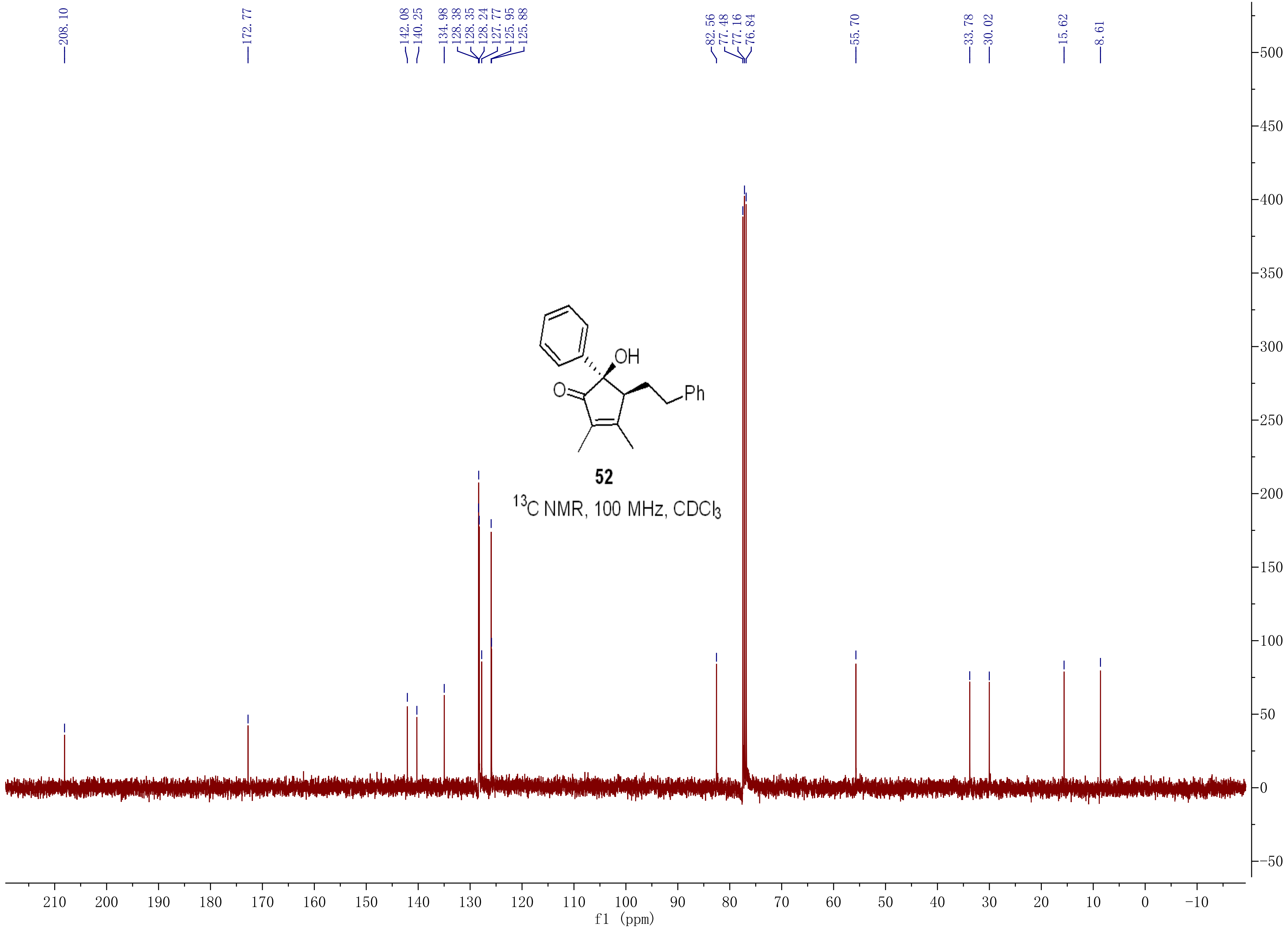

Supplementary Figure 141. ^{13}**C NMR of compound (-)-52.**

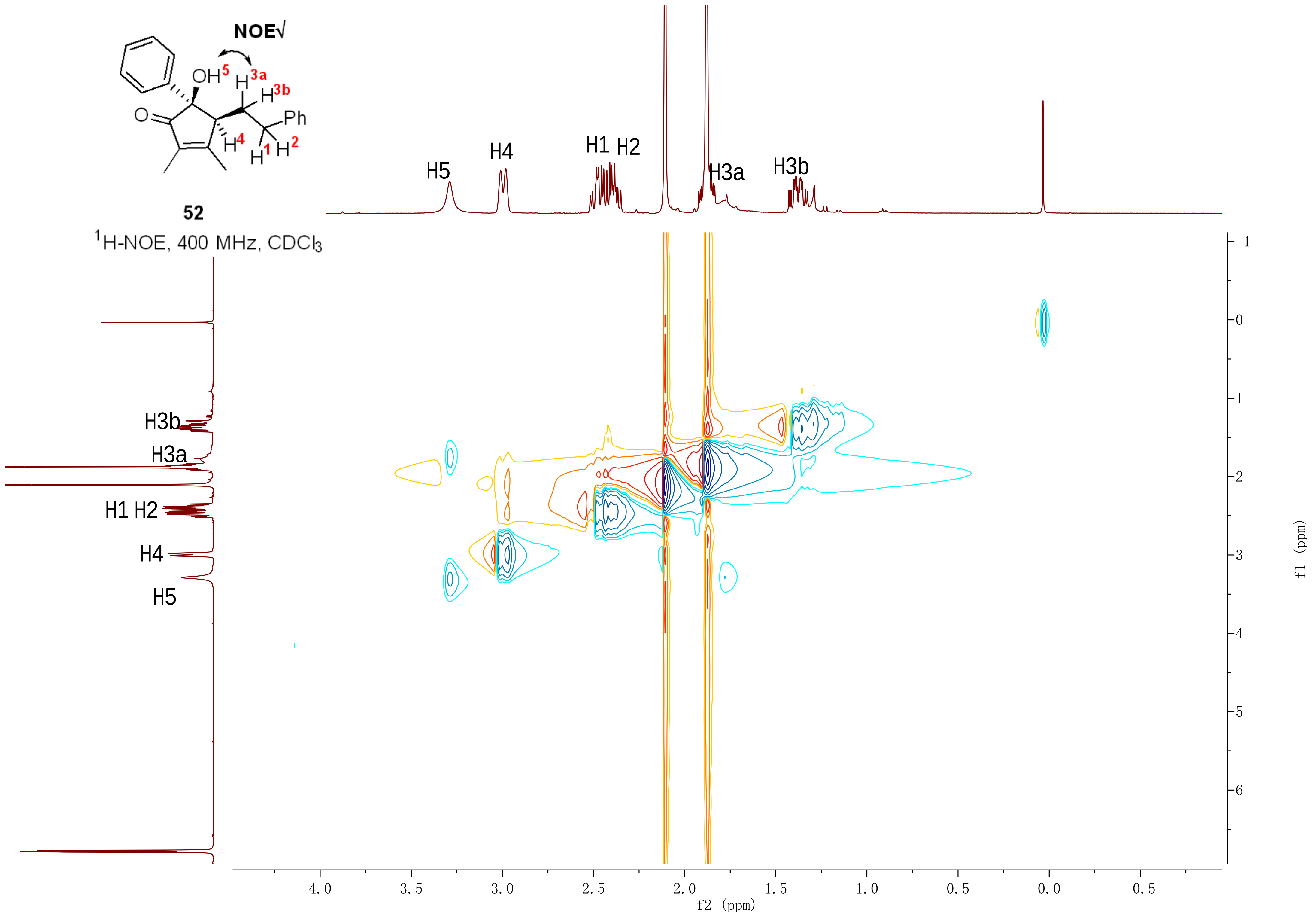

Supplementary Figure 142. NOE of compound (-)-52.

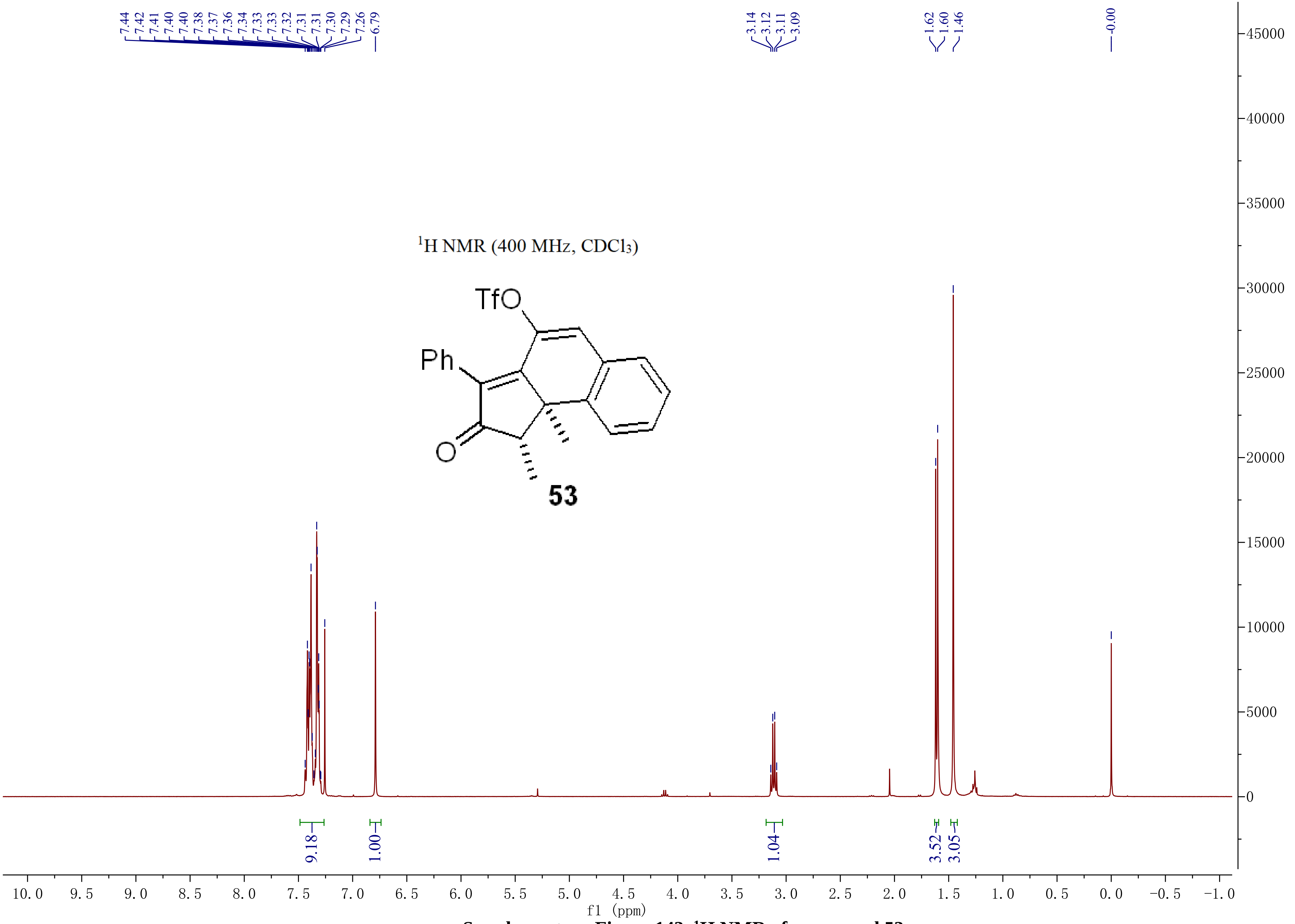

Supplementary Figure 143. ^{1}H NMR of compound 53.

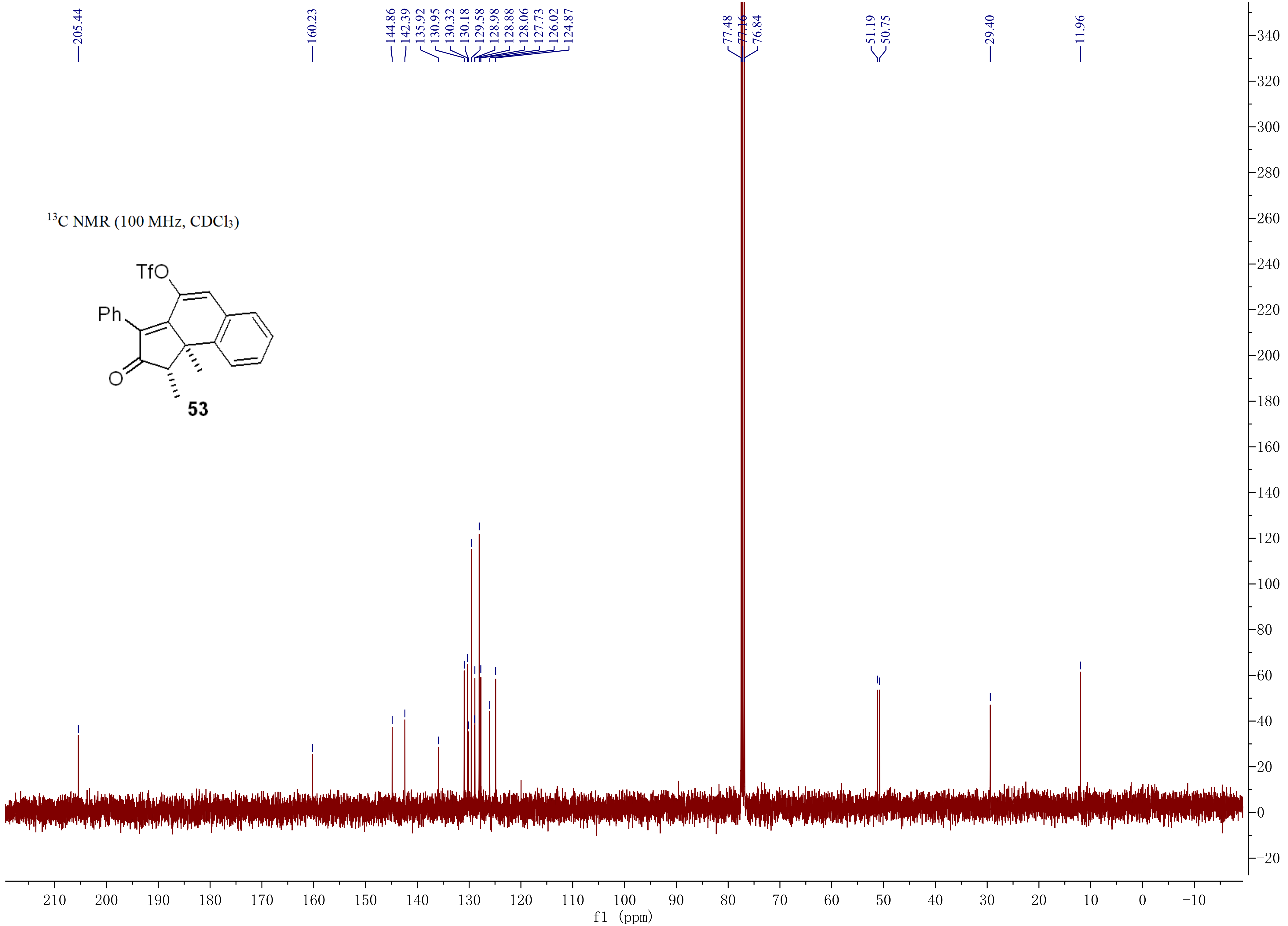

Supplementary Figure 144. ^{13}C NMR of compound 53.

Racemic sample 4: HPLC (Daicel Chiralpak IC column (hexane/iPrOH = 70:30, flow rate: 1.0 mL/min, λ= 254 nm)

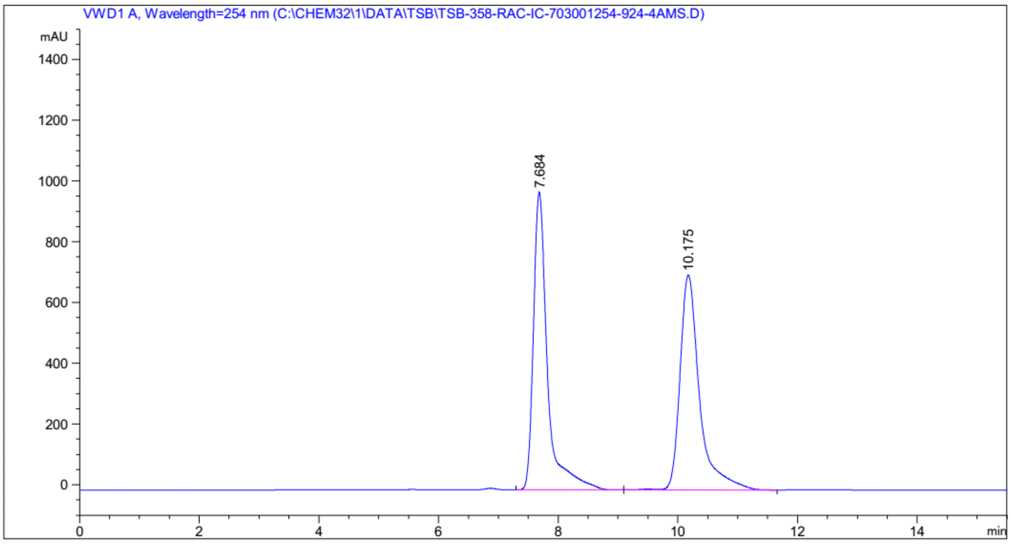

Peak Name	RT [min]	Type	width [min]	Area [mAU*s]	Height [mAu]	Area ratio %
1	7.684	BB	0.2425	1.59262e4	980.49811	49.8295
2	10.175	VB R	0.3383	1.60352e4	708.10919	50.1705

Enantioenriched sample (-)-4:

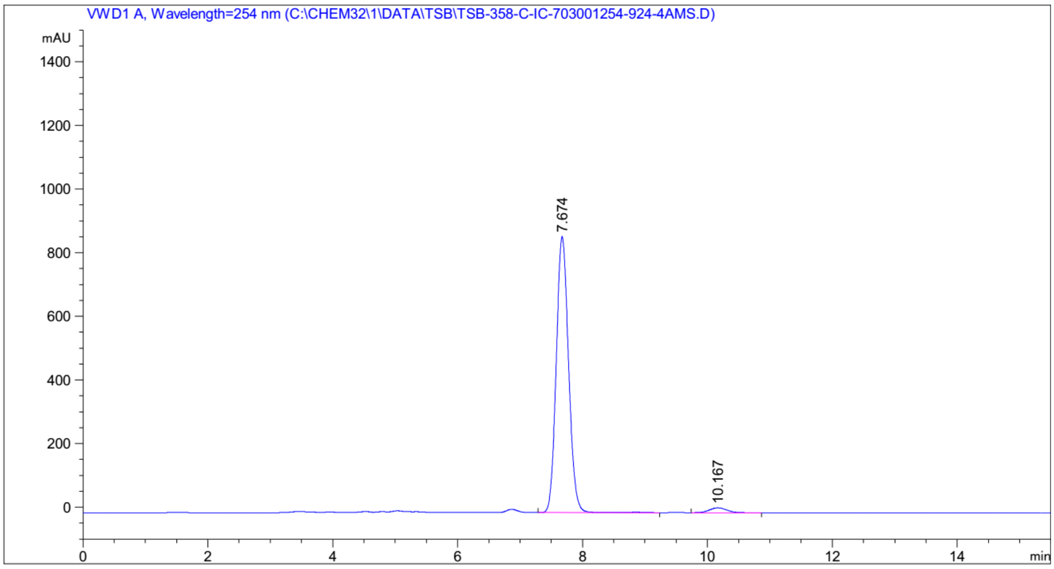

Peak Name	RT [min]	Type	width [min]	Area [mAU*s]	Height [mAu]	Area ratio %
1	7.674	BV R	0.2127	1.18735e4	867.63147	97.5748
2	10.167	BB	0.2984	295.11334	15.41461	2.4252

Supplementary Figure 145. HPLC analysis of compound (-)-4.

Racemic sample 5: HPLC (Daicel Chiralpak IC column (hexane/iPrOH = 50:50, flow rate: 1.0 mL/min, λ= 254 nm)

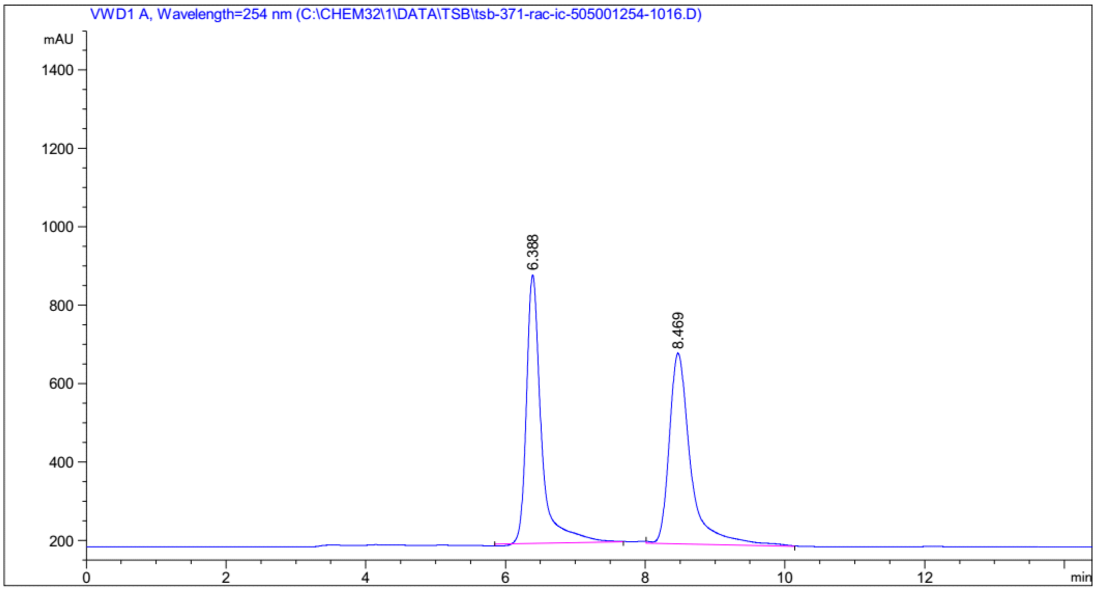

Peak Name	RT [min]	Type	width [min]	Area [mAU*s]	Height [mAu]	Area ratio %
1	6.388	MM	0.2482	1.02080e4	685.60553	49.5085
2	8.469	MM	0.3568	1.04107e4	486.25751	50.4915

Enantioenriched sample (-)-5:

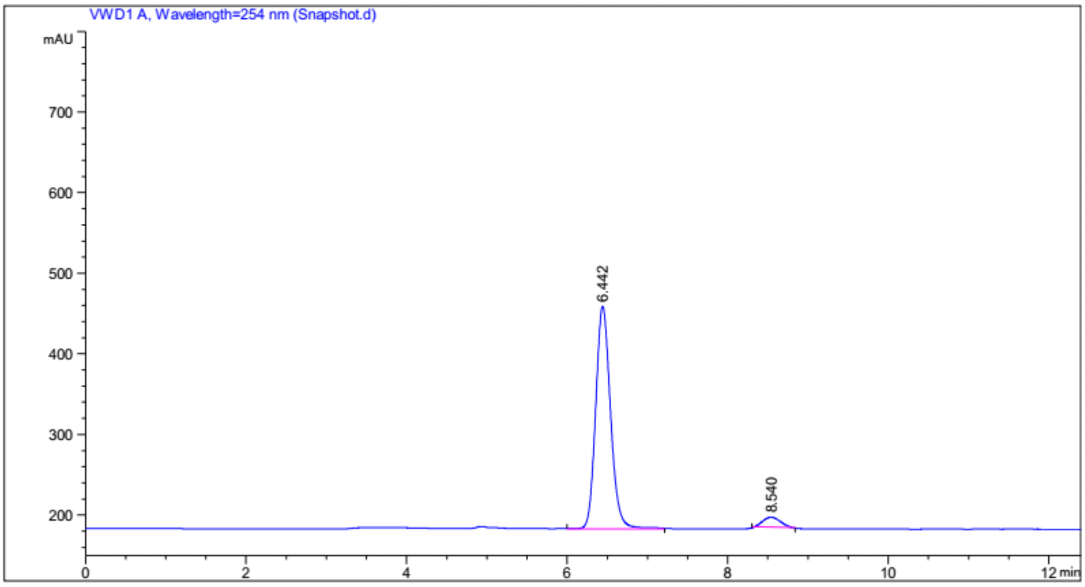

Peak Name	RT [min]	Type	width [min]	Area [mAU*s]	Height [mAu]	Area ratio %
1	6.442	MM	0.2140	3545.34009	276.06555	94.9456
2	8.540	MM	0.2531	188.73390	12.42898	5.0544

Supplementary Figure 146. HPLC analysis of compound (-)-5.

Racemic sample 6: HPLC (Daicel Chiralpak IC column (hexane/iPrOH = 70:30, flow rate: 1.0 mL/min, λ= 254 nm)

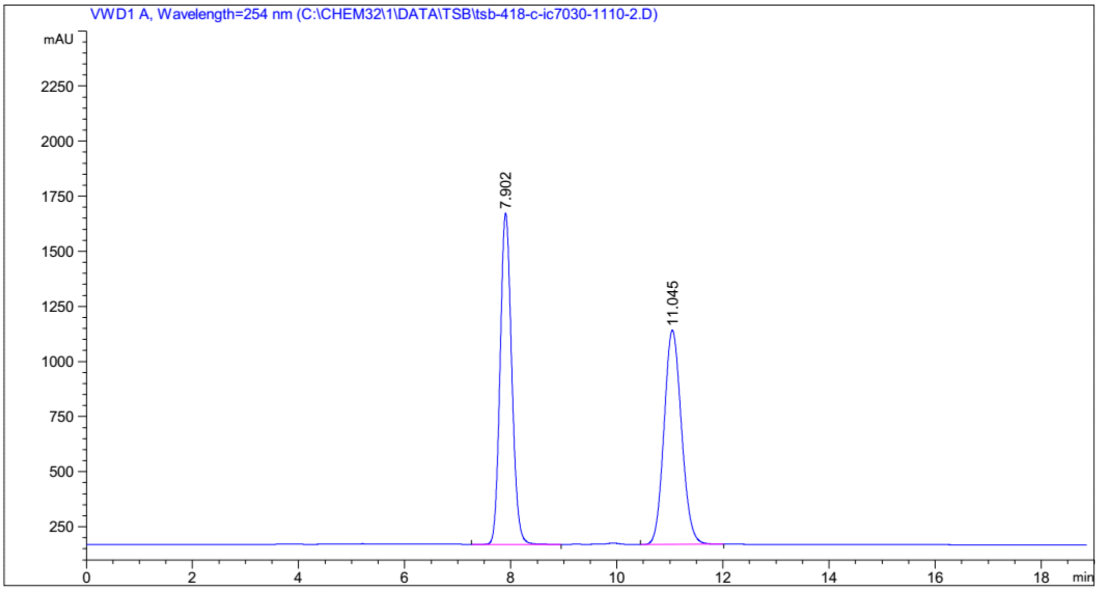

Peak Name	RT [min]	Type	width [min]	Area [mAU*s]	Height [mAu]	Area ratio %
1	7.902	BB	0.2346	2.27645e4	1504.22815	50.5972
2	11.045	BB	0.3559	2.22271e4	971.64020	49.4028

Enantioenriched sample (-)-6

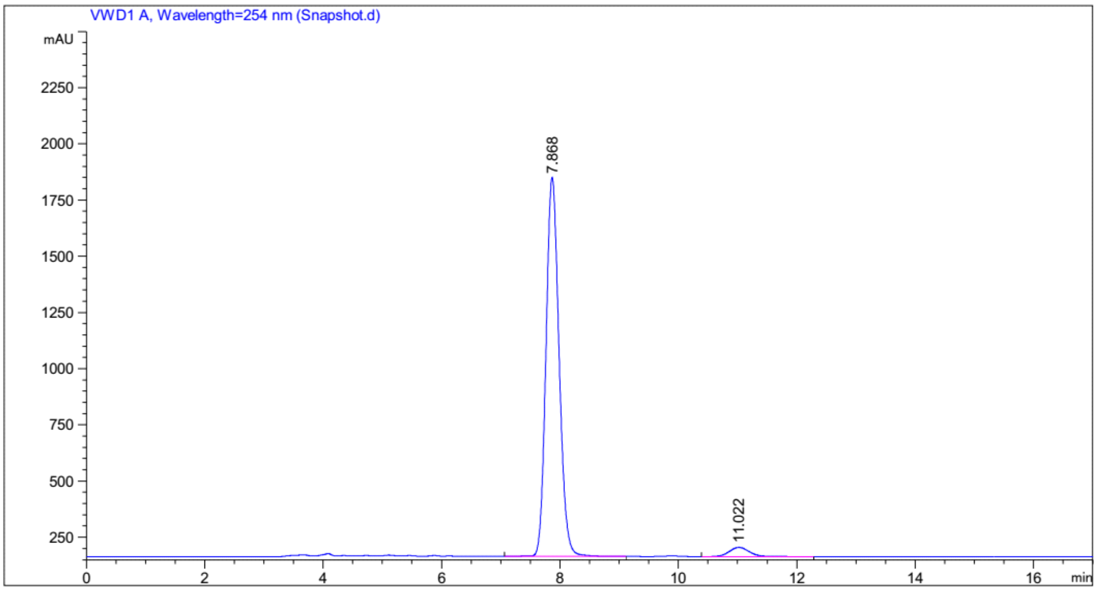

Peak Name	RT [min]	Type	width [min]	Area [mAU*s]	Height [mAu]	Area ratio %
1	7.868	VB R	0.2364	2.58147e4	1686.27466	96.3650
2	11.022	BB	0.3657	973.75201	41.04969	3.6350

Supplementary Figure 147. HPLC analysis of compound (-)-6.

Racemic sample 7: HPLC (Daicel Chiralpak IC column, hexane/iPrOH = 60:40, flow rate: 1.0 mL/min, λ= 254 nm)

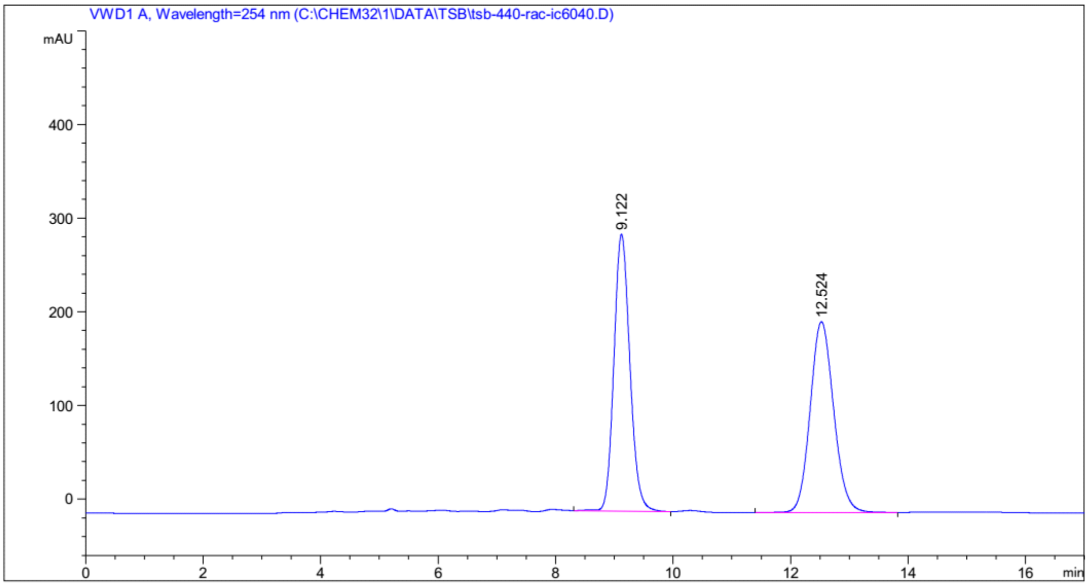

Peak Name	RT [min]	Type	width [min]	Area [mAU*s]	Height [mAu]	Area ratio %
1	9.122	VB R	0.2922	5581.82324	295.75531	49.6235
2	12.524	BB	0.4303	5666.53418	203.86111	50.3765

Enantioenriched sample (-)-7:

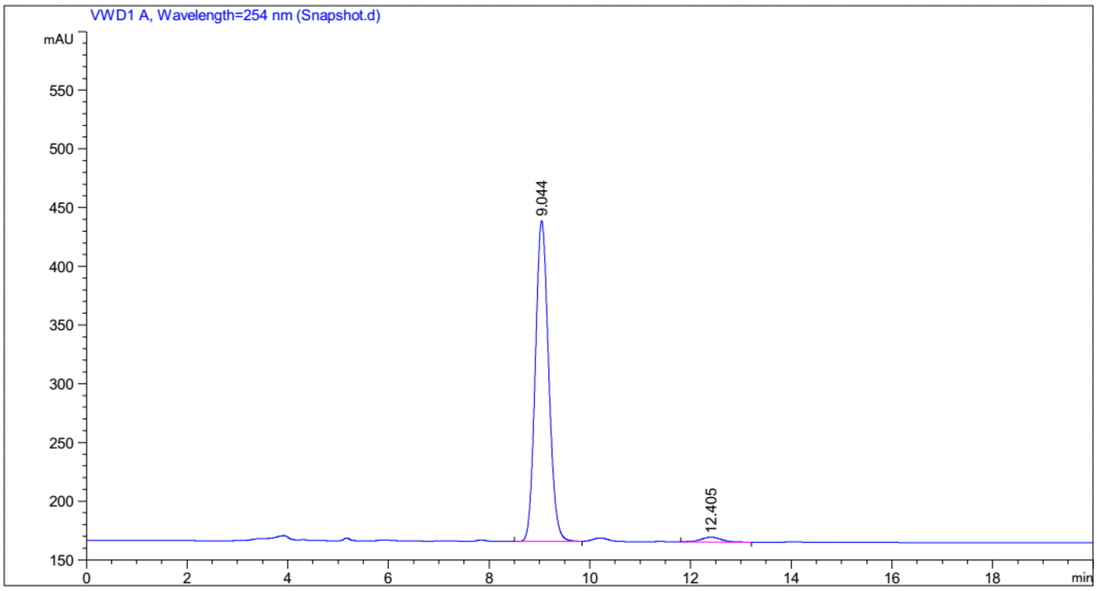

Peak Name	RT [min]	Type	width [min]	Area [mAU*s]	Height [mAu]	Area ratio %
1	9.044	BB	0.2850	5014.70996	273.32541	97.9193
2	12.405	BB	0.4153	106.56010	3.95561	2.0807

Supplementary Figure 148. HPLC analysis of compound (-)-7.

Racemic sample 8: HPLC (Daicel Chiralpak IC column (hexane/iPrOH = 60:40, flow rate: 1.0 mL/min, λ= 254 nm)

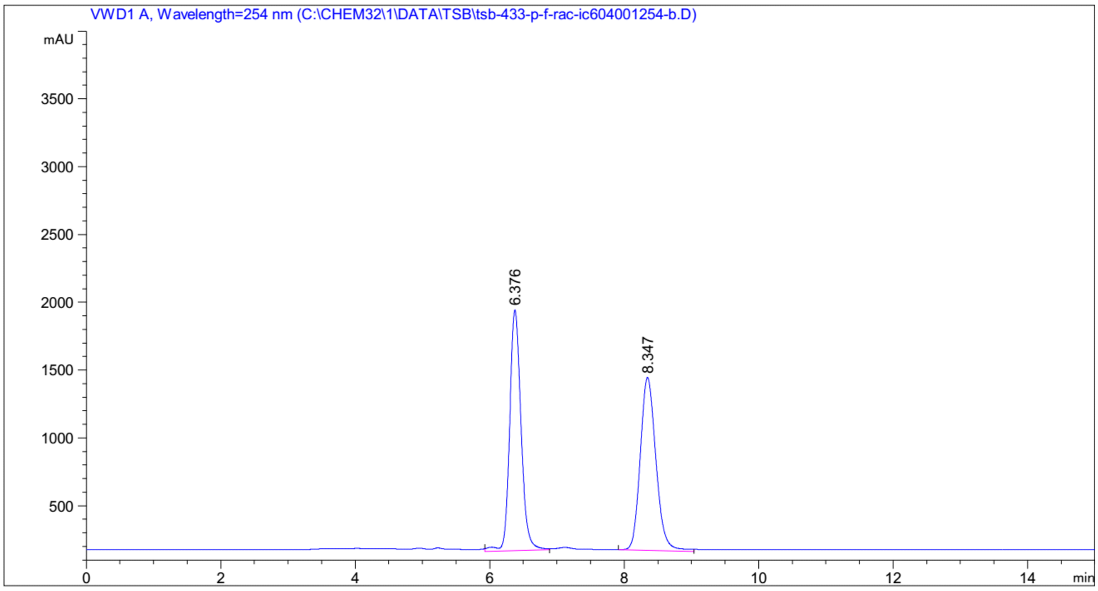

Peak Name	RT [min]	Type	width [min]	Area [mAU*s]	Height [mAu]	Area ratio %
1	6.376	MM	0.1961	2.08968e4	1775.73279	50.2161
2	8.347	MM	0.2705	2.07169e4	1276.60315	49.7839

Enantioenriched sample (-)-8:

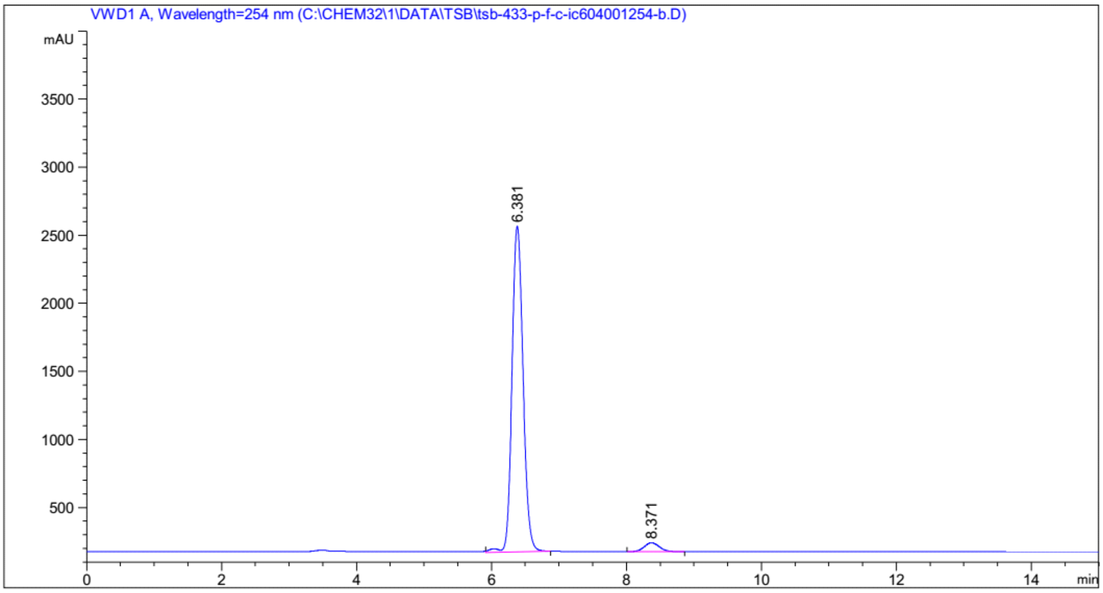

Peak Name	RT [min]	Type	width [min]	Area [mAU*s]	Height [mAu]	Area ratio %
1	6.381	MM	0.1898	2.72366e4	2391.43188	96.4274
2	8.371	BB	0.2407	1009.10962	65.16249	3.5726

Supplementary Figure 149. HPLC analysis of compound (-)-8.

Racemic sample 9: HPLC (Daicel Chiralpak IC column, hexane/iPrOH = 70:30, flow rate: 1.0 mL/min, λ= 254 nm)

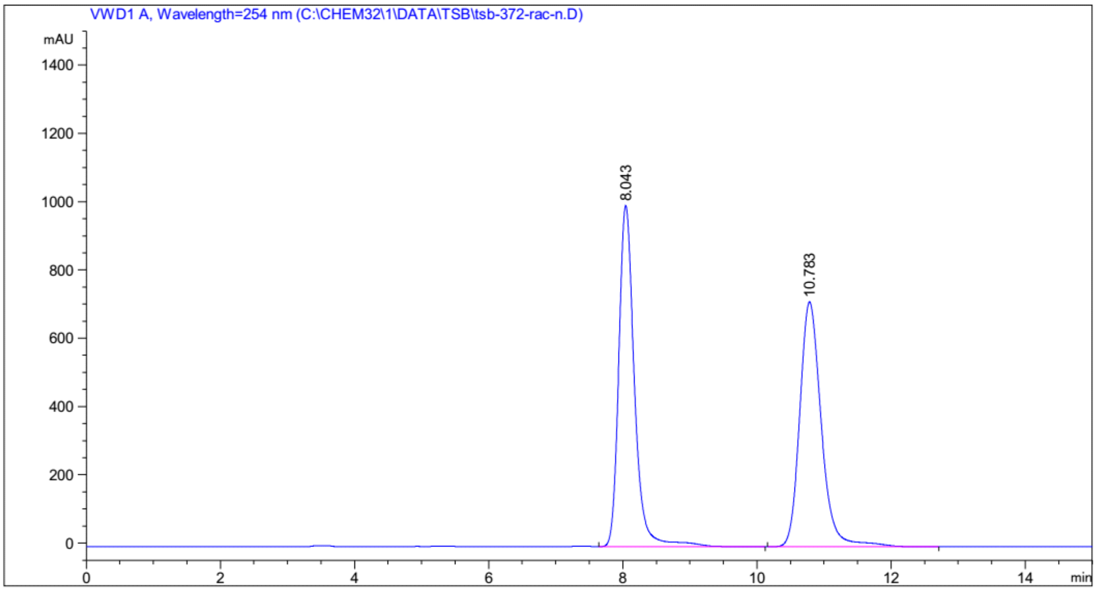

Peak Name	RT [min]	Type	width [min]	Area [mAU*s]	Height [mAu]	Area ratio %
1	8.043	VB	0.2472	1.62011e4	999.09375	50.3210
2	10.783	BB	0.3411	1.59944e4	717.54413	49.6790

Enantioenriched sample (-)-9:

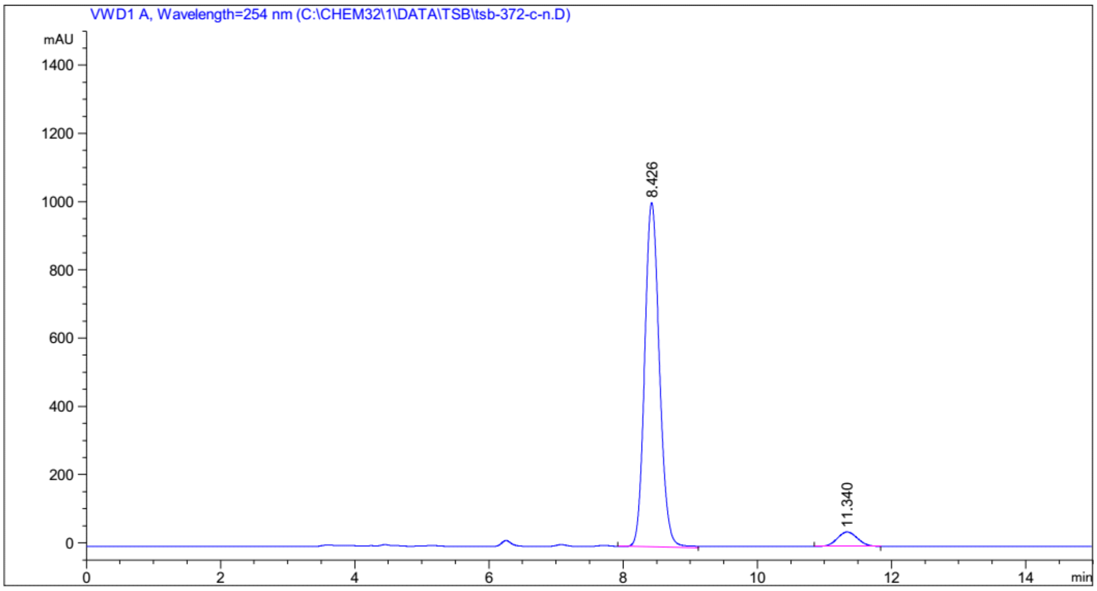

Peak Name	RT [min]	Type	width [min]	Area [mAU*s]	Height [mAu]	Area ratio %
1	8.426	MM	0.2541	1.53899e4	1009.35229	94.7946
2	11.340	MM	0.3387	845.09778	41.58365	5.2054

Supplementary Figure 150. HPLC analysis of compound (-)-9.

Racemic sample 10: HPLC (Daicel Chiralpak IC column, hexane/iPrOH = 60:40, flow rate: 1.0 mL/min, λ= 254 nm)

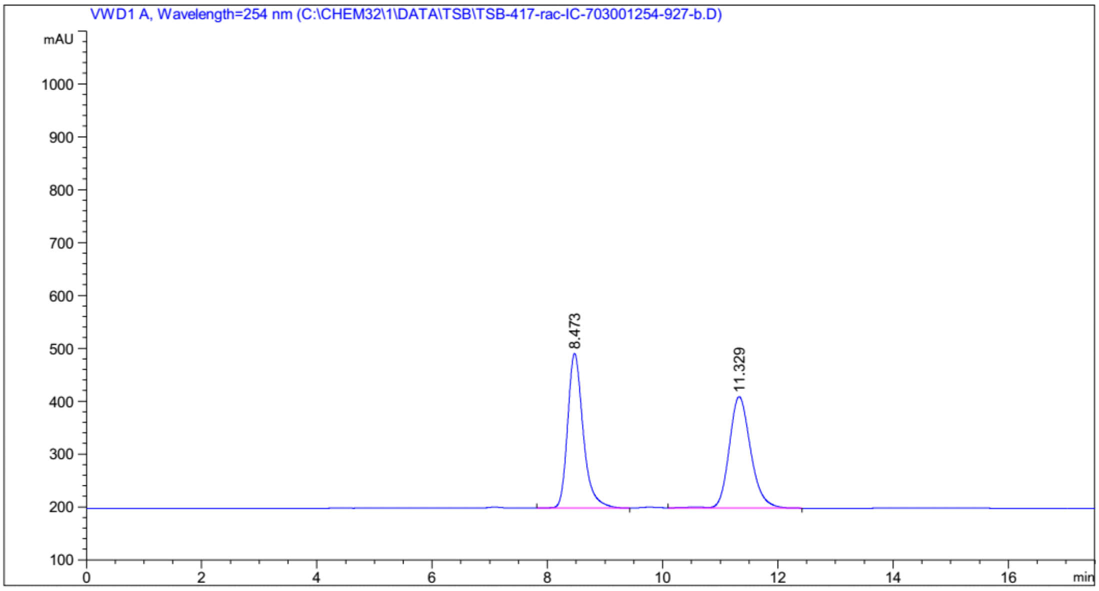

Peak Name	RT [min]	Type	width [min]	Area [mAU*s]	Height [mAu]	Area ratio %
1	8.473	BB	0.2849	5459.13525	292.24261	50.1087
2	11.329	VB R	0.3961	5435.45264	210.62904	49.8913

Enantioenriched sample (-)-10:

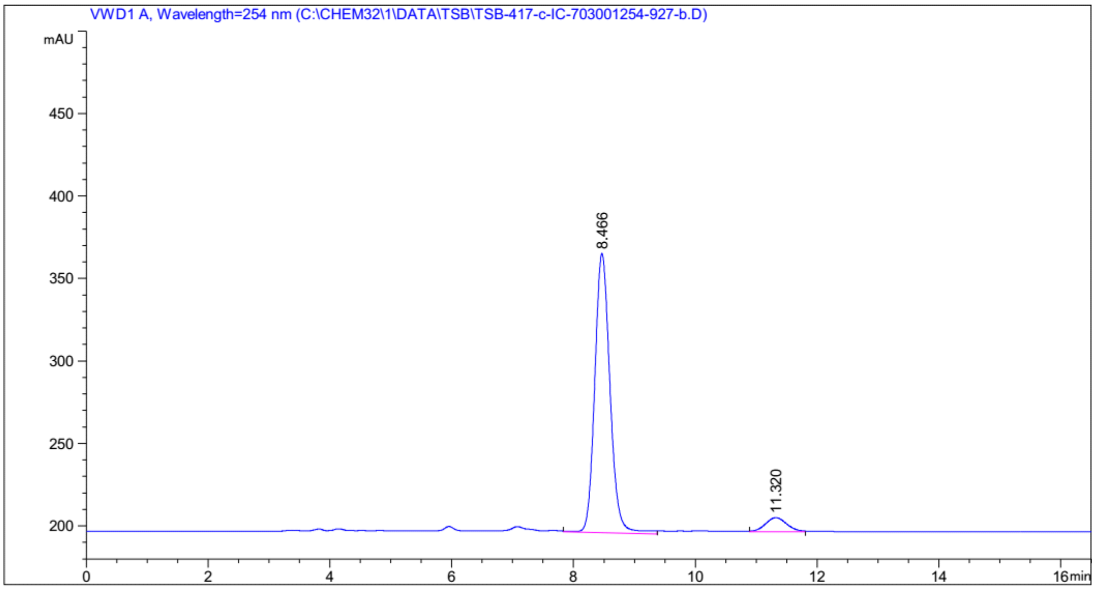

Peak Name	RT [min]	Type	width [min]	Area [mAU*s]	Height [mAu]	Area ratio %
1	8.466	MM	0.2899	2945.26953	169.32851	93.2661
2	11.320	MM	0.4118	212.65031	8.60645	6.7339

Supplementary Figure 151. HPLC analysis of compound (-)-10.

Racemic sample 11: HPLC (Daicel Chiralpak IC column, hexane/iPrOH = 70:30, flow rate: 1.0 mL/min, λ= 254 nm)

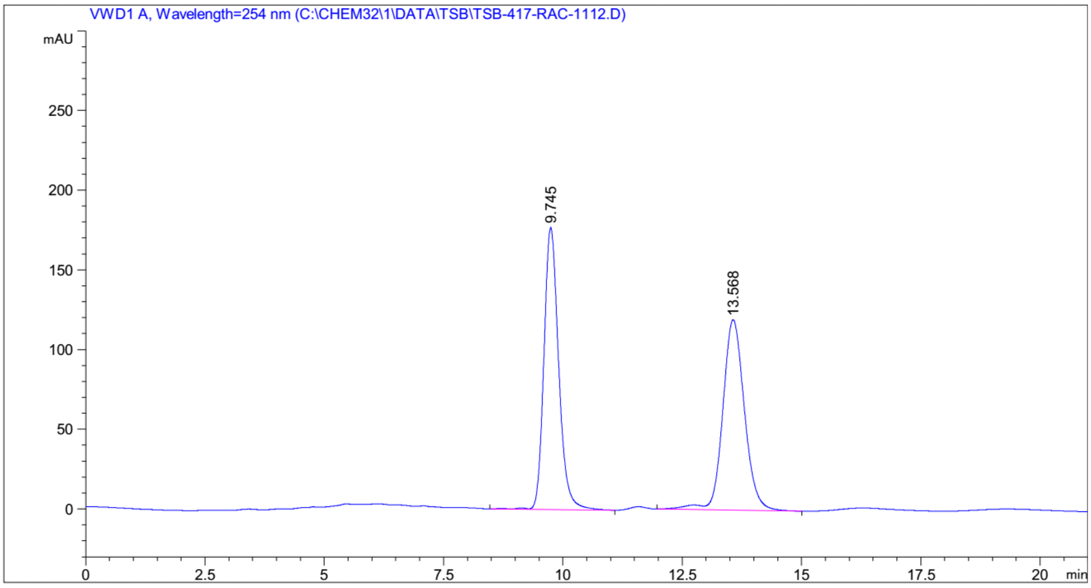

Peak Name	RT [min]	Type	width [min]	Area [mAU*s]	Height [mAu]	Area ratio %
1	9.745	VB R	0.3293	3795.32275	176.90773	50.0500
2	13.568	VB R	0.4794	3787.74048	119.47675	49.9500

Enantioenriched sample (-)-11:

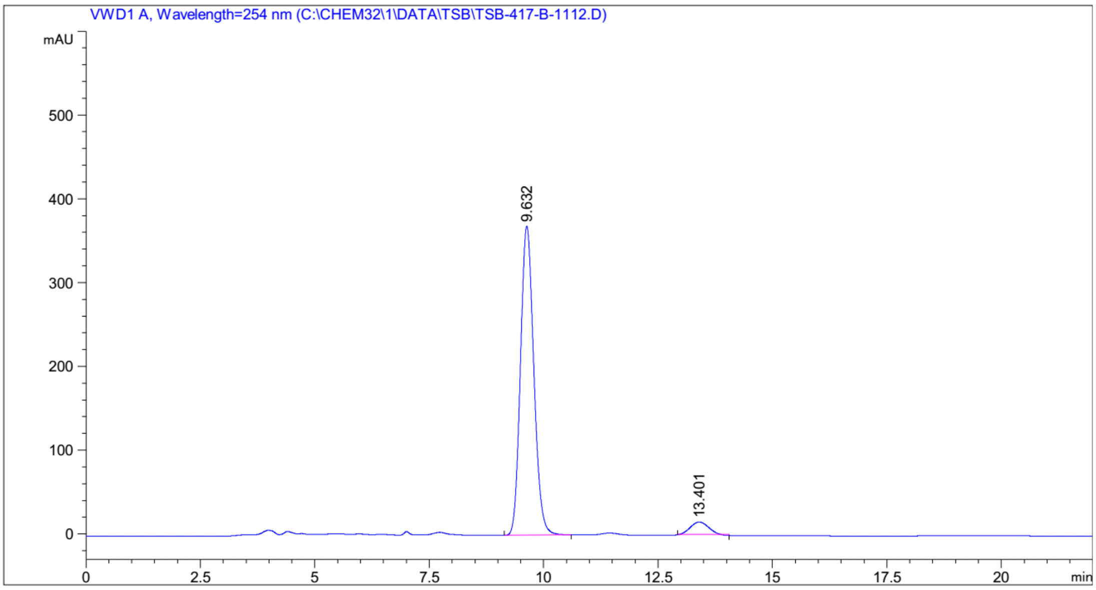

Peak Name	RT [min]	Type	width [min]	Area [mAU*s]	Height [mAu]	Area ratio %
1	9.632	BB	0.3131	7467.00830	369.01715	95.1396
2	13.401	MM	0.4397	381.47000	14.45805	4.8604

Supplementary Figure 152. HPLC analysis of compound (-)-11.

Racemic sample 12: HPLC (Daicel Chiralpak IC column, hexane/iPrOH = 70:30, flow rate: 1.0 mL/min, λ= 254 nm)

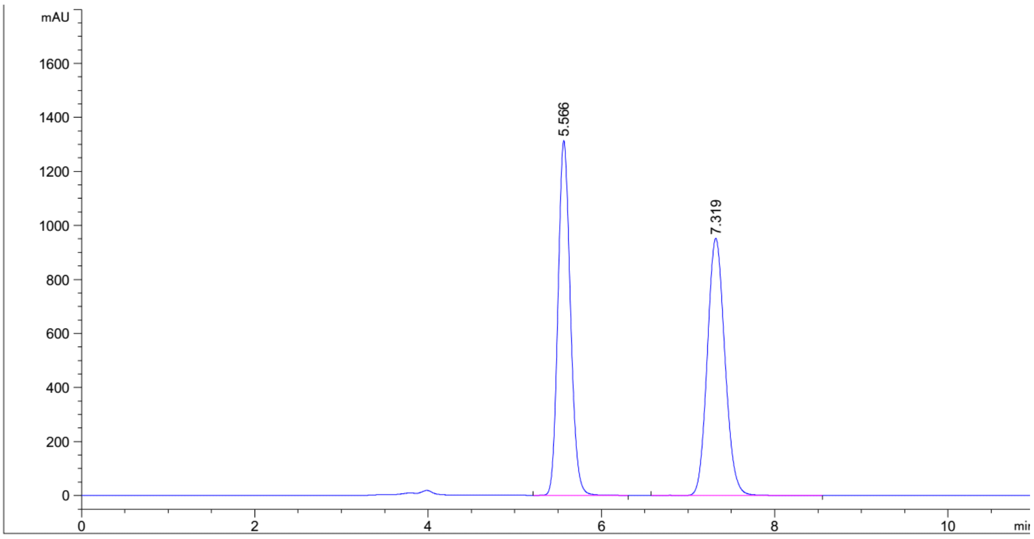

Peak Name	RT [min]	Type	width [min]	Area [mAU*s]	Height [mAu]	Area ratio %
1	5.566	BB	0.1526	1.30108e4	1313.63513	49.2223
2	7.319	VB R	0.2184	1.34220e4	952.66248	50.7777

Enantioenriched sample (-)-12:

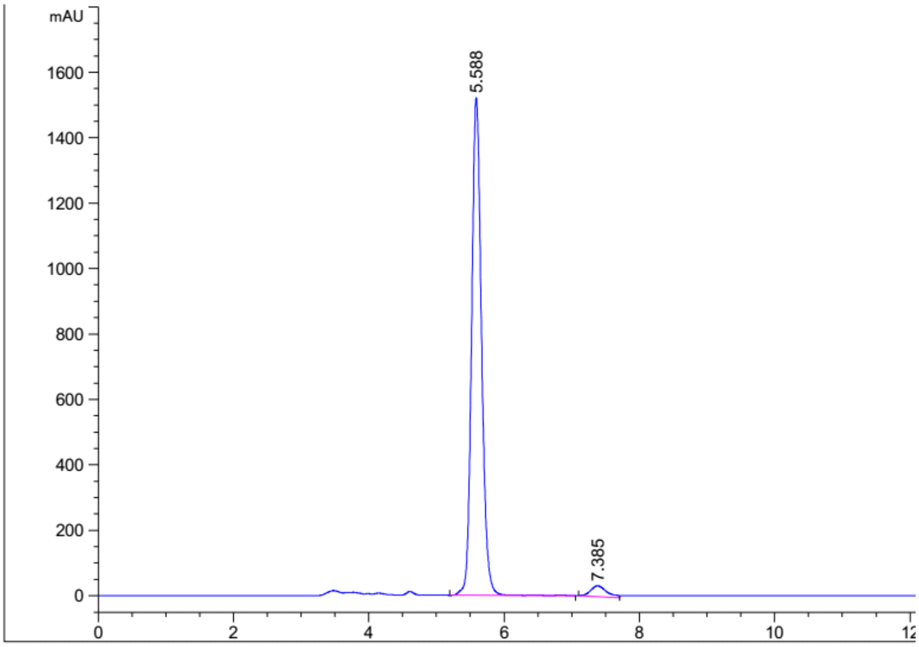

Peak Name	RT [min]	Type	width [min]	Area [mAU*s]	Height [mAu]	Area ratio %
1	5.588	BV R	0.1566	1.54711e4	1521.60303	96.6137
2	7.385	MM	0.2754	542.26178	32.81590	3.3863

Supplementary Figure 153. HPLC analysis of compound (-)-12.

Racemic sample 13: HPLC (Daicel Chiralpak IC column, hexane/iPrOH = 70:30, flow rate: 1.0 mL/min, λ= 254 nm)

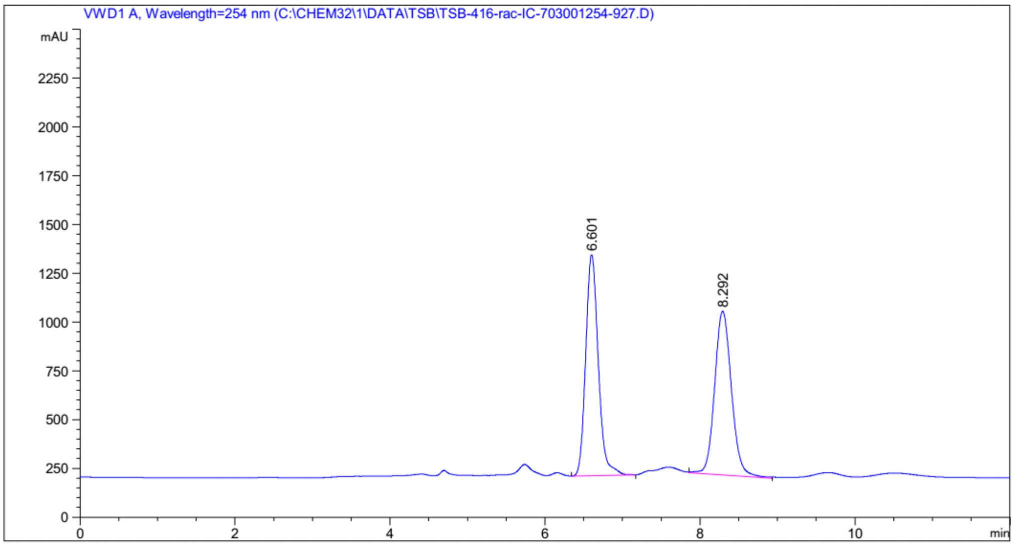

Peak Name	RT [min]	Type	width [min]	Area [mAU*s]	Height [mAu]	Area ratio %
1	6.601	BV R	0.1734	1.27550e4	1132.57593	50.2484
2	8.292	MM	0.2509	1.26289e4	839.00031	49.7516

Enantioenriched sample (-)-13:

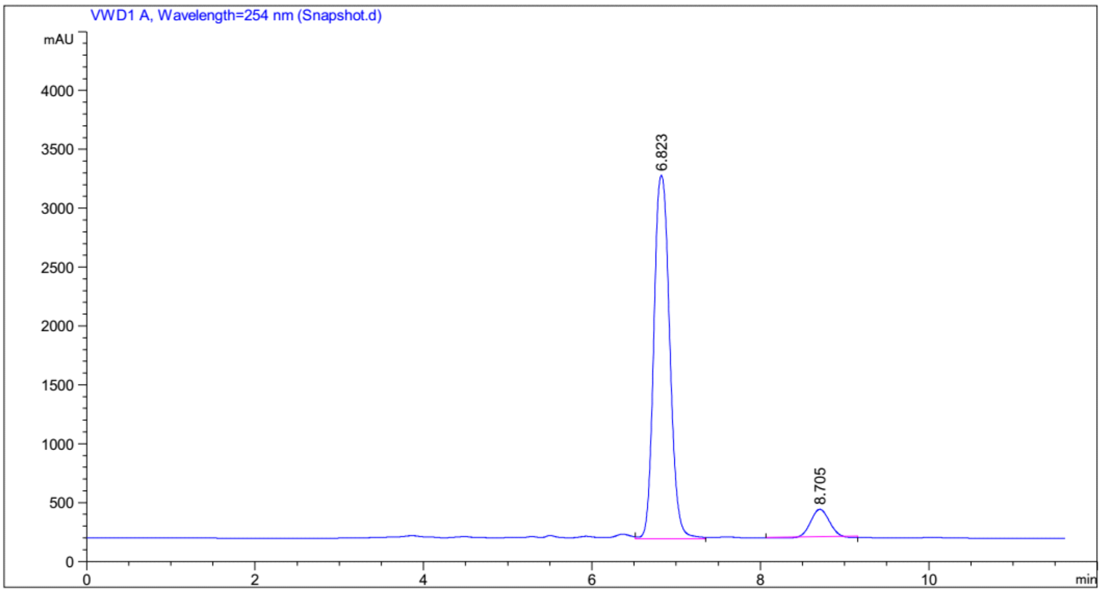

Peak Name	RT [min]	Type	width [min]	Area [mAU*s]	Height [mAu]	Area ratio %
1	6.823	MM	0.2182	4.04312e4	3088.12671	92.1084
2	8.705	MM	0.2494	3464.04126	231.46683	7.8916

Supplementary Figure 154. HPLC analysis of compound (-)-13.

Racemic sample 14: HPLC (Daicel Chiralpak IC column, hexane/iPrOH = 70:30, flow rate: 1.0 mL/min, λ= 254 nm)

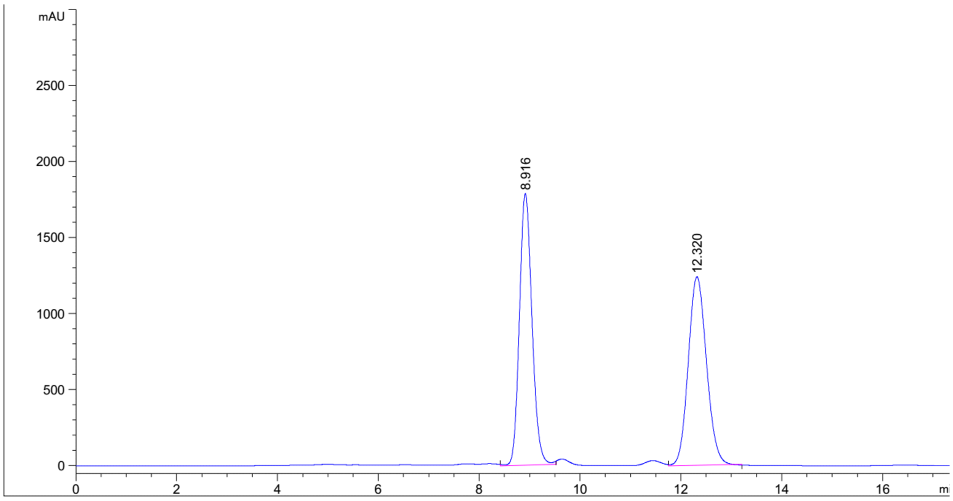

Peak Name	RT [min]	Type	width [min]	Area [mAU*s]	Height [mAu]	Area ratio %
1	8.916	MM	0.2891	3.09818e4	1786.06580	49.8827
2	12.320	MM	0.4187	3.11276e4	1239.20178	50.1173

Enantioenriched sample (-)-14:

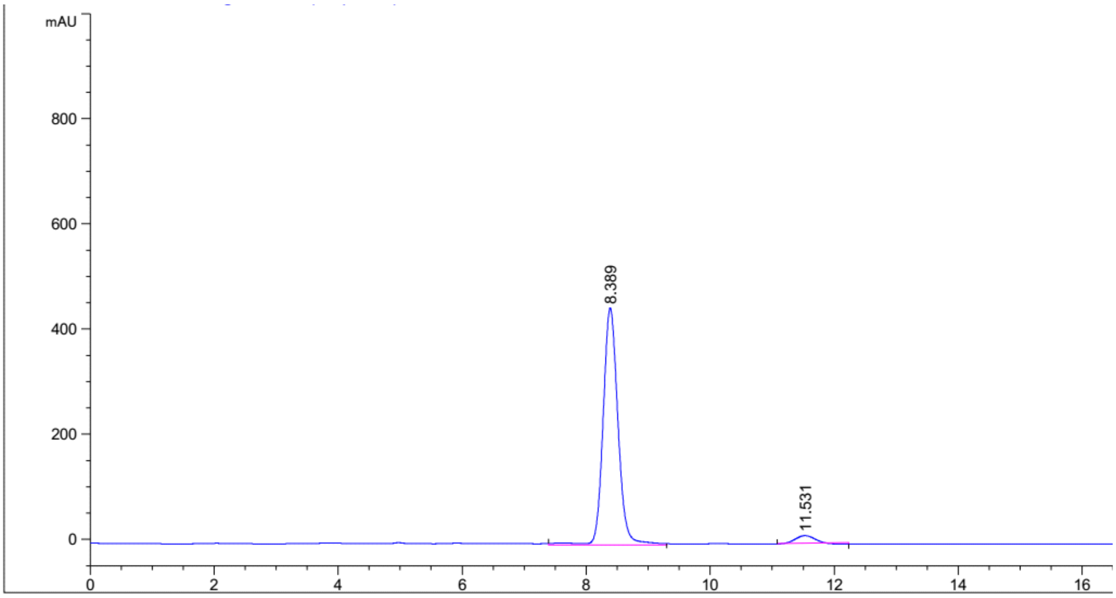

Peak Name	RT [min]	Type	width [min]	Area [mAU*s]	Height [mAu]	Area ratio %
1	8.389	MM	0.2783	7521.44482	450.38080	96.0359
2	11.531	MM	0.3521	310.46472	14.69678	3.9641

Supplementary Figure 155. HPLC analysis of compound (-)-14.

Racemic sample 15: HPLC (Daicel Chiralpak IC column, hexane/iPrOH = 60:40, flow rate: 1.0 mL/min, λ= 254 nm)

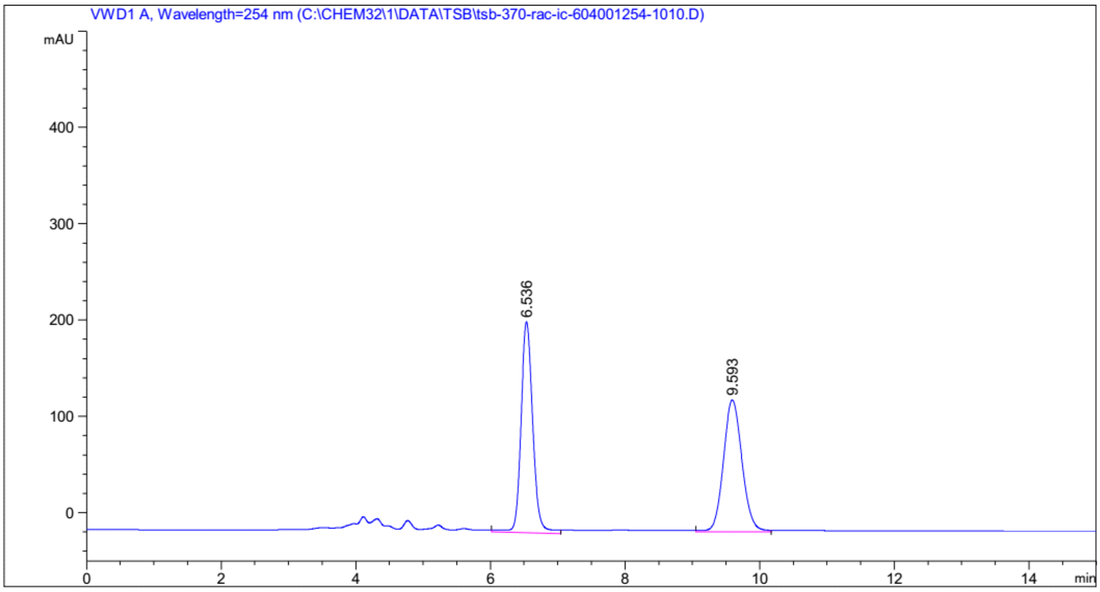

Peak Name	RT [min]	Type	width [min]	Area [mAU*s]	Height [mAu]	Area ratio %
1	6.536	MM	0.2019	2652.04956	218.97559	50.2380
2	9.593	MM	0.3203	2626.92212	136.67316	49.7620

Enantioenriched sample (-)-15:

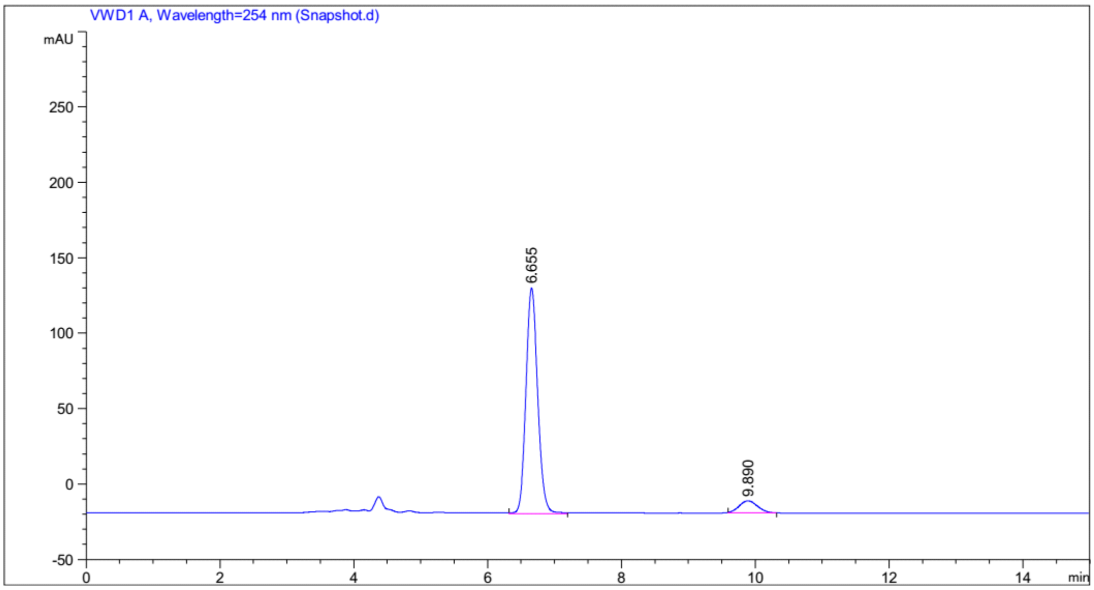

Peak Name	RT [min]	Type	width [min]	Area [mAU*s]	Height [mAu]	Area ratio %
1	6.655	MM	0.2035	1827.01611	149.63739	92.3458
2	9.890	MM	0.3181	151.43431	7.93525	7.6542

Supplementary Figure 156. HPLC analysis of compound (-)-15.

Racemic sample 16: HPLC (Daicel Chiralpak IC column, hexane/iPrOH = 50:50, flow rate: 1.0 mL/min, λ= 254 nm)

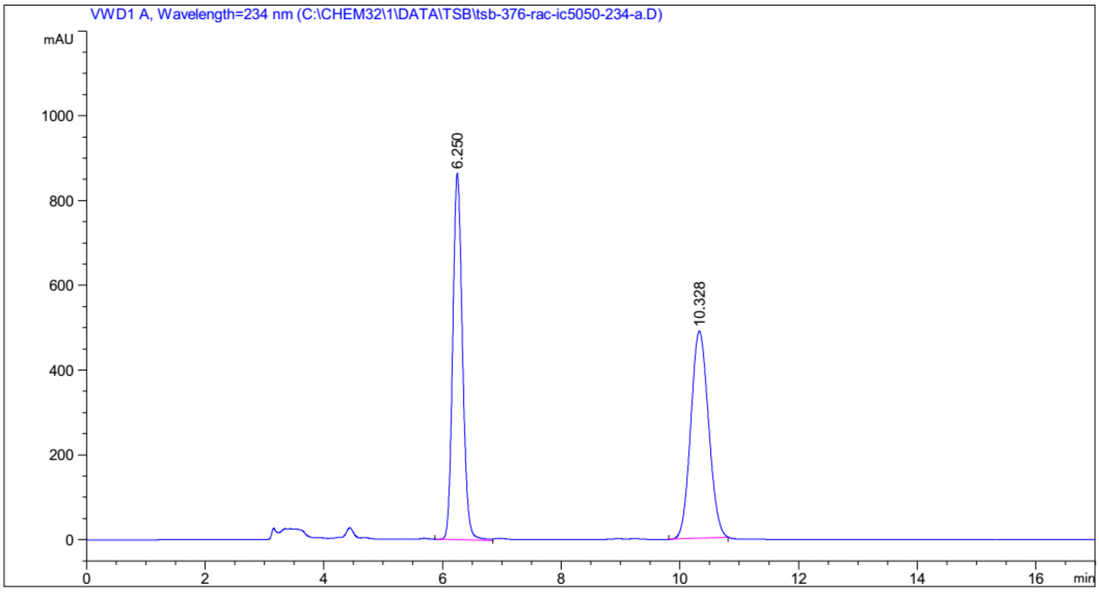

Peak Name	RT [min]	Type	width [min]	Area [mAU*s]	Height [mAu]	Area ratio %
1	6.250	MM	0.1888	9793.12500	864.65063	49.0971
2	10.328	MM	0.3460	1.01533e4	489.03418	50.9029

Enantioenriched sample (-)-16:

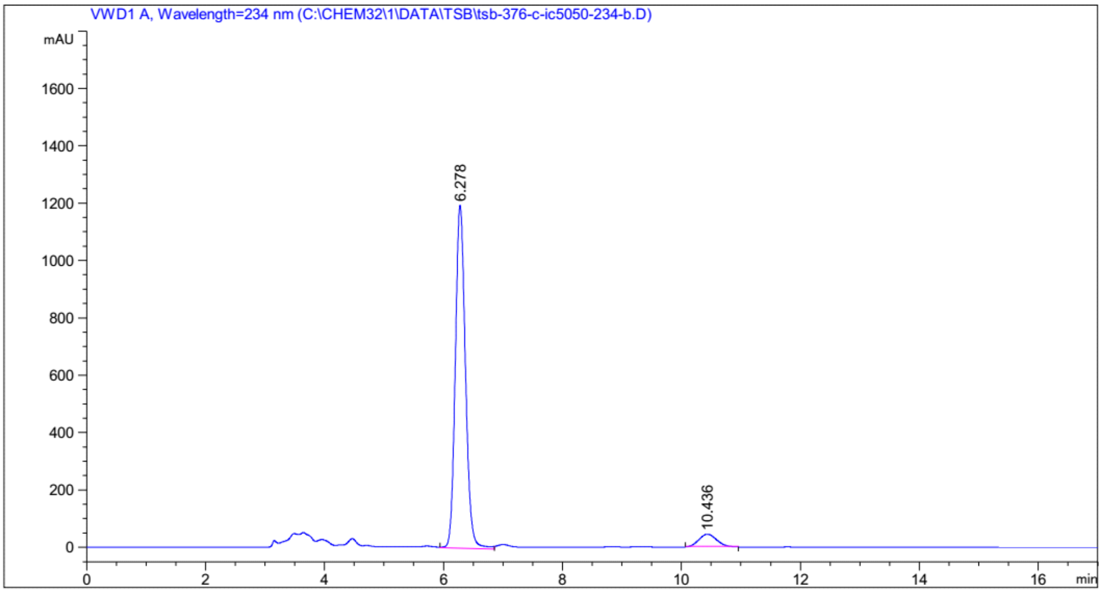

Peak Name	RT [min]	Type	width [min]	Area [mAU*s]	Height [mAu]	Area ratio %
1	6.278	MM	0.1964	1.40844e4	1195.49365	94.5922
2	10.436	MM	0.3231	805.19446	41.53139	5.4078

Supplementary Figure 157. HPLC analysis of compound (-)-16.

Racemic sample 17: HPLC (Daicel Chiralpak IC column, hexane/iPrOH = 50:50, flow rate: 1.0 mL/min, λ= 254 nm)

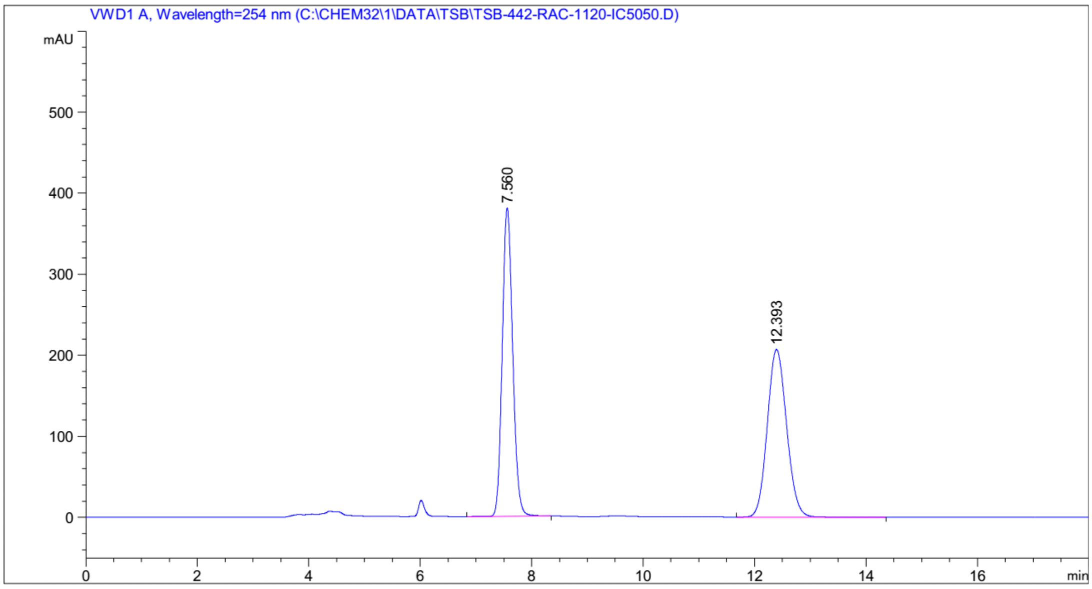

Peak Name	RT [min]	Type	width [min]	Area [mAU*s]	Height [mAu]	Area ratio %
1	7.560	VB R	0.2020	4962.91797	380.88559	49.9398
2	12.393	BB	0.3745	4974.88916	206.89395	50.0602

Enantioenriched sample (-)-17:

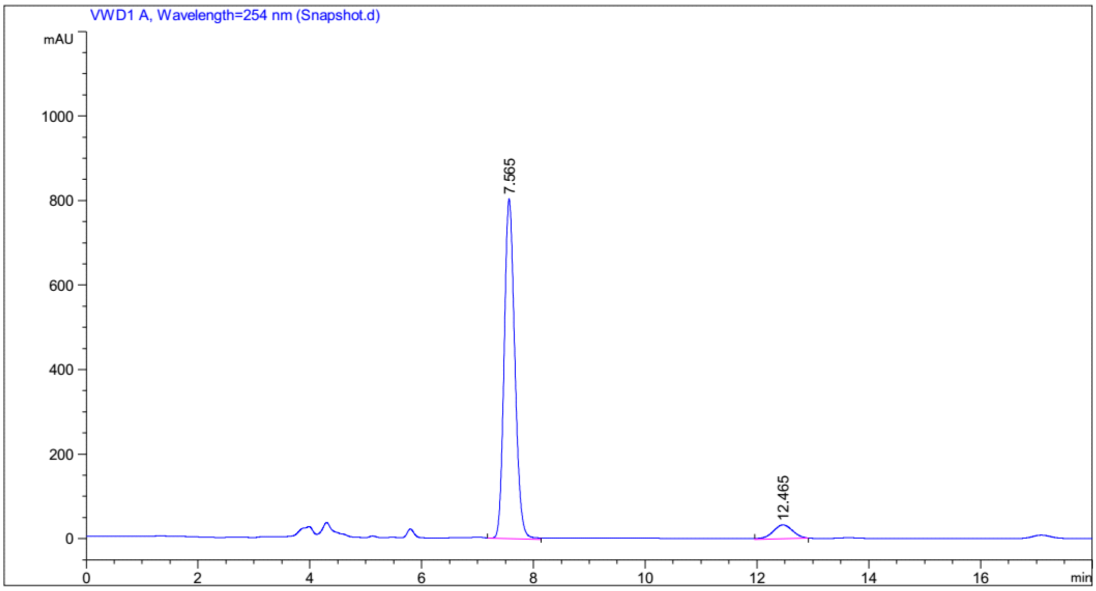

Peak Name	RT [min]	Type	width [min]	Area [mAU*s]	Height [mAu]	Area ratio %
1	7.565	MM	0.2201	1.06094e4	803.48981	93.1021
2	12.465	MM	0.4051	786.04742	32.34270	6.8979

Supplementary Figure 158. HPLC analysis of compound (-)-17.

Racemic sample 18: HPLC (Daicel Chiralpak IC column, hexane/iPrOH = 50:50, flow rate: 1.0 mL/min, λ= 254 nm)

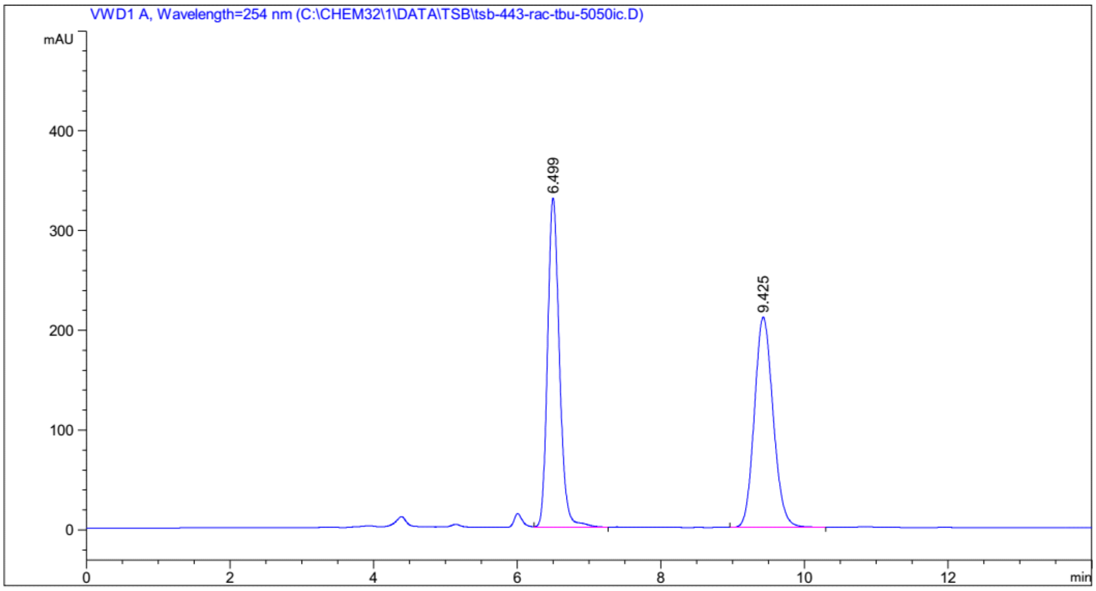

Peak Name	RT [min]	Type	width [min]	Area [mAU*s]	Height [mAu]	Area ratio %
1	6.499	VB	0.1754	3772.60645	329.90118	49.7237
2	9.425	BB	0.2813	3814.52808	210.62274	50.2763

Enantioenriched sample (-)-18:

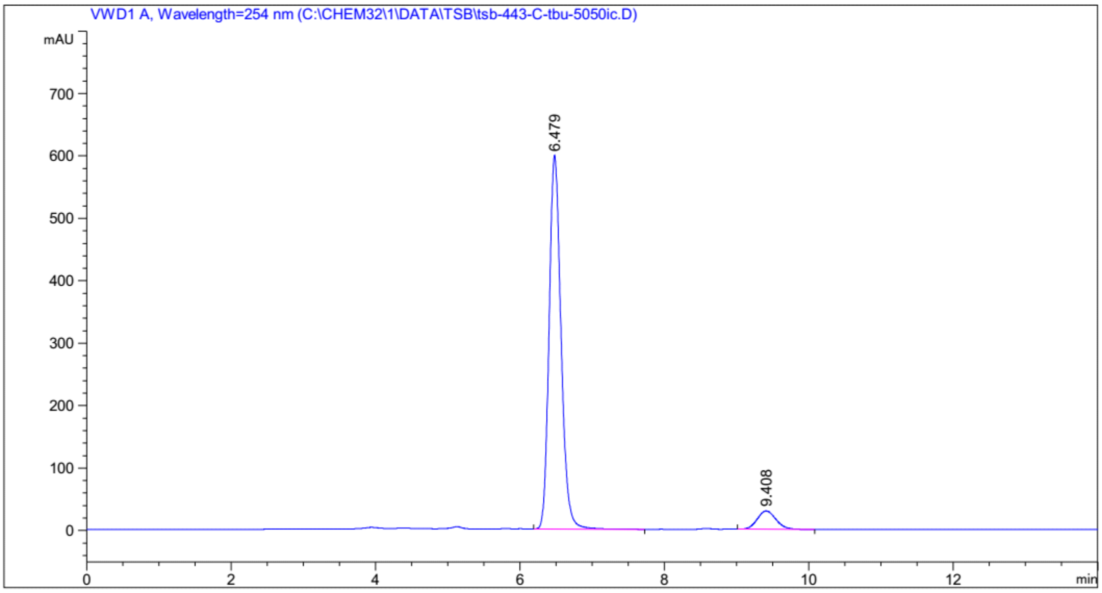

Peak Name	RT [min]	Type	width [min]	Area [mAU*s]	Height [mAu]	Area ratio %
1	6.479	BB	0.1763	6839.17871	598.53064	92.8250
2	9.408	BB	0.2797	528.63910	29.40702	7.1750

Supplementary Figure 159. HPLC analysis of compound (-)-18.

Racemic sample 19: HPLC (Daicel Chiralpak IC column, hexane/iPrOH = 50:50, flow rate: 1.0 mL/min, λ= 254 nm)

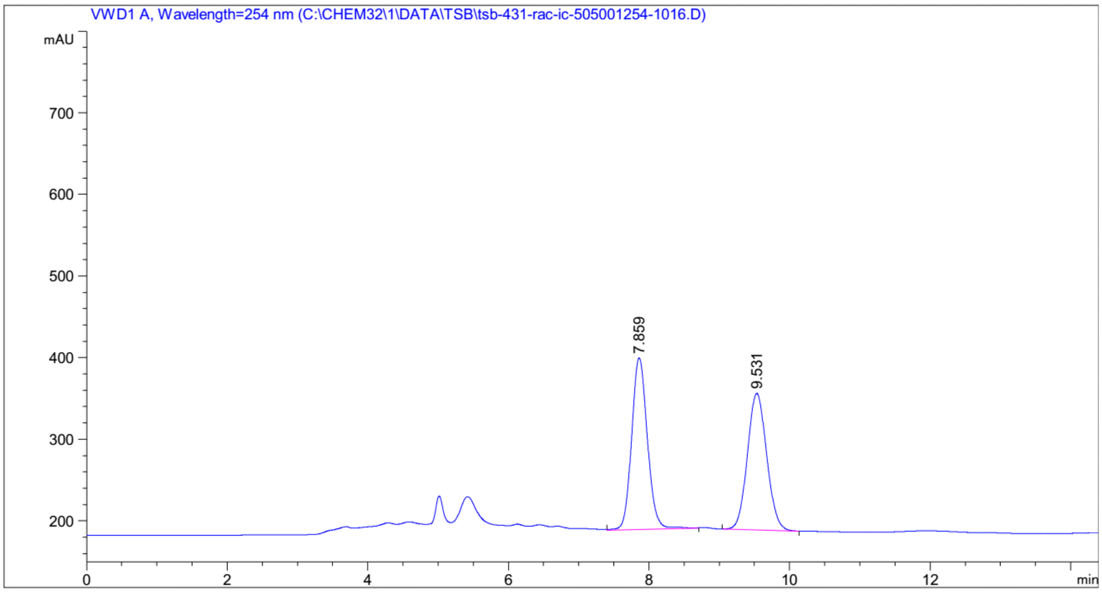

Peak Name	RT [min]	Type	width [min]	Area [mAU*s]	Height [mAu]	Area ratio %
1	7.859	MM	0.2603	3281.64575	210.14519	50.7126
2	9.531	BB	0.2974	3189.42578	167.30484	49.2874

Enantioenriched sample (-)-19:

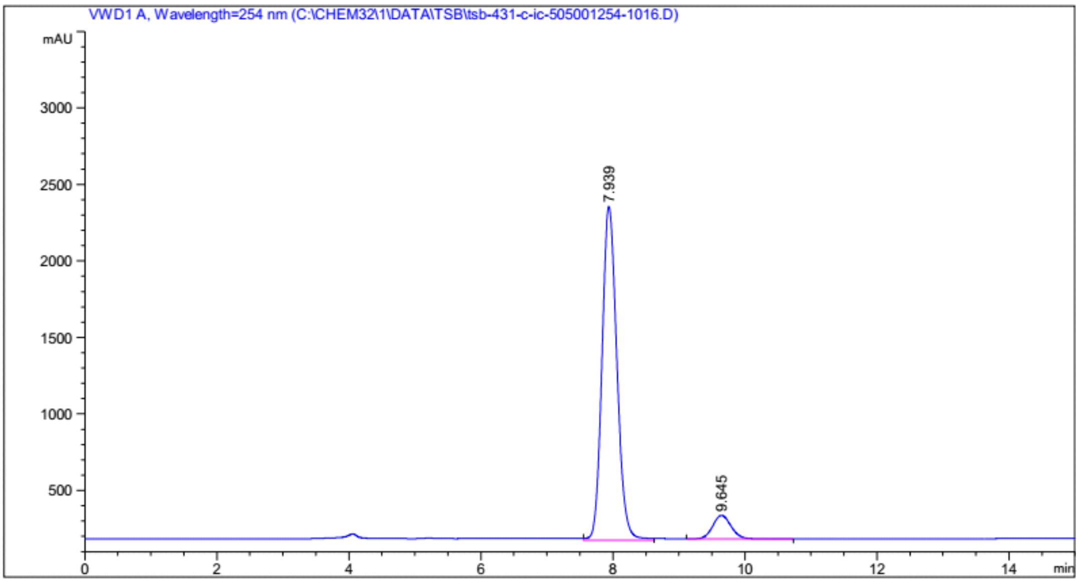

Peak Name	RT [min]	Type	width [min]	Area [mAU*s]	Height [mAu]	Area ratio %
1	7.939	MM	0.2611	3.41624e4	2181.00269	92.0428
2	9.645	BB	0.2996	2953.38306	153.40781	7.9572

Supplementary Figure 160. HPLC analysis of compound (-)-19.

Racemic sample 20: HPLC (Daicel Chiralpak IC column, hexane/iPrOH = 70:30, flow rate: 1.0 mL/min, λ= 254 nm)

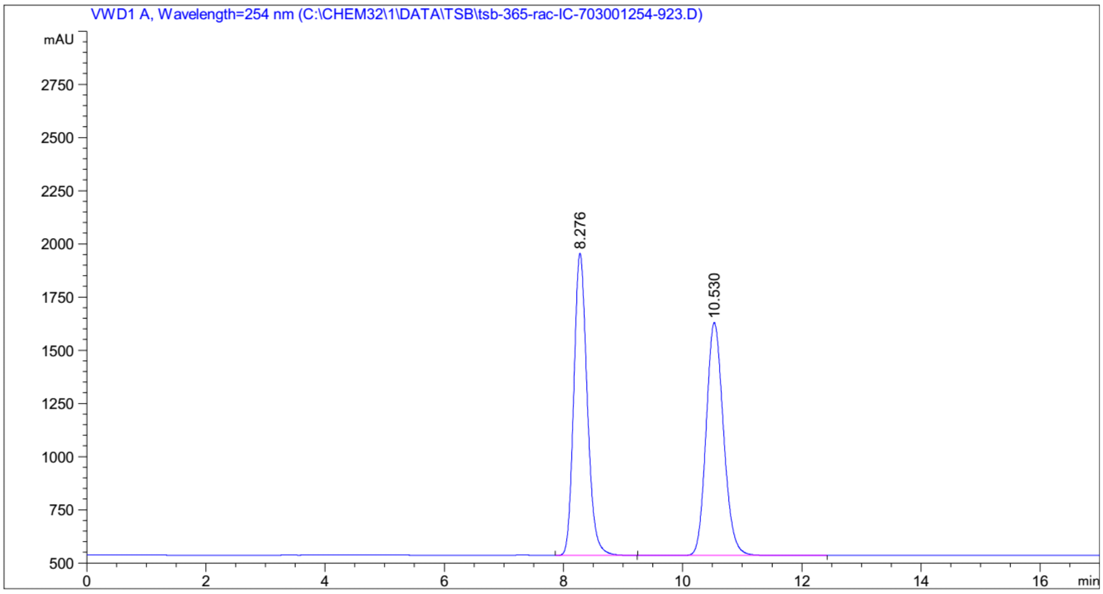

Peak Name	RT [min]	Type	width [min]	Area [mAU*s]	Height [mAu]	Area ratio %
1	8.276	BB	0.2404	2.20935e4	1421.02185	49.9731
2	10.530	VB R	0.3127	2.21173e4	1094.60583	50.0269

Enantioenriched sample (-)-20:

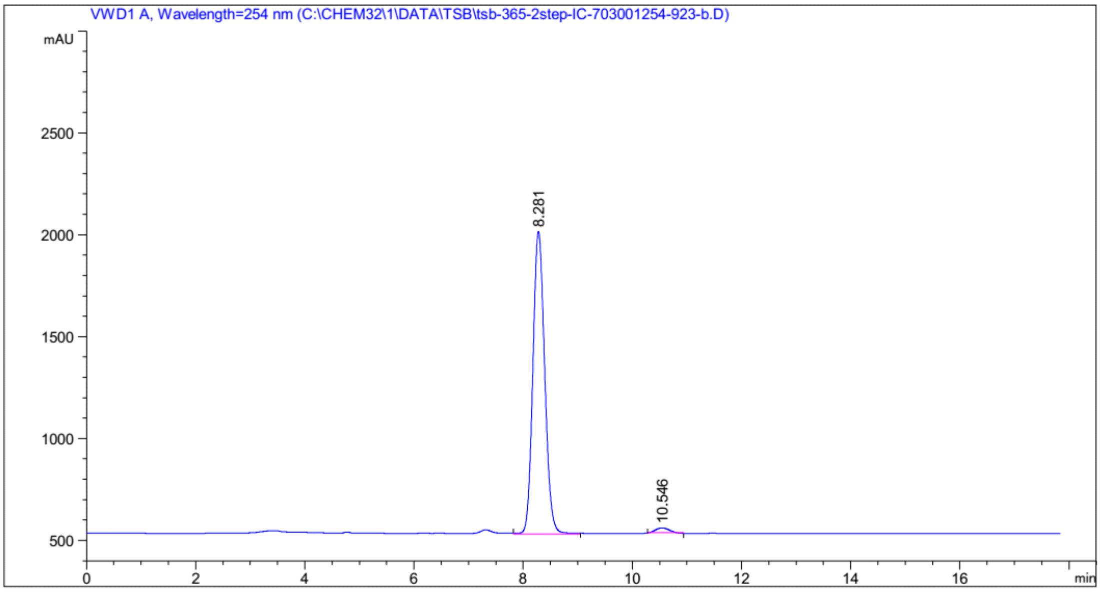

Peak Name	RT [min]	Type	width [min]	Area [mAU*s]	Height [mAu]	Area ratio %
1	8.281	MM	0.2505	2.23232e4	1485.40991	98.4438
2	10.546	MM	0.2614	352.88663	22.49650	1.5562

Supplementary Figure 161. HPLC analysis of compound (-)-20.

Racemic sample 21: HPLC (Daicel Chiralpak IC column, hexane/iPrOH = 60:40, flow rate: 1.0 mL/min, λ= 254 nm)

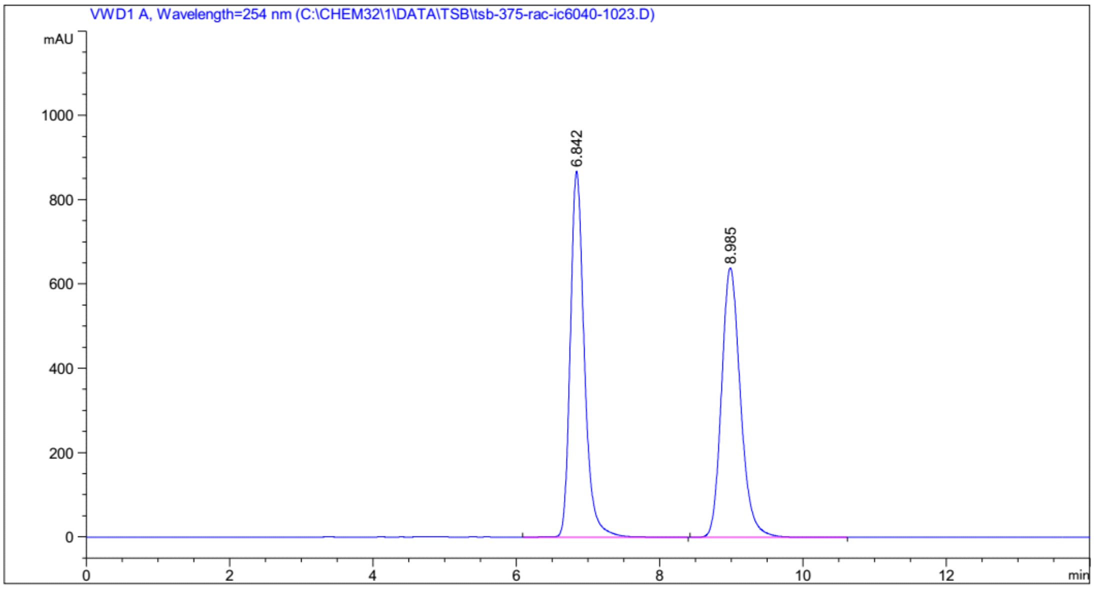

Peak Name	RT [min]	Type	width [min]	Area [mAU*s]	Height [mAu]	Area ratio %
1	6.842	VB R	0.2074	1.17911e4	868.32715	50.1065
2	8.985	BB	0.2824	1.17410e4	639.01331	49.8935

Enantioenriched sample (-)-21:

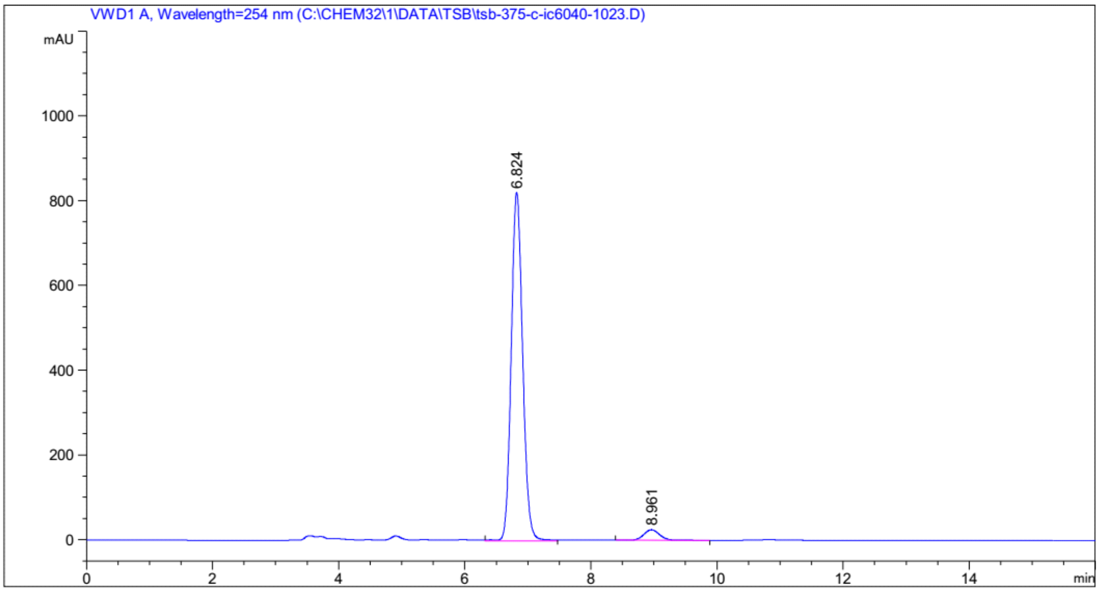

Peak Name	RT [min]	Type	width [min]	Area [mAU*s]	Height [mAu]	Area ratio %
1	6.824	MM	0.2106	1.04007e4	823.10474	96.0245
2	8.961	BB	0.2695	430.60245	24.81174	3.9755

Supplementary Figure 162. HPLC analysis of compound (-)-21.

Racemic sample 22: HPLC (Daicel Chiralpak IC column, hexane/iPrOH = 70:30, flow rate: 1.0 mL/min, λ= 254 nm)

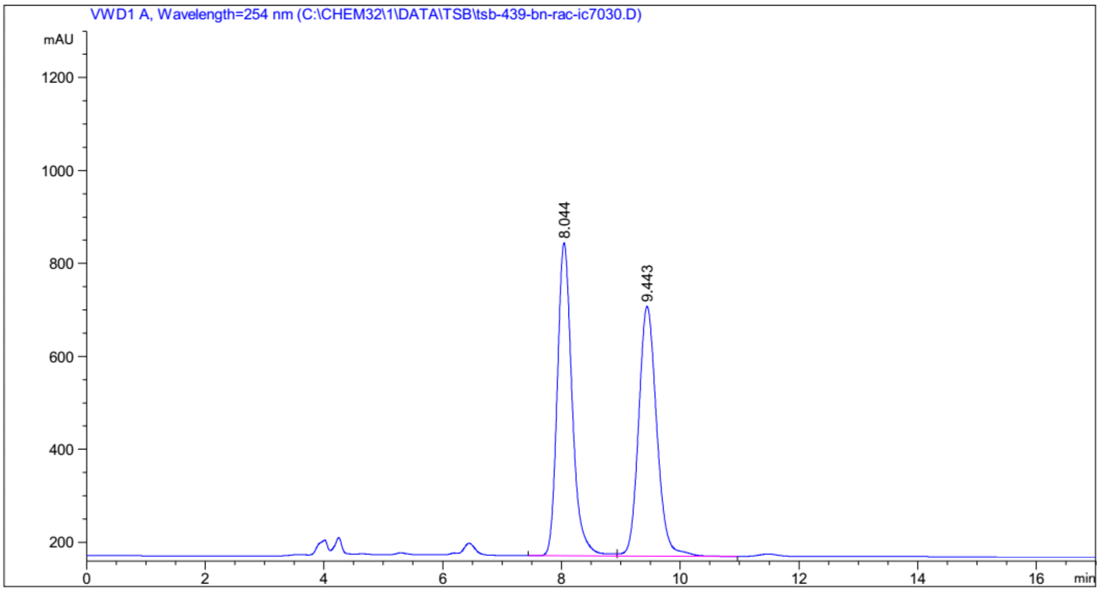

Peak Name	RT [min]	Type	width [min]	Area [mAU*s]	Height [mAu]	Area ratio %
1	8.044	BV	0.2651	1.16722e4	673.79559	50.6719
2	9.443	VB	0.3257	1.13627e4	537.37115	49.3281

Enantioenriched sample (-)-22:

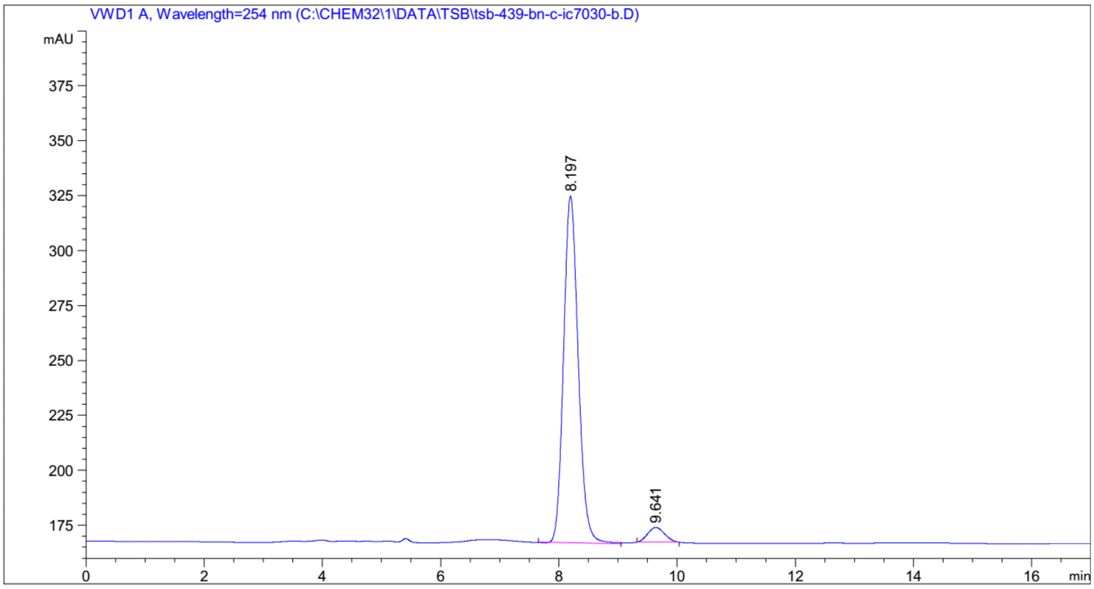

Peak Name	RT [min]	Type	width [min]	Area [mAU*s]	Height [mAu]	Area ratio %
1	8.197	MM	0.2863	2710.09692	157.74257	95.3925
2	9.641	MM	0.3292	130.89844	6.62695	4.6075

Supplementary Figure 163. HPLC analysis of compound (-)-22.

Racemic sample 23: HPLC (Daicel Chiralpak IC column, hexane/iPrOH = 50:50, flow rate: 1.0 mL/min, λ= 254 nm)

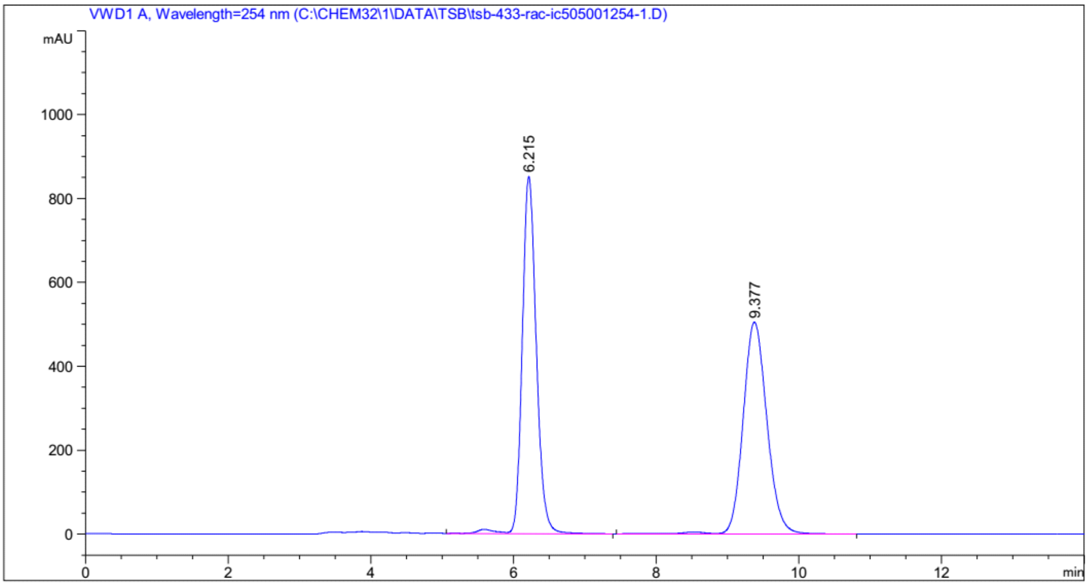

Peak Name	RT [min]	Type	width [min]	Area [mAU*s]	Height [mAu]	Area ratio %
1	6.215	VB R	0.2092	1.17233e4	851.51849	49.9793
2	9.377	VB R	0.3578	1.17330e4	505.09076	50.0207

Enantioenriched sample (-)-23:

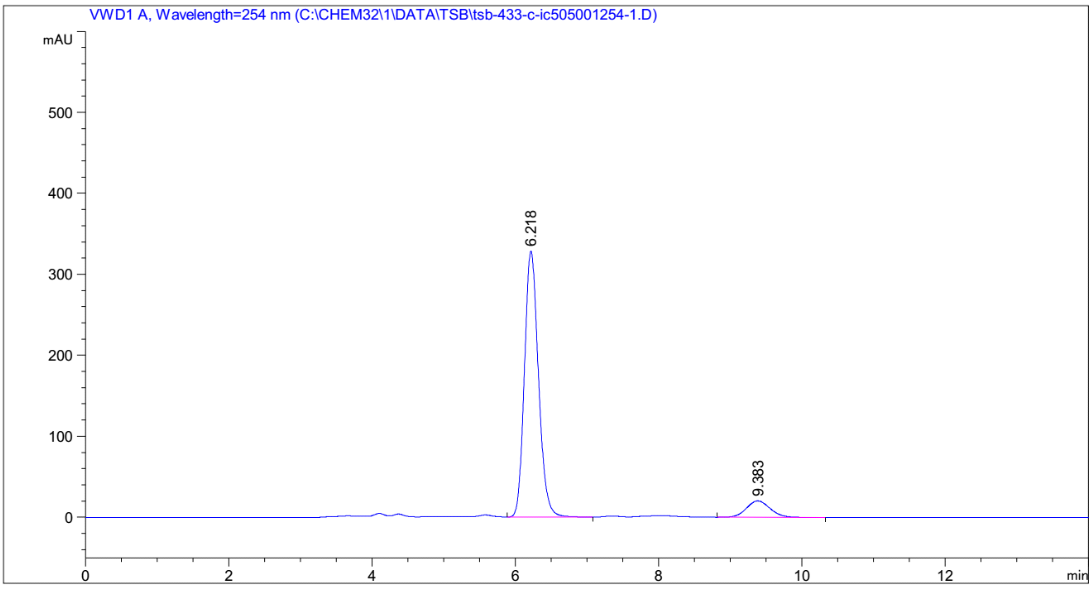

Peak Name	RT [min]	Type	width [min]	Area [mAU*s]	Height [mAu]	Area ratio %
1	6.218	BB	0.2094	4460.45410	328.59430	90.5182
2	9.383	BB	0.3575	467.23550	20.30014	9.4818

Supplementary Figure 164. HPLC analysis of compound (-)-23.

Racemic sample 24: HPLC (Daicel Chiralpak IC column, hexane/iPrOH = 70:30, flow rate: 1.0 mL/min, λ= 254 nm)

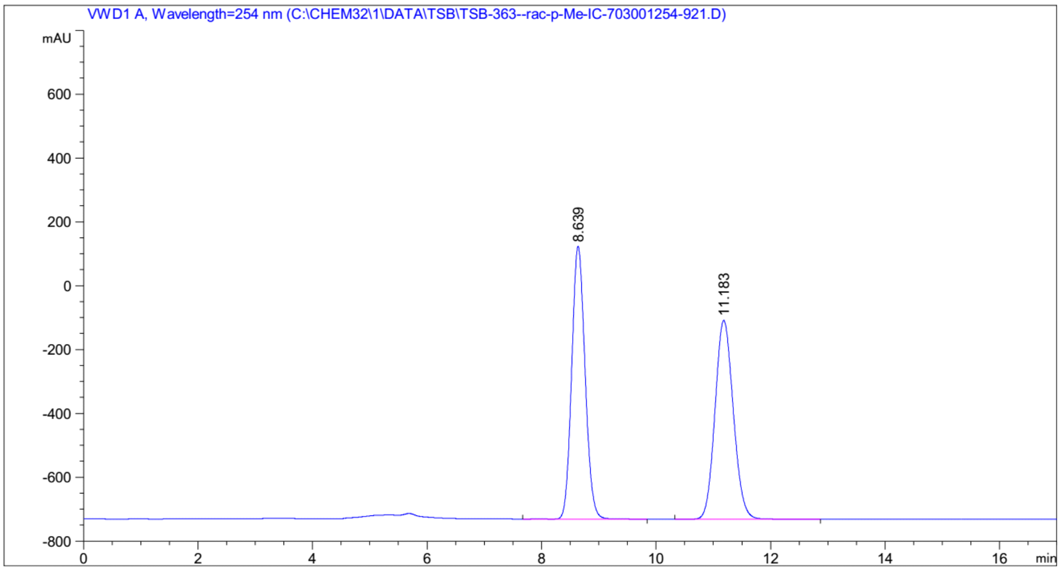

Peak Name	RT [min]	Type	width [min]	Area [mAU*s]	Height [mAu]	Area ratio %
1	8.639	VB R	0.2525	1.38742e4	854.26202	50.1183
2	11.183	BB	0.3456	1.38087e4	622.94043	49.8817

Enantioenriched sample (-)-24:

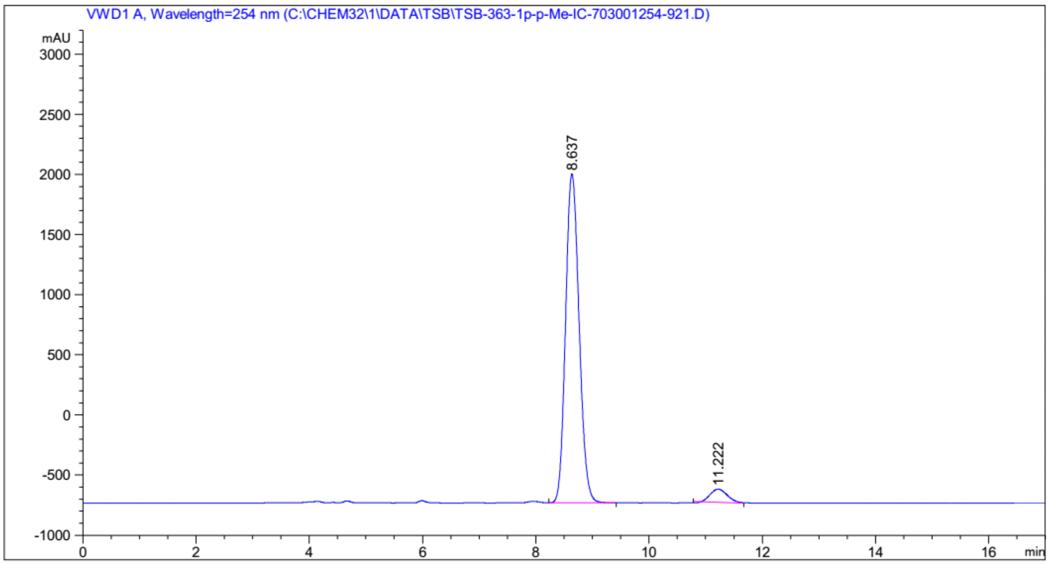

Peak Name	RT [min]	Type	width [min]	Area [mAU*s]	Height [mAu]	Area ratio %
1	8.637	VB	0.2585	4.53873e4	2736.03833	95.1950
2	11.222	MM	0.3507	2290.92358	108.87604	4.8050

Supplementary Figure 165. HPLC analysis of compound (-)-24.

Racemic sample 25: HPLC (Daicel Chiralpak IC column, hexane/iPrOH = 70:30, flow rate: 1.0 mL/min, λ= 254 nm)

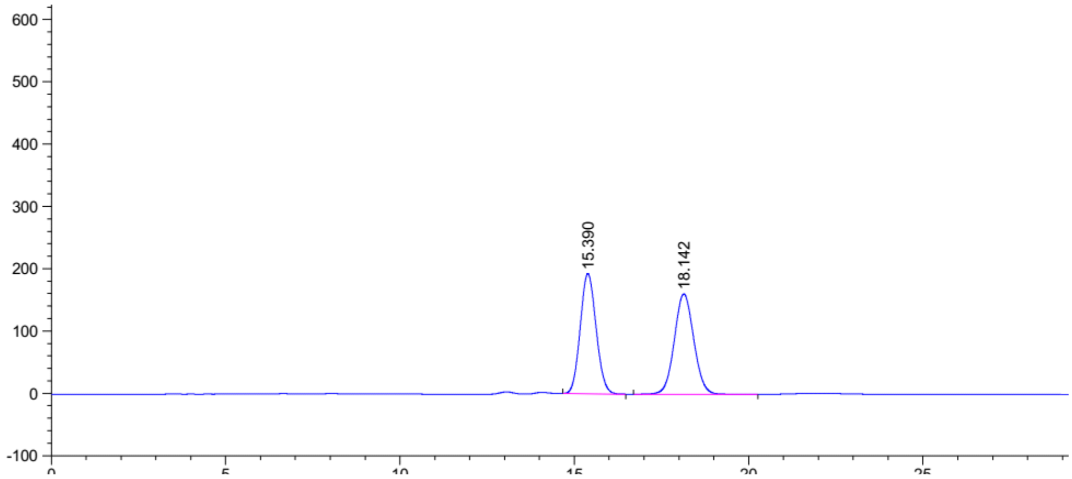

Peak Name	RT [min]	Type	width [min]	Area [mAU*s]	Height [mAu]	Area ratio %
1	15.390	BB	0.4928	6094.14990	192.76077	49.3718
2	18.142	BB	0.6034	6249.22949	161.16110	50.6282

Enantioenriched sample (-)-25:

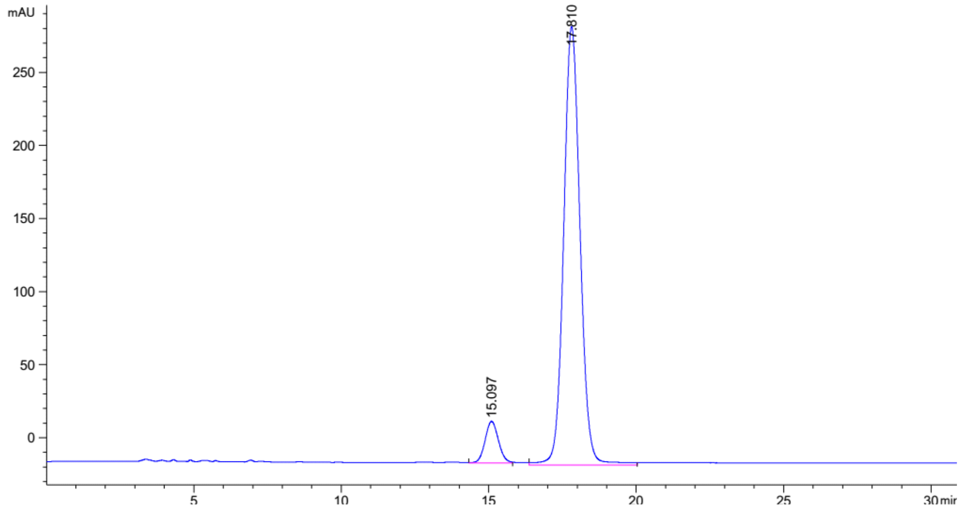

Peak Name	RT [min]	Type	width [min]	Area [mAU*s]	Height [mAu]	Area ratio %
1	15.097	MM	0.5256	897.29395	28.45368	7.1649
2	17.810	MM	0.6461	1.16262e4	299.88940	92.8351

Supplementary Figure 166. HPLC analysis of compound (-)-25.

Racemic sample 26: HPLC Daicel Chiralpak IC column, hexane/iPrOH = 70:30, flow rate: 1.0 mL/min, λ= 254 nm)

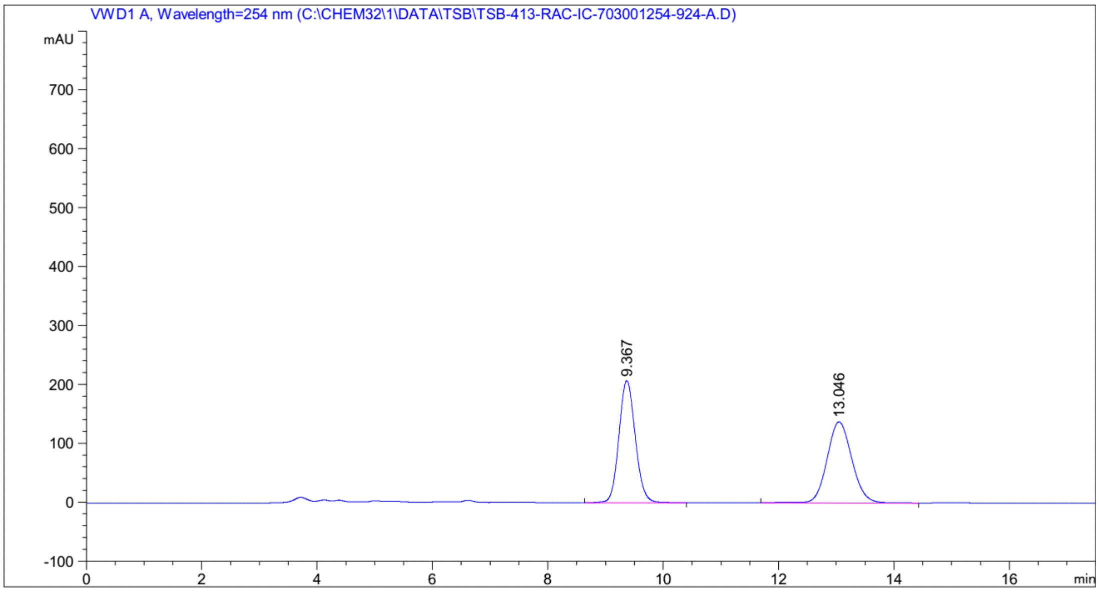

Peak Name	RT [min]	Type	width [min]	Area [mAU*s]	Height [mAu]	Area ratio %
1	9.367	BB	0.3058	4083.48682	207.30243	50.0216
2	13.046	BB	0.4614	4079.96777	137.39517	49.9784

Enantioenriched sample (-)-26:

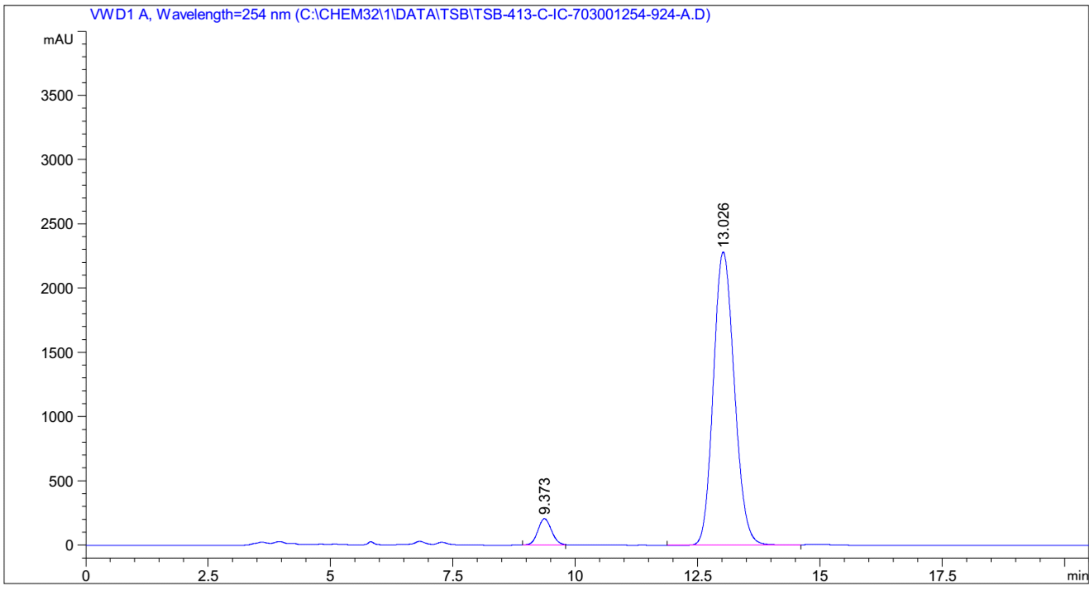

Peak Name	RT [min]	Type	width [min]	Area [mAU*s]	Height [mAu]	Area ratio %
1	9.373	MM	0.3208	3923.22754	203.84216	5.4687
2	13.026	MM	0.4959	6.78171e4	2279.42676	94.5313

Supplementary Figure 167. HPLC analysis of compound (-)-26.

Racemic sample 28: HPLC (Daicel Chiralpak OD column, hexane/iPrOH = 90:10, flow rate: 1.0 mL/min, λ= 254 nm)

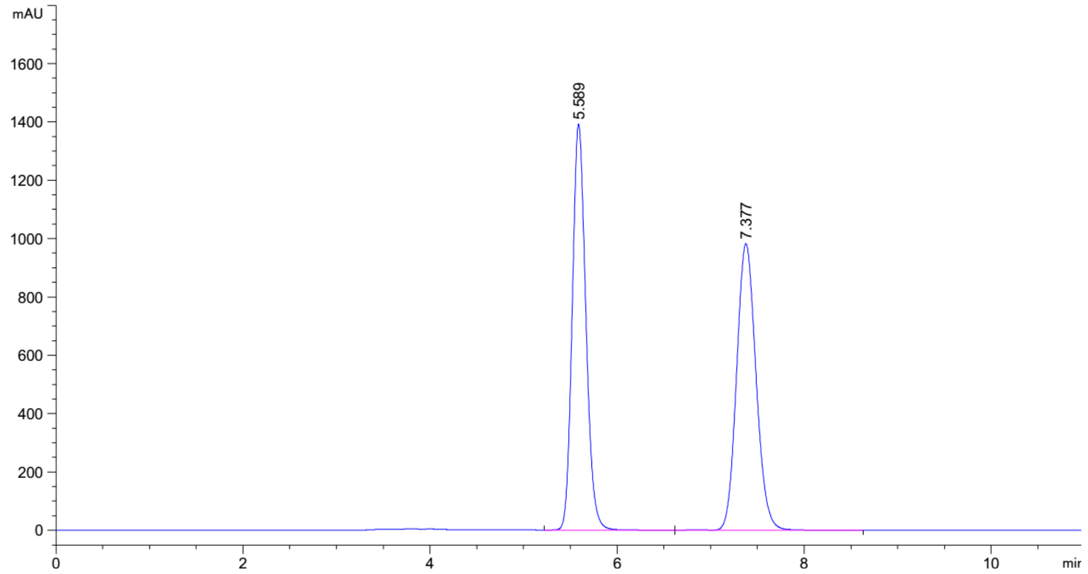

Peak Name	RT [min]	Type	width [min]	Area [mAU*s]	Height [mAu]	Area ratio %
1	5.589	BV R	0.1556	1.40411e4	1392.42078	49.7386
2	7.377	VB R	0.2231	1.41887e4	983.11682	50.2614

Enantioenriched sample (-)-28:

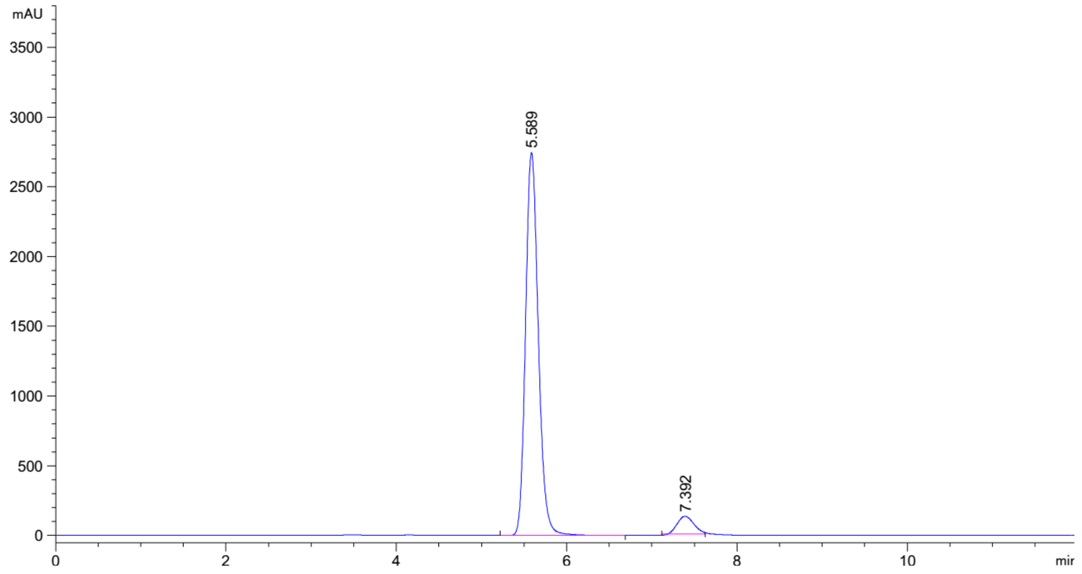

Peak Name	RT [min]	Type	width [min]	Area [mAU*s]	Height [mAu]	Area ratio %
1	5.589	BB	0.1612	2.85449e4	2746.63403	94.2554
2	7.392	MM	0.2272	1739.73718	127.61334	5.7446

Supplementary Figure 168. HPLC analysis of compound (-)-28.

Racemic sample 29: HPLC (Daicel Chiralpak IC column, hexane/iPrOH = 70:30, flow rate: 1.0 mL/min, λ= 254 nm)

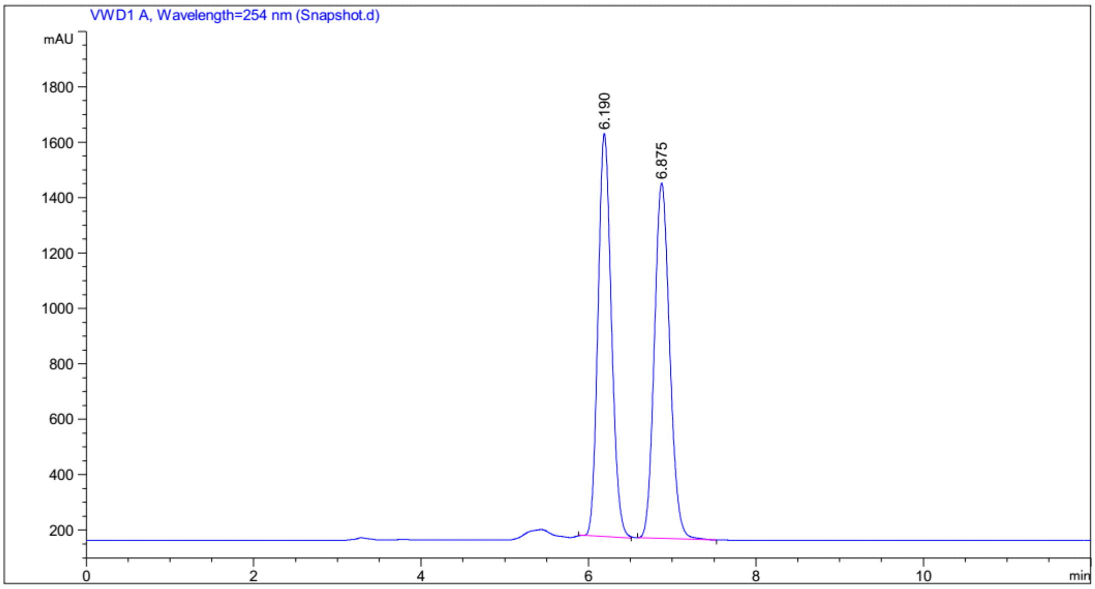

Peak Name	RT [min]	Type	width [min]	Area [mAU*s]	Height [mAu]	Area ratio %
1	6.190	MM	0.1852	1.61624e4	1454.68628	49.6740
2	6.875	BB	0.1980	1.63746e4	1282.29211	50.3260

Enantioenriched sample (-)-29:

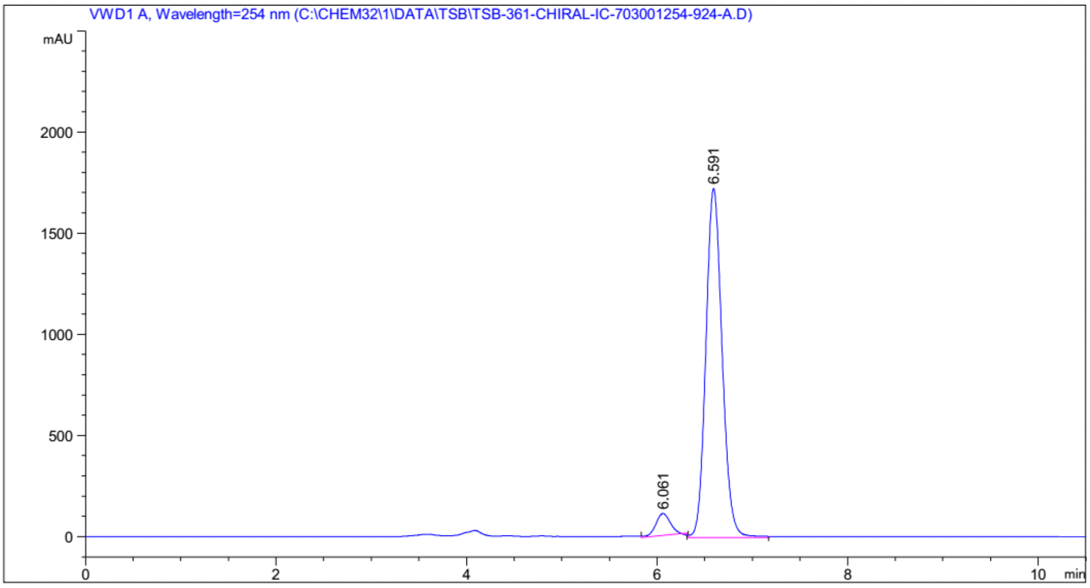

Peak Name	RT [min]	Type	width [min]	Area [mAU*s]	Height [mAu]	Area ratio %
1	6.061	MM	0.1741	1125.77002	107.77047	5.2280
2	6.591	MM	0.1973	2.04076e4	1724.27161	94.7720

Supplementary Figure 169. HPLC analysis of compound (-)-29.

Racemic sample 30: HPLC (Daicel Chiralpak IC column, hexane/iPrOH = 60:40, flow rate: 1.0 mL/min, λ= 254 nm)

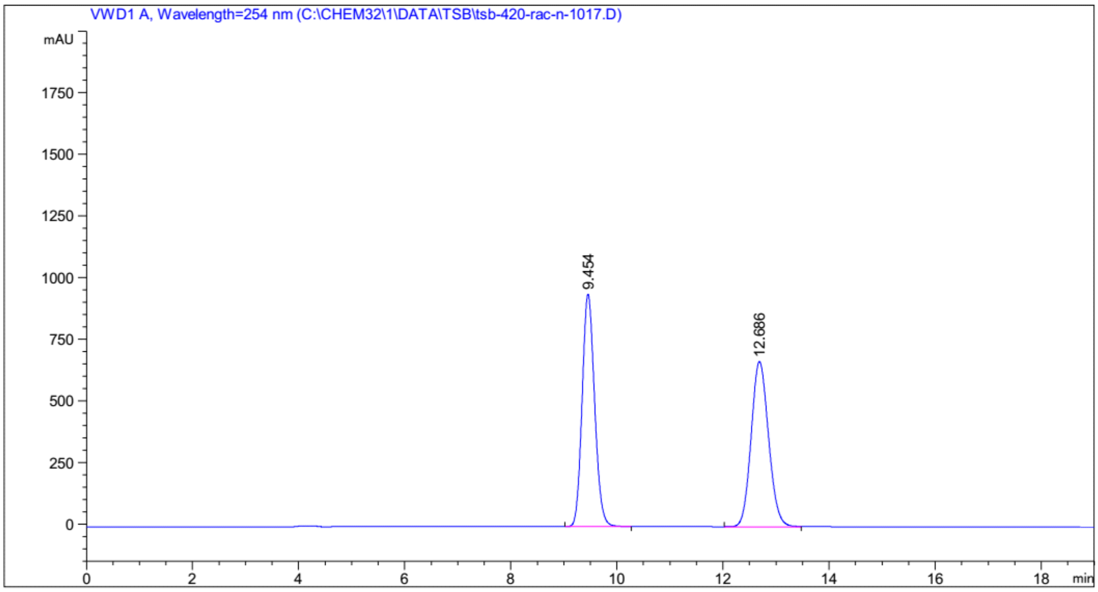

Peak Name	RT [min]	Type	width [min]	Area [mAU*s]	Height [mAu]	Area ratio %
1	9.454	BB	0.2523	1.53796e4	942.99603	49.8855
2	12.686	MM	0.3831	1.54502e4	672.08905	50.1145

Enantioenriched sample (-)-30:

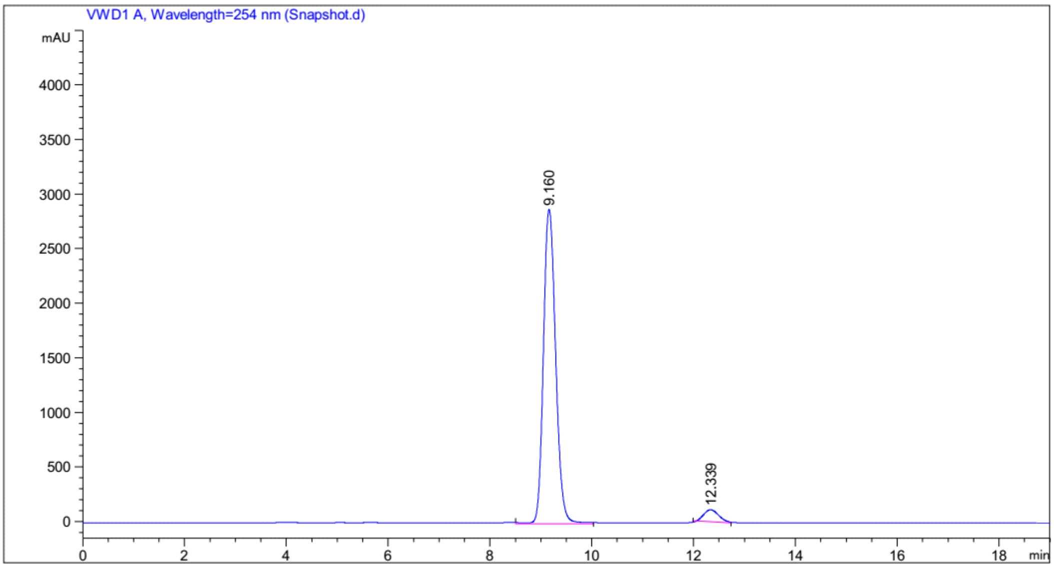

Peak Name	RT [min]	Type	width [min]	Area [mAU*s]	Height [mAu]	Area ratio %
1	9.160	MM	0.2796	4.83084e4	2880.12134	95.5780
2	12.339	MM	0.3413	2235.00928	109.14394	4.4220

Supplementary Figure 170. HPLC analysis of compound (-)-30.

Racemic sample 31: HPLC (Daicel Chiralpak IC column, hexane/iPrOH = 60:40, flow rate: 1.0 mL/min, λ= 254 nm)

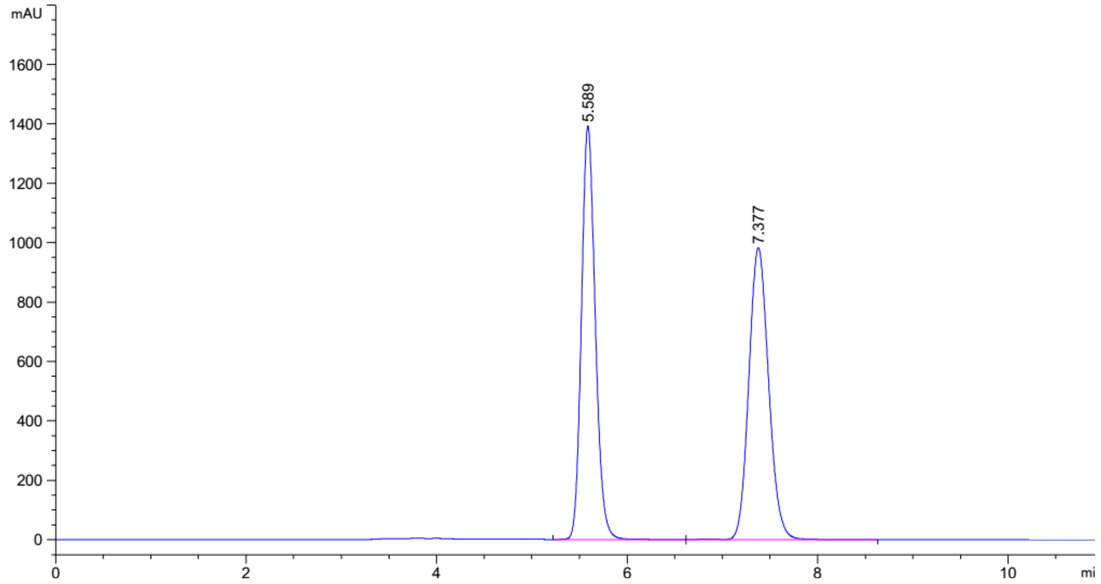

Peak Name	RT [min]	Type	width [min]	Area [mAU*s]	Height [mAu]	Area ratio %
1	5.589	BV R	0.1556	1.40411e4	1392.42078	49.7386
2	7.377	VB R	0.2231	1.41887e4	983.11682	50.2614

Enantioenriched sample (-)-31:

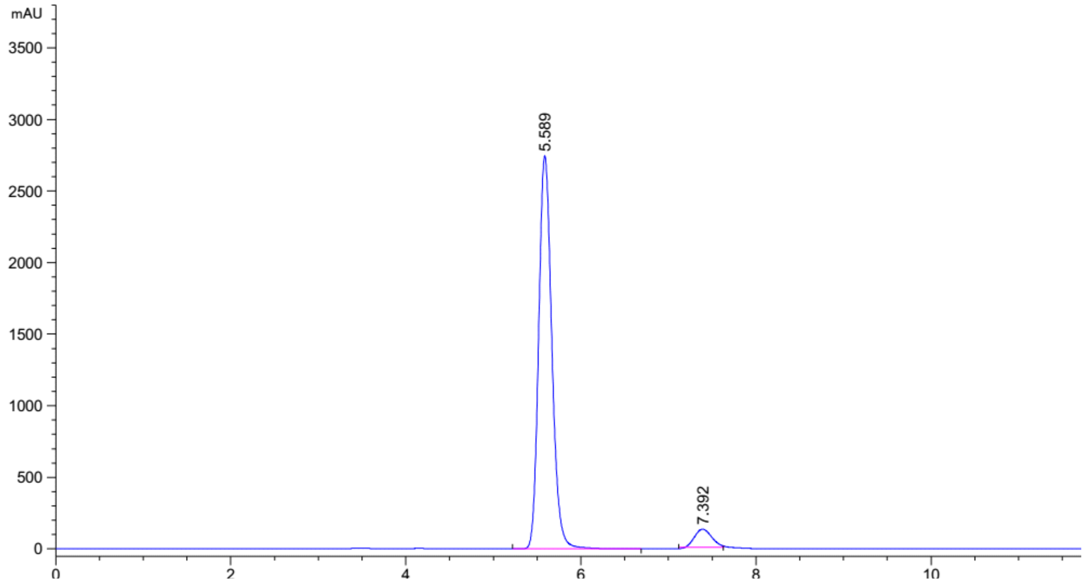

Peak Name	RT [min]	Type	width [min]	Area [mAU*s]	Height [mAu]	Area ratio %
1	5.589	BB	0.1612	2.85449e4	2746.63403	94.2554
2	7.392	MM	0.2272	1739.73718	127.61334	5.7446

Supplementary Figure 171. HPLC analysis of compound (-)-31.

Racemic sample 32: HPLC (Daicel Chiralpak IC column, hexane/iPrOH = 60:40, flow rate: 1.0 mL/min, λ= 254 nm)

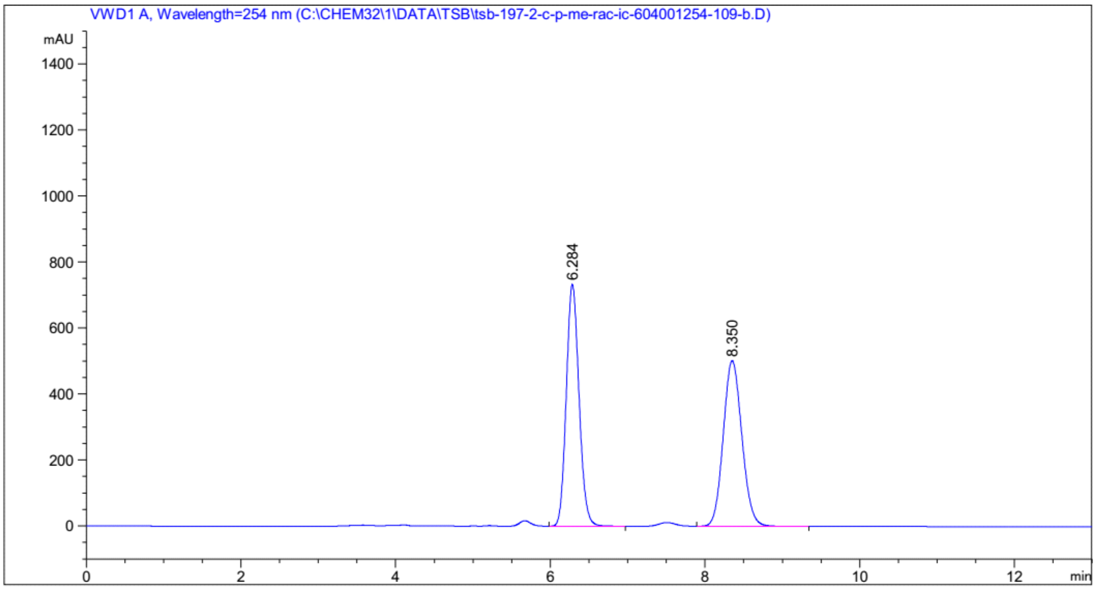

Peak Name	RT [min]	Type	width [min]	Area [mAU*s]	Height [mAu]	Area ratio %
1	6.284	BB	0.1777	8400.53418	732.88818	49.9885
2	8.350	BB	0.2604	8404.40820	501.88208	50.0115

Enantioenriched sample (-)-32:

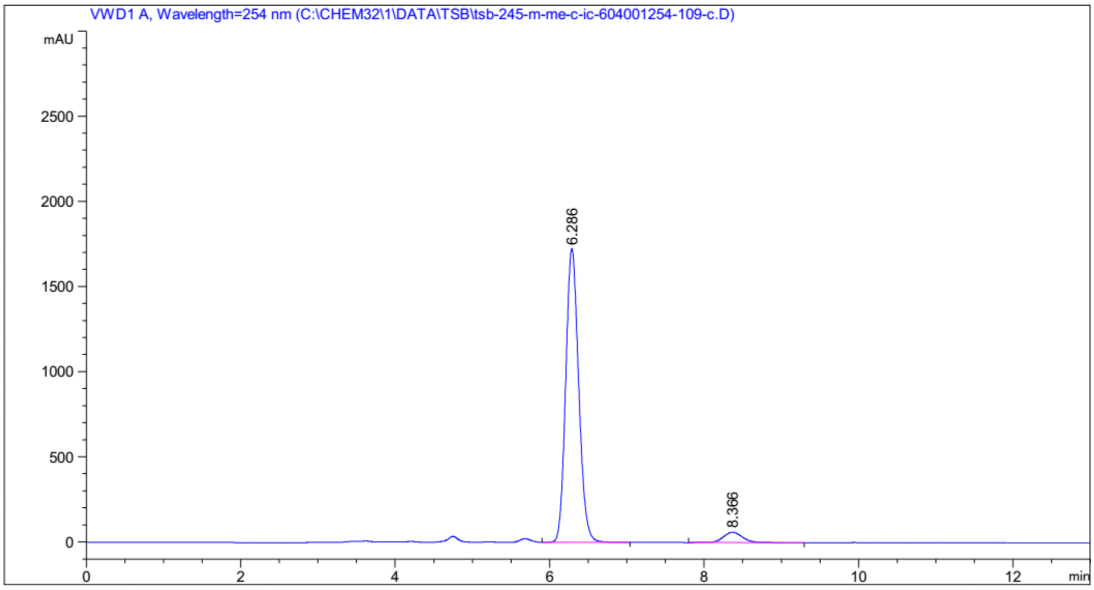

Peak Name	RT [min]	Type	width [min]	Area [mAU*s]	Height [mAu]	Area ratio %
1	6.286	VB	0.1810	2.00950e4	1723.40259	95.1112
2	8.366	BB	0.2643	1032.90869	60.44993	4.8888

Supplementary Figure 172. HPLC analysis of compound (-)-32.

Racemic sample 33: HPLC (Daicel Chiralpak OD-H column, hexane/iPrOH = 95:05, flow rate: 1.0 mL/min, λ= 254 nm)

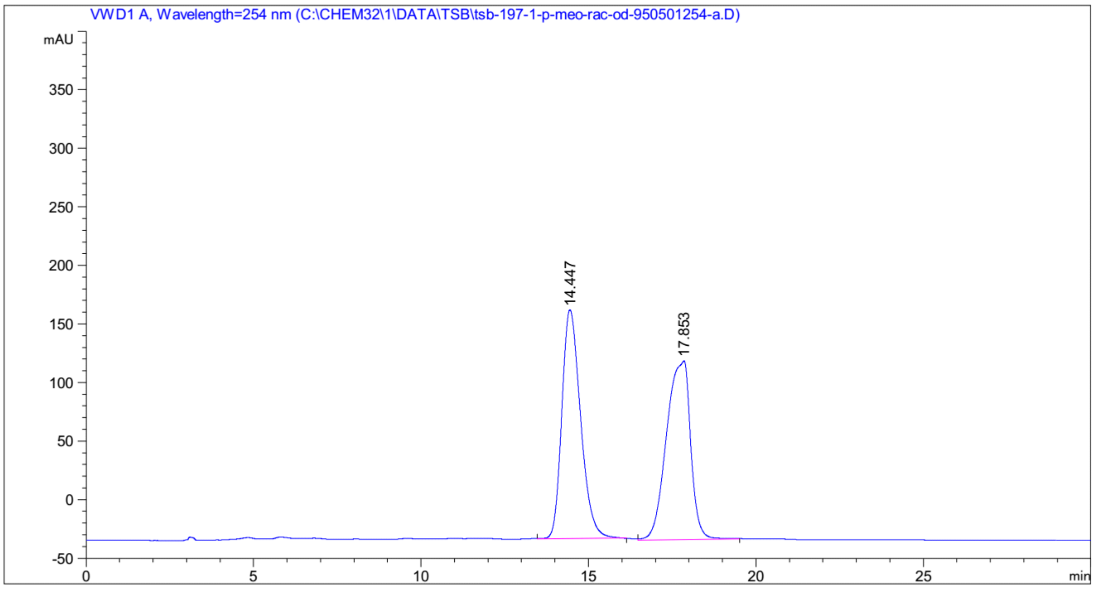

Peak Name	RT [min]	Type	width [min]	Area [mAU*s]	Height [mAu]	Area ratio %
1	14.447	MM	0.6482	7600.23926	195.41176	49.8889
2	17.853	MM	0.8334	7634.10352	152.67778	50.1111

Enantioenriched sample (-)-33:

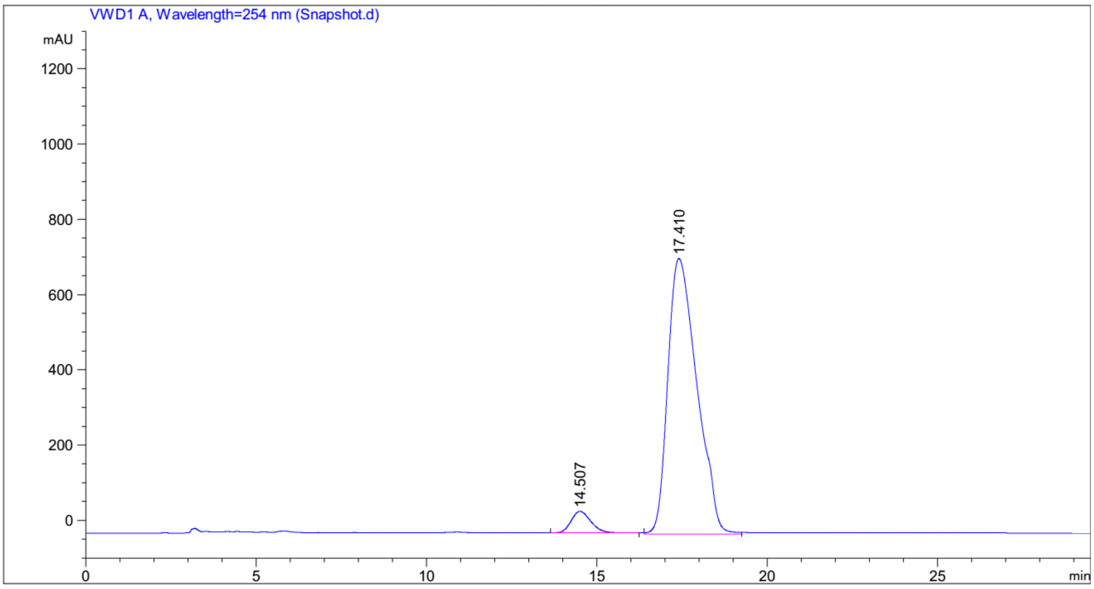

Peak Name	RT [min]	Type	width [min]	Area [mAU*s]	Height [mAu]	Area ratio %
1	14.507	BB	0.6181	2278.02466	57.01461	4.9768
2	17.410	MM	0.9893	4.34948e4	732.75134	95.0232

Supplementary Figure 173. HPLC analysis of compound (-)-33.

Racemic sample 34: HPLC (Daicel Chiralpak IC column, hexane/iPrOH = 70:30, flow rate: 1.0 mL/min, λ= 254 nm)

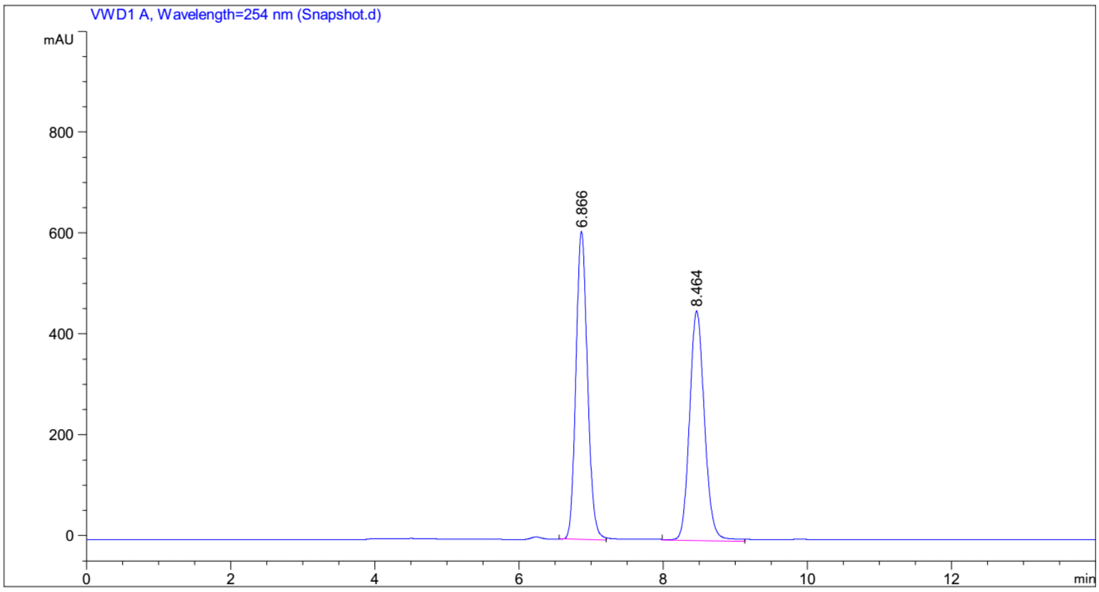

Peak Name	RT [min]	Type	width [min]	Area [mAU*s]	Height [mAu]	Area ratio %
1	6.866	MM	0.1850	6769.43066	609.88129	50.6422
2	8.464	MM	0.2415	6597.74121	455.33661	49.3578

Enantioenriched sample(-)-34:

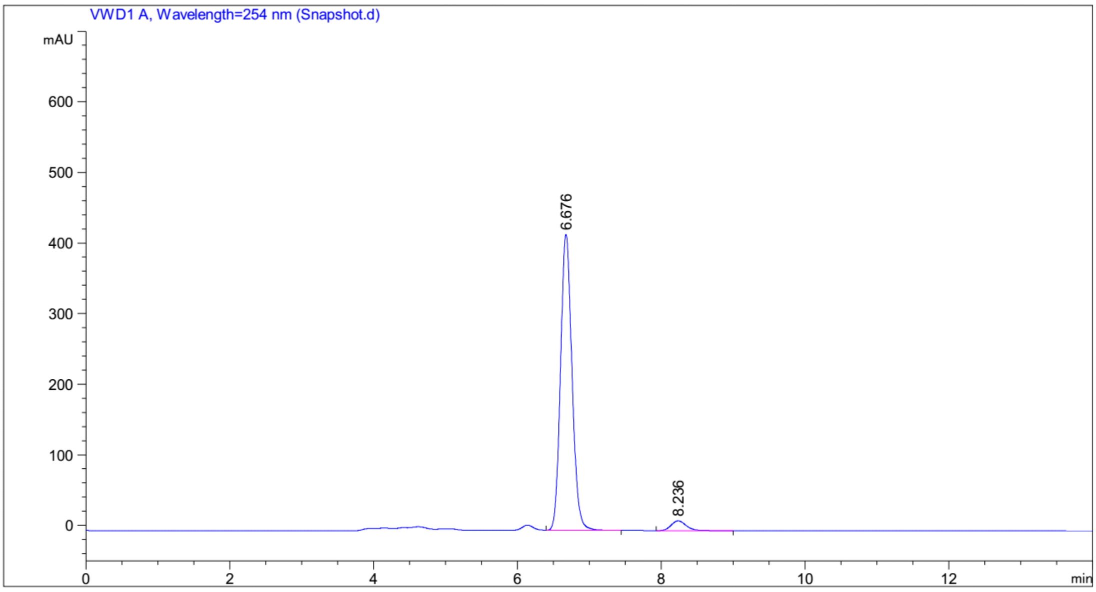

Peak Name	RT [min]	Type	width [min]	Area [mAU*s]	Height [mAu]	Area ratio %
1	6.676	VB	0.1713	4646.72119	419.35507	95.7891
2	8.236	BB	0.2260	204.27197	13.93881	4.2109

Supplementary Figure 174. HPLC analysis of compound (-)-34.

Racemic sample 35: HPLC (Daicel Chiralpak IC column hexane/iPrOH = 70:30, flow rate: 1.0 mL/min, λ= 254 nm)

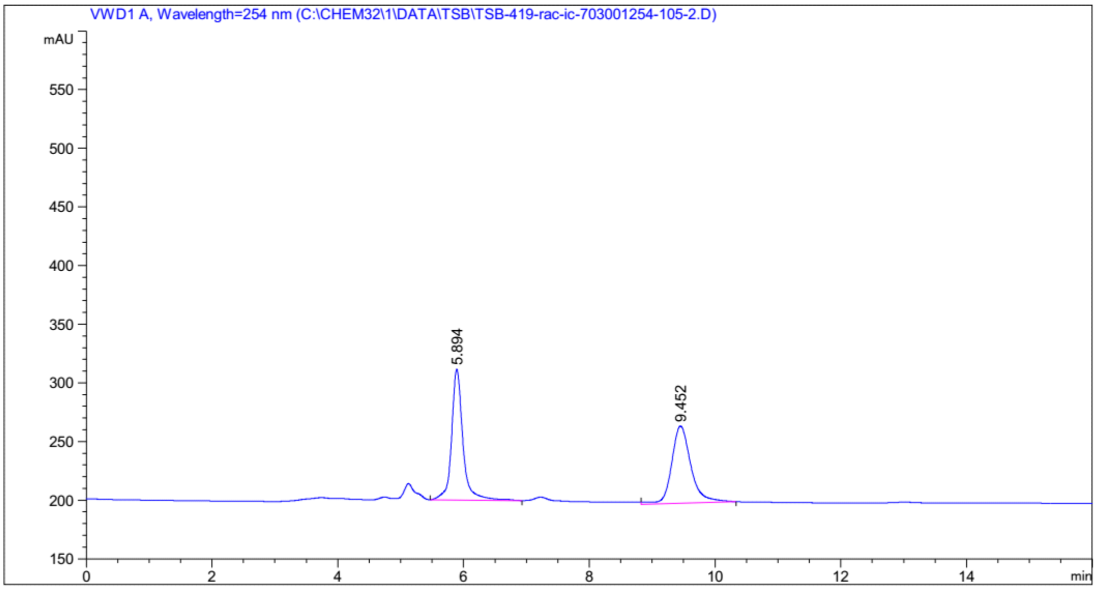

Peak Name	RT [min]	Type	width [min]	Area [mAU*s]	Height [mAu]	Area ratio %
1	5.894	BB	0.1862	1396.45740	111.47293	49.7964
2	9.452	MM	0.3557	1407.87683	65.96391	50.2036

Enantioenriched sample (-)-35:

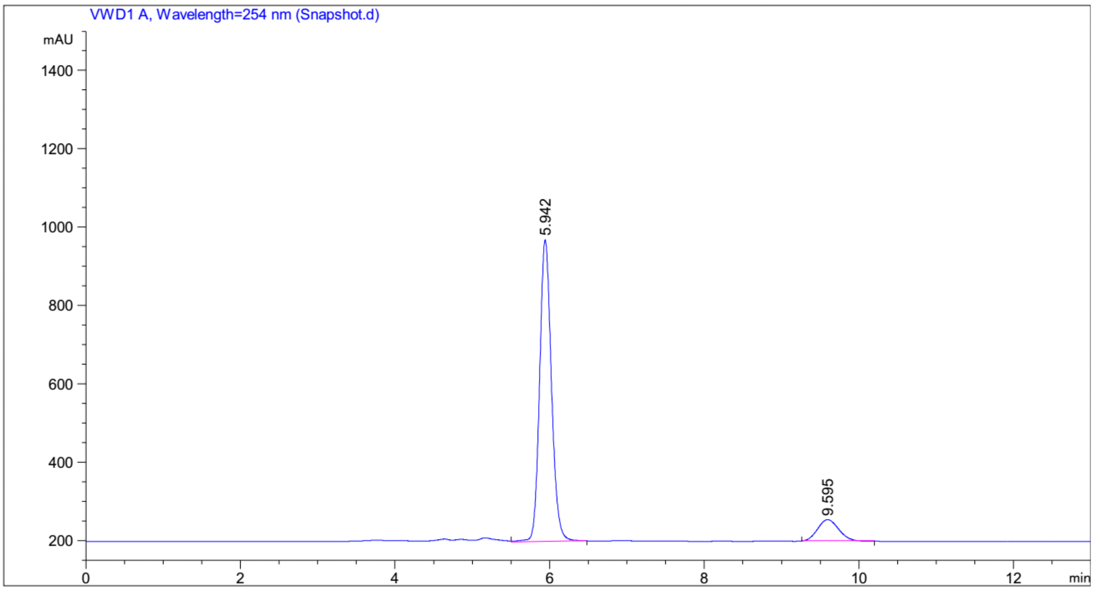

Peak Name	RT [min]	Type	width [min]	Area [mAU*s]	Height [mAu]	Area ratio %
1	5.942	MM	0.1766	8162.04248	770.34375	89.3239
2	9.595	MM	0.3052	975.53510	53.27424	10.6761

Supplementary Figure 175. HPLC analysis of compound (-)-35.

Racemic sample 36: HPLC (Daicel Chiralpak IC column, hexane/iPrOH = 70:30, flow rate: 1.0 mL/min, λ= 254 nm)

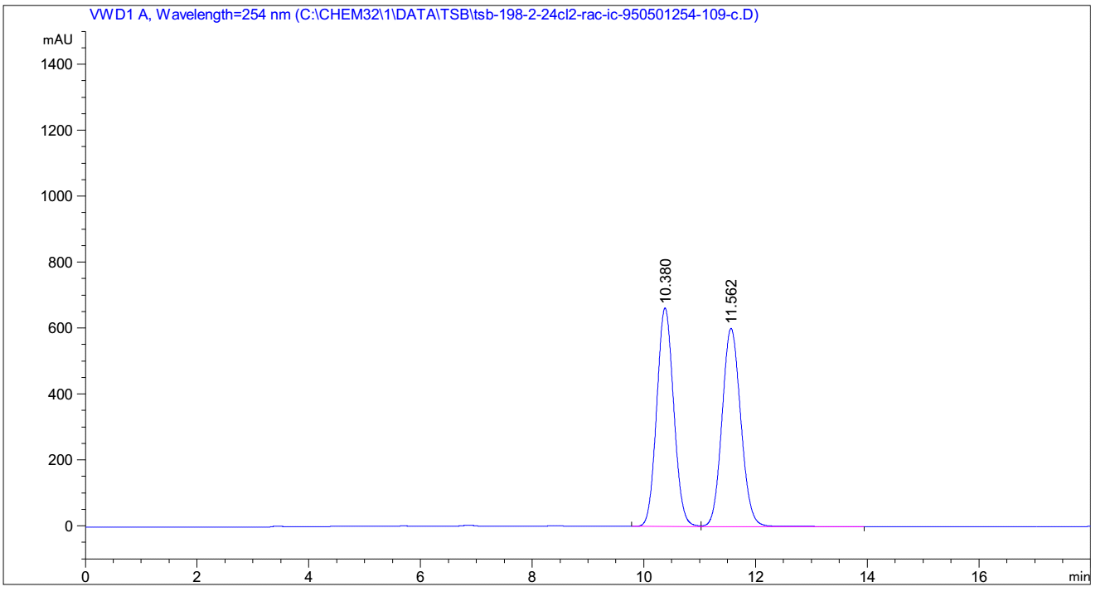

Peak Name	RT [min]	Type	width [min]	Area [mAU*s]	Height [mAu]	Area ratio %
1	10.380	BV	0.3291	1.39839e4	662.86511	49.9187
2	11.562	VB	0.3644	1.40294e4	600.90002	50.0813

Enantioenriched sample (-)-36:

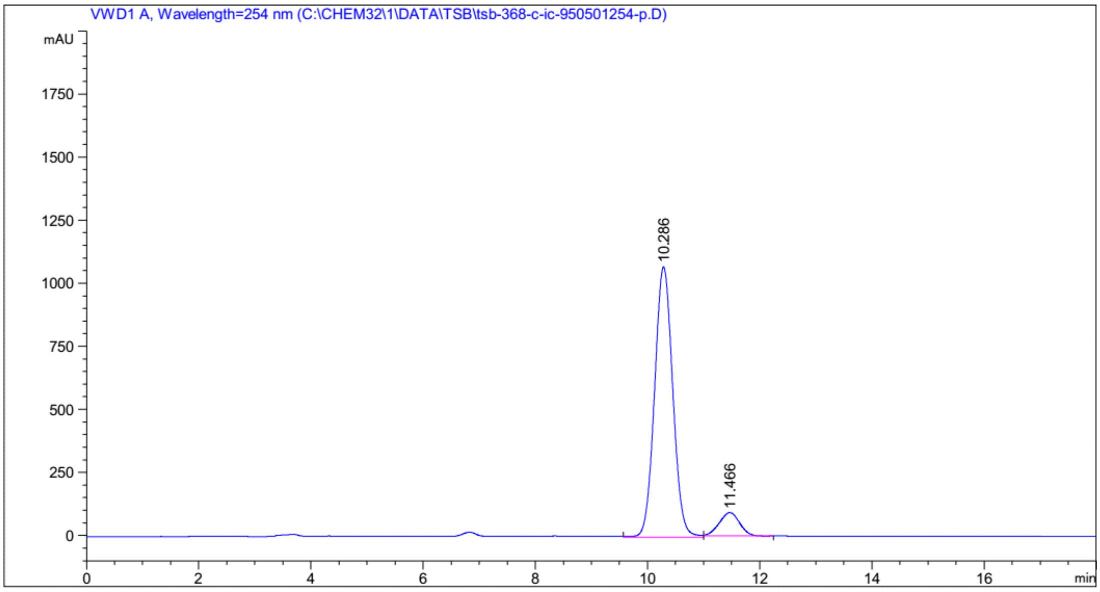

Peak Name	RT [min]	Type	width [min]	Area [mAU*s]	Height [mAu]	Area ratio %
1	10.286	MM	0.3753	2.41329e4	1071.80859	91.1530
2	11.466	MM	0.4206	2342.25684	92.81612	8.8470

Supplementary Figure 176. HPLC analysis of compound (-)-36.

Racemic sample 37: HPLC (Daicel Chiralpak IC column, hexane/iPrOH = 70:30, flow rate: 1.0 mL/min, λ= 254 nm)

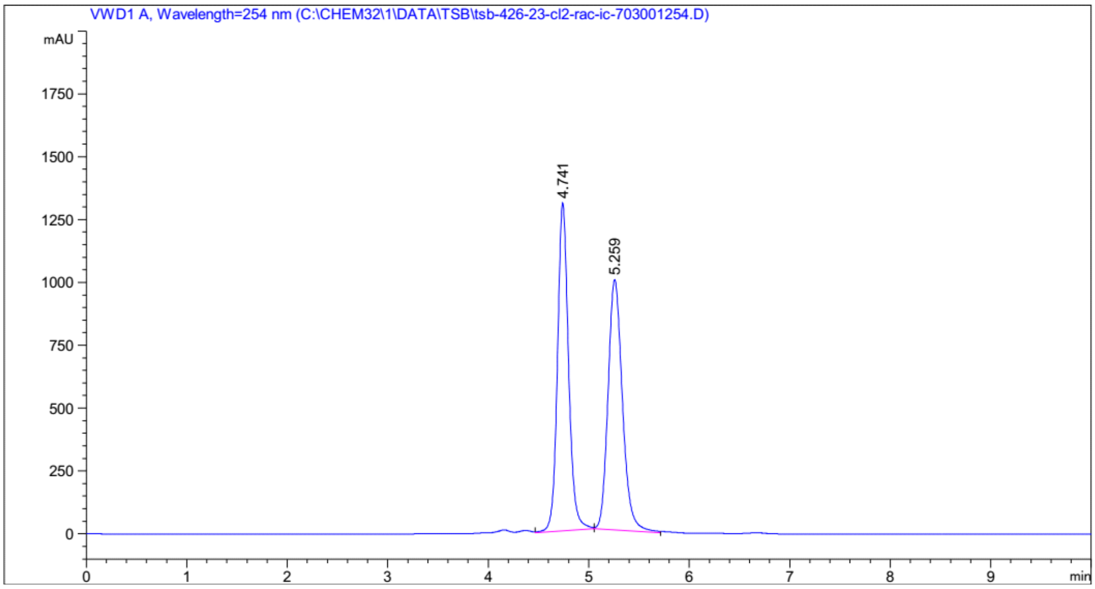

Peak Name	RT [min]	Type	width [min]	Area [mAU*s]	Height [mAu]	Area ratio %
1	4.741	MM	0.1271	9950.67676	1304.69104	50.1074
2	5.259	MM	0.1658	9908.03320	996.09015	49.8926

Enantioenriched sample (-)-37:

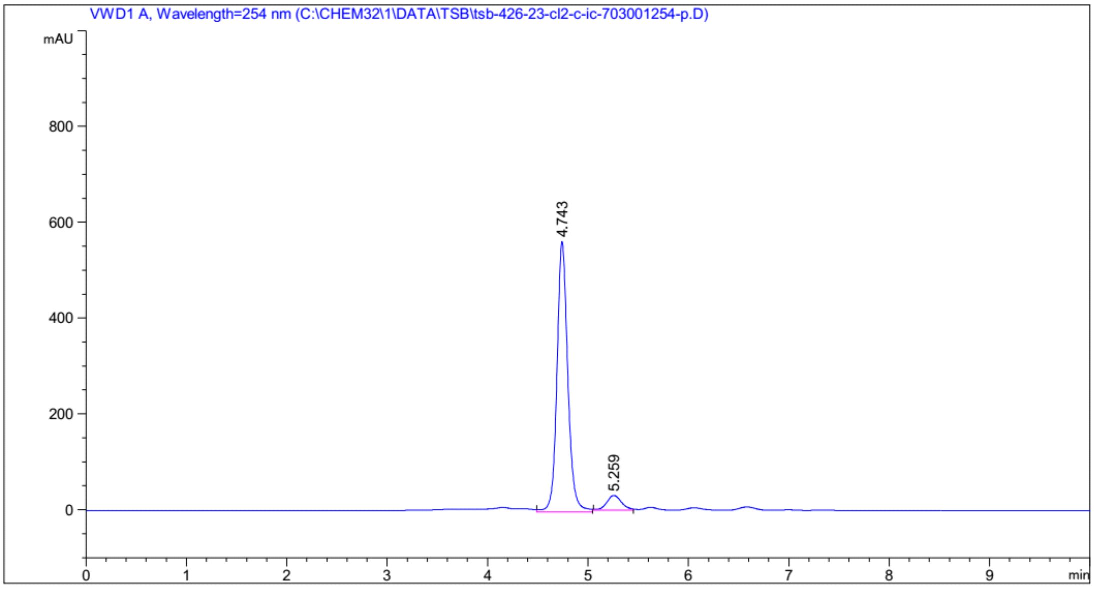

Peak Name	RT [min]	Type	width [min]	Area [mAU*s]	Height [mAu]	Area ratio %
1	4.743	MM	0.1249	4226.09033	564.10431	93.1094
2	5.259	MM	0.1700	312.75247	30.65715	6.8906

Supplementary Figure 177. HPLC analysis of compound (-)-37.

Racemic sample 38: HPLC (Daicel Chiralpak IC column, hexane/iPrOH = 70:30, flow rate: 1.0 mL/min, λ= 254 nm)

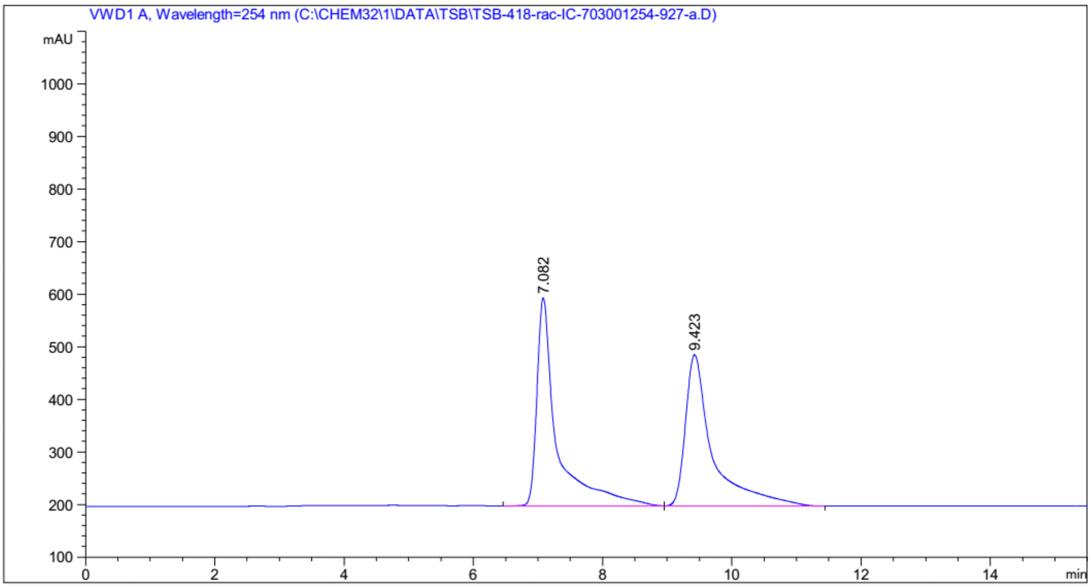

Peak Name	RT [min]	Type	width [min]	Area [mAU*s]	Height [mAu]	Area ratio %
1	7.082	BB	0.3036	8530.06641	395.50348	50.4864
2	9.423	BB	0.4156	8365.71094	287.39694	49.5136

Enantioenriched sample (-)-38:

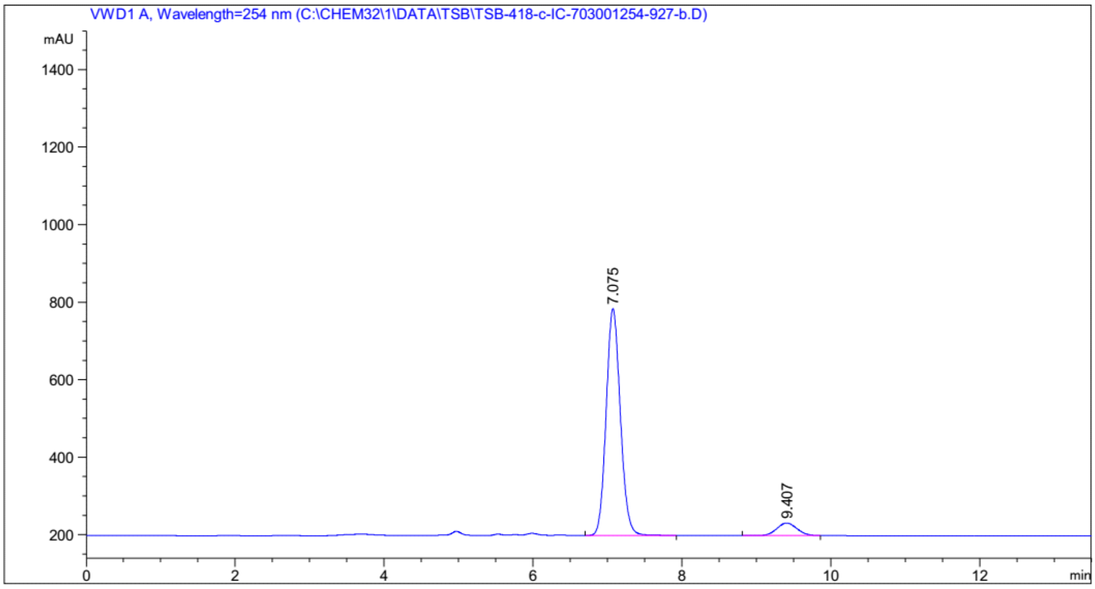

Peak Name	RT [min]	Type	width [min]	Area [mAU*s]	Height [mAu]	Area ratio %
1	7.075	BB	0.2058	7757.92188	584.84784	92.8534
2	9.407	MM	0.3097	597.09857	32.13320	7.1466

Supplementary Figure 178. HPLC analysis of compound (-)-38.

Racemic sample 39: HPLC (Daicel Chiralpak IC column, hexane/iPrOH = 60:40, flow rate: 1.0 mL/min, λ= 254 nm)

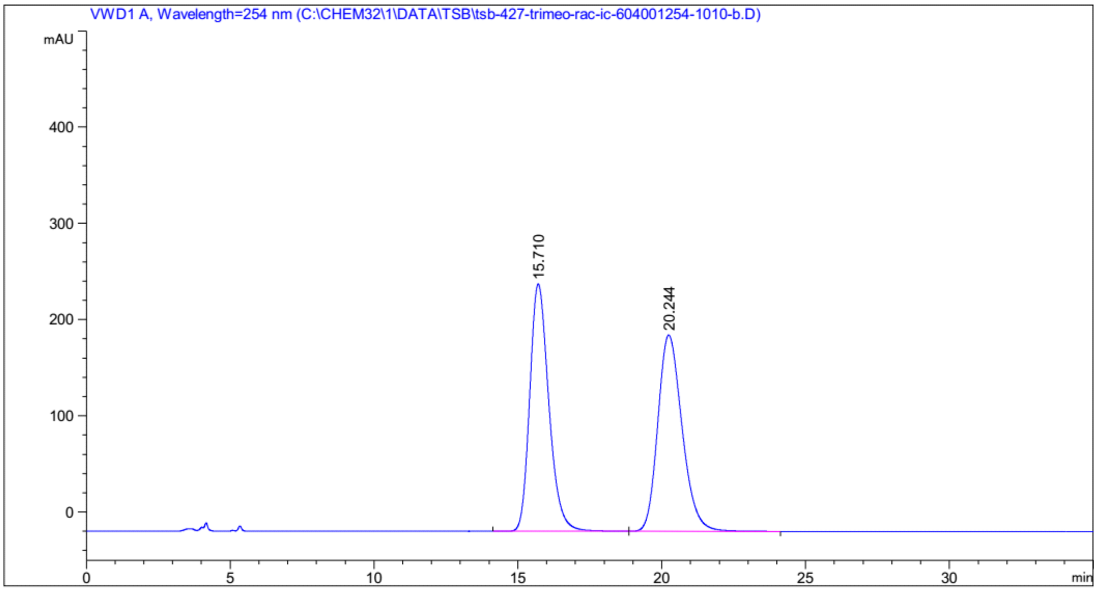

Peak Name	RT [min]	Type	width [min]	Area [mAU*s]	Height [mAu]	Area ratio %
1	15.710	BB	0.7095	1.18630e4	257.00473	49.8022
2	20.244	BB	0.9008	1.19573e4	204.01814	50.1978

Enantioenriched sample (-)-39:

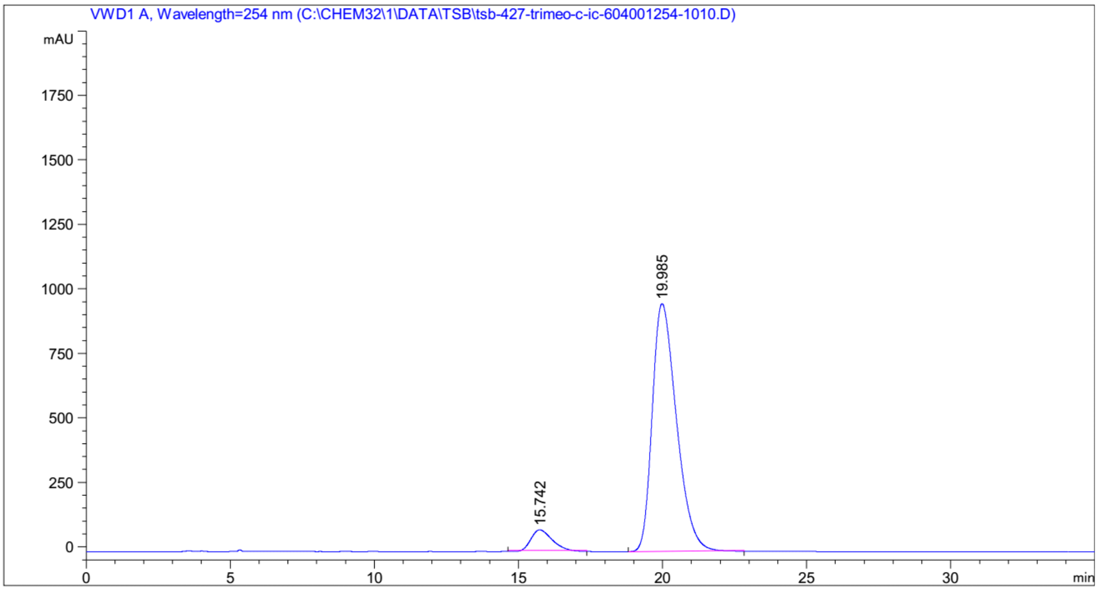

Peak Name	RT [min]	Type	width [min]	Area [mAU*s]	Height [mAu]	Area ratio %
1	15.742	MM	0.8085	3849.93945	79.36732	6.4252
2	19.985	MM	0.9725	5.60690e4	960.85956	93.5748

Supplementary Figure 179. HPLC analysis of compound (-)-39.

Racemic sample 40: HPLC (Daicel Chiralpak IA column, hexane/EtOH = 70:30, flow rate: 1.0 mL/min, λ= 254 nm)

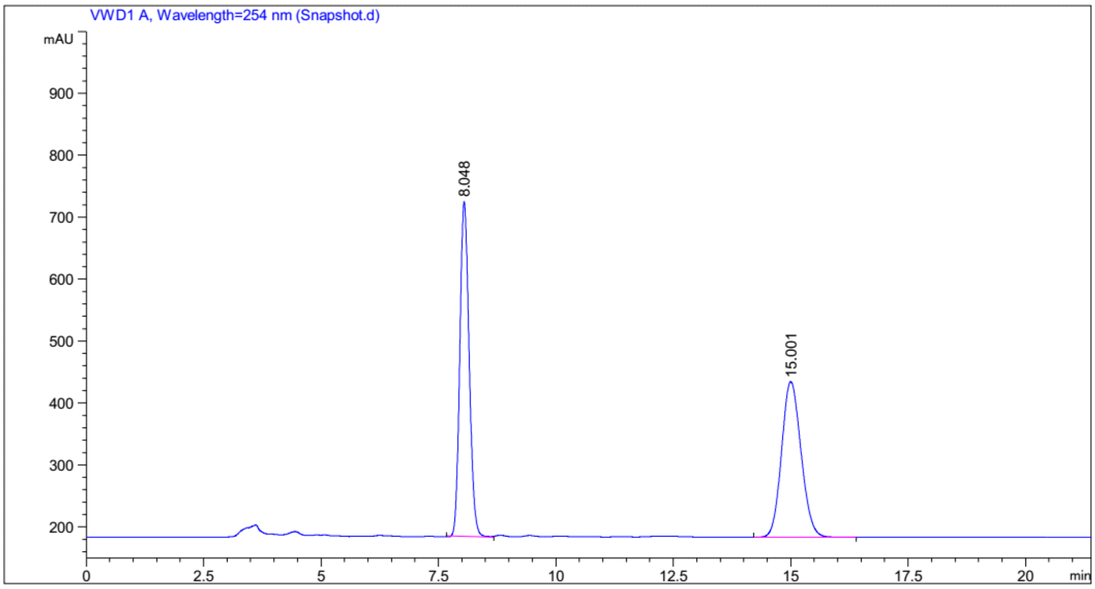

Peak Name	RT [min]	Type	width [min]	Area [mAU*s]	Height [mAu]	Area ratio %
1	8.048	MM	0.2298	7449.25244	540.35718	50.7090
2	15.001	BB	0.4492	7240.95898	251.24245	49.2910

Enantioenriched sample (-)-40

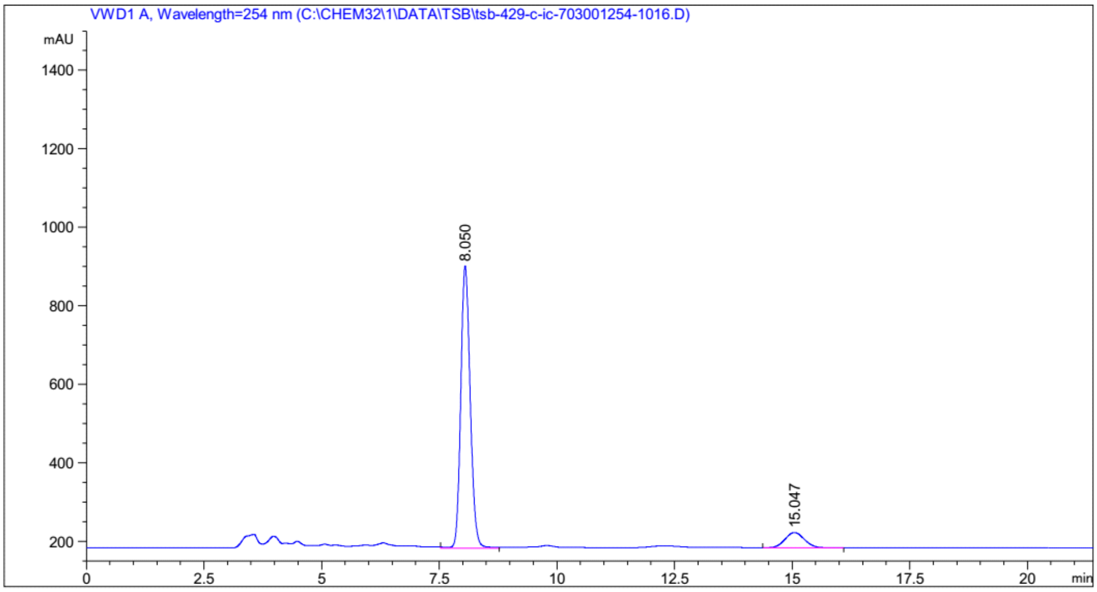

Peak Name	RT [min]	Type	width [min]	Area [mAU*s]	Height [mAu]	Area ratio %
1	8.050	MM	0.2338	1.01016e4	720.08295	90.0467
2	15.047	BB	0.4456	1116.57825	39.04440	9.9533

Supplementary Figure 180. HPLC analysis of compound (-)-40.

Racemic sample 41: HPLC (Daicel Chiralpak ID column, hexane/iPrOH = 70:30, flow rate: 1.0 mL/min, λ= 254 nm)

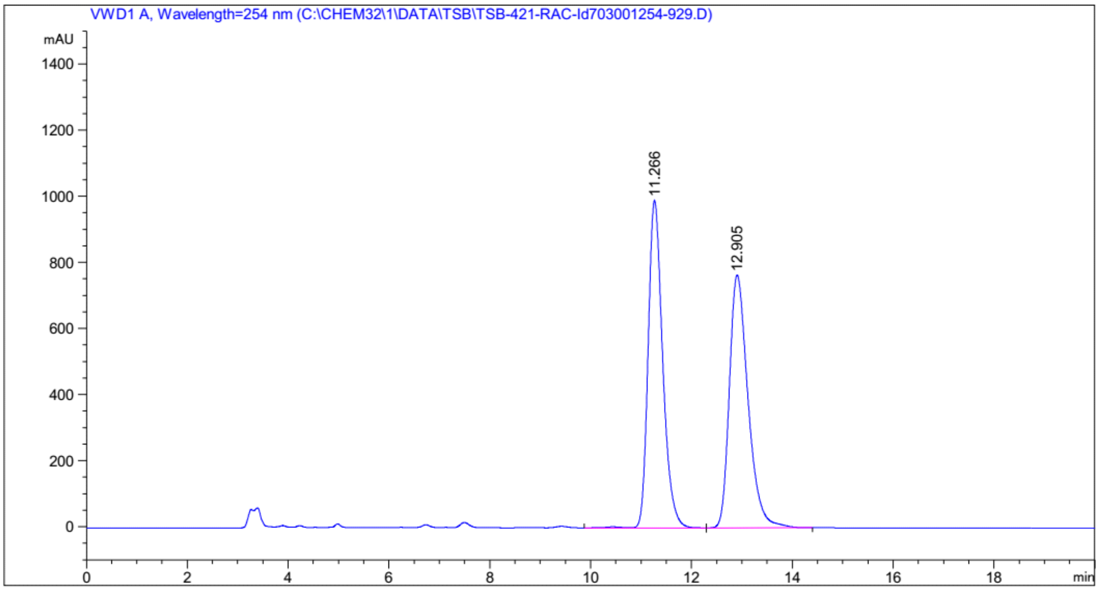

Peak Name	RT [min]	Type	width [min]	Area [mAU*s]	Height [mAu]	Area ratio %
1	11.266	VB R	0.3057	1.98276e4	990.70697	50.2346
2	12.905	BB	0.3916	1.96425e4	764.79230	49.7654

Enantioenriched sample (-)-41:

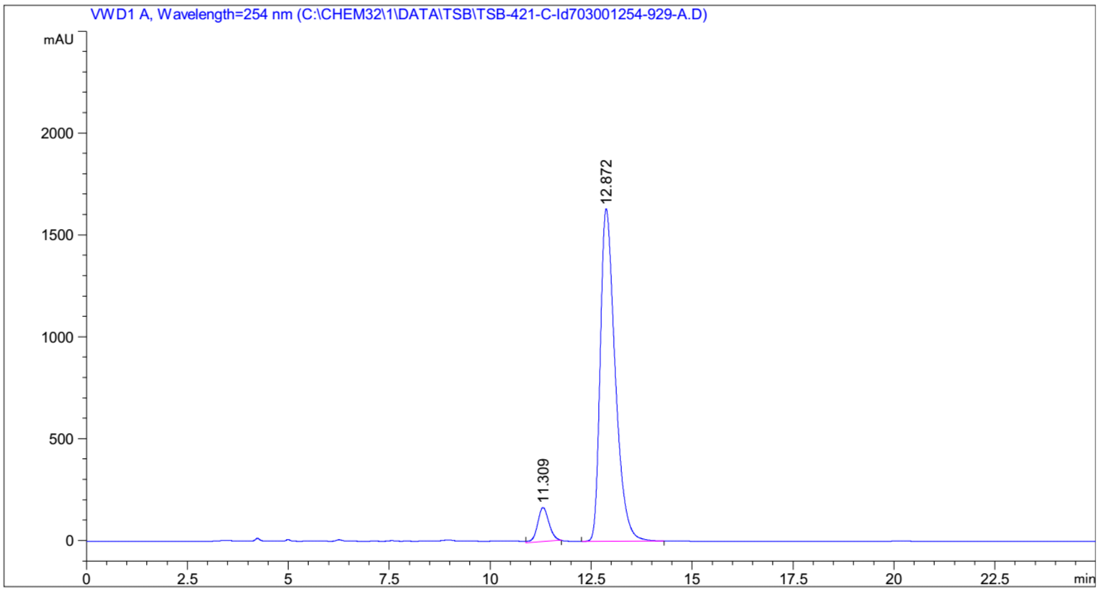

Peak Name	RT [min]	Type	width [min]	Area [mAU*s]	Height [mAu]	Area ratio %
1	11.309	MM	0.3245	3249.69629	166.89886	7.2055
2	12.872	BB	0.3913	4.18506e4	1631.29175	92.7945

Supplementary Figure 181. HPLC analysis of compound (-)-41.

Racemic sample 42: HPLC (Daicel Chiralpak IA column (hexane/iPrOH = 70:30, flow rate: 1.0 mL/min, λ= 254 nm)

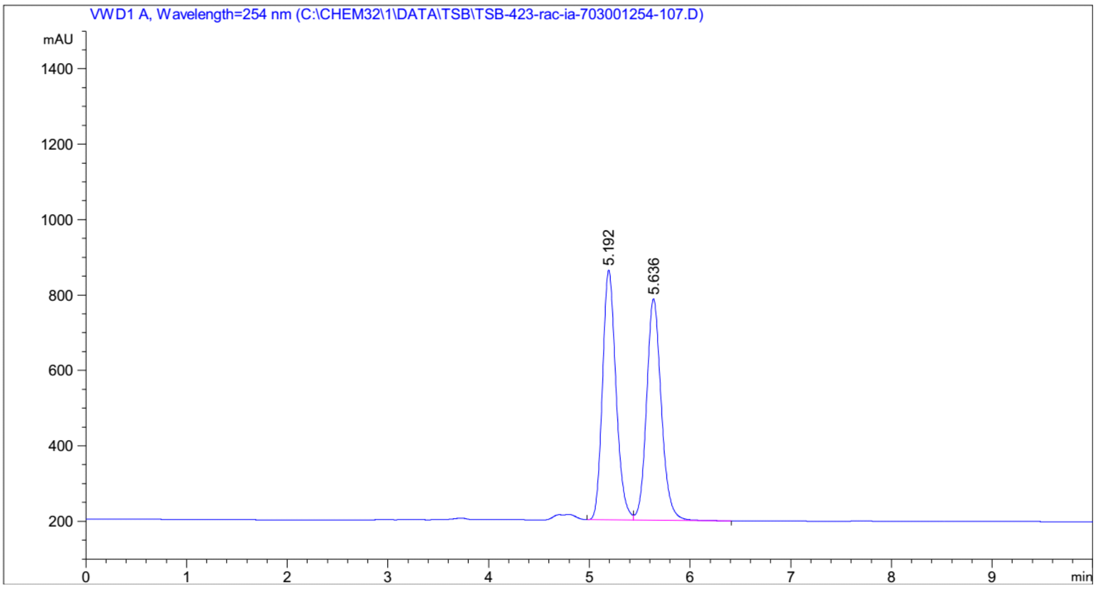

Peak Name	RT [min]	Type	width [min]	Area [mAU*s]	Height [mAu]	Area ratio %
1	5.192	BV	0.1438	6179.61328	662.87085	50.8074
2	5.636	VB	0.1558	5983.21240	587.57269	49.1926

Enantioenriched sample(-)-42:

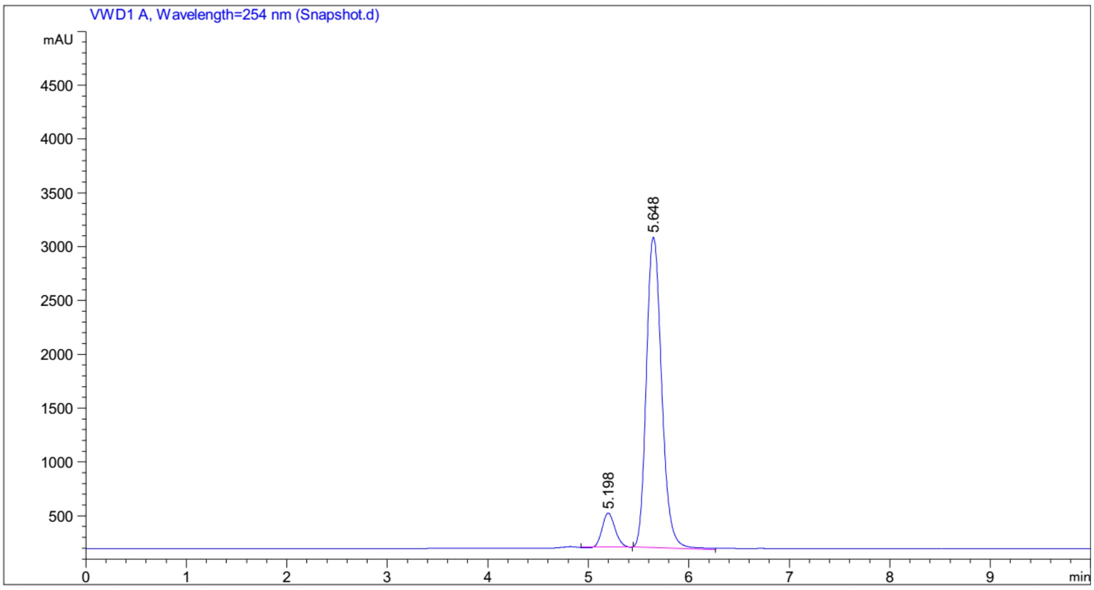

Peak Name	RT [min]	Type	width [min]	Area [mAU*s]	Height [mAu]	Area ratio %
1	5.198	MM	0.1456	2737.82837	313.32254	8.2442
2	5.648	MM	0.1763	3.04711e4	2881.19409	91.7558

Supplementary Figure 182. HPLC analysis of compound (-)-42.

Racemic sample 43: HPLC (Daicel Chiralpak IC column, hexane/iPrOH = 70:30, flow rate: 1.0 mL/min, λ= 254 nm)

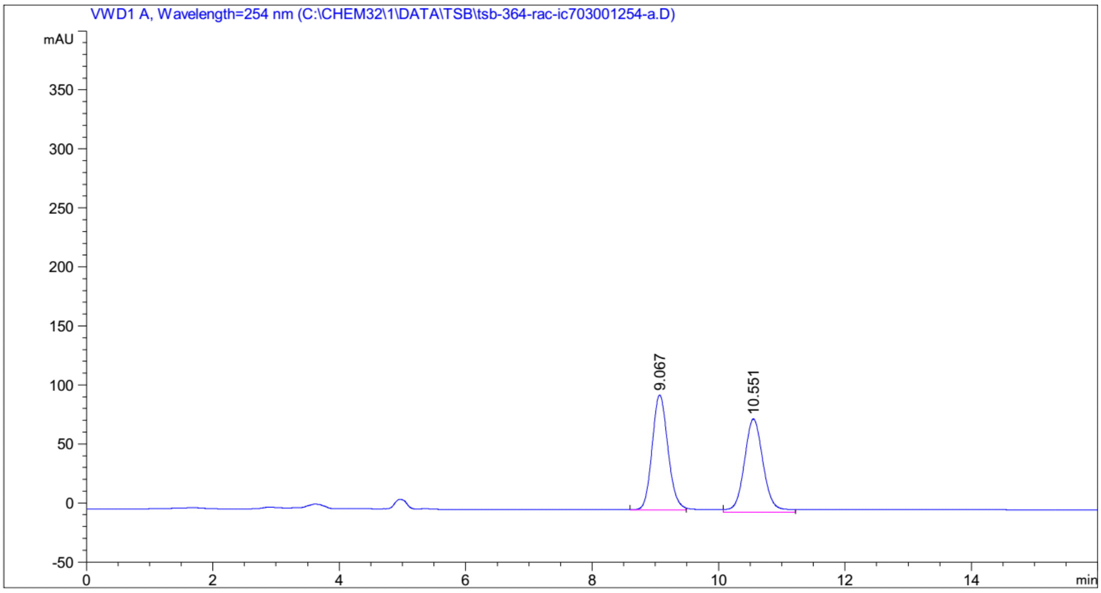

Peak Name	RT [min]	Type	width [min]	Area [mAU*s]	Height [mAu]	Area ratio %
1	9.067	MM	0.2893	1691.78796	97.48120	50.1658
2	10.551	MM	0.3537	1680.60278	79.19607	49.8342

Enantioenriched sample (-)-43:

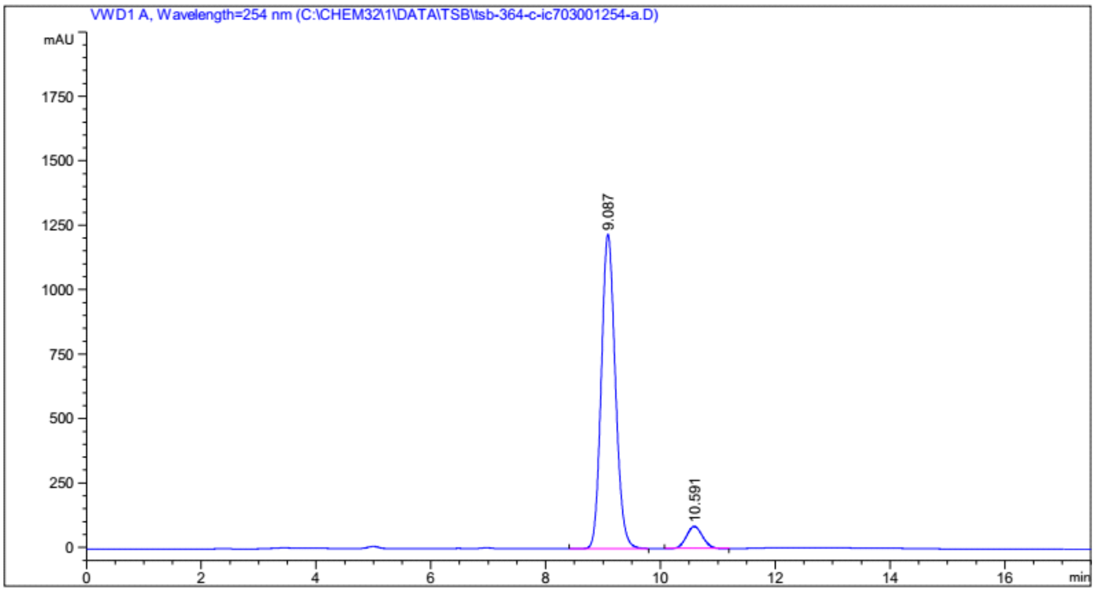

Peak Name	RT [min]	Type	width [min]	Area [mAU*s]	Height [mAu]	Area ratio %
1	9.087	MM	0.2837	2.07694e4	1220.14514	92.7404
2	10.591	MM	0.3195	1625.81067	84.81387	7.2596

Supplementary Figure 183. HPLC analysis of compound (-)-43.

Racemic sample 36': HPLC (Daicel Chiralpak IC column, hexane/iPrOH = 95:05, flow rate: 1.0 mL/min, λ= 254 nm)

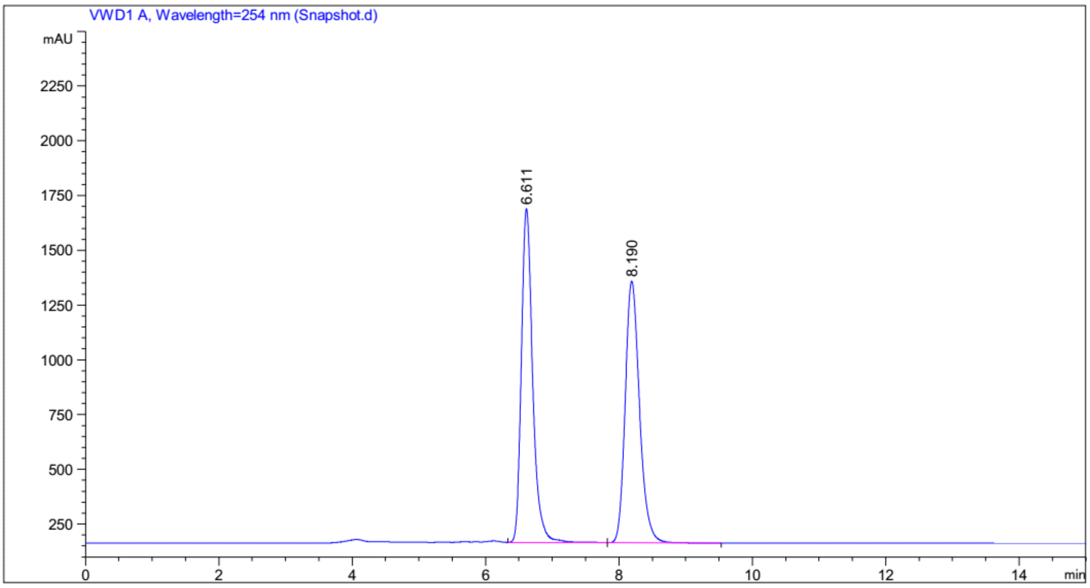

Peak Name	RT [min]	Type	width [min]	Area [mAU*s]	Height [mAu]	Area ratio %
1	6.611	BV R	0.1791	1.79332e4	1524.26758	50.2327
2	8.190	BB	0.2284	1.77671e4	1195.23157	49.7673

Enantioenriched sample (-)-36':

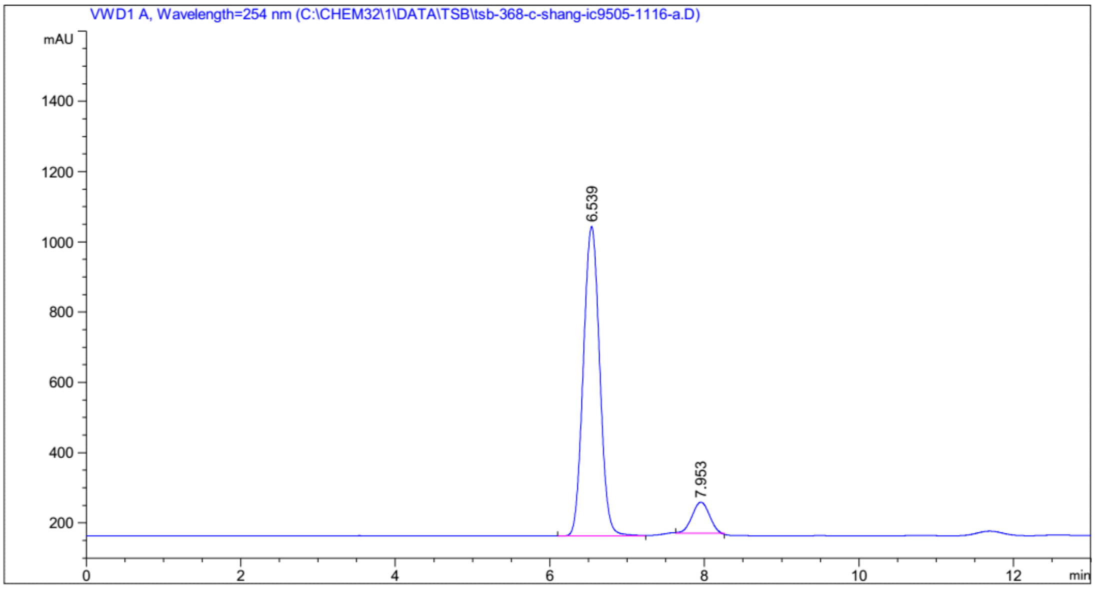

Peak Name	RT [min]	Type	width [min]	Area [mAU*s]	Height [mAu]	Area ratio %
1	6.539	BV	0.2322	1.29093e4	879.83276	90.4366
2	7.953	MM	0.2582	1365.11462	88.13373	9.5634

Supplementary Figure 184. HPLC analysis of compound (-)-36'.

Racemic sample 50: HPLC (Daicel Chiralpak IC column, hexane/iPrOH = 90:10, flow rate: 1.0 mL/min, λ= 254 nm)

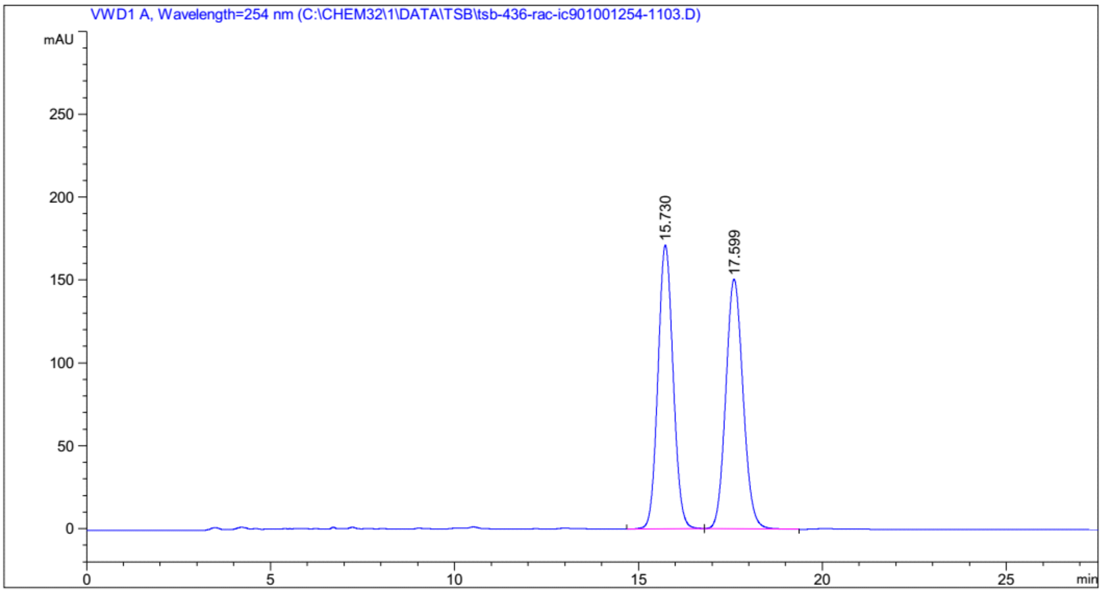

Peak Name	RT [min]	Type	width [min]	Area [mAU*s]	Height [mAu]	Area ratio %
1	15.730	BB	0.4463	4903.78467	171.15587	49.9644
2	17.599	BB	0.5066	4910.77490	150.55002	50.0356

Enantioenriched sample (-)-50:

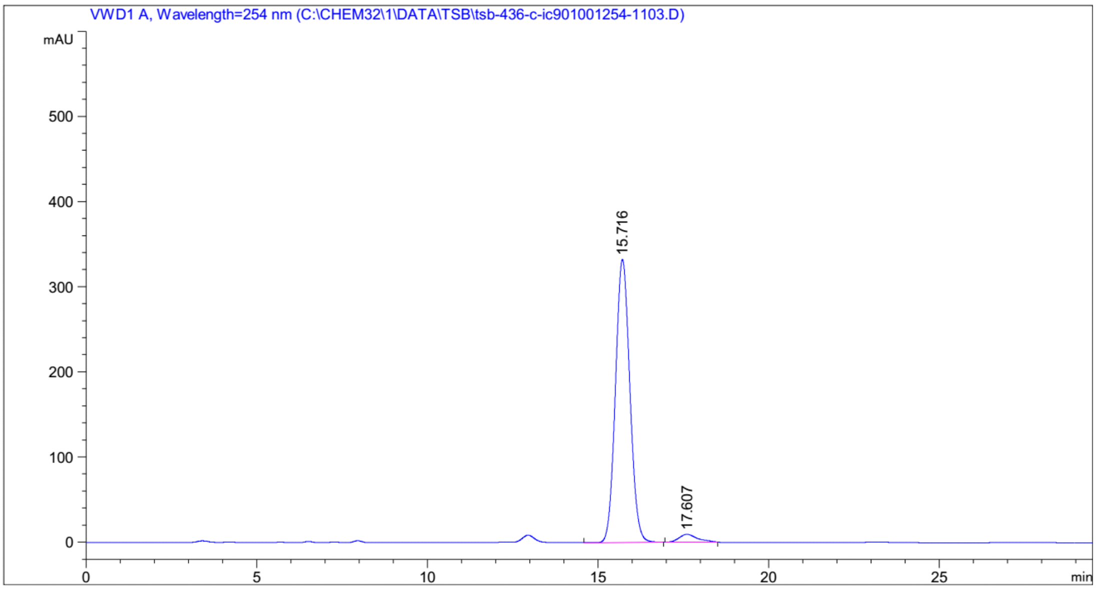

Peak Name	RT [min]	Type	width [min]	Area [mAU*s]	Height [mAu]	Area ratio %
1	15.716	MM	0.4888	9751.17383	332.49332	96.7835
2	17.607	MM	0.6014	324.06589	8.98067	3.2165

Supplementary Figure 185. HPLC analysis of compound (-)-50.

Racemic sample 51: HPLC (Daicel Chiralpak IC column, hexane/iPrOH = 95:05, flow rate: 1.0 mL/min, λ= 254 nm)

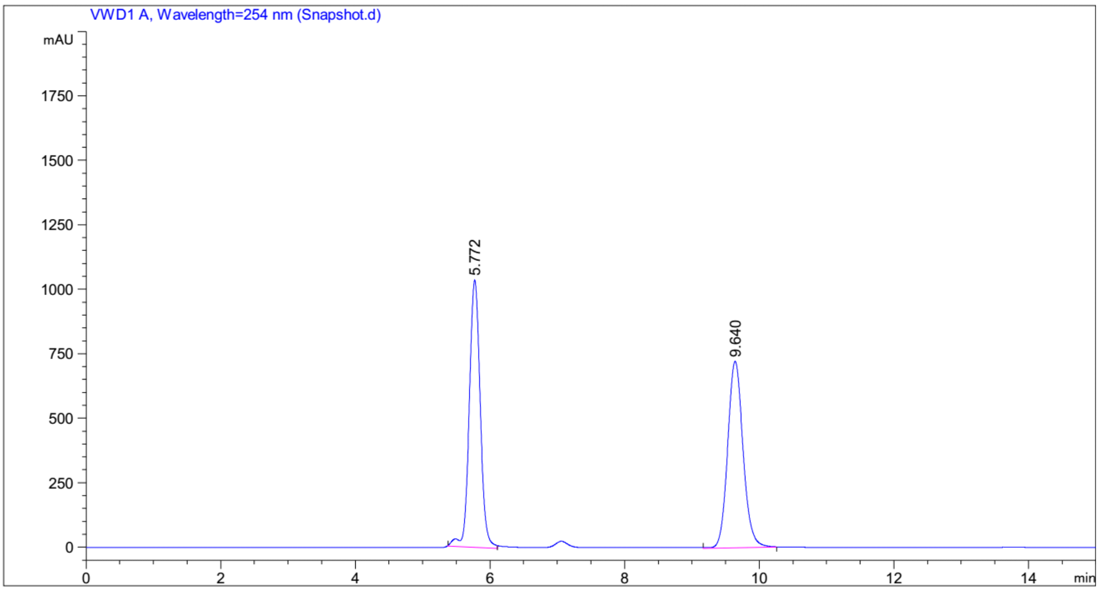

Peak Name	RT [min]	Type	width [min]	Area [mAU*s]	Height [mAu]	Area ratio %
1	5.772	MM	0.1882	1.17004e4	1035.94653	50.4287
2	9.640	MM	0.2649	1.15014e4	723.70532	49.5713

Enantioenriched sample (-)-51:

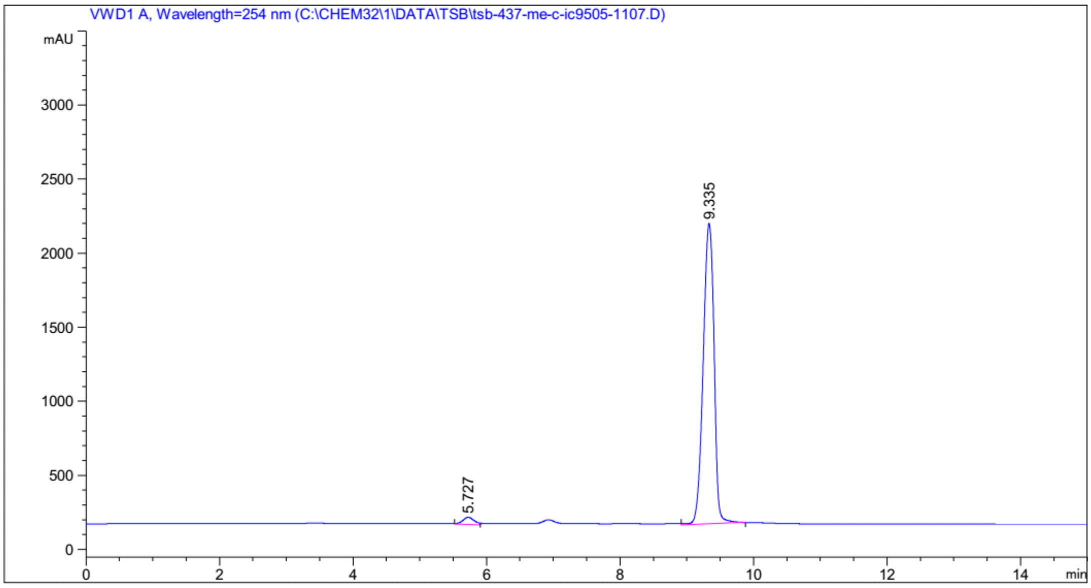

Peak Name	RT [min]	Type	width [min]	Area [mAU*s]	Height [mAu]	Area ratio %
1	5.727	MM	0.2028	599.93597	49.30097	2.5463
2	9.335	MM	0.1887	2.29611e4	2027.77673	97.4537

Supplementary Figure 186. HPLC analysis of compound (-)-51.

Racemic sample 52: HPLC (Daicel Chiralpak IC column, hexane/iPrOH = 50:50, flow rate: 1.0 mL/min, λ= 254 nm)

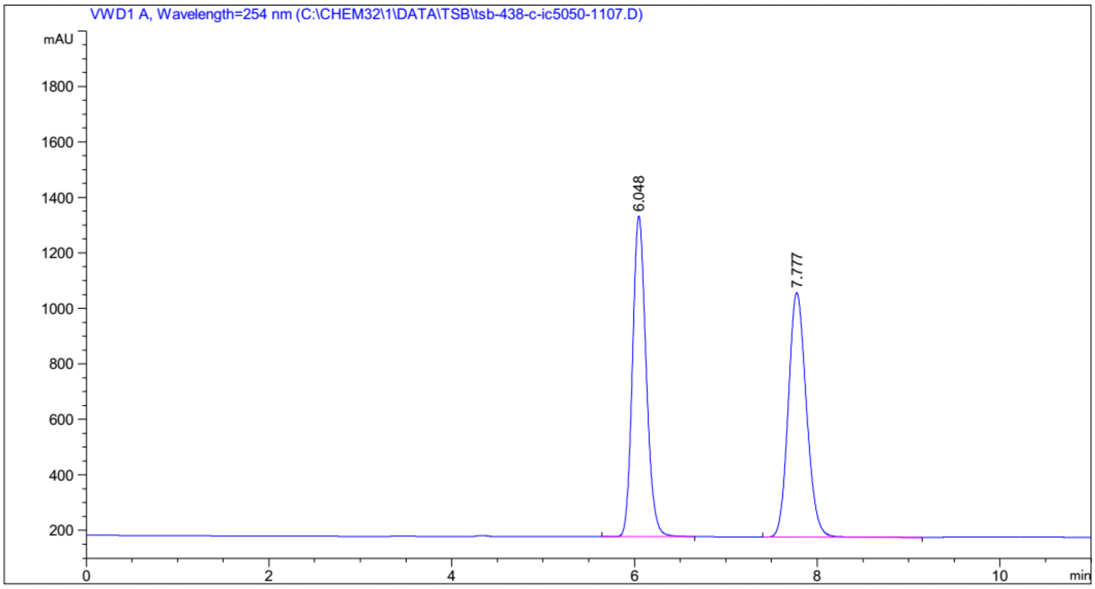

Peak Name	RT [min]	Type	width [min]	Area [mAU*s]	Height [mAu]	Area ratio %
1	6. 048	BB	0. 1613	1. 20167e4	1155. 45740	49. 7907
2	7. 777	BB	0. 2134	1. 21177e4	881. 27106	50. 2093

Enantioenriched sample (-)-52:

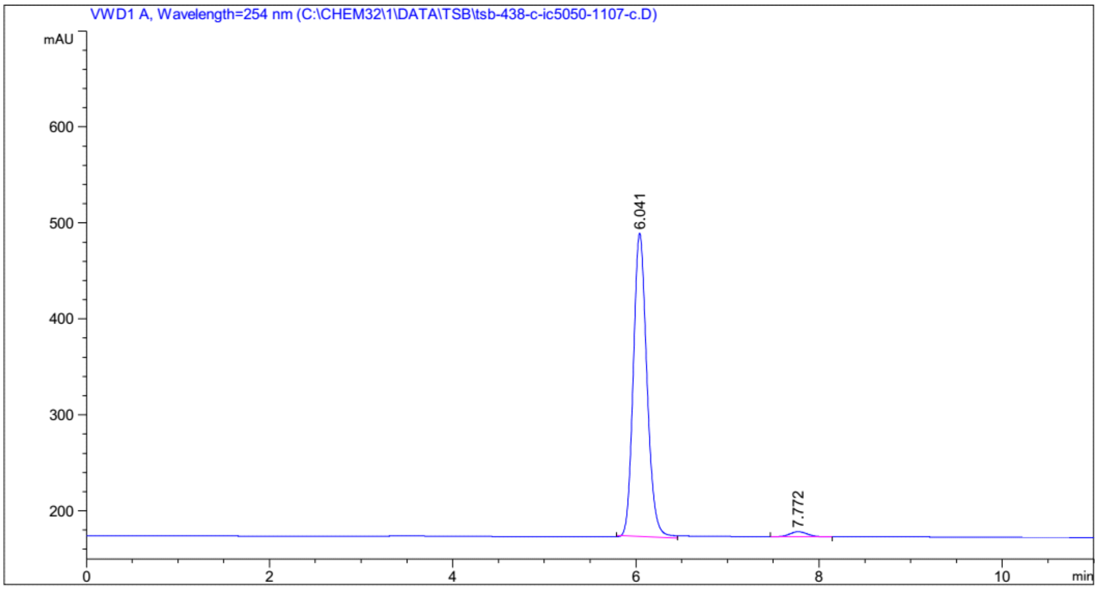

Peak Name	RT [min]	Type	width [min]	Area [mAU*s]	Height [mAu]	Area ratio %
1	6. 041	MM	0. 1725	3271. 26929	316. 14111	97. 8878
2	7. 772	BB	0. 2083	70. 58652	5. 26785	2. 1122

Supplementary Figure 187. HPLC analysis of compound (-)-52.

Supplementary References

1. Zhu, S.-F., Chen, C., Cai, Y. & Zhou, Q.-L. Catalytic Asymmetric reaction with water: enantioselective synthesis of α-hydroxyesters by a copper–carbenoid O-H insertion reaction. *Angew. Chem. Int. Ed.* **47**, 932-934 (2008).
2. Zhu, S.-F., Cai, Y., Mao, H.-X., Xie, J.-H. & Zhou, Q.-L. Enantioselective iron-catalysed O–H bond insertions. *Nat. Chem.*, **2**, 546-551 (2010).
3. Maier, T. C. & Fu, G. C. Catalytic enantioselective O−H insertion reactions, *J. Am. Chem. Soc.* **128**, 4594-4595 (2006).
4. Davies, H. M. L. & Townsend, R. J. Catalytic asymmetric cyclopropanation of heteroaryldiazoacetates. *J. Org. Chem.* **66**, 6595-6603 (2001).
5. Hari, D. P. & Waser, J. Copper-catalyzed oxy-alkynylation of diazo compounds with hypervalent iodine reagents. *J. Am. Chem. Soc.* **138**, 2190-2193 (2016).
6. Pospech, J., Lennox, A. J. J.& Beller, M. Rhodium-catalysed alkoxylation/acetalization of diazo compounds: one-step synthesis of highly functionalised quaternary carbon centres. *Chem. Commun.*, **51**, 14505-14508 (2015).
7. Ye, F., Wang, C., Zhang, Y. & Wang, J. Synthesis of aryldiazoacetates through palladium(0)-catalyzed deacylative cross-coupling of aryl iodides with acyldiazoacetates. *Angew. Chem. Int. Ed.* **53**, 11625-11628 (2014).
8. Lee, E. C. & Fu, G. C. Copper-Catalyzed Asymmetric N−H Insertion Reactions: Couplings of Diazo Compounds with Carbamates to Generate α-Amino Acids. *J. Am. Chem. Soc.* **129**, 12066-12067 (2007).
9. Chen, G., Song, J., Yu, Y., Luo, X., Li, C. & Huang, X. Aminofluorination: transition-metal-free N–F bond insertion into diazocarbonyl compounds. *Chem. Sci*. **7**, 1786-1790 (2016).
10. Kidonakis, M. & Stratakis, M. Au Nanoparticle-catalyzed insertion of carbenes from α-diazocarbonyl compounds into hydrosilanes. *Org. Lett*. **20**, 4086-4089 (2018).
11. Oh, J.-S., Lee, J.-W., Ryu, T. H., Lee, J. H. & Song, C. E. Self-association free bifunctional thiourea organocatalysts: synthesis of chiral α-amino acids via dynamic kinetic resolution of racemic azlactones. *Org. Biomol. Chem.*, **10**, 1052-1055 (2012).
12. Huang, H., Konda, S. & Zhao, J. C.-G. Diastereodivergent catalysis using modularly designed organocatalysts: synthesis of both cis- and trans-fused pyrano[2,3-b]pyrans. *Angew. Chem. Int. Ed.* **55**, 2213-2216 (2016).
13. Ye, J., Dixon, D. J. & Hynes, P. S. Enantioselective organocatalytic Michael addition of malonate esters to nitro olefins using bifunctional cinchonine derivatives. *Chem. Commun.* 4481-4483 (2005).
14. Bürki, C., Whyte, A., Arndt, S., Hashmi, A. S. K. & Lautens, M. Expanding the scope of the gold(I)-catalyzed rautenstrauch rearrangement: protic additives. *Org. Lett.* **18**, 5058-5061 (2016).
15. Dai, H., Yu, S., Cheng, W., Xu, Z.-F. & Li, C.-Y. Rhodium-catalyzed synthesis of 1,2-dihydropyridine by a tandem reaction of 4-(1-acetoxyallyl)-1-sulfonyl-1,2,3-triazole. *Chem. Commun.* **53**, 6417-6420 (2017).
16. Wu, Y.-K. & West, F. G. Brønsted acid-mediated Nazarov cyclization of vinylallenes. *J. Org. Chem.* **75**, 5410-5413 (2010).
17. Marson, C. M., Edaan, E., Morrell, J. M., Coles, S. J., Hursthouse, M. B. & Davies, D. T. A catalytic asymmetric protocol for the enantioselective synthesis of 3(2H)-furanones. *Chem. Commun.* 2494-2496 (2007).
